# Evolution of the functionally conserved *DCC* gene in birds

**DOI:** 10.1038/srep42029

**Published:** 2017-02-27

**Authors:** Cedric Patthey, Yong Guang Tong, Christine Mary Tait, Sara Ivy Wilson

**Affiliations:** 1 Umeå Center for Molecular Medicine, Umeå University, 901-87 Umeå, Sweden

## Abstract

Understanding the loss of conserved genes is critical for determining how phenotypic diversity is generated. Here we focus on the evolution of *DCC*, a gene that encodes a highly conserved neural guidance receptor. Disruption of *DCC* in animal models and humans results in major neurodevelopmental defects including commissural axon defects. Here we examine *DCC* evolution in birds, which is of particular interest as a major model system in neurodevelopmental research. We found the *DCC* containing locus was disrupted several times during evolution, resulting in both gene losses and faster evolution rate of salvaged genes. These data suggest that *DCC* had been lost independently twice during bird evolution, including in chicken and zebra finch, whereas it was preserved in many other closely related bird species, including ducks. Strikingly, we observed that commissural axon trajectory appeared similar regardless of whether *DCC* could be detected or not. We conclude that the *DCC* locus is susceptible to genomic instability leading to independent disruptions in different branches of birds and a significant influence on evolution rate. Overall, the phenomenon of loss or molecular evolution of a highly conserved gene without apparent phenotype change is of conceptual importance for understanding molecular evolution of key biological processes.

Increasing lines of evidence suggest that core genes that are regarded as ‘highly conserved’ can be absent from selected species^1^,^2^,^3^,^4^,^5^,^6^. This phenomenon is of importance for understanding the molecular evolution of pathways underlying key biological processes and the evolution of animal characteristics. In addition this has significant implications for choice of comparative experimental model systems for examining particular biological questions. Here we focus on the developing nervous system, which has numerous well-studied molecular and anatomical features that are conserved across bilaterian animals and therefore provides a good model system to study molecular evolution. For example, the main signalling pathways regulating neural guidance are broadly conserved throughout evolution^7^,^8^. Moreover, several equivalent neuroanatomical characteristics are conserved widely between diverse species as exemplified by commissural neurons that evolved at the emergence of bilateral symmetry^9^. These neurons facilitate communication of left-right neural information permitting sophisticated movement strategies and bilateral integration of sensory information within the body.

Commissural axons develop under the influence of the Netrin-1 ligand and DCC receptor in a wide evolutionary span of animals^10^,^11^,^12^,^13^,^14^,^15^,^16^,^17^,^18^,^19^. More broadly, Netrin/DCC signalling is a central feature of a wide range of neuronal and non-neuronal developmental processes in addition to adult physiological and pathological processes in a wide spectrum of animals^18^,^20^,^21^,^22^,^23^,^24^,^25^,^26^,^27^,^28^,^29^,^30^,^31^,^32^,^33^,^34^,^35^,^36^. However, despite DCC being such a highly conserved and fundamental component of major biological processes, a recent study suggested that chickens do not possess a *DCC* gene^37^. This is striking since loss of *DCC* in other animal models results in severe commissural neuron defects and lethality, but in chicken, commissural axons do not have this phenotype^11^,^37^. Moreover, genuine loss of the *DCC* gene from chicken could provide a platform for understanding more generally how loss of a core genetic mechanism is compensated for or what consequences arise.

Armed with the latest genomic and transcriptomic data available we therefore aimed to clarify the *DCC* status during bird evolution. We found that *DCC* is either lost or derived to a form that is undetectable in a branch of Galliformes including chicken, turkey, guineafowl and quail. This happened in conjunction with an inversion at the *DCC* containing locus. This disruption happened independently in a different branch of birds, subsets of Passeriformes, zebra finch and ground finch. Strikingly, despite the fact that *DCC* could not be detected in subsets of Galliformes and Passeriformes, we found that *DCC* is preserved in most bird species examined including duck and basal birds. Our data suggests that genes salvaged from the original disrupted locus, including in a few cases *DCC*, were evolving significantly faster than orthologous genes in species where the original locus stayed intact. Moreover, developing commissural axons appeared similar in mouse, chicken, zebra finch and duck despite the apparent loss of *DCC* in chicken and zebra finch. Overall, these data highlight an example of a gene that has a high level of sequence and functional conservation between different animal taxa yet cannot be detected in discrete subsets of bird species.

## Results

### Analysis of bird genomic assemblies revealed that *DCC* is present in diverse bird families

Most vertebrate genomes contain two genes that share high sequence similarity and domain composition, *DCC* and *Neogenin*. Molecular phylogenetics and conservation of synteny between the human *DCC* and *Neogenin* loci suggest a duplication of the ancestral gene in conjunction with a whole genome duplication at the root of vertebrates 450–550 million years ago (Fig. S1, Supplementary File S1)^38^.

Since clarification of the *DCC* status is critical to the developmental biology field, especially in common experimental model systems such as chicken embryos, we first addressed the presence of *DCC* in birds in an evolutionary context by taking advantage of recently available genomic data. We focused this analysis on reptiles and birds and mined genome assemblies to examine the gene complement in each species (referencing in Supplementary File 1)^39^,^40^,^41^,^42^. In these analyses we included the latest chicken genome build (*Gallus gallus* 5.0) which has been developed to address the apparent lack of widely conserved genes in previous versions. Both *DCC* and *Neogenin* are highly similar to their respective orthologs in other species, which is an important feature in identifying them since a wide range of more distantly related homologues to *DCC* and *Neogenin* can be identified in most animals. Therefore, we initially used a high stringency screen for the *DCC* and *Neogenin* genes within existing genomic assemblies of different birds. We observed that all species examined (including members of the Galliformes, Anseriformes, Cuculiformes Caprimulgiformes, Apodiformes, Opisthocomiformes, Chradriiformes, Sphenisciformes, Falconiformes, Psittaciforme, Passeriformes, Struthioniformes and crocodilian species) contained a *Neogenin* gene.

However, using existing annotations of *DCC* sequences as bait, the top hit corresponded to the *Neogenin* gene in the Galliformes (chick, quail, turkey, guinea fowl) and Passeriformes (rifleman, ground finch, ground tit, zebra finch) (Table 1). This raised the possibility that *DCC* was lost in these species. Importantly, *DCC* was preserved in the closely related duck (Anseriformes, sister group of Galliformes) and budgerigar (Psitaciformes, sister group of Passeriformes) in addition to a number of more distantly related bird species including rock pigeon, common cuckoo, chimney swift, hoazin, killdeer, emperor penguin and peregrine falcon (Table 1). Since *DCC* was also found in ostrich and alligator this suggested the possibility of a single loss of *DCC* in the chicken lineage at the base of Galliformes and another independent loss in the Passeriformes lineage.

**Figure 1:**
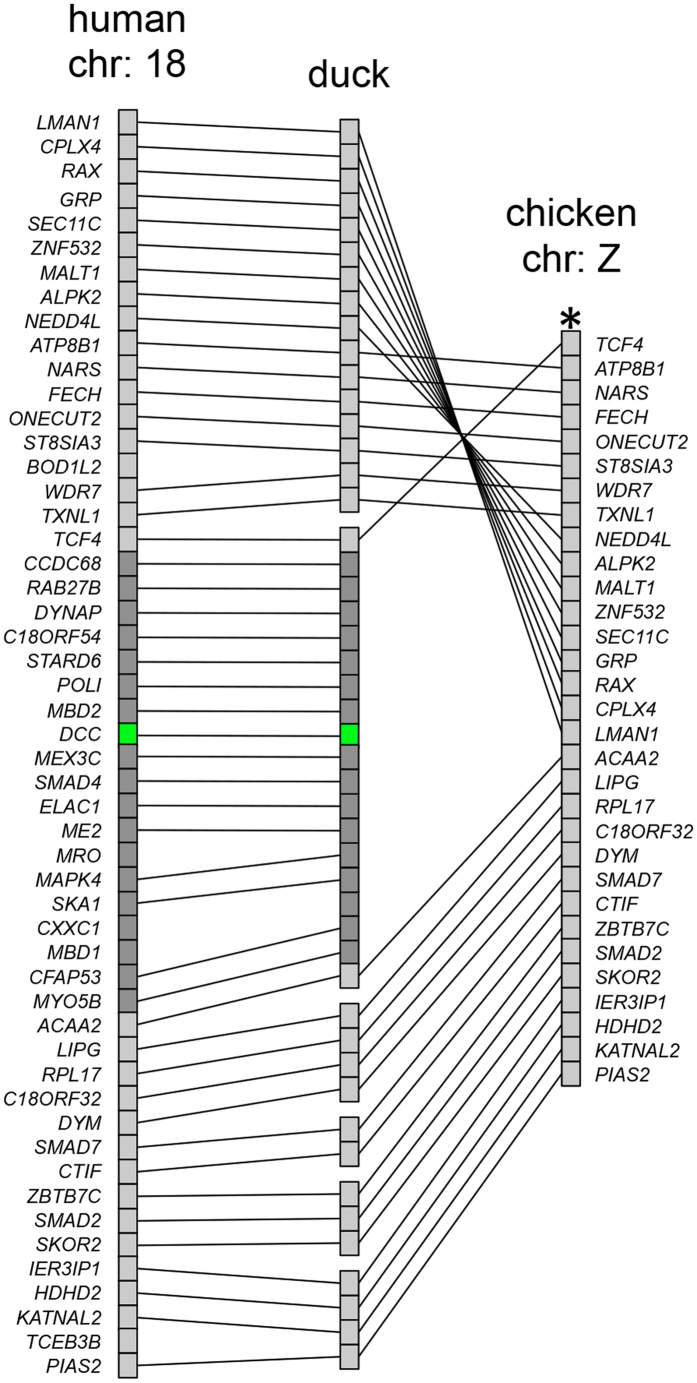
*DCC* was lost in conjunction with an inversion and deletion of the locus on the Z chromosome of Galliformes. The synteny of the *DCC* containing locus in human chromosome 18 is shown. Each individual gene is indicated with a rectangle (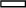). The *DCC* gene is indicated with a green rectangle (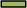) whereas other genes are shown in grey (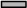). The synteny at the equivalent locus in human, duck and chicken are shown and lines indicate orthology relationship. In duck, overlapping scaffolds were merged into single synteny blocks. The scaffolds from top to bottom are: 1st block LMAN1-TXNL1: KB743487.1, KB743741.1 and KB746388.1, 2nd block TCF4-ACAA2: KB742564.1 and KB743255.1, 3rd block LIPG-DYM: KB743549.1, 4th block SMAD7-CTIF: KB744096.1, 5th block ZBTB7C-SKOR2: KB752069.1, 6th block IER3IP1-PIAS2: KB743344.1. The asterisk (*) indicates the position of the telomere on the Z chromosome of chicken. This comparison highlights the inversions and loss/rearrangement of a 17-genes segment (dark grey) in this locus between human and duck compared with chicken.

**Figure 2:**
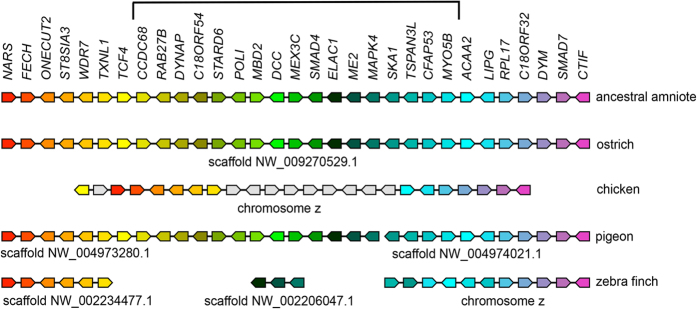
Conservation of synteny at the *DCC* locus between the amniote ancestor, ostrich, chicken, rock pigeon and zebra finch. The gene name is shown at the top of the figure, which corresponds to the ancestral amniote synteny of the reference 31-genes segment. Orthologos genes are represented by color-coded block arrows (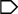) as indicated on the top row and the direction of the point of the block indicates the orientation of the gene. Genes represented in light grey (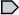) represent genes outside of the reference 31-genes segment which have been brought to the locus by inversions. Genes linked on the same scaffold/chromosomes are joined by a line whereas genes within the reference 31-genes segment not on the same scaffold are not joined by a line. The chromosome or scaffold accession number is indicated (ostrich: scaffold NW_009270529.1, chicken: chromosome Z, pigeon: scaffold NW_004973280.1 and scaffold NW_004974021.1 zebra finch scaffold NW_002234477.1, scaffoldNW_002206047.1, chromosome Z). The bracket at the top indicates the 17-gene region disrupted in Galliformes and Passeriformes.

**Figure 3:**
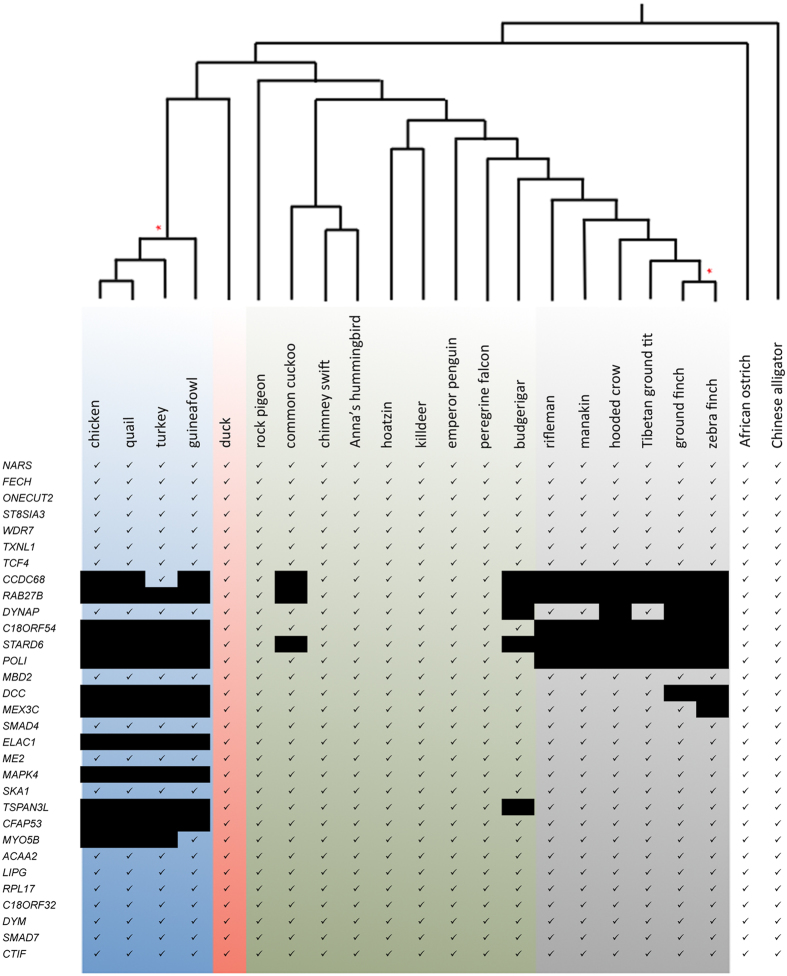
*In silico* analysis of genes within the original *DCC* locus indicates lost and salvaged genes in different bird species. Genes are shown in the same order as they are on the *DCC* locus in the ostrich genome. The syntenic order for each species may be different due to rearrangements or unknown due to too short scaffolds. The phylogenetic tree and species name are indicated at the top of the figure and gene name at the side. Based on *in silico* analysis, the presence or absence of a gene is indicated with a tick (√) or a black box (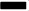) respectively. Galliformes (blue), Anseriformes (pink), Neoaves are in green apart from Passeriformes which are shown in grey. Ostrich and alligator are also shown. Red asterisks (*) in the phylogenetic tree indicates the evolutionary points at which the *DCC* gene was lost in a subset of bird species.

We next aimed to further characterize the *DCC* genomic region by a more in depth analysis. Since synteny is often conserved between different vertebrate species we examined the conservation of gene order at the *DCC* locus between mammalian and avian species (Fig. 1). We found that at least 50 genes surrounding the human and duck *DCC* loci were arranged in a similar order (Fig. 1). This is in contrast to the chicken where the comparable genomic region had undergone inversion thereby bringing a 17-gene-wide segment close to the telomere and an apparent loss of genes within this region (Fig. 1). In the genome of the Passeriforme zebra finch, where scaffold length allows for a degree of synteny analysis, genes in the block upstream of the disrupted region (*NARS* to *TXNL1*) were in a single scaffold and corresponded to the synteny in basal birds (Fig. 2). The genes in the block from *SKA1* to *CTIF* were also in a single scaffold and followed the same syntenic order as basal birds apart from the genomic region between *SKA1* and *LIPG* which had undergone inversions (Fig. 2). Thus as in Galliformes, in the Passeriforme, zebra finch, the 17-gene region from *CCD68* to *MYO5B* was disrupted. Finally, using either genomic or transcriptomic data sets we observed that part of this 17-gene region was also disrupted in the Cuculiforme common cuckoo and the Psittaciforme budgerigar, although in these cases *DCC* was detected (Supplementary Data Set 1, Table S1, Fig. 3). Overall, these data suggested that the *DCC* containing locus was susceptible to disruption in this region and initial high stringency analysis of genomic assemblies raised the possibility that *DCC* may be lost in subsets of Galliformes and Passeriformes.

### Several genes from the original *DCC* locus were salvaged in Galliformes and Passeriformes

While this genomic assembly analysis suggested that *DCC* and other genes from the disrupted locus were lost in Galliformes and Passeriformes it is nevertheless technically possible that these genes are present but not represented in genomic assemblies used in this study or evolved to a form that were not identified with the stringency criteria used. In order to examine this further, we performed additional extensive database searches that included lowering the stringency on our original searches and mining raw unassembled genomic reads and analysis of RNAseq data.

At the time of analysis, 10 of the 17-genes in the disrupted locus, including *DCC*, could not be detected in chicken, quail, turkey or guineafowl (Galliformes) genomic or transcriptomic databases examined (Fig. 3, Table 1 and Supplementary Data Set 1, Table S1). While no genomic assembly or transcriptomic data are currently available for the basal Galliforme brush turkey, strikingly, using available genomic raw reads, we detected a *DCC* gene in brush turkey similar to the canonical duck/mouse *DCC* (Table 1). This suggested that the disruption of the *DCC* locus happened in a sub-branch of Galliformes including chick, quail, turkey and guineafowl. The two genes at the end of the 17-gene locus, *CCD68* and *MYO5B*, were detected in turkey and guinea fowl respectively but not other Galliformes such as chicken or quail (Fig. 3 and Supplementary Data Set 1, Table S1). In contrast, 5 of the remaining genes from the original locus (*DYNAP, MBD2, ME2, SMAD4* and *SKA1*) were detected in the transcriptomic data for all Galliformes species examined and in the genomic assemblies from chicken (*Gallus gallus* 5.0) and turkey on scaffolds containing only one or two genes (Fig. 3 and Supplementary Data Set 1, Table S1). Since these were short scaffolds it was not possible to determine if these genes were within the same locus. This suggested that these particular genes had been salvaged during the disruption of the 17-gene locus during evolution of Galliformes.

**Figure 4:**
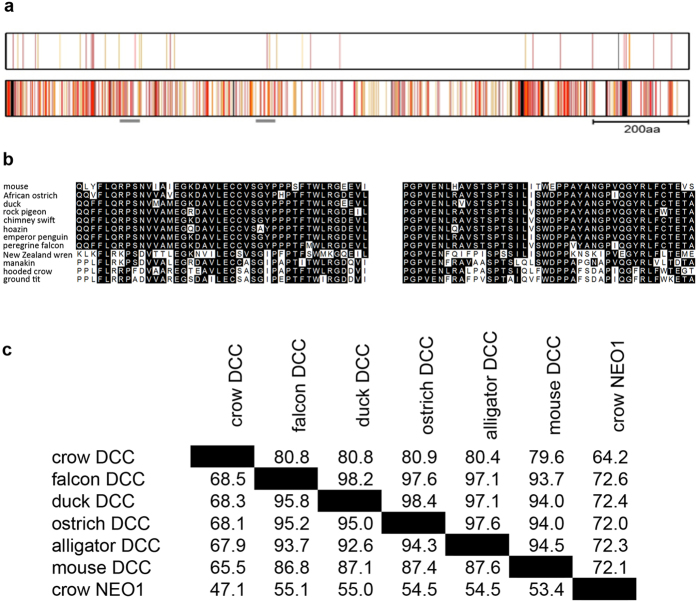
Hooded crow *DCC* has relatively low sequence similarity to orthologs from other bird species. **(a)** The amino acid sequence of DCC is represented by a rectangle from the N- to C terminal (left to right respectively). This is a visual representation to compare amino acid mutations between the inferred ancestral bird DCC sequence and either the peregrine falcon (upper rectangle) or the Passeriforme hooded crow (lower rectangle). Individual amino acids are represented by vertical lines along the length of the rectangle with the following colours indicating degrees of change in sequence; white - no change, orange (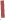) - conservative mutations and red (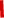) non-conservative mutations. Insertions/deletions are represented in black (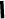). The scale bar represents 200 amino acids. **(b)** The amino acid sequences of regions highlighted by the gray line (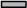) below the schematic in **(a)** are shown in comparison with a range of different bird and human DCC sequences in these regions. Alignments were performed using MAFFT. **(c)** Amino acid comparison between DCC sequences from different birds, and outgroups. Above the diagonal is the percentage similarity and below the diagonal percentage identity in Passeriformes birds compared with other birds and animals.

**Figure 5:**
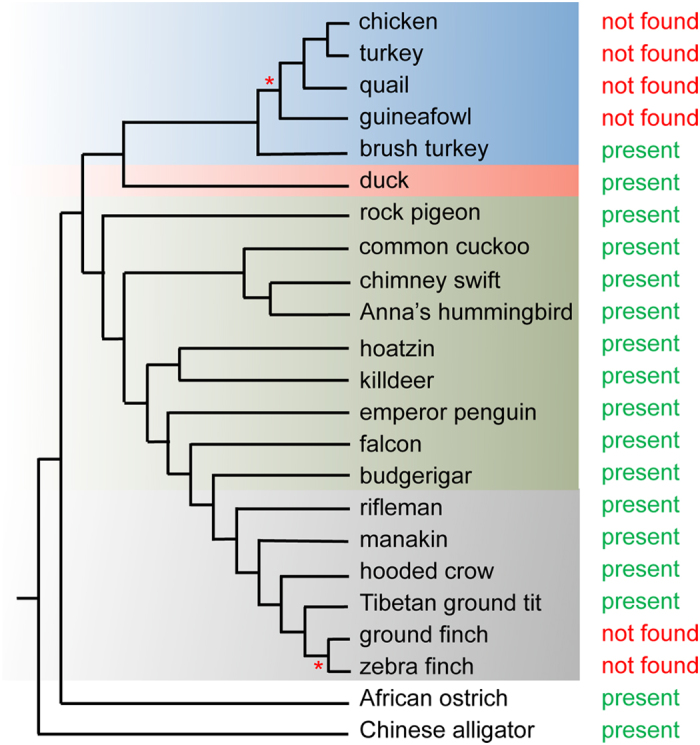
*DCC* was detected in most bird species examined. The status of *DCC* was mapped onto the currently accepted phylogeny of birds^41^,^78^. The *DCC* status is shown indicating presence or absence in bird and crocodilian species. The following are indicated in colour: Galliformes (blue), Anseriformes (pink), Neoaves are in green apart from Passeriformes which are shown in grey. The red asterisk (*) indicates the points of putative *DCC* loss during bird evolution.

Of the Passeriformes, six genomes; rifleman, golden-collared manakin, hooded crow, Tibetan ground tit, ground finch and zebra finch were examined^2^,^39^,^43^. By analysis of both genomic and RNAseq data we observed that *CCD86, RAB27B, C18OF54, STARD6* and *POLI* were not detected in any Passeriforme examined (Supplementary Data Set 1, Table S1, Fig. 3). *DYNAP* and *MEX3C* were salvaged from this deleted region in subsets but not all of the Passeriformes examined (Figs 2 and 3 and Supplementary Data Set 1, Table S1). Moreover, other genes from this region including *MBD2* and *SMAD4* – *MYO5B* were detected in all Passeriformes examined (Figs 2 and 3 and Supplementary Data Set 1, Table S1). Importantly, similar to the chick, quail, turkey, guineafowl branch of Galliformes we did not detect a *DCC* gene in ground finch or zebra finch by this analysis (Figs 2 and 3 and Table 1, Supplementary Data Set 1, Table S1). Unlike in zebra finch and ground finch where *DCC* could not be detected in assembled/unassembled genomic or transcriptomic data sets, in the genome assemblies of more basal Passeriformes including manakin, crow and Tibetan ground tit, we detected short fragments that were similar to the falcon *DCC.* To determine if these short hits were artefacts or a genuine *DCC* ortholog we next used these short sequences as bait to find other fragments of a potential *DCC* orthologs from these species within available transcriptomics datasets. By this approach we assembled a full-length sequence for hooded crow *DCC* and detected fragments that together covered the full length of *DCC* orthologs in the golden-collared manakin and Tibetan ground tit (Fig. 4a). Strikingly, these Passeriformes *DCC* orthologs had a much lower percentage identity to other vertebrate *DCC* genes compared with that normally observed amongst *DCC* genes, which are typically highly conserved (Fig. 4). In contrast to our previous analysis using falcon *DCC*, using this lower similarity crow *DCC* as bait in BLAST searches of genomic assemblies we detected *DCC* as the top hit in the more basal Passeriforme rifleman by this method and a full-length gene model could be reconstructed from raw genomic short reads (Table 1, Fig. 5). Overall, by thorough analysis of genomic or RNAseq data we detected *DCC* in many bird species including an ‘atypical’ diverged *DCC* in the Passeriformes rifleman, manakin, crow and ground tit. We did not detect *DCC* in chicken, quail, turkey, guineafowl (Galleformes) or ground finch and zebra finch (subset of Passeriformes), whereas other genes within the disrupted locus were salvaged (Figs 3 and 5, Table S1, Supplementary Data Set 1).

### DCC was not detected in chicken or zebra finch brachial spinal cord sections using well-verified DCC antibodies

This *in silico* analysis suggested that *DCC* may be lost in subsets of Galliformes and Passeriformes. Since this type of *in silico* analysis is contingent on the accuracy and completeness of genomic assemblies and mRNA databases we next aimed to robustly verify the *DCC* presence or absence by alternative experimental approaches.

Since DCC orthologs are typically highly similar to each other we reasoned that if a *DCC* ortholog was present in Galliformes and Passeriformes but not represented in the databases, a probe directed against duck *DCC* mRNA or well-characterized and robust antibodies that recognize DCC in other species would detect DCC in birds. For this experiment we focused on chicken (Galliforme) and zebra finch (Passeriforme) which are experimentally important model systems in developmental biology and neuroscience respectively and where embryonic material is more readily available. Since DCC expression and function is well characterized in the spinal cord, we compared the expression of DCC in spinal cord sections of mouse, chicken, zebra finch and duck embryos.

**Figure 6:**
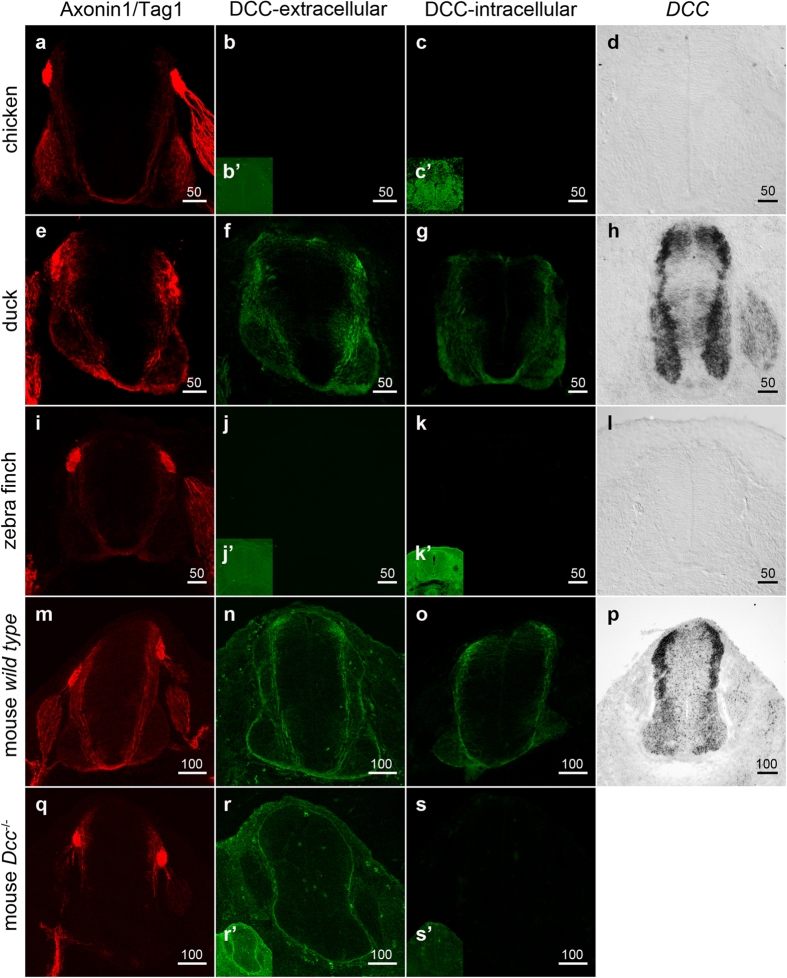
*DCC* protein is expressed similarly between duck and mouse but is not detected in chicken or zebra finch embryos. (**a**–**s**) Photomicrographs of transverse sections of the embryonic brachial spinal cord is shown in E5.5 chicken (**a**–**d**), E5.5 duck (**e**–**h**), E5.0–5.5 zebra finch (**i**–**l**), E11.5 wild type/transgenic mouse (**m**–**p**) and E11.5 *Dcc*^*−/−*^ mouse embryos (**q**–**s**) immunohistochemically labelled with the commissural axonal markers Axonin-1/Tag-1 (red) (**a**,**e**,**i**,**m**,**q**), DCC-extracellular (green) (**b**,**f**,**j**,**n**,**r**), DCC-intracellular (green) (**c**,**g**,**k**,**o**,**s**) or labelled by *in situ* hybridization with duck *DCC* (**d**,**h**,**l**) or mouse *Dcc* (**p**). The inset in **b’,c’,j’,k’,r’** and **s’** were imaged from the same samples as in (**b**,**c**,**j**,**k**,**r** and **s**) respectively at greater confocal gain and adjusted for brightness, contrast and gamma in Adobe Photoshop CS4 to examine if a low level of DCC immuno labelling could be detected in the samples. It could not. At least 3 embryos from each species were analysed. Representative images are shown. Scale bars are 50 μm or 100 μm as indicated in the Figure.

To test if *DCC* could be detected in chicken and zebra finch embryos we used *in situ* hybridization with a probe directed against duck *DCC. DCC* mRNA was detected in duck but not in chicken or zebra finch samples (Fig. 6). Next we used immunohistochemistry with a well verified monoclonal DCC antibody commonly used in the field raised against the extracellular domain of the human DCC, referred to here as ‘DCC-extracellular’. This antibody labelled spinal cord section of wild type but not *Dcc*^*−/−*^ knockout mouse embryos, confirming the integrity of the antibody under the conditions used (Fig. 6). The antibody labelled mouse and duck samples and we observed that the distribution of DCC protein and mRNA in mouse and duck spinal cord was similar, being expressed in commissural axons and cell bodies (Fig. 6). However, using this antibody, no signal was detected in either chicken or zebra finch embryos, consistent with the notion that in chicken and zebra finch, *DCC* was lost (Fig. 6).

While the monoclonal antibody used is well verified and commonly used in the study of DCC in different contexts, it is still possible that the epitope at a putative chicken or zebra finch DCC could have evolved so that it is not recognised by this antibody. Therefore we next identified and verified a commercially available polyclonal antibody directed against a 98 amino acid intracellular human DCC peptide region, referred to here as ‘DCC-intracellular’ (Fig. 6). This antibody labelled wild-type mouse spinal cord samples but not *Dcc*^*−/−*^ mouse embryos, confirming the integrity of this antibody (Fig. 6). Using the DCC-intracellular antibody we detected strong consistent labelling in both mouse and duck samples whereas we did not detect labelling in zebra finch embryos in any samples examined (Fig. 6). In chicken embryos we did not detect labelling in any samples at brachial level (Fig. 6). We did however, detect an inconsistent and very weak signal in some embryonic sections analysed at caudal levels (Supplementary File S1, Fig. S2). While very weak and inconsistent, this labelling was in the floor plate region in a pattern consistent with it being in commissural axons (Supplementary File S1, Fig. S2). Of note, the highest BLASTP hit for the 98 amino acid antigen for this antibody was chick Neogenin, raising the possibility that this weak labelling could be Neogenin. Strikingly, despite the apparent absence of DCC in chicken, and zebra finch, using commissural axonal labels, Tag-1 and Axonin-1 we observed that the commissural axonal trajectory of chicken, duck, zebra finch or mouse embryos at equivalent stages were comparable (Fig. 6a,e,i,m).

Taken together these data show that DCC was not detected in chicken or zebra finch by well-verified antibodies or a mRNA probe against the duck *DCC.* These data support the *in silico* data that DCC was not detected in chicken or zebra finch. Moreover, we observed that the expression levels and distribution of DCC were similar in mouse, duck and that the pattern of spinal commissural axons development, were similar in mouse, duck and chicken regardless of whether DCC was detected or not.

### Duck and chicken *Neogenin* are expressed in a comparable manner but are distinct from duck *DCC*

**Figure 7:**
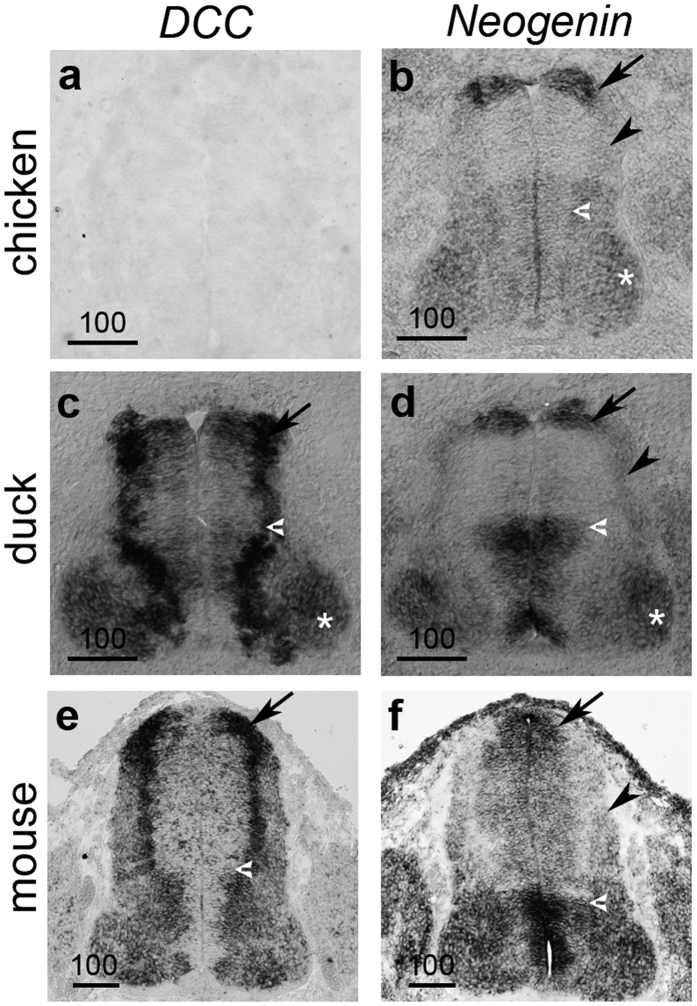
Comparative expression of *DCC* and *Neogenin* mRNA in chicken, duck and mouse embryos. **(a**–**f)** Photomicrographs of transverse sections of the embryonic mid-gestation brachial spinal cord are shown for chicken (**a**,**c**), duck (**c**,**d**) and mouse (**e**,**f**) after *in situ* hybridization using riboprobes against *DCC* and *Neogenin* in respective species (for chicken, the duck *DCC* probe was used). Consecutive sections on different slides were imaged apart from the chicken sample where a different embryo of equivalent stage and axial level were used. The number of embryos analyzed for each species was n = 3 for chicken, duck and mouse, respectively. Representative images are shown. Black arrows point to the dorsal spinal cord where dI1 neurons are derived from. Black arrowhead points to dorsal neurons expressing *Neogenin.* The white arrowhead points to the border between the ventricular and mantel zone. Scale bars are 100 μm.

Since our data suggested that a branch of Galliformes had only a *Neogenin* gene whereas the closely related duck had both *DCC* and *Neogenin* paralogous genes we next aimed to determine if the expression pattern of *Neogenin* had co-evolved together with the loss/molecular evolution of *DCC* in chicken. This was accomplished by comparing the expression patterns of chicken, duck and mouse *Neogenin* in parallel with that of duck and mouse *DCC*. We observed that duck embryos had a distinctive expression of *DCC* and *Neogenin* with consecutive sections whereas *DCC* expression in duck was comparable to *Dcc* in mouse (Fig. 7). Moreover, at the equivalent development stage in chicken we observed that *Neogenin* had an expression pattern more similar to duck *Neogenin* than duck or mouse *DCC* (Fig. 7). Overall, we observed that the paralogues *DCC* and *Neogenin* have overlapping but distinct expression patterns in the duck and that chicken *Neogenin* is more similar in expression to duck *Neogenin* than duck *DCC.* Taken together this suggested that the expression of *Neogenin* had not fully co-evolved to subsume the expression domains of *DCC* in chicken.

### gPCR analysis of putative lost and salvaged genes in Galiformes and Passeriformes

**Figure 8:**
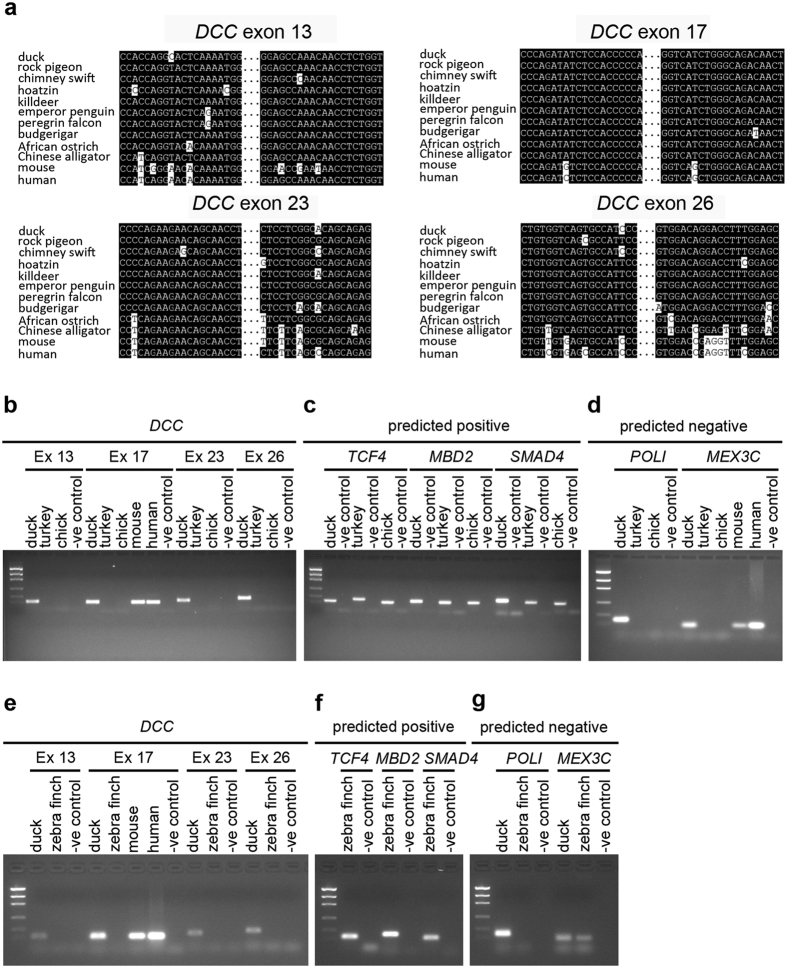
gPCR analysis of lost and salvaged genes. **(a**) Alignments of the *DCC* gene in the regions of primer design: *exons 13, 17, 23* and *26.* **(b**–**d)** gPCR of duck, turkey, chicken, mouse and human samples. **(b**) gPCR for duck *DCC exons 13, 17, 23* and *26* primer pairs indicated in **(a)**. **(c)** gPCR of genes in Galliformes (*TCF4, MBD2* and *SMAD4*) putatively salvaged from the original *DCC* containing locus. **(d)** gPCR of other genes missing in the Passeriforme zebra finch (*POLI* and *MEX3C*). **(e**–**g)** gPCR of zebra finch, duck, mouse and human samples. **(e)** gPCR for duck *DCC exons 13, 17, 23* and *26* primer pairs indicated in **(a)**. **(f)** gPCR of genes (*TCF4, MBD2* and *SMAD4*) putatively salvaged from the original *DCC* containing locus of Passeriformes. **(g)** gPCR of other genes predicted to be missing in Galliformes (*POLI* and *MEX3C*). Replicates from at least 3 independent individuals/samples (7 for zebra finch) were analysed and repeated at least 3 times. One representative sample from each species is shown. The negative control indicated for each primer pair is a template free reaction.

Our genomic, transcriptomic and immunohistochemical labelling supported the loss of chicken and zebra finch *DCC* whereas commissural axons appear to form normally regardless. This, taken together with the finding that in basal Passeriformes an uncharacteristically low similarity ‘atypical’ *DCC* ortholog was detected lead us to consider that *DCC* may be present in chicken and zebra finch but derived to a form that is undetectable by the above methods. Therefore, in a final attempt to identify a chick or zebra finch *DCC* gene we used a genomic PCR (gPCR) approach. We first used gPCR to determine if the absence or presence of genes in this 17-gene locus by *in silico* analysis in different birds could be experimentally verified or not. We generated gDNA from cells from at least 3 independent samples for each species examined (human, mouse, chicken, turkey, duck, zebra finch). We designed oligonucleotides to amplify different genes either from the known sequence or from the duck sequences, within regions of high sequence conservation to other birds and human. Consistent with the *in silico* analysis was the observation that *TCF4, MBD2* and *SMAD4* were detected by genomic PCR (gPCR) in duck, turkey, chicken and zebra finch samples thereby confirming that these genes are indeed present in the genomes of these animals despite the disruption in the locus (Fig. 8). Similarly, PCR fragments of other genes predicted to be absent (*POLI* and *MEX3C*) were not amplified in the Galliformes chicken and turkey gPCR using conserved primer sequences against these genes from duck (Fig. 8d). In zebra finch, while, as predicted, no band was amplified for *POLI* a faint band of the right size was detected in 4/7 samples for *MEX3C* (Fig. 8g). This suggested that in contrast to its apparent absence in the high quality genomic and transcriptomic assemblies and raw read data currently available *MEX3C* may in fact be present in zebra finch. By analogy, this raised the possibility that the *DCC* gene was not lost in zebra finch and chick but rather not represented in the databases or derived to a form that is undetectable by *in silico* searches and antibodies. We therefore next designed primer pairs in conserved regions of *DCC* exons to examine if *DCC* could be detected by this method. A product of the predicted size was amplified in the positive control duck samples for all primer pairs in all *DCC* exons examined (Fig. 8). However, we did not find any evidence for chicken, turkey or zebra finch *DCC* by genomic PCR using primers directed towards highly conserved exons 13, 23 or 26 of the duck *DCC* gene (Fig. 8). Of note, for the duck *exon 17* primer pair, a band was amplified in mouse and human gDNA samples suggesting that primer design and stringency was appropriate to amplify chicken, turkey or zebra finch *DCC* if it had been present in the samples (Fig. 8). Importantly, using this primer pair, we did not detect a chicken, turkey or zebra finch *DCC* sequence (Fig. 8). Taken together, our *in silico* and experimental data suggests that a *DCC* ortholog was either indeed absent from the chicken, turkey or zebra finch genomes or has such a highly derived sequence that it could not be detected by the stringent methods employed in this study.

### Salvaged genes in the disrupted locus of Galliformes and Passeriformes evolved faster

**Figure 9:**
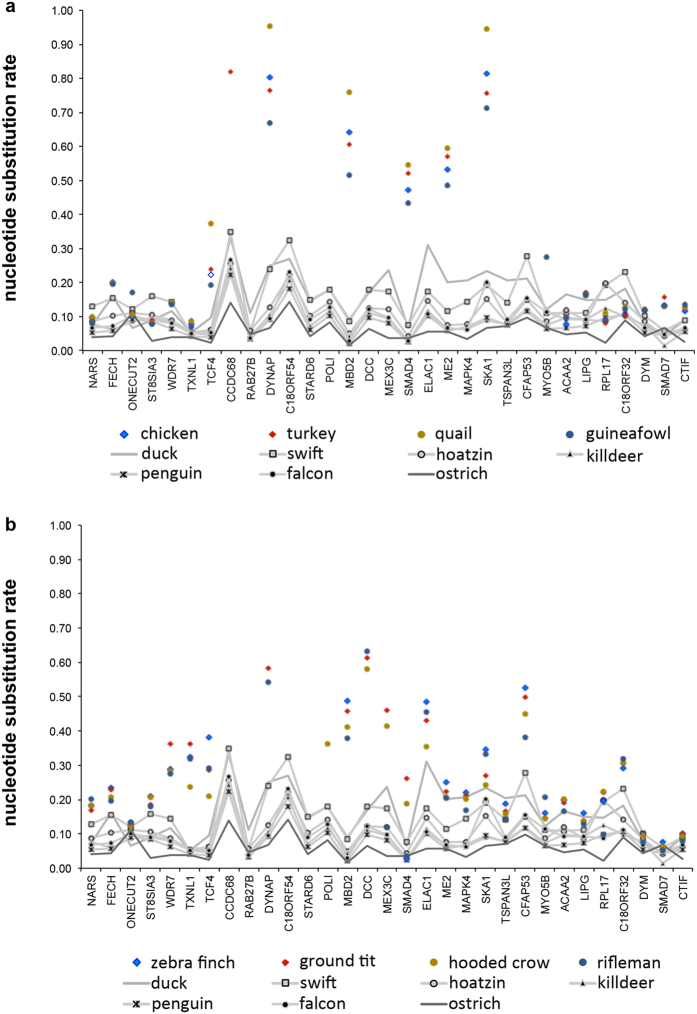
Genes salvaged from the original *DCC* locus are evolving at a faster rate than orthologs that remained at the original locus. The graphs show the gene name on the x-axis with the original ostrich synteny preserved. On the y-axis is the rate of nucleotide substitution measured as the total branch length between the alligator/bird common ancestor and each particular species. **(a)** Galliformes, which have lost *DCC*, are indicated in colour; turkey (red 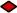), chicken (blue 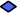), quail (mustard 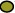) and guinea fowl (blue circle 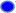). Passeriformes are not shown on this graph. Other birds that retained the genes from the locus are shown in grey. The value for each gene/species is shown and is therefore a single data point meaning a standard error is not presented in the graph. **(b)** Passeriformes are indicated in colour; zebra finch (blue 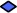), ground tit (red 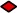), hooded crow (mustard 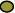) and rifleman (blue circle 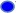). Galliformes are not shown on this graph. Other birds are shown in grey. The lines between the data points do not represent an analysis of evolution rates between one data point and the next and are included as a visual anchor to follow the data points from individual species. The full dataset, including other Galliformes and Passeriformes and corresponding statistical analysis is shown in Tables S1–S5 (Supplementary Data Set 1). The curated predicted coding sequences used are shown in Supplementary Data Set 2.

Since our previous data had identified a *DCC* gene in the Passeriforme crow, which was uncharacteristically diverged from other vertebrate *DCC* genes, we aimed to assess whether the disruption of the 17-gene locus in birds may have resulted in or coincided the salvaged genes from the region evolving at a faster rate. To test this idea, we next examined if the salvaged genes had undergone a changed rate of evolution compared with genes in this region where the locus was not disrupted. To approach this we generated a list of predicted sequences from genes of this region for all species examined and calculated the nucleotide substitution rate of these sequences (Figs 3 and 4, Supplementary File S1, Supplementary Data Set 1, Tables S1–S5). We observed that the salvaged genes, *DYNAP, MBD2, ME2, SMAD4* and *SKA1*, in chicken, quail, turkey and guineafowl (Galliformes) had undergone a significantly higher rate of nucleotide substitution compared with orthologous genes in other bird species or genes that had been adjacent in the original locus (Fig. 9 and Supplementary Data Set 1, Tables S1, S2 and S5, Supplementary Data Set 2). In Passeriformes we also observed that the salvaged genes had a significantly higher nucleotide sequence substitution compared with orthologous genes in other bird species (Supplementary Data Set 1, Table S1). Of note we observed a generally higher rate nucleotide sequence substitution in Passeriformes compared with other species examined (Fig. 9, Supplementary Data Set 1, Tables S1, S3 and S5). Importantly, in the Passeriformes rifleman, manakin, crow and ground tit, the ‘atypical’ *DCC* gene was highly derived. This branch length data provides evidence that *DCC* in these species too had evolved at a significantly faster rate than the *DCC* genes in species where the locus stayed intact. Taken together, these data support the notion that genes salvaged from the disrupted locus had been relocated to genomic landscapes favouring higher evolutionary rates.

## Discussion

Here we demonstrated that the *DCC* containing locus was disrupted independently in different branches of birds. This resulted in species-specific genome change through apparent gene losses and by a faster evolution rate of genes salvaged from the disrupted locus. The *DCC* gene was not detected and therefore appeared to be lost independently in subsets of Galliformes and Passeriformes whereas *DCC* was present in other birds including duck. Moreover, spinal commissural axons appear to project in a similar way in mouse, duck, chicken and zebra finch embryos, despite the apparent loss of *DCC* in chicken and zebra finch.

Avian genomes are known to be compact, have a relatively high rate of chromosomal rearrangement and are missing a number of genes^1^,^39^,^44^,^45^. This is confounded by the knowledge that in chicken and other birds genomic information is encoded on microchromosomes which are less well represented in genomic assemblies compared with the larger chromosomes^46^. This raises the possibility that the ‘missing’ *DCC* locus could have been translocated to a region poorly represented in the genomic assemblies. However, the finding that *DCC* loss happened independently twice during bird evolution supports the view that *DCC* was truly absent and not simply a technical artefact of genome assembly or the loss of a gene as an oddity in an inbred or farmed strains. Nevertheless, it is still formally possible that *DCC* was missing from the databases for technical reasons. Arguing against this idea, we did not detect *DCC* in the Galliformes, chicken, quail, turkey or guniea fowl or the Passeriformes zebra finch or ground finch after exhaustive alternative *in silico* and experimental strategies. However, of note, using one of the well-verified antibodies raised against DCC very faint, occasional immunolabelling was observed in caudal chicken embryo spinal cord sections (Supplementary File S1, Fig. S2). The Neogenin gene shares extensive sequence similarity to DCC in most vertebrates and BLAST analysis of the peptide used to make the antibody resulted in hits for chicken Neogenin. This suggested that the antibody may have weak cross reactivity to chicken Neogenin. However, we did not detect this weak labelling in the mouse *Dcc*^*−/−*^ embryos arguing that this weak labelling was either specific for chicken Neogenin or that it was recognising a very derived chicken DCC. Based on all our current evidence, we consider that this weak antibody labelling most likely represents a signal from binding to chicken Neogenin.

As with other vertebrate species, spinal commissural neurons in chicken embryos develop in a similar way to mouse and other vertebrates and are attracted to the floor plate by the DCC ligand Netrin-1^37^,^47^,^48^. Since the spinal commissural neurons in chicken and zebra finch embryos were found to be similar to duck and mouse the putative loss of *DCC* in these birds would suggest DCC is dispensable for commissural neuron formation. This is in sharp contrast to findings in other animal models and humans where disruption of *DCC* has been associated with significant phenotypic changes, disorder and disease states^10^,^11^,^12^,^13^,^14^,^15^,^16^,^17^,^18^,^19^,^21^,^22^,^23^,^25^,^30^,^36^. Given this, it is tempting to speculate that the independent losses of *DCC* in different birds were permitted by an evolutionary release on the constraints on *DCC* sequence/function before it was lost. In that case, the loss could have remained as a result of genetic drift rather than adaptive change. This raises the interesting question of how genes that are otherwise highly conserved become dispensable and what ‘internal’ molecular adaptations have occurred, such as the use of an alternative pathways or redundant receptor. In this respect, known DCC ligands such as the Netrin family members and Draxin are present and expressed in the chicken^49^,^50^. Therefore, to the best of our knowledge, there is currently no evidence to suggest there has been an associated loss of ligands specifically in Galliformes and Passeriformes which lack *DCC* versus other bird species. While *DCC* was found to be absent in subsets of birds, the *DCC* paralog *Neogenin* was detected in all bird species examined. It has been previously suggested that Neogenin could functionally substitute for loss of *DCC* in chicken as a result of adopting a more ‘DCC like’ expression pattern^37^. However, in contrast, the data presented here suggest that the expression of *Neogenin* in chicken and duck embryos are similar to each other but distinct from duck and mouse *DCC* (Fig. 7). A recent study in mouse demonstrated that in the spinal cord, *DCC* and *Neogenin* are partly overlapping in expression and that *Dcc*^*−/−*^*: Neogenin*^*−/−*^ double mutant embryos have a stronger commissural axon phenotype than either *Dcc*^*−/−*^ or *Neogenin*^*−/−*^ single mutant embryos^51^. This suggests that, in mouse, Neogenin has a role in commissural axon guidance in addition to DCC. This may account for the previous observations in chick by Phan *et al*. that Neogenin has a role in commissural axon guidance^37^,^51^. However, the relatively low penetrance of the commissural axon phenotype in Phan *et al*., taken together with our observations that Neogenin expression was similar between duck and chicken despite the respective presence or absence of *DCC* suggests that the molecular adaptation/evolution of the pathway is not entirely accounted for by a change in Neogenin expression^37^,^51^. Overall, the evidence in the present study strongly suggests that in chicken, zebra finch and other birds *DCC* was lost.

That loss of several neighbouring genes at the *DCC* locus happened independently twice during bird evolution, in both subsets of Galliformes and Passeriformes, also suggests that the *DCC* containing locus could be in general a region of relatively higher genomic instability. Consistent with this general idea, in the common cuckoo (Cuculiformes) and the budgerigar (Psittaciformes) we also observed a deletion in this region, although in these cases, the *DCC* gene was retained. In chicken this region was rearranged bringing the remaining locus close to the telomere of the Z- chromosome. Both telomere regions and Z- chromosomes are regions known to influence genomic instability^52^,^53^. Moreover, the finding that the salvaged genes from the locus were evolving at a faster rate suggested that they are within a genomic landscape, which favours faster rates of evolution. Fundamental to this concept was the observation that in the Passeriformes, in which we found a *DCC* gene (hooded crow, rifleman and manakin) the *DCC* gene was ‘atypical’ being significantly derived from the canonical *DCC* sequences. Importantly, a salvaged/derived *DCC* was not detected in the Galliformes chicken, quail, turkey or guinea fowl or in the Passeriformes ground finch or zebra finch. While we do not detect DCC in these animals after exhaustive and diverse analysis, we do not formally exclude the possibility of a DCC-like molecule evolutionarily derived from the original bird *DCC* still assuming DCC function in these animals. Based on the nucleotide and amino acid mining and antibody binding evidence presented here, we predict that if a remnant of the *DCC* gene existed it would be highly derived at the sequence level regardless whether functional or not. In other areas of biology, examples of protein groups that can have a well-conserved function and tertiary structure but low nucleotide and amino acid sequence conservation are well documented. We conclude in this study that a canonical *DCC* ortholog is not present in chicken and zebra finch amongst others based on nucleotide, amino acid sequence and antibody binding and future work will focus on examining if a gene with a highly derived sequence or a gene of different origin subsumes the function of *DCC* as a result.

Whether a *DCC* ortholog is completely lost in subsets of Galliformes and Passeriformes, functionally replaced by *Neogenin* or derived to a form that is functionally conserved but unrecognizable by sequence implies that a signalling pathway fundamental to a wide range of biological processes is different in these vertebrates. In particular, chicken is commonly used as a direct complementary model system to mouse and other species that have a *DCC* gene especially in the field of neural guidance where Netrin/DCC signalling has a broad role^10^,^11^,^12^,^13^,^14^,^15^,^16^,^17^,^18^,^19^. It therefore could be beneficial in the field to explore duck as a model system for such analysis. Overall the general concept of loss/evolution rate of key genes is an important consideration for experimental biologists focusing on molecular genetic mechanisms of diverse biological processes as to their choice of model system. Moreover, the phenomenon of loss of an otherwise highly conserved gene while the characteristics it regulates remain unchanged is of conceptual importance for understanding molecular evolution of key biological processes and how such losses may provide the basis for latent genetic and phenotypic diversity and therefore biodiversity within the population.

## Methods

### Animals, embryos and muscle tissue

Wild type SV/EV129, *Math1nGFP* transgenic and *Dcc*^*+/−*^ mice were used^11^,^54^,^55^. Wild type SV/EV129 mice were obtained from Taconic, Denmark and bred in house at the Umeå University animal facility. Fertilized chicken and duck eggs were obtained from Stellan Hennström, Vännäs, Sweden and Agrisera AB, Vännäs, Sweden, respectively. Mouse embryos were generated and staged as previously described^30^. Duck, chicken, zebra finch and mouse embryos were harvested approximately 5.0–5.5 days of incubation (bird) or 11.5 days of gestation (mouse), fixed and processes as described in detail previously^56^. Muscle tissue from duck, chicken and turkey was obtained from local food retailers (ICA gourmet and Duå, Umeå) from 3 independent animals for each species. Mouse experiments were performed in accordance with national laws, local guideline and were approved by the Committee for Animal Experimentation in Northern Sweden (A65–14). Use of fertilized chicken, zebra finch and duck eggs/embryos incubated to the stages examined here and use of meat from animals killed for food does not require an ethical permission/permit under Swedish law. The zebra finch embryos were generously provided by Lauren Guillette and Susan Healy, University of St Andrews, UK.

### Duck and chicken egg incubation

Duck and chicken eggs were incubated in a standard chicken egg incubator using the same method as incubating chicken eggs (∼38 °C in a humidified incubator). Eggs were harvested at approximately 5.5 days after the start of incubation, fixed and processed as described above.

### Genomic PCR

gDNA was generated from at least three independent samples or individuals for each species; mouse (n = 3), duck (n = 3), chicken (n = 3), turkey (n = 3), zebra finch (n = 7) and humans (n = 3). gPCR was performed using the primers and procedures detailed below. Full-length mRNA sequence alignments of *TCF4, POLI, MBD2, DCC, MEX3C* and *SMAD4* were performed using MAFFT (alignment algorithm) with the default settings. Primer pairs for each gene were designed within single exons. In addition, primer pairs for *DCC, POLI* and *MEX3C* were designed in regions of high conservation between species, within single exons and ensuring that the 3′ nucleotide of the oligonucleotides was not at a codon-variable position (Table S6, Supplementary File S1). Mouse tail biopsies, pelleted cells derived from two independent human cell lines (HEK-293-T and chIPSC4 (Cellartis #Y0026/iPS-CHIPSC4-VIAL)), embryo tissue from zebra finch and chick embryos and muscle tissue from duck, chicken and turkey were used to isolate genomic DNA (gDNA) using Direct Lysis PCR (tail) solution (Viagen #102-T) according to manufacturers instructions. In short, 200 μl of Direct Lysis PCR solution containing proteinase K (11 μg/ml) was added to the tissue/cells and incubated in a shaking heating block at 55 °C overnight. The digested samples were then denatured at 85 °C for 45 minutes, microfuged for 1 minute and subsequently stored at either 4 °C or −20 °C. gDNA from at least three individual animals of each species was isolated. The third human gDNA sample was isolated from human ES cells and was a gift from Dr. Iwan Jones, Umeå University, Sweden. PCR was performed at 62 °C for 35 cycles using GoTaq hot start green mastermix (Promega #M512B) with the primers detailed in Supplementary File S1, Table S6.

### Cloning of duck *Neogenin* and *DCC* cDNA

Tissue from a duck embryo was homogenized using a pestle and mortar in RTC Buffer and total RNA isolated using the RNeasy^®^ Minikit kit (Qiagen cat #74104) according to manufactures instructions. cDNA was synthesized using a SuperScript^®^III First-Strand Synthesis system for RT-PCR according to manufacturers instructions (Invitrogen #18080–051). Duck *DCC and Neogenin* cDNA were amplified by PCR using the wizard setting in a Unocycler PCR machine (VWR) with the primers indicated in Table S7, Supplementary File S1) and cloned into pGEMT (Promega). The cloned sequenced were verified by sequencing (Supplementary Data Set 3).

### *In situ* hybridization

*In situ* hybridization was performed as described previously using a digoxigenin-labelled riboprobes^56^,^57^. cDNA plasmids were used to generate riboprobes for mouse and duck *DCC* and mouse, duck and chick *Neogenin*. All plasmids were verified by sequencing (Supplementary Data Set 3).

### Analysis of synteny

Chromosome/scaffold gene order information was retrieved from the following genomes: human: assembly GRCh37.p13; Ensembl annotation release 83, duck: assembly BGI_duck_1.0; Ensembl annotation release 83, chicken: assembly Gallus_gallus-5.0; NCBI annotation release 103, ostrich: assembly ASM69896v1; NCBI annotation release 100, pigeon: assembly Cliv_1.0, NCBI annotation release 101, zebra finch: assembly Taeniopygia_guttata-3.2.4, Ensembl annotation release 67. 25 genes upstream and 25 genes downstream of *DCC* were analysed. Orthology was verified using the Ensembl orthology tables available on BioMart (Ensembl release 83)^58^.

### Identification of *DCC* and neighbouring genes in avian and crocodilian genomes

The analysis in Figs 1, 2 and 3 was performed using the approach below. Genes that were not identified by these methods were provisionally labelled as lost. Based on the human *DCC* locus, orthologs of human *DCC* and 30 neighbouring genes were searched for in a selection of 18 high-coverage avian genomes representing the main bird taxa including chicken, turkey, duck, rock pigeon, cuckoo, chimney swift, Anna’s hummingbird, hoatzin, killdeer, emperor penguin, peregrine falcon, budgerigar, golden-collared-manakin, hooded crow, Tibetan ground tit, medium ground finch, zebra finch and African ostrich and the Chinese alligator (*Alligator* sinensis)^2^,^40^,^42^,^43^,^59^,^60^,^61^,^62^,^63^,^64^,^65^,^66^,^67^. In addition, genomic sequences of 3 additional birds with lower coverage genomes - Japanese quail, *guineafowl* and rifleman were searched in the genomic RefSeq database at NCBI and in the quail genome (available at www.nodai-genome.org)^40^,^68^. Finally, genomic raw reads of the brush turkey available in the sequence read archive (SRA, accession number SRP066515) were searched. The Latin name and datasets searched for each species are listed in Table S8 together with references (Supplementary File S1). The predicted coding sequence of each gene in each species analysed was identified in a three-step strategy followed by verification of gene identity.

(i) First, many orthologs were identified by performing gene name search on Avianbase and NCBI^69^. (ii) If the ortholog was not found by this standard method, the genome assembly was searched using tblastn with relaxed settings, using the full-length duck protein as a query (E value < 1000, maximum matches in a query range = 5). (iii) Failure to identify appropriate orthologs using these two approaches could be due to a gene having evolved to a point that it could not be detected at the stringency settings defined above (i.e. very derived from the parent sequence), absence/evolutionary loss of a gene or incomplete and/or poorly compiled assemblies of the genomic region being examined. If a gene was present in the genome but not detected by the above methods, it may be deposited in transcriptomic databases. Next, transcriptomic data (raw RNAseq reads) available from the sequence read archive at NCBI was searched using tblastn with relaxed settings (E value < 1000). In species where no RNAseq data was available, raw genomic reads were searched^40^. Since we found the duck ortholog of each gene at the human locus we used the corresponding duck protein as a query bait for searches in Galliformes. Similarly, the falcon ortholog was used as a query for searches in Passeriformes. The datasets searched for each species are listed in Table S9, Supplementary File S1).

Finally, each ortholog was verified by the following methods: for each gene analysed, the full length predicted coding sequences in all the species examined (orthology group) were aligned. Since the sequences used are dependent on hypothetical predictions of transcribed units which represent only one possible splice variant, in some cases manual editing or curation of the gene model were required. Where required sequences were curated by two methods: (i) Where available, genomic sequence was retrieved from the genome assembly and GeneWise2 was used to predict the coding sequence^70^. (ii) RNAseq reads were retrieved from the sequence read archive and assembled using transcriptome-wide de novo assembly with Trinity (de novo assembly program) or alternatively reads were retrieved on a gene by gene basis using blastn and assembled with Trinity^71^. When no RNAseq data was available genomic raw reads were retrieved and assembled exon by exon. The RNAseq and genomic raw reads datasets used for curation of predicted coding sequences are listed in Table S7, Supplementary File S1. The curated predicted coding sequences are available in FASTA format in Supplementary Data Set 2.

Identity of these predicted coding sequences was verified by conservation of synteny if the genomic scaffold was long enough and/or by the percentage identity to the corresponding duck nucleotide sequence in the alignment (using a cut off of greater than 75% identity). Since some genes analysed in Passeriformes, Galliformes, cuckoo and budgerigar, evolved at a faster rate than their orthologs, in some cases orthology could not be concluded from high percentage similarity or conservation of synteny. In such cases, orthology was inferred from consistent annotation of the first 100 hits of a blastx search on NCBI’s nr database (genes with a branchlength >0.4 substitutions per sites). In the case of the DCC gene, orthology was verified by constructing a phylogenetic trees with Maximum Likelihood (ML) algorithm in MEGA7 software (Supplementary File, Fig. S1)^72^.

### Verification of *DCC* status in Galliformes and Passeriformes

No *DCC* gene was found using the above methods in databases for the Galliformes chicken, turkey, Japanese quail or one word nor the Passeriformes zebra finch and medium ground finch. Therefore, additional searches were performed as follows to find a putative *DCC* gene in these species. Raw RNAseq and/or genomic reads were searched directly in the sequence read archive (SRA) using tblastn with relaxed settings with the Neogenin/intracellular domain of duck DCC as a query (E value < 1000, number of hits kept >200000). An extensive number of available RNAseq datasets were searched by blocks of >1 billion reads in guineafowl and quail (see Table S9 in Supplementary File 1. In turkey, chicken and zebra finch, searched datasets included tissues where *DCC* is expected to be expressed including brain and gut as well as whole embryos. A complete list of the SRA project accession numbers of the datasets searched is available in Table S9, Supplementary File 1. The hits were assembled by *de novo* assembly using trinity and then similar and overlapping reads were merged using cd-hit-est with a threshold of 97% identity^73^. By this method, some of these reads resulted in longer assembled contigs whereas other remained as single reads. First, both the assembled and non-assembled reads found to be representing *Neogenin* using blastn were discarded (>40 nt and >95% identity alignment). To further validate the identity of potential *DCC* candidates, a blastx search was performed using the nr protein database on the remaining assembled contigs and single reads (relaxed settings E value > 1000). From that analysis, where any match to *DCC* was identified in the first 100 hits a more in depth analysis of the sequence was undertaken. In order to establish if these putative *DCC* hits corresponded to another sequence from the same species, the candidate sequences were mapped to transcriptome *de novo* assemblies or available genomic assemblies from the same species using blastn. In cases where a matching sequence was identified (>95% identity) it was subsequently mapped to the chicken or turkey genome using blastn in order to determine if it corresponded to a gene or intergenic region other than *DCC.* All putative DCC sequences found corresponded to genes and intergenic regions other than DCC and/or the fraction of the sequence aligning with DCC was shorter than 20 amino acids.

Finally DCC in chicken was searched for using the following additional method: De novo assemblies (derived from the raw reads from SRA project number PRJNA308865) were compressed using cd-hit to remove redundancy and translated in 6 frames with a requirement of an ORF >50aa long. Next HMM profiles of DCC protein domains (IgG, FN or the Neogenin domain) were compiled using DCC proteins from several vertebrate species (including duck, ostrich, peregrine falcon, Chinese alligator, human, xenopus, spotted gar, zebrafish and elephant shark) but excluding vertebrate Neogenin. The HMMER tool was then used to aim to find chicken DCC using these DCC profiles. We did not find any chicken DCC ortholog using these methods.

### Calculation of percentage identity and mapping of mutations

For the mapping of mutations in Fig. 4a, the avian ancestral DCC sequence was inferred in MEGA7 software using an alignment of full length DCC protein from ostrich, duck, chimney swift, killdeer, hoatzin and peregrine falcon. The resulting sequence was aligned with either peregrine falcon or hooded crow DCC amino acid sequence using MAFFT. Substitutions at each position were scored as conservative or non-conservative based on the blosum62 substitution matrix. For calculation of percent identity and percent similarity in Fig. 4, the full length amino acid sequences of crow, falcon, ostrich, alligator and mouse DCC as well as mouse NEO1 were aligned with MAFFT. Regions with insertions/deletions were trimmed manually in Bioedit and the percent identity was calculated by comparing the aligned sequences two by two. Percent similarity was calculated using the substitution matrix BLOSUM62.

### Quantification of evolution rates

For quantification of evolutionary rates in Fig. 9, nucleotide alignments were generated with MAFFT using the nucleotide sequences from the predicted coding sequences curated as described above and listed in Supplementary Data Set 2^74^. ML phylogenetic trees were constructed using MEGA7 including the alligator ortholog sequence as a root. The evolutionary rates were assessed using the total branch length from the common ancestor of alligator and birds to a particular species. The total branch length (also called sympatric distance to the root) was calculated using the phylogenetic trees derived from MEGA7. The raw data and statistical analysis are in Tables S1–S5, Supplentary Data Set 1, legends in Supplementary File S1 and the statistical analysis described below.

### Statistical analysis

The statistical analysis of the data in Table S1, the nucleotide substitution rate in different genes is shown in Tables S2–S5 (Supplementary File S1, Supplementary Data Set 1). In Table S1 all the raw data is shown and in in Tables S2–S5 the appropriate raw data extracted from Table S1 is shown (Supplementary Data Set 1). All statistical analyses were performed using Prism7 (GraphPad Software). Since two unlinked groups were being compared in each case, unpaired two-tailed Student’s t-tests were used to determine statistical significances. First, each data set were tested to determine if they were Gaussian using D’Agostino-Pearson omnibus normality test as indicated in the Tables S2–S5 (Supplementary Data Set 1). Where at least one of the two groups being compared did not pass this test, a non-parametric analysis was used followed by a Mann-Whitney test. If the data passed the normality test a non-paired parametric test assuming equal standard error was used. The raw data used to calculate the significance is in Table S1 (Supplementary Data Set 1). Some of this data was excluded from analysis for the following reasons: In common cuckoo and budgerigar the locus being analysed was disrupted but *DCC* was found (Table S1, Supplementary Data Set 1, Fig. 3, Supplementary File S1). Alligator was not included as it is not a bird. Where there were less than 3 data points the gene was excluded since it would not be possible to perform statistical analysis (for example only one *POLI* gene was detected in all Passeriformes examined (Table S1, Supplementary Data Set 1). The statistical analysis is shown in Tables S2–S5 (Supplementary Data Set 1) represent different comparisons. In Table S2, Supplementary Data Set 1, statistical analysis of: Group1 - nucleotide substitution rate of genes outside the disrupted locus (*NARS - TCF4* and *ACAA2 - CTIF*) versus group 2 - nucleotide substitution rate of 5 genes salvaged from the disrupted locus in Galliformes (*DYNAP, MBD2, SMAD4, ME2, SKA1*). In Table S3, Supplementary Data Set 1, statistical analysis of: Group 1 - nucleotide substitution rate of genes outside the disrupted locus (*NARS - TCF4* and *ACAA2 - CTIF*) versus group 2 - nucleotide substitution rate of genes within the disrupted locus in Passeriformes. In Table S4, Supplementary Data Set 1, statistical analysis of orthologous genes in the locus were analysed: Group 1: nucleotide substitution rate of orthologs from birds where the *DCC* locus was disrupted in Galliformes versus group 2: nucleotide substitution rate of orthologs from birds where the *DCC* locus was not disrupted. In Table S5, Supplementary Data Set 1, statistical analysis of orthologous genes in the locus were analysed: Group 2: nucleotide substitution rate of orthologs from birds where the *DCC* locus was not disrupted (same group as in Table S4) versus group 3: nucleotide substitution rate of orthologs from Passeriformes. The full table legends are in Supplementary File S1. The number of data points (n), means and standard error of the mean are shown for each group and the exact P-value as calculated by the program is shown for each group comparison for Tables S2–S5. Tables S1–S5 are provided as separate excel files (Supplementary Data Set 1).

### Immunohistochemistry

The following primary antibodies were used: mouse- αDCC-extracellular (1:1000) (Calbiochem #OP45), αDCC-extracellular (1:1000) (Oncogene #OP45), rabbit- αDCC-intracellular (1:1000) (Sigma# HPA069552), αAxonin-1 (1:2000)^75^, αTag-1 (1:10000)^76^. Immunohistochemistry was performed as described previously apart from the DCC-intracellular antibody (Sigma# HPA069552) where antigen retrieval was performed before the immunohistochemistry procedure^30^,^77^. In short, slides were washed 3× in PBS and submerged in sodium citrate buffer pH 6.0 (10 mM) in a domestic pressure cooker (Manttra microwave pressure cooker). The samples were then heated in a microwave (Electrolux, 900 w) at full power for four minutes followed by approximately 20 minutes to equilibrate to room temperature before performing the immunohistochemistry. Cy3, FITC and Alexa 488 secondary antibodies were obtained from Jackson Immunochemicals, Agrisera AB and Thermofisher.

### Microscopy and image analysis

Nikon Eclipse E800 brightfield/fluorescence and Zeiss LSM 710 confocal microscopes were used. Images were cropped in Adobe Photoshop CS4. The images in Fig. 6b’,c’,j’,k’,r’ and s’ were imaged from the same samples as in Fig. 6b,c,j,k,r and s respectively at greater exposure and adjusted for brightness, contrast and gamma in Adobe Photoshop CS4 to examine if a low level of DCC immuno labelling could be detected in the samples. It could not. All figures were assembled in Adobe Photoshop CS4 or Powerpoint.

## Additional Information

**How to cite this article:** Patthey, C. *et al*. Evolution of the functionally conserved *DCC* gene in birds. *Sci. Rep.* **7**, 42029; doi: 10.1038/srep42029 (2017).

**Publisher's note:** Springer Nature remains neutral with regard to jurisdictional claims in published maps and institutional affiliations.

## Supplementary Material

Supplementary File 1

Supplementary Dataset 1

Supplementary Dataset 2

Supplementary Dataset 3

## Figures and Tables

**Table 1:** The *DCC* gene was identified in different birds.

	Common name	Genomic assembly: Top hit using Falcon *DCC* sequence as bait	Genomic raw reads: Is *DCC* detected?	Transcriptomic data: Is *DCC* detected?
Galliformes	chicken	*Neogenin*	NO	NO
Galliformes	quail	*Neogenin*	NO	NO
Galliformes	turkey	*Neogenin*	NO	NO
Galliformes	guineafowl	*Neogenin*	NO	NO
Galliformes	brush turkey	none available	YES	none available
Anseriformes	duck	*DCC*	YES	YES
Columbiformes	rock pigeon	*DCC*	YES	YES
Cuculiformes	common cuckoo	*DCC*	YES	NO
Apodiformes	Anna’s hummingbird	*DCC*	YES	none available
Caprimulgiformes	chimney swift	*DCC*	YES	NO
Opisthocomiformes	hoazin	*DCC*	YES	YES
Chradriiformes	killdeer	*DCC*	YES	NO
Sphenisciformes	emperor penguin	*DCC*	YES	YES
Falconiformes	peregrine falcon	*DCC*	YES	YES
Psittaciformes	budgerigar	*DCC*	YES	NO
Passeriformes	rifleman	*Neogenin*	YES	none available
Passeriformes	hooded crow	*Neogenin*	YES	YES
Passeriformes	ground tit	*Neogenin*	YES	YES
Passeriformes	ground finch	*Neogenin*	NO	none available
Passeriformes	zebra finch	*Neogenin*	NO	NO

The common name and the source of data used (genomic assemblies, analysis of raw reads or transcriptomic data) to identify the presence (YES) or absence (NO) of *DCC* or the top hit gene identified using each method are indicated for each species.
