# Supplementary material for: Evolution of the functionally conserved DCC gene in birds
Source: Sci Rep. 2017 Feb 27;7:42029. doi: 10.1038/srep42029 (PMC5327406; doi:10.1038/srep42029)
Supplement: Supplementary File 1 [file srep42029-s1.pdf]

Supplementary file 1

Evolution of the functionally conserved *DCC* gene in birds.

Cedric Patthey, Yong Guang Tong[1], Christine Mary Tait[1] and Sara Ivy Wilson*

Umeå Center for Molecular Medicine, Umeå University, 901-87 Umeå, Sweden.

[1] equal contribution

*corresponding author: sara.wilson@umu.se

This file contains:

- **Figures S1 and S2.**
- **Supplementary tables S4 –S 9 and supplementary table legends for tables S1 – S9 (Tables S1 - S5 are provided separately).**
- **Supplementary references.**

Supplementary figures.

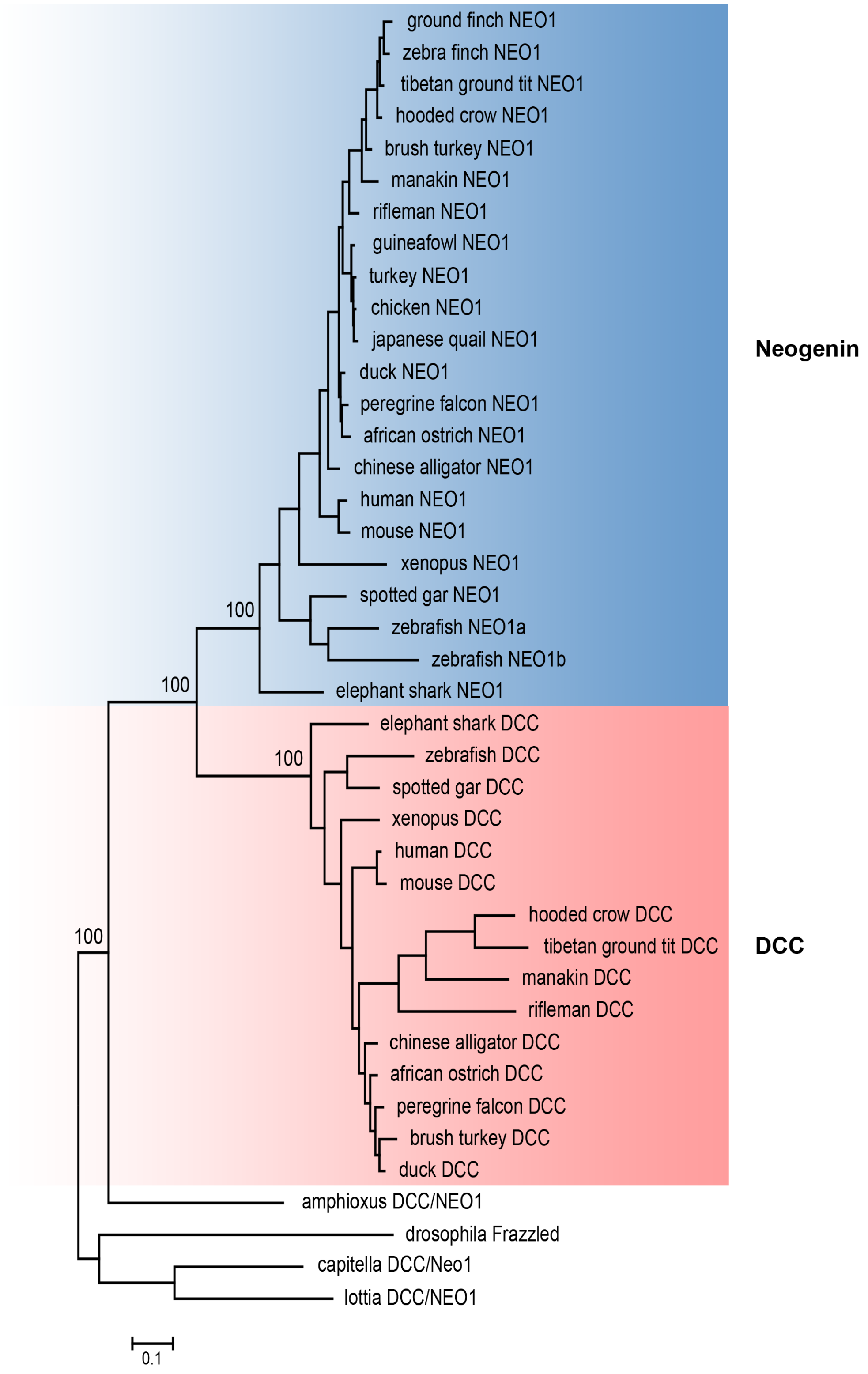

Figure S1: Phylogenetic tree of DCC (Pink) and *Neogenin* (Blue). Maximum likelihood amino acid tree of the DCC/Neogenin gene family including DCC and

Neogenin in various vertebrates as well as the common invertebrate orthologous (DCC/NEO1). Bootstrap values generated with 100 replicates are shown at each node. The scale bar represents 0.1 substitutions per site.

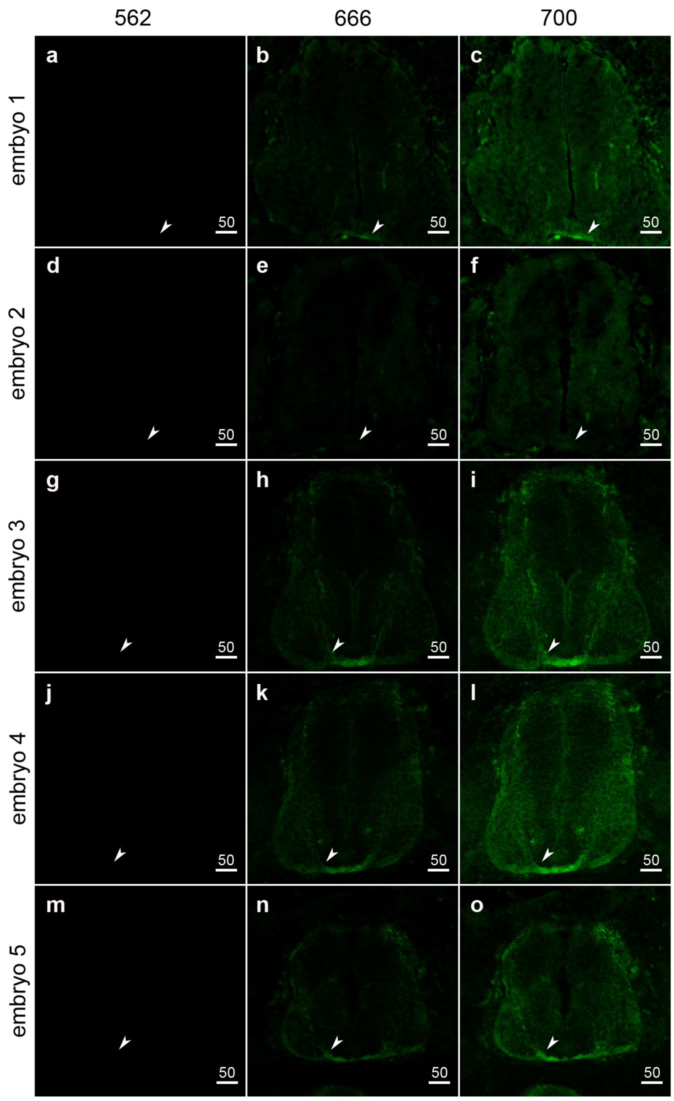

Figure S2: Weak labelling of caudal chick spinal cord sections with DCC intracellular antibody. (a - o) Photomicrographs of transverse sections of the embryonic lumbar spinal cord are shown in E5.0 –E5.5 chicken embryos labelled by immunohistochemistry with the DCC-intracellular antibody. To show the variation in labelling, sections from 5 different embryos are shown; embryo 1 **(a – c)**, embryo 2 **(d – f)**, embryo 3 **(g – i)**, embryo 4 **(j – l)**, embryo 5 **(m – o)**. Confocal images taken

with different confocal gain exposures are shown,: 562 (the same exposure/image settings as the images shown in Fig. 6), 666 and 700 gain exposure settings. The white arrowhead points to commissural axons. Representative images are shown. Scale bars are 50 μm.

Supplementary tables S6 –S9 and supplementary table legends for tables S1 – S9

Table S1: File uploaded separately – Patthey supplementary data set 1.xlsx. Nucleotide substitution rate raw data. Raw data values for the nucleotide substitution rate (branch length) calculations shown in Fig. 9 in addition to other birds not shown in the main figure. All birds that were calculated are shown. Gene name is on the top line and follows the duck synteny. The common bird name on the left side and the Latin name on the right side. A cross indicates that that gene could not be found in our analysis. A heat map is superimposed on the table to highlight the trends within the data. The values represent the total branch length from the crocodile-bird common ancestor to each particular species (represented as values between 0 and 1), measured in substitutions per site (called sympatric distance to the root). The lowest substitution rate is designated a light orange colour and increasing colour intensity represents higher nucleotide substitution rates. Of note the common cuckoo and budgerigar both had disruptions within the *DCC* locus but retained *DCC* itself. We observed a trend of higher nucleotide substitution rates in the remaining genes in the locus for these birds. Statistical analysis of these data are shown in Tables S2 – S5.

Table S2: File uploaded separately – Patthey supplementary data set 1.xlsx. Statistical analysis of nucleotide substitution rate data in Table S1. Salvaged

genes in Galliformes versus non salvaged genes. Statistical analysis of the nucleotide substitutions per site from Table S1 was performed using a non-paired, t-test as described in detail the methods. The raw data from Table S1 analysed is shown in the Table with the gene name at the top of the table and the species name at the left side of the table. The nucleotide substitution rate (branch length) data from Table S1 was used to determine any statistical significance between groups as follows: group 1 - nucleotide substitution rate of genes outside the disrupted locus (*NARS - TCF4* and *ACAA2 - CTIF*) versus group 2 - nucleotide substitution rate of 5 genes salvaged from the disrupted locus in Galliformes (*DYNAP, MBD2, SMAD 4, ME2, SKA1*). On the right hand side of the table the raw statistical analysis is shown for each comparison; whether the group passed the normality test, number of samples, mean, standard error of the mean and exact P value. The relative significance of the comparison between the groups is shown in the last column (not significant (n.s.), *, ** or ***). A heat map is superimposed on the table to highlight the trends within the data. This represents the total branch length from the crocodile-bird common ancestor to each particular species, measured in substitutions per site (called sympatric distance to the root). The lowest substitution rate is designated a light orange colour and increasing colour intensity represents higher nucleotide substitution rates.

Table S3: File uploaded separately – Patthey supplementary data set 1.xlsx. Statistical analysis of nucleotide substitution rate data in Table S1. Salvaged genes in Passeriformes versus non salvaged genes. Statistical analysis of the nucleotide substitutions per site from Table S1 was performed using a non-paired, t-test as described in detail the methods. The raw data from Table S1 analysed is shown in the Table with the gene name at the top of the table and the species name at the left

side of the table. The nucleotide substitution rate (branch length) data from Table S1 was used to determine any statistical significance between groups as follows: group 1 - nucleotide substitution rate of genes outside the disrupted locus (*NARS* - *TCF4* and *ACAA2* - *CTIF*) versus group 2 - nucleotide substitution rate of the genes salvaged from the disrupted locus in Passeriformes. On the right hand side of the table the raw statistical analysis is shown for each comparison; whether the group passed the normality test, number of samples, mean, standard error of the mean and exact P value. The relative significance of the comparison between the groups is shown in the last column (not significant (n.s.), *, ** or ***). A heat map is superimposed on the table to highlight the trends within the data. This represents the total branch length from the crocodile-bird common ancestor to each particular species, measured in substitutions per site (called sympatric distance to the root). The lowest substitution rate is designated a light orange colour and increasing colour intensity represents higher nucleotide substitution rates.

Table S4: File uploaded separately – Patthey supplementary data set 1.xlsx. Statistical analysis of nucleotide substitution rate data in Table S1. Salvaged genes in Galliformes versus orthologs where the locus was not disrupted. Statistical analysis of the nucleotide substitutions per site from Table S1 was performed using a non-paired t-test as described in detail the methods. The raw data from Table S1 analysed is shown in the Table S4 with the gene name at the top of the table and the species name at the left side of the table. The nucleotide substitution rate (branch length) data from Table S1 was used to determine any statistical significance between groups as follows: group 1: nucleotide substitution rate of orthologs from birds where the DCC locus was disrupted in Galliformes versus group 2: nucleotide

substitution rate of orthologs from birds where the DCC locus was not disrupted. On the right hand side of the table the raw statistical analysis is shown for each comparison; whether the group passed the normality test, number of samples, mean, standard error of the mean and exact P value. The relative significance of the comparison between the groups is shown in the last column (not significant (n.s.), *, ** or ***). A heat map is superimposed on the table to highlight the trends within the data. This represents the total branch length from the crocodile-bird common ancestor to each particular species, measured in substitutions per site (called sympatric distance to the root). The lowest substitution rate is designated a light orange colour and increasing colour intensity represents higher nucleotide substitution rates.

Table S5: File uploaded separately – Patthey supplementary data set 1.xlsx. Salvaged genes in Passeriformes versus orthologs where the locus was not disrupted. Statistical analysis of the nucleotide substitutions per site from Table S1 was performed using a non-paired t-test as described in detail the methods. The raw data from Table S1 analysed is shown in the Table S5 with the gene name at the top of the table and the species name at the left side of the table. The nucleotide substitution rate (branch length) data from Table S1 was used to determine any statistical significance between groups as follows: group 3: nucleotide substitution rate of orthologs from birds where the DCC locus was disrupted in Passeriformes versus group 2: nucleotide substitution rate of orthologs from birds where the DCC locus was not disrupted. On the right hand side of the table the raw statistical analysis is shown for each comparison; whether the group passed the normality test, number of samples, mean, standard error of the mean and exact P value. The relative significance of the comparison between the groups is shown in the last column (not

significant (n.s.), *, ** or ***). A heat map is superimposed on the table to highlight the trends within the data. This represents the total branch length from the crocodile-bird common ancestor to each particular species, measured in substitutions per site (called sympatric distance to the root). The lowest substitution rate is designated a light orange colour and increasing colour intensity represents higher nucleotide substitution rates.

Gene name	Species	Oligonucleotide primers sequence
DCC exon13	duck	5’ CCACCAGGCACTCAAAATGG 3’<br>5’ ACCAGAGGTTGTTTGGCTCC 3’
DCC exon17	duck	5’ CCCAGATATCTCCACCCCCA 3’<br>5’ AGTTGTCTGCCCAGATGACC 3’
DCC exon23	duck	5’ CCCCAGAAGAACAGCAACCT 3’<br>5’ CTCTGCTGTGCCGAGGAG 3’
DCC exon26	duck	5’ CTGTGGTCAGTGCCATCCC 3’<br>5’ GCTCCAAAGGTCCTGTCCAC 3’
POLI exon2	duck	5’ CACAGGCTGTGCAGGAGTG 3’<br>5’ TGGATGTGATCAAGGCTGCG 3’
MEX3C exon2	duck	5’ TTCTGCATGGAGTGTGCCAA 3’<br>5’ TGCCTGAGTAACAGCTGTCTG 3’
MBD2 exon 5	duck	5’ GCCCAGGTAACAACGACGAT 3’<br>5’ AGATGTGTTGAGCCAGACGG 3’
SMAD4 exon 1	duck	5’ CACAGCTTGATGTGCCATCG 3’<br>5’ CGCACTTGCTAGGATGAGCT 3’
TCF4 exon 17	duck	5’ AAAGCCGAGCGAGAAAAGGA 3’

		5’ TCAGGTGGAGTTGCACCATC 3’
MBD2 exon 5	turkey	5’ CCAGCGGGAAGAAATTTCGC 3’<br>5’ CTGCAATTTGCTCGGCATCA 3’
SMAD4 exon 1	turkey	5’ TTGAGCATCGTCCACAGCTT 3’<br>5’ GGTGATGAGCGAATCCAGCT 3’
TCF4 exon 17	turkey	5’ ACGACGACGAGGATTTGACC 3’<br>5’ TATCGCTCTTCAGGTGCAGC3’
MBD2 exon 5	chicken	5’ GCTCTCCATGTCGGTTCTGT 3’<br>5’ GGTGACAACGAAAGCTCTGC 3’
SMAD4 exon 1	chicken	5’ TCCACAGCCTCATGTGTCAC 3’<br>5’ GAGCGAATCCAGCTCGTCTT 3’
TCF4 exon 17	chicken	5’ GATCTGACCCCGGAGCAAAA 3’<br>5’ ACTCCTTGAAGGCCTCGTTG 3’
MBD2 exon 5	zebra finch	5’ CCATCACCGGCCAGAACTC 3’<br>5’ CGAATGTCGTCGTCGGTGA 3’
SMAD4 exon 1	zebra finch	5’ AGCTTGATGTGCCATCGACA 3’<br>5’ ACACTTGCTAGGATGAGCTCC 3’
TCF4 exon 17	zebra finch	5’ CAACGACGACGAGGACCTG 3’<br>5’ CTCCTTGAAGGCCTCGTTGA 3’

Table S6: Oligonucleotide primers used in gPCR in Figure 6.

**Gene name**	**Oligonucleotide primers used for cloning**
DCC	5’ CGAGCTCCCACTACGTGATC 3’

	5’ TGACGAGGAGGTTGCTGTTC 3’
Neogenin	5’ CACTCGGTGGCAGTAACAGT 3’<br>5’ ATTGGCCCTGTCTGAATGGG 3’

Table S7: Oligonucleotide primers used to amplify duck *DCC* and *Neogenin* cDNA.

		**Genome assembly searched**		
**species**	**latin name**	**assembly name**	**accession**	**references**
chicken	*Gallus gallus*	Gallus_gallus-5.0	GCA_000002315.3	[1]
quail	*Coturnix japonica*	Japanese quail assembly	DRA000595	[2]
turkey	*Meleagris gallopavo*	Turkey_5.0	GCA_000146605.3	[3]
guineafowl	*Numida meleagris*	-	-	no genome assembly available.
brush turkey	*Alectura lathami*	-	-	no genome assembly available.
duck	*Anas platyrhynchos*	BGI_duck_1.0	GCA_000355885.1	[4]
rock pigeon	*Columba livia*	Cliv_1.0	GCA_000337935.1	[5]

common cuckoo	*Cuculus canorus*	ASM70932v1	GCA_000709325.1	6
chimney swift	*Chaetura pelagica*	ChaPel_1.0	GCA_000747805.1	6
Anna's hummingbird	*Calypte anna*	ASM69908v1	GCA_000699085.1	6
hoatzin	*Opithocomus hoazin*	ASM69207v1	GCA_000692075.1	6
killdeer	*Charadrius vocifrerus*	ASM70802v2	GCA_000708025.2	6
emperor penguin	*Aptenodytes fosteri*	ASM69914v1	GCA_000699145.1	7
peregrine falcon	*Falco peregrinus*	F_peregrinus_v1.0	GCA_000337955.1	8
budgerigar	*Melpsittacus undulates*	Melopsittacus_undulatus_6.3	GCA_000238935.1	9
rifleman	*Acanthisitta chloris*	ASM69581v1	GCA_000695815.1	6
golden-collared manakin	*Manacus vitellinus*	ASM171598v1	GCA_001715985.1	6
hooded crow	*Corvus cornix*	Hooded_Crow_genome	GCA_000738735.1	10
Tibetan ground tit	*Pseudopodoces humilis*	PseHum1.0	GCA_000331425.1	11

medium ground finch	*Geospiza fortis*	GeoFor_1.0	GCA_000277835.1	[6]
zebra finch	*Taeniopygia guttata*	Taeniopygia_ guttata-3.2.4	GCA_000151805.2	[12]
African ostrich	*Struthio camelus*	ASM69896v1	GCA_000698965.1	[6]
Chinese alligator	*Alligator sinensis*	ASM45574v1	GCA_000455745.1	[13]

Table S8: List of genomic assemblies searched.

**Species**	**SRA Project**	**Sample type**	**Tissues**	**Number of reads**	**References**
chicken	PRJNA308865	RNAseq	cranial sensory ganglia	1,154,882,226	[14]
chicken	PRJEB5921	RNAseq	embryonic spinal cord	246,990,218	[15]
chicken	PRJNA257672	RNAseq	yolk sac	179,000,718	[16]
chicken	PRJNA177791	RNAseq	lung, liver, colon, brain	1,247,279,460	[17]
chicken	PRJNA246653	RNAseq	HH4-36 whole	1,926,209,281	[18]

			embryo		
chicken	PRJEB4677	RNAseq	various	3,776,860,404	-
quail	PRJNA296888	RNAseq	lung, muscle, liver, brain	1,970,984,192	-
turkey	PRJNA307355	RNAseq	liver	882,837,776	[19]
turkey	PRJNA251549	RNAseq	liver	469,657,575	[20]
guineafowl	PRJNA271731	RNAseq	ovary, spleen	999,124,076	[21]
brush turkey	PRJNA303085	genomic	non-applicable	9,593,178	-
duck	PRJNA194464	RNAseq	muscle, gut	623,625,926	[4]
duck	PRJNA189172	RNAseq	liver	157,493,832	-
duck	PRJNA271731	RNAseq	ovary, spleen	928,266,640	[21]
duck	PRJNA248056	RNAseq	fat tissue	350,741,284	[22]
rock pigeon	PRJNA170656	RNAseq	heart, liver	287,229,216	[5]
common	PRJNA212	genomic	non-	2,826,579,748	[6]

cuckoo	870		applicable		
Chimney swift	PRJNA210808	genomic	non-applicable	1,764,333,294	[6]
Anna's hummingbird	PRJNA212866	genomic	non-applicable	2,169,080,144	[6]
hoatzin	PRJNA212873	genomic	non-applicable	2029187160	[6]
killdeer	PRJNA212867	genomic	non-applicable	2,553,537,228	[6]
emperor penguin	PRJNA268920	RNAseq	muscle	12,043,109	[23]
emperor penguin	PRJNA308448	genomic	non-applicable	1,086,708,062	[24]
peregrine falcon	PRJNA159791	RNAseq	blood	79,571,270	[24]
peregrine falcon	PRJNA159791	genomic	non-applicable	1,967,741,514	[24]
budgerigar	PRJEB1588	ggenomic	non-applicable	3,162,684,266	[9]
rifleman	PRJNA212877	genomic	non-applicable	424,552,912	[6]
golden-collared manakin	PRJNA297576	RNAseq	muscle	687,959,522	[25]

golden-collared manakin	PRJNA320632	RNAseq	kidney, brain, liver	251,755,194	-
golden-collared manakin	PRJNA212872	genomic	non-applicable	2,017,182,860	[6]
hooded crow	PRJNA192205	RNAseq	brain, liver, skin and more	3,479,157,316	[10]
ground tit	PRJNA191625	RNAseq	muscle, liver	237,412,948	[26]
ground tit	PRJNA179234	genomic	non-applicable	1,839,177,476	[26]
medium ground finch	PRJNA156703	genomic	non-applicable	2,352,241,424	[6]
medium ground finch	PRJNA302636	genomic	non-applicable	1,041,990,058	[27]
zebra finch	PRJNA241410	RNAseq	forebrain	69,836,901	[28]
zebra finch	PRJNA314502	RNAseq	Brain	302,409,003	[29]
zebra finch	PRJNA264604	RNAseq	Brain	9,535,066	[30]

zebra finch	PRJNA297576	RNAseq	muscle	712,859,240	[25]
ostrich	SRP012236	RNAseq	brain, liver	587,599,446	[31]
alligator	PRJNA215016	RNAseq	testis, ovary, scales, blood	266,666,670	[13]

Table S9: List of raw reads databases searched.

Supplementary references

1 International Chicken Genome Sequencing, C. Sequence and comparative analysis of the chicken genome provide unique perspectives on vertebrate evolution. *Nature* **432**, 695-716, doi:10.1038/nature03154 (2004).

2 Kawahara-Miki, R. *et al.* Next-generation sequencing reveals genomic features in the Japanese quail. *Genomics* **101**, 345-353, doi:10.1016/j.ygeno.2013.03.006 (2013).

3 Dalloul, R. A. *et al.* Multi-platform next-generation sequencing of the domestic turkey (Meleagris gallopavo): genome assembly and analysis. *PLoS Biol* **8**, doi:10.1371/journal.pbio.1000475 (2010).

4 Huang, Y. *et al.* The duck genome and transcriptome provide insight into an avian influenza virus reservoir species. *Nat Genet* **45**, 776-783, doi:10.1038/ng.2657 (2013).

5 Shapiro, M. D. *et al.* Genomic diversity and evolution of the head crest in the rock pigeon. *Science* **339**, 1063-1067, doi:10.1126/science.1230422 (2013).

6 Zhang, G. *et al.* Comparative genomic data of the Avian Phylogenomics Project. *Gigascience* **3**, 26, doi:10.1186/2047-217X-3-26 (2014).

7 Li, C. *et al.* Two Antarctic penguin genomes reveal insights into their evolutionary history and molecular changes related to the Antarctic environment. *Gigascience* **3**, 27, doi:10.1186/2047-217X-3-27 (2014).

8 Zhan, X. *et al.* Peregrine and saker falcon genome sequences provide insights into evolution of a predatory lifestyle. *Nat Genet* **45**, 563-566, doi:10.1038/ng.2588 (2013).

9 Ganapathy, G. *et al.* High-coverage sequencing and annotated assemblies of the budgerigar genome. *Gigascience* **3**, 11, doi:10.1186/2047-217X-3-11 (2014).

10 Poelstra, J. W. *et al.* The genomic landscape underlying phenotypic integrity in the face of gene flow in crows. *Science* **344**, 1410-1414, doi:10.1126/science.1253226 (2014).
11 Cai, Q. *et al.* Genome sequence of ground tit Pseudopodoces humilis and its adaptation to high altitude. *Genome Biol* **14**, R29, doi:10.1186/gb-2013-14-3-r29 (2013).
12 Warren, W. C. *et al.* The genome of a songbird. *Nature* **464**, 757-762, doi:10.1038/nature08819 (2010).
13 Wan, Q. H. *et al.* Genome analysis and signature discovery for diving and sensory properties of the endangered Chinese alligator. *Cell Res* **23**, 1091-1105, doi:10.1038/cr.2013.104 (2013).
14 Patthey, C. *et al.* Identification of molecular signatures specific for distinct cranial sensory ganglia in the developing chick. *Neural Dev* **11**, 3, doi:10.1186/s13064-016-0057-y (2016).
15 Sasai, N., Kutejova, E. & Briscoe, J. Integration of signals along orthogonal axes of the vertebrate neural tube controls progenitor competence and increases cell diversity. *PLoS Biol* **12**, e1001907, doi:10.1371/journal.pbio.1001907 (2014).
16 Yadgary, L., Wong, E. A. & Uni, Z. Temporal transcriptome analysis of the chicken embryo yolk sac. *BMC Genomics* **15**, 690, doi:10.1186/1471-2164-15-690 (2014).
17 Merkin, J., Russell, C., Chen, P. & Burge, C. B. Evolutionary dynamics of gene and isoform regulation in Mammalian tissues. *Science* **338**, 1593-1599, doi:10.1126/science.1228186 (2012).
18 Gardner, P. P. *et al.* Conservation and losses of non-coding RNAs in avian genomes. *PLoS One* **10**, e0121797, doi:10.1371/journal.pone.0121797 (2015).
19 Monson, M. S., Cardona, C. J., Coulombe, R. A. & Reed, K. M. Hepatic Transcriptome Responses of Domesticated and Wild Turkey Embryos to Aflatoxin B(1). *Toxins (Basel)* **8**, doi:10.3390/toxins8010016 (2016).
20 Monson, M. S. *et al.* Response of the hepatic transcriptome to aflatoxin B1 in domestic turkey (Meleagris gallopavo). *PLoS One* **9**, e100930, doi:10.1371/journal.pone.0100930 (2014).
21 Wright, A. E. *et al.* Variation in promiscuity and sexual selection drives avian rate of Faster-Z evolution. *Mol Ecol* **24**, 1218-1235, doi:10.1111/mec.13113 (2015).
22 Chen, L. *et al.* Transcriptome analysis of adiposity in domestic ducks by transcriptomic comparison with their wild counterparts. *Anim Genet* **46**, 299-307, doi:10.1111/age.12294 (2015).
23 Figuet, E., Ballenghien, M., Romiguier, J. & Galtier, N. Biased gene conversion and GC-content evolution in the coding sequences of reptiles and vertebrates. *Genome Biol Evol* **7**, 240-250, doi:10.1093/gbe/evu277 (2014).
24 Cristofari, R. *et al.* Full circumpolar migration ensures evolutionary unity in the Emperor penguin. *Nat Commun* **7**, 11842, doi:10.1038/ncomms11842 (2016).
25 Fuxjager, M. J. *et al.* Research Resource: Hormones, Genes, and Athleticism: Effect of Androgens on the Avian Muscular Transcriptome. *Mol Endocrinol* **30**, 254-271, doi:10.1210/me.2015-1270 (2016).

26 Qu, Y. *et al.* Ground tit genome reveals avian adaptation to living at high altitudes in the Tibetan plateau. *Nat Commun* **4**, 2071, doi:10.1038/ncomms3071 (2013).
27 Chaves, J. A. *et al.* Genomic variation at the tips of the adaptive radiation of Darwin's finches. *Mol Ecol*, doi:10.1111/mec.13743 (2016).
28 Balakrishnan, C. N. *et al.* Brain transcriptome sequencing and assembly of three songbird model systems for the study of social behavior. *PeerJ* **2**, e396, doi:10.7717/peerj.396 (2014).
29 Davidson, J. H. & Balakrishnan, C. N. Gene Regulatory Evolution During Speciation in a Songbird. *G3 (Bethesda)* **6**, 1357-1364, doi:10.1534/g3.116.027946 (2016).
30 Lautwein, T., Lerch, S., Schafer, D. & Schmidt, E. R. The serine/threonine kinase 33 is present and expressed in palaeognath birds but has become a unitary pseudogene in neognaths about 100 million years ago. *BMC Genomics* **16**, 543, doi:10.1186/s12864-015-1769-9 (2015).
31 Adolfsson, S. & Ellegren, H. Lack of dosage compensation accompanies the arrested stage of sex chromosome evolution in ostriches. *Mol Biol Evol* **30**, 806-810, doi:10.1093/molbev/mst009 (2013).
