# Supplementary material for: Evolution of the functionally conserved DCC gene in birds
Source: Sci Rep. 2017 Feb 27;7:42029. doi: 10.1038/srep42029 (PMC5327406; doi:10.1038/srep42029)
Supplement: Supplementary Dataset 2 [file srep42029-s3.doc]

Evolution of the functionally conserved DCC gene in birds.

Cedric Patthey, Yong Guang Tong1, Christine Mary Tait1 and Sara Ivy Wilson*

Umeå Center for Molecular Medicine, Umeå University, 901-87 Umeå, Sweden.

1 equal contribution

*corresponding author: sara.wilson@umu.se

Supplementary data set 2

>African ostrich ACAA2

ATGGCGCTCCTGCGGGGTGTATTCATTGTTTCAGCGAAGCGAACTCCCTTCGGGGCCTATGGAGGGTTGC

TCAAAGACCTCACGGCCATAGATCTGGCAGAGCATGCTGCCCGAGCTGCACTGGTGGCTGGCAAGGTCTC

TCCTGAGATCGTTGACAGTGTCATTGTTGGCAATGTCATGCAGAGCTCCGTAGATGCTATTTATATTGCA

AGACATGTTGGTTTACGTGTGGGAATTCCTGTCCCAGTTCCAGCCCTCACTATCAGCAGACTCTGTGGCT

CTGGTTTCCAGTCCATTGTCAATGGATGTCAGGAAATTTGCCTTAATGACTCAGAAGTGGTTCTGTGTGG

TGGAGCTGAAAATATGAGCCAATCTCCTTACGCTGTTCGAAACATTCGATTTGGAACCAAATTAGGAGCA

GATTTGAAGTTGGAAGACACGTTGTGGGCAAGTCTAACTGACACGCATGTTAAAATACCTATGGCAGTTA

CAGCTGAAAATCTGGCTGTAAAATACAACATCACACGAGAGGACTGTGACCGATATGCAGTCAAAACGCA

ACAGAGGTGCAAAGCCGCTTATGATGCTGGTTACTTTAATGCTGAGATGGCACCAGTTGAAGTGAAAACA

AAAAAAGGGAAAGAAAGTATGCAAAAGGATGAGCACCCAAAACCCCAGACTACGCTGGAACAATTGGCAA

AGCTCCCACCTGTCTTTAAGAAGGATGGAACGGTCACTGCTGGGAATGCTTCAGGTGTGTGTGATGGCGC

TGGAGCGGTCGTCATTGCCAGTGAATCAGCACTTACAAAGCACAGTCTTACTCCACTGGCGAGAGTAGTA

GCTTATCACTCATCTGGCTGCGACCCTACCATAATGGGCATTGGCCCTGTACCTGCAATTACTGAAGTCC

TGAAGAAGGCAGGACTGACCCTGAAGGACATGGATTTGGTGGAGGTGAATGAAGCATTTGCACCCCAGTA

TCTAGCAGTTGAAAAAGCTTTGGGCCTTGACCCTGAAAAAACCAATGTCAATGGAGGTGCCATCGCAATT

GGTCATCCTCTGGGTGCTTCGGGATCGCGGATCACAGCTCATCTGGTTCATGAATTAAGGCGTCGTGGTG

GGAAATATGCAGTTGGGTCAGCTTGCATTGGAGGTGGACAAGGTATTGCTCTCATCATTGAGAACACAGC

CTGA

>African ostrich C18ORF32

ATGGTGTGCATTCCTTGTATTGTCATTCCGGTTCTCCTCTGGGTCTATAAGAAATTCCTTGAACCGTATA

TCTATCCTGTTATTGCACCTTTCATTAAACGTGTGTGGCCCAAGAAAGCTGTGCAAGAAACAACAGCCAC

AAAGCAAGGTCAAGGAGTTGGCGCTGGAAATCCACAGGCACCTTCAGCCACAAAAAGAGATCAGGAGGAT

GAGTCTGGAATTTATAAATTTGAAAGCAATGGGTTTGCAAATGGAATTGCTGCAAAGAGAGACACAGAAG

TTTCTGACAAGAAAACAGATTAA

>African ostrich C18ORF54

ATGGCAAGCTCCATAAAAAAATTCAGTGTCAGCTCTCCTGACTCAACAGTATCTTCTCTCCTAGCAAGCT

ATGGCACTGACAGCAATAATCCGTGCTCAAACAATGTGATTTGCTACAAGGACAAGCTTTACAGCTCTGC

ATCTGAGGCTCTGGAGGCATATATTGAAGATTTTGATTTAAGCCTCATGTCTCCAGAAATAAGCACTGGA

AAAATCTGCATAAGTCAAAGCACTCCCAAACATACTGAGTTTTCCAAGTACCATGCCAAAGGAAGATATG

TATTGGAGGACTTTAATCATCGTGCAGGATTAGGTTCTACTGCTTCATCCTGTAGAAGGAGGACTGAGTG

TGACCCAGACTTGATTAGCCTCACAACAGATGACCTATTGGCTTTTCCAGCAGATGGATCTCAGCCCTTT

GTCCAGCGCCCTCCTTTTAAAACAAGGCATCAAAATAGTGAGTGCAGCAGGCGGTCGCTTAAAAAGTCTT

TCTACCCTTACCAAACCTCATCACTTGATATTGAGAAGGATTTCTGTCTCCAAGAGAATGACAAATCTCT

TGCTAATCAGAAGTTACACAAGAGCTTCTGCAAAAAGAAGCACAGCATATGTACGTCTCATAGGTACGGT

TCTGTCTCTTGTAAAGGAAATCCAAGAGCCTTGTCTTTTGAAGAGAACTCTAATGCTTTTCCTGTTAAGA

ATTATCCAAGATGGCTTACTAGTGGGAAGCCTGACTTAAGCGTGTCAGGGATAAGTAGTATTCCAAATTT

TGACTACCCAATCTGGCTTAACAGTCATAACCTTTTTTCTGATTCAGCTAATGAAGGTGATGGTCAAACT

TTTAATATGCGACATGAAACTTCCTCTTCACAAACTTCCGAGATCCTGAAAAGGAGACACTCCATGGATA

AAGACAATTCTAATTTTTTTGAACACAATGATTGCCTGGATCTAATAGGTGATAACGAAATAGCAGAAAG

TTGCAACTATGACAGTCCGGATTCATGCTTCCAATTCGGTAACTCCTTTTCAAGACACACCAAAGAGCCA

TTTAGAGAAGATCGGCTTGAGCTACTTACCTTGAAGACTGAGAAATCTCTGGAGACTTCAACTGAAGATT

TGTCAAATTCTCTGAAAAATGATGACAGTCCTTCTACAACAGATATCCTAGAAGCAGAAAGATCATGGGA

AAATATTCCAGTTGCTTTCAAATCACCAGTGCCTGTACGCTGTGAGGATGAGGAGAATACTCTACCATTC

CCTAAGGCAAATATAGTTCATGAGTTCTTCGAAGACTGTATAAATGACACAAACAAGGAAAATACCTTTT

CTGGAGGTAATCATCATGGACCAACTGAAACTTTAAAGCTAATGTTGTTTAACCTTCAAGCAGTTCATGA

AAGTTTAAACCAGAATAAAATTACTGAAGAAAAGGAAGAGTTTGAAAAACTTTCTGAAAAAGCAGATGCT

GAATTAGAAGTGTGTGACAGTGAGATGATCCCTCTTACTAATTCTCTTCAGAAGGCTTTACACCATTTGT

CTCGTCTTAAAAGTCTTGTTGAAGATAACAGTAACAAGCAAGAGCAAGCTGATGATCACCAA

>African ostrich CCDC68

ATGTCCTCCCCAGCCGGAGAACAGAGAAACGCACGTATAGTGACAACTACCCTGCTGCTGACTGAACATA

TAACGAGAGAGGACCGAGGCTCCGAGGGAAATTACCTCCTCTATGGATCCTCTTGTGCCCAGATCACCGA

AGAAGCTGAATATGTGAAAAAGCAGCTTCCTCAAATCTCGGGCAACAAACTGGAATCAAAGAACAGTATG

TCTAGCTCCAACTACAGTCCTATTGTGGTGAAGATGGAAGAAACGGAACACCAACTCTTGCTGGTGAACA

AGGAAAATCAAGTACTAAAGATCAAGCTGGAAGCTACAAGAGAAGCAGGTGTGCAGGCTCTCAGATCTGC

CTCCCAGACACTTTATGAAAATTACCATACTCGGTCAGAAGAACTTAAAAAAAGGCATGAGAATGATAAG

AAGCAAATACAGGCCCACAATCTCGAACAAGAACGAAAGCTCCAGCAGAATACAGAAAAAGCCAATCACC

TAGCTGAAGGAATCACAGAAAAATGCACTCAGATTACAGAAATGGAGAAAAGAGTGCAAAGGATGGAAGA

GGAAAAGAAAACTCTGATGGAAAGGAAACTGTCACTTGAACAGAAGCTTCTACAGATGTCAAAGAAAGAA

GACAGCAAGCGGTGTGTGGCTCTTCAGATGGAGATTTCCACTCTGCAAGAACAGATTTGCCATTTGCAGC

ATGTGATCCAGGCACAGCATCAGAACCTGCGCAGTGTGATACAGGAGGTGGAGGAACTGAACAAGGAACT

CAGAAACCAAGATAAAAAAATAGGAAATCTGACAGAAAAGCTAAATGCACTCGAAATTCAGAATAAAGAA

CTTAAAGAAAAAGTGGAATTCTGGTCTGGCCAATCCAAGACTAAAATTTCAAAAGGTGTCATGACAGACA

CACGACGAGACTTTGGAGCATCACCTTACTTGATGCTCACCAGGATAAGGAAGCAAGAAAGCTAG

>African ostrich CFAP53

ATGNNNGCGGTGGAGGCGCGATGGCACCGGCGGCACCGCGGGGGCCGGGCGCCCGCCCGGCGCTCCTTGG

CGCTGAGGTCCAAGCCTCCTAAACAAGATGTATGTGAAAAACTTGCCTTAACCTGCAGAAAGAAGGAAAA

GGAACTCCTTAAATGTATTGATTTCTCCATGCTGTACAGCAAGTATCGTAATATCAATGAGCAGCAGGAA

CGCAGTGAACGCAAAAGACTGCACAACAGTGTACAGAGAAGGGTAGATGAGACAATGCAGGCATACCTGG

CAGAGACTGAAGACAGGAGAGAGAGGCTTCGTGAACTTTTGGAATCAGAGGAAAGCAGGTACTTTGCTGA

GATGGAGTCACTTGAAGAAACTGTCCTGGAAAAACAAGCAAAAATGAGGGAGCGAGCAAAGTTGCTGAGA

GAGAAGAGAGAGCAAGAAAGACAAAAACTGGTTACTGAAAAACGGGAGCAGCAATTCAGAGAACAATGTG

AGGAGTTTCGCACGTGGTGGGTGCAGAAGCACCGCAGGGAAGTGTGTGCAGACCGACTGGCCCAGCTAGC

TCTTAAGGAGGAATTGAAAAAGCAACAACAGAAGGAGGAGCAAATGTTTGCCGAGCTTTGGGAAGAGGAT

AGGTTGGCCAAGGAAAAACGAGAGGCGGCGGATATGCAGAAAGCAGCAGAACAACATCGAGAAGTGCTGA

GCGTCCTCGGAGCCCAGGTAGCCGTGCTGAACGCTCACAGAGAGGAAGCAAAGCGGCTGAAAGAAGAAGA

GGCTCGCTTGCTGGAAGAAGAAAAGCAACTGCTTAAACTAGAAAGCGAACGACTTCAGATGGAGAAATTA

CAAAAGCAGAAGGAGTGCGGGGACATGTTGGTCAGTGCAGCACAGGACAAGATGAAGCGTCTGAATGAAG

AAAAACAGGAGGAACTTGCCCTAGATATGAAGATCCTAGAAAAAACTCTTCAGGAATCCCAAGGGGCCAC

AGAGGAGAAAAAGAAAAGAAAACAAGAGCTTTTCAAGGAGCAGCAGACTTACTGGGCACACCTGGCACAA

CAGCTGGAGGAGGAGAAACAGCGAGAAAAAGAAGCGGACAAGCTCCTTGATGAAGAGATGGCGAAGGCTT

GGGCCAAGAAAGCTGAGCAATTGCGATTAGAAAAGGAAGCTAGAAAACAGCTACTGAAAGATGTCCTGGA

TACAAGACAACTGCAGATTGAGGAGAAGTTGGAGAGAAATGCAAAGGAACAGGAAGAACTTGCCCAAGAA

AGGAAGCTATTTGCTGAAGCAATCACAGAACTCAAACGTATAGAAGAAGAAAAATACGCAAGAAAAGTAA

AGGAAGCAAAAGAATACCAAGAGCAACTCAAGGCTCAGATTGCCTATCAACAACAAGCCCGTGATGCTGA

GGAAGAAGAGAAGCAGCGAGAATATGAATCAGCCCTGGCAGCAGAGAGAGCTTACCAGAAAAGAATAGAG

GATATTCTAGCAAGGCCTTCTGTGACACTAGCAGAAATCCATCCTTTGAGGAGGAGACTCTTGTCTAGCA

CACAAGATCACTTATGA

>African ostrich CTIF

ATGGAAAACTCGTCTGTGGCGTCTGCCTCCTCTGAGGCAGGGAGTAGCCGTTCTCAGGAAATTGAGGAGC

TGGAGAGATTCATTGACAGCTACGTCCTGGAGTACCAAGTCCAAGGCTTGCTGACCGATAAAACGGAGGG

GGACGGCGAGAGTGAGAAGACGCAGTCCAACATCTCACAGTGGACGGCGGATTGTAGTGAGCAGCTTGAT

GGCAGCTGTTCCCCATCGAGAGGGAAGGGCTCGTCGGCTCACGAACACAATCAGAATGGCAACAAAGAGA

GCTCCCTTGACATGCTGGGCACAGACATCTGGGCAGCCAATACCTTTGACTCGTTCAGTGGTGCGACGTG

GGACTTACAGCCTGAAAAACTAGACTTCACCCAATTTCACAGGAAGCTCCGAAACACCTCCAAACACCCG

CTGCCTCACATAGACAGAGAAGGGCTCGGCAAAGGGAAATATGAGGATGGAGACAGCATCAACTTGAACG

ACATAGAGAAAGTCCTTCCCGTGTGGCAGGGTTACCATCCATTGCCTCATGAAGCTGAAATCGCACACAC

CAAAAAACTGTTCAGAAGGAGGAGAAATGACCGCAGGCGACAGCAGAGACTTCCTGGTGGGAATAAATCT

CAGCAGCACACAGATCATCAGCAAGGTGGCACCAAACACAACAGGGAACACCAGAAACTCTACCAGGGAG

GCCAGGCCCCTCACTCCTCAGGCAGGACGGGCCACCACGGCTACAGCCAGAACCGGAGATGGCACCACAA

CCAGAAGCACTCACCCAACGACAAAGAAACGCACAGAAATGCCAAAGAGACTGAGAATCTGAAAATCGAG

GACACCTCCGTCTGCACGGTGCATATTCCCTTAGAAATGCACCGGGGCCCGGAGGCTGTGGAGAAACAGT

CTCAGCAGTACACCCAGGAGTCAGAAACCAAGCGGAAAGACAGTATTCACGAGCGCATTGGGGAAAGACC

CAAGATCAATTTGCTTCAGTCTTCCAAAGACAGGCTGCGAAGGAGACTAAAAGAAAAGGACGAAGTCACG

GTGGAAACCACCAATCCCCAAAAGAACAAAATGGACAAATTAATTGAAATCCTCAACAGCATGAGGAACA

ACAGCAGCGATGTTGACTCCAAGCTAACCACATTCATGGAGGAGGCCCAGAACTCCACCAACTCAGAGGA

GATGCTGGGTGAGATCGTCAAGACCATCTATCAGAAAGCAGTGACAGACCGCAGCTTTGCTTCCACAGCT

GCCAAGCTCTGTGATAAAATGGCTCTCTTCATGGTGGAAGGAACCAAGTTCCGGAGCCTGCTCCTCAACA

TGTTGCAGAAGGATTTCACCATGAGGGAGGAGTTGCAGCAGCGGGACGTGGAGCGCTGGCTGGGCTTCAT

CACCTTCCTCTGCGAAGTCTTCGGCACCATGAGGAGCAGCACCGGAGAGCCCTTTCGAGTCCTTGTCTGT

CCCATTTATACCTGCCTCAGGGAGTTGTTGCAATCTCAGGATGTGAAGGAAGACGCTGTCCTTTGCTGCT

CCATGGAGCTGCAAAGCACCGGCCGGCTGCTGGAGGAGCAGCTGCCCGAGATGATGACGGAGCTGCTGGC

CATCGCCCGCGACAAGATGCTGTGCCCCTCGGAGTCCATGCTGACGCGCTCGCTGCTGCTGGAGGTCATC

GAGCTGCACGCCAACACCTGGAACCCGCTGACACCCACCATCACGCAGTACTACAACAAGACCATCCAAA

AACTGACGGCCTGA

>African ostrich DCC

ATGGAGAATAGTCTCGGATGTGTTTGGGTACCAAAGCTGGCTTTTCTGTTCTTCGGGTTTACGCTGCTCA

GCCCGCATCTTCGAGTCGCCGGTTCCCAGGTCAAGGGGTTTACATCACTGAGGTTCTTGACGGAGCCTTC

AGATGCTGTCACTATGCGTGGAAGCAACGTGCTCTTGAACTGCGCAGCCGAGTCGGATCAAGGAGCTCCA

GTTATTAAATGGAAGAAAGATGCAGTCTTCTTAAACCTGGCAGTAGATGAAAGGAGACAGCAGTTGGCCA

ATGGCTCACTCTTGGTACAAAACATAGTCCATTCAAGGCACCACAAGCCAGATGAAGGTCTCTACCAGTG

TGAAGCATCTTTAGAAGGCATTGGAGCTATCATCAGTCGGACAGCTAAGGTCATGGTAGCAGGACCGCTG

AGGTTTCTTTCCCAGACGGAATCTGTCACGGCTTTTGCAGGAGACACGGTTTTATTGAAGTGTGAAGTTG

TTGGAGAGCCCATGCCCATGGTACACTGGCAGAGAAACGAGGAGGACTTGTTCCTGAGCCCAGCTGACGC

ACGGGTTGCTGTCCTGCCCTCTGGAGCTTTACAGATTAGCAGAATTCAGCCGGGGGACAGTGGAATCTAT

AGATGCCTGGCAAAAAATCCAGCGAGTTCAAGAATCGGAAATGACGCAGAAGTCAGGGTTTTGGCAGATC

CGGGTTTGCACAGGCAACAGGTTTTCCTTCAGCGACCGTCGAACGTGGTGGCCGTGGAGGGGAAGGACGC

CGTCCTGGAGTGCTGCGTTTCCGGATACCCCCATCCCACCTTCACGTGGCTGCGCGGAGACGAAGTGCTC

CCCGTCAGGTCCAGAAAGTATTCTTTACTGGCTGGCAGTAACTTACTCATTTCTAATGTGACTGATGACG

ATTCTGGGACATACACTTGTGTCGTCTCCTACAAAAATGAGAACAGCAGTGGTTCTGCAGAGCTGTCGGT

GATGGTTCCACCATGGTTTTTAATTCGCCCTTCAAATCTTTATGCCTATGAGAGTATGGATATTGAGTTT

GAATGTGCTGTATCCGGTAAGCCAGTTCCGACGGTGGAGTGGATCAAGAACGGCGAAGTGGTCATTCCTA

GTGACTATTTTCAGATAGTGGGTGGCAGCAACTTGCGGATTCTGGGCTTGGTAAAGTCAGATGAAGGTTT

TTATCAGTGTGTAGCTGAAAACGATGCTGGAAACGCACAGACCAGCGCACAGCTAATCATCCCGGAGCCT

GCCGTCCCGAGCTCCAGTGTCCTCCCCTCTGCCCCCCGAGATGTCGTCCCTGTCTTGGTCTCCAGTCGGT

TTGTCCGTCTCAGCTGGCGCCCACCCGCCGATGCGAGAGGGAGCGTCCAGGCGTACACGGTCTACTTCTC

CAGGGAAGGTGTCAACAGGGAGCGGGCGCTCAACACGTCTCAGTCTGGGATGCTTCAGCTGACGGTGGGA

AACCTGAAGCCGGAGGAGACCTACACCTTCCGCGTGGTGGCCTACAACGAGTGGGGGCCCGGAGAGAGCT

CACAGCCCGTCAAGGTTGCCACACAGCCCGAGCTGCAAGTTCCAGGGCCAGTAGAAAACCTGCGGGCTGT

ATCTACCTCACCTACCTCGATCCTCATTTCCTGGGATCCCCCTGCCTATGCAAATGGTCCGATTCAAGGT

TACCGGCTCTTCTGCACCGAGACCGCAACTGGAAAAGAGCAGAATATCGAGGTCGACGGACTCTCCTACA

GGCTGGAAGGGCTGAAGAAATTCACGGAATACACTGTGCGCTTCCTGGCGTACAACCGCTACGGCCCCGG

TGTGTCCACCGAAGAGATCGCGGTCACGACGCTTTCTGACGTACCGAGCGCGATGCCTCAGAACGTCTCC

TTGGAAGTGGTTAATTCGAGGAGCATTAAAGTGAGCTGGTTGCCTCCACCACCAGGTACACAAAATGGAT

TTATTACGGGCTATAAAATCCGACATAGAAAGACTACCCGCAGGGGCGAGATTGAAACACTGGAGCCAAA

CAACCTCTGGTACTTGTTCACAGGACTTGAGAAAGGAAGCCAGTACAGTTTCCAGGTTGCTGCCATGACA

GTGAACGGGACTGGACCCCCCTCGGACTGGTACACAGCAGAGACGCCAGAGAATGATCTTGACGAATCTC

AGGTTCCTGACCAGCCAAGCTCTCTTCATGTCAGGCCATTGACAACAAGTATCGTCATGAGTTGGACTCC

GCCGCTGAACCCAAACATTGTTGTCCGTGGGTACATCATCGGCTACGGCGTAGGCAGTCCATATGCTGAG

ACTGTGCGGGTGGACAGTAAACAGCGTTATTATTCCATTGAAAATTTGGAGCCGAGTTCCCATTACGTAA

TTTCCTTAAAGGCCTTTAACAACGCAGGCGAGGGAGTGCCCCTGTATGAAAGCGCGACCACCAGGTCCAT

GACAGACCCCATTGATCCATTAGAAGTTGATTTTTATCCTTTGCTTGATGATTTCCCTACCTCAGTCCCA

GATATCTCCACCCCCATGCTCCCACCAGTAGGTGTCCAGGCTGTTGCACTTACCCATGATGCAGTGAGGG

TCATCTGGGCAGACAACTCTGTCCCGAAGAACCAAAAGACTACTGAGGTTCGCTTTTACACTGTCCGATG

GAGAACCAGCTATTCTACAAGTGCTAAATACAAGTCGGCGGATACGACAGCTTTGAGTCACACCGTGATA

GGCCTTAAGCCAAACACCATGTACGAGTTCTCCGTCATGGTGACCAAAGGTCGGCGGTCGAGCACGTGGA

GCATGACCGCACACGCCACAACCTATGAAGCAGCTCCAACCTCTGCACCCAAGGATTTGACAGTCATTAC

ACGGGAGGGGAAGCCCCGAGCTGTCATTGTCAGCTGGCAGCCACCGTTAGAAGCCAATGGAAAAATTACT

GCGTACATCCTCTTCTATACTTTGGACAAGAACACCCCCATTGATGACTGGATTATGGAGTCCATCAGCG

GTGACCGACTTACCCACCAAATCATGGACCTCAACCTAGACACCATTTACTACTTCCGAATCCAGGCTCG

CAACGCCAAAGGAGTGGGGCCTCTCTCTGATCCGATTTTCTTCCGGACGCTGAAAGTCGAGCACCCTGAC

AAAATGGCTAATGACCAAGGTCGCCATGGAGATGGGTCCTACTGGCCAGTGGATACGAACCTGATCGACA

GAAGCAGTCTGAATGAACCTCCCATAGGGCAGATGCACCCTCCGCATGGCAGCGTCACACCTCAGAAGAA

CAGCAACCTGCTTGTCATCATCGTCGTCACCGTCGGGGTCATTACGGTGGTGGTCGTGGTGATCGTCGCT

GTCATCTGCACCAGGCGTTCCTCGGCGCAGCAGAGGAAGAAACGTGCCACCCACAGTGCTGGTAAAAGAA

AGGGCAGTCAGAAGGATTTGAGGCCTCCAGATCTCTGGATACACCATGAAGAGATGGAGATGAAGAACAT

CGAGAAGCCGGCGGGCTCCGACACTGCAGGAAGAGAGTCCCCCATACAGAGCTGCCAGGACATCACCCCC

GTCAGTCACAGCCAGTCCGAGACGCAGCTGGGCAGCAAGAGCGCCCCGCAGTCCGGTCCTGAGACAGAGG

ACGCTGGAAGTAGCATGTCCACGTTGGAGCGCTCACTTGCCGCCCGCAGAGCCACCCGTGCCAAGCTCAT

GATTCCCATGGATTCACAGCCAAACAACCCTCCTGTGGTCAGTGCCATTCCAGTGCCAACACTAGAAAGT

GCCCAGTACCCCGGGATCCTGCCATCTCCCACCTGTGGATACCCACACCCTCAGTTCACTCTTCGGCCTG

TGCCATTCCCAACGCTCTCTGTCGACAGGACCTTTGGAACAGGAAGAACTGTAAACGAAGGCCCGGCATC

CCAGCAGCCGTCCTTGCTACCACCGGCGCAGCCTGAACACTCAAGCAACGAGGATGCCCCAAGCAGAACG

ATCCCCACGGCCTGCGTCCGCCCCACGCACCCCCTCCGCAGCTTTGCCAACCCCTTGCTACCTCCACCCA

TGAGTGCAATAGAACCGAAAGTCCCTTACACACCGCTTCTGTCTCAAACGGGGCCTAACCTTCCCAAGGC

TCAGGTTAAAACAGCGTCGCTTGGATTGGCAGGAAAGGCAAGATCACCGTTGCTACCGGTGTCGGTGCCC

ACAGCCCCGGAGGTCTCCGAGGAGGGCCACAAGCAGACGGAGGACCCCGCTAATGTTTATGAACAGGATG

ATCTGAGTGAACAGATGGCCAGTTTGGAGGGGCTAATGAAGCAACTCAATGCTATCACAGGCTCAGCCTT

CTAA

>African ostrich DYM

ATGGGAGCAAATAGCAGCAGCATCAGCGAACTTCCAGAAAATGAGTACTTAAAAAAGTTATCAGGAGCAG

AGCCAATCTCTGAGAATGACCCATTCTGGAATCAGCTATTATCTTTTAGCTTTACCGCTCCAACAAACAG

TGCTGACTTAAAGCTCCTGGAAGAAGCCACTATCTCAGTCTGCAAGTCTTTAGTTGAGAAGAATCCTCGA

ACAGGAAACCTTGGGTCGTTGATTAAAGTCTTTCTTTCTAGAACCAAAGAATTAAAAATTTCGGCAGAAT

GTCAGAATCACCTCTTTATTTGGCAAGCTCACAATGCATTGTTTATTATCTGCTGTTTGCTGAAAGTATT

CATCAGTCGAATGTCCGAAGAGGAACTACAACTTCATTTTACTTACGAAGAGAAAACACCAGGCTCATAC

GGAACAGAATGTGAAGATCTCATAGAAGAGCTGCTGTGTTGCCTCATCCAGCTCATTGTTGAAGTTCCCC

TCTTAGATATAACATACAGCATTTCCTTGGAGGCTGTGACAACGCTTATTGTCTTCCTTTCCTGCCAATT

ATTTCACAAGGAAATTCTGCGAGAGAGCATCATTCATAAATACCTGATGCACGGTCGATGTCTCCCATAT

ACCAGCAGACTTGTGAAAACTTTACTGTATAACTTCATTAGACAAGAAAGAAGCCCGCCTCCAGGGACCC

ATGTCTTTCAGCAGCAAACAGATGGAGGAGGACTACTTTATGGAATTGCCTCTGGGGTGGCAACTGGCCT

GTGGACAGTCTTCACATTAGGGGGAGTGGGGAGCAAAGCAACGCCGCAGCTGGAACAGTGCTCCCCTCTA

GCTAATCAGAGTCTGCTGCTTCTGCTCGTCTTGGCCAACCTGACCGATGCTCCAGATACGCCGAACCCCT

ACAGACAAGCTATTATGTCCTTCAAGAACACACAGGATAGCACTGCTTTTTCTTCATCGAACCCACACGC

TTTCCAGATTAATTTTAACAGTTTATACACGGCTTTGTGTGAGCAGCAGAAATCCGATCAAGCAACTCTT

CTTTTATACATGCTTCTGCATCAAAATGGCAATGTGCGGACATACGTGTTGGCACGCACGGACATAGAAA

ACCTTGTTTTGCCAATTCTTGAAATTCTGTATCATGTCGAAGAAAGGAATTCACACCATGTTTACATGGC

TCTTATAATTTTGTTGATTCTTACAGAGGACGATGGCTTCAATCGATCCATTCATGAAGTGATACTGAAA

AATATCACTTGGTATGCTGAGCGTGTTTTAACAGAGATCTCACTTGGGAGTCTTCTGATACTGGTCGTGA

TAAGAACCATCCAGTACAACATGACACGGACAAGGGACAAGTACCTTCATACAAATTGTCTGGCAGCCTT

AGCAAATATGTCAGCACAGTTCCGCTCACTTCATCAGTATGCTGCTCAGAGGATCATCAGTTTGTTTTCT

TTGTTATCCAGAAAACACAACAAAGTTTTGGAGCAAGCCACGCAGTCCTTAAGAGGTTCCCTCAGTTCAA

ATGACTCTCCACTTCCTGATTATGCGCAGGACCTGAACGTGATCGAGGAAGTGATCCGAATGATGCTGGA

GATTATCAACTCCTGCTTGACAAATTCCCTCCATCACAACCCGAACTTGGTGTACGCTCTGCTTTACAAG

CGGGATCTGTTTGAGCAATTTCGAACTCACCCTTCCTTCCAGGACATAATGCAAAATATAGATCTGGTGA

TCAGCTTCTTCAGCTCACGATTAGAGCAAGCTGGAGCCGAGTTGTCAGTGGAAAGAGTTCTGGAAATCAT

CAAACAAGGAGCTGTTGCTTTGCCCAAAGACAGGCTGAGAAAATTTCCTGAGCTGAAGTTCAAGTACGTG

GAGGAGGAGCAGCCGGAGGAGTTCTTCATCCCGTACGTTTGGTCCTTGGTTTACAATTCGGCGGTGGCCC

TGTACTGGAACCCACGCGACATCCAGCTCTTCACCATGGACTCGGGCTGA

>African ostrich DYNAP

ATGGATAACCAGGCATTTGAAATGCACGGAGAGAGCATACCAAGTTCTTCAAAAACAACAGAATGGCCAA

AGAAAGAGGAAAGAAAGAGCTACTGCTCCCTAATGAAAATATTTCTAGTTTGTCTGTTGGCCTGTGTTAT

CACCACCACAATAGGAGTGCTGGCCCTGTCTTTCATCTATGCAGAAAACATTGGCTTTCTAAAACAGACA

GATGTTAAAAACTCGGACACAACCCCCCCGAAAGCAGATGAAAAAGGAGTGGATATCAAATTCCGGTTCC

TGAATCAGCTGGGAAAATCCAAGGTACATCGTTTCCCAGGTGGTGATATTCAGTGGGCAAGATTCAGGAA

TGATGTAAATGAATATCAAAGCGTTCAAGAAATGGAATTTGGAAAAAGTATCAATAACTTTCGCTCTAAA

ATGACTTTTGGAACCTTACGAATCAAGAGCGAAGGGCTTCGAGTTCCCCACTGGCATTTTAATGCTAATG

AACATGGCTACCTGTTAAAGGGTACTGCCTGGATTGGAGTAATTGATGCAGATGACAGCGTGGTTACCAC

ATACAATGTCACAGCTGGCCAAGTGATCTTCTTCCCTAGAAACACTTTGCATTGGATAAAGAATGTAGGA

AAAGAAGAGTGTATGTTCTTGCTGTTTTTTACAACACATGAAGAACTTAAGACCTTGGATGTAGATGATG

CATTTTTCTCTACCCCAGAAGATATAGCAGCGAGAGCATTAAAGCCACAGGGTGGAGTTAACTTTATCAG

AACATTCAAGAAACAAACAGAAGATCAAGCCATTAACCTCCCACCAAACTTAAAAGAGCTCGTACAGAAT

GCCAGCTATGTGCAATCTCCAGATAGCCTTGTATGGCGGTACTTCTACGACCTCAAAGGGTCAGCAGAAT

ACCCTTTTCCGGGAGGAATCTTCCAGTGGGCTCGCTACCGCATAAATGGAACTGGACTGAACAAGACAGA

GAAAATTTTTAGTGAGTCACTGAATAAGCATGAAAATACCCTTACCTTAGCAACTCTCAGAATATTCAGC

AATGGACTGGGGCAGCCTCATTTCCACTTCAATGCTAATGAGATGGGTTACGTCATTAGTGGCTGTGGAA

AGGTTGGGGTTATTGCATCTGATGTCACAGCCAACTTCAACATTGACATTGGAGATGTCATATTTTTCCC

TGTTGGAAGCCAACATTATATCAAGAGCGTATGTGATGAGGATTTATTTTTGATTCTAGCCTACAGTACA

GGCAACCAGCTGGAAACTCTTCGTATGAATGACTACTTCCATGCAACAGCAGATCATATCCTTGCTCAGC

TTTTTTTCAAGGAACAGAATGAGTTTAAAAAGATCCTACAGCCTTCTAAATAA

>African ostrich ELAC1

ATGTCCGTGGATATAACTTTCCTTGGGACAGGCTCCGCGTACCCCTCTCCAACGAGGGGCGCGTCAGCGT

TAGTGCTTCGCAGAGAAGGGGAGTGCTGGCTCTTTGACTGCGGAGAGGGAACTCAGACACAGTTTATGAA

AAGCCATCTGAAAGCAGGTAGAATTACAAAGATTTTCATAACTCATCTTCATGGTGACCATTTTTTTGGA

CTTCCTGGCCTGCTGTGTACGCTGAGCCTCCAAAGTAGCCCTGACCCAAACAAACCACCCATTGATATTT

ATGGACCATTAGGACTGCAAAACTTCATATGGATGACTATGGAGCTCTCCCACTCGCAGCTTCTGTTTCC

TTACGTTGTTCACGAACTTGTACCTACACGGGACCAGTGTCCTGCAGAAGAGTTTAAAGAACTTCCTTGT

CTGACCAGAGATGAAATACCTTCAGAGGAGGTGCAACAGAAGATACTCCACCTGGATCCTGTAGAAAACT

CGTACTTGCTAGTTGATGATGAGCAGTTCGTTGTGAAAGCATTTCGTCTCTTCCACCGCATTCCTTCATT

TGGCTTTGTCGTGGAAGAGAAGCCACGGACGGGTAAACTCAATGTACAAAAACTAAAAGACCTTGGAGTT

CAGCCTGGTCCTCTATATGGAAAACTGAAGAATGGTATTACAGTTGTTCTAGAAAATGGAATAACTATTT

CTCCTTCGGATGTCGTAGAAGACCCTATTCCTGGAAGAAAAATTTGCATTTTAGGGGACTGCTCGGGGGT

GGTTGGAGATGCAGCCATGAAGCTTTGCTATGAAGCAGATATATTGATTCATGAAGCCACGTTGGATGAT

AGCCAAGAGGAAAAGGCCAGAGAGCATGGTCATAGCACTCCAAAAATGGCATCTGATTTTGCAAAATTGT

GTAAAGTTAAGAAACTCGTTTTGACTCACTTCAGTCAACGATATAAACCAACTGCTCAAATAGGTGAAGG

AGATACTGACATAACAGAACTGAAGAGACAGGCAGAGTCAGTGTTAGATGGTCAAGAAGTAACACTAGCT

GAGGATTTTATGACAATAGAAGTTCCAATGAAAAAGCAAAAATAG

>African ostrich FECH

ATGTCAGGGTCCCGCAGCACAGCTCCGGCAGAAGTTGTACAAAGCAGCAGTCAAATGAGGGTCCCAGTGC

GATGGAGAAGTCAGGCAGCTGCAGCTGCAGCCACAGCAACAAAAAGCACAAAACCTCAAATTCAGCCAGA

AGCGCGGAAACCTAAAACGGGAATCTTGATGTTAAACATGGGAGGTCCAGAAAGGCTGGATGATGTGCAT

GATTTCTTACTCCGTCTCTTCTTGGACAGAGATCTAATGACGCTTCCAGCACAAAATAAATTAGCACCAT

TCATTGCTAAACGCCGCACCCCGAGAATCCAGGAACAATACAGCAGGATTGGAGGCGGATCACCAATCAA

AAAGTGGACGGCGGTGCAGGGAGAAGGCATGGTGAAACTGTTGGACAGAATGTCTCCTCACACTGCACCT

CACAAATACTACATTGGTTTCCGGTATGTCCACCCTCTGACAGAAGAGGCAATTGAAGAGATGGAGAAAG

ATGGGATTGAAAGGGCTGTTGCCTTCACGCAGTATCCACAATACAGCTGCTCTACCACAGGAAGCAGTTT

AAATGCTATTTATCGCTACTATAATAAAAAGGGGGAGAAGCCAAAGATGAAGTGGAGTATAATTGACCGA

TGGCCCACACATCCCCTTCTTATTCAGTGCTTTGCTGAACATGTACAGAAGGAACTGGATTTGTTTCCAC

CTGACAAAAGAAAAGATGTTGTTATCCTTTTCTCGGCTCACTCGCTCCCAATGTCTGTAGTGAACCGCGG

TGATCCATATCCTCAAGAAGTGGGAGCTACTGTCCAAGGAGTCATGGAGAGGCTGAACTATTCCAATCCT

TACCGGCTTGTGTGGCAGTCCAAGGTTGGGCCAATGCCTTGGCTTGGTCCACAGACAGATGAAACCATTA

AAGGACTGTGTCAAAGAGGAAAGAAGAACATGTTGTTAGTCCCGATAGCTTTTACAAGTGATCACATTGA

AACACTTTATGAGCTGGATATTGAGTATGCCCAAGTTTTAGCAAATGAGTGTGGAGTTGAGAACATCAGA

AGAGCAGAATCTCTTAATGGAAATCCTCTGTTCTCCAAGGCTCTGGCAGACTTGGTCTATTCACATATCC

AGTCGAATGAAATCTGCTCTAAGCAGTTAACCCTCTGCTGTCCGCTCTGTGTAAATCCTGTCTGCAGGGA

GACAAAAGCCTTCTTCACTAATCAACAGCTGTGA

>African ostrich LIPG

ATGGCAGCCGGGGCCGCGGAGGCAACGCTGCTGGGAGGAGATGATGCTCTGGCCGCGCTGCTGGAGGAAG

GCAAAGAGCAGGCGCCGGCTCCCAAGCGGCAGGTGAAGTTCACCCTCCGCTCCTCGCCCGACTCCGAGGA

CGAAGGCTGCGCCCTCGCCGTAGGCCACGACCGCTGTTTAGAGGAGTGCAACTTCAACGCCACGGCTAAA

ACCTTCTTCATTATTCACGGCTGGACGATGAGCGGCATGTTCGAAACCTGGCTGGGCAGCCTGGTCTCAG

CCCTTCAGGAGAGAGAGAAGGATGCGAACGTGGTGGTGGTGGACTGGCTGGCGCTCGCCCACCAGCTCTA

TACGGATGCTGTGAACAACACGGAGGTCGTTGGGAAAAGCATAGCCAGGCTGCTGAACTGGTTACAGGAG

AACCCTCTCTTCCAGCTTCAAAATGTTCACTTGATTGGATACAGCCTTGGGGCCCATGTTGCTGGCTTTG

CTGGTAACCATGTCCAGGGGACAATAGGCAGAATTACAGGCTTGGATCCGGCTGGCCCTATGTTTGAAGG

AGTGGACCCTAGCAAGCGCCTCTCCCCCGATGATGCTAGCTTTGTGGATGTCCTGCACACCTACACCAGG

GAAACGCTAGGCATTAGCATTGGGATCCAGATGCCTGTAGGCCATGTTGACATCTACCCCAATGGGGGAG

ACTTCCAGCCTGGCTGTGGACTAAGTGATGTCTTAGGAGCAATTGCCTATGGGACTATTGGTGACGTTGT

TAAATGTGAGCACGAGCGGTCTGTGCACCTCTTTGTGGACTCCCTTGTGAACCAAGATAAACAAAGCTTC

GCGTTTCAATGTACTGATTCCAGTCGCTTCAAGAAGGGAATCTGCCTGAGCTGCCGGAAGAACCGCTGCA

ATGGCATAGGCTACAATGCCAGGAAAACTAGGAACAAAAGAAACAGCAAGATGTACTTAAAAACAAGAGC

TGACATGCCATTCAAAGTCTACCATTATCAGATGAAAATGCACGTCTTCAGCTACAAGAGCTTGGGAGAG

GCTGACCCCACCTTCTCCATCACCCTTCATGGCACCAATGGAGACTCTGAACCCTTCTCTTTAGAAATGC

TTGAGCGAATTGGCCTTAATGCCACCAACACCTTCCTGGTCTATACTGAAAAGGACATGGGTGACCTCTT

AAAAATAAAGCTCACCTGGGAGGGAACTTCGCAGTCATGGTACCATCTATGGAAAGAGTTTAAGAGTTAC

TGGTATCAGCCTTCAAAGTCGCCCCAGGAACTGCACATCAGACGTATACGGGTGAAATCTGGGGAAACGC

AGCAGAGGTTTGCTTTCTGTGTGGAAGATTCCCAGCTGACCAGCATATCTCCTGGTAAAGAGCTCTGGTT

TGTGAAATGTGCAGACAACTGGCAAAAAAGGTCTGTATCAAATTTACTCTGA

>African ostrich MAPK4

ATGGCAGAGAAGTGCGACTGTATTGCCAGCATGTACGGGTACGATCTAGGCTGCCGCTTCATTAATTTCC

GGCCCTTGGGCTTTGGTGCGAATGGACTGGTGCTGTCAGCCCTTGACAGCAAGAGCTGCCGCAAAGTTGC

CGTGAAGAAGATCACCATCAGTGATGCACAGAGTATGAAACACGCCTTCCGGGAGATCAAAATCATCCGC

AGGCTGGACCACGACAACATTGTGAAGGTGTATGAGGTGCTGGGACCGAAGGGGACCAACCTGCACGGAG

ACTTTTTCAAGTTTAACATGGTGTACATCGTTCAGGAGTACATGGAGACAGACCTGGCTCGGCTACTGGA

GCAGGGGAAGCTCACCGAGGAGCATGCCAAGCTCTTCATGTACCAGCTGCTCCGAGGGCTGAAGTACATC

CATTCGGCTAACGTGCTCCACAGAGACCTGAAGCCGGCCAATATTTTCATCAGCACGGAGGACCTGGTGT

TGAAGATTGGTGATTTTGGGCTAGCCAGAATTGTGGATCAACATTACTCGCATAAGGGTTACCTCTCCGA

AGGCTTAGTAACAAAGTGGTATCGCTCCCCTCGCCTCCTCCTCTCGCCGAACAACTACACCAAAGCCATC

GACATGTGGGCGGCTGGCTGCATTCTGGCCGAGATGCTCACGGGAAGGATGCTCTTCGCCGGGGGTCATG

AGCTGGAACAGATGCAACTTATTCTGGAGACGATCCCTGTGATCCACGAGGAAGACAAAGAGGAGCTGCT

CAAAGTGATGCCCAGTTTCATCAACAGTACCTGGGAAGTGAGGAAGCCCCTGCGCAAGCTGCTCCCCGAA

GTGAACAGTGAAGCTATTGATTTTCTGGAGAAAATACTGACATTTAACCCTATGGATCGATTAACGGCTG

AGATGGGTCTGCAGCATCCTTATATGAGTCCGTATTCTTGCCCTGAGGATGAACCAGTGTCTCAGCATCC

GTTCCGGATTGAGGATGAGATTGATGATATTTTACTGATGGAAGCCAACCAGAGCCAGATGTCCAACTGG

GACAGGTATCAAGTAAGCCTCTCCTCTGATTTGGAATGGAGACATGATAAATATCACGACATGGATGAGG

TCCAGCGAGACCCACGGGCAGGGTCTGAATCCATTGCTGAAGAAGCACAAGTTGATCCACGAAAATATTC

ACAGAGCAGCTCAGAGAGGTTCTTGGAGCTATCACACTCATCGATGGACCGGGTATTTGATGTTGATTGT

GGGAAATCATGTGATTACAAAGTGGGGTCACCTTCCTACTTGGACAAATTGCTGTGGAGAGACAATAAGC

CCCATCATTACTCAGAGCCCAAGCTGATTTTAGATTTATCGCACTGGAAAAGAGCAACCATAGCACCCAC

AGCTGAGCTATCTCTGGAAGAAGAACCATCCAATCTCTTTCTGGAGATTGCTCAATGGGTGAAGAGCACG

CAGGTGGGTCTTGAGTGTCCCAGTCCTCTTCCAGAGATTCAAGAACGGAGCCTGCCGTCTTCTCCTCACC

ATCTCCACAAGGAATCCAAGGAAGTAGGCAGTGAAACAGACCCTGAGTTTGACTTGGATGTCTTCATCTC

CAGGGCACTGAAACTTTGCACAAAACCTGAGGATCTTCCAGACAACAAGCTCAATGACATCAACGGAGCC

TGCATAGCTGAGCACCCCAATGAGATCGTACAAACAGAAGTGTACCAGAAGGAACGATGGTGA

>African ostrich MBD2

ATGGAGAAGCAGGGGAGGATGGACTGCCCCGCGTTGCCCCCGGGCTGGAAGAAGGAAGAGGTGATCCGCA

AGTCGGGCCTCAGCGCCGGCAAGAGCGATGTCTACTACTTCAGTCCAAGTGGTAAGAAGTTCAGAAGCAA

GCCTCAGTTGGCAAGATACCTGGGAAACACTGTTGATCTCAGCAGTTTTGACTTCAGAACGGGAAAGATG

ATGCCCAGTAAATTGCAGAAGAACAAACAGAGACTAAGGAATGATTCTCTCAATCAAAATAAGGGAAAAC

CAGACTTAAATACAACTTTGCCAATCAGACAAACAGCATCGATTTTCAAACAACCAGTCACCAAAGTTAC

CAATCATCCTAGTAACAAAGTGAGATCTGATCCACAGCGAGTAACAGAGCAGCCACGGCAGCTTTTCTGG

GAGAAGAGGCTACAAGGCCTCAGTGCATCTGATGTCAGTGAACAAATCATAAAATCCATGGAGCTACCTA

AGGGTCTTCAAGGAGTCGGCCCAGGTAACAACGACGATACCCTGTTATCAGCTGTTGCTAGTGCTTTGCA

CACCAGTTCTGCGCCTATCACGGGACAGCTCTCTGCAGCTGTAGAAAAGAACCCAGCTGTTTGGCTTAAT

ACATCTCAACCCCTCTGCAAAGCTTTCATAGTTACAGATGATGACATTAGAAAACAAGAAGAGCGGGTGC

AACAAGTGCGTAAAAAACTGGAGGAAGCACTAATGGCAGACATCTTGTCACGAGCAGCTGATACGACAAA

AGATATAGATGTAGAAATGGATAATGGAGATGAAGCATAA

>African ostrich ME2

ATGTTCTCCCGATTAAGAGTAGCTGCCACTCCCTGTGCCATGGCATGTCGCAGTGCGCATACGAAAGAGA

AAGGCAAGCCACTGATGTTAAACCCACGAACAAACAAGGGCATGGCCTTTACGTTGCACGAACGACAGAT

GCTTGGGTTGCAAGGACTTCTGCCTCCTAAAATAGAGACACAAGACATTCAAGCCTTACGCTTCCATAAG

AATTTGGCAAAAATGAACGACCCCTTGGAAAAGTATATCTACATAATGGGAATCCAAGAGAGAAATGAAA

AATTATTCTATAGGGTATTACAAGATGATATTGAGCGGTTAATGCCAATTGTATATACACCAACAGTAGG

CCTTGCCTGCTCCCAATATGGACACATCTTCAGGAGACCAAAAGGATTATTTATTTCTATCTCAGACAGA

GGTCATATAAGATCAATTGTGAACAACTGGCCAGAGAATGACGTTAAGGCTGTTGTTGTCACTGATGGAG

AAAGAATATTGGGTCTTGGAGACCTAGGTGTCTATGGAATGGGAATTCCAGTAGGAAAACTGTGTTTGTA

TACGGCCTGTGCTGGCATACATCCAGATAAATGCTTGCCCGTGTGTATCGACGTTGGAACTGACAATACA

ACACTCTTGAAAGATCCGTTTTATATGGGCCTGTATCAAAAACGGGATCGCTCACAAGTCTATGATGACC

TAATTGATGAGTTTATGGAAGCCATTACAGACAGGTATGGCCAGAACACGCTTATCCAGTTTGAAGACTT

TGGAAATCACAATGCTTTTCGTTTTTTGAGAAAATACAGAGAGAAATATTGTACCTTCAATGATGATATT

CAAGGAACAGCTTCAGTGGCCTTAGCAGGACTGTTGGCAGCACAGAGAGCCACTGGCAAACCAGTTGCAG

AGCAGAAAGTGCTGTTCCTTGGAGCAGGAGAGGCTGCCCTGGGAATTGCAAACCTCATTGTTATGGCTAT

GATGGAAAGCGGTGTTTCTGCGGAGGAAGCCTACAAGAAAATATGGATGTTTGACAAATATGGTTTACTG

ATTCAGGGCCGAGAACACATGGTAGATTCCAATCAAGAACCATTTACGCACCATGCTCCAGAGCTGATAC

CAAAGACATTTGTAGAGGCAGTGAACGTACTTCGGCCTTCAGCTATCATTGGAGTTGCAGGAGCTGGGCG

GCTTTTCTCACATGAGGTGATCAAAGCAATGGGCTCTATCAATGAGCGACCCATCATATTTGCACTAAGT

AACCCCACAGTGAAAGCTGAATGCACAGCAGAGGAAGCATATACGTTAACAGAGGGACGATGCTTGTTTG

CCAGTGGCAGTCCCTTCGACCTGGTGACCTTGAAAGATGGACGAATCTTCAAACCGGGCCAGGGGAACAA

TGCTTACATCTTTCCAGGCGTGGCTCTCGCTGTGATCCTCAGCAGTGTTCGACATATTAGTGATAAGGTT

TTCCTAGAGGCTGCTAAGGCTTTGACAGAACAGTTGACTGATGAAGAACTTGCGCAAGGAAGACTTTATC

CTCCACTGTCTAATATCAGGGAAGTTTCTATTTATATTGCCGTCAAGGTTATGGAATTTTTGTACGCGAA

CAACATGGCTTTCCACTATCCTGAACCTGCAGACAAGAACCATTACATTCGATCAAAAGTTTGGACCTAC

GAATATGAATCCTTCATGCCAGATGTGTATGACTGGCCTGAGTCTAAAGTACACTGA

>African ostrich MEX3C

ATGGCCGTCAACAGAAACCGAACGAGAGTGCGTCTGTGCTCTCCTGGGCTCACGTTAGCTCTCTTTGGCT

GGAATCTGATCGGGCCTAACCTGTTGCCTTCCCTGGACCCCGCCGAACTGCACCGCCGGCCTCCTGGGGG

AGGTTGCAAAATAAAAGCACTAAGGGCCAAGACAAATACGTACATTAAGACCCCCGTTCGTGGAGAAGAA

CCCATCTTTGTTGTCACTGGACGAAAAGAGGATGTAGCCATGGCCAAAAGGGAAATTCTCTCAGCTGCTG

AACACTTCTCCATGATCAGAGCATCACGCAACAAAAACGGTCCTGCCCTGGGAGGGTTGCCATGTACTCC

CAACCTACCAGGTCAGACGACAGTCCAAGTCAGGGTGCCTTACCGTGTAGTTGGGCTAGTGGTTGGACCT

AAAGGAGCTACAATCAAAAGAATTCAGCAGCAGACACACACCTACATAGTCACTCCCAGCAGGGACAAGG

AGCCCGTCTTTGAAGTTACCGGAATGCCTGAAAACGTTGATCGCGCCCGGGAGGAAATTGAGATGCACAT

AGCCATGCGTACTGGAAACTACATTGAGCTGAACGAAGAGAATGATTTCCATTACAACGGTACAGATGTG

AGCTTTGAAGGAGGCACTCTGGGATCTGCTTGGCTTACTTCTAATCCTGTCCCTCCTAGCCGCACCAGAA

TGATTTCTAATTACAGAAATGATAGCTCCAGCTCCTTGGGAAGTGGCTCCACAGATTCCTATTTTGGAAG

CAATAGATTGGCTGACTTCAGCCCAACAAGTCCCTTCAGCACAGGCAACTTTTGGTTTGGAGAAACGCTC

CCTTCAGTGGGCACAGAAGATCTCGCAGTCGACTCTCCTGCTTATGACTCCTTACCAACACCTTCCCAAA

CCATTTGGACCCCTTTTGAACCTGTAAACCCGCTCTCTGGCTTTGGCGGCGACCCTACTAGTAATATCAA

GCCTCAGCGAAGAGGGAGTCAACCATCTACTCCTCGTCTGTCACCAACGTTTCCGGAAAGTCTGGAACAT

CCGCTTGCTAGGAGAGTAAGAAGTGACCCACCTAGTGCAGGCAACCAAGCTGGCCTTCCAATATACATCC

CTGCTTTTTCTAATGGTACCAACAGCTATTCCTCTTCCAATGGTGGCTCCACCTCCAGTTCGCCTCCCGA

GTCGCGACGAAAGCACGACTGCGTGATCTGCTTCGAGAACGAAGTCATTGCGGCCCTAGTCCCGTGTGGT

CACAATCTCTTCTGCATGGAATGTGCCAACAAAATCTGTGAAAAAGAAACGCCATCATGTCCCGTTTGCC

AGACAGCTGTTACTCAGGCAATCCAAATTCACTCTTAA

>African ostrich MYO5B

ATGCCTGCGCCAGGCTCGAGGCGGGAGGATTTGCGGCGCTCGAGCGAAGGCCAAAAACTTTCCATCTCGG

AGGCAGCCTCGTGCCGGGTACGCTCCGATCCCGAGCTATTTCGGGCGTTCCTGCCACGCGTCTCAGCTCT

CTCCTTGCCGAGAGGTGCCCGGCACTGGACGCAGTCCCGGCACCGCGGGAAACTTTGTGCGCGAAGAGGG

AAAGAAAGGGAAGGTGCTGTGCAGGGGGGGGGCGGCGAGCCACCGCCACCATCCAGAAAGGCTGTTTCCT

ACACTAGGGTTTGGATTCCTGACCCTGATGAAGTTTGGAAATCGGCAGAAATAATCAAGGATTACAAGGA

GGGAGATAAAAGCCTGCGTCTGAGACTCGAAGATGAAACTATCTGCGAGTATCCCGTTGACCTCCACGGA

AACGAGCTGCCTTTCCTGCGCAATCCTGATATCCTGGTGGGAGAGAATGACCTGACGGCCCTGAGCTACC

TGCATGAGCCTGCGGTCCTCCACAACCTCAAAGTGAGGTTCCTCGAGTCCAACCACATCTACACGTACTG

TGGTATTGTGCTTGTTGCCATCAATCCCTATGAGCAGCTGCCAATCTACGAGCAAGATGTCATCTACGCC

TACAGCGGCCAAAACATGGGGGACATGGATCCTCATATCTTCGCGGTGGCAGAAGAGGCCTATAAGCAGA

TGGCCAGGGACGAGAAGAACCAGTCCATCATTGTAAGCGGCGAGTCGGGTGCTGGCAAGACGGTCTCGGC

CAAATACGCCATGCGCTTCTTTGCAACCGTCGGTGGGTCCGCCAGCGAGACCAACATTGAAGCCAAAGTC

CTCGCCTCAAGCCCAATTATGGAGGCAATTGGAAACGCTAAAACAACAAGGAATGACAACAGCAGCCGCT

TTGGGAAATATATTCAGATCGGCTTTGATAAAAGATACCATATCATTGGTGCCAACATGAGGACCTATCT

GTTGGAAAAATCAAGAGTGGTGTTCCAGGCGGAGGACGAGAGGAACTACCACATCTTCTATCAGCTTTGT

GCCTCAGCGAGTCTTCCAGAATTTAAAGACCTTGCACTAACATGTGCCGAAGACTTCTTCTATGCTTCCC

AGGGAGGCAACGTGTCCATCGAGGGCGTGGATGATGCTGATGACTTTGAGAAGACCAGGCATGCCTTCAC

CCTGCTCGGAGTGAAGGAGTCTCACCAGATGACCATTTTCAGGATAATCGCTGCCATTCTGCACCTAGGC

AACTTGGACATCCAAGCCGAACGGGATGGCGATGCCTGTAGCGTCTCGAGTACAGACGAGCACCTGAACA

CCTTCTGCAGCTTGCTGGGCGTGGAGCACAGCCAGATGCAGCACTGGCTCTGCCACCGGAAGCTGGTCAC

CACGGCCGAGACCTACGTGAAGAGCATGTCCCTTCAGCAGGTGCTCAACGCCAGGGATGCCCTGGCTAAG

CACATCTATGCCCAGCTCTTCAACTGGATCGTGCAGCACATCAACAAAGCCCTGTACACCACTGTGAAGC

AGCACTCCTTCATCGGCGTGCTCGATATCTATGGGTTTGAAACTTTTGAAGTGAATAGCTTTGAGCAGTT

TTGTATCAACTACGCCAACGAAAAGCTCCAGCAGCAATTCAACTCGCACGTGTTTAAGCTGGAACAAGAA

GAGTACGTGAAGGAAGAAATCCCGTGGACTCTTATAGACTTTTATGATAACCAGCCCTGCATAGACCTTA

TAGAAGCGAAACTTGGTATCTTGGACCTGCTGGATGAAGAGTGCAAGGTTCCCAAAGGAACCGACCACAA

CTGGGCACAGAAGCTGTACGACCGGCACGCCAGCAGCCAGCACTTCCAGAAGCCCCGCATGTCCAACACC

TCCTTCATCGTCGTGCACTTTGCAGACAAGGTGGAGTACCAGTGCGAGGGGTTTCTCGAGAAAAACAGGG

ACACCGTGTATGAAGAGCAGATCAACATCCTGAAAGCCAGCAAGTACCAGATGGTAGCAGACTTGTTCCA

AGATGAGAAGGATTCGGCACCCGCTGCCCCTATGGGGAAGGGGACCTCCAAGATCAACATCCGTTCTGCT

AGGCCGGTTGTCAAAGCGGCCAACAAGGAGCACAAGAAGACGGTGGGGCACCAGTTCAGGAACTCCTTGC

ACTTGCTTATGGAGACGCTGAATGCCACCACCCCGCACTACGTACGCTGCATCAAGCCAAATGACGAGAA

ACTCCCGTTCAAATTTGATCCAAAGAGAGCAGTCCAGCAGCTCAGAGCTTGTGGTGTGTTGGAGACCATC

CGCATCAGTGCAGCTGGCTTCCCGTCCAGGTGGTCCTACCACGACTTCTTCAATAGGTACCGTGTTCTTA

TGAAGAAGAGAGACATCTCCAAGAATGACAAGAAGCAGATCTGCCGGACCCTGTTGGAAGACCTCATTAA

GGATCCAGACAAGTTCCAGTTTGGACGTACCAAGATCTTTTTCCGTGCAGGCCAGGTGGCATATCTGGAG

AAACTGCGAGCAGATAAGTTCAGAGCTGCCACAATCATGATTCAGAAGACAGTGCGGGGCTGGCTGCAGA

GGGTGAAGTACCAAAGGCTGAAGAGGGCTGCGATAGTAATCCAGCGTTACGCCCGTGGGCACCTGGCTCG

CAGGCTTGCAGAGCACCTGCGGAGAACGCGAGCGGCCACCGTCTTCCAGAAGCAATATCGAATGCTAAGG

ATCCGCCGGGCTTTCCAGCGGGTCCGCAACGCCACCGTCACCATCCAGGCTTTTGCTCGGGGCATGTTTG

TCAGGAGAGTTTATCGCAAGATGCTTGTAGAGCACAAAGCAACCATCATCCAGAAGTACCTTCGCACCTG

GCTGGCCCGTACTCGCTTCCTCCGCATCAGGAGTGCCACCATTGTCCTGCAGTGCTACTACCGGCGCATG

AAGGCCCGACAGGAGCTGAAGGCCCTGAAGATCGAGGCCCGCTCAGCGCAGCACCTGAAGAAACTCAACA

TTGGCATGGAGAACAAGGTTGTCCAGCTTCAAAGGAAGATTGATGAGCAGAGCAAGGAGTACAAACTTCT

GAATGAGCAACTCTCCGCGCTCACCTCTGCCCATACGTCTGAAGTGGAAAAGCTGAAGAAGGAACTGGAG

CAATATCAGCAGTGCCATCGGGGTGATGGGAACCAACTCGTTAGCTTGCAAGAAGAGACGGAAAGCCTCC

GCCTGGAGCTTGAGAAAGCCCATGGTGAGAGGAAGGTGGTGGAAGACAGCTACGTGAAGGAGAAGGACCT

GCTCAAAAAGCGCATATCGGACCTGGAGGAGGAGAACGCGCTCCTGAAGCGGGAGAAAGAGGAGCTTAAC

CACAAGATCCTGTGTCAATCCCAAGATGGATTTGCACAAAATACGGTTGAGGAAAATCTCCAGATGAAGA

AAGAGCTGGAAGAAGAGCGATCTCGTTACCAGAACCTGGTAAAAGAATATTCGAGGCTGGAGCAGAGATA

CGACAATCTGAGGGATGAAATTACTATTATAAAGCAAGCTCCGGGGCACAGGAGAAACCCGTCCAACCAA

AGCAGTTTGGAGTCCGACTCCAATTACCCGTCGATATCGACCTCCGAGCTAGGGGACACTGAGGATGCAA

TTCAGCAAGTGGAGGAGGCTGGGATGGAGGAAGCAGCGAAGCGAGTGAGAGAGCTCGAGCAGGAGAGGAA

GAAACTGCAAACCCAGCTGGAGAAAAAGGAGCAGGAAGGCAAGAAAGCCCAGGTAATTGAAACAAAGACT

GAAATGGCTTCAGACAGTGAAGATTTTGCATACAACAGCCTGAAGAGGCAAGAACTGGAATCGGAGAACA

AGAAACTGAAAAACGAACTGAACGAACTGAGGAAGGCTATTGCAGAGCGGGCGACCCAGAACAATTCCTC

CAATGACATCCAGGACAGTTACAATCTCTTGCTGAACCAGCTGAAATCGGCCAATGAAGAGCTGGAAGTG

CGGAAAGAAGAGGTTCTTATCCTGAGGACGCAGATTATGAACGCGGCCCAGCAAAAGGAGACAGGCAAAA

ATATGGAAACAAACATCAACACCCATGCCAGCTGGCCCAACAGCGACAAGCATGTGGATCAAGAGGATGC

GATTGAGGCTTACCAAGGGATGTGCGAGACGAACCGCAAGACTGAGGACTGGGGTTATCTCAATGAAGAT

GGAGAGCTCGGCTTGGCTTATCAAGGCTTAAAGCAAGTTGCCAGGTTGCTGGAAGCGCAGCTCCAGGACC

AGCGAAGAGAGCATGAGGAGGAGGTCGAAACTCTGAAAAACCAAGTAGAGGCAATGAAAGAAGAGATGGA

GAAACAGCAGCAGGCCTTCTTGCAGACCCTGCAGCTGTCTCCAGAGGCCCAGGTGGAGTTTGGTCTTCAG

CAAGAAATTACGCGCCTCACCAATGAAAATCTGGATCTCAAAGAATTGCTGGAAAAGTTGGAAAAGAATG

AAAAGAAGCTGAAGAAGCAGCTAAAGATTTACATGAAGAAGGTCCAAGATTTTGAAGCGTCCCAAGTGAC

GGTACAGACGGAGAGGAGGCGGCACGAGCTCAACCGGCAGGTTGCCGTCCAGAGGAAGGAGAAAGATTTC

CAGGGAATGCTGGAATATTACAAAGAGGACGAGCCACTTCTTGTGCGAAACCTCATCACAGATCTGAAGC

CCCAGACGGTGTCTGCCACCGTTCCTTGCCTCCCTGCCTACATCCTCTACATGTGCATCAGGCACGCGGA

CTACGTCAACGATGACCAGAAAGTGCACTCCTTGCTCACCTCCACCATCAACGGCATCAAGAAAGTATTG

AAGAAACACAACGAGGACTTTCAGATGACGTCGTTTTGGCTGGCTAACACATGTCGCCTCCTGCACTGCT

TAAAGCAATATAGCGGAGATGCGGGTTTCATGACACAAAACACCGCTAAGCAGAATGAGCACTGTCTCAA

AAACTTCGACCTGACCGAGTACCGCCAGGTGCTGAGCGATCTCTCCATCCAGATCTATCAGCAGCTCATT

AAGATAGCAGAGGGCATACTGCAGCCCATGATAGTGTCTGCAATGCTGGAAAATGAGAGCATTCAGGGGC

TTTCCGGCGTGAAACCAATGGGCTACAGGAATCGCTCCTCCAGCATGGCAGACAGTGACAGCTCGTACAG

CTTGGACGCGGTCATCCGCCAGATGAATGCGTTCTACAGCATCATGTGCGATCAGGGCCTGGACCCAGAG

ATCATCCAGCAGGTCTTCAAGCAGCTCTTCTACATGATCAATGCAGTCACCCTCAACAACCTCCTGCTGC

GGAAGGACGTCTGCTCGTGGAGTACTGGCATGCAGCTAAGGTTTAACATAAGCCAGCTAGAGGAGTGGCT

GCGCGGGAAGAACTTGCAGCAGAGCGGAGCAGCGCAGACGTTGGAGCCCTTGATTCAGGCAGCGCAACTC

CTGCAGCTGAAAAAGAAGACCTCAGAGGACGCTGAGGCCATCTGCTCCTTGTGTACCTCACTCACTACCC

AGCAGATTGTAAAAATACTTAATCTCTACACTCCTGTGAACGAGTTTGAAGAGCGTGTTACAGTAGCTTT

CATACGAAACATACAGGCCCACTTGCAAGAGCGAAATGACCCTCCGCAGCTGCTGTTAGACTTCAAGTAT

ATGTTCCCAGTTTTGTTTCCATTCAACCCATCCTCCATAACCATGGACTCTATTCATCTCCCTGCTTCTC

TCAACTTGGAATTTCTCAATAAAGTCTGA

>African ostrich NARS

ATGTCGCACCTCTTGTTCAACAAGACTCACCGTGAAAAGAGAACCCTATCTGCACTCACTCCCGACTGCA

GCGATCAAGTCGAACTCTGGGTGCCCACAGCTAATTTGTTTGCATCCATAGTCCCCGGGGTGGGTACTGC

TCATGCACTCCGTACTATCAGCAAACTCGCTTGCTGGATGGTGAAACAATCCAACGTGACGACTCAGGTC

CTTAGTGAATTGGCACAGGATATGGAGAACCTGCAACATCAGGTCCTCCAGAATAGAACTGCCATTGATT

TTTTGCTATTGGCACACGGCCATGGTTGTACGGAGTTCGAAGTTTCCTACCTTCGCAAGCTGTTTTTGCG

CTTACTTGCTAAAGTACAGATTAACTATGCCCTACTACAAGAAAGCGGGGGACGTGTGGAAGGTATTTTG

GGAAGTCGGCTACGTGACGAGAGACATGAAGACACGGCTGCAAGAACTGCTTGGATCCGTCTGTATTTGT

CCTGTGTTACAGAAGAGCTGTATGTCTCTGAACGAGAGGGCAGTGATTCCGCTGGTGATGGGACACAAAA

GAAACCATTCAAAACTGTTTTAAAGGCTTTAATGACAGCAGGAAAGGAACCGTTTCCTACTATTTATGTG

GATTCGCAAAAAGAAAATGAGAGATGGAGCATTATTTCAAAGTCACAGATGAAGAACACCAAAAAACTGT

GGCAGAAGGAACAAATGAAGAATGAAGCTAAGGAGAAGAAAGAGGTAGAAGATCTCTTGAGAAGGGAGAA

GAACCTAGAGGAAGCTAAGAAAGTTATTATCAAGAATGATCCTAGTCTTCCAGAGCCAAAATGTGTAAAG

ATTGGTGCTCTGGAGGCTTACAGAGGCCAAAGAGTGAAGATTTTTGGTTGGATTCACAGATTACGTAGGC

AAGGAAAAAATTTGATGTTTGTTGTTTTGAGAGACGGCACAGGATTTCTACAGTGTGTCCTTTCAGATGA

ACTGTGTCAGTGTTACAATGGGCTACTTCTCTCTACAGAGAGCAGTGTTGCAGTGTATGGGATGCTGAAC

CTTGTTCCTGAAGGCAAGCAGGCTCCAGGAGGCCATGAGCTGAACTGTGACTACTGGGAGCTTATCGGTC

TAGCCCCAGCAGGAGGAGCTGACAATCTGCTCAATGAGGATTCGGAGGTTGATGTGCAGCTTAACAACAG

GCATATGATGATTCGAGGCGAGAATATGTCCAAAATCTTCAAGGTGCGCTCCATGGTAGTACAAGCCTTC

AGGGATCACTTCTTTGCCAATGGATATTATGAAGTGACACCACCAACGTTAGTCCAAACACAGGTTGAAG

GAGGCTCAACCCTATTCAAGCTGGATTATTTTGGTGAAGAGGCATTCTTGACGCAATCATCCCAGCTCTA

CCTGGAGACCTGCCTTCCAGCATTAGGAGATGTTTTTTGTATTGCTCAGTCATACAGAGCTGAGCAATCC

AGGACACGCAGACACTTGGCAGAATACACTCACATTGAAGCTGAATGTCCTTTTATAAGTTTTGAGGACC

TGCTGGACCGTCTGGAGAACTTGGTTTGTGATGTAGTGGACAGAGTTCTGAAATCACCTGCAGCAAGCTT

ACTGTTTGACATAAACCCGGGCTTCCAGCCCCCTAAACGTCCTTTCCGACGAATGAACTATACTGATGCC

ATCGCATGGTTAAAGGAACATGATGTGAAGAAGGACGATGGCACTTACTATGAGTTTGGAGAGGATATTC

CTGAAGCTCCTGAGAGATTAATGACGGACACCATTAATGAGCCAATCCTGTTGTGTCGATTTCCTGCAGA

GATAAAGTCCTTTTATATGCTGCGTTGTTCTGAGGATTCCCGGCTTACGGAATCTGTTGATGTGTTGATG

CCTAACGTTGGTGAGATTGTTGGAGGCTCTATGCGTATCTGGGACAGTGATGAGCTAATAGAAGGCTATA

AGAGAGAGGGCATCGATCCCACACCGTACTACTGGTACACTGATCAGAGGAAATATGGCACCTGTCCTCA

TGGTGGATATGGTTTGGGGTTAGAACGATTCCTGACCTGGATTCTGAACAGGCACCATATCAGAGATGTC

TGTCTCTATCCACGCTTTGTCCAGCGCTGCAAACCGTAG

>African ostrich NEO1

ATGGACGCTCTGTCAGTCCGCGGCGCTTCTGTTATCATGAACTGCTCAGCCTATTGCGAAACTTCGCCAA

AAATTGAATGGAAAAAAGATGGGACTTTTCTCAACTTGGCGTCCGATGACCGGCGCCAGTTGCTACCAGA

TGGATCTTTATTAATAAACAGTGTGGTGCATTCCAAACATAATAAACCCGATGAAGGATATTATCAGTGT

GTGGCAACCGTGGATAGCCTGGGGACCATCGTAAGCAGAACAGCAAAGCTCACAGTAGCGGGTCTTCCCA

GGTTCACCAGTCAGCCAGAATCATCATCTGTCTACAGAGGAAGCAGTGCAATCCTTAACTGTGAAGTCAA

TGTTGACCTTGCACCATTCGTGAGGTGGGAGCAGGACCGTCAGCCAGTCTTTCTGGATGATCGTGTGTTT

AAATTACCAAGTGGAGCTCTAATTATTAGCAATGCTACTGATATGGATGGAGGACTCTATCGTTGCATCA

TTGAAAGTGGTGGGACCCCCAAATACAGCGAAGAGGCCGAGCTCAAAATTCTTCCAGATCCAGAGGTGTC

GCGGAACTTGATGTTTCTGAGGCAGCCAACTTCACTTACTAAAGTTACTGGGCAAAGCGCGGTTTTTCCA

TGTGTTGCGGTAGGATTTCCAACACCATACGTCAGATGGACAAGAAATGAAGAAGAGCTTATCACAGAAG

GCTATGAAAGATTTCTTTTACTTGCGGGAGGGAGCCTGGAGATCCGCGAGGTTACGGCGGGTGATGCTGG

GACGTATTCCTGCGTAGCCGACAACGGGAACGAGACGATCGAAGCTCAGGCAGAGCTTACGGTTCAAGTT

CCTCCTGAGTTTCTGAAGCGGCCTGCAAATATATATGCGCACGAATCTATGGACATTGTGTTTGAGTGTG

AGGTGACAGGAAAACCTACTCCAACGGTGAAATGGGTCAAGAATGGCGACATGGTGATCCCAAGCGACTA

CTTCAAAATTGTTAAAGAACATAATCTGCAAGTTTTGGGTCTGGTGAAATCAGATGAGGGATTCTATCAG

TGCATTGCAGAAAATGATGTTGGAAATGCACAGGCTGGAGCCCAGCTGATAATACTTGACCTTGATGTTG

CCATCCCAACATTACCTCCCACTTCACTGACCAGTGCCACTAATGACCATCTAGCACCAGCAACAACTGG

ACCATTGCCTTCAGCCCCTCGAGATGTTGTGGCCACCCTCGTCTCCACTCGCTTCATCAGACTGACGTGG

CGGACGCCTGTATCAGACCCACAAGGAGACAACCTCACCTATTCAATCTTCTACACCAAGGAAGGTATAA

GCAGGGAACGTGTCGAAAATACAAGTCGTCCGGGAGAGATGCAAGTGACGATCCAAAACCTGATGCCAGA

AACAGTTTATGTCTTTAGAGTTGTGGCTCAAAATAAACATGGCCCCGGCGAGAGCTCAGTACCCCTGAAG

GTGGCAACTCAGCCTGAGGTTCAGCTTCCTGGTCCAGCACCCAATATTCGAGCGTATGCCAGTTCACCCA

CTTCAGTTACCGTGACATGGGAAACGCCGTTGTCTGGCAATGGAGAAATCCAGAACTACAAGCTCTATTA

CATGGAGAAGGGACAGGACAATGAACAGGATGTTGATGTAGGAGGCCTCTCCTATACCATTAATGGGCTG

AAGAAATACACAGAGTATAGTTTCCGAGTGGTAGCCTACAATAAACATGGCCCTGGAGTCTCTACCCAAG

ATGTTGTTGTACGAACACTGTCAGACGTCCCCAGCGCCCCACCACAGAATCTAACCCTGGAGGTTCGGAA

TTCCAAGAGCATCATGCTACAGTGGCAGCCTCCGCCTCTAGGAACACACAGCGGACAAATCACTGGCTAC

AAAATTCGCTACCGTAAAGTGTCCCGTAAGAGCGATGTAACGGAGAGCATTGGTGGGACACAGCTGTTTC

AGCTAATTGAAGGTCTCGAACGAGGCACAGAATACAGCTTCCGCGTAGCTGCCTTGACTGTTAACGGCAC

TGGACCGGCTACTGACTGGGTATCGGCAGAAACGTTTGAGAGTGATCTAGATGAAACTCGTGTTCCTGAA

GTGCCAAGTTCCTTGCATGTCCGCCCACTTGTCACCAGTATTGTAGTAAGCTGGACTCCGCCTGAAAACC

AGAACATTGTGGTAAGAGGCTATGCTATAGGGTATGGCATTGGCAGTCCCCACGCACAGACCATCAAGGT

GGACTACAAACAGAGATATTACACCATTGAGAACTTAGACCCAAGCTCCCACTACGTCATAACTCTGAAA

GCATTTAACAACGTCGGTGAAGGAATCCCTCTCTACGAGAGCGCAGTGACTCGGCCTCATTCAGACACTT

CCGAAGTTGATTTGTTTGTTATTAATGCTCCATACACTCCAGTGCCAGATCCATCTCCCATGATGCCACC

GGTGGGAGTTCAGGCTTCCATTCTGAGTCATGACACCATCAGGATCACTTGGGCAGACAACTCTCTGCCA

AAGAACCAGAAGATCACAGATGCTCGCTACTACACAGTTCGCTGGAAAACCAATATTCCCGCAAATACAA

AGTACAAGACTGCAAACGCAACCACTTTGAGCTATTTAGTGACCGGGTTAAAACCAAATACCTTGTATGA

ATTCTCTGTGATGGTGACTAAAGGTCGAAGATCGAGTACTTGGAGTATGACAGCACACGGAACAACTTTC

GAATTAGTTCCTACTTCTCCTCCTAAAGATGTGACTGTGGTGAGCAAAGAGGGAAAACCTCGGACAATAA

TTGTTAACTGGCAGCCTCCATCTGAAGCCAATGGGAAAATTACAGGATACATCATTTACTACAGTACAGA

TGTGAATGCTGAAATACACGACTGGGTTATTGAACCCGTCGTGGGAAACAGACTGACCCATCAGATACAA

GAATTAACCCTCGATACACCCTATTATTTCAAAATTCAGGCTCGCAACTCCAAGGGCATGGGGCCTATGT

CTGAGGCAGTTCAGTTCAGAACCCCAAAAGCTGAATCCTCAGATAAAATGCCTAATGATCAAGCTTCAGG

ATCTGCAGGAAAAGGAAGCCGGCCAGTGGACATAGGGCCAGATTACAAACCGCCGCTTGGTGGCAGTAAC

AGTCCCCACGGAAGTCCTACTTCTCCCTTGGATAGCAACATGCTTCTTGTAATCATAGTATCTGTTGGAG

TGATTACCATCGTGATAGTGGTGATAGTTGCAGTCTTCTGCACTCGTCGTACTACTTCTCACCAGAAGAA

GAAACGAGCTGCCTGCAAATCAGTGAATGGGTCCCATAAGTACAAAGGAAACTCCAAAGATGTCAAGCCT

CCTGACCTTTGGATCCATCACGAACGACTGGAGCTAAAACCCATTGATAAATCTCCAGATCCCAATCCAA

TCATGACAGATACCCCAATCCCTCGCAACTCCCAGGACATCACCCCCGTTGACAATTCCATGGATAGCAA

TATCCATCAAAGGCGGAATTCCTACAGAGGGCACGAGTCAGAGGATAGCATGTCCACACTGGCAGGAAGA

AGGGGGATGAGGCCCAAGATGATGATGCCTTTTGATTCTCAGCCACCTCAGCCTGTGATTAGTGCTCATC

CCATCCATTCACTCGATAACCCTCACCATCATTTCCACTCCGGCAGCCTCGCTTCTCCAACCCGCAGCTA

TCTCCATCACCAGGTCAACCCGTGGCCGATTGGCACATCCTTGTCCCATTCAGACAGGGCCAATTCCACA

GAATCTGTTCGAAATACACCTAGCACAGACACCATGCCAGCTTCCTCGTCTCAGACGTGCGCTGACCATC

AGGAGGCAGAAAGCGCCACAGGAGCTTATCTGGCGAACGCTCAAGAGGAGGACTCGGCTCAGAACCTCCC

TACCGCACACGTCCGTCCTTCTCACCCGCTGAAGAGCTTCGCAGTGCCCGCAGTCCCACCAGCTGGTCCC

ACGTATGATCCCGCGTTGCCAAGCACACCGTTACTGACTCAGCAAGCTCCTAACCATCCAGTTCACTCGG

TGAAGACTGCGTCGATTGGGACTTTAGGAAGAACTCGACCTCCTATGCCAGTGGTAGTTCCTAGTGCCCC

TGACGTGCAGGAGACCACCAGGATGCTCGAGGACTCGGAAAGCAGTTATGAACCAGATGAGCTGACCAAA

GAGATGGCCCACCTGGAAGGACTTATGAAGGACCTTAATGCCATCACGACAGCATGA

>African ostrich ONECUT2

ATGGTGCCCAGCATGGCCTCGCTGCTGGACGGCGCCGCCGAGTCCCGGCCCGAGCTCTCCATCCCGCTGC

ACCACGCCATGGCCATGCCCTGCGACTCCTCGCCGCCCGGCATGGGCATGAGCGGCACCTACACCACGCT

GACGCCGCTCCAGCCCCTGCCGCCCATCTCCACCGTCTCCGACAAGTTCCACCACCCGCACCCCCACCCG

CACCCCCACCCCCCCCACCCCCCCCAGCGCCTCGCCGGCAACGTCAGCGGCAGCTTCACCCTCATGCGCG

ACGAGCGGGGGCTGCCCGCCATGAACAACCTCTACGGGCCCTACAAGGAGATGCCGGGCATGGGCCAGAG

CCTCTCCCCGCTGGGCAACGGCCTGGGCCCCATCCACAACGCCCAGCAGGGCCTCCACGGCTACGGGCCG

CCCGGCCACGACAAGATGCTCAGCCCCAACTTCGAGGCCCACGGCGCCATGCTGGCCCGGGGGGACCAGC

CCCTCCCCCGGGGGCTGGGGACCCCCCCGGCCGCCGTCATGTCCCACCTCAACGGCGCGCACCACCCCGC

GCCCCCCGGCCACACGCCGTCGCACGGGCCCGGGCTGCCCGCCGGCCGGGAGCGGCCGCCCTCCTCCTCG

GGCTCCCAGGTGAGCAGCTCCGGGCAGGTGGAGGAGATCAACACCAAAGAGGTGGCTCAGCGGATCACGG

CCGAGCTGAAGCGCTACAGCATCCCGCAGGCGATCTTTGCGCAGAGGATCTTGTGCCGCTCTCAAGGCAC

CCTGTCGGACCTGCTGCGGAACCCCAAGCCCTGGAGTAAGCTCAAGTCGGGCCGGGAGACTTTCCGGAGG

ATGTGGAAATGGTTGCAGGAGCCGGAATTTCAGAGGATGTCCGCGCTCAGGCTCGCAGCATGCAAACGCA

AAGAGCAAGAACCGAACAAAGAAAGGAACAACTCCCAGAAGAAATCTCGCCTGGTTTTCACCGATCTCCA

ACGCAGAACACTTTTCGCCATCTTCAAGGAGAACAAGCGTCCGTCCAAAGAAATGCAGATCACCATCTCC

CAGCAGCTGGGCTTGGAGCTCACCACCGTCAGCAACTTCTTCATGAACGCGCGGCGGCGCAGCCTGGAGA

AGTGGCAGGGCGATCTCAGGCCCGGGGGCTCCTCCGCGGCCTCCAGCACTTGCACCAAAGCATGA

>African ostrich POLI

ATGNNNGCAGGCCCCAGTAAGTCTCTGTCAGCAAGAAATACAGCATGCAGAGTGATTGTTCACATTGACT

TGGACTGCTTTTATGCACAAGTAGAAATGATCCGTAATCCTGAATTAAGAGAGAAGCCTTTAGGTGTGCA

ACAGAAAAACATTGTAGTTACCTGTAACTATGAAGCCAGAAAACTTGGAATTAAGAAACTGATGTCTGTC

AGGGACGCTAAAGAGAAGTTTCCTCAACTGATACTGGTTAATGGAGAGGATCTAACTCAATATAGGGAAA

TGTCATACAAGGTTACAGATTTGTTGGAAGAATTTTGTCCACTAGTGGAAAGGCTTGGGTTTGATGAAAA

TTTTGTGGATATCACAGAGATTGTAGAGAAAAGACTAAAACTACTACAACAAACTGGATGTTCCCAAGTA

TGCGTGTCCGGCCACATATACAATAACGAAACTATCAATTTACAAGATACAATGCATGTAAGACTAGCTA

TTGGATCTCGGATTGCAGAAGAGTTGAGGGAAGCTGTGCATGCTAGATTGGGCCTCACAGGCTGCGCTGG

AGTGGCTTCTAACAAACTACTGGCTAAGCTGGTTTCTGGTACCTTTAAACCAAATCAACAAACAGTTCTT

CTGCCTGAAAGCTGTCGGGATCTAATATGCAACCTTGAGCACCTCCACAAAGTGCCTGGCATTGGCTATA

AAACTACTAAACGCCTCGAAACGCTGGGCCTTAAAAGTGTGTGTGATCTCCAGGCGTTTCCATCTGCTAT

ATTAGAGAAGGAGCTTGGTGTTTCTACTGCTCAGCGTATCCAAAAACTCAGCTATGGAGAGGATGACTCC

CCTGTGACTCCATCAGGCCCTCCTCAGTCCTTTAGTGAAGAAGATTCCTTTAAAAAATGTTCATCAGAAG

TGCAAGTTAAAGAGAAAATAGAAGCATTGCTTGCTAACCTCTTAGACAGAATACACAAAGATGGAAGAAA

ACCACACACAATAAGGCTGGCCACCCGCCAGTTTTCTTCAACTAATAAATGGTTTAATCGGGAAAGTCGT

CAGTGTCCTATTCCACCTCATCTCATTCAGAAATTTGGAAAAGAAAGCAGCAGTATTATATCCCCATTGG

TTGATATTCTAATGAAACTCTTTCGAAAGATGATAAACGTAGACCTACCATTTCATCTGACGCTTCTGAG

TGTCTGCTTCTCCAACCTCAAAGATCTTCCTAGCAGCAAGAAAGGATCAATTGGTTTTTATCTAACAGAG

GTGTCACCACCTTCAGCCTCTGGTAATAATGTCCAGGAAACGGAAGATGTCTCACAGGGCCAGGCAAGCT

CTTCCTGGAGCCAGAATTTCAACAGAACTGGAAATACACCAGTGAGGAAACTTTCAGAAGAAAAGCAAAG

CTGTATAAGAAAAGCTGGAATTCTTGACTTCCCATTTCATTTGTCTCCTGGTGACATTGACCAGGAGGTC

TTCCGGGAACTTCCAGAAGATATTAGGAAAGAAATTATTTCTGAAAGAACAGGAGAAACGATCCCTACAG

AGAATGTTTTTAGTAGGCCGTCACTGTGTTTTCCAAAAGAGATAAATAGCACTTCTCCAAATTCCAAAAG

AGTAAATGATGATATGAATGCTTCAGGGTGCAGTATATACTTCAGATCAGCTCATGACTCTGCAACTGCT

CTGGCACACAGCTCCAGCGCCAGTTGTTCTTCTGAGTATCCTGGAAGTATATTGATAGATGGCGATATAG

AAAAAGACGCGACTGACTCGCTGAATTTCCGAGAGAGAGATGCGCTGGCTCTGGAAGTTGGAGCCAGCCA

AACTGTTCTGCCTGCACCTGTCTTCAGTAAAGATGAGCAAGCCTTTGGGACAACTTCTGAGGATAAAACC

CATGGTAGCAGAGAAGGAATCGTATTTCCCCTTAATGTTGACCCAAAGACTTTTTTTGAACTACCTGCAG

ATGTGCAAAAAGAACTACTAGCTGAATGGAAGAACCAGGAACTTGCATCTAAAATGTTTATGGGTAAACC

TCCTGAAAAGCCTAAAGCAAACAGAAGAAGAAAGAATACAGCATCGTGTTTTTCACAATCTAACAGTTTA

CTAAGATATTTTAAACCACAGTGA

>African ostrich RAB27B

ATGACTGATGGAGACTATGATTATCTGATCAAACTCCTGGCCCTCGGAGACTCTGGGGTTGGAAAAACAA

CGTTCCTGTACAGATATACTGATAACAAATTTAATCCAAAATTCATCACGACAGTAGGGATAGATTTTCG

GGAAAAACGAGTGGTGTACAATAGCAGAGGACCAAATGGATCTCCAGGAAAAGCCTTTAAGGTACATCTC

CAGCTTTGGGACACAGCCGGACAGGAAAGATTTCGAAGTCTTACCACAGCATTTTTCAGAGATGCGATGG

GCTTTTTACTGATGTTTGATCTCACCAGTCAACAGAGCTTCTTAAATGTCAGAAATTGGATGAGTCAGCT

GCAAGCCAATGCGTATTGTGAGAATCCAGATATAGTGTTGATTGGTAATAAAGCTGATTTATCAGACCAA

AGGGAGGTAAATGAAAGGCAAGCAAAAGACCTGGCGGACAAATATGGCATACCATACTTTGAAACGAGTG

CTGCTACTGGACAAAACGTGGAGAAGGCTGTGGACACTCTCCTGGACTTGATAATGAAACGCATGGAGCA

GTGCGTGGACAAGACACAAGTCTCTGACACCGCCAATGGAGGAAGCTCAGGAAAGCTAGATTCGGCAAAA

CCGGAGGAGAAAAAGTGTGCCTGCTAA

>African ostrich RPL17

ATGGTCCGCTACTCTCTGGATCCGGAGAACCCCACGAAATCATGCAAGTCAAGGGGATCCAACCTGCGAG

TCCATTTCAAGAACACTCGTGAAACTGCCCAAGCTATCAAGGGCATGCATATCCGGAAGGCCACCAAGTA

CTTGAAGGATGTGACCCTAAAGAAGCAGTGTGTTCCCTTCCGTCGCTACAATGGCGGAGTTGGTAGATGT

GCCCAGGCCAAGCAGTGGGGCTGGACACAGGGACGCTGGCCCAAGAAAAGTGCAGAGTTCTTACTGCACA

TGCTCAAAAATGCAGAGAGCAATGCTGAGCTGAAGGGTCTTGATGTGGATTCTCTGGTAATAGAGCACAT

CCAGGTCAACAAGGCTCCCAAAATGCGCAGGCGTACCTACAGAGCTCATGGTAGGATCAACCCCTACATG

AGCTCCCCCTGCCATATTGAGATGATCCTCACTGAGAAAGAGCAGATTGTTCCCAAACCGGAAGAAGAAG

TTGCTCAAAAGAAAAAGATATCCCAAAAGAAGCTGAAGAAGCAAAAGCTCATGGCTCGGGAGTAA

>African ostrich SKA1

ATGGCTTCTTTAGATCTGCAGGACTTATGCTTGCACATCAACATGAAGATTTCAACTATCAAAAAGACTC

TTCAATTAAGAAACATAGGTCAAGAACCATCACTGAAATCTATGCTCTGTAAAATAGGACATGAGATGGT

TCTCTTAAATGAGCTCCTAAACAAAATGGAAATGGAAGTGCAACAGCAAGAAAAGTTGAAGAATTTGCTC

AAAGAGCTCCAGAAGTCTGCTGAGAGAGATCAAAGTGAAGCACATCACCTCTGTGAAAATATTCCTCCCC

ATCTGCCTAAACCAACTCAGAGCTGCATCACTGGGCTAACGGTAAAATGTGGAGAACAAACAAAAGTCAT

AGAACCTGAACGTGCAAAGAAATCTGTAAAAGAGCCAAGATTTATTAAAGAAGCAGCCTTAATAACTGCA

GAAGAATTTGAAAGTGTTCCTGCGTACATGAGAGGTCGTTTAACATATGATCAAATTAACGCGGTGGTTC

AAGACATGAACAAGGCTGTGGTTAGCAAGTACAGGATCCTGCATCAGCCACTGAAATCTATGAATGCCAC

TGTCAGAAATCTCTACCACAGATTCCTGGAAGAAGAAACTAAGGATACAAAAGGTGAATTCTTTATTGTG

GAGGCTGATATCAAGGAGTTCACTCTGCTGAAAGTCGATAAGCGCTTTCATAGCATCCTCAATATCCTGC

GCCACTGCCAGAGAGTGAGAGAAGTCCGTGGCTCACGACTTGTCCGCTACGTCATCTGCTAG

>African ostrich SMAD4

ATGGACAATATGTCTATTACTAACACGCCAACAAGTAATGATGCTTGTCTGAGCATTGTTCACAGCTTGA

TGTGCCATCGACAAGGTGGAGAGAGTGAAACTTTTGCCAAACGCGCAATTGAAAGTTTAGTTAAAAAGCT

AAAGGAGAAAAAAGATGAATTGGATTCTTTGATTACAGCTATAACTACAAATGGAGCTCATCCTAGCAAG

TGTGTTACAATACAGAGAACGCTGGATGGGAGGCTTCAGGTGGCTGGTCGCAAGGGATTCCCTCATGTGA

TTTACGCTCGTCTTTGGAGGTGGCCTGATCTTCATAAAAATGAACTCAAGCATGTTAAATATTGTCAGTA

TGCTTTTGACTTAAAATGTGACAGTGTCTGTGTAAATCCTTACCATTATGAGCGTGTAGTATCGCCTGGC

ATCGATCTTTCAGGACTGACACTACAGAGTTCTACTCCATCAAGCATGTTGGTGAAAGATGAATATGTTC

ATGACTACGAGGGGCAGCCGTCGTTGTCGTCTGCTGAAGGCCATTCAGTCCAAACCATCCAGCATCCACC

AAGTAACAGAGCATCTACAGAGCCTTACAGCACCCCAGCCATGCTAGCTCCTGCTGAGGCTAGCACTACC

AGCACCACTAATTTTCCCAACATTCCTGTGGCTTCAACAAGTCAACCTCCCAGTATATTGACAGGTAGCC

ATAGTGATGGACTCTTACAGATTGCTTCAGGGCCTCAGCCAGGAACTCAGCAGAATGGGTTTACAGCTCA

GCCAGCTACTTACCATCACAATAGTACTACAACTTGGACTGGAAGTCGAACAGCAGCCTACACACCTACC

ATACCTCACCACCAGAATGGCCATCTTCAGCATCATCCACCTATGCACCCTGGACATTACTGGCCAGTTC

ACAATGAACTTGCATTCCAGCCTCCTATATCAAATCATCCTGCTCCAGAATATTGGTGTTCAATCGCGTA

CTTTGAAATGGATGTGCAAGTTGGGGAAACATTTAAGGTTCCTTCAAGCTGTCCAATTGTTACTGTTGAT

GGATATGTGGATCCTTCTGGAGGAGACCGTTTTTGCCTGGGCCAGCTTTCCAATGTGCACAGAACAGAAG

CCATTGAGAGAGCAAGGTTGCACATAGGTAAAGGGGTACAGTTGGAGTGCAAAGGAGAAGGTGACGTGTG

GGTTAGATGCCTCAGTGACCATGCAGTCTTCGTCCAGAGTTACTACCTGGATAGAGAAGCAGGGCGTGCG

CCAGGTGATGCTGTTCACAAGATTTACCCAAGTGCATATATAAAGGTTTTTGATTTACGCCAGTGTCACC

GTCAGATGCAACAGCAGGCTGCCACTGCCCAAGCCGCTGCTGCTGCCCAAGCCGCAGCAGTAGCAGGAAA

TATCCCTGGACCAGGATCAGTAGGTGGAATAGCCCCAGCCATTAGTTTGTCAGCTGCCGCTGGAATTGGT

GTAGATGACCTTCGCCGCTTATGCATACTCAGGATGAGTTTTGTAAAAGGTTGGGGACCTGATTACCCAA

GACAGAGCATCAAAGAGACACCCTGCTGGATTGAAATTCACTTACACCGTGCCCTCCAGCTTCTAGATGA

AGTACTTCATACCATGCCTATTGCAGACCCACAACCTTTAGACTGA

>African ostrich SMAD7

ATGTTCAGGACCAAACGGTCGGTGCTCGTCCGGCGGCTCTGGCGGAGCCGCGCTCCCGGCGGCGAGGAGG

AGGCGGGCGAGCCCGGCGGCGGCGCGGCGGCGGCCGAGGCCAAGGCGCTGACCCACGCCGTGCTCAAGCG

GCTCAAGGAGCGGCAGCTGGAGGGGCTGCTGCGCGCCGTGGAGTCGCGCGGCGGGGCGCGCACGCCCTGC

CTGCTGCTGCCCGCCAAGGCGGCCGACGCGCGCCTGGGCGCGCCCTGGTACCCGCTGCCGCTGCTGCTCT

GCAAGGTGTTCCGCTGGCCCGACCTGCGCCACTGCGCCGAAGTCAAGCGCCTCTGCGGCTGCGAGTCCTA

TGGCAAGGCGCACCCCGAGCTCGTCTGCTGCAACCCGCACCACCTCAGCCGGCTCTGCGAGCTNGAGTCT

CCCCCTCCACCCTACTCCAGATATCCGATGGATTTTCTCAAACCAACCGCAGATTGTCCAGACTCTGTGC

CTTCCTCCACTGAAACAGGGGGAACTAATTGTCTAGCCCCTGGGGGGCTTTCAGATTCCCAAGTTCTTCA

GGAGCCGGGGGATCGGTCACACTGGTGCGTGGTGGCATACTGGGAAGAGAAAACGCGCGTGGGTCGGCTA

TATTCTGTCCAAGAGCCCTCCCTGGATATCTTCTATGATCTACCTCAGGGGAACGGTTTCTGCCTCGGCC

AGCTCAATTCGGACAACAAGAGCCAGCTGGTGCAGAAAGTGCGCAGCAAAATCGGTTACGGTATCCAGCT

CACCAAGGAAGTGGACGGCGTGTGGGTCTACAACCGCAGCAGTTACCCCATCTTCATCAAGTCGGCCACA

CTGGACAACCCCGACTCGAGGACGTTGCTGGTTCACAAAGTGTTTCCAGGTTTTTCCATCAAGGCGTTTG

ACTACGAGAAGGCGTACAGCTTGCAGAGGCCTAACGACCATGAGTTCATGCAGCAACCATGGACCGGATT

TACTGTTCAGATCAGCTTTGTGAAAGGCTGGGGCCAGTGCTACACGAGACAGTTTATCAGCAGTTGCCCG

TGCTGGTTGGAGGTTATTTTTAATAACCGATGA

>African ostrich ST8SIA3

ATGNNNCTGCGGGCGTGGGTCCGCGTGTGCGCGCTCCTCCCCAGGTCCCAGTTCGCCCTGAAGTTCCTGG

ACCCCTCGTTTGTGCCCATTACAAACTCCCTGACCCACGAGCTGCAGGAGAAGCCCTCCAAGTGGACGTT

CAACAGGACAGCGTTCGCACATCAGAGGCAAGAAATCCTTCAGCATGTTGATGTAATAAAAAATTTTTCT

TTGACTAAGAACAGTGTTCGGATTGGACAGCTGATGCATTATGATTATTCCAGCCATAAGTATGTTTTTT

CTATTAGCAATAACTTCAGATCACTGCTTCCAGATGTGTCACCAATCCTGAATAAGCATTATAACATTTG

CGCCGTGGTTGGGAATAGTGGAATCCTGACTGGAAGTCAGTGTGGACAAGAAATAGATAAATCTGATTTT

GTTTTTCGTTGCAATTTTGCTCCAACTGAGGCTTTCCAAAGAGATGTTGGAAGGAAAACCAATCTTACAA

CCTTCAATCCCAGCATCCTGGAGAAGTATTACAACAATCTTTTGACCATTCAGGATCGCAACAACTTTTT

TTTAAGTTTAAAAAAGCTTGATGGGGCCATTCTTTGGATCCCCGCTTTTTTCTTCCACACGTCAGCAACA

GTCACAAGAACACTGGTTGACTTCTTTGTTGAGCATAGAGGGCAACTAAAAGTCCAGTTGGCTTGGCCAG

GAAATATAATGCAACATGTTAACAGATACTGGAAAAACAAGCATTTGTCACCCAAGCGCCTGAGCACAGG

TATTCTCATGTACACCCTTGCTTCTGCGATATGTGAAGAGATTCACTTGTATGGATTCTGGCCGTTCGGA

TTCGATCCCAACACCCGGGAAGATCTCCCATACCATTACTATGATAAGAAAGGAACAAAGTTTACGACCA

AGTGGCAGGAGTCCCACCAGCTGCCTGCAGAGTTCCAGCTGCTCTACAGGATGCACGGTGAAGGACTGGC

CAAACTCACCTTGTCGCATTGTGCCTAA

>African ostrich STARD6

ATGGACTATAAGAAAATTACGGATGAAGTTTCTACAAAAATTCTATCATACAACCAAGATACTTCAGGAT

GGAGAGTGATAAAAGTTTCAAAAAATATTACAGTTGCTTCAAAGCCTTCAAAAGAGTATTCAGGAAACAT

ATACCGAGGAGAAGGGATAATTAAGGAAGTCCCTAGTAAAATTATTCCTTTTATGTATCTTCCTGAATAT

CGAAACAAGTGGGACAAAGCATTACAATCTTACAAGCTGTTGGAAAAGATTGACCAGGATACCGGTATAT

ACCACAGTGTAACACACAGTTATGGTATGGGACTGATTTCATCAAGAGATTTTGTTGACTTGGTGCATGT

TAAACCCTATCCTGGTGGTATCCTTACAACTAACTCTGTCAGTGTGGAATATTCAGGATGCCCTCCAACT

CCTCCTTGCGTCCGTGGCTATAACAATCCTTGTGGGTATGTGTGTTCACCCTTGCCAGAGAATCCAGAGC

ATTCCAAGCTAGTTGTATTTATTCAGCCAGAATTAGGAGGAATGCTTCCCTACTCTGTGGTGGAGACAGC

AATACCTACTACTCTCATAAATTTAATAACTGAAACAAGAGCTGGACTGAAAGGCTTGAAAGACCATAAT

TAA

>African ostrich TCF4

ATGCATCACCAACAGCGAATGGCTGCCTTAGGGACGGACAAAGAACTGAGTGATTTACTGGATTTCAGTG

CGATGTTTTCACCTCCTGTGAGCAGTGGGAAAAATGGACCAACTTCTCTGGCAAGTGGACATTTTACTGG

CTCAAATGTAGAAGACAGAACTAGCTCAGGGTCCTGGGGGAATACAGGACATCCTAGTCCATCCAGGAAC

TATGGCGATGGGACTCACTATGATCACATGGCGAGCAGAGACCTTGGCTCACATGACAATCTCTCTCCTC

CTTTTGTCAATTCCAGAATACAAAGTAAAACAGAAAGGGGTTCATACTCGTCGTATGGAAGAGACTCGAA

TTTACAGGGTTGCCACCAGCAAAGTCTCCTTGGAGGTGACATGGATCTCGGCAACCCGGGCGCGCTCTCC

CCCACCAAACCCGGCTCGCAGTACTATCAGTATTCTAGCAATAACCCCCGGAGGAGGCCTCTGCACAGCA

CCTCTATGGAAGTACAAACAAAGAAAGTGCGCAAGGTCCCTCCAGGTTTGCCGTCTTCGGTTTATGCCCC

GTCAGCAAGCACTGCCGACTACAATAGGGATTCACCAGGTTATCCATCCTCAAAACCAGCAGCCAGCACT

TTTCCTAGCTCCTTCTTCATGCAAGATGGCCATCACAGCAGTGACCCGTGGAGCTCCTCCAGCGGGATGA

ACCAGCCCGGTTACGGAGGGATGCTGGGCAACTCGTCTCATATTCCACAGTCCAGTAGCTACTGCAGTCT

GCACCCCCATGACCGCTTGAGCTACCCATCACACTCCTCAGCAGACATCAATTCCAGTCTTCCTCCGATG

TCCACCTTCCACCGCAGTGGCACGAATCATTACAGCGCATCCTCTTGCACACCCCCAGCCAATGGCACGG

ACAGTATAATGGCAAACAGAGGAAGTGGGGCAGCAGGCAGCTCGCAGACTGGTGATGCGCTGGGGAAAGC

ACTTGCCTCTATCTATTCTCCAGATCACACCAACAACAGCTTTTCATCAAATCCTTCAACTCCTGTTGGT

TCTCCCCCTTCTCTCTCAGCAGGCACAGCTGTTTGGTCTAGAAATGGAGGTCAAGCGTCATCATCTCCCA

ATTATGAAGGTCCCTTACACTCTTTGCAAAGCCGGATTGAGGACCGCTTGGAGCGGCTGGACGACGCTAT

TCATGTGCTGCGGAACCACGCCGTGGGGCCGTCCACCGCCATGCCCGGCAGCCATGGCGACATGCACGGA

TTAATAGGGCCGGCGCACAATGGAGCGATGGGAGGTCTCGGCTCCGGCTACGGCACCGGCCTGCTCTCGG

CCAATCGGCACTCGCTCATGGTCGGCGCTCACCGCGAGGACGGCGTCAGCCTGCGCAGCAGCCACTCGCT

CGTGCCAAACCAGGTCCCGGTCCCCCAGCTGCCCGTCCAGTCCGCCACCTCCCCGGACCTCAACCCGCCC

CAGGATCCCTACAGGGGCATGCCAACCGGACTGCAAGGGCAGAGCGTCTCTTCAGGTAGCTCCGAAATCA

AGTCTGATGACGAGGGAGACGAAAACCTCCAGGACACAAAATCTTCTGAGGACAAGAAACTAGAGGATGA

CAAGAAGGATATCAAATCAATTACTAGGTCAAGATCTAGCAATAACGATGACGAAGACCTGACACCTGAG

CAGAAGGCCGAGAGAGAAAAGGAGAGGAGAATGGCCAACAACGCTCGCGAGCGCCTGCGCGTGCGCGACA

TCAACGAGGCTTTCAAGGAGTTGGGCCGGATGGTGCAACTCCATCTGAAGAGCGACAAGCCCCAGACCAA

GCTCCTGATTTTACACCAGGCTGTAGCTGTCATCCTCAGCTTAGAGCAGCAAGTCAGAGAAAGAAATCTG

AACCCTAAAGCAGCGTGTCTGAAAAGAAGGGAAGAAGAGAAAGTATCTTCAGATCCTCCTCCACTTTCCC

TGGCGGGACCCCACCCTGGGATGGGAGATGCATCCAATCACATGGGACAGATGTAA

>African ostrich TSPAN3L

ATGGACTGCGGCGTGATCACCTCCAAGACGGTGCTGGTGCTGCTGAGCCTCGCCTTCTGGGCAGCAGCAG

CTGGTCTTAGCTATGTTGGGGGATATGTCATTAATACCTACAAGAGCTATGACAACTTCCTGCAAGACAA

GTACGCTCTGTTGCCAGCGGTGATCATTATTTGCGTTGCCATAGTAATGTTCATCATTGGGTTGATCGGC

TGCTGTGCCACCCTCCGGGAGTCACGAGTTGGACTGGGGCTGTTTTTGGCCATTATTCTGGTTATCTTTA

TTGCAGAAGTGTCAGCTTTTGTCTTGGGGTTTGTTTACAGGGAAAAGGTTAAAACTGACGTGCAGGGCAC

AATGCGTTCGGTCTTCCAAAAGTATGATGGGAAAAATCCAGAGTCTACGGTTGTGGATTACTTGCAAAAA

CAGCTTCACTGTTGTGGGGTAAAGAACTACAGTGACTGGACGACCACACAGTGGTTTAACTCCACGAGTA

ATAGCAGTGTCCCTCTGAGCTGCTGCAGGCAAGATCTGAACAACTGCACGGGGCGTCTGGATCAGCCACA

GGAACTCAATACACAGGGCTGTGCGGAGGAGCTGGAGTCTGGGCTGCAGAGTGTACTCAGCTATGCTATG

CTTGTAATCCTGGGGTTTGCCATCGTAAAGTTCTTTGGCATGCTGAGCGTCTGTGTGCTCACTTGCAAGA

GAGAAGACAGTGGATATCAGCCTCTTTACTCAGGGGTGTTTGCTTGA

>African ostrich TXNL1

ATGCGCGGATGTGGGCCTTGTTTAAGGATAGCTCCAGCTTTCAATGCCCTGAGTAACAAATATCCTCAGG

CAACTTTTTTGGAGGTGGATGTACATCAGTGCCAGGGAACAGCTGCTACCAATAATATATCAGCCACACC

AACATTTCTGTTTTTTCGAAACAAAGTGCGAATCGACCAGTATCAAGGAGCAGATGCTGTAGGTTTAGAA

GAAAAAATTAAACAGCACCTGGAGAATGACCCTGGAAACAATGAAGATACAGATATCCCAAAAGGATATA

TGGATTTAATGCCATTTATCAATAAAGCTGGCTGTGAATGTCTTAACGAAAGTGATGAGCATGGCTTTGA

TAATTGTTTACGTAAAGACGCTACCTACCTGGAATCAGACTGTGATGAGCAGCTACTTATTACTGTAGCT

TTTAGTCAACCTGTCAAGCTTTATTCTATGAAACTTCAGGGGCCAGATAACGGGCAAGGTCCAAAGTACA

TAAAAATTTTTATTAACCTTCCTCGATCTATGGATTTTGAGGAAGCAGAACGAAGCGAACCAACTCAAGC

CCTGGAGCTAACACCAGAGGATATTAAAGAAGATGGTATTGTCCAGCTTCGCTATGTAAAATTTCAGAAT

GTTAACAGTGTAACTTTATTTGTCCAGTCCAATCATGGTGATGAAGAGACAACAAGAATTACGTACTTCA

CATTTATTGGAACTCCAGTCCAAGCAACAAATATGAATGACTTCAAGAGAGTAGTTGGCAAAAAAGGAGA

GAGCCACTAG

>African ostrich WDR7

ATGGCTGGAAATAGCCTTGTTCTACCCATTGTTCTTTGGGGCCGTAAAGCTCCCACCCACTGCATATCAA

CGTTGCTGTTAATGGACGACGTGTCAATGATTGTCACAGGCTGTCATGATGGACAAATATGTCTCTGGGA

CCTCTCTTTAGATTTAGAGATTAATCCCAGAGCTCTGTTGTTTGGTCATACAGCCTCAATTACTTGTTTA

TCAAAGGCCTCTGCTTCCAGTGAGAAGCAGTATATAGTGAGTGCATCAGAAAGCGGGGAGATGTGCCTGT

GGGATGTGAATGATGGGAGATGCATAGAGTTTACTAAATTAGCCTGTGCACACACTGGCATACAGTTCTA

CCAGTTCACTGTTGGGACTCAGCGTGAAGGGAGACTATTATGCCACGGACATTATCCAGAAATTCTTGTC

GTGGATGCTACCAGCCTTGAAGTTCTTTACTCTTTATTATCAAAGATATCTCCTGACTGGATCAGCTCCA

TGAGTATCATTCGATCCAATAGAACACAAGAGGATACTGTTGTCGCTGTTTCGGTGACTGGCATTTTGAA

AGTATGGATAATAACCTCTGAAGTTAGTCGCATGCAGGATACTACGCCAGTATTTGAAGAGGAGTCTAAA

CCTATTTATTGTCAAAACTGCCAAAGCATTTCTTTCTGTGCATTTACCCAACGGTCATTGTTGGTAGTGT

GCTCCAAATACTGGAGGGTTTTTGATGCTGGAGATTATTCCCTCTTATGTTCAGTCCCTAGCGAGAATGA

ACAGACCTGGACTGGCGGTGACTTTGTGTCAGCTGATAAAGTGATTGTATGGACAGAAGATGGGCAAAGT

TTTATATACAAATTACCAGCCAGTTGTCTACCAGCTAGTGATTCATTTCGCAGTGGTGTGGGAAAAGCAG

TAGAAAATTTAATTCCACCTTTATTGTACAGTGTATTGGACAGAGCGGATAAACAGTTACTAATATGTCC

CCCAGTTACTCGATTCTTCTATGGACGTAGAGAGTTTTGCAATAAACTCTTAATCCAAGGAGACTCTTCA

GGGAGACTGTGTATTTGGAGTGTGCCTGATACATTTGAACAACAGGACAGTGCAGAAGGACTACAAGCAA

CCACCTCAGTATCCCTACAGGAAGCTTTTAATAAACTTACCCCTCACCCTGCTGGAATTATAGACCAATT

AAGCTTAATACCAAACATCAATGAACCGCTTAAAGTGACAGCGAGTGTATATATCCCAGCGCATGGGCGT

CTGGTTTGTGGTCGTGAAGATGGAAGCATCGTTATTGTGCCAGCAACGCAAACTGCTATAGTTCAGCTTC

TGCAAGGAGAGCATATGCTTAGGAGAGGTTGGCCACCTCACCGGACTCTCCGAGGCCATCGAAACAAAAT

TACATGTTTACTCTATCCTCATCAGGTTTCTTCTCGTTATGATCAAAGGTATTTGATCTCAGGTGGTGTG

GATTTCTCAGTCATCATATGGGATATATTTTCTGGAGAGATGAAACATATCTTCTGTGTACATGGTGGAG

AAATTACACAGCTTCTAGTTCCACCAGAAAACTGTAGTGCAAGAGTCCAGCACTGTATTTGCTCTGTTGC

CAGTGATCACTCAGTAGGTCTTCTGAGTTTGCGGGAGAAAAAATGTATCATGCTGGCATCCCGTCACCTT

TTTCCTATCCAAGTAATAAAATGGAGGCCTTCAGATGACTATCTGGTGGTGGGGTGTTCGGATGGATCTG

TGTATGTCTGGCAAATGGATACTGGTGCGCTGGACAGGTGTGTGATGGGAATAACAGCCGTTGAAATCTT

GAGCGCTTGTGATGAGGCAGTTCCAGCTGCTGTAGACTCCCTCAGTCATCCGGCCGTCAACCTGAAGCAG

GCCATGACAAGACGTAGTCTTGCTGCCCTGAAAAACGTGGCCCATCAGAAACTACAGACTCTTGCCACTA

ACCTTTTAGCTTCAGAAGCATCTGACAAAGGAAATTTACCTAAATATTCACACAACTCCCTGATGGTTCA

AGCTATAAAGACCAACCTAACAGATCCGGATATACATGTGCTCTTCTTTGATGTAGAAGCACTGATTATT

CAGCTACTGACGGAAGAAGCCTCTAGGCCTAATACTGCACTTATTTCCCCAGAGAACTTACAGAAAGCAT

CTGGCAGTTCTGACAAAGGAGGCTCTTTTCTGACTGGCAAACGAGCCGCAGTTCTCTTCCAGCAGGTCAA

GGAAACAATCAAAGAGAATATAAAAGAACATCTCCTCGATGATGAAGATGAGGATGAAGAGTCAATAAGA

CAGAGGAGAGAAGACGGTGACCCAGAATATCGCTCTAGCAAATCTAAACCATTAACCTTGTTAGAATATA

ATCTAACCATGGATACAGCAAAGCTTTTTATGTCTTGTCTTCATGCCTGGGGCTTGAATTCTGTTCTAGA

TGAGCTTTGCCTTAATCGTCTTGGGATGCTTAAGCCACACTGTTCTGTGTCCTTTGGCCTGCTGTCTAGA

GGAGGCCACATGTCTCTCATGCTTCCTGGTTATAATCAGTCTATAGGTAAACCGCCGTATGATAGTATGG

AATTAGGAAGAAAAATGTCCATTACCGAAGGACTAGGAAAGGGGACATACGGTGTATCACGTGCTGTCAC

CACTCAGCATCTCCTGTCTATTATTTCATTGGCAAACACATTGATGAGTATGACTAATGCCACTTTTATT

GGAGACCATATGAAGAAAGGTCCAACAAGGCCTCCTAGGCCAGGCACTCCTGAGATGTCAAAAGTGAAGG

CACCTCCTTCAATTTCAAGTCATGCAGCTCAAGGACAAATTAAGCAAGTTGCTCCTGCTGTTTCTTCTAG

CACTGAAGCTGGTCACTCTGGCTCTGACACTGCTCCTACTTTACATACCTGTTTCTTAGTAAACGAAGGG

TGGAGCCAGCTCGCTGCTATGCACTGTGTTATGCTCCCTGACCTGTTAGGACTGGACAAATTTAGACCTC

CTCTTCTGGAGATGCTAGCTCGCAGGTGGCAGGATCGATGCTTGGAGGTAAGAGAAGCTGCCCAGGCGTT

ACTGTTAGCAGAACTGAGAAGAATCGAACAGGCAGGTCGGAAAGAAACAATTGATGCATGGGCTCCGTAT

TTACCACAATATATTGACAGTGTTATATCACCTGGAGTAACAACAGAAGCCATTCAGACTGGTAGTGCAA

GCCCAGATTCGTTGGGAGCAGAAGCGAAAGTTCAGGAAGAAGAGCATGATCTGGCTGATGATGACATCAC

AGCAGGTTGTCTGTCTGGTCTCCCACAAATGAAAAAAATATCTACCTCGTATGAAGAGAGAAGGAAGCAA

GCTACCGCCATTGTTTTGCTTGGAGTGATTGGAGCAGAGTTTGGAGCTGAAATTGAACCTCCAAAACTTT

TGACTCGGCCACGCAGTTCTAGTCAAATTCCTGAGGGATTTGGTTTAACTAGTGGTGGATCAAATTATTC

CTTGGCAAGGCATACATGCAAGGCGCTGACGTTCCTGCTGCTGCAGCCGCCCAGCCCCAAGCTGCCCGCG

CACAGCACCATCCGCCGCACCGCCATCGACCTCATCGGCCGCGGCTTCACCGTCTGGGAGCCCTACATGG

ACGTCTCCGCCGTGCTCATGGGGCTGCTGGAGCTCTGCGCCGACGCCGAGAAGCAGCTCGCCAACATCAC

AATGGGGCTGCCGCTGAGTCCCGCGGCCGACTCGGCGCGCTCGGCACGCCACGCTCTCTCGCTCATCGCC

ACGGCCAGGCCACCCGCCTTCATCACCACCATCGCCAAGGAGGTGCACAGGCACACGGCCCTGGCCGCCA

ACACGCAGTCCCAGCAGAATATTCACACCACTACCCTTGCCCGGGCCAAAGGAGAGATCTTGAGAGTCAT

CGAGATACTTATTGAGAAGATGCCTACCGATGTAGTGGACCTTCTTGTGGAGGTTATGGACATCATCATG

TATTGCCTTGAAGGATCTTTGGTTAAGAAAAAAGGTCTTCAGGAATGCTTTCCAGCCATCTGCAGGTTCT

ACATGGTCAGCTATTATGAGCGGAGTCACAGAATAGCAGTTGGAGCTCGCCATGGTTCAGTGGCCCTCTA

CGACATCCGGACTGGGAAATGTCAGACTATCCATGGCCATAAAGGACCCATAACTGCTGTAGCTTTTGCC

CCTGATGGCCGGTACCTTGCCACGTACTCCAATTCAGACAGTCATCTCTCCTTCTGGCAGATGAACACCT

CGCTGCTGGGGAGCATCGGCATGCTGAACTCCGCGCCCCAGCTCCGCTGCATCAAGACCTACCAGGTGCC

CCCCGTGCAGCCCGCTTCTCCGGGGTCCCACAACGCGCTCAAGCTGGCACGACTGATCTGGACGTCCAAT

CGTAACGTCATCCTCATGGCTCATGACGGCAAGGAGCACCGGTTTATGGTCTAG

>Anna's hummingbird DCC

ATGGAGAATTGTCTTGGATGTCTTTGGGTACCAAAGGTGGTTTTTCTCCTCTTCGGGTTTATCCTGCTGA

GCCTTCTCCAGCCCATTTTAGGTTCCCAAACCAAGCCTTTCACATCCTTGAGGTTCTTGGCTGAGCCTTC

AGATGCTGTCACCATGCGTGGAAGCAACTTGGTGTTGAATTGCAGAGCAGAATCAGACAGAGGAACCCCA

GTGATCAAGTGGAAGAAAGATGCTGTCTTCTTGAACCTGGCAGTAGATGAAAGGAGGCAGCAGTTGGCCA

ATGGATCCCTCTTGATAGAAAATATTGTCCATTCCAGACATCACAAACCAGATGAAGGGCTCTACCAGTG

TGAAGCATCTCTAGAAGGCATTGGAGCCATCCTCAGCAGGACAGCAAAGGTCATGGTTGCAGGTCCCCTG

AGGTTTCTTTCCCAGACAGAATCCGTCACAGCTTTTGCAGGTGACACCATTCTCCTCAAATGTGAAGTTG

TTGGTGAGCCCATGCCCGTGGTGCACTGGCAGAGGAACCAGGAGGACTTTATCCCCAGCCCCACTGACCC

ACGGGTGGCTGTCCTGCCCTCAGGAGCTTTACAGATCAGCAGGCTTCAACATGGGGACACTGGGATCTAC

AGATGCCTGGCCAAAAACCCAGCCAGTTCCAGGACTGGGAATGATGCAGAAGTCAGAGTTTTGGCAGATC

CAGGTCTGCACAGGCAGCAGTTTTTCCTGCAGCGCCCAGCCAGCGTGGTGGCCATGGAAGGGAAGGATGC

TGTTCTGGAATGCTGTGTTTCTGGCTACCCCCCTCCCACTTTCACCTGGCTGAGAGGAGATGAAGTGCTC

TCCATGAGGTCCAAAAAGTATTCCTTACTGGCTGGCAGTAATTTACTCATCTCTAATGTGACTGATGATG

ATTCTGGGACGTACACATGTGTGGTCACCTACATGAATGAGAACAGCAGTGGCTCTGCAGAGCTCTCAGT

GATGGTTCCCCCATGGTTTGTAATTCACCCTTCCAATATTTATGCCTATGAGAGCATGGATGTGGAATTT

GAATGTGCTGTGGCTGGAAAACCTGTTCCTACAGTGGAGTGGATCAAGAATGGAGAAGTGGTCATTCCCA

GTGACTATTTTCAGATAGTGGGTGGCAGCAACTTAAGGATTTTGGGCTTGGTCAAGTCAGATGAAGGTTT

TTATCAGTGTGTAGCTGAAAATGAAGCTGGGAATGCTCAGGCCAGCACACAGCTCATCATCCCAGAGCCA

GCTGTCCCAAGTTCCAGTGTCCTCCCCTCTGCCCCCCGAGATGTGGTCCCTGTTTTGGTCTCCAGCCGCT

TTGTCCGTCTCAGCTGGCGCCCACCTGCAGAAGCCAGAGGCAACATCCAGACCTACACAGTCTTCTTCTC

CAGGGATGGAATCAACAGGGAAAGAGCAGTGAACACATCTCAATCTGGGATGCTTCAGCTGACTGTGGGC

AACCTGAAGCCAGAGGAGACCTACACCTTCCGAGTGCTGGCATACAATGAGTGGGGACCAGGAGAGAGCT

CTCATCCCATCAAGGTTGCCACCCAACCAGAGTGCAAGTTCCTGGACCAGTGGAAAACCTCCGGGCTGTG

TCTACCTCACCTACCTCCATTCTGGTCTCCTGGGATCCTCCAGCCTATGCAAATGGTCCTGTTCAAGGCT

ACAGACTCTTCTGCACTGAGACAGCAACTGGGAGAGAGCAGAACGTGGAGGTGGAGGGGCTGTCCTATCA

CCTGGAAGGGCTGAAGAAGTTCACAGAGTACACTCTGCGTTTCCTGGCCTACAACCGCTACGGCCCCGGC

GTCTCCACCGAGGAGGTGGTGGTCACCACCCTTTCAGATGTGCCCAGCGCGATGCCTCAGAACGTCTCCT

TGGAAGTGGTGAACTCCAGGAGCATCAAAGTGAGCTGGTTGCCTCCACCACCAGGTACTCAAAATGGATT

TATTACAGGCTATAAAATCCGACACCGCAAGACAACCCGCAGGGGGGAGATTGAAACACTGGAGCCAAAC

AACCTCTGGTACTTGTTCACAGGACTTGAAAAAGGAAGCCAGTACAGTTTCCAGGTGGCTGCCATGACAG

TGAATGGGACAGGGCCCCCCTCGGACTGGTACACAGCAGAAACACCTGAGAATGATCTTGATGAATCCCA

GGTTCCTGACCAGCCAAGCTCTCTTCATGTCAGGCCCTTGACCACAAGTATTGTCATGAGTTGGACCCCA

CCACTCAACCCCAACATCGTCGTCCGCGGGTACATCATCGGCTACGGCGTGGGCAGCCCCTACGCAGAGA

CTGTGAGGGTGGACAGCAAACAACGGTATTATTCCATAGAAAACTTGGAGCCAAGTTCCCATTATGTCAT

CTCCTTAAAAGCCTTCAACAACGCAGGGGAAGGAGTCCCTCTCTATGAAAGTGCCACCACCAGGTCCATG

ACAGATCCCATTGACCCATTAGAAGTTGATTTTTATCCTTTGCTTGACGATTTCCCTACCTCAGTCCCAG

ATATCTCCACCCCCATGCTCCCACCAGTAGGTGTCCAGGCTGTTGCACTGACACATGATGCTGTGAGGGT

CATCTGGGCAGACAACTCTGTCCCCAAAAACCAGAAGACACCAGAGGTTCGGTTCTACACGGTCCGCTGG

AGGACGAGCTACTCCACGAGTGCCAAGTACAAGTCAGCAGACACGACTGCCCTGAGTCACACAGTGATGG

GGCTGAAGCCCAACACCATGTATGAGTTCTCTGTCATGGTCACCAAAGGGAGGAGATCCAGCACCTGGAG

CATGACTGCTCATGCCACCACCTACGAAGCAGCTCCAACCTCTGCTCCCAAGGATCTGACAGTCATTACC

CGGGAAGGGAAGCCCCGGGCTGTCATTGTCAGCTGGCAACCACCCCTGGAAGCCAATGGAAAAATCACTG

CTTACATCCTCTTCTACACTTTGGAGAAGAATGCTCCCATTGATGACTGGATCATGGAATCCATCAGTGG

AGACAGACTCACCCACCAGATCATGGATCTCAACCTGGACACTGTCTACTACTTCCGAATCCAAGCTCGG

AATGCCAAAGGAGTTGGACCTCTCTCTGATCCCATTTTCTTCCGGACACTCAAAGTGGAACACCCAGACA

AGATGGCAAATGACCAAGGTCGCCACGGGGATGGTTCCTTTTGGCCAGTGGACACCAACCTGATTGACAG

GAGCAGTCTGAATGAGCCTCCCATCGGGCAGATGCACCCTCCCCATGGCAGTGTCACCCCCCAGAAGAAC

AGCAACCTCCTGGTCATCCTGGTGGGCACAGTTGGGGTGATCACAGTGGTGGTGGTGGTGGTGGTGGCCG

TGGTCTGCACCCGGCGTTCCTCGGAGCAGCAGAGGAAGAAACGTGCAACCCACAGTGCTGGGAAAAGGAA

GGGTAGCCAGAAGGACCTGAGACCCCCAGATTTATGGATCCACCATGAGGAGATGGAAATGAAGAACATT

GAGAAACCCACAAACTTGGACCCTGCAGGAAGGGAATCACCAATGCAGAGCTGCCAGGACATCACCCCTG

GAAGCCACAGCCAGTCAGAAACACAACTGGGCACCAAAAGTGCCCCCCAAACTGGTCCTGAGACAGAAGA

GGTTGGGAGCAGCATGTCCACCCTGGAGCGCTCGCTGGCTGCCCGCAGAGCCACCCGGGCCAAGCTGATG

ATCCCCATGGATTCACAACCCAGCAACCCTCCTGTGGTCAGTGCCATCCCAGTGCCAACTCTAGAAAGTG

CCCAGTACCCTGGGATCCTGCCATCCCCCACCTGTGGGTACCCCCACCCACAGTTCACCCTCCGCCCGGT

GCCCTTCCCCACCCTCTCTGTGGACAGGACCTTTGGAGCAGGACGAAGTCATGAAGGGACAGCCCCCCAG

CAGCCCTCCTTGCTACCCCAGACCCAGCCTGAGCACCCCAACCACGAGGATGCTCCCAGCAGAACCATCC

CCACCGCCTGCGTCCGTCCCACCCACCCTCTCCGCAGCTTTGCCAACCCCTTGCTACCTCCTCCCATGAG

TGCAATAGAACCCAAGGTCCCTTACACACCACTTCTGTCTCAAACAGGGCCCAACCTGCCCAAGGCTCAG

GTTAAAACAGCATCCCTTGGCTTGGCAGGAAAAGCCAGGTCACCTCTGCTGCCTGTCTCCGTGCCCACAG

CCCCAGAGGCAACAGAAGATGGCCACAAGCAGCCAGAGGACTCCACCAATGTTTATGAGCAGGATGACCT

GAGTGAACAGATGGCCAGTTTGGAGGGGCTAATGAAGCAACTCAATGCTATCACAGGCTCAGCCTTTTAA

>Branchiostoma floridae (Amphioxus) DCC/NEO1

ATGGCGAGTCCGCGGCGTGGGGAGGGCGTGCGATGGACTTTCTCAACCGCGATCTGTGTACTTCTGGCCG

TCTCTGCGTTAGGCGATGTCAGACAATTTAGTGAATTCTACTTTGCAAACGAGCCACAGGACATGACGGT

AGAGCGTGATGTGCCCACAATGTTCAACTGTATGGCCATCGGGGATCCTAAACCAACCATTGAGTGGAAA

AAGGATGGCACTTTCCTGAACTTGTTTGGAGAGATGAGGAGATCAATACTGAACAATGGATCGTTGCGTT

TTAGCAACATCATTCATAATAAGAACGAAAAACCAGACGAGGGGCTCTATCAGTGTGTGGCGTCCGTGGA

TAGCTTAGGAACCATCGTTAGTAGGATGGCAAGACTTCAGGTCGCATCCCTGAGTAGATTTGAAAGAGAG

CCTCAGGCTACCTCAGTACACATGGAAGAGAACCTGAGACTGGAGTGTAGAATACAGGGCTCACCCAAGC

CTGACATCAGATGGCTCAAGGATGGACAAGACATAGAACATGACCTCTTTGAGGGAATCACGATACTGCC

GAGCGGTGCGCTGGAAATTGCGTATGTCCAGTTTACGGATGCAGGGAGATATAAATGTGTGGCAGTAAAC

GCAGCCAGGGAGAGAGAAAGTGCTGAAGTTGAAGTCTCAGTGCTTCCAGCTCTGGAACAAGTGAAGGAGC

CAGAGCTGATCGTACTGCCGGAGGACATGACTGTGTTAGAGGGAGAGACGGTCGTTCTGGAGTGTGTCGT

TAATGCCATGCCTGAGGCTGACGTCATTTGGAGCTTTGGTGGACAAGACATTGACACAGGCACCGATTCT

GTTGACAGATCCGGCAGCAGACATTCCATCCTTGGGGTCAGTAACCTGAGAATAGAGAACGCCCGCGAGT

CCGACAGTGGGATGTACCGCTGTACGGGAGAGAACGACCACGGCTCAGTCACTGCCTCTGCTCAGCTTAT

CGTAAACGTGCCTCCAAAGTTTATGACGACCCCAAGCAATACCTACGCCCACGAGCGCAGTGATATTGAG

TTTGAGTGTGATGTCTACGCAAAGCCAGCGCCACAGATCTCATGGATAAAGAACGGTGACACTGTCATCC

CCAGTGACTACTTCCAGATTCAGAATGGACAGAACCTGCGTATCCTTGGTCTGGTCAGGTCAGATGAGGG

CATGTATCAGTGTGTGGCGACAAACGAGTACGGAAGCATCCAGTCGTCAGCACAACTTATCATTGTCGAC

CCAGACGTGCCTCTTCCCACAATGCACTACTCATTAACGACTCCTACGCCGACCGCAGCAGCGGAAACGC

CTACTCCGCACCCCGTTCCGTCGGGGCCCAGGGATGTGGTCGCCGTGACAGTGTCCACTAGATTTGTGGC

CCTATCATGGCGGATGCCGGCTGAGACCCACGGAGAAATCGTGGCATGTTCTGTCTACTTCAAGCAAGAG

GGCTCGAATAGGGAAAGAGTTGTGAACACAACACGCTACAATCTAGAGGAGGTGGATGTCCGTGACCTTC

AACCCTCCACAAGCTACGTGTTCAGGGTCGTGGCATACAACGCTAACGGGCCCGGCGAGAGCTCGGACCC

GATCTACGTGCAGACGCAGCCAGAAGTCCACGTCCCTGGCCCGGCCACTTCACTCCAGGCTGAGTCTGTA

TCACCTGTTGCCATCACTGTATCATGGCAACCCCCCACACAGAGAAATGGGGTCATCCTAAACTATAAGC

TGTATTATGTTGAGATGGCGGCAGGGCCTCTTAATGAGGGTTCTGTGGATGTCAGCGGTCTGTCGCATAC

CCTGAATGGCCTGAAAAAGTACACAGAGTACAGCTTCCGTGTGGTGGCATACAACCAGCACGGGCCAGGC

ATGTCTACAGAAGAAGTCATCGTTAGGACTCTGTCAGACATTCCAAGCCAGCCACCAACGAATGCTACAT

TGGAACCACAAAGTTCCACTTCCATTCTTGTGAAATGGAACCCGCCTCCTCGTGACAGCCACAATGGCAT

CATCAGTAGCTACAAGATCCGCTACAAGAAGTCTGGAGCGCGTCGAGGAAGGGTTGTAGAGACAGGCGGA

AACATGAGACAATACCTTCTCAATGGCCTGGAGAAGGGATCTCAGTACAGCATCCGAATCTCTGCACAGA

CGGTAAATGGTAGCGGACCCCCCACAGAGTGGCATGTGGCAGAGACGCCAGAGAACGACTTGGACGAATC

TAAAGTCCCACAACAACCAAGCTCTCTCCATGTGCGACCTTTAACACACAGCATCGTGGTGTCATGGACG

CCACCACTTGACACGAACATCATGATCCGCGGTTACATCATTGGATACGGAAAAGGCTTCCCGGATGAGT

ACCGTGTGGTAGTGGACGGCAAGACGCGATACTTCACCATAGAGAACCTAGAAGCTGCATCCTTATATGT

TATCAAGGTGCGGGCGTACAACCAGCTTGGGGAGGGAATCCCCATCTACGAGAACACCTATACTCGACCC

AAAACTAGTAGGTCCTTTTTTTCTTCTCTCTTGCAAAGGTCTGCAGAGGTCAAGTCACCGCAGGCCGCCA

AGGTCACCTGGGCAGACACAACACTGACACGTAACCGGATCACGGATAATCGATACTACACTGTTCGATG

GATGTCACTTTTCCCTGAAAGCAAGTATTTCTATGCAAATGCAACCAGTCTGGAGTACATTGTGACAGAC

CTGAAGCCTTACACACGCTACGAGTTTGCTGTCAAGGTCACTAAAGGTCGTCAGGAAAGCGACTACAGCA

TGACTGTCACTAACAGGACTTATGAAGATAAGCCTAAGTCTCCTCCCAGAGACCTGACAGTTGTGGGAAT

CGAGGGGAACCCAGGAGGGGTAAACCTCAACTGGCAACCTCCTGCCAAGTCCAACGGACCGATCACAGGC

TACATCATTTTCTACACGACCGATCCGTTGCTGAACGATGCAGACTGGGTGTTTGACCCTATAATGGAGG

GCGACAAATTGACGTACACTGTAAAGCAACTCACTCCAAACACCAAATACTACTTCAAGATACAGGCCAG

GAATTCTATGGGCATTTCGCCCATCTCTCCCATCATGGAATACCAGACGCCGCCAGGTTGCGGGAATTCC

TATAATCCCAAGGAAGTTGGCAAAGGAGAGGAATCCAATTCTGGAGGAGCTGGAAGATCAACCAACAGCC

CTTCCAACCAATCATCTGGCGGCTTCACCTTGACTGACACCATGTTGTGGATCATTATCGCCTGTGTTGT

CACGGTGACGGTGACCATCATCTTCATCATCGTGACCTTCATCCTGTGCAGGCGACGCAACGGAGACAAC

AACAAGAACAAGAACAAGAAACGTGGGCAATACAAGGGTCGTGACAACGGCAAGAGGAAAGGGCCCCATA

AGGACGTGAAACCACCTGACCTGTGGATCCACCATGAGCAGATGGAGCTGAAGTCTATGGACAAGTCGAG

TCCCCAGCCTGGGGAGATGACAAACACACCAATCCCACGCAACTCTGCAGAGATGAAACCTGTGGACCAG

CAGCCAATGGACGGGCCTCAGATGGACAGGAGGAGAAACTCGTTTGTTGGTACAGATGAGGATGACCTGG

ATTACTCCTCCATCCCGCCGGTTCGACGACCTCCCTTCAAGCCCAAGCCCATCATGATCCCTGTGGACCA

CCAGCCACCCCCGCCCAGAGATCCTGCCAAAGTTCAGTCACCTGTGGGAACGTGGCCCCGCCCTAAAGAC

TTCTCCTTTGAACCGCCCCCTCCGCCCTCCCCCGCACCATCCATTGCTACCTCAGTGACCCTGAACCATC

CTGACCAGCGGCCGGTCTCCCCGTCCAGACCGCTCTACCCGCGTACGCAGTACTCCGCCAACATCCCGCG

CTCCGTCTCCGTGGAGCCCAGCCCCATGAATGATGTGGCAGACTACCCTCCCCCTCCCCTCAGTGATCAT

GAGATCCATACACCTGGATCTACTGTCAGCGGACACTCGTATCTGCACACTCCGCACACTCCGCACACGC

CATCAGAGAGGAGTTACACTCCTTCCTCGGAGGCGCGGTCGCTTGGCGCCCGTCCTGCCCATCCCCTGAA

GAGCTTCAGTGTGCCAGGCCCCCCTCCCCCACCTCCGTACAACAGCCTCCCCACTACACCTCAGTCCTCT

CACCTCTCTGAACCAAAACCAGGGCCCTCCAGTACCCTGTCTACTCCACTGAGCACGCCCAGTAAACCCC

AGACTCCAGCAGACATGTACCCAGATCCACAGACGTTACAGCCTCCCCCTCCACCACTGCCTGAGGCACC

GCCTGGGTCTGAGCATTCCTACGAGGACGATGATCTGACCACTGAGATGGCAAACTTGGAGGGGCTCATG

AGGGACCTGAACGCCATCACACAGTCTGAGCTGAACTGTTAG

>Brush turkey DCC

ATGNNNAACTTGCTCGTGGCGAACGTGAGCGACGACGATTCGGGGACGTACACCTGCGTGGTCACCTACA

AGAACGAGAACNNNNNGGGCCTGGTGAAATCGGACGAAGGATTCTATCAGTGCATTGCAGAAAATGATGT

TGGAAATGCACAGGCTGGNNNNAGCGTCCAGGCGTACACCGTCTTCTTCTGCAGGGAAGGCGTCAACAGN

NNGACCTACACCTTCCGCGTGGTGGCCTTCAACGAGTGGGGGCCGGGCGAGAGCTCGCAGCCCGTCAAGG

TGGCCACNNNNGCTGTGTCGACCTCGCCGACCTCCATCCTGGTGTCCTGGGACCCCCCCGCCTACGCCAG

CGGCCCCGTGCAAGGCTACAGGCTCTTCTGCACGGAGACNNNNGGCGACAGCATGTCGACGCTGGAGCGC

TCGCTGGCGGCCCGGCGAGCCACGCGCGCCAAGCTCATGATCCCGATGGATTCCNNNCTGGAGAGCGCCC

AGTACCCCGGGATCCTGCCGTCCCCCACCTGCGGGTACCCGCACCCCCAGTTCACCCTGCGG

>Brush turkey NEO1

ATGNNNNTTCCTACTTCTCCTCCCAAGGATGTGACTGTGGNNNGCAAAGAGGGGAAGCCTCGGACCATCA

TCGTTAACTGGCAGCCTCCGTCCGAAGCCAACGGCAAAATCACAGGCTACATCATTTACTACAGCACGGA

CGTGAACGCGGAGATACACGACTGGGTGATCGAGCCCGTGGTGGGGAACAGGCTGACGCACCAGATCCAG

GAGCTGACCCTCGACACGCCCTACTACTTCAAGATCCAGGCCCGCAACTCCAAGGGCATGGGGCCCATGT

CCGAGGCCGTGCAGTTCAGAACACCCAAAGGTAGGTNNNCTTCAGGAGCTGCGGGAAAAGGAAGCCGCCC

GCTGGACGTGGGGCCGGATTACAAACCCCCGCTGGGTGGCAGCAACAGTCCCCACGGAAGCCCTACTTCT

CCCTTGGATAGCAACATGCTCC

>Budgerigar ACAA2

ATGTTGATTACCAGGGACCCTGCCAAAAGGGAGGAAAACAAGTTCATTTACCTTGGCCAGCTTCTGTACA

TCTGGATAATTCTTGCTTGTCTTGCAGGTGTGTTCATTGTTGCAGCAAAGAGAACTCCTTTTGGGACCTA

TGGAGGTTTGTTGAAGGACTTCACAACCACTGACCTAGCAGAACATGCTGCTCGAGCTGCACTGGCTGCT

GGCAAGGTCCCTCCTGAGGTCATCAACAGTGTCGTCGTTGGCAGTGTCATGCAGAGCTCCGCAGATGCAG

TTTATATTGCAAGACATGTTGGTCTGCGTGTGGGGATTCCTGTCCCAGTTCCAGCCCTCACTGTCAACAG

ACTTTGTGGCTCTGGTTTCCAATCCATTGCCAATGGATGTCAGGAAATTTGCCTCAATGAATCAGAAGTT

GTTCTGTGTGGTGGAGCTGAAAATATGAGCCAAGCACCGTATGCAGTTCGAAACATTCGATTTGGAACCA

GATTAGGAGCAGACCTCAAGCTGGAAGACACATTGTGGGCAGCTCTAACAGATACACATGTTAAAATACC

CATGGCAATTACAGCTGAAAATCTGGCTGCAAAGTACAACATCACACGAGAGGACTGTGACCGATATGCT

TTCAAAACACAACAGAGATGCAAAGCTGCTCAGGATGCTGGTTACTTTAATGCTGAGATGGCACCAATTG

AAGTGAAAACCAAAAAGGGGAAAGAAAGTATGCAAAAGGACGAGCACCCCAAACCCCAGACCACTCTGGA

ACAATTGGCAAAACTCCCAACTGTCTTTAAAAAGGATGGAACAGTCACTGCTGGGAATGCTTCAGGGGTA

TGCGATGGAGCTGGTGCAGTCATCATTGCCAGTGAATCGGCACTTAAAAAGCACAGTCTTACTCCTCTGG

CAAGAATAGTAGCGTATCACTCATCTGGCTGTGATCCTTCCATAATGGGCATTGGTCCTGTACCTGCAAT

TACTGAGGTTCTGAAGAAAGCAGGATTGACCCTGAAGGACATGGATTTAGTCGAGGTGAATGAGGCATTT

GCACCGCAATACCTAGCTGTCGAAAAGGTTTTGGGCCTTGACCCTGAAAAAACCAATGTCAATGGAGGTG

CCATTGCGATAGGTCATCCTTTGGGAGCATCAGGCTCACGGATCACAGCTCATCTGGTTCATGAGTTGAG

GCGTCGTGGTGGGAAATATGCAGTTGGGTCAGCTTGCATTGGCGGTGGACAAGGTATTGCTATTATCATT

GAGAACACAGCCTGA

>Budgerigar C18ORF32

ATGGTGTGCATTCCCTGTATTGTCATTCCTGTTCTCCTCTGGGTCTACAAGAAATTCCTTGAGCCCTATA

TCTATCCTGTCATCGCACCATTCGTGAAGCGTGTATGGCCCAAGAAAGCTGTGCAAGAAACAACAGCCAC

AAAAGAAGGTCAAGGAGGCAGCACTGGGAATCCAAGGGCACCTTCAGCCATGAAAAGAGATCAGGAGGAT

GACTCTGGAATTTATAAATTTGAAAGTAATGGCGTTGCAAATGGAATCGCTGCAAAAAGATCCACAGAAG

TTTCCGACAAGAAAGCGGATTAG

>Budgerigar C18ORF54

ATGNNNTACAGCTCCGCATCCGAAGCCTTGGACGCCTACATTGAGGACTTCGACCGGAGCCAAGCAGATC

CCTATGGAAGCTGCATCCATGGGAGCGTTCCCAAGGAAATCCGACTTCCCAAGCACTGTGCCAAGGGAAA

ACACGGCCTGGAATCTTTCCCTTCAACAACAAGCTTGGAATTCTTCCCTTCCTTGTATGGGGGATCCCAG

GATCCCGACTCCATCAGCCTGACAACAGAGGAGCTTTTAGCCGTTCCCCCTGATGGATCCCAGCCTTTTC

CCCATCCCATTCCATCCAATCCTAGGAATCCAAGGGGAGAATCCCTAAAAACCTCCCCTCTGCGTTTGGG

AAGCGCTTGGAATTCTCATAGGAATCCAGGCAGGAAAACGCTGCCCAATGTCTCCAAAGGAAGCCCAAAC

CCTATGGAAAGGTTTTCCAGCACTTTTCCTTGTGGGAATGAACCCGGATGGCTCACAAACCTCAGTGGAT

CCAGGATGAGCGGCATTCCCACTTCTCCCTGTCCGATGTGGCTCCGGAATTCCAACCTCTTCCGTGACCC

AGCTCCGAATGGGATCAACCAAAGCCAAGCTTCCTCATCCCAGATCTGGAAAACAGGACCTCCCCATCCA

GACAATTCCCACTTTTCCCTACCTTTTTCCTTGCATTCAAGAGGTGATAACAGGGAAGGAAGCTGCTGCT

CTGACTTTACACATGGATGTATCCCAATGGATAACTCCTCTTCGGACCACGCTAAGGGAGAGCATCAGCT

CCTCACCTTGGAGGCCATTCCAGAGGCTTGGAGCAGGGATGGAATCAGTACGGAAAGGGGCAGTATTCCT

GCCACAACGGAGATCTTGGGATCATGGGAAGAGGCTCCGAGTGCTTCCGACCCCGTTGCATGTTGGGAGG

CTATGGATGCTCCTCTGGCATTCCCAAAGGCAAACCTGATCCCTAAATTCCTGGAAGACTGCTTGAAGGA

CAGCTACAAGG

>Budgerigar CFAP53

ATGTGTGAACCCCTTGAGGAAGAAGGGTTGAAGGCTACAAGAGCCAAGCCTCCTAAAGAGCAAAGAAATG

AAAATTTTGTCTTGGCCTGGAGACAGAAAGAAAAGGAACTCCTTGAAAATACTAAATTAATAAAGCTGTT

CGACCAGTACTGCAAGACCTGCAGGTGGCAGCAACAGAAAGAACAGAAGTGGCTGAACAGTGCTGTGCAG

AGAAAGGTTGATGTGGCAATGCAGGAGTACCTCGCTGGGGTTGATGAGAGGAGAATGAGGCTTTGTGAGC

TTCTGGAAGCAGAGGAAAGTAGGTACTATGCTGAAATGGAGGTGCTTGAAGAAACAGCACAGGAGAAACA

AGCAAAGATTAGGGAGAGAGCAAAATTACTGAAAGAGAAAAGAGAAGAAGAAAGACAGCAACTGGTGGCT

GAAAAACGAGAGCAGCAATTCAGAGAACAATCCGAAGAGCTTCGTACACTGTGGATGAAGAAGCATCAGA

AGGAAGTGTCTGAAGACCAGCTGGCTCAGGTAGCACTGAAGAAGGAGTTGGAAAAGCAGCAGAAGAAGGA

AGAGCAGATGCTTGAAGAGCTTTTGAAAGAGGATATGTTAGCAAAGGAAAAGCAAGAGGCATTGAAGGTG

CGGAAAATAGCAGAACAGAATCGGGAAACTCTGAATGCACTCAATGCCCAGGTAGCAGTGCTCAAGGCTC

ACAAAGAGGACGCAAAGCGGCTGAAGGAGGAAGAGGCTCGACTGCTGGAAGAAGAGCAGCAACTGCTTAA

ACTAGAAAATGAACAACTTCAGGTGAAGAAACTGCAGAAACAGAGGGAATGCAGGGATGTGTTGCTCAGT

GCAGCACAGGACAGGAAGAATCGTCTTAATGAAGAAAAACAAATTGAACTTGCCTTAGAGATGAAGATCT

TAGAAAAAGCTCTTCAGGACCCCCAGGAGAACATTGAGAAAGCAAGAAGAAAACAAGAGCTCTTAAAGGA

GCAGAAGGCTTACCTGGCACACCTGGCTCAACAGCTGGAGGAGGAGAGACAGCGACAAAAAGAAGAGGAC

AAGATCATTGATGAGGAGATAGTGGAGATTTGGGACAAAAAGGCTGAAAAATTGCGACTAGAAAAGGAGG

CTAGAAAGCAGCTCCTGAATGATGTCCTAAATACAAGACAACTGCAGATTGAGGAGAAGTTGCAGAGAAA

TGTAAAGAAGCACGAAGAACTCGCTCAGGAGAAGAAGTTACTAGCTGAAGCAATCACCGAACTCAAACAT

ACAGAAGAAGAAAAATATGCAAGAAAAATAAAAGAAGCAAAAGAATACCAAGAGCAACTCAGGGCTCAAA

TGGCCTACCGGCAACAGGCCCGTGATGCTGAGCAAGAAGAGAAGCAACAAGATTATGAATTGAACCTAGA

AGCAGAGAGAGCTTACCAAGAAAAGGTACAAGACATTCTATCAACACCTCGTGAGAAAGTAGCAAAAACC

CACCCTTTCAGAAGAAAACTAATGTCTTAA

>Budgerigar CTIF

ATGGAGAACTCATCGGTGGCATCGGCCTCCTCGGAGGCAGGGAGCAGTCGCTCTCAGGAGATCGAGGAGC

TGGAGCGTTTCATTGACAGCTATGTCCTGGAGTACCAGGTCCAGGGGCTGCTGACGGATAAAACAGAGGC

GGATGGCGAGAGTGAGAAGACACAGTCCCACGTCTCACAGTGGACTGCAGATTGTAACGAGCAGCTTGAT

GGCAGCTGTTCCCCATCCCGAGGGAAGGGCTCCTGTCAGCAAAATGGCAACAAGGACAGCAACCTTGACA

TGCTGGGCACAGACATCTGGGCTGCCAACACCTTTGACTCCTTCAGTGGTGCGACGTGGGACTTGCAGCC

TGAAAAACTAGATTTCACCCAATTTCACAGGAAGCTGCGAAACACCTCCAAACACCCGTTGCCTCACATA

GACAGAGAAGGGCTCGGAAAAGGAAAATACGAGGATGGAGACAGCATCAACTTGAACGACATAGAGAAAG

TCCTTCCAGTGTGGCAGGGCTACCACCCATTGCCTCATGAAGCTGAAATTGCACACACCAAAAAACTGTT

CAGAAGGAGGAGAAACGACCGGAGACGGCAGCAGAGACTTCCTGGTGGGAACAAGTCTCAGCAGCACGCA

GATCATCAGCAAGGTGGAACCAAACACAACAGGGACCACCAGAAACTCTACCAAGGAGGCCAGGCCCCTC

ACTCCTCAGGCAGGACGGGCCACCATGGCTACAGCCAGAACCGGAGATGGCATCACAACCAGAAACACTC

ACCCAACGACAAAGAAACGCACAGAAACGCCAAAGAGACTGAGAATTTGAAAATCGAGGACACCTCCGTC

TGCACCGTGCATATCCCTGTGGAGACACACCGAGGCCCGGAGGCTGTGGAGAAGCAGTCTCAGCAGTACA

ACCAGGAGTCAGAGACCAAGAGGAAAGACAGTGTTCATGAGCGCATTGGGGAAAGACCCAAGATCAATTT

GCTTCAGTCCTCCAAAGACAGGCTGCGGAGGAGGCTAAAAGAAAAGGACGAAGTCACGGTGGAAAACACC

GACCCTGAAAAGAACAAAATGGACAAATTAATTGAAATCCTCAACAGCATGAGGAACAACAGCAGTGATG

TTGACTCCAAGCTCACCACCTTCATGGAGGAGGCTCAGAACTCCACCAATTCTGAGGAGATGCTGGGGGA

GATAGTCAAGACCATCTACCAGAAAGCGGTGACGGACCGCAGCTTTGCTTCCACAGCAGCCAAGCTGTGT

GACAAAATGGCCCTTTTCATGGTGGAAGGAACCAAATTCCGGAGTCTGCTCCTCAACATGTTGCAGAAGG

ATTTCACCATGCGGGAGGAGTTGCAGCAGCGGGATGTGGAGCGCTGGCTGGGGTTCATCACCTTCCTCTG

CGAGGTCTTCGGCACCATGAGGAGCAGCACCGGAGAGCCCTTCCGAGTCCTTGTCTGCCCCATTTATACC

TGCCTCAGGGAGTTGTTGCAGTCTCAGGATGTGAAGGAAGATGCTGTGCTTTGCTGCTCCATGGAGCTGC

AGAGCAGTGGCCGTCTGCTGGAGGAGCAGCTGCCCGAGATGATGACGGAGCTGTTGGCGATAGCTCGTGA

CAAGATGCTGTGTCCCTCCGAGTCCATGCTGACGCGGTCCCTGCTGCTGGAGGTCATTGAGCTGCATGCC

AACAACTGGAACCCGCTGACTCCCACCATCACGCAGTACTACAACAAGACCATCCAAAAACTGACGGCTT

GA

>Budgerigar DCC

ATGGAGCATAGTCTTGGATGTGTTTGGGTACCAAAGCTGGTTTTTCTCCTCTTGGTGTTCTCGCTGGCGA

GCCTGCATCCTCGAGTTGACGGTTCACAGTTTAAGGCTTTTACAGCACTGAAATTCTTGACTGAGCCTTC

AGATGTTGTCACCATGCGTGGAAGCAACGTGCTTCTGAACTGTGTTGCAGAATCAGATCAAGGAGCCCCA

GTTATTAAATGGAAGAAAGATTCTGTCTTCTTAAACCTGGCAGTAGATGAAAGGAGACAGCAGCTGGCAA

ATGGATCACTTCTGATACAAAACATAGTCCACTCCAGACACCACAAGCCAGACGAAGGTCTCTACCAGTG

TGAAGCATCTCTAGAAGGCATTGGAGCTATCATCAGTCGGACAGCTAAGGTCATGGTAGCAGGACCGCTG

AGGTTTCTTTCCCAGACGGAATCTATCACGGCATTTGTAGGAGACACGATTCTGCTGAAGTGTGAAGTCA

TTGGGGAGCCCATGCCCGTGGTGCACTGGCAGCGAAACCAGGAGGACTTGTTCCTGAACTCAGCCGACAG

CCGGGTGGCTGTCCTGCCCTCTGGAGCTTTACAGATCAGCAGGGTCCAGCATGGGGACAGTGGGATCTAC

AGGTGCCTGGCGAAAAACCCAGCCAGCTCGAGAACTGGAAACGATGCAGAAGTCAGGGTTTTGGCAGATC

CAGGTTTGCACAGGCAGCAGTTTTTCCTGCAGCGACCGTCAAATGTGGTGGCCATGGAAGGGAAGGATGC

TGTTTTGGAGTGCTGCGTTTCTGGGTACCCGCCTCCCACCTTTACATGGCTGCGAGGAGATGAAGTGCTC

CCCATCAGGTCCAAAAAGTATTCCTTGCTGGCTGGGAGTAACCTCCTTATATCAAATGTGACTGATGATG

ATTCTGGAACATACACATGCGTAGTCACCTACAAAAATGAGAACAGCAGCGGCTCTGCAGAGCTGTCAGT

GATGGTTCCACCGTGGTTTTTAGTTCACCCTTCAAATATTTATGCCTACGAGAGTATGGATATTGAGTTT

GAATGTACTGTGTCTGGTAAGCCTGTTCCTATGGTGGAGTGGATCAAGAATGGAGAAGTGGTCATTCCCA

GCGACTATTTTCAGATAGTGGGTGGCAGCAACTTAAGGATTCTGGGCTTGGTAAAGTCAGATGAAGGTTT

TTATCAGTGTGTAGCTGAAAATGAAGCTGGAAACGCACAGGCCAGTGCACAGCTAATCATCCCAGAGCCT

GCTGCTCCAAGTTCCTTGTCCTCCCCTCCTGCTCCCCGAGATGTGGTCCCTGTTTTGGTCTCCAGCCGAT

TTGTCCGTCTCAGCTGGCGCCCACCCGCTGAAGCCAGAGGCAGTGTCCAGACATACACGGTCTTCTTCTC

CAGGGATGGCGTTAACAGGGAACGGGCAGTCAACACGTCTCAGCCTGGAACACTTCAACTCACTGTGGGC

AACCTGAAACCAGAGGAAACCTACACCTTCCGAGTGGTGGCGTACAACGAGTGGGGACCTGGAGAGAGCT

CGCAGCCCATCAAGGTCGCCACGCAGCCAGAGTTGCAAGTTCCTGGTCCGGTGGAAAACCTGCGGGCTGT

GTCTACTTCACCGACCTCAATTCTCGTCTCCTGGGATCCTCCTGCCTATGCCAATGGCCCTGTCCAAGGC

TACAGACTCTTCTGTACAGAGACAGCGACTGGAAGAGAGCAGCACGTGGAAGTGGATGGGCTCTCATACC

GGCTGGAAGGGCTGAAGAAGTTCACTGAGTACACCCTACGCTTCCTCGCCTACAACCGCTACGGCCCCGG

CGTCTCCACTGAGGATGTGACAGTCACCACGCTTTCAGATGTGCCCAGTGCGATGCCTCAGAACGTTTCC

TTGGAAGTGGTGAACTCCAGGAGCATCAAAGTTAGCTGGTTGCCTCCACCACCAGGTACTCAAAATGGAT

TTATTACGGGCTATAAAATCCGGCATAGAAAGACTACCCGCAGGGGTGAGATTGAAACACTGGAGCCAAA

CAACCTCTGGTACTTGTTCACAGGACTTGAGAAAGGAAGCCAGTACAGTTTCCAGGTGGCTGCAATGACA

GTGAATGGGACAGGTCCCTCTTCAGACTGGTACACAGCAGAAACACCCGAAAACGATCTCGATGAATCTC

AGGTTCCTGACCAGCCAAGTTCTCTTCATGTCAGGCCCCTGACAACCAGTATTGTCATGAGTTGGACTCC

GCCATTGAACCCGAACATTGTCGTCCGTGGGTACATCATTGGCTATGGTGTAGGCAGTCCGTATGCTGAG

ACTGTGCGGGTGGACAGTAAACAGCGGTATTATTCCATTGAAAACTTGGAGCCAAGTTCCCACTATGTGA

TTTCCTTGAAGGCCTTTAACAATGCAGGAGAAGGGGTGCCTCTGTATGAAAGTGCGACCACCAGGTCAAT

GACAGACCCCATTGATCCATTAGAAGTTGATTTTTATCCTTTGCTTGATGATTTCCCTACCTCAGTCCCA

GATATCTCCACCCCCATGCTCCCACCAGTAGGTGTCCAGGCTGTTGTACTTACGCATGATGCAGTGAGGG

TCATCTGGGCAGATAACTCTGTCCCAAAGAATCAAAAGACAACGGAGGTTCGCTTCTACACGGTCCGATG

GAGAACAAGCTATTCAACAAATGCTAAGTATAAGTCGGCAGATACGACTGCTCTGAGTCACACTGTGATC

GGCCTGAAGCCGAACACCATGTACGAGTTCTCTGTCATGGTCACCAAAGGCCGGCGGTCCAGCACCTGGA

GCATGACAGCACACGCCACCACCTACGAAGCAGCTCCAACCTCTGCTCCCAAGGATTTGACAGTCATTAC

ACGGGAAGGGAAACCACGGACTGTCATTGTCAGCTGGCAGCCACCGTTGGAAGCCAATGGAAAAATCACT

GCTTACATCCTCTTCTATACTTTGGACAAGAACGCTCCCATTGATGACTGGATTATGGAGTCCATTAGCG

GTGACCGGCTCACCCACCAGATCATGGATCTCAACCTGGACACCGTGTACTACTTCAGAATCCAAGCTCG

CAACGCCAAGGGAGTGGGGCCCCTCTCTGATCCTACTTTCTTCCGGACGCTGAAAGTGGAGCACCCTGAC

AAAATGGCTAATGACCAAGGTCGTCATGGGGATGGTTCCTATTGGCCAGTGGACACCAACCTGATTGATC

GGAGCAGTCTGAATGAGCCCCCCATTGGGCAGATGCACCCTCCCCACGGCAGCGTCACACCCCAGAAGAA

CAGCAACCTCCTTGTCATCATCGTCGTCACCGTCGGCGTCATCACGGTGGTGGTGGTGGTGGTGGTGGCC

GTCATCTGCACCAGGCGCTCCTCAGCACAGCAGAGGAAGAAACGTGCAACCCACAGTGCTGGTAAGAGGA

AGGGCAGCCAGAAGGACCTGAGACCCCCAGATCTGTGGATACACCACGAGGAGATGGAAATGAAGAACAT

CGAGAAGCCAGCGGGCTCAGACCCTGCAGGAAGGGACTCACCAATGCAGAGCTGCCAGGACATCACCCCT

GTTAGCCACAGCCAGTCGGAAACACAACTGGGAAACAAGAACAACACACAGCCTGGTCCTGAGACAGAAG

ATGTTGGAAGTAGCATGTCCACATTGGAGCGCTCACTTGCTGCCCGCAGAGCCACCCGTGCCAAGCTCAT

GATCCCCATGGATTCACAACCAAACAACCCTTCTGTGGTCAGTGCCATTCCGGTGCCAACACTAGAAAGT

GCCCAGTACCCTGGAATCCTGCCATCCCCAACCTGTGGATACCCACACCCGCAATTCACCCTTCGTCCAG

TGCCATTCCCAACCCTCTCCATGGACAGGACCTTTGGACCAGGAAGAACTGTTAACGAAGGACCAGCACC

ACAGCAGCCGCCCTTGCTGCCGCAGACACAGCCTGAGCACTCCAACAATGAGGATGCCCCGAGCAGAACC

ATCCCCACCGCTTGTGTCCGCCCTACACATCCTCTCCGCAGCTTCGCCAACCCCTTGCTACCTCCACCCA

TGAGTGCAATAGAACCGAAAGTCCCTTACACACCACTCCTGTCTCAAACAGGGCCTAACCTCCCCAAGGC

TCAAGTCAAAACTGCATCCCTTGGCTTGGCAGGAAAAGCCAGGTCACCTCTGCTGCCCGTCTCAGTGCCC

ACAGCACCGGAGGTTGCAGAAGAGGGACACAAGCAGACTGAGGACTCCGCAAATGTTTATGAGCAGGATG

ATCTGAGTGAACAGATGGCCAGTTTGGAGGGGCTAATGAAGCAACTCAATGCTATCACAGGCTCAGCCTT

CTAA

>Budgerigar DYM

ATGGGAGCAAATAGCAGCAGCATCAGTGATCTTCCAGAAAATGAGTACTTAAAAAAGTTATCAGGAGCAG

AGCCCATCTCTGAGAATGACCCGTTCTGGAATCAGCTGCTGTCTTTTAGCTTTACCACTCCAACAAACAG

TGCTGACTTAAAGCTCTTGGAAGAAGCCACAATCTCAGTCTGCAAGTCTTTAGTTGAGAAGAATCCTCGA

ACAGGAAACCTTGGGTCATTGATTAAAGTCTTTCTTTCTAGAACCAAAGAGTTAAAAATTTCAGCAGAAT

GTCAGAATCACCTCTTTATTTGGCAGGCTCACAATGCACTGTTTATTATTTGCTGTTTGCTGAAAGTATT

CATCAGTCGAATGTCAGAGGAGGAGCTGCAACTTCATTTTACTTACGAAGACAAAGCACCGGGCTCATAC

GGAATAGAGTGTGAAGACCTCATAGAAGAATTGCTGTGTTGCCTTGTCCAGCTCATTGTTGAAATTCCTC

TCTTAGATATTACATACAGCATTTCTTTGGAAGCTGTGACAACTCTCATCGTCTTCCTCTCCTGCCAGTT

ATTCCGCAAAGAAATCCTGCGGGAGAGCATCATCCACAAATACCTGATGCATGGCCGATGTCTCCCATAT

ACCAGCAGACTTGTGAAAACTTTGCTATATAATTTCATTAGACAAGAAAGAAGCCCTCCTCCAGGGACCC

ATGTCTTTCAGCAGCAAAGTGATGGTGGAGGACTGCTTTATGGAATTGCATCTGGGGTGGCAACTGGCCT

GTGGACAGTCTTCACACTCGGTGGTGTGGGCAGCAAACCAGCACCACAGCTGGAGCAGTGCTCCCCTCTG

GCTAACCAGAGCCTACTGCTGCTGCTGATCTTGGCCAATCTCACCGATGCTCCAGATACGCCGAATCCCT

ACAGGCAAGCTATTATGTCCTTCAAAAACACCCAAGATAGCACTGCTTTTTCATCATCAAATCCACACGC

TTTCCAGATTAACTTTAACAGTTTATACACAGCTTTGTGTGATCAGCAGAAATCTGATCAAGCGACTCTT

CTTTTATACATGCTTCTGCATCAGAATGGCAATGTACGGACGTATGTGTTGGCACGAACGGACATAGAAA

ATCTTGTTCTGCCAATTCTTGAAATTCTCTATCATGTTGAAGAAAGGAATTCACACCATGTTTACATGGC

TCTTATCATTCTGCTGATCCTTACAGAGGATGATGGCTTCAACCGATCCATCCATGAAGTGATATTGAAA

AATATCACCTGGTATGCTGAGCGTGTTTTAACAGAGATCTCACTTGGGAGTCTCCTGATATTAGTCGTGA

TAAGAACCATCCAGTACAACATGACACGGACAAGGGACAAATACCTTCATACAAATTGTCTGGCAGCCTT

AGCAAATATGTCAGCACAGTTCCGCTCACTTCATCAGTATGCGGCTCAGAGGATCATCAGTTTATTTTCT

TTGTTGTCTAAAAAACACAACAAAGTGCTGGAACAAGCCACGCAGTCCTTGAGAGGTTCCGTTGGTTCAA

ATAACTCTCCACTTCCTGATTATGCACAAGACCTGAATGTGATCGAGGAAGTGATCCGAATGATGTTGGA

GATCATCAACTCCTGCCTGACAAATTCCCTTCATCACAACCCAAACTTGGTGTACGCGCTGCTTTACAAG

CGGGATCTCTTTGAGCAGTTCCGAACTCACCCTTCCTTCCAGGACATAATGCAAAATATAGATCTGGTGA

TCAGCTTTTTCAGCTCCCGATTAGAGCAAGCTGGAGCTGAGCTGTCAGTGGAGCGAGTTCTGGAAATCAT

CAAGCAAGGAGCTGTTGCTTTGCCCAAAGACAGGCTAAGAAAGTTCCCCGAGCTGAAGTTCAAGTACGTG

GAGGAGGAGCAGCCCGAGGAGTTCTTCATCCCCTACGTTTGGTCCTTGGTCTACAACTCCGCCGTGGCCC

TGTACTGGAACCCGCATGACATCCAGCTCTTCACCATGGACTCTGGCTGA

>Budgerigar ELAC1

ATGTCAATGGATATAACTTTCCTCGGCACTGGCTCGGCATATCCCTCTCCAACCAGAGGAGCGTCGGCAC

TGGTGCTTCGCAGGGAAGGAGAGTGCTGGCTGTTCGACTGCGGGGAGGGAACTCAAACACAGTTCATGAA

GAGCCATCTCCGAGCAGGCAGAATTACCAAGATCTTCATCACTCATCTTCACGGTGACCACTTTTTTGGA

CTTCCTGGCCTGCTGTGTACAATTAGCCTCCAAAGCAGCCCTGATGCAGACAAACTACCTCTGGATATTT

ATGGACCATTAGGGCTGCGAGACTTCATCTGGAGAAGTATGGAGCTCTCCCACTCCCAACTTCTCTTTCC

CTACACTGTTCATGAACTGGTGCCTACACGGGACCAGTGCCCTGCAGAAGAATTCAAGGAGTTCTCTTAC

ATGGGCAGTGATGAGATACGTCCTGATGGAGCACAAGGGAGAATACTCCATCTGGATCCAGTAGAAGACT

CTTACTTGCTGCTTGAGGATGAGCAGCTAGTTCTGAAAGCATTTCGCCTATTTCACCGCATTCCTTCCTT

TGGCTTTGTGGTGGAAGAGAAGCCCCGGACTGGTAAACTCAATGTACAGAAACTGAAAGACCTTGGAGTT

CAACCAGGTCCTTTATATGGGAAGCTGAAGAATGGGAGTACAGTTGTTCTAGAAAATGGACTAACAATTT

CTCCTTCTGATGTCTTAGAAGACCCTATTCCTGGAAGAAAAATTTGCATTCTGGGGGATTGTTCAGGGGT

GGTTGGAGATGCGGCCGCAAAGCTTTGCTGTGGAGCAGATGTACTGATACACGAAGCCACGTTGGATGAT

ACACAAGAGGAAAAGGCCAGAGAACATGGTCATAGCACTCCAAAAATGGCTTCAGAGTTTGCAAAATCGT

GTAAAGTTAAGAAAGTGGTGTTGACTCACTTCAGTCAGCGGTACAAACCGGCTGCACAGAGAGGTGAGGG

GGATGCGGACATCACCGAACTGAAGAGACAGGCAGAGTCAGTGCTAGATGGCCAAGAAGTAACACTAGCA

GAGGATTTCATGACAATAGAAATTCCAATGAGAAAGATAAAATGA

>Budgerigar FECH

ATGNNNNTCGCCCCCTCCCGGTCCCGGCTGCGCAGCGCCAGGGTCCAGCAGCAGTACCAGCGCATCGGGG

GGGGCTCCCCCATCCGCCATTGGACTGAGGTGCAGGGAGAGGCTGTGGTGGAGCTGCTGGACAGGATGTG

TCCTCNCATAGCGCCCCATAAGTACTACATAGGCTTCCGCTATGCCCACCCCCTAACCGAGGAGGCCATA

GGGGCCATGGAGCAGGACGGCATCGAACGCGCCATTGCCTTCTCCCAGTATCCCCAGTACAGCTGCTCCA

CCATAGGCAGCAGCCTCAACGCCATCTATAGGTACTATAGGGACAATGGGATCCAGCCCAGGATGAAGTG

GAGCATCATTGACCGCTGGCCAACACATCCCCTATTGCTGCAGTGCNNGGGTGATCCCTACCCCCAAGAG

GTTGGGGCCACAGTCCAGGGGGTGATGGAGAAGCTCAAGTACAGCAACCCCTATAGGCTGGTGTGGCAGA

GCAAGGTNNNGCTACTGGTCCCAGTTGCCTTCACCAGTGACCACATTGANACCCTCTATGAGCTGGACAT

TGAGTATGGGCAGAACTTGGCCAAGGAGGTGAGG

>Budgerigar LIPG

ATGCAGGGGGTCGCCCTCCTGCTCTGCGCCGCCGTCATCTGCTGCACCGCGGCGCCGTCTGCGCTGCCGG

CCCGAGATGCCACCGTCGCCGAGCAGCCTGCGCCGACGGCAGCCAAGCCGCGGGTGAAGTTCTTGCTCCG

CTCCGCGTCGCACGCCGAGGAGGAGGGTTGCGCGATCGCCGTCGGGCAGAGCAAGTGCCTGGAGGACTGC

AGGTTCAACGTGACAGCCACGACCTTCTTCATCATCCACGGGTGGACCATGAGTGGCATGTTCGAAACCT

GGCTGGGCAACTTGGTATCCGCTCTCCAGAACAGGGAGAAGGATGCCAACGTGGTGGTGGTGGATTGGCT

TTCACTCGCCCACCAGCTCTACACCGATGCCGTGAACAACACGCAGATTGTTGGAAAAAGCATAGCAAGG

CTGCTTGACTGGTTACAGGAGAACCCGCTCTTCCAGCTCGAGAATGTCCACCTGATTGGGTACAGCCTGG

GTGCTCACGTCGCTGGCTTTGCTGGTAACCATGTCCATGGGACTATAGGCAGGATTACAGGTTTGGATCC

TGCTGGCCCTATGTTTGAAGGAGTGGACCCTAGCAAGCGCCTCTCCCCTGATGATGCTAACTTTGTGGAT

GTCCTTCACACCTACACAAGGGAAACACTAGGTGTTAGCATTGGGATCCAGATGCCTGTAGGCCATGTTG

ACATCTACCCCAATGGGGGAGACTTCCAGCCTGGCTGTGGTTTAAGTGACGTCTTGGGAGCAATTGCCTA

TGGGACGATCGGTGAAGTGGTTAAATGTGAGCATGAGCGGTCTGTGCACCTCTTCGTGGACTCCCTCGTG

AACCAGGACAAACAGAGCTTCGCATTTCAATGCACCGACTCCAGCCGCTTCAAGAAGGGCATCTGCCTGA

GCTGCCGGAAGAACCGCTGCAACGGCATCGGCTACAATGCCAGGAAAACACGGAACAAAAGGAACAGCAA

GATGTACTTGAAAACAAGAGCTGACATGCCCTTCAAAGTCTATCATTATCAGATGAAAATGCATGTCTTC

AGCTACCAGGGCTTGGGAGAGGCTGATCCCACTTTCTCTGTCACCCTTTATGGCACCAATGGAGACTCTG

AACCTCTCTCTTTAGAAATGCTTGATCAAATTGGTCTAAACGCTACTAACACCTTCCTGGTGTACACTGA

AAAGGACATGGGTGAACTTCTGAAAATAAAGCTCACCTGGGAGGGAACATCTCAGTCATGGTATGACCTA

TGGAAAGAGCTGAAGAGCTACTGGTATCGGCCTGCAAAGCTTTCCCAGGAGCTGCATATCCGACGTATCC

GTGTGAAATCTGGGGAGACGCAACAGAGGTTTGCTTTCTGTGTGGAGGATGCCCAGCTAACCAGTATAAC

TCCTGGTAAGGAGCTCTGGTTTGTGAAGTGCACAGAAGAATGGCAAAAAAGATCTGTCTCAAATGTGCTC

TGA

>Budgerigar MAPK4

ATGGCAGAGAAGTGCGACTGCATCGCCAGCATGTACGGATATGACCTGGGCTGTCGCTTCATTAACTTCC

GACCCTTAGGCTTCGGGGCCAATGGGCTGGTGCTGTCAGCCCTCGACAGCAAGAGCTGCCGCAAAGTGGC

AGTGAAAAAGATCACCATCAGCGACGCGCGCAGCATGAAGCACGCCTTCCGTGAGATCAAGATCATCCGC

CGCCTGGACCACGACAACATCGTGAAGGTGTACGAGGTGCTGGGCCCCAAGGGTGCCTGCCTGCGTGGGG

ATTTCTTCAAGTTCAACATGGTGTACATCGTCCAGGAGTACATGGAGACAGACCTGGCGCGCCTGCTGGA

GCAGGGGAAGCTTGCCGAGGAGCATGCCAAGCTCTTCATGTACCAGCTGCTGCGAGGGCTCAAGTACATC

CACTCGGCCAACGTCCTCCACCGCGACCTCAAGCCGGCCAATATTTTCATCAGCACAGAGGACCTGGTGC

TGAAGATCGGAGACTTCGGGCTGGCCAGGATTGTGGATCAGCATTACTCACACAAGGGTTACCTTTCTGA

AGGCTTAGTGACAAAATGGTACCGCTCCCCCAGGCTCCTCCTCTCGCCAAACAACTACACCAAAGCCATC

GACATGTGGGCAGCTGGCTGCATTCTTGCTGAGATGCTGACGGGAAGGATGCTCTTTGCTGGGGGTCATG

AGCTGGAACAGATGCAACTTATTCTGGAGACAATTCCAGTTATCCATGAGGAGGACAAAGAGGAGCTGCT

CAAAGTGATGCCCACGTTCATCAACAGCACCTGGGAAGTGAAGAAGCCACTGCGTAAGCTGCTCCCCGAA

GTGGACAGTGAAGCCATTGATTTTCTGGAGAAAATACTGACATTTAACCCTATGGATCGATTAACGGCTG

AGATGGGTCTGCAGCATCCTTACATGAGTCAGTATTCCTGTCCCGAGGATGAACCAGTGTCTCAGCATCC

ATTCCGGATTGAGGATGAGATTGATGATATTTTACTGATGGAAGCCAGCCAGAGCCAGATGTCTAACTGG

GACAGGTGTCCCAGCCAGTATCATGTAAGCCTCTCCTCTGACCTGGAATGGAGACACGATAAATATCATG

ACATGGATGATGTTCAGCGGGACCCTCGGGCAGGGTCTGAATCCATCGCTGAGGAAGCACAAGTTGATCC

ACGGAAATACTCACAAAGCAGCTCAGAGAGGTTCTTGGAGCTCTCCCACTCATCCATGGACCGAGTATTT

GATGCTGATTGTGGAAAATCATGTGATTACAAAGTGGGGTCACCTTCCTACTTGGACAAATTGCTGTGGA

GAGACAATAAGCCCCATCACTACTCAGAACCCAAATTGATTTTAGATTTATCCCACTGGAAAAGAGCAGC

CATAGCACCCACAGCTGAGCTGTCACTAGAAGAAGAACCATCCAACCTCTTCCTGGAGATTGCTCAGTGG

GTGAAGAGCACGCAGGTGGGTCTTGAGTGTCCCAGTCCACTTCCAGAGATTCAGGAACGGAGCCTGCCAT

CTTCTCCTCACCACCTCCACAAAGAACCCACAGAGGTGAACAGCAAAAAAGACTCTGAGTTTGATTTGGA

CGTCTTCATCTCCAGGGCACTGAAACTTTGCAAAAAACCTGAAGATCTTCCAGACAGCAAGCTTAATGAC

ATCAATGGGGCCTGCATATCTGAGCACCCCAATGAGATTGTACAAACAGAGGTGTATCAGAAAGACCGAT

GGTGA

>Budgerigar MBD2

ATGGAGCGGCCGGGCCGCAGTGAGTGCCCCGCTCTGCCCCCGGGCTGGAGGAAGGAGGAAGTGACCCGGA

AGTCAGGGATCAGCGCCGGGAAGTGCGATGTCTACTACTNNNNCCCCAGTGGGAAGAAGTTCCGGAGCAA

GCCGCAGCTGGCGCGATACCTTGGGAATGCCGTGGATCTGACCGGCTTCGACTTCCGGACTGGGAAGATG

CTTCCCAGTAAAGTCCAGAGGAGCAGGCAAAGAGGGAGGAATGAGGCCGTCATTCCCAAGGTTGGAAAAC

CGGATCTCAACACCACCCTTCCCATCCGTCAAACAGCCTCCATCTTCAAGCAACCCGTTACCAAGGTAAC

CAATCACCCTAGCAACAAGGTGAGGGCCGACCCCCAGCGAGTCATGGACCAACCGCGTCAGCTTTTCTGG

GAGAAGAGGCTCCAAGGGCTCAGCGCTTCCGACGTCAGCGAGGAAATCCTGCGGGCGATGGAGCTTCCCA

AAGGCCTCCAAGNNNNGAAGCAGGAGGAGCGGGTGCAGGAGGTGAGGAAGAAGCTGGAAGAGGCTCTCAT

GGCTGATGTCCTATCCCGAGCCGTGGACATGGCGAAGGATGGGGACATGGATGGGGATCATGGAGCCGAA

GCATAG

>Budgerigar ME2

ATGTTTTCCCGACTGAGAGCCGCTACCACTCCCTGTGCGATGGCACGTTGCAGTGTGCATACGAAAGAGA

AGGGCAAGCCACTTATGTTAAACCCAAGAACAAACAAGGGTATGGCCTTCACATTACATGAACGACAAAT

GCTTGGGCTGCAAGGACTTCTACCTCCTAAAATAGAGACACAAGACATTCAAGCCTTACGCTTCCATAAG

AATTTGGCAAAAATGACTGATCCCTTGGAAAAGTATATCTATATAATGGGAATCCAAGAGAGAAATGAAA

AATTATTCTATAGGGTATTACAAGATGATATCGAGCGGTTAATGCCAATTGTATACACTCCAACAGTGGG

CCTTGCCTGCTCCCAGTACGGCCACATCTTCAGGAGACCAAAAGGATTATTTATTTCTATCTCAGACAGA

GGCCATATAAGGTCAATTGTGAACAACTGGCCAGAGAATGACGTTAAGGCTGTTGTTGTAACTGATGGAG

AAAGAATATTGGGTCTCGGAGATCTAGGTGTGTATGGGATGGGAATACCAGTAGGAAAACTGTGTTTATA

TACAGCCTGTGCAGGAATAAATCCAGATAAATGCTTGCCTGTGTGCATCGACGTTGGAACTGATAATACA

ACGCTCTTAAAAGACCCATTTTACATGGGACTATACCAAAAAAGGGATCGCTCTCAGGTCTATGACGACC

TAATTGATGAATTTATGGAAGCCATTACAGACAGGTATGGCCAGAACACACTTATCCAGTTTGAAGACTT

TGGAAACCACAACGCTTTTCGTTTTTTAAGAAAATACAGAGAGAAATATTGTACCTTCAATGATGATATT

CAAGGGACAGCTTCAGTGGCTTTGGCAGGACTGCTGGCAGCACAGAAAGCCACGGGTAAACCACTTGCAG

AGCAGAAAGTGCTGTTCCTTGGGGCAGGAGAGGCTGCGCTGGGAATTGCAAACCTCATTGTTATGGCTAT

GATGGAAAGTGGTGTTTCTGCTGAGGAAGCCTATAGGAGAATATGGATGTTTGACAAATACGGTTTACTG

GTTCAGGGCCGAGAACAAAAGGTTGATGCTAATCAAGAACCATTTACGCATCAGGTTCCAGAGCAGATAC

CAAAGACATTTGTGGAGGCAGTGAATGTACTTCGGCCTTCAGCTATCATTGGAGTTGCAGGAGCTGGGCG

GCTCTTCTCTCAGGATGTGATCAAAGCAATGGCCTCTATCAATGAGCGACCCATAATATTTGCACTAAGT

AACCCCACAGTGAAATCTGAGTGCACAGCAGAGGAAGCATATACATTAACAGAGGGCCGTTGCTTATTTG

CCAGTGGCAGTCCCTTTGAGCTGGTGACTCTGAAAGATGGAAGAAGCTTCAAACCAGGCCAAGGAAACAA

TGCTTATATTTTTCCAGGTGTGGCTCTTGCTGTGATCCTCAGCAGCGTTCGACATATTAGCGATAAGGTT

TTCCTAGAAGCTGCTAAGGCATTGTCGGAACAGTTGACTGATGAAGAACTTGCACAGGGAAGACTCTATC

CTCCACTGTCTAATATCAGGGAAGTTTCTATTTATATTGCTGTCAAGGTTATGGAATTTTTGTACGCAAA

CAACATGGCTTTCCATTACCCTGAACCTGCAGACAAGAACAGTTACATTCGATCAAAGGTTTGGTCCTAC

GAATACGAATCCTTCATGCCAGATGTGTATGACTGGCCTGAATCTAAGGTTCACTGA

>Budgerigar MEX3C

ATGTGGCCACAGGACCCAGCATGGAGCAGTCAGTACCTTGTGCTCTGCCTCCTGAGCCAGCTGGTTCTGG

AGCTGCTGGAGCTGCTCTGTGGAGTCACTGCACAGCTCCTGCCCACCGAGCTGCCGCCGCTGCCTCCTCC

CCGTCCGCCGCCGTCGCCGTTGCTGGTGCTCCAGCAAAGCTTCGCCGGCCTCGGCCTCCGCGGCCAAGGA

GGGGTAGGAGCGGCCCAGAGGCGAGCCCGGCTGCGGGAGGCAGGCCTGGCTCCGGCCGATACCCCCCTGG

AGCCGGGGCCTCGAGGGTCAGAGGAGGAGGAGGAGGAGGAGGCCGGGGACGAGGCGGACCTGGATCTGGA

GCTGGACGAGGAGGAACTGCTGGCGGGGGAGGACGCGCCGGAGGAGGACCAGGACACGGCGGCCGTGCTG

GTGCTGTCGCCCTCCCAGCCGCTGTCGCTGCTGCCGCCGCTGGGCTCCGTGCTCCTGTCGCCTTCCTTGG

ACGTGCGGGAGCCGGCGGCTGCGGGAGCGTTCCGCGGGGGGCACGATCCTCCGGGCATGATGGCGGCGAT

GCTGTCCCGTGCCTACGGCGGCGGCCTGGGCAGCGGGGCCGCGGGCCTCAGCAGCGAGCAGGCGGCTCTG

CTCCGCCGGAGGAGCGTCAACACCACCGAGTGTGTGCCCGTGCCCAGCTCGGAGCATGTGGCCGAGATCG

TGGGGAGACAGGGTTGCAAAATAAAAGCACTAAGGGCCAAGACAAATACTTACATTAAGACCCCTGTTCG

TGGAGAAGAACCCATCTTTGTTGTCACTGGACGAAAAGAGGATGTAGCCATGGCCAAAAGGGAAATTCTC

TCAGCTGCCGAACACTTCTCCATGATCAGAGCATCACGCAACAAGAACGGTCCTGTCCTGGCAGGTTTGC

CATGTACCCCCAACCTGCCAGGTCAGACAACTGTCCAAGTCAGGGTGCCTTACCGTGTAGTTGGGCTGGT

GGTTGGTCCAAAAGGAGCTACAATCAAAAGAATTCAGCAGCAGACCCATACCTACATAGTCACTCCCAAC

AGAGACAAGGAGCCTGTCTTTGAAGTCACGGGGATGCCTGAAAATGTGGACCGTGCACGTGAGGAGATAG

AGATGCACATAGCGATGCGCACCGGGAACTACATCGAGCTGAGCGAGGAGAATGACTTCCACTACAATGG

TACAGATGTGAGCTTCGAAGGAGGCACCCTCAGCTCAGCATGGATCCCCTCTAACCCCGTCCCACGTAGC

CGTACCAGAATGATCTCTAATTACAGAAATGACAGCTCCAGCTCCTTGGGAAGTGGTTCCACAGATTCCT

ATTTTGGAAGCAATAGATTGGCTGACTTCAGCCCAACAAGTCCCTTCAGCACAGGTAACTTCTGGTTTGG

AGAAGCGCTGCCTTCAGTTGGCACGGAAGATCTTGTGGCCAACTCTGCCACGTATGACTCCTTGCCAACG

CCTTGCCAAACCATCTGGACTCCTTTTGAACCGGTAAACCCTCTCTCTGGCTTCGGTAGCGATGCTGCAA

GCAATGCCAAGCCTCGGCGCCAAGCCAGCCAGCCATCTACTCCTCACTTGTCACCCACGTTTCCAGAAAG

CCTGGATCACTCGCTGGTGAGGAGGGCGACGAGCGACCCACCTACCGTCATCCACCAAGCTGGCCTTCCC

GTATACATCCCTGCTTTCTCCAATGGTACCAACAGCTATTCCTCTTCCAATGGTGGCTCCACATCCAGCT

CACCCCCCGAGTCAAGACAGAAGCATGACTGCGTGATCTGCTTTGAGAGTGAAGTCATTGCGGCCCTGGT

CCCCTGTGGCCACAATCTCTTCTGCATGGAGTGTGCCAACAAAATCTGTGAGAAGGAGATGCCATCGTGT

CCTGTTTGCCAGACAGCTGTTACTCAGGCAATCCAAATTCACTCTTAA

>Budgerigar MYO5B

ATGGATTCCTGTGGGGTAACGGGCAACGAAACTCCTCTGAAAGGAAACGAGTGGTCAATGAGCATGTTGC

TGGGGATGACCCTTTTGCTGCTGAGAGCTCCAAAGCCTTCAGTGGTGCCTCCTCTCCTCGTCGTTCCCTT

CCTTTCCCTGCCCGCACTCCGTCGGACGCGGTGGTGTAAAGGTCGCTCGGTCCGGCGGTCGCTCGCTGCC

TGCCGGCCCCATGCCCCTCGCCTAGCGCCGCTCGCCCCTGCCATGTCCGCCTCGGAGCTCTACACCAAGT

ACACAAGGGTTTGGATTCCTGACCCTGATGAAGTTTGGAGATCGGCAGAAGTTATCAAGGATTACAAAGA

GGGAGATAAAAGCCTGCATCTGAAACTTGAAGATGAAACTCTCTACGAATATCCCATTGACCTGCAAGGA

AATGCATTGCCTTTCCTGCGCAATCCAGATATCCTTGTGGGAGAGAATGACCTGACTGCCCTGAGTTACC

TGCACGAGCCTGCAGTGCTCCACAACCTCAAAGTCAGGTTCACCGAGTCCAACCACATCTACACGTACTG

CGGTATTGTACTTGTCGCCATTAATCCATATGGGCAGCTGCCAATCTATGGACAAGATGTCATCTACGCG

TACAGTGGCCAAAACATGGGGGATATGGATCCTCACATCTTTGCCGTGGCAGAGGAAGCATATAAACAGA

TGGCTAGGGATGAGAAGAACCAGTCCATCATCGTGAGTGGGGAATCTGGTGCTGGAAAGACAGTTTCTGC

CAAATATGCCATGCGCTTCTTTGCAACCGTTGGTGGTTCTGCCAGCGAGACCAACATTGAGGCCAAAGTT

CTTGCATCGAGCCCAATTATGGAGGCAATTGGAAATGCTAAAACAACAAGGAATGACAACAGCAGTCGCT

TTGGGAAATATATTCAGATTGGCTTTGATAAACGATACCACATCATTGGTGCGAATATGAGGACTTACCT

GTTGGAAAAATCACGAGTTGTATTCCAGGCGGAGGATGAGCGCAACTACCATATCTTCTATCAGCTTTGT

GCCTCAGCGAGTCGCCCAGAATTCAAAGACCTTGGGCTAACGTGTGCTGAAGACTACTTCTACACATCTC

AGGGGGGTAGCACATCTATTGATGGTGTGGATGATGCCGAGGACTTTGAGAAAACAAGGCATGCCTTCAC

CCTACTTGGAGTGAAGGAGTCTCATCAGATAACCATTTTTAGGATAATTGCTGCCATTCTGCACCTAGGG

AACTTGGAAATCCAAGCGGAACGAGACGGCGATGCCTGCAGTATATTGAGTGATGATGAGCACTTGAACA

ACTTCTGCAGCTTGCTGGGTGTGGAGCACAGCCAGATGGAGCACTGGCTGTGCCATCGCAAGCTGGTCAC

CACGGCCGAGACCTACGTGAAGAGCATGTCCATGCAACAAGTGGTGAATGCCAGAAATGCCCTGGCCAAG

CACATCTATGCCCGGCTCTTCAACTGGATTGTGCAACACATCAACAAGGCCCTGCACACCAGTATCAAGC

AGCACTCCTTCATCGGTGTGCTCGATATCTATGGGTTTGAAACTTTTGAAGTGAACAGCTTTGAACAGTT

TTGTATCAACTACGCCAATGAAAAGCTCCAGCAGCAGTTCAACTTGCATGTGTTTAAGCTGGAACAAGAA

GAGTACATGAAGGAAGGAATCCCTTGGACTCTCATAGACTTCTATGATAACCAGCCCTGCATAGACCTTA

TAGAGACGAAACTTGGTATCTTGGACCTGCTGGATGAAGAGTGCAAGGTTCCCAAAGGCACTGACCAGAA

CTGGGCTCAGAAGCTGTATGACCGGCACTCCAACAGCCAGCACTTCCAGAAGCCCCGGATGTCCAACACC

TCTTTCATTGTCCTGCACTTTGCTGATAAGGTGGAATACCAGAGTGAGGGATTTCTGGAGAAGAACAGGG

ACACTGTGTATGAGGAACAGATCAACATCCTGAAAGCCAGCAAGTATCAGATGGTAGCAGACTTATTCCA

AGATGAGAAGGATGCTACACCCACCACCCCTGTGGGAAAGGGAACACCCAAAATCAGTGTCCGTTCTGCC

AGACCAGCCATCAAAGCTGCCAATAAGGAGCACAAGAAAACTGTGGGGCACCAGTTCCGCAACTCACTGC

ATTTGCTCATGGAGACCCTGAATGCCACCACCCCACACTACGTGCGCTGCATCAAGCCCAACGATGAAAA

GCTCTCCTTCAAGTTTGATTCAAAGAGAGTGGTGCAGCAGCTGAGAGCTTGTGGAGTGCTGGAGACCATC

CGCATCAGTGCAGCTGGCTTCCCATCCAGATGGTCCTACCACGACTTTTTCAATAGGTATCGTGTTCTTA

TGAAGAAGAGAGACCTCTCTAACAATGACAAGAAACAGATATGTCGGACCCTGTTGGAAGACCTCATTAA

GGATCCAGACAAGTTCCAGTTTGGCCGTACCAAGATCTTTTTCCGTGCAGGCCAGGTGGCTTATCTGGAG

AAACTGCGAGCAGATAAGTTCAGAGCTGCCACAATCATGATTCAGAAGACAGTGCGGGGTTGGCTGCAGT

GGGTCAGGTACAGAAGGCTCAGACAAGCTACAGTCATCATCCAGCGCTACACACGTGGGCACCTGGCACG

GAGACTCGCTGAGCACCTGAGGAGGACAAGAGCTGCCATCATCTGCCAGAAGCAGTACCGAATGCTACGG

ATCCTCCGAGCTTACCAGAGGGTCCGCACTGCAACCATCACCATTCAGGCTTTTGCTCGGGGCATGATTG

TCAGGAGGATTTATCACAAGATCCTGGTGGAGCACAAAGCCATCATCCTCCAGAAGTACATGCGGGGCTG

GCTGGCCCGCACTCGGTTCCGCCGGGTCAGGGCAGCCACCATTGTCCTGCAGTGCTGCTACCGGCGCATG

AAAGCCAGGCAGCAGCTGAAGGCACTGAAGATTGAGGCCCGCTCAGCACAACACCTAAAGAAGCTCAATG

TTGGCATGGAGAACAAGGTGGTCCAGCTCCAGAGGAAGATTGATGAGCAGAACAAGGAATACAAACTTCT

GAATGAGCAGCTCTCAACACTTACGTCAGCCCACTCCTCTGAGGTGGAAAAGCTGAAGAAAGAGCTGGTG

CGGTATCAGCAGAGCCACCAGGGTGATGGCAACCAGCTTGTCAGCTTGCAAGAAGAGATGGAGCACCTCC

AGCTGGAGCTTGAAAAGGCTCATCAGGAGAGGAAGGTCATGGAAGACAGCTACGTTAAGGAGAAAGACCT

ACTGAGAAAGCGCATATCCGAATTAGAAGAAGAAAATGCTTTCCTGAAGCAGGAAAAAGAGGAGCTTAAC

AGCAGGATCCTGTGTCAATCTGAAGATGAATTTGCACAAAACACAGTTGAGGAAAATATCCAGGTGAAGA

AAGAGCTGGAAGAAGAGAGATCTCGTTATCAGAACCTGGTAAAAGAGTATTCAAGGCTGGAGCAGAGATA

TGACAACTTGAGGGATGAAATTACTATTATAAAGCAGGCACCTGGGCACAGAAGAAACCCATCCAACCAG

AGCAGTTTGGAGTCCGATTCCAATTACCCATCCATCTCAACCTCTGAGATAGGAGACACCGAAGATGTAA

TGCCACAGGTGGAGGAGGTTGGGATGGAAGAAGCAGCCATGGACATGACCCTCTTCCTGAAGCTACAGAA

GCGAGTGAGGGAGCTCGAGCAGGAGAGGAAGAAGCTGCAAACCCAACTGGAGAAAAAGGAGGATGAGAGC

AAGAAGCCCCAGGTAACTGAAGTGAAGACTGAAGTGACTTCAGAGCACGAGGATTTTGCATACAACAGTC

TGAAGAGGCAAGAGCTGGAATCGGAGAACAAGAAACTGAAGAATGAGCTTAATGAGCTGAGGAAAGCTAT

TGCAGACAAAGCAACCCAGAACAACTCGTCCAACGATATTCAGGACAGTTATAACCTGTTGCTGAACCAG

CTGAAATTGGCCAATGAGGAGTTGGAAGTGAGGAAAGAAGAGGTGCTCATCCTGAGGACACAAATTATAA

AGGCAGCCCAGCAAAAGGAGATTGGGAAAGACATGGAAAGCATTGCCAGCGACACCAGATGGCCGAACAG

TGACAAGCACATTGATCAGGAGGACGCGATTGAAGCCTACCAGGGGATGTGCAAGACAAACCACATGACT

GAGGACTGGGGGTATCTCAATGAAGATGGAGAGCTCGGCTTGGCTTATCAAGGTTTAAAGCAAGTTGCCA

GGTTGCTGGAAGCACAGCTACAGGATCAGAGAAGAGAGCATGAAGAGGAGGTGGAAGCTCTGAAGAACCA

GATGGACACAATGAAAGAAGAGATGGAGAAGCAGCAGCAGAGTTTCCTGCAGACCCTGCAGCTCTCTCCA

GAGGTTCAGGTGGAGTTTGGACTTCAGCGAGAAATTACACGTCTTACCAATGAGAACCTGGATCTTAAAG

AATTGCTAGAGAAGTTGGAAAAGAATGAGAAGAAGCTGAAGAAGGAGCTGAAGATTTATATGAAGAAGGT

CCAAGATTTTCAAGCATTCCAGACCATGGTACCAGCAGAGAGAAGGCAGCGTGAATGTCACATGCAAGTT

GCTGTTCAGAGGAAGGAGAAAAATTTTCAGGGCATGTTGGAATATTATAAGGAAGACGAGCCACTCCTCA

TCCAAAACCTCATTACAGATCTCAAGCCCCAGGCGGTGTCTGCTACTGTTCCTTGCCTTCCTGCCTATAT

CCTCTACATGTGCATCAGACACGCGGATTACATCAATGATGACCAGAAAGTGCACTCCTTGCTCACCTCC

ACCATCAATGGCATTAAGAAAGTGCTGAGGAAACACAAAAAAGATTTTGAGATGACATCGTTTTGGCTGG

CGAATACACGTCGCCTCCTGCACTGTTTAAAGCAGTACAGCGGAGATGCGGGTTTCATGACACAAAATAC

GCCTAAACAGAACGAGCACTGTCTGAAGAACTTTGACCTGACCGAATACCGCCAGGTGCTGGACCACCTC

TCCATCCAGATCTACCAGCAGCTCATTAAGATAGCAGAGGGCATACTCCACCCCATGATTGTGTCTGCGG

TGTTGGAAAATGAGAGTATCCAAGGGCTTTCTGGTATCAAACCAATGGGCTACAGAAAACGCTCCTCCAG

CATGGAAGATTGCGACAGCTCCTACAGCCCAGATGAAATCATTCGCCAGCTGAACACATTCCACACCATC

ATGTGTGACCAGGGTCTGGACCCAGAGATCGTGCAGCAGGTCTTCAAGCAGCTGTTCTACGTGATCAATG

CAGTTACCCTGAACAACCTCCTATTAAGGAAGGATGTCTGCTCATGGAGCACAGGCATGCAGCTAAGGTT

TAACATAAGCCAGCTGGAGGAATGGCTCCGTGCGAAGAATCTGCAGGAGAGTGGAGCAGCACAAACTTTG

GAGCCCTTGATTCAGGCAGCACAGCTTCTGCAGCTGAAAAAGAAAACTTCGGAAGATGCTGAGGCCATCT

GCTCCTTGTGCACATCACTCACAACCCAGCAGATTGTAAAGATACTTAATCTCTACACTCCAGTGAATGA

GTTTGAAGAACGTGTGACAGTAGCTTTCATTCGAGACATACAGATGCACTTGCAAGAGCGAAATGACCCT

CCGCAGCTGCTGTTAGACTTCAAGCATATGTTCCCAGTTTTGTTCCCATTCAACCCATCCTCCATAACCA

TGGACTCTATCGATCTCCCCGCTTCTCTCAACTTGGAATTTCTCAATAAAGTCTGA

>Budgerigar NARS

ATGNCCCCCGGTGGCCACGAGCTGCACTGTGACTACTGGCAGGTTGTGGGGCTGGCCCCAGCTGGGGGGG

CCGACAACGTCCTCAACGAGGCCGCGGACGTGGACGTGCAGCTGAACCAGAGGCACATCCCGCTGCGAGG

CGAGGCCATGGCCAAGGTGCTGCGGGTGCGTGCGGGGCTGCTGCAGGCCTTTAGGGAACACTACTGTGCC

CATGGGTACTGTGANGTGACCCCCCCCACACTGGTGCAGACACAGGTCGAGGGGGGCTCGACCCTATTCA

AACTGGATTTCTTTGGGGACGAGGCTTTTCTGTCCCAGTCCTCCCAGTTGTACCTGGAAACCTGCATCCC

GGCACTGGGGGATGTGTTCTCTATAGGGCCGTCCTATAGGGCCGAGCAGTCCCGCACGCGGAGGCACCTG

GCAGAGTACACCCACATCGAGGCTGAGTGTCCCTTCATCACCTTCGAGGACCTATTGGATCGTATAGAGA

CCCTAGTGTGTGATGTCCTCTATAGGGTCCTGGCCTCCCCTGTGGCCCCATTGATCCAGGACCTCAACCC

TGGTTTCCAGCCCCCCAAGCGACCCTTTAGACGTATGGACTATAGGGAGGCCATAGAGTGGCTGAAGGAA

CATGGAGTAAAGAAGGAGGATGGGACTTACTATGAGTTTGGGGACGACATTCCCGAAGCCCCCGAGCGCC

TCATGACCGACACCATAAATGAGCCGATCCTATTGTGCCGCTTCCCAAGGGACATCAAGTCCTTCTATAT

GGAACCTTGTCCCGAGGATCCCAGTTTAACCCAGTCTGTTGACGTCCTAATGCCCAACGTGGGGGAGGTC

GCAGGCGGCTCCATGCGCATATGGGACCTGGAGCAGCTACTCCAGGGTTACCAACGGGAGGGCATCGACC

CCAAAGCCTATTACTGGTACACAGACCAGAGGAAATATGGGACCTGCCCCCATGGAGGTTATGGGTTGGG

ACTGGAGCGGCTGCTGACCTGGATCCTCAACCGGCCCCATATCCGGGATGTGTGTCTGTATCCTCGCTTC

CTGCAGCGCTGCCGGCCATAA

>Budgerigar ONECUT2

ATGNNNCGGCCCGCCATGGTGCCCAGCATGGCCTCGCTGCTGGACGGCGCCGCCGAGTACCGGCCCGAGC

TCTCCATCCCGCTGCACCACGCCATGAGCGTGCCCTGCGAGGCCTCGCCCCCCGGCATGGGCATGAGCGG

CACCTACACCACGCTGACGCCGCTCCAGCCCCTGCCGCCGATCTCTGCCGTCTCCGACAAGTTCCACCAC

CCGCACGCCCACCCGCACGCCCACCACCACCATCACCACCACCAGCGCCTGCCGGGCAGCGCCGGCGGTG

GCTTCGCGCTCATGCGGGACGAGCGCGGGCTGCCGGCCGTCAACAGCCTCTACGGGCCCTACAAGGAGGT

GCCGGCCGTGGGGCAGAGCCTCTCGCCGCTGGGCAACGGGCTGGGCCCGCTCCCCGGCGCCCAGCAGGGC

CTCCACGGCTACGGGCCGCCCGGCCACGACAAGATGCTGAGCCCCAACTTCGAGGCGCACGCGGCGATGC

TGGCGCGGGGGGAGCAGCACCTGGCCCGGGGGCTGGGGACACCCCCGGCCATGCTGCCGCCCCTGAACGG

CGCCCACCACTCCGCGCCCCCCGGGCCGCCGCCGCCGCACGGCCCCGCGCTGCCCGCCGGCCGGGAGCGG

CCGCCCCCCGCCGCCGGCCCGCAGGGGAGCGGCGCGGGTCAGCTGGAGGAGATCAACACCAAGGAGGTGG

CTCAGCGGATCACGGCCGAGCTGAAGCGCTACAGCATCCCGCAGGCGATCTTCGCGCAGAGGATCTTGTG

CCGCTCGCAGGGGACCCTCTCGGACCTGCTGCGGAACCCCAAGCCCTGGAGTAAGCTCAAGTCAGGCCGG

GAGACTTTCCGGAGGATGTGGAAATGGTTGCAGGAGCCGGAATTTCAGAGGATGTCGGCGCTCAGGCTGG

C

>Budgerigar POLI

ATGNNNNGCAGCGTCATTGCACACCTTGACCTCGACTGCTTCTATGCGCAGGTGGAGATGCTCCGGGCCC

CGGAGCTCAGGGACAAGGCCTTGGGGGTGCAGCAGAAGTCCCTCGTGGTCACCTGTAACTACGAAGCCCG

GCGCCATGGGGTGAAGAAGTTGATGTCCCTCAAAGAGGCCACGGAGAAGTGTCCTCACTTGGTGCTGGTC

AATGGAGAAGACCTCACTCCCTATAGGGAGATGTCCTACAAGGTCACAGCCATCAACGTGCAGGATCCAA

CCCATCGTAAGCTTGCCATTGGATCCCAAGTGATGGAAGAGCTCCGGGAATGCCTTTACTCCACGTTGGG

CCTCACCAGCAGCGCTGGGGTGGCCACCACCAAGTTCTTGGCCAAGGTGGTCTCTGGGACCTTCAAACCC

AACCAACAAACCCTGCTCCTGCCCCACAGCACCCAGGAGCTGCTGAGGAACCTGGGGTCCATCCGGAAGG

TGCCAGGCATTGGCTCCAGGACTGCGGAACGCCTGGAAGCATTGGGTGTGAAGACAGTGGTGGATCTCCA

AAGGTTTCCATCTGCTGGCTTGGAGAAGGAGCTGGGAATTGCGCTTGCTCAACGGATCCGGAAGCTTGGC

TATGGGCAGGATGAGGCCCCTGTGACACCATCAGGACCTCCTCAGTCCTTTAGCGATGAAGATTCCTTCC

GGAAATGTTCCTCAGAGGCAGAAGTCCAGGAGAAACTGGGAACGATGCTTCCAAGCCTCTTGGAAAGAAT

CCGCCGGGATGGGAGGCAACCACGCACCATAAGGTTGAGCATCCGCCGCTTCTCCTCATCAGGAAAGGGG

TTCCATCGGGAATGCCGTCAGTGCCCTATCCCACCGCATCTCCTTCCCAAATTTGGGAAAGGTCCTGGGA

ACCTCCTCTCCCCCTTGGGGAGCATCTTAATGAAGCTCCTGCGGAAGATGATCCCAACAGAGCTCCCATT

CCACCTCACCCTCCTCAACGTCTGCTTCTCCAACCTCCAGGAGCTTCCCACCAGGAAGGGTTCCATTGGC

TTCTACCTCAAGGGCAAGGGCCCAGCACCTGCTTCTGGCCAAGGAGGGAGTGGGAAGGTGCTTGTCTAG

>Budgerigar RPL17

ATGGTGCGCTACTCTCTGGACCCTGAAAACCCCACGAAATCATGCAAATCACGGGGGTCCAACCTGCGAG

TGCATTTCAAGAACACTCGTGAGACAGCCCAGGCCATCAAGGGCATGCACATCCGCAAGGCCACCAAGTA

CCTGAAAGATGTCACCCTGAAGAAGCAGTGCGTTCCTTTCCGCCGCTACAATGGGGGTGTCGGTAGATGC

GCCCAGGCCAAGCAGTGGGGCTGGACTCAGGGACGCTGGCCAAAGAAAAGCGCAGAGTTCTTGCTGCACA

TGCTCAAAAACGCAGAGAGCAACGCTGAGCTCAAGGGTCTTGACGTGGATTCTCTGGTCATCGAGCACAT

CCAAGTCAACAAGGCCCCCAAGATGCGCCGGCGCACCTACAGAGCGCATGGCAGGATCAACCCCTACATG

AGCTCCCCCTGCCACATCGAGATGATCCTCACTGAGAAGGAGCAGATTGTCCCCAAGCCGGAGGAGGAGG

TTGCTCAAAAGAAAAAGATCTCCCAGAAGAAGCTGAAGAAGCAGAAGCTCATGGCTCGGGAGTAA

>Budgerigar SKA1

ATGNNNNTTCCTGCGTATATGAAATGCCGCATAACATGTGATCAGATCAATGCAGTTGTTGAAAACCTGA

ACAAAGCAATTATGAGCAAATACAGGATCCGCAATCAGGCTCCAAGTTCTATGAGTGCAGCAGACAGAAA

CACGTACTACAGATACTTAAGAGAAGAAAGGAGTAACAAAGAAGGTTGTGTTTTTATTGTGGAGGAGGAT

ATCAAACTGTTCACCCCTATGAAAACGGATAAGCGCTTCCATAAGATCATCGTCATCCTGCGCCACTGCC

AAAGGCTGAGAGAAATTCGTGTCTCTGGAATTACCCGCTATGTCATATGCTAA

>Budgerigar SMAD4

ATGGACAATATGTCTATTACTAACACACCAACAAGTAATGATGCCTGTCTGAGCATTGTTCACAGCTTGA

TGTGCCATCGACAAGGTGGAGAGAGTGAAACTTTTGCAAAACGCGCCATTGAAAGTTTAGTTAAAAAGCT

AAAGGAGAAAAAAGATGAATTGGATTCTTTGATTACAGCTATAACCACAAATGGAGCTCATCCTAGCAAG

TGTGTTACAATACAGAGAACGCTGGATGGGAGGCTTCAGGTGGCTGGTCGCAAGGGATTCCCTCATGTGA

TTTATGCTCGTCTTTGGAGGTGGCCTGATCTTCATAAAAATGAACTCAAGCATGTTAAATATTGTCAGTA

TGCTTTTGACTTAAAATGTGACAGTGTCTGTGTAAATCCTTACCATTATGAGCGTGTAGTATCACCTGGC

ATCGATCTCTCAGGACTGACACTACAGAGTTCTGCTCCATCAAGCATGCTGGTGAAAGACGAATACGTTC

ATGACTACGAGGGGCAGCCATCACTGTCTTCTGCAGAAGGCCATTCAGTCCAAACCATCCAGCATCCACC

AAGTAACAGGGCATCTAACGAGCCTTATAGCACCCCAGCCATGCTAGCTCCTACTGAGGCTAGCACTACC

AGCACCACTAATTTTCCCAACATTCCTGTGGCTTCAACAAGTCAACCTACCAGTATATTGACAGGTAGCC

ATAGTGATGGACTCTTACAGATTGCTTCAGGGCCTCAGCCAGGAACTCAGCAGAATGGGTTTACAGCTCA

GCCAGCTACTTACCATCACAATAGTACTACAACTTGGACTGGAAGTCGGACGGCAGCCTACACACCTACC

ATACCTCACCACCAGAATGGCCATCTTCAGCATCATCCACCTATGCATCCTGGACATTACTGGCCAGTTC

ACAATGAACTTGCATTCCAGCCTCCTATATCAAATCATCCTGCTCCAGAATATTGGTGTTCAATCGCTTA

TTTTGAAATGGACGTGCAAGTTGGGGAAACATTTAAAGTCCCTTCAAGCTGTCCAATTGTGACTGTTGAT

GGATATGTGGATCCTTCTGGAGGAGATCGCTTTTGCCTGGGCCAGCTTTCCAACGTGCATAGAACAGAAG

CCATTGAGAGAGCAAGGTTGCACATAGGTAAGGGAGTGCAGCTGGAGTGCAAAGGTGAAGGTGACGTGTG

GGTTAGATGCCTCAGTGACCATGCAGTCTTCGTCCAGAGTTACTACCTGGATAGAGAAGCAGGGCGTGCT

CCAGGGGATGCTGTTCACAAGATTTACCCAAGTGCATATATAAAGGTGTTTGACCTGCGCCAATGCCATC

GTCAGATGCAGCAGCAGGCTGCCACTGCCCAAGCTGCTGCTGCTGCTCAAGCTGCAGCAGTAGCAGGAAA

CATCCCTGGACCAGGATCAGTAGGTGGAATAGCCCCAGCCATTAGTTTGTCAGCTGCTGCTGGAATTGGT

GTAGATGATCTTCGCCGCTTATGCATACTCAGGATGAGTTTTGTGAAAGGTTGGGGACCTGATTACCCAA

GGCAGAGCATCAAAGAGACACCGTGCTGGATTGAAATTCACTTACACCGTGCGCTCCAGCTTCTAGATGA

AGTACTTCATACCATGCCTATTGCAGACCCACAACCTTTAGACTGA

>Budgerigar SMAD7

ATGTTCAGGACCAAACGCTCGGTGCTCGTTCGGCGGCTCTGGCGGAGCCGTGCGCCCGGCGGTGAGGAGG

AGGAGGAGGAGGAGGCGGCGGCGGCGGCCGAGGCCCGGGCGCATGCCTGCGGCGAGGGGCGCGGGTGCTG

CCCGGGCAAAGCGGGCCGCGGGTGCGGAGCGTCCGCCGGTGCGGAATCGGAACTGAAGGCGCTGACCCAC

ACCGTGCTGAAGCGGCTGAAGGAGCGGCAGCTGGAGGGGCTGCTGCACGCCGTCGAGTCCCGCGGTGGGG

CTCGGACCCCCTGCCTGCAGCTGCCTGCCAAGGCCGACTCCCGGCTCGGCCAGCACTCGTACCCGCTGCC

GGTGCTGCTCTGCAAGGTGTTCCGCTGGCCAGACCTCCGGCACTGCTCCGAAGTGAAGCGGTTATGTTGC

TGTGAATCCTACGGCAAGGCTCACGCCGAGCTCGTCTGCTGCAACCCGTACCACCTCAGCCGGCTCTGCG

AGCTAGAGTCTCCCCCTCCACCCTACTCCAGATATCCAATGGATTTTCTCAAACCAACTGCAAATTGTCC

AGACTCTGTGCCTTCCTCCACTGAAACAGGGGGAGCTAATTGCCTAGCCCCTGCGGGGCCTTCAGATTCT

CAAGTACTTCAGGAGCCGGGGGATCGTTCACACTGGTGCGTGGTAGCATACTGGGAAGAGAAGACACGTG

TGGGTCGGCTGTACTCTGTCCAAGAGCCCTCCCTGGATATCTTCTATGATCTACCTCAGGGGAATGGTTT

CTGCCTCGGACAGCTCAACTCTGACAACAAAAGCCAGCTGGTTCAGAAGGTCCGCAGCAAGATTGGCTAT

GGCATCCAGCTCACCAAGGAAGTGGACGGCGTGTGGGTATACAACCGCAGCAGTTACCCCATCTTCATCA

AGTCGGCCACACTGGACAACCCTGACTCCAGGACGTTGCTGGTTCACAAAGTGTTCCCAGGATTTTCCAT

CAAGGCTTTTGACTACGAGAAGGCATACACCTTGCAGAGACCCAACGACCACGAGTTCACGCAGCAGCCA

TGGACTGGATTTACCGTTCAGATCAGCTTTGTGAAAGGCTGGGGCCAGTGCTACACTAGACAGTTCATCA

GCAGTTGCCCATGCTGGTTGGAGGTTATCTTTAATAACCGGTGA

>Budgerigar ST8SIA3

ATGNNNTCCCAGTTCGCCCTCAAGTTCCTGGATCCTTCCTTTGTTCCCATCACCAATTCCCGAAGTCAGG

AATTCCAGGACCAGCCTCCCAAGTGGGTCTTCAACCGGACCGCGTTCGACCAGCAGAGGCNNNNNTACTC

CAGCCACAAATACGTCTACTCCGTCAGCTCCTCCTTCCTCTCCCTCCTTCCGGAACATTCCCCAATCCTG

GAATCCCATCCCCCCACCTGCGCCGTGGTCGGGAATGGGGGAATCCTATTGGGAAGCGGCTGTGGGGCCG

AGATCGATAGGGCCGACTTCGTGTTCCGCTGTAACTTCGCCCCTACGGATGGATTCCATAGGGATGTGGG

GCGGAAAACCAACCTGACCACCTTTAATCCCAGTATCCTAGAGAAGGATTATAACAACCTATTGACCATA

CAGGACAGGAATAACTTCCTATTGAGCTTAAAGGGTCTGGATGGGGCCATCCTATGGATCCCGGCCTTCT

TCTTCCATACGTCGGCCCCGGTGACTAGGACACTAGTGGATTTCTTTGTGGAGCATCGGGATAGGATCAA

GGTCCGGTTGGCTTGGCCGGGGAATATCATACAGCATATTAATAGGTATGGGAAAGCCAAGCAGCTCAAC

CCCAAGCGCCTGAGCACCGGGATCCTAATGTACACCTTGGCCTCGGCCCTATGCGACCGGATCCACCTTT

ATGGGTTCTGGCCCTTCCCATTCCACCCCCATTCCCGACGGGATCTGCCCTATCACTACTATGACAAGAG

GGGCACCAAGTTCACCACCAAGTGGCAGGAATCCCATCAGCTCCCATTGGAATTCCAGCTGCTCTATAGG

ATCCATAGGGAAGGGTTGGCCAAGATGACCCTATCCCATTGCTCCTAA

>Budgerigar TCF4

ATGCATCCCCACCACCACCAGCGCATGGCTGCCTTAGGGACGGACAAAGAGCTCAGCGATCTCTTGGATT

TCAGTGCGATGTTCTCCCCCCCGGTGAGCAGTGGGAAGAACGGACCAACTTCCTTGGCAAGCGGACACTT

CTCCGGCTCCANNNNNCAAAGCCTCTTGGGAAGCGCCATGGAGCTGGGCAATCCCGGAACGCTGTCTCCC

ACCAAAGCCGGCTCCCAGTATTACCAGTATTCCAGCAATAATCCCCGACGGAGAGGCATCCATAACGCTT

CCATGGAAGTGCAGAGCAAGAAAGTTCGGAAAGTTCCGCCGGGATTGCCCTCATCCGTTTACGCCCCATC

CGCGAGCACTGCCGACTACAACCGGGATTCTCCCGGTTTTCCATCCTCCAAACCGGGCGCCAGCACTTTT

CCAAGCTCCTTCTTCATGCAAGATGGCCACCACAGCGGTGATCCATGGAGCTCTTCCAGTGGGATGAACC

AGTCCGGCTATGGGGGAATGCTGGGAAACTCTTCCCATCTCCCACAATCCAGCAGTTACTGCAGCCTGCA

TCCGCACGAGCGCTTGAGCTTCCCATCCCATTCCTCGGGGGAGCTCAATTCCAGCCTGCCCCCGATGTCC

ACCTTCCATAGGGGCACCAGTGGACACTACAGCACCTCCTCCTGCACCCCCCCGGCCAACGGCACCGACC

CCATCATGGCCAACAGAGGTAAGGGGCCAGCCGGAAGCTCCCAGACTGGGGATGCCCTTGGGAAAGCGCT

CGCATCGATCTACTCTCCAGAGCCCACCACCACCAGCTTCCCATCCACTCCTTCAACTCCTGTTGGCTCT

CCCCCTTCTCTCGCAGCAGGCCCAGCTGTATGGTCTAGGAATGGAGGCCAAGCATCATCATCTCCCACTT

ATGAAGGTCCCTTACACTCCTTGCAAAGCCGGATCGAGGACCGCTTGGAAAGGTTGGATGATGCCATCCA

CGTCCTAAGGAATCACGCTGTGGGGCCGGCCCCATCCCTGCCCCATAGCGACATGCACGGCTTGATAGGA

GCCGCTCCCAACGGATCCATNNNNGTCGGATCCCATCGGGAAGAGGGCGTCGGAATCCGAGGGAGCCATT

CGCTGGTCCCCAACCAGGTCCCTGTTCCCCAACTTCCCGTCCAATCTTCCACGTCTCCGGAATTGAATCC

CCCCCAGGATCCCTATCGGGGAATTCCCAGCGCTCTCCAAGGCCAAAGCGTCTCGTCGGGAAGCTCGGAG

CTGAAATCCGATGAGGAAGGCGATGAGAACATCCAAGATTCCAAAGCTCTGGAAGACAAGAAGCTGGAGG

AGGACAAGAAGGAGCTCAAATCCATTACTAGGTCAAGATCTAGCACCAACGACGACGAGGACCTGACCCC

GGAGCAGAAGGCCGAGCGCGAGAAGGAGCGGCGCATGGCCAACAACGCCCGCGAGCGGCTGCGCGTGCGC

GACATCAACGAGGCCTTCAAGGAGCTGGGCCGGATGGTGCAGCTGCACCTCAAGAGCGACAAGCCCCAGA

CCAAGCTGCTCATCCTGCACCAGGCCGTGGCCGTCATCCTCAGCCTGGAGCAGCAAGTGCGGGAGAGGAA

CCTGAACCCCAAGGCCGCCTGCCTCAAGAGACGGGAAGAGGAGAAGGTCTCCTCGGATCCCCCTCCGCTC

TCCCTGGCCGGGCCCCATCCTGGCATGGGGGATGCAGCCAATCACATGGGACAGATGTAA

>Budgerigar TXNL1

ATGNNNCCCTGTGTCCGGATCGCGCCGGCCTTCAGCGCCCTCAGCACCAAGTACCCGCAGGCCGCGTTCC

TGGAGGTGGATGTGCACCAGTGCCAGGNNNNNATCTCGGCCACGCCGACATTCCTGTTCTTCCGGAACAA

AGTCCGGATCGACCAGTACCAGGGAGCGGATCCCATGGGATTGGAGGAGAAGCTCAAGCTGCACCTGGAG

CACGAGGCCGGGGAGGAGGCCGATATTCCCAAGGGATANNNAAAGGCCGGCTGCGAGTGCCTCAATGAGA

GCGACCAGCATGGCTTCGACAACTGCCTCCGGAGAGATGCTTCCTACCTGGAATCCGACTGCGACCAGCA

GNNNTTCAGCCAACCCGTCAAGCTCTACTCCATGAAATTCCAAGGCCCGGACAACGGCCAAGGCCCCAAA

TCCGTCAAGATCTTCACTAATCTCACTCGATCCATGGATTTTGAGGAAGCCGAACGGAGCGAGCCCACCC

AATCCCTGGAATTAAGCCGGGATGAGATCCGGGAAGACGGGATCATCCCGTTGCGCTACGTCAAGTTCCA

GAATGTCAATAGCGTGACCCTCTTTGTCCAGTCTAACCATGGGGATGAGGAGACCACCAGGATTTCCTAC

TTCACCTTCATTGGGACTCCAGTTCAGGCCACTAACATGAATGACTTCAAGAGGGTGGTGGGCAAGAAAG

GAGAGAGCCACTGA

>Budgerigar WDR7

ATGNNNTCCAGCATCACTCCAGGCCTCCCCCTGAGCCCCGCAGCAGACTCAGCTCGCTCAGCACGCCACG

CTCTGGCCCTCATCGCCACCGCCCGCCCGCCAGCCTTCATCACCACCATCGCCAAGGAGGTCCACAGGCA

CACGGCCCTGGCGGCCAACGCGCAGTCCCAGCAGAGCATCCACACCTCCAGCCTGGCCAGAGCCAAGGGG

GAGATCCTCAGGGTGCTGGAGATCCTCATTGAGAAGATGCCCACGGATGTGGTGGAGCTGCTGGTGGAGG

TGATGGACATCATCATGTATTGCCTGGAGGGATCCCTAGTCAAGAAGAAAGGACTCCAGGAATGCTTCCC

AGCCATCTGCAGGTTCTATATGGTGAGCTACTACGAGCGGAGCCATAGGATAGCAGTGGGAGCTCGCCAT

GGATCCGTGGCCCTATATGATATCCGCACTGGGAAGTGCCAGACTATCCATGGCCATAAAGGTCCCATAA

CTGCAGTGGCTTTTGCTCCCGATGGAAGGTACCTGGCTACCTATTCCAACACGGATAGTCACCTCTGCTT

CTGGCAGGTA

>Capitella telata DCC/NEO1

ATGNNNGAATCTTTCCGCGAATTCCGCTTCGTCGTGGAGCCTTCGGACACGGTCGCCGTCCGATCTCGAC

CCGCGATGCTCGACTGCAGTCTGCAGAACGCCAACGGTGCCTCACCTCAAATCGAGTGGCTCAGAGAAGG

GGCCGCGTTGAACCTGGACAACAGAAGGTTCATGTTTGCAAACGGATCGCTGTATTTGGAGCAAGTGGAG

CATTCGAAGACGAATCGTCCCGATGAGGGTTTTTACCAATGCAGCGGCAAAGTGGACGGACTGGGCACCC

TGGTCAGCCGCAAAGCTCGCTTCGACGTCGCTTATCTGGGCAAAACCTTCACGGAGGAACCCGGGGACGT

GCGGGTGCACCTGGGCGACGTGGCCATGCTGCCCTGCCGCATGGACGCCTCCCCCAGCCCCTCGGTGCGC

TGGTACAAGGACGACCAGCAGCTGCACCCCGATCTCTCCTCCTTCCACATCGTGCACGCGGACGGCATGC

TGGAGATCCAGAAGGTGCAGTTCGCCGACTTCGGGCGATACAAGTGTCGCGTCGAGAACGTGGAGCGGTC

GAAAATGAGCGAAGAAGCCGAACTGAGCCAGAACAGCGATCCAGCCACCTACCAGGGAGTCGCCCCGCAC

TTCATCCGTGAACCCTCAAACCAGGTGGCGGTCGAAGGCACCAGTGTTATGATGTACTGTGGCGCCAATG

GTCGTGACATCCAAGCCAACCCGCCTACTTTGACCTGGCTCAAAGATGGTGTCACTGTGGAGTTAGGCAC

TAGATTGTCCACGGTTGGCTCCGGCAGTTTGCGTATTGATAGCTTGCAGTTAGAAGACGCAGGACTTTAC

ACCTGCAGAGCAACCAACCAGGATGACTCCATTGACGCTGTGGCCACTCTGACTGTCCATGTGCCGCCGA

GATTCTTAGCTCGGCCAAAGAATCTCGCCGCTCAGGTTAACATGGACGCGGAGTTGGAGTGTGACGTGGC

GGGCGTTCCTAAGCCGACCATCACTTGGATGAAGAACGGTGACGTCGTCATCAGCAGTGATTACTTCCAG

ATCATCGAGGGTCGCAACTTGCGAATCCTCGGCCTGGTCAACACAGACGCTGGCATGTACCAGTGCATGG

CGGAGAACATTGTTGGAAGCGTACAAGCGAGCGCCCAGTTGATTGTGACGCAGCCAGCTGGCTCCACCCA

CTCTGTGCATTCTCTCTGGCTGACCACACCCCCCTCTCCAGAACACCAAGCGCCCTCTGTCAGCCACGAC

CTGCCGTCTGAACCCACCTACCTCGTAGCAGTCATCATCCAGGAGCGCTTTGTCACTTTGTCGTGGCAGC

CGCCCAACCACCCGGGAACCTCGGAAGTGACGTCATATGGCGTCTTCTGGAAAGAGGCTGGCTCTGACCG

TGAACGCACGCTCAATACCTCTGCCCTGGAAGCAAATGTGCAGCACCTGAAGCCCGGAACCCAGTACGAC

TTCCGAGTGTTGGGGTACAACCGGGCGGGGCCCAGTTCATCAGCCGCCGCCATGACCATCCACACCCCAG

AAGAAGTTGACGTTCCTGGTGAACCAACAGACTTAGAAGCAACCGTGCTCAGTCAGAATTCAATTAAACT

CGACTGGTCCCCTCCTGCCGACTCTCACGGTGGGATCATTAACTACAAACTCTTCTACTATGAAGTTGGA

GCTGATGGAGAGCATGAAGTGGATGTGGTTGAGACGGAATATGTGCTGGAGAAGCTGGAAGAGTATCATC

AGTATAGTTTCCGGGTTGTGGCGTGCAATCGCAATGGCGCTGGAACTAGCACCCCTGAAGCCATGGTGCG

AACCTTCTCAGATAAGCCATCAGAGCCCCCTCAGAACGTCACCATCGAGACGTCCAGTTCAACCAGCTTA

GTTTTGCGATGGGAGCCACCTCCAGAGGAAGATCAGAACGGTCGCATAACAGGATACAAGATCAGATACA

AAAAGAGGGGAGAGCAGGGTTCCTCTGTGACCACTGATGGCAACCGCAGGATGTACGCTCTCACTGAATT

AAGCAGAGGAGTTGAGTATCAATTGCGCATCCAAGCCTTAACTGTCAACGGCACAGGTCCCGCCACTGAC

TGGCTCAGCGGTGAGACTTACATGCACGATCTGGACGAGACTTCCGTCCCGGGCCAACCAAGCTCTCTGC

AAGTCCGTCCAATGACCAACTCCATCGTGGTCAGCTGGACTCCTCCGGTTGAGCAGGATATTCTCATCCG

TGGCTACATCCTCGGTTATGGCATCGGTGTCCCTGACATCTACAAACAAGTCTTGGATCCCAAGCAGCGC

TACCACACCATCAAGAACCTGAAACCATCATCACAATACGTCATCAGTCTAAGAGCGTTCAACAACATTG

GTGAGGGACGTCAGATCTACGAAACGACATTCACCCGTTACGAAGCCGTTGAAGAGGCTCCCACCCCTAT

GACGCCCCCAGTGGGATTGCGTGCCATCGTGCTCTCTTCCTCAACCATCGTACTCACATGGACTGACACC

ACCCTCGGCCGCAATCAGAGGGTCACTGACAACCGCTTTTACACTGTGCGCTATAACCCCACATCTTCAC

GTAAACACCGAGCACTCAACTCAACCAGCCTCAACACTCACATCGACGGACTCAAACCCAACACTGATTA

CGAATTCAGCGTAAAAGTTCTCAAAGGTCGAAGACAGAGCACTTGGAGTCTGAGTGCGTTCAACAAAACC

TTTGAAACTGCTCCCGCTACTGCCCCCCGCGACTTGACGCCAATTGGGGTGATTGGTCACCCCACTATGG

TACAGCTTAACTGGCAGCCCCCTAGACTGCCCAACGGTCAAATCACTGGTTACCTAGTCTTCTACACAAC

TGACGCGACTCAGAAAGATCGCGATTGGGTCGTGGAAGGAGTCCTTGGAGAGAAACTCTCAGCGAATATT

CGGGACCTCACCCCAGAAACAACTTATTACTTCAAAGTACAAGCGCGCAACAGCAAAGGCTATGGACCCA

TGTCACCGACAGTTATCTTCAGAACGCCACGCTTGGACGGTACAGGTGGCGGGATGATCGAACTACCCGA

TGACCACGGTCAACCGCATTTAACGCGAGGAGATCTGGTTCCCAAGAGCAAGGAGCAGGAGGAGGAGTCC

GGGGGAATTCCCCCCATGGTTCTGTGGATCAGCATCGGTTGCATAGGCGCCGTTTCAGTCATAGCCGTCA

TTGTAGTCACCATCATTATGTGTCGTAAACGCAATGAGAATGATCGCAGGAACAGAAAGGGTGGAGCTTA

CAAGAAAGCGCCGGTGAAGCCAGGGCCCAAAGACCTCAAACCGCCAGATTTATGGATCCACCACGAAGGC

ATGGAACTGAAGAACATTGAGAAAGCAAACAGTGATGGTACAATGGTTGTCACTCCGCTACCGAGAGATG

GGGAGGATCCTGGAGGAGTTTCATATGACCCATTAGAAGACGACCGTTACAAGAGGAATCCCATTCGTCC

AAAGCCAATCATGATTCCAGTTGACCAGGCACCTCCTCCACGGGAACCGATTGCCACGGCCATCACGAAC

GGTCATCTGGTGACCGCTGACGGCTGCACGCCAATTAGGCCCATGTATCCCAGGACCAACTCACAATTTG

CAAACAGCACTCCGCGTGTAAATGCTGGTGATATCTCACAACCGAGCTCAGGGCCCCCGTCCCCGAATGA

CCTGGACTCCCCGTCCTTTAGCAACCAGCACATCCCAATCTCCTATGAGCAGCTACAAATGTTGCCTGGA

CAACCGTCACCCTCAGCAACCACCAGCGGCTACAGCAGTGGGAGCGACAGTGGCAAAGGCACGCTGCAGC

TGCGAGGGCGGCTGCCCCACCCCCTGCGCAGCTTCACAGTGCCAGGGCCCCCAGACCCCTCCTCCCCTTC

TCCTAACCCATCTATCAACCCATCTCTCCTTAAGAAGCAGATAGTGCGACCGCAGCCTCAGCAGCCCACT

TCCCCCAACCCCACCAGTCCATACAAGAAGCCTGCTCCCCCTCCCGGCTCAAGACCAGACGTCACTCTGA

AGGCAGGTGCCAGCGATGACTTCCACGGCAGCTCAGACCTCACGATTTTTCTTCAGAAGTCATTCAGTAC

AGAAGAGTTGACAGCAGAGATGGCGAATCTGGAAGGCCTCATGAAAGACCTCAATGCCATCACGCAGCAG

GAGTTTGAGTGC

>Chicken ACAA2

ATGGCGCTGCTGAGAGGTGTATTCATTGTTGCAGCAAAGCGGACTCCTTTTGGCACCTATGGAGGGCTGC

TGAAGGGCTTGTCAGCCACTGATCTGACGGAGCATGCTGCTCGGGCTGCACTGGCTGCTGGCAAGGTCCC

TCCTGAGATCATTGACAGTGTCATTGTTGGCAATGTCATGCAGAGCTCTCCAGATGCTATTTACATTGCA

AGACATGTTGGTTTACGTGTGGGAGTCCCTGTCCCAGTTCCAGCCCTCACTGTCAACAGACTCTGTGGCT

CTGGTTTCCAGTCCATTGCTAATGGATGCCAGGAAATTTGCCTTAATGACTCAGAAGTGGTTCTGTGTGG

TGGAGCTGAAAACATGAGCCAGGCGCCTTATGCAGTTCGAAACATCCGATTTGGAACCAGATTAGGAACA

GAACTCAAGTTGGAAGACACATTGTGGGAAGGTCTAACTGACACGTACGTTAAAATCCCAATGGCAATTA

CAGCTGAAAATCTGGCTGCAAAACACAACATCACGCGAGAGGACTGCGACCAATATGCATTGAAAACACA

ACAGAGATGCAAAGCTGCTCAAGATGCTGGTCATTTTAATGCTGAGATGGCACCAATTGAAGTGAAAACA

AAAAAAGGGAAAGAAAGTATGCAAAAGGATGAGCACCCGAAACCTCAGACCACTCTGGAACAGCTGGCAA

AACTCCCACCTGTCTTTAAAAAGGATGGGACAGTCACTGCTGGGAATGCTTCAGGGGTGTGTGATGGAGC

TGGTGCAGTCATCATTGCCAGTGAATCAGCACTTAAAAAGCACAGCCTCACTCCTCTGGCAAGAATAGTT

GCCTACCACTCATCTGGCTGTGACCCTTCCATAATGGGCATTGGCCCTGTTCCTGCAATTACTGAGGTTC

TGAAGAAAGCAGGACTGACTTTGAAGGACATGGACTTGGTAGAGGTGAACGAGGCATTTGCACCTCAGTA

TCTGGCTGTTGAAAAAGTGTTGGGCCTGGACCCTGAAAAAACCAACGTCAATGGAGGTGCCATCGCTGTG

GGTCATCCTTTGGGCGCTTCAGGATCACGGATCACAGCACATCTGGTTCATGAATTAAGGCGTCGTGGTG

GGAAATATGCAGTTGGGTCAGCTTGCATTGGAGGTGGACAAGGTATTGCTCTTCTCATCGAGAACACAGC

TTGA

>Chicken C18ORF32

ATGGTGTGCATCCCTTGTATTGTCATTCCGGTTCTTCTTTGGGTCTACAAGAAATTCCTTGAGCCCTACA

TCTATCCCGTTATTGCTCCTTTCATTAAGCGTGTGTGGTCCAAGAGAGCTGTGCAAGAAACAGCCACCAA

ACAAGGCCAAGGAGGCAGTGCTGGAAATCCACAGGCACCTTCAGCCGTGCAGAGAGATAAGGGGGATGAA

TCTGGAACTTACAAGTTTGAGAGCAATGGGGTTGCAAATGGAATTGCTGCGAAGGGATCAACAGAAGTGT

CTGACAAGAAAGCAGATTAG

>Chicken CTIF

ATGGAGAACTCATCAGTGGCTTCGGCCTCCTCGGAGGCGGGCAGCAGCCGCTCACAGGAGATTGAGGAGT

TGGAGCGCTTCATCGACAGCTACGTGCTGGAGTACCAGGTGCAGGGGCTGCTCACCGACAAGACTGAGGG

CGACGGCGAGAGCGAGAAGACACAGTCCAACATCTCTCAGTGGACGGCGGATTGTAGTGAGCAGCTCGAT

GGCAGCTGCTCCCCATCCAGAGGGAAGGGCTCGTCGTCCCAGCAGCACAAGCAGAATGGCAACAAGGAGG

GCTCCCTTGACATGCTGGGCACCGACATCTGGGCTGCCAACACCTTCGATTCCTTCAGTGGTGCAACGTG

GGACCTGCAGCCTGAAAAACTAGATTTCAGCCAATTCCACAGGAAGCTCCGGAACACCTCCAAACATCCA

CTGCCCCACATCGACAGGGAAGGGCTTGGAAAAGGGAAATACGAGGATGGTGACAGCATCAATCTGAACG

ACATCGAGAAGGTCCTCCCAGCATGGCAGGGTTACCACCCGCTGCCTCACGAAGCTGAAATCGCACACAC

CAAAAAGCTGTTCAGAAGGAGGAGGAACGACAGGAGGCGACAACAGAGACTTCCTGGTGGGAACAAGTCC

CAGCAGCACGCAGACCATCAGCAAGGTGGCAGCAAACACAACAGAGACCACCAGAAGCTCTACCAAGGAG

GCCAGGCCCCTCATTCCTCTGGCAGACCAGGCCACCACGGCTACAGCCAGAACCGGCGATGGCACCACAA

CCAGAAGCACTCGCCCAACGACAAGGAAACGCACAGGAACACCAAAGAGACTGAGAATCTGAAAATTGAG

GACACCCCCACCAGCACAGGGCACAGTCCTGCGGAGACGCAGCGCAGTCCGGAGGCGGTGGAGAAGCAGT

CCCAGCCCTACAGCCCGGAGGTGGAGACGAAGCGGAAGGACAGCGTTCACGAGCGCGGTGGGGAAAGGCC

CAAGATCAACTTGCTTCAGTCTTCCAAAGACAGGCTGCGGAGGAGGCTAAAAGAAAAGGACGAAGTCACA

GTGGAAACCACCCATCCTGAAAAGAACAAAATGGACAAACTAATTGAAATCCTTAACAGCATGCGTAACA

ACAGCAGTGACGTTGACTCCAAGCTGACCACCTTCATGGAGGAGGCTCAGAACTCCACCAACTCTGAGGA

GATGCTTGGGGAGATAGTTAAAACCATCTACCAGAAAGCAGTGACAGACCGCAGCTTTGCTTCCACGGCC

GCCAAGCTCTGTGACAAAATGGCCCTCTTCATGGTGGAAGGAACCAAGTTCCGGAGCCTGCTCCTCAACA

TGCTGCAGAAGGATTTCACCATGCGTGAGGAGCTGCAGCAGCGGGACGTGGAGCGCTGGCTGGGCTTCAT

CACCTTCCTCTGCGAGGTCTTCGGCACCATGAGGAGCAGCACCGGGGAGCCCTTCCGAGTCCTCGTGTGC

CCCATCTACACCTGCCTCAGGGAGTTGCTACAATCTCAGGATGTGAAGGAGGATGCTGTGCTGTGCTGCT

CCATGGAGCTGCAGAGCACCGGCCGGCTGCTGGAGGAGCAGCTGCCTGAGATGATGACGGAGCTGCTGGC

TGCAGCCCGCGACAAGATGCTGTGCCCCTCTGAGTCCATGCTGACGCGGTCCCTCCTGCTGGAGGTCATC

GAGCTGCACGCCAACAACTGGAACCCGCTGACGCCCACCATCACGCAGTACTACAACAAGACAATCCAGA

AACTGACGGCCTGA

>Chicken DYM

ATGGGAGCAAATAGCAGCAGCATCAGTGAGCTTCCAGACAATGAGTACTTGAAGAAGTTATCAGGAGCAG

AGCCCATCTCTGAGAATGACCCCTTCTGGAATCAGCTGCTGTCCTTCAGCTTCACCACTCCCACAAACAG

TGCTGACTTAAAACTTTTGGAAGAAGCGACCGTCTCAGTCTGCAAATCTTTAGTTGAGAAGAATCCTCGA

ACAGGAAACCTTGGGTCGTTGATTAAAGTCTTTCTTTCTAGAACCAAAGAATTAAAAATATCAGCAGAAT

GTCAAAATCACCTCTTTATTTGGCAGGCTCACAATGCATTGTTTATTATCTGCTGTTTGCTGAAAGTGTT

CATCAGTCGAATGTCAGAAGAGGAACTGCAACTTCATTTTACTTACGAGGATAAGACACCTGGCTCATAT

GGAACGGAATGTGAAGACCTTATAGAAGAGCTGCTGTGCTGCCTCATCCAGCTCATTGTTGAGATTCCCC

TCTTAGATATCACATACAGCATTTCCTTGGAAGCTGTGACAACGCTCATTGTTTTCCTTTCCTGCCAGCT

CTTCCACAAGGAAATTCTGCGAGAGAGCCTCATTCACAAATACCTGATGCGTGGGCGATGTCTCCCATAT

ACCAGCAGACTTGTGAAAACTTTGCTGTACAACTTCATTAGACAAGAAAGAAGCCCTCCTCCGGGATCAC

ATGTCTTTCAACAGCAGACAGATGGAGGAGGACTGCTGTATGGGATTGCATCTGGGGTGGCAACTGGCCT

GTGGACAGTCTTTACCTTGGGCGGAGTGGGGAGTAAGGCAACGCCGCAGCTGGATCAGTGCTCCCCACTG

GCTAACCAGAGCCTGCTGCTGCTGCTGGTCCTGGCCAACCTGACTGACGCTCCGGACACACCAAACCCCT

ACAGGCAGGCAATTATGTCATTCAAGAACACACAAGACAGCAGTGCTTTCTCATCGTCACATCCCCACGT

TTTCCAGATTAATTTTAACAGCTTGTACACGGCTTTGTGTGAGCAGCAGAAGTCTGACCAAGCAACCCTT

CTGCTGTACATGCTTCTGCACCAGAATGGCAACGTACGGACATATGTGTTGGCACGGACAGACATAGAGA

ACCTTGTTCTGCCAATTCTTGAAATTCTATATCACGTTGAAGAAAGGAATTCGCACCATGTCTACATGGC

TCTCATCATCTTGTTGATCCTCACAGAGGATGATGGCTTCAACCGATCCATTCATGAAGTGATATTGAAA

AATATCACTTGGTATGCTGAGCGTGTCCTAACAGAGATCTCACTTGGGAGTCTCCTGATACTCGTTGTGA

TAAGAACCATCCAGTACAACATGACACGGACACGGGACAAATACCTGCACACGAACTGCCTGGCAGCCCT

AGCCAATATGTCAGCACAGTTCCGCTCGCTGCATCAGTACGCTGCGCAGAGGATCATCAGTTTATTTTCT

CTGCTGTCTAAAAAGCACAACAAAGTGCTGGAGCAAGCCACGCAGTCCTTGAGAGGTTCCCTCGATTCCA

ATGACTCTCCACTTCCTGACTATGCACAAGACCTGAATGTGATTGAGGAGGTGATCCGGATGATGCTGGA

GATCATCAACTCCTGCCTGACCAACTCCCTCCACCACAACCCCAACCTGGTGTACGCGCTGCTCTACAAG

CGTGATCTGTTCGAGCAGTTCCGGACCCACCCCTCCTTCCAGGACATCATGCAGAACATAGACCTGGTGA

TCAGTTTCTTCAGCTCCCGCTTGGAGCACGCCGGAGCTGAGCTGTCGGTGGAGCGCGTTCTGGAGATCAT

CAAGCAGGGAGCAGTGGCTTTGCCCAAGGACAGGCTCAGGAAGTTCCCTGAGCTGAAGTTCAAGTACGTG

GAGGAGGAGCAGCCCGAGGAGTTCTTCATCCCCTACGTCTGGTCCCTGGTGTACAACGCGGCCGTGGCAC

TCTACTGGAACCCACGCGACATCCAGCTCTTCACCATGGACTCCGGCTGA

>Chicken DYNAP

ATGGACAACCGGGCCTTCGAGATGGAACCTGAGGGCGCCGCCAGGTCCTGGAAGGGCAAAGATGGGCCGC

AAAGAGAGGCCCCCCAAAGCAACGGAACCCTCCGGAAGCTGTTTGCGCTTTGCCTTCTGGCCGGCGTGAT

GAGCACAGCTCTGAGCGTGGCCAGTTTGGCCCTGGTGTACAGCAAGGCGGGCGGCCTCGTTAGGGAAATG

AGCGCTAACGAGGGAGGGGTTGTTAACGAGGAAGGAGTTAACGAGGAGGGAGGAGGCGGCGGAGGCGCCC

GGTTTCGCTTCTTCTATCGGTTGAGTCGGACGGAGCCCCAAAAATACCCCGGCGGCCAAATCCAATGGGC

GACCTCGAGATCCAAGCGGGACCTTTACCCCGACCCCGAAGGGCTGCAATTCGGGGAGAGCCTCCGGCAG

CACCAATCCCACATGAGCGTGGCCTTGCTTCGGATCAAAGCCGGCGGCCTCCGCGTCCCCCATTGGCACT

TCAACGCCGCCGAGCACGGCTACGTGCTGCAAGGCACGGCGTGGGTCGGCGTGGCGAGCGGCGCCAACGC

CACGACCTACAACGCCACCGTGGGGCAGGCGGTGTTCTTCCCCCCCGGCGCCGTCCATTGGCTTAAGAAC

GTCGGCGGGGGGGAGTTGGCGGTGGTCCTCTTCTTCGGGAGCCACGAGGAGGTGAAGACTTTGGACGTGG

ACGAGGCTTTCTTCGGCACGGCGGAGGATATCGCGGCTCGGGCGCTTCAGCCGAGCGGAGGGCTGGAGTT

CATCAGAAGCTTCCGGAAGGCGTCGGAGGACCAAAGGGTGAACCTCCCCCCCAATTTGGCCCAGTTGGTG

CACAGCGCGGCCTACGGGCGCTCGGAAGACAGCCGGGTGTGGCGCTACTTCTACGACCTGGCGGCCTCTC

CTCTCCACCCTTTCCGGGGCGGTTCCTTTCGCTGGGCTCCTTATCGCCCTTCCAGGACCTTCATGAGCCC

CATGGAACGCATTTACACCCAATCCCTGGACGAGAGCTCCGCCCCCCTGACGTTGGCTCTCCTCCGCATC

CACCCTAACGAGCTGGGCCAGCCTCATTTGCATGGCAACGCTAACGAGCTCGCCTACGTCGTTAGCGGCC

GAGGCCGGGCCGGCCTGGTGACGGAGAAGGGGTTGAAGGTGGAGATGGAGGTCGGGGTCGGAGATGTGGT

GTTTTTCCCTGCCGGGACCCAACACTATTTGGAAGCCGGAGGCGATGAGGGACTGCTGCTGGTCGTGGCC

TACAGCACCGGCAAGAAGGAGCTGAAGACCCTTCGGATGAACCAATACTTCAAGGCCACGGCGGATCATA

TCCTGGCCCAGCTCTTCCGGAAGGAACAACGGGAGTTCCAGAGGTTCCCCCGCTCTTAA

>Chicken FECH

ATGGCTGCTGCCGGCCGGGCGGCGCGACCCCTGGTGGCGGGCGGCAGGCAGCTGAGGGTCCCGCTGCGCT

GGAGGGGGCAGGTGGCCGCGGCAGCGCCCAGCACCAAGCCGCAGGCCGAGCCAGAAACGCGGAAACCTAA

AACGGGGATCTTGATGTTAAACATGGGAGGTCCAGAAAGGCTGGATGATGTGCATGACTTCTTGCTGCGC

CTCTTCCTGGACCGGGACCTCATGACGCTTCCAGCACAAAATAAACTTGCACCGTTCATCGCTAAACGCC

GCACACCACGAATTCAGGAGCAGTACAGCAGGATTGGAGGGGGGTCACCCATCAAGAAGTGGACGGCGGT

GCAGGGAGAAGGCATGGTGAAACTGCTGGACAGCATGTCTCCTCAGACCGCACCCCACAAGTACTACATT

GGCTTCCGGTACGTGCACCCTCTGACAGAGGAAGCAATTGAGGAGATGGAGGATGACGGCATCGAGAGAG

CCATCGCCTTCACGCAGTACCCGCAGTACAGCTGCTCCACCACAGGAAGCAGCTTAAATGCCATTTATCG

CTACTACAATAAAAAGGGGAAGAAGCCAAAGATGAAGTGGAGTATCATTGACCGATGGCCCACACACCCC

CTGCTCATCCAGTGCTTTGCCGATCACATACAGAAGGAGCTGGACCTGTTCCCACCTGACAAAAGGAAAG

ATGTCGTCATCCTTTTCTCAGCCCACTCGCTCCCCATGTCTGTGGTGAACCGTGGTGATCCGTATCCACA

AGAAGTGGGAGCTACTGTCCAGAGAGTCATGGAGAAGCTGAACCACTCCAACCCCTACCGGCTGGTCTGG

CAGTCCAAGGTTGGGCCAATGCCTTGGCTTGTTCCACAAACAGACGAGACGATTAAAGGACTGTGCCAAA

GAGGAAAGAAGAACATGTTGTTGGTCCCCATAGCATTTACAAGTGACCACATTGAAACGCTTTACGAACT

GGATATTGAGTATGCCCAAGTTTTAGCAAACGAGTGTGGCGTTGAGAACATCAGAAGAGCGGAGTCACTT

AATGGAAACCCACTGTTCTCCAAGGCTCTGGCAGACTTGGTCTGCTCACACATCCAGTCCAATGAGATCT

GCTCCAAGCAGCTGACGCTCTGCTGCCCGCTCTGCGTGAATCCTGTCTGCAGGGAGACCAAAGCCTTCTT

CACCAACCAACAGCTGTGA

>Chicken LIPG

ATGAGCGGTGCGCTTGGCTCAGGCCCCGCCCCATCGGGCCATGAGCTCCGCCCCGCGGGGGATTTTAAGG

CGGCGACTGCGGTGCGCCTGCGGAGCGCCATGAGCTGGGCAGTGCTGGTACTGCTGTGCGCCGGGCTGGG

CGCCTGCCTCGCCGCGGGGACCACAGGTGCCCCCGGCGCGCCCCCTACCCCGCGGCTGCGAGTGAAGTTC

GGGCTCCGGCGTTCGGCCGATGCCGAGGATGAGGGCTGTGCGCTGGAACTGGGCAGCGAGAGGAGCCTGG

AGGAGTGCGGCTTCAACGCGACGGCCAGGACCTTCCTCATCATCCACGGGTGGACGATGAGTGGCATGTT

TGAGACCTGGCTGGGCAGCTTGGTCTCAGCCCTGCAGGAGAGGGAGAAGGATGCCAATGTGGTGGTGGTG

GATTGGCTTTCTCTTGCCCACCAACTCTACACCGATGCAGTGAACAACACACAGGTTGTGGGGAAGAGCA

TAGCGAGGCTGCTCAACTGGTTGCAGGAGATCCCTCTCTTTAAGCTGGAGAATGTCCATCTTATTGGGTA

CAGCCTGGGTGCTCATGTTGCTGGCTTTGCTGGTAACCATGTCCATGGGACAATAGGCAGAATCACAGGC

TTGGACCCTGCTGGCCCTATGTTTGAAGGAGTAGACCCCAGCAGGCGCCTTTCACCTGATGATGCTGCCT

TTGTGGACGTCCTTCACACCTACACGAGGGAAACACTGGGTGTCAGCATTGGGATCCAGATGCCTGTGGG

CCATGTTGATATCTATCCCAATGGAGGGGACTTCCAGCCTGGCTGCGGACTGAGCGATGTCCTGGGAGCC

ATTGCCTATGGCACAATTGGTGAAGTTGTTAAGTGTGAGCACGAGCGGTCTGTGCACCTGTTTGTGGACT

CCCTCGTGAACCAGGATAAACAAAGCTTCGCGTTTCAATGTACTGATTCCAGTCGCTTCAAGAAGGGCAT

CTGCCTGAGCTGCCGCAAGAACCGCTGCAGCGGCATTGGCTACAACGCCAGGAGGACACGGCACAAAAGG

AACAGCAAGATGTACCTAAAAACAAGAGCTGACATGCCATTCAAAGTCTACCACTATCAGATGAAGATGC

ATGTCTTCAGCTACAATAACGCGGGGGAGGCTGACCCAACCTTCTCTGTCACTCTCCATGGCACCAACAG

AGACTCCGAACCCCTCTCTTTGGAAGTGCTTGAGCAAATTGGCCTTAATGCTACCAACACCTTCCTGGTC

TATACTGAAGAGGACATGGGTGAACTTCTAAAAATAAAGCTCACCTGGGACGGAGCATCTCAGTCGTGGT

ACGATCTGTGGAAGGAGCTAAGGAGCTACTGGTACCGACCTGCTAAGTCCTCCCAGGAGCTGCACATCCG

ACGGATACGTGTGAAATCTGGGGAGACGCAGCAGAGGTTTGCTTTCTGCGTGGAGGACTCCCAGCTGACC

AGCATATCTCCTGGCAAAGAGCTCTGGTTTGTGAAGTGCACAGATGTATGGAAAAGAAGATCTGTCTCAA

ACACACTCTGA

>Chicken MBD2

ATGGATAAACAGGGCAGGACGGACTGCCCGGCCCTTCCACCGGGTTGGAAGAAAGAAGAGGTGATCCGTA

AGTCGGGTTTAAGCGCAGGCAAAAGCGACGTGTATTACTTCAGTCCGAGCGGGAAGAAGTTCCGAAGCAA

ACCTCAACTTGCCCGTTATTTGGGGAACGCCGTGGATTTGAGTTGTTTCGATTTCCGCACGGGCAAAATG

ATGCCGAGTAAATTACAGAAGAATAAACAGAGATTGAGGAACGAATCGCTTCATCCCAATAAGGGCAAAC

CGGATCTCAACACGGCCCTCCCCATCCGGCAGACGGCGTCCATCTTTAAGCAACCCGTCACCAAAGTCAC

CAACCACCCCGACAACAAAGTCCGTTCCGACCCACAACGCCTCGCAGACCAACCCCGCCAGCTCTTCTGG

GAGAAACGACTTCGGGGTCTGAGCGCTTCCGATGTGGGGCAGGAGATCCTCCGGGCTATGGAGCTGCCCA

GGGGGCTGCAAGCCCTTGGTCCCGTCCCGGACGACGTCACTTTGCTATCGGCCGTCGCCAGCGCTCTCCA

TGTCGGTTCTGTTCCCGTTACCGGACAGCTTTCGAGTGCTGCAGAGAAGAATCCGGCGGTGTGGCTCAAT

TCTTCCCAACCTCTTTGCAGAGCTTTCGTTGTCACCGACGACGACATCCGGAAGCAAGAGGAGCGGGTGA

GGCGCGTGAGGAAGAAACTGGAAGAAGCACTTCTGGCCGGCGACCCGGCGGGGTCACGGGGTCAAGACGA

GTGA

>Chicken ME2

ATGCTGTCCCGCTTCCGCCTGACCCTACAACGCTGCGTCCACACCAGGGAGAAGGGGAAACCCCTCATGC

TCAACCCTCGTACCAACAAGGGCATGGCTTTCACGTTGTTGGAGCGGCAGATGTTGGGCCTCCAAGGCCT

CCTCCCCCCCAAAATCGAGACGCAGGACATCCAAGCCTTACGCTTCCACAAGAACATGGCCAAGATGAGC

GACCCCCTCCAGAAGTACATTTACCTCATGGGGATCCAGGAGAGGAACGAGAAGCTCTTCTATCGGGTCC

TCCAGGACGACATCGAGAGGCTGCTGCCGGTTGTTTACACGCCAACAGTCGGACTCGCCTGTACCCAATA

CGGCCACATCTTCCGGCGGCCCAAGGGTTTGTTTATCTCCATCTCGGATCGGGGTCACATCAGATCGGTC

GTTAACAACTGGCCGGAGAACGACGTGAAGGCCGTGGTCGTCACCGACGGCGAACGCATTTTAGGATTGG

GGGACCTCGGAGTTTATGGGATGGGGATCCCCGTGGGGAAACTCTGCCTTTACACCGCCTGCGCCGGGAT

CCACCCCCACAAATGTCTGCCTGTCTGCATCGACGTCGGCACCGACAACACCAGCCTCTTAAAGGACCCC

TTCTACATGGGGCTGTACCAAAAACGCGACCGCTCGCCGGCCTACGACGACCTCATCGACGAATTCATGG

AGGCCATCACCGACCGGTACGGTCAGAACACCCTGATCCAATTTGAGGACTTTGGGAACCACAACGCTTT

CCGTTTCCTCCGCAAGTACCGGGAGAAGTACTGTACCTTTAACGACGACATTCAAGGTACAGCCGCCGTC

GCCTTGGCCGGCCTATTGGCAGCTCAGAAAGCAACGGGGAAACCCATAGCAGAACACAAAGTGCTGTTCC

TCGGGGCCGGAGAGGCGGCTCTGGGCATCGCCAACCTCATCGTTATGGCCATGGTGGAGAATGGGGTTCC

CGTGGAGGACGCGCGGAGGAGGATTTGGATGTATGACAAACACGGCTTGGTCCGCCAGGGCCGGGAAGAG

AAAGTGGACTCCAACCAAGAGCCGTTTGCACACCCAGCTCCGGATTCCATCCCGCACAGCTTCGAGGAGG

CGGTGACGTTGTTGAAGCCGTCGGCCATCATCGGTGTAGCGGGCGCCGGACGCCTCTTCACCCCTTCAGT

CCTCCAAAAGATGGCCGCCATCAACGACCGCCCCATCATCTTCGCCCTCAGCAACCCTACAGCCAAAGCG

GAGTGTACAGCCGAAGAGGCCTACACCTTAACCGAGGGTCGCTGCCTCTTCGCCAGCGGGAGCCCTTTCG

ACGTCGTGACCCTCCCGGATGGGCGCAGCTTCAAAGCGGGCCAAGGGAACAACGCTTACATCTTCCCAGG

GGTGGCGTTGGCCGTCATATTGAGCAGCGTGAGGCACATCAGCGACCGCGTCTTCCTGGAGGCGGCCAAG

GCCCTATCGGAGCAGCTGAGCCCCCCGGATTTGGCGCAGGGCCGCCTCTACCCCCCGTTATCCCACATCC

GGGAGGTGTCCGTCTGCATCGCCGTCCGGGTGATGGAGTTCCTCTACGCCAACGGGATGGCCTTCCATTA

CCCGGAACCCCCCGAGAAGGAACCGTACGTCCGTTCCAAAGTGTGGACTTCCCAATACGAGTCGTTCGTC

CCCGACGTTTACCAATGGCCCCGAAACGCCCCGGCCTAA

>Chicken NARS

ATGGCGGGGGAGGTGATCGGCAGGACGGCGGCTCTGGCGCTGGAGGAGTTGTATGTTTCTGAACGAGAGG

GCAGCGATTCCACCGGCGATGGGACGCAGAAGAAACCATTCAAAACTGTTTTAAAGGCTTTGATGACAGC

AGGAAAGGAACCATTTCCTACTATTTACGTGGATTCACAGAGGGAAAATGAGAGATGGGCCATTATTTCA

AAGTCACAGATGAAAAATGTCAAAAAACTGTGGCACAGGGAACAAATGAAGAATGAGGCAAAGGAGAAGA

AAGAGGCAGAAGATCTCTTGAGAAGAGAGAAGAACCTGGAGGAAGCCAAGAAAATTATTATCAAGAATGA

TCCTAGTCTTCCAGAGCCAAAATGTGTAAAGATTGGAGCTCTGGGGGCTTACAGAGGCCAGAGAGTAAAG

ATTTTTGGCTGGATTCACAGATTACGTAGGCAAGGAAAGAATTTGATGTTCATTGTTTTGAGAGATGGCA

CAGGTTTTCTTCAGTGTGTCCTTTCAGATGCACTGTGTCAGTGTTACAACGGGCTCCTTCTCTCTACAGA

GAGCAGTGTTGCGGTATACGGTATGCTGAACCTTGTTCCTGAGGGCAAGCAGGCTCCAGGAGGCCATGAG

CTGAACTGTGACTACTGGGAGCTTATTGGTCTGGCCCCAGCAGGAGGGGCTGACAATCTCCTCAATGAGG

ATTCGGAGGTTGATGTGCAACTTAACAACAGGCACATGATGATTCGAGGCGAGAATATGTCCAAAATCTT

CAAGGTGCGCTCCATGGTGGTACAGGCCTTCAGGGATCATTTCTTTGCCAATGGATATTATGAAGTCACA

CCACCAACATTAGTCCAGACACAGGTGGAAGGAGGTTCAACCCTATTCAAGCTGGATTATTTTGGTGAAG

AGGCTTACTTAACACAGTCATCTCAGCTCTACCTGGAGACCTGCATTCCAGCACTAGGAGATGTTTTCTG

TATTGCTCAGTCGTACAGGGCTGAGCAATCCAGGACACGCAGACACTTGGCTGAATACACTCACATTGAA

GCTGAATGCCCTTTTATAAGTTTTGAAGACTTGCTGAACCGTTTGGAGAGCTTAGTTTGTGATGTAGTTG

ACAGAGTCTTGACATCACCTGCATCAGCCTTACTGCTGGACCTCAACCCGAACTTCAAGCCCCCAAAACG

TCCTTTCCGACGAATGAACTATGCTGAAGCAATTGAGTGGCTAAAGGAACATGATGTGAAAAAGGATGAT

GGTACTTACTATGAGTTTGGGGAAGATATTCCTGAAGCTCCTGAGAGGTTCATGACAGACACCATCAATG

AGCCAATCCTGCTGTGCAGATTTCCTGCAGAGATCAAGTCCTTCTACATGCAGCGCTGTCACGATGATTC

CCGGCTTACAGAATCTGTTGATGTGTTGATGCCTAATGTTGGCGAAATTGTTGGAGGCTCTATGCGTATC

TGGGACAGTGAGGAGCTACTGGAGGGCTACAAGAGAGAAGGCATTGATCCCACACCATACTACTGGTACA

CTGATCAGAGAAAGTACGGTACGTGTCCTCATGGCGGATATGGTTTGGGATTGGAGCGATTCCTGACCTG

GATTCTGGATAGGCACCACATCCGAGATGTCTGTCTGTACCCTCGCTTTGTCCAGCGCTGCAAACCGTAA

>Chicken NEO1

ATGCTGACGCCCAGCAAACTGTCACGGCTGCCTGGGATTGGTGACATCTCGAGTGACACGTGTTGGATCG

TCAGATCAGAGTGGGAGCAGCACAGCTTTGGGAGCCTAAAAATAGGGTCCGTAGTGAGAACCTTCACTCC

TTTTTACTTCCTGGTGGAGCCGATGGACATTCTGTCAGTTCGTGGGGCATCTGTTATAATGAACTGCTCA

TCTTATTGTGAAACTCCTCCGAAGATCGAATGGAAGAAAGATGGGACTCTTCTAAACTTGGTCTCAGATG

ACCGTCGCCAGTTGCTGCCAGATGGGTCTTTATTAATAAACAGCGTGGTGCATTCCAAGCATAATAAACC

CGATGAAGGATATTATCAGTGCGTGGCAACTGTGGAAAGCCTGGGGAGCATTGTAAGCAGAACAGCAAAG

CTCACGGTGGCAGGTCTTCCCAGGTTCACCAGCCAGCCGGAGCTATCGTCTGTCTATAAAGGAAACAGTG

CAATCCTGAACTGCGAAGTGAACGTTGACCTCGCGCCGTTTGTGAGGTGGGAGCAGGACCGGCAGCCCCT

CTCCCTGGATGACCGTGTCTTTAAGCTGCCGAGCGGAGCGCTTCTTATTGGTAACGCTACCGATACAGAT

GGAGGATTCTATCGCTGCGTCATTGAAAGTGGTGGGACCCCCAAATACAGTGAGGAGGCAGAGCTCAAAA

TTCTTCCAGATCCAGAAGAGCCCCAGAGCTTGGTCTTCGTGAGGCAGCCATCTTCACTTACTAAAGTTAC

TGGGCAAAATGCGGTTTTTCCATGTGTTGCTGGAGGATTTCCAACTCCGTATGTCAGATGGACAAAAAAT

GGAGAAGAGCTAATCACAGAAGACTCCGAGAGGTTTGCGCTGCGTGCGGGGGGCAGCCTGCTCATCAGTG

ATGTTACGGAAGAGGACGTTGGGACGTACACCTGCATAGCAGATAACGAGAACGAGACGATTGAAGCTCA

GGCAGAGCTTGCAGTACAAGTTCCTCCTGAGTTTCTGAAGCGACCCGCAAACATTTATGCTCATGAATCT

ATGGACATTGTCTTCGAGTGTGAAGTGACTGGAAAACCTACTCCAACTGTGAAATGGGTCAAGAATGGAG

ACGTGGTGATTCCAAGCGACTACTTCAAAATCGTTAAAGAACATAATCTGCAAGTTTTGGGTCTGGTGAA

ATCAGATGAAGGATTCTATCAGTGCATTGCAGAAAATGATGTTGGAAATGCACAGGCTGGAGCCCAGCTG

ATAATACTTGACCTCGATGTTGCCATCCCAACATTACCTCCCACTTCACTGACCAGTGCCACTAATGACC

ATCTAGCACCAGCTACAACGGGACCACTGCCTACAGCCCCTCGGGACGTCGTGGCCACCCTCGTCTCCAC

TCGCTTCATCAGGCTGACGTGGCGGACACCGGTGTCAGACCCGCAGGGAGACAACCTCACCTATTCAATC

TTCTACACTAAGGAAGGTATCAACAGGGAACGTGTTGAAAACACCAGTCGTCCTGGAGAGACACAAGTGA

TGATCCAAAACCTGATGCCAGAAACAGTTTATGTCTTCAGAGTTGTGGCTCAGAACAAGCACGGCCATGG

AGAGAGCTCAGCACCACTGAAGGTGGCCACGCAGCCTGAGGTTCAGCTGCCTGGTCCAGCACCCAATATC

CGAGCGTACGCCGGCTCCCCCACCTCTGTCACCGTCATGTGGGAAACACCACTGTCTGGCAATGGGGAAA

TCCAGAACTACAAGCTCTACTACATGGAGAAGGGGCAGGACAGTGAGCAGGATGTTGATGTAGCAGGACT

CTCCTACACCATTACTGGATTAAAGAAGTACACTGAGTATAGTTTCCGAGTGGTGGCTTACAATAAACAC

GGCCCTGGCGTCTCTACCCAAGATGTTGTTGTGCGGACGCTGTCAGATGTTCCCAGTGCTGCACCACAGA

ATCTAACGCTGGAGGCACGGAATTCCAAGAGCATCATGCTGCACTGGCAGCCTCCTCCTGCAGGGACACA

CAGCGGTCAAATCACCGGCTACAAAATCCGCTACCGCAAAGTGTCCCGTAAGAGTGATGTGACCGAGAGC

GTCGGGGGGACACAGCTTTTCCAGCTCATTGAAGGTCTTGAACGGGGCACGGAGTACAACTTCCGGATAG

CTGCCATGACTGTGAATGGCACTGGGCCAGCTACTGACTGGGTGTCAGCAGAAACATTTGAGAGCGATCT

GGATGAAAGCCGTGTTCCTGAAGTTCCGAGCTCCTTACACGTCCGTCCTCTTGTCACCAGTATTGTGGTG

AGCTGGACTCCACCTGAGAACCAGAACATCGTGGTGAGAGGCTATGCCATAGGGTATGGCATCGGCAGTC

CTCACGCGCAGACCATCAAGGTGGACTACAAACAGAGATACTACACCATTGAAAACTTAGACCCGAGCTC

GCACTACGTTATAACTCTGAAAGCATTCAACAATGTTGGTGAAGGAATTCCTCTCTATGAGAGCGCAGTG

ACCAGGCCTCATTCAGACACTTCCGAAGTTGATTTGTTTGTTATTAATGCTCCATACACTCCAGTGCCAG

ATCCATCTCCCATGATGCCACCGGTGGGAGTTCAGGCTTCCATCCTGAGCCATGACACCATACGGATCAC

TTGGGCAGACAACTCTCTGCCAAAGAACCAGAAGATCACGGATGCTCGCTACTACACAGTTCGCTGGAAA

ACCAATATTCCTGCAAATACAAAGTACAAGACGGCCAACGCGACCACGCTGAGCTATTTAGTGACTGGGT

TAAAACCAAACACGTTGTATGAATTCTCCGTGATGGTGACTAAAGGGCGAAGATCGAGCACGTGGAGCAT

GACAGCACATGGGACAACTTTTGAGCTAGTTCCTACTTCACCTCCCAAGGATGTGACTGTGGTGAGCAAA

GAGGGAAAGCCTCGGACCATCATTGTGAACTGGCAGCCTCCGTCCGAAGCCAACGGCAAAATTACAGGAT

ACATCATTTACTACAGTACGGACGTGAATGCTGAAATACACGACTGGGTTATTGAGCCCGTTGTGGGGAA

CAGGCTGACCCACCAGATACAAGAGCTGACCCTCGACACGCCGTACTACTTCAAAATTCAGGCCCGCAAC

TCAAAGGGCATGGGGCCCATGTCTGAGGCCGTCCAGTTCAGGACCCCTAAAGCTGAGTCCTCAGATAAAA

TGCCTAATGATCAAGCTTCGGGATCTGCAGGGAAAGGAAGCCGCCCAGTGGACGTGGGGCCGGACTACAA

ACCCCCGCTCAGCGGCAGTAACAGTCCCCATGGAAGTCCGACTTCTCCCTTGGATAGCAACATGCTTCTT

GTCATCATAGTGTCTGTTGGGGTCATCACCATTGTGATAGTGGTGATCGTCGCCGTTTTCTGCACTCGTC

GTACCACTTCTCACCAAAAAAAGAAACGAGCTGCCTGCAAATCAGTGAACGGCTCCCACAAGTACAAAGG

GAATTCCAAAGATGTCAAGCCTCCTGACCTTTGGATCCATCATGAAAGACTGGAGCTAAAGCCCATTGAT

AAATCTCCAGATCCCAATCCAATCATGACAGATACCCCAATCCCTCGCAACTCCCAGGACATCACCCCAG

TTGATAATTCCATGGACAGCAATATTCACCAAAGGCGGAACTCCTACAGAGGGCATGAGTCGGAGGACAG

CATGTCCACGCTGGCAGGAAGAAGGGGGATGAGGCCCAAGATGATGATGCCTTTTGATTCTCAGCCGCCT

CAGCCTGTGATTAGTGCCCATCCCATCCATTCACTCGATAACCCCCACCATCATTTCCACTCCGGCAGCC

TCGCTTCTCCAACTCGCAGCTATCTCCATCACCAGGTCAGCCCGTGGCCAGTGGGCACATCCATGTCCCA

TTCAGACAGGGCCAATTCCACAGAATCCGTTCGGAACACACCGAGCTCTGACACCATGCCGGCCTCCTCG

TCCCAGCCGTGTGCGGACCACCAGGACCCCGACAGCAGCTCAGGCGCTTACCTGGGCAGTGCTCAGGAGG

AGGACGCGGCCCAGAGCCTGCCCACAGCTCACGTGCGTCCATCCCACCCCCTGAAGAGCTTTGCAGTGCC

AGCAGTGCCAGCAGCCGGCTCTGCCTATGACCCCACGCTGCCCAGCACCCCGCTGCTGACCCAGCAAGCT

CCCTCCCATCCAGTTCACTCGGTGAAGACTGCATCGATTGGGACTTTAGGAAGAACACGGCCTCCCATGC

CTGTGGTGGTTCCCAGCGCCCCTGACGTGCAGGAGACCACCAGGATGCTCGAGGACTCGGAAAGCAGCTA

TGAACCAGATGAGCTGACCAAAGAGATGGCCCACCTGGAAGGACTGATGAAGGACCTTAATGCCATCACT

ACAGCATGA

>Chicken ONECUT2

ATGAGGAGCGGCCGCGGCGCCTACCGATGCCTCGCCGCCGAGCCCGCTGCCTGCGCCATGAACCCCGAGC

TGGCTCTGGAGCCGCTGGGCAGCCTGCATGAGCCCGAGCTGCTGGGCAGCCCCGGCCCGCACCACGGCGG

CCGAGGCGGGGGATCGCTGCGGGTGCCGCCGCCCCAGGAGCTGCCCGGCGGCGGCGGCGGCGGCGGAGGC

GGCGGGGGGGGGCGGGCGGCCATGGTGCCGGGCATGGCCCCGCTGCTGGACGGCGCCGAGTTCCGGCCCG

AGCTGTCGGTGCCGCTGCACCACGCTATGAGCGTGCCCTGCGAGCCCTCGCCGCCCGGCATGGGCATGAG

CAGCACCTACACCACGCTGACGCCGCTGCAGCCGCTGCCGCCCATCTCCGCCGTGTCCGACAAGTTCCAC

CACCCGCACCCCCACCCGCACCACCACCACCACCACCACCACCAGCGCCTGGCGGGCAACGTCGGCGGCG

GCTTCGCGCTGATGCGGGACGAGCGCGGGCTGCCCGCCGTCAACAACCTCTACGGGCCGTACAAGGAGGT

GCCGGCCGTGGGGCAGAGCCTGTCCCCGCTGGGCGCGGGGCTGGCCCCCCTGCACGGCGCTCAGCAGGGC

CTGCACGGCTACGGGCCGCCCCCGCCCCCGGGCCACGACAAGATGCTGGGGGCCGGCTTCGAGGCCGTGC

CGCACCTGCCGCGGGGGCTGCCGGCGGCCCCGGCCCCGCTGCCGCACCTCAACGGGCAGCACCCCCCTGC

CCCGCCGCCCCACGCGCTGCCCGCCGCCCGCGACCGCCCGCCCGCCGCCGCCCCCGCGCAGCAGCTGGAG

GAGATCAACACGAAGGAGGTGGCCCAGAGGATCACGGCGGAGCTGAAGCGCTACAGCATCCCCCAGGCCA

TCTTCGCCCAGCGGGTGCTGTGCCGCTCTCAGGGCACCCTCTCGGACTTGCTACGGAACCCTAAGCCTTG

GAGTAAACTGAAATCCGGCAGGGAGACCTTCCGGAGGATGTGGAAGTGGCTGCAGGAGCCGGAGTTCCAG

AGGATGTCAGCCTTAAGGCTCGCAGCATGCAAACGTAAAGAGCAGGAACCGAACAAGGAAAGGAACAACT

CCCAGAAGAAATCCCGGCTGGTTTTCACTGACCTCCAGCGCCGAACACTTTTCGCCATCTTCAAGGAGAA

CAAGCGCCCGTCCAAAGAAATGCAGATCACCATCTCCCAGCAGCTGGGCCTGGAGCTCACCACCGTCAGC

AACTTCTTCATGAATGCCCGTCGACGCAGCCTGGAGAAGTGGCAGGATGACCTGAGCACTGGGGGCTCCT

CCTCAGCCTCCAGCACCTGTACCAAAGCATGA

>Chicken RPL17

ATGGTGCGCTACTCGCTGGATCCAGAGAACCCTACCAAGTCATGCAAGTCCAGGGGCTCCAACCTGCGTG

TGCACTTCAAGAACACTCGTGAGACTGCCCAGGCCATCAAGGGCATGCACATCCGCAAGGCCACCAAGTA

CCTGAAGGATGTGACCCTGAAGAAGCAGTGTGTTCCCTTCCGTCGCTACAACGGAGGAGTTGGTCGATGT

GCCCAGGCCAAGCAGTGGGGCTGGACACAGGGACGCTGGCCCAAGAAGAGCGCGGAGTTCCTGCTGCACA

TGCTCAAAAATGCAGAGAGCAATGCTGAGCTCAAGGGTCTCGATGTGGATTCTCTGGTGATTGAACACAT

CCAGGTGAACAAGGCTCCCAAAATGCGCCGGCGCACCTACAGAGCTCACGGGAGGATCAACCCCTACATG

AGCTCCCCGTGCCACATTGAGATGATCCTCACCGAGAAGGAGCAAATCGTTCCCAAGCCAGAGGAAGAAG

TTGCTCAAAAGAAAAAGATATCCCAGAAGAAGCTGAAGAAGCAAAAACTGATGGCTCGCGAGTAA

>Chicken SKA1

ATGGAGGCCGAAGGGTTGGAGGCTCTGAGCCGCCATTTGGCCGCCAAGATCGGGCTCATTAAGCGGCTGC

TGCAATTAAGAGGGCTGGGGAGGAGGGAGATGCTGGCCGAGGTGGGCATGGAGGTGGCGGTGCTGCATGG

GCTGCTGGGCCGGATGGAAGAGGAAGTCAATCAACGGCGGCAATTAGCGGCCGCCCTGCAGGAGATGCGG

AAGAGGGCGGAGAAGGAGAAGTGGGAGGCGGAGAGGCTGCAGCTCGTTAAGAAGGCCGCCAAGGAGGCGC

CGCTCGTTAACGACGCCCCGCTAATTAGCGACGAGGAGTTCGAGAGCGTTCCCGGCTACATGAGGGGCCG

CCTGACCCTGGCCCAAACCAACGCCGCCCTCCGCGCCCTCCACGCCGCCGCCGCCTCCAAATACCGCCTC

CTCCGCCACCCCCCCAAATCCCTCCCCGCCTCTTCCCGCTCCCTTTGCCACCGCTTCCGGGAAGAGGAAA

CCAAAGAGACTCAAGGGCTCACCTTCGTGGTGGAGGCCGACCTGAAGGAGTTCACCCAGCTGAAGGTGGA

CCGAACCTTCCATCGCATCGTCGGCGTCCTCCGCCATTGCCGACGGCTTCGGGAGGTCAGGGGGGCCCGA

TTGGTTCGTTACGTCCTCCCTTAA

>Chicken SMAD4

ATGTCCATTTCCAACCCGCCGAGCAGCAACGACGCGTGCCTCAGCATCGTCCACAGCCTCATGTGTCACC

GGCAGGGGGGCGAGAGCGAGACGTTCGCCAAGCGGGCCATCGAGAGCCTGGTGAAGAAGCTGAAGGAGAA

GAAAGACGAGCTGGATTCGCTCATCGCCGCCATCACCACCAACGGGGCCCACCCGAGTAAATGCGTGACC

ATCCAACGGACGTTGGACGGGAGGCTGCAGGTGGCGGGGAGGAAAGGTTTCCCTCACGTCATCTACGCCC

GCCTGTGGCGTTGGCCCGACCTGCATAAGAACGAGTTGAAGCACGTCAAGTATTGCCAGTACGCCTTCGA

TCTCAAGTGCGATAGCGTGTGTGTCAATCCCTATCACTATGAGAGGGTCGTCTCGCCCGGCATCGATCTG

TCCGGGCTGACCCTGCAGAGCTCTGCCCCGTCCAGTTTGTTGGTGAAGGACGAATACGTCCCCGACTTCG

AAGCTCAGCCCTCCCTCTCCTCCACCGACGGCCATTCGGTTCAGACCATCCAACACCCACCGGGCGGTAG

AGCTCCCGCTGAGGCCTACAGCGGCGCTACGGAAACCGGAGCCGCCAACTTCCCTTCTATCCCGGTGGCG

TCCAGCAGCCAACCCCCCACCGTATTACCGGGCGCCCATAACGACTCTCTCCTCCCGTTGGCTCCGGGAC

CGCAGGCGGCGGCGGCGGCGGCGGCGGCGGCGGCGGCTCAGAACGGTTTCCCGGCCCAACCGGCGACCTA

TCACCACAACAGCGGCGGTAACGGAGGGTGGAGCGGAAGCCGCGGCGCGGCGTATCCTTCCGCCATCCCT

CACCATCAGAACGGTCACCTGCAGCACCACCCCCCCCTCCACCCGGGGCATTACTGGCCGGTGCACAACG

AGCTCGCCTTCCAACCGCCCATCTCCAACCATCCCGCCCCGGAATATTGGTGTTCCATCGCCTATTTCGA

GATGGACGTTCAGGTCGGGGAGACCTTTAAGGTCCCTTCCAGTTGTCCCGTGGTCACCGTGGACGGTTAC

GTGGATCCGTCGGGAGGCGACCGCTTCTGTTTGGGGCAGCTGTCCAATGTCCATCGCACCGAAGCCATTG

AGAGAGCACGGTTGCACATCGGGAAGGGGGTGCAGCTGGAGTGCAAAGGGGAAGGGGACGTGTGGGTGAG

GTGCCTGAGCGACCACGCCGTGTTCGTGCAAAGCTATTACCTGGATCGGGAAGCCGGGAGGGCGCCGGGG

GACGCCGTGCACAAGATCTATCCCAGCGCTTATATTAAGGTGTTTGATCTGCGACAGTGCCACAGACAGA

TGCAACAACAGGCAGCCACGGCGCAGGCAGCGGCAGCGGCGCAGGCAGCGGCCGTAGCCGGGAATATCCC

CGGGCCGGGCTCTGTGGGTGGCATCGCGCCGGCAATTAGCCTCTCCGCCGCCGCCGGCATCGGAGTCGAC

GACCTCCGCCGCCTCTGCATCCTCCGCATGAGCTTCGTCAAAGGTTGGGGCCCCGATTACCCCCGGCAGA

GCATTAAGGAGACGCCGTGTTGGATCGAGATCCACCTCCACCGCGCCCTCCAACTCCTCGACGAGGTCCT

GCACACCATGCCCATCGCCGACCCGCAGCCCTTGGACTGA

>Chicken SMAD7

ATGTTCAGGACCAAACGCTCGCTCCTCGTCCGACGGCTCTGGCGGAGCCGCGCACCCGGCGGCGGAGAGG

AGGAGACGGGCGACGGCGGTGCGCCGGCCGAGCCTCGGCCGCACTCATGCGGCGGGGGGGGCCGGGGGTG

CTGCCCGGCCAAACCCCCCCGCGGGGGCGGCCGGGGGGCGGCGGAGGGCGAGCTGAAGGCGCTGACCCAC

GCCGTGCTGAAGCGCTGCAAAGAGCGGCAGCTGGAGGGGCTGCTGCGCGCCGTCGAGTCCCGCGGGGCGG

CCCGCACCCCCTGCGTGCTGCTGCCGGCGCGCGGAGAGGCGCGGTTGGGCGCGCAGCGGGACGCGCTGCC

CGCGCTGCTGTGCCGGGTGTTCCGCTGGCCCGAGCTGCGGCACGGCGCGCCCCTCAAGCGGCTCCGCGGC

TGCTGCCAGGCCGACGGCGCGGCGCCCACAGAGCTCGTCTGCTGCAACCCGCACCACCTCAGCCGTCTCT

GCGAGCTCGAGTCTCCTCCTCCGCCCTACTCCAGATATCCCATGGATTTCCTCAAGCCGACGGCAGGTTG

TCCAGACTCTGTGCCTTCCTCCACTGAAACAGGGGGAACTAATTGTCTAGCCCCTGGGGGGCTCTCAGAT

TCCCAAGTTCTCCAGGAGTCAGGGGATCACTCACACTGGTGCGTGGTGGCATACTGGGAAGAGAAGACAC

GCGTGGGTCGGCTGTACTCTGTCCAAGAGCCCTCCCTGGATATCTTCTATGATCTACCTCAGGGGAACGG

CTTCTGCCTTGGGCAGCTCAACTCGGACAACAGAAGCCAGCTGGTGCAGAAGGTGCGCAGCAAGATTGGG

TACGGCATCCAGCTCACCAAGGAGGTGGACGGCGTGTGGGTCTACAACCGCAGCAGCTACCCCATCTTCA

TCAAGTCGGCCACACTGGACAACCCCGACTCCAGGACGTTGCTGGTGCACAAAGTGTTCCCGGGTTTTTC

CATCAAGGCTTTTGATTACGAGAAGGCGTACAGCCTGCAGAGGCCGAATGACCACGAGTTCATGCAGCAG

CCATGGACTGGATTTACCGTGCAGATCAGCTTTGTGAAGGGCTGGGGCCAGTGCTACACCAGGCAGTTCA

TCAGCAGCTGCCCGTGCTGGTTAGAGGTTATTTTTAATAACCGATGA

>Chicken ST8SIA3

ATGGTGCGGGTGGCCAGCGTGCTGGGGCTGGTGATGCTCAGCATCGCGCTGCTCATCCTCTCCCTCATCA

GCTACGTCTCGCTCAAGAAGGACAACATCTTCGGCGCACCCCGCGCCGCCGGCCCGGGGGGGCCCCGCAT

GTACATGTTCCACGCGGGATTCAGGTCCCAGTTCGCGCTGAAGTTCCTGGACCCCTCGTTCGTCCCCATT

ACAAATTCGCTGGGCCAAGAGCTGCAGGAGAAGCCCTCCAAGTGGGTGTTCAACCGGACGGCATTCGCAC

AGCAGAGACAAGAAATCCTTCAGCACGTCGATGTCATAAAAAATTTTTCTTTGACCAAGAATAGTGTTCG

GATTGGACAGCTGATGCATTATGATTATTCCAGCCATAAGTATGTTTTTTCCATAAGCAATAACTTCAGA

TCGCTGCTTCCAGATGTGTCACCAATCTTGAATAAACATTACAACGTTTGTGCCGTGGTTGGAAACAGTG

GAATCCTGACTGGGAGTCAGTGTGGGCAAGAAATAGATAAGTCTGATTTCGTTTTTCGCTGCAATTTTGC

TCCAACTGAGGCATTCCAAAAAGATGTTGGGAGGAAAACCAATCTCACAACCTTCAACCCCAGCATCCTG

GAGAAGTATTACAACAATCTTTTGACCATTCAGGATCGCAACAACTTCTTTCTGAGCTTAAAAAAGCTTG

ATGGGGCGATTCTTTGGATCCCTGCTTTCTTCTTCCACACGTCAGCAACGGTCACAAGAACACTGGTTGA

CTTCTTTGTTGAGCATCGAGGCCAACTAAAGGTCCAGTTGGCTTGGCCAGGAAATATAATGCAGCATGTT

AACAGATACTGGAAGAACAAACACTTGTCACCCAAGCGGCTGAGCACAGGTATTCTCATGTATACCCTTG

CTTCTGCCATATGTGAAGAGATTCACTTGTATGGATTCTGGCCCTTCGGGTTTGATCCCAACACGAGGGA

GGACCTCCCATACCACTACTATGACAAGAAAGGAACCAAGTTCACCACCAAGTGGCAAGAGTCCCACCAG

CTGCCTGCAGAGTTCCAGCTGCTCTACAGGATGCACGGTGAAGGACTGGCCAAACTGACCTTGTCGCGTT

GTGCCTAA

>Chicken TCF4

ATGCATCACCAACAGCGAATGGCTGCCTTAGGGACGGACAAAGAACTGAGTGATTTACTGGATTTCAGTG

CGATGTTTTCACCTCCCGTGAGCAGTGGCAAAAATGGACCAACTTCCTTGGCGAGTGGACATTTTACTGG

CTCAAATGTAGAAGACAGAACTAGCTCAGGGTCCTGGGGGAATGCAGGACATCCTAGTCCATCCAGGAAC

TATGGAGATGGGACTCACTATGATCATATGGCGAGCAGAGACCTGGGGTCACATGACAATCTCTCTCCTC

CATTTGTCAATTCCAGAATACAAAGTAAAACAGAAAGGGGATCATACTCATCATACGGAAGAGACTCGAA

TTTGCAGGGTTGCCACCAGCAAAGTCTCCTGGGCGGGGAGATGGACATCGGCAACCCCGGGGCGCTGTCC

CCCAGCAAGCCGGGCTCCCAGTACTACCAGTACTCCAGCAATAACCCGCGCCGCCGGCCGCTGCACAGCA

CCTCCATGGAAGTTCAGACGAAGAAAGTTCGGAAGGTTCCTCCGGGTTTGCCGTCCTCAGTGTATGCCCC

GTCAGCCAGCACTGCCGACTACAACCGCGACTCCCCCGGTTACCCGGCCTCCAAACCAGCAGCCAGCACT

TTCCCCAGCTCCTTCTTCATGCAAGATGGGCACCACAGCGGAGACCCGTGGAGCTCCTCCAGCGGGATGA

ACCAATCGGGTTACGGGGGGATGCTGGGCAACTCCTCGCACCTCCCCCAGTCCGGCAGCTACTGCAGTCT

GCACCCCCACGACCGCCTGAGTTACCCATCCCACTCCTCAGCCGACATCAACTCCAGTCTTCCTCCGATG

TCCACCTTCCACCGCAGCGGCACCAATCACTACAGCGCGTCGTCCTGCACCCCTCCAGCCAATGGGACGG

AAGGCATCATGGCCACCAGAGGAAGCGGGGCGGCCGGCAGCTCGCAGACCGGCGACGCGTTGGGGAAAGC

ACTCGCATCTATCTATTCTCCAGATCACACCAACACCAGCTTCCCATCCAACCCTTCAACTCCTGTCGGT

TCTCCCCCTTCTCTCTCAGCAGGCACAGCTGTTTGGTCTAGAAATGGAGGTCAAGCGTCATCATCTCCCA

ATTATGAAGGTCCCTTACACTCTTTGCAGAGCCGCATCGAGGACCGCTTGGAGAGGTTGGACGACGCCAT

CCACGTGCTGAGGAACCACGCGGTCGGAGCCGGGACGGCCGTCGGGGGTGGCCACGGGGATATGCACGGC

TTGATAGGAGCTGCTCACAACGGGGCCATGGCGGGCTTGGGGTCCGGCTACGGGACCGGGTTGCTGTCGG

CCAACAGGCACTCGTTGATGGTCGGTGCTCACCGCGAGGACGGCGTCGGGCTGCGCTCTGCTCACTCTCT

GGTGCCCAATCAGGTTGCGGTGCCGCAGCTCCCCGTGCAATCGGCCACGTCCCCGGAGCTCAACCCGAGC

CAGGATCCTTATAGGGGCCTGGCGGCGGGTTTGCAAGGGCAGAGCGTGTCGTCGGGCAGCTCGGAGCTGA

AGTCGGACGACGAAGGCGACGAGAACGCGCAGGAGGCCAAATGCGGCGACGACAAGAAGCTGGACGAGGA

CAAGAAGGAGCTCAAGTCAATTACGAGCAATAACGACGACGAGGATCTGACCCCGGAGCAAAAAGCCGAG

AGGGAGAAAGAGAGGCGGATGGCCAACAACGCCCGCGAGAGGCTGAGGGTGAGGGACATCAACGAGGCCT

TCAAGGAGTTGGGGCGGATGGTGCAGCTGCACCTGAAGAGCGACAAACCGCAGACGAAGCTGCTCATCTT

GCACCAGGCCGTGGCGGTGATCCTGAGCTTGGAGCAGCAAGTGAGAGAGCGGAACCTGAACCCGAAGGCG

GCGTGCCTGAAGAGGAGGGAGGAGGAGAAGGTGGCGGCCGAACCGCCCCCATTGGCCCTGGCGGGAGCCC

ACGCGGCCATGGGGGACGGAGGCACCCATATGGGGCAGATGTGA

>Chicken TXNL1

ATGGTGGGCGTGAAGCTGATCGCTAACGACACCGAGTTCCAGCCCGAGCTGAGCGCGGCCGGCTCCCGCC

TGGCCGTGGTGAAGTTCACGATGCGGGGATGTGGCCCTTGTTTAAGGATAGCTCCAGCTTTCAATGCTTT

GAGTAACAAGTACCCTCAGGCAACGTTTTTGGAAGTGGATGTGCATCAGTGCCAGGGAACAGCTGCTACC

AATAATATATCAGCAACGCCGACATTTCTGTTCTTTCGGAACAAAGTGCGGATTGACCAGTATCAAGGAG

CGGATGCTGTGGGGTTAGAAGAAAAAATTAAGCAGCACCTGGAGAATGATCCTGGAAATAATGAAGATAC

AGATATTCCCAAAGGATATATGGACTTAATGCCATTTATCAACAAAGCTGGCTGTGAATGTCTTAATGAA

AGTGATGAGCATGGATTTGATAATTGTTTACGTAAAGATTCTACGTACTTGGAATCGGATTGTGATGAGC

AGCTGCTTATTACTGTTGCTTTTAGTCAACCAGTCAAGCTTTATTCTATGAAACTTCAGGGGCCAGATAA

TGGACAAGGTCCAAAGTACATAAAAATCTTTATCAACCTTCCTCGATCTATGGATTTTGAAGAAGCAGAA

AGAAGTGAACCAACTCAAGCCCTGGAACTAACACCAGATGATATTAAAGAAGATGGCATTATCCAACTTC

GCTACGTTAAATTTCAGAATGTTAACAGTGTAACTTTGTTTGTCCAGTCTAATCACGGGGATGAAGAGAC

AACAAGAATTACATACTTCACGTTTATTGGAACTCCAGTCCAAGCAACAAATATGAATGACTTCAAGCGA

GTAGTTGGCAAAAAAGGAGAGAGCCACTAA

>Chicken WDR7

ATGGCTGGGAACAGCCTTGTGTTGCCCATTGTCCTGTGGGGCCAGAGGGCTCCCACCCACTGCATATCAA

CGCTGCTGCTCATGGATGACACGTCGATGATCATCACTGGCTGCCACGATGGCCAGATATGTCTCTGGGA

CATTGCTCCAGATTTAGAGATTAACCCCAGAGCTCTGCTGTTTGGTCATACAGCCTCAATCACTTGTTTA

TCAAAGGCCTCTGCTTCCAGTGAGAAGCAGTACATAGTGAGTGCGTCAGAGAGTGGGGAGATGTGTCTGT

GGGATGTGAATGATGGCAGATGCATAGAGTTTACTAAGCTAGCCTGCACACACACTGGCATACAGTTCTA

CCAGTTTATGGTTGGGACTCAGCGTGAAGGGAGACTGTTATGCCATGGACATTATCCAGAAATTCTTGTT

GTGGATGCTACCAGCCTTGAAGTTCTTTACTCTTTATTATCAAAGATATCTCCTGATTGGATCAGCTCCA

TGACTATCATTAAATCCAACAGAACACAAGAGGATACTGTTGTTGCAGTTTCAGTGACTGGCATCCTGAA

AGTATGGATAATAACATCTGAAGTTAATCGCATGCAGGATACAACACCAGTATTTGAAGAGGAGTCAAAA

CCTATTTATTGTCAAAACTGTCAGAGTATTTCTTTCTGTGCATTTACCCAGCGGTCGTTGTTGGTAGTGT

GCTCTAAATACTGGAGGGTTTTTGATGCTGGAGATTATTCCCTTTTATGCTCGGTCCCTAGTGAGAATGA

ACAGACCTGGACTGGTGGTGACTTTGTGTCAGCTGATAAGGTGATCGTGTGGACAGAAGATGGTCAGAGT

TTCATATATAAATTACCAGCCAGCTGTCTTCCAGCCAGTGACTTGTTTCGTAGAGATGTGGGGAAAGCAG

TAGAAAACTTAATTCCTCCTTTATTGTACAGTGTACTGGACAGAGCAGATAAACAGTTACTGATATGTCC

CCCAGTCACCCGGTTCTTCTATGGACAAAGGGAGTTTTTCTCTAATCTGCTGATCCAAGGAGACTCTTCA

GGGAGGCTGTGCATTTGGAGTATACCTGATACACTGGAACAACAAGACTGTGCAAAAGGACTGCAAACAA

CAACTTCAATATCCCTCCAGGAAGCTTTTGATAAACTTACTCCTCGCCCAGCTGGAATTATAGACCAACT

AAGCTTAATACCAAACATCAAGGAGCCACTTAAGGTTACAGCCAGCGTGTACATTCCAGCCCATGGGCGG

TTGGTTTGTGGTCGGGAAGATGGAAGCATTGTTATTGTGCCAGCAACCCAAACTGCTATAGTTCAGCTTT

TGCAAGGAGAGCATATGCTCAGGAGAGGTTGGCCACCTCACCGGACTCTCAGAGGTCATCGAAACAAAAT

TACTTGCTTACTGTATCCTCATCAGGTTTCTTCTCGTTATGATCAAAGGTATTTGATCTCAGGTGGTGTG

GATTTCTCAGTCATCATATGGGATATATTTTCTGGAGAGATGAAACATATCTTCTGTGTACATGGTGGAG

AAATTACACAGCTTCTAGTTCCACCAGAAAATTGTAGTGCAAGAGTCCAGCACTGTATTTGCTCTGTGGC

CAGTGATCACTCTGTAGGTCTTCTTAGTCTGCGTGAGAAAAAATGCATCATGCTGGCATCCCGCCACCTC

TTCCCTATTCAAGTGATCAAGTGGAGGCCTTCTGATGACTACCTGGTAGTGGGATGCTCTGACGGGTCCG

TGTATGTCTGGCAAATGGATACTGGTGCTCTGGACAGATGTGTGATGGGAATAACAGCAGTTGAAATCCT

GAATGCCTGTGATGAGGCACTGCCCGCAGCTGTGGACTCCCTGAGCCACCCTGCTGTCAACCTGAAGCAG

GCCATGACACGACGCAGCCTGGCTGCCCTGAAGAATGTGGCACACCAGAAGCTGCAGACCTTGGCCACCA

ACCTGCTGGCTTCTGAGGCATCTGACAAAGGGAATTTACCTAAATATTCACATAACTCTCTGATGGTTCA

AGCTATAAAGACTAACTTGACAGATCCAGATATACACGTGCTCTTCTTTGACGTGGAAGCACTGATAATT

CAGCTGCTGACCGAAGAAGCCTCAAGACCCAACAGTGCCCTCATCTCTCCAGAGAATTTACAGAAGGCCT

CTGGTGGGTCTGACAAGGGAGGCTCCTTCCTGACTGGTAAGCGAGCAGCTGTCCTCTTCCAGCAGGTCAA

GGAAACCATCAAAGAAAACATAAAGGAACATCTTCTTGATGATGAAGACGAGGATGAAGACTCAGTGAGA

CAGAGAAGAGAAGACACTGACCCAGAATATCGTTCTGGCAAATCCAAACCATTAACTCTACTAGAGTACA

ACCTAACCATGGATACAGCAAAGCTTTTTATGTCCTGTCTTCATGCATGGGGCTTGAATTCTGTTCTAGA

TGAGCTTTGCCTTGATCGTCTCGGGATGCTTAAGCCACACTGCTCAGTGTCCTTTGGCCTTCTGTCTAGA

GGAGGCCACATGTCTCTGATGCTTCCTGGCTACAATCAGCCTGTAGGCAAACCATCTTATGATGGCATGG

AGTTGGGCAGGAAGATGTCCATTACAGAAGGATTGGGAAAGGGGACATATGGCGTGTCACGTGCTGTCAC

CACCCAGCATCTCCTCTCTGTCATATCGCTGGCAAACACGCTGATGAGCATGACCAATGCAACCTTTATT

GGAGACCACATGAAGAAGGGCCCAACCAGGCCACCCAGGCCAGGCACACCCGAAATGTCAAAAATGAAGG

CACCTCCATCAATGGCAAGTCATGCAGCCCAAGGACAAATTAAACAAGTTGCTCCTGCTGTTTCTTCTAG

CACTGAAGCTGGTCACTCTGGCTCTGACACTGCTCCTCCTTTACATACCTGTTTCTTAGTAAATGAAGGG

TGGAGCCAGCTTGCTGCCATGCACTGCGTGATGCTGCCAGACCTGCTGGGTCTGGACAAGTTCAGGCCTC

CTCTTCTGGAGATGTTGGCTCGCAGATGGCAGGACCGCTGCCTGGAGGTAAGAGAAGCTGCCCAGGCCCT

GCTGCTCGCAGAACTGAGGAGGATTGAACAGGCAGGCCGGAAGGAGACCATTGATGCTTGGGCTCCATAT

TTGCCACAGTACATTGACAGTGTCATATCGCCTGGAGTAACCACTGAAGCCATTCAAACTGCTACTGCAA

GCCCAGATTCCTTGGGAACAGAGGCAAAAGTTCAGGAGGAAGAACATGATCTAGTGGATGACGACATCTC

AGCAGGCTGCCTGTCCGGTCTCCCACAGCTAAAGAAGATCTCTACATCCTATGAGGAGCGCAGGAAGCAG

GCTACAGCCATCGTTCTGCTGGGTGTGATCGGGGCAGAATTTGGGGCTGAGATTGAGCCCCCTAAGCTGC

TCACACGGCCACGCAGCTCCAGCCAGATCCCCGAGGGCTTTGGGCTGACAAGCGGTGGGTCCAATTATTC

CCTGGCAAGGCATACCTGCAAGGCATTGACATTTCTGCTGCTACAGCCACCCAGCCCCAAGCTGCCTGCG

CACAGTACCATCCGCAGAACTGCCATTGACCTCATCGGCCGTGGCTTCACTGTCTGGGAGCCCTACATGG

ATGTCTCTGCGGTGCTGATGGGCCTGCTGGAGCTCTGCGCTGATGCTGAGAAGCAGCTTGCCAACATCAC

AATGGGGCTGCCCCTGAGCCCGGCGGCTGACTCTGCACGCTCGGCACGCCACGCGCTCTCGCTCATCGCC

ACAGCCCGGCCGCCCGCCTTCATTACCACCATCGCCAAGGAGGTGCACAGACACACCGCCCTTGCAGCAA

ACACACAGTCTCAGCAGAATATTCACACCACTACTCTTGCTCGGGCTAAAGGAGAGATCTTGAGAGTCAT

TGAAATCCTTATTGAGAAGATGCCTACCGACGTCGTGGACCTTCTTGTTGAGGTTATGGACATCATCATG

TATTGCCTCGAAGGATCTTTAGTTAAGAAAAAAGGTCTTCAAGAATGCTTTCCAGCCATCTGCAGGTTCT

ACATGGTCAGCTATTATGAGCGAAGTCACAGAATAGCAGTTGGAGCTCGCCATGGTTCAGTGGCCCTCTA

CGACATCCGGACTGGGAAATGTCAGACAATCCATGGGCACAAGGGGCCGATCACGGCAGTGGCCTTTGCT

CCTGACGGCCGGTACCTCGCCACCTACTCCAACTCAGACAGCCACCTCTCCTTCTGGCAGATGAACACGT

CACTGCTGGGCAGCATCGGCATGCTCAACTCAGCACCCCAGCTGCGCTGCATCAAGACCTACCAGGTGCC

ACCTGTCCAGCCCGCTTCCCCTGGCTCGCACAACGCCCTGCGCCTGGCCCGCCTCATCTGGACCTCCAAC

CGCAACGTCATCCTCATGGCCCATGATGGCAAGGAGCACCGCTTCGTGGTCTAG

>Chimney swift ACAA2

ATGGCGCTGCTGCGGGGTGTCTTCATTGTTGCAGCCAAGAGAACTCCCTTTGGGAGCTATGGGGGTTTGC

TGAAGGACTTCTCAGCCACTGATCTGACAGAACATGCTGTCCGGGCTACCTTGGCTGCTGGCAAGATCTC

TCCTGAGATTATTGACAGTGTCATTGTTGGCAATGTCATTCAGAGTGCCTCAGATGCCATTTATATTGCA

AGACACGTGGGTTTACGTGTGGGAGTTCCTGTCCCAGTGCCAGCCCTCACTGTCAACAGGCTTTGTGGCT

CTGGTTTCCAGTCCATTGCCAGTGGATGTCAGGAAATCTGTCTGAATGACTCAGAAGTTGTCCTGTGTGG

TGGAGCTGAAAATATGAGCCAGGCTTCTTATGCAGTTAGAAACATTCGATTTGGAACCAGATTAGGAGCA

GAACTCAAGTTGGAAGACACATTGTGGGAAGGACTGACAGATACACATGTTAAGATACCTATGGCAGTAA

CAGCTGAAAACCTGGCTGCAAAATACAACATCACACGTGAGGACTGTGACAAATATGCCCTCAAAACACA

ACAGAGATGCAAAGCTGCTCAGGATGCTGGTTACTTCAACACTGAGATGGCACCAATTGAAGTGAAAACG

AAGAAGGGGAAAGAAAGTATGCAAAAGGATGAGCACCCCAAGCCCCAGACCACGATGGAACAGTTGGCCA

AACTCCCCTCTATCTTTAAAAAAAATGGCACAGTGACTGCTGGGAATGCTTCAGGGGTGTGTGATGGAGC

TGGTGCAGTCATCATTGCCAGTGAATCAGCACTTAAAAAGCACAGTCTTATTCCTCTGGCAAGGATAGTG

GCCTATCACTCAGCTGGCTGTGACCCTTCCATAATGGGCATTGGCCCTGTACCTGCAATTACTGAGGTTC

TGAAGAAAGCAGGCCTGACCCTGAAGGACATGGATTTGGTAGAGGTGAACGAGGCCTTTGCCCCTCAGTA

TCTCTCTGTCGAAAAAGTCTTGGGCCTTGACCCTGAAAAAACCAATGTCAACGGAGGTGCCATTGCTATA

GGTCATCCCTTGGGTGCTTCAGGATCAAGGATCACAGCTCATCTGGTCCATGAATTAAGGAGACGTGGTG

GGAAATACGCAGTTGGGTCAGCTTGCATTGGAGGTGGACAGGGCATTGCTCTTATCATTGAGAACACAGC

CTGA

>Chimney swift C18ORF32

ATGGTGTGCATTCCCTGTATTGTCATTCCTGTTCTCCTCTGGGTCTACAAGAAGTTCCTGGAGCCTTACA

TCTACCCCATCATCGTGCCCTTCATCAAACGGGTTTGGCCCAAGAAAGCTGTGGAAGAAAGAACAACCAC

AGGAGGAGGCCAAGGAGCCACCACTGGAAATCCACAGGCACCTTCAGGCACCAAAAGAGATCAGGAGGAT

GAGTTCGGAATTTACAGATTTGAAAGTAATGGTGTGGCCAATGGAATCATTGCTGGGAGATCTACAGAGG

CTTCCAACAAGAAAACAGATTAA

>Chimney swift C18ORF54

ATGACAAGCTCAGTGAAAGGAAGCAGTGGCTGCTCTCCTGATTCAACAGTCTCTTCTCTTTTAAGAAGCT

GTGGCATTGACAGCAATCATCCACACTCAAGCAGTTGGATTCACTACAAGGGCAAGCTTTACAGCTCTGC

ATCCGAGGCTTTGGAGGCTTATATTGAAGATTATAATTTAAGTCTCACATCCCCAGGAGTAAGCACTGGA

AAAATCTGCATATGTCAAAGCACTCCCAAGCAAGTCAATTTTTCAAGACAGCATGCCAAAGAAACACACG

CAATTCATGTAAAATATCTTTGTTTTTCAGCACTGGGAGATTTTAATCAGCAAAGAGGACTGGGCCTTCT

TGCCTCACCCTGTGGAGGGCAGGCTGAGTGTGACCCAGACTTGGTTAGTCTTACAACAGATGATCTGTTA

GCATTTCCAGAGGATGGGTCACTGCCCTTGGTCGAGAGTCCTTCTCAACCAAAGCATCCCAGCAGTGGCT

GGAACAGATGGAGTAGTGGAACACTTCAACCACCTTTCTGCCCTCAGCAGACCTCATCTCTCAATCCTGA

AAGTCCCTCAAGTCCTCAGAAGAATGGGAAAGCTGCTGCTCACCAGGATCCACACAAAGACTTGAAGAAA

TGGAACCTCTCTACACCTGCTGGGTATGATCCTGTCTCTAGAGGAAGTCCAAGAGCCTTGTCTTTGGAAG

AGAACTCCAACACTGTGCATCTTAAGAATTATCCTGGCTGGCTCAGCAGTCACAAGTCTGACTTCAGTGT

ATCAGAGATGAGCAGTATCCCCAAGCTGTACTTCCCAGCCTGGCTCCAGAAAGAAGGACCCTCTGCAGAC

AAAGACAATGCTGATTTTCTTGACCCAAGTGGTTTCCTGGATCTGAGAGGTGACAACCAAGTAGAAGATG

CCAGCCCAGTTGCATGCTGCCCATTTGATACCTCGTTTTTGAGAGGCACTAAAAAGCCCTTCAGAGAAGG

GCAGCTTGGGCTGCCTCCCTTGAAGGTTGACATGGGTCTGGAGAGTTCAGCTGAAGACCTGGCAAAGAAT

CTGGAGCCTGATGGCAGCTCCTCCACCACAGAGACACTAGAAACTGAAAGGTCATGGGAAAAGGCTCCAG

CTGCTGTCAAACCACCAGTGCCTGGGTGCTGTGAGGACATGGAGAATGCTCTCTCATTCCCCAAGGCAGA

AATCATACATAAATTCCTGGAAGACTGTTTACGGGACAAAAACCAGGAACAGACTTTTTGTGGAGATCAT

CACGACAGACCCCTGGAATCTTTGAAGTTCATGCTGTTTAAACTCCAAGCACTTCAAGGAAGCTTAAGCC

AGAATGAAACTGCTGAGCCAAAGGAAGAGTTTGAAAAACTTCCTGAAAAAGCAGAGGATGAATTAAAGCT

GTGTGGCAGTGAGATAATCCCTCTTGCTAATTCTGTTCAGAAGACTTTACACCATTTCTCACATCTGAAA

AGTCTGGTTGAAGACAAGAACCAACAAGAACTGACCTGTGATCCTGAAGGAGATATAGAAGGAAAAGGAA

TTATCTGTGAAGCTGATATCTAA

>Chimney swift CCDC68

ATGACCACCCTGCTGCTCACAGAGCAGCTCACACGAGAAGACCATGGCCCAGAGGGGACCTACACCCTCT

ATGGATCCTCCTGCTCCCAAATCACTGAGGAAGCTGAATACATGAAAAAGCTTCCTGGAGGTTCAGGCCA

CGAAGCTGAGCCCAAGAGCAGCAGCTGGAGCTGCAGCCCAGTCGGGAGGAGGGTGATGGAGACAGAGGAG

CAGCTGCTGGTGGTGAGCAGGGACAACCAAGTGCTGAGGATCAAGCTGGAAGCCACGAGAGAAGCAGGGG

TTCAGGCCCTCAGATCTGCCTCCCAGAAGCTGTATGAGACTTACCAGAGTTGGGCAGAAGGCCTGAAGAA

AAGCCACGAGAAGGAGAAGCAGCAGCTCCAGGCTTACAAACTCCAACAAGAAGAGAAGCTCCAGGAAAGC

ATGGGGAGCACCAGCCACCTCAGCCAAGGGCTCCAGGAAAGATCTGCCCACATAGCAGAGCTGGAGAAGC

GAGTGCAGAGGATGGAGGAGGAAAAGAAAAGTCTGCTGGAGAAGAAGATGTCATTTGAAAAGATGCTTCA

GCAGATGATGGGGAGGAGTGAAGATGGCAAGAGGTGCCTGGATCTCCAGAGGCAGATTTCCACCCTGCAG

CAGCAGATCTGCCACCTGCAGCTCCTGATCCAGGGGCAGCACCAGGCCCTGCGTGGTGTCATCCAGGAGG

CAGAGGAACTGAAGAACCAGCTCAAAAGCCACGATGAGAAAATAGAAAACCTGACAGAGAAGCTGACCAT

GCTGGAAGCTCAGGCAGAGGAACTGAAGAACCAGCTCAAAAGCCACGATGAGAAAATAGAAAACCTGACA

GAGAAGCTGACCATGCTGGAAGCTCAGAACAAAGAACTGAAAGATCAAGTGGAGCTGTGGTCAGGCCAGC

CCAAGCCAAAGGTTTCAAGAGCTGTCTGGACAGACACCCCAGGAGGTTCTGGAGCTTTTGGAGCACCCTC

CTACCTGATGCTCAGCAGGCTAAGGAAGCAAGAGAGCTAA

>Chimney swift CFAP53

ATGGGGTGTGGGGAGACACGGGAGCCCCGGGGGGCTGGGAGGGAGGAGGAGGAGGCAGCGGGAGAGAGGT

TGAGGGCCAAGCCTCCAAAGGGTGAATCAAGTGAACACTTTGTCTTTGTCCGGAGGCAGAAGGATAAGGA

GGTCCAGGAATATTCTGATTTTGCCAAGGAGTTCCACCAGTATTGCAACATCTACAAGTGGCACAAAAGC

AACCAACACAAATGGCTCCATGGGGATGTGCAGAGAAAGGTGGATGCCAAAATGCAGGAATATCTGGCAG

GGCTTCATGAAAGAAGAGAGAGGCTTCGTGAGCTTCTGGAGGGAGAGGAAAGTCAGTACCTTGTTGAGAT

TGATTCACTTGAAGAAACAGTCCAGGGGAGACAACAGAAAATGAGGGAAAGAGCACAATTACTGAGAGAG

AAGAGAGAAAAAGAGAGACAACAACTGGCGGATGAAAAACGAGAGCAGCAATTCAGACAACAATGTGATG

AGCTTCGCTTGCTGTTGATGAAGAAGCATCAGAAGGAAGTGTGTGAAGACCGGTTGGCCCAGGTAGCTCT

GAAGGAGGAGCTGAAAAAGCAGCAGCAGAAGGAGGAGCAGATGTTTGCTGAGCTTTGGAAAGAGGACAGG

TTGGCTAAGGAAAAGAGAGAAGTGAAGGAGATGCAGAGATTGGCCAAGCAGCAGCGGGAAGTCCGTGATG

GGCTCAGTGCCCAGGTAGCAGAGCTCAACGCTCAGCAGGAGGAGGCCAGGAGGTTGAAGGAGGAAGAAGC

TCAGTTGTTGGAAGAGGAGCAGCAGCTGCTTCAGCAGGGAAAGGAGCAACTTCAGAAAGAGGAACTCCAG

AAGCAGAAGGAATGCAGGGATATGTTGCTCAGTGCAGCCCAGGAGAAGCAGAAACGTCTTGATGAGGAAA

AACAAGATGAACTTGCCCTCGAGTTGAAGATCTTGGAAAAATGTCTTCAGGACCCCCAGAAAGACACTGA

GGAGAAAACAAAAAGAAAACAAGAGCTGTTAAGGGAGCAGCAGACATACCTGGCACACCTGGCTCAACAG

CTGGAGGAGGAGAGACAGCGAGAACAAGAAGCAGAGAGAGCCCACAAGGAAGAGATTGAGAAGGTTTGGG

CTAAGAAGGCAGAGAAAATGAGAGCAGAAAAGGAGGCTAGAAAGCAGCTACTGAAGGATGTCATGGACAC

ATGGCAATGGCAGATAGAGCAGAAAGTGCAGAGAAATGTGAAGGAGCAGGAAGAACTTGCTGAGGAGAAG

AAGTTATTAGATGAAGCAATCTCGGAACTCAACCACATAGAAGAGAAGAAATATGCAAGGAAACTAAAGG

AAGCCAAAGAATACCAGGAGCACCTCAGGGCTCAGATTGCCCATCGAGAACAGGCACGTGATGCTGAGGA

AGAGGAGAAGCAACGAGAGTATGAATCAGCTCGTGAAGAAGAGAGAGCCTACCAGGAGAGGATACAGAAC

ATGTTATCAAATCCTTGGAGGAAACCAGCAGCCATCCACCCTTTCAGAAGACAAGTCCTGTCTAACTCCC

TACAACAGAATTTACACTGA

>Chimney swift CTIF

ATGGAGAACTCGTCGGTGGCATCGGCCTCCTCGGAGGCGGGGAGCAGCCTCTCCCAGGAGATCGAGGAGC

TGGAGCGTTTCATCGACAGCTACGTCCTGGAGTACCAGGTCCAGGGGCTGCTGACGGATAAAGGAGAGGG

GGATGGAGAGAGTGAGAAGACCCAGTCCCACGTCTCGCAGTGGACGGCAGATTGTACTGAGCAGCTGGAT

GGCAACTGTTCCCCATCCCGAGGGAAGGGCTCCTCATCTCACCAACACAATCAGAATGGCAACAAGGAGA

CCTCCCTGGACATGCTGGGCACAGACATCTGGGCTGCCAACACCTTTGACTCCTTCAGTGGAGCGACGTG

GGACTTGCAGCCTGAAAAATTAGATTTCAGCCAATTTCACAGGAAGCTGAGAAACACCTCCAAACACCCA

CTGCCTCACATAGACAGAGAAGGGCTTGGAAAAGGGAAATATGAAGATGGAGATGGCATCAACTTGAATG

ACATAGAGAAAGTCCTTCCAGCATGGCAGGGCTACCACCCGTTGCCTCATGAAGCTGAAATCGCACACAC

CAAAAAACTGTTCCGAAGGAGAAGAAACGACCGGAGGAGACAGCAGAGACTTCCCAGTGGGAACAAACCT

CAGCAACACACAGAACATCAGCAAGGTGGCACCAAACACAACAGGGACCACCAGAAACTCTACCAAGGAG

GTCAGGCCCCTCACTCCTCAGGCAGGACGGGCCACCACGGCTACAGCCAGAACCGGAGATGGCACCACAA

CCAGAAACACTCGCCCAACGACAAAGAAACGCACAGAAATGCCAAAGAGACTGAGAATTTGAAAACTGAG

GACACCTCTGTCTGCACGGAGCACGTTCCCGTGGAGACACAGCGAGGTCCAGAGGCTGTGGAGAAGCAGT

CTCAGCAGTACCTCCAGGAGTCAGAGGCCAAGAGGAAGGACAGTATTCACGAGCGCATTGGGGAAAGACC

CAAGATCAATTTGCTTCAGTCTTCCAAAGACAGGCTGAGGAGGAGACTAAAAGAAAAGGACGAAGTCACG

GTGGAAACCACCAATCCTGAAAAGAACAAAATGGACAAACTAATTGAAATCCTCAACAGCATGAGGAACA

ACAGCAGCGACGTCGACTCCAAGCTCACCACCTTCATGGAGGAGGCCCAGAACTCCACCAACTCTGAGGA

GATGCTGGGGGAGATAGTCAAGACCATCTACCAGAAGGCGGTGACAGACCGCAGTTTTGCTTCCACGGCA

GCCAAGCTCTGTGACAAAATGGCCCTTTTCATGGTGGAAGGAACCAAATTTCGGAGTCTGCTCCTCAACA

TGTTGCAGAAGGATTTCACCATGCGGGAGGAGCTGCAGCAGGGGGACGTGGAGCGTTGGTTGGGCTTCAT

CACCTTCCTCTGTGAAGTCTTTGGCACCATGAGGAGCAGCACTGGGGAGCCCTTCCGAGTCCTCGTCTGC

CCCATCTATACCTGCCTCAGGGAGTTGTTGCAATCTCAGGATGTGAAGGAAGATGCTGTGCTTTGCTGCT

CCATGGAGCTGCAGAGCACCGGCCGGCTGCTGGAGGAGCAGCTACCCGAGATGATGACAGAGCTGTTGGC

AATAGCACGTGACAAGATGCTGTGTCCTTCCGAGTCCATGCTGACACGGTCCCTGCTGCTGGAGGTCATC

GAGCTGCACGCCAACAACTGGAACCCCCTGACGCCCACCATCACGCAGTACTACAACAAGACCATCCAAA

AACTGACGGCTTGA

>Chimney swift DCC

ATGGAGAACACTCTTGGATGTCTCTGGGTACCCAAAGTGGCTTTTTTCTTCTTGGGGTTCACCCTGGCGA

CCCTGCAACCCGGAGTTCCAGGTGCCCAGATGAAGCCTTTTACATCCTTGAGGTTCTTGACTGAGCCCTC

AGATGCTGTCACCACGCGGGGAAGCAACGTGCTGTTGAACTGCGCGGCCGAATCGGATCGAGGAGCCCCC

GTTGTGAAGTGGAAGAAGGATGCTGTCTTCTTAAACCTGGCAGTAGATGAAAGGAGGCAGCAGCTGGCCA

ATGGATCCCTCCTGATAGAAAACATTGTCCACTCCAGGCACCACAAGCCAGATGAAGGTCTCTACCAGTG

TGAAGCATCTCTAGAAGGCGTCGGAGCCATCATCAGTCGGGCAGCCAAGGTCATGGTGGCAGGACCACTG

AGGTTTCTTTCCCAGACGGAATCAGTCACGGCTTTTGCAGGAGACACCATCCTGCTCAAGTGTGAAGTTG

TTGGGGAGCCCATGCCCGTGGTGCACTGGCAGAAGAACCAGGAGGACTTGTTGCCCAGCCCAGCTGACCC

AAGGGTGGCCATCCTGCCCTCAGGAGCTCTGCAGATCAGCAGGGTTCAGCACGGGGACAGTGGGATCTAC

CGCTGCCTGGCCAAAAACCCCGCCAGTTCCAGAACTGGGAATGATGCTGAAGTCAGAGTTTTGGCAGATC

CAGGGTTGCACAGGCAGCAGTTTTTCCTGCAGCGCCCATCAAACGTGGTGGCCATGGAGGGGAAGGATGC

TGTCCTGGAGTGCTGTGTTTCTGGGTACCCCCCTCCCACCTTCACGTGGCTACGAGGAGATGAAGTGCTC

CCCATCAGGTCCAGAAAATATTCCCTGCTGGCTGGCAGTAACCTGCTCATCTCCAACGTGACCGACGACG

ACTCGGGGACCTACACGTGCGTCGTCACCTACCGGAACGAGAACAGCAGCGGCTCCGCCGAGCTCTCCGT

GATGGTTCCACCCTGGTTTTTAATTCGTCCCTCCAACGTTTATGCCTACGAAAGTATGGATATTGAGTTT

GAATGTGCTGTGGCTGGGAAGCCTGTTCCTACAGTGGAGTGGATCAAGAATGGAGAAGTGGTCATTCCAA

GTGACTACTTTCAGATAGTGGGTGGCAGCAACTTAAGGATTCTGGGCTTGGTAAAGTCAGATGAAGGTTT

TTATCAGTGTGTAGCTGAAAATGAAGCTGGAAACTCACAGGCCAGTGCACAGCTAATCATCCCGGAGCCT

GCTGTCCCAAGCTCCAGTGTCCTCCCCTCTGCCCCCCGAGATGTGGTCCCTGTGTTGGTCTCCAGCCGCT

TTGTCCGTCTCAGCTGGCGCCCGCCCGCGGAAGCCAGAGGCAACGTCCACACCTACACGGTCTTCTTCTC

CAGGGATGGCATCAACAGGGAACGGGCAGTGAACACCTCTCAATCTGGGACACTCCAGCTGACCGTGGGC

AACCTGAAGCCAGAGGAGACCTACACCTTCAGAGTGGTGGCCTACAACGAGTGGGGACCAGGAGAGACCT

CCCAGCTCATCAAGGTGGCCACGCAGCCCGAGCTGCAAGTCCCTGGGCCGGTGGAAAACCTACGGGCTGT

GTCTACCTCCCCCACCTCCATCCTGGTCTCCTGGGATCCTCCAGCCTATGCCAATGGCCCTGTTCAAGGC

TACAGGCTCTTCTGTACAGAGACAGCAACTGGAAGAGAGCAGACCGTGGAGGTGGACGGGCTGTCCTACC

AGCTGGAGGGGCTGAAGAAGTTCACCGAGTACACCCTGCGCTTCCTCGCCTACAACCGCTACGGCCCCGG

CGGCTCCAGCCAGGACGTGGTGGTCACCACCCTTTCAGATGTGCCCAGCGCGACGCCTCAGAACGTCTCC

TTGGAAGTGGTTAACTCCAGGAGCATCAAAGTGAGCTGGTTGCCTCCACCACCAGGTACTCAAAATGGAT

TTATTACAGGCTATAAAATCCGACACAGAAAAACCACCCGCAGGGGGGAGATTGAAACACTGGAGCCCAA

CAACCTCTGGTACTTGTTCACAGGACTGGAGAAAGGAAGCCAGTACAGTTTCCAGGTGGCTGCCATGACA

GTGAATGGGACAGGACCCCCCTCGGACTGGTACACGGCAGAAACCCCCGAGAACGACCTCGATGAATCTC

AGGTTCCTGACCAGCCAAGCTCCCTCCACGTCAGGCCCTTGACAACAAGCATTGTCATGAGTTGGACTCC

CCCTCTGAACCCCAACATCGTGGTCCGTGGCTACATCATCGGCTACGGCGTGGGCAGCCCCTACGCCGAG

ACCGTGAGGGTGGACAGCAAACAACGTTACTACTCCATTGAAAACCTGGAGCCAAGTTCCCATTATGTCA

TTTCCTTGAAGGCCTTTAACAATGCAGGAGAAGGGGTCCCTCTCTATGAAAGTGCCACCACCAGGTCCAT

GACAGACCCCATCGACCCATTAGAAGTTGATTTTTATCCTTTGCTTGACGATTTCCCTACCTCAGTCCCA

GATATCTCCACCCCCATGCTCCCACCAGTAGGTGTCCAGGCTGTTGCACTTACACATGATGCAGTGAGGG

TCATCTGGGCAGACAACTCTGTCCCCAAGAACCAAAAGGCAACGGAGGTTCGTTTCTACACGGTGCGATG

GAGAACGAGCTACTCGACCAGTGCCAAGTACAAGTCAGCAGACACAACTGCTCTGAGTCACACCGTGCTG

GGGCTGAAGCCCAACACCATGTATGAGTTCTCTGTCATGGTCACCAAGGGGCGACGGTCCAGCACCTGGA

GCATGACGGCGCACGCCACCACCTACGAAGCAGCTCCAACCTCTGCTCCCAAGGACCTGACAGTTATTAC

AAGGGAAGGGAAGCCCCGGGCTGTCACTGTCAGCTGGCAGCCACCCTTGGAAGCCAATGGAAAAATTACT

GCTTACATCCTCTTCTACACCCTGGACAAGAACGCTCCCATTGACGACTGGATGATGGAGTCCATCAGCG

GCGACCGCCTGACCCACCAGGTGCTGGACCTCAACCTGGACACTGTCTACTACTTCAGAATCCAAGCTCG

GAATGCCAAGGGTGTGGGGCCTCTCTCAGATCCTATTTTCTTCCGGACACTGAAAGTGGAGCACCCTGAC

AAAATGGCCAATGACCAAGGTCGTCACGGGGATGGTTCCTTCTGGCCAGTGGACACCAACCTGATTGACA

GGAGCAGCCTAAATGAGCCCCCCATCGGGCAGATGCACCCTCCCCACGGCAGCGTCACCCCCCAGAAGAG

CAGCAACCTGCTGGTGGTCATCGTGGTCACCGTGGGGGCCCTCACGGTGGTGGTGGTGGCCGTGGTGGCC

GTGGTGTGCACCCGGCGCTCCTCGGCCCAGCAGAGGAAGAAACGTGCAACCCACAGTGCTGCCAAAAGGA

AAGGCAGCCAGAAGGACCTGAGGCCTCCAGATCTGTGGATCCACCACGAGGAGATGGAGATGAAGAACAT

TGAGAAACCAGCAGGCTCAGACCCTGCAGGAAGGGACTCCCCAATGCAGAGCTGCCAGGACATCACCCCC

GTGAGCCACAGCCAGTCAGAAACACAACTGGGCAACAAGAGCACCCCACAACCAGGCCCTGAGACAGAAG

AGGTTGGGAGCAGCATGTCCACGCTGGAGCGCTCGCTGGCTGCCCGCAGAGCCACCCGGGCCAAGCTCAT

GATCCCCATGGAGACCCAACCCAACAACCCTCCTGTGGTCAGTGCCATCCCAGTGCCAACCCTGGAGAGC

GCCCAGTACCCCGGGATCCTGCCGTCGCCCACCTGCGGGTACCCGCACCCGCAGTTCACCCTGCGCCCGG

TGCCCTTCCCGACCCTCTCTGTGGACAGGACCTTTGGAGCAGGACGAACTGCCACAGAAGGGCCAGCAGC

CCAGCAGCCCTCCCTGCTGCCCCCCAGCCAGCCCGAGCACCCCGGCAGCGAGGACGCCCCCAGCAGGACC

ATCCCCACCGCCTGTGTCCGCCCCACCCACCCTCTCCGCAGCTTCGCCAACCCCCTGCTACCTCCACCCA

TGAGTGCAATAGAACCCAAAGTCCCTTACACCCCACTTCTGGCTCAAACAGGGCCCAGCCTCCCCAAGGC

TCAGGTTAAAACAGCATCCCTTGGCTTGGCAGGCAAGGCCAGGTCCCCCCTGCTGCCCGTCTCCGTGCCC

ACTGCCCCAGAGGCTGCAGAAGAAGGCCACAAGCAGACAGAGGACTCTGCCAACGTTTATGAGCAGGATG

ACCTGAGTGAACAGATGGCCAGTTTGGAGGGACTCATGAAGCAACTCAACGCCATCACGGGCTCAGCCTT

CTAG

>Chimney swift DYM

ATGGGAGCCAACAGCAGCAGCATCAGTGAGCTTCCAGAAAATGAGTACTTGAAAAAGTTATCAGGAGCAG

AGCCCATCTCTGAGAATGACCCATTCTGGAATCAGCTGCTCTCCTTCAGCTTTACCACCCCAACCAACAG

TGCTGACTTAAAGCTCCTGGAAGAAGCCACAATCTCAGTCTGCAAGTCTTTAGTTGAGAAGAATCCTCGA

ACAGGAAACCTTGGGTCCTTGATTAAAGTCTTTCTTTCAAGAACCAAAGAGTTAAAAATCTCAGCAGAAT

GTCAGAATCACCTCTTCATCTGGCAGGCTCACAATGCACTGTTCATTATTTGCTGTTTGCTGAAGGTCTT

CATCAGTCAGATGTCAGAAGAGGAGCTGCAGCTTCACTTCACTTATGAAGAGAAAGGTCCAGGCTCATAT

GGAACAGAGTGTGAAGACCTCATAGAAGAGTTGCTGTGTTGCCTGATCCAGCTCATTGTTGAAATTCCCC

TCTTAGATATCACATACAGCATTTCCTTGGAAGCTGTGACCACTCTCATTGTCTTCCTCTCCTGCCAGTT

ATTCCACAAGGAAATTCTACGGGAGAGCATCATTCACAAGTACCTGATGCACGGTCGATGTCTCCCATAC

ACCAGCAGACTTGTGAAGACTTTACTCTATAATTTCATTAGGCAAGAAAGAAGCCCTCCTCCAGGGAGCC

ATGTCTTTCAGCAGCAAACAGATGGAGGAGGACTGCTTTATGGAATTGCATCTGGGGTGGCAACTGGCCT

GTGGACAGTCTTCACGCTCGGTGGAGTGGGCAGTAAACCAACACCTCAGCTGGAGCAGTGCTCCCCTCTT

GCCAACCAGAGTCTCCTGCTCCTGCTGGTCTTGGCTAATCTGACTGATGCTCCAGATACACCAAATCCCT

ACAGACAAGCTATTATGTCCTTCAAGAACACACAAGATAGCTCTGCTTTCTCCTCATCAAATCCCCACGC

TTTCCAGATTAATTTTAACAGTTTATACACAACTCTGTGTGAGCAGCAGAAATCTGATCAAGCAACTCTT

CTGTTGTACATGCTCCTACATCAAAATGGCAACGTCCGGACCTACGTGTTGGCACGAACCGACATAGAAA

ACCTTGTTCTGCCAATTCTGGAAATCCTGTATCACGTCGAAGAAAGGAATTCCCACCATGTTTACATGGC

TCTGATCATCCTGCTAATCCTGACTGAGGATGATGGTTTCAATCGGTCCATTCATGAAGTGGTATTGAAA

AATATCACTTGGTATGCTGAACGTGTCTTAACTGAGATCTCACTTGGGAGTCTCCTGATATTAGTGGTGA

TAAGAACCATCCAGTACAACATGACACGGACCAGGGACAAATACCTTCACACCAATTGTCTGGCAGCCTT

AGCAAACATGTCAGCACAGTTCCGCTCACTTCACCAGTATGCTGCTCAGAGGATCATCAGTTTATTTTCT

TTGCTGTCTAAAAAACACAACAAAGTTCTGGAGCAAGCCACACAGTCCTTGAGAGGTTCCTTGGGTTCAA

ATGACTCTCCCCTTCCTGATTATGCACAAGACCTGAATGTGATTGAGGAAGTCATCCGCATGATGTTGGA

GATCATCAACTCCTGCTTGGCCAACTCTCTTCACCACAACCCCAACCTGGTCTACGCGCTGCTCTACAAG

CGGGATCTGTTTGAGCAGTTTCGGACTCATCCTTCCTTCCAGGACATCATGCAAAATATAGATCTGGTGA

TCAGCTTCTTCAGCTCCCGATTGGAACAAGCTGGAGCTGAGCTGTCGGTGGAGAGAGTCCTGGAAATCAT

CAAGCAAGGAGCTGTTGCTTTGCCCAAAGACAGACTAAGAAAGTTCCCCGAGCTGAAGTTCAAGTACGTG

GAGGAGGAGCAGCCTGAGGAGTTCTTCATCCCCTACATTTGGTCCTTGGTCTACAACTCTGCCGTGGCCC

TGTACTGGAACCCTCATGACATCCAGCTCTTTACCATGGACTCTGGTTGA

>Chimney swift DYNAP

ATGGATAACCAGGCATTTGAAATGCATGGAGAGAGCATCCAGAGTTCTCCTAAAGCCAAAGAGTGGCCAA

AGAAAGAAGAGGGCAGAAAGGGCAGCTGGTCCCTGATGAAAGTCTTCCTGCTCTGCTTGCTGGCCTGTGT

CATCACCACTGCCATTGGGGTGCTGGCCCTCTCCTTGGTCTATGTCAACACCACTGCCTTCCTGAGAGAC

ACAGGGGGGAAAGAGGATGGTCCACCTTCCCCAGAGGCCGAGGAGAAAAGAGTGGATGTCAAATTCCAGT

TCCTCAATCACCTGCAGAAATCAAAGGTATTTAACTACCCAGGTGGTGAAATCCAGTGGGCAAGATTCAG

GAACGACATCAACGAATATCAAAATGATGAGGAAATGGAATTTGGGAAAAGCATCAATAACCATCGATCC

AAAATGACTTTTGGCACCTTAAGGATCAAGAGCAAAGGGCTCAGGGCTCCTCACTGGCATTTTAATGCCA

ATGAACATGGCTACCTGGTCCAGGGCAGTGCCTGGATTGGAGTCATTGGTCCAGATGACAGCGTGGTCAC

CACCTACAACGTCACAGCTGGCCAGGTGGTCTTCTTCCCTAGGAACACTGTGCACTGGATCAAGAATGTA

GGAGCAGAGGACTGTCTCTTCCTGCTGTTTTTTACAACACATGAAGAACTGCAGACCTTGGATGTAGATG

ATGCCTTTTTCTCCACCCCAGAGGATATAGCAGCTAGAGCATTAAAGCCACAAGGTGGAGTCAACTTCAT

CAGAACATTCAAGAAACAAGCAGAAGACCAAGCAGTGAACCTCCCAGCCAACTTGGATGAGCTTGTCCAC

AACGCCACCTACGTGCAGTCCCCGGACAGGCTGGTGTGGCAGTACTTCTACAACCTGAAAGGGTCAGCAG

AGTATCCCTTTCCTGGAGGAGTCTTCCAGTGGGCTCGCTACCGCAGGAACACCACTGGCCTCAACGAGAC

CGAGAAGATCTTCAGTGAGTCCCTCAACAAGCACGAGGACACCCTCACCTTGGCAACCCTCAGGATATTC

AGCAACGGGCTGGGCCAGCCCCACTTCCACTTCAATGCCAACGAGATGGGCTATGTCATCAGTGGCTGTG

CACAGGCTGGAGTTATTCTCTCTGGAGTCACTTCCAACTTCAACATTGGCATTGGGGATGTCATCTTTTT

CCCTGTTGGGACCCAGCATTACCTCAAGAGTGTGTGTGATGAGGATCTGCTCCTGATTCTGGCCTACAGC

ACAGGAAACCAGCTGGAAACCCTCCGGATGAAGGACTATTTCCATGGCACAGCAGATCATATCCTTGCTC

AGCTCTTCTTCAAGAAGCAGGAGGAGTTTCAGAAGTTCCCAAAGGCTGCCAAAAAATGA

>Chimney swift ELAC1

ATGTCCATGGATGTAACTTTCCTGGGCACAGGCTCAGCCTATCCCTCTCCAACAAGAGGAGCTTCGGCCC

TCGTGCTGCGCAGGGAAGGAGAGTGCTGGCTCTTTGACTGTGGAGAGGGAACCCAGACCCAGCTCATGAA

GAGCCATCTCAGAGCAGGCAGAATTACCAAGATTTTCATCACTCATCTGCACGGGGACCACTTTTTTGGC

CTGCCTGGCCTGCTGTGTACACTGAGCCTCCAAAGCAACCCTGACCCAAACAAACCACCAGTTGATATTT

ATGGACCTTTAGGACTGAGGGACTTCATCTGGAGGAGCATGGAGCTCTCCCACTCACAGCTTCTCTTTTT

TGTTCATGAGCTGGTACCTACACAGGACCAGTGCCCTGAGGAGGAATTTAAAGACTTTTCCTACCTGGAC

AGAGATGAGGGATTTCCCAAGGGAACACAAGGGAGAATCCTCCACCTGGATCCAGTAGAAAGCTCTTACC

TGCTGGTTGAGGATGAGCAGCTGGTTGTGAAAGCTTTTCGCCTGTTTCACCGTGTCCCTTCCTTTGGATT

TGTGGTGGAGGAGAAACCCAGGCCTGGGAAACTCAACGTAGAGAAACTGAAAGACCTTGGAGTTCAACCA

GGTCCTTTATATGGGAAACTGAAGAATGGAACTGCAGTTGTTCTAGAAAATGGAGTCACCATTTCTCCTT

CTGATGTCTTGGAAGACCCTATTCCTGGAAGGAAAATCTGCATCTTGGGGGATTGTTCAGGGGTGGTTGG

AGATGCAGCCATCAAGCTTTGCTGGGAAGCAGATGTGCTGGTACACGAAGCCACGTTGGATGATACCCAA

GAGGAAAAGGCCAGAGAACATGGTCACAGCACTCCCAAAATGGCATCAGAGTTTGCAAAGTTGTGTCAAG

CTCAGAAACTGGTTTTGAATCACTTCAGTCAGAGGTATAAACCAGCTGCTCAGGGGGGTGAGGGAGATGT

GGACATCAGCCAGCTGAAGAGACAGGCAGAGGCAGTGTTAGGAGGTCAAGAGGTGACACTAGCTGAGGAT

TTGATGACACTAGACATTCCAATGAAAAAGGAGAAGCAGCAGTGA

>Chimney swift FECH

ATGCTTTTGAACCAGAGAGCCCTGAGAGGCAGCAGCCAGCTGCGGGTCCCGGCGCGATGGAGGGGGCAGG

CGACGGCAGCTGCGGTGACAGAGAGCACAAGGCCTCGGACCCAGCCCGCGGCGCGGAAACCCAAAACAGG

AATCTTGATGTTAAACATGGGAGGCCCAGAAAGGCTGGATGATGTGCACGATTTCTTACTTCGTCTCTTC

CTGGACAGAGATCTGATGACAATGCCAGCTCAAAATAAATTAGCACCGTTCATTGCCAAGCGCCGCACAC

CGAAAATCCAGGAGCAGTACAGCAGGATTGGAGGAGGATCACCCATCAAGAAGTGGACAGCAGTGCAGGG

AGAAGGCATGGTGAAGCTGCTGGACAGCATGTCCCCTCACACAGCCCCTCACAAGTACTACATTGGGTTC

CGGTACGTCCACCCCCTGACGGAAGAGGCGATCGAGGAGATGGAGCGGGACGGCGTCGAGAGAGCCATCG

CCTTCACCCAGTACCCCCAGTACAGCTGCTCCACCACCGGAAGCAGTTTAAATGCCATTTATCGCTACTA

TAATAACAAGGGGGAGAAGCCAAAGATGAAGTGGAGCGTGATTGACCGCTGGCCCACACATCCCCTTCTG

ATTCAGTGCTTCACCGATCATATCCAGAAGGAGCTGAGCCTGTTTCCACCTGACAAAAGGAAAGATGTTG

TCATCCTCTTCTCGGCACACTCGCTGCCCATGTCTGTTGTGAACCGTGGTGATCCGTATCCTCAAGAAGT

GGGAGCTACTGTCCAGAGGGTCATGGAGAAGCTGAACTATTCCAACCCTTACAGGCTTGTGTGGCAGTCC

AAGGTTGGGCCAATGCCTTGGCTTGGTCCACAGACAGATGAGACCATTAAAGGACTGTGCCAAAGAGGAA

AGAAGAACATGTTGTTGGTCCCAATAGCATTTACAAGTGACCACATTGAGACACTTTATGAGCTGGATAT

TGAGTATGCCCAAGTTTTAGCTAACGAGTGTGGAGTTGAAAATATCAGAAGAGCCGAGTCTCTTAATGGA

AATCCACTGTTCTCCAAGGCTCTGGCAGACCTGGTCTGTTCCCATATCCAGTCGAAGGAGACCTGCTCGA

GGCAGCTGACGCTGTGCTGCCCGCTCTGCGTCAACCCCGTCTGCAGGGAGAGCAAAGCCTTCTTCACCAA

CCAGCAGCTGTGA

>Chimney swift LIPG

ATGAGTGGCATGTTCGAGACCTGGCTGGGCAGCCTGGTGTCTGCTCTCCAGGAGAGGGAGAAGGATGCCA

ACGTGGTGGTGGTGGACTGGCTGCCCCTGGCCCACCAGCTCTACACCGACGCCGTCAACAACACGCGGGT

GGTCGGGAAGAGCATCGGGAGGCTGCTGGACTGGCTGCAGGTGAGGCAGGGGAACCCAAACTTCAAGCTT

GAGAATGTCCACCTGATTGGGTACAGCCTTGGTGCCCACGTCGCTGGCTTTGCTGGTAACCACGTCCGTG

GGACAATAGGCAGAATTACAGGCTTGGATCCAGCTGGCCCCATGTTTGAAGGAGTGGACCCCAGCAGGCG

CCTGTCCCCCGATGATGCCAGCTTTGTGGATGTCCTTCACACCTACACAAGGGAAACCCTTGGTGTTAGC

ATTGGGATCCAGATGCCTGTAGGCCACCTTGACATCTACCCCAACGGGGGAGACTTCCAGCCTGGCTGTG

GCCTAAGTGATGTCTTGGGAGCAATTGCCTATGGGACCATCGGTGAAGTTGTGAAGTGTGAACACGAGCG

CTCCGTGCACCTCTTCGTGGACTCGCTGGTGAACCAAGACAAGCAGAGCTTCGCCTTCCAGTGCACGGAT

TCCAGCCGCTTCAAGAAGGGCATCTGCCTGAGCTGCCGGAAGAACCGCTGCAACGGCATCGGCTACAACG

CCAGGAAAACCAGGAACAAAAGGAACAGCAAGATGTACCTCAAGACTAGAGCTGACATGCCCTTCAAAGT

CTACCATTATCAGATGAAGATGCATGTCTTCAGCTACAGCAGTCTGGGAGAGGCTGACCCCACTTTCTCT

GTCACCCTTCATGGCACCAATGGAGACTCTGAACCTCTCTCTTTAGAAATGCTTGATCAAATCGGCCTGA

ATGCCACGAGCACCTTCCTGGTCTATACAGAAGAGGACATGGGTGAACTTTTAAAAATCAAACTCACCTG

GGAGGGAACATCTCAGTCCTGGTATGATCTGTGGAAGGAGCTGAAGAGCTACTGGTATCGACCTGCCAAG

GCTCCCCAGGAGCTCCACATCAGACGTATCCGTGTGAAATCTGGGGAGACACAGCAGAGGTTTGCCTTCT

GTGTGGAGGATTCCCAGCTAACCAGCATATCTCCTGGTAAAGAGCTCTGGTTTGTGAAGTGCCCAGAGGA

ATGGCATAAAAGACCTGTCTCAAACTCCCTCTGA

>Chimney swift MAPK4

ATGGCCGAGAAGTGCGACTGCGTCGCCAGCGCCTACGGCTACGACCTGGGCTGTCGCTTCCTCGACTTCC

ACCCTTTGGGCTTTGGAGCCAACGGGTTGGTCCTCTCAGCCCTGGACAGCAGGAGCTGCAGGAAGGTGGC

AGTGAAGAAGCTGACCATTGGTGATGCACGGAGCATGAAACACGCCTTCCGGGAGGTGAAGATCATCCGC

CGCCTGGACCACGACAACGTGGTCAAGGTGTACGAGGTGTTGGGCCCCAAGGGGACCAGCCTGAGGGGGG

ATTTCTTCAAGTTCAACGTGGTCTACATCGTCCAGGAGTACATGGAGACGGACCTGGCGCGGCTGCTGGA

GCAAGGGAAGCTGGCTGAGGAGCATGCCAAGCTCTTCATGTACCAGCTGCTGCGGGGGCTGAAGTACATC

CACTCGGCCAACGTCCTCCACCGCGACCTCAAGCCGGCCAATATCTTCATCAGCACGGAGGACCTGGTGC

TGAAGATTGGGGACTTTGGGCTGGCCAGGATCGTGGACCAGCACTACTCACACAAGGGGTACCTTTCTGA

AGGCTTGGTGACCAAGTGGTACCGCTCCCCTCGCCTCCTGCTCTCCCCAAACAACTACACCAAAGCCATC

GACATGTGGGCAGCTGGTTGCATCCTGGCTGAGATGTTGACAGGAAGGATGCTCTTTGCTGGGGGTCACG

AGCTGGAGCAGATGCAGCTTATCCTGGAAACCATCCCTGTGATCCATGAGGAAGACAAAGAGGAGCTGCT

CAAAGTGATGCCCACGTTTATCAACAGCACCTGGGAAGTGAGGAAGCCGCTGCGCAAGCTGCTCCCTGAA

GTGGACAGTGAAGCTATTGATTTTCTGGAGAAGATCCTGACATTTAACCCCATGGATCGATTAACAGCCG

AGATGGGGCTGCAACATCCCTACATGAGTCCCTATTCCTGCCCTGAGGATGAGCCAGTGTCTCAACATCC

CTTCCGGATTGAGGATGAGATTGATGATATTTTACTGATGGAAGCCAACCAGAGCCAGATATCCAACTGG

GACAGGTACCACGTCAGCCTCTCCTCAGATTTGGAATGGAGACAGGAGAAATACCATGACATGGATGAGG

TTCAGCGGGACCCCCGGGCAGGGTCTGAATCCATCGCTGAAGAAGCACAAGTTGATCCCAGGAAATATTC

CCAAAGCAGCTCCGAGAGGTTTTTGGAGCTGTCCCACTCATCCATGGACAGAGTGTTTGATGCTGATTGT

GGGAAATCATGTGATTACAAAGTGGGGTCACCTTCCTACTTGGACAAATTGCTGTGGAGGGACAATAAGC

CCCATCATTACTCAGAGCCCAAGCTGATTTTGGATTTATCCCACTGGAAAAGAGCGACCATAGCACCTGC

AGCTGAGCTGTCACTGGAAGAAGAACCATCCAACCTCTTTCTGGAGATTGCTCAGTGGGTGAAGAGCACT

CAGGTGGGTCTGGAGTGTCCCAATCCTCTTCCAGAGATTCAGGAACGGAACCTGCCACCTTCTCCTCATC

ATCTCCACCAAGAACCCACAGAGGTGAACAGTGCAACAGATCCTGAGTTTGACTTGGATGTCTTCATCTC

CAGGGCACTGAAACTTTGCACAAAACCTGAGGATCTTCCAGACAACAAGCTCAATGACATCAACGGGGCC

TGCATATCTGAGCACCCCGGGGAGATGGTACAAACAGAGGTCTTCCAGAAAGAGAGGTGGTGA

>Chimney swift MBD2

ATGNNNCCAAGTGGTAAGAAGTTCAGAAGCAAGCCCCAGTTGGCAAGATACCTGGGAAACACTGTTGATC

TCAGCAGTTTTGACTTCAGAACGGGAAAGATGATGCCCAGTAAATTGCAGAAGAACAAACAGAGACTAAG

GAATGATTCTCTCAATCAAAATAAGGGAAAACCAGACCTAAATACAACTTTACCAATCAGACAAACAGCA

TCCATTTTCAAACAACCAGTCACCAAAGTCACCAACCATCCCAGCAACAAGGTCAGGTCTGATCCACAGC

GGGTGACGGAGCAGCCACGACAGCTTTTCTGGGAGAAGAGGCTACAAGGCCTTAGTGCATCAGATGTCAG

TGAACAAATCATAAAATCCATGGAGCTTCCTAAAGGTCTTCAAGGGGTTGGCCCTGGGAACAATGATGAC

ACCCTGCTCTCAGCTGTTGCCAGTGCCCTGCACACCAGTTCTGCCCCCATCACAGGGCAGCTCTCTTCAG

CTGTGGAGAAGAACCCAGCAGTCTGGCTGAACACATCTCAGCCCCTCTGCAAAGCTTTCATAGTCACAGA

TGATGACATTAGGAAACAAGAAGAGCGGGTGCAACAAGTGCGCAAGAAGCTGGAGGAAGCTCTGATGGCA

GATATCCTGTCCCGGGCTGCTGACACAACAAAGGACCTAGATGTGGAGATGGATAATGGAGAGGAGGCAT

GA

>Chimney swift ME2

ATGTTCTCCAGGCTCCGAGGAGCTGCCACTCCCTGGGTGGTTGCTTGTCGCAGAGCACACACCAAGGAAA

AAGGGAAACCACTGATGTTAAACCCACGAACAAACAAGGGCATGGCCTTCACGTTACACGAGCGACAGAT

GCTTGGGCTGCAAGGACTTCTGCCTCCTAAAATAGAGACTCAGGACATCCAAGCCTTACGTTTCCACAAG

AACTTGGCCAAAATGACTGACCCCTTGGAAAAGTATATCTACATCATGGGAATCCAAGAGAGGAATGAAA

AATTATTCTATAGGGTATTACAAGATGATATTGAGAGGTTAATGCCAATTGTTTACACACCAACAGTAGG

CCTTGCCTGCTCCCAGTATGGACACATCTTCAGGAGACCAAAAGGCTTATTTATTTCTATCTCAGACAGA

GGCCACATAAGGTCAATTGTGAACAACTGGCCAGAGAATGATGTCAAGGCTGTAGTTGTCACTGATGGAG

AAAGAATATTGGGTCTTGGTGACCTAGGTGTGTATGGGATGGGAATTCCAGTAGGAAAACTGTGTTTATA

CACAGCCTGTGCAGGAATCCATCCAGACAAATGCTTGCCTGTGTGCATCGACGTTGGAACTGATAATCCA

ACACTCTTAAAAGATCCATTTTATATGGGCCTGTACCAAAAAAGGGATCGCTCACAGGTCTACGATGACC

TAATTGATGAATTCATGGAAGCCATCACAGACAGGTATGGCCAGAACACACTGATCCAGTTTGAAGACTT

TGGGAACCACAACGCTTTTCGCTTTTTAAGAAAATACAGGGAGAAATATTGTACCTTCAATGATGATATT

CAAGGGACAGCTTCAGTGGCCTTGGCAGGGCTGTTGGCAGCACAGAAAGCCACTGGGAAACCCCTCTCAG

AGCAGAAGGTGCTGTTCCTTGGAGCAGGAGAGGCTGCCCTGGGCATTGCAAACCTCATTGTCATGGCCAT

GATGGAAAGTGGAGTTTCTGCTGAGGAAGCCTACAGGAGGATATGGATGTTTGACAAGTATGGTTTACTG

GTTCAGGGCCGAGAACAAATGGTGGATTCCAATCAAGAACCATTTACACATCAGGCTCCAGAGCAGATAC

CAAAGACATTTGTAGAGGCAGTGAATGTACTTCGACCTTCAGCTATTATTGGAGTTGCCGGCGCTGGGCG

GCTCTTCTCTCAGGATGTGATCAAAGCCATGGCCTCTATCAATGACAGACCCATCATCTTTGCCCTGAGT

AACCCCACAGTGAAAGCTGAATGCACAGCAGAAGAAGCCTACACATTAACAGAGGGCCGTTGCCTGTTTG

CCAGTGGCAGTCCCTTTGAGCTGGTGACTCTGCAAGATGGGAGAACCTTCAAACCAGGCCAAGGAAACAA

TGCTTATATTTTTCCAGGTGTGGCTCTGGCTGTGATCCTCAGCAGTGTGAGACATATTAGTGATCAGGTT

TTCCTAGAGGCTGCTAAGGCCTTGGCAGAACAGTTGACTGATGAAGAGCTTGCCCAGGGAAGACTTTATC

CTCCACTGTCTAATATCAGGGAAGTTTCTATTTATATCGCTGTCAAGGTGATGGAATTTTTGTATGCCAA

CAACATGGCTTTCCATTACCCGGAGCCCGCCGACAAGAACCGCTACATTCGCTCCAAGGTTTGGTCCTAT

GAATACGAGTCCTTCATGCCAGATGTGTATGACTGGCCTGAATCTAAAGTTCACTGA

>Chimney swift MEX3C

ATGNNNNGTTGCAAAATCAAAGCACTAAGGGCCAAGACAAATACTTACATCAAGACCCCCGTTCGTGGAG

AAGAACCCATCTTTGTTGTCACCGGCCGGAAAGAGGACGTAGCCATGGCCAAAAGGGAAATCCTCTCAGC

TGCTGAACACTTCTCCATGATCCGGGCGTCGCGCAACAAGAACGGCCCTGCCCTGGGGGGTCTGCCCTGC

AGCCCCAACCTGCCGGGCCAGACCACAGTCCAAGTCAGGGTGCCTTACCGGGTGGTCGGGCTGGTGGTTG

GACCCAAAGGAGCTACCATCAAAAGAATCCAGCAGCAGACCCACACCTACATTGTCACTCCCAGCAGAGA

CAAGGAGCCCGTCTTCGAGGTGACCGGGATGCCCGAGAACGTGGACCGGGCGCGGGAGGAGATCGAGATG

CACATCGCCATGCGCACCGGGAACTACATCGAGCTCCACGAGGAGAACGACTTCCACTACAACGGCACGG

ATGTCAGCTTCGAAGGGGGCACCCTGGGATCTGCCTGGCTGGCTTCTCATCCCGTCCCTCCCAGCCGCAC

CAGGATGATTTCTAATTATAGAAACGACAGCTCCAGCTCCTTGGGAAGCGGCTCCACAGATTCCTATTTC

GGAAGCAATAGATTGGCTGACTTCAGCCCCACCAGTCCGTTCAGCACAGGCAACTTCTGGTTTGGAGAAA

CGCTGCCCTCGGTGGGCACCGAAGACCTCGCGGGCGACTCTCCCGCCTACGACTCCTTAGCAACACCTTC

CCAAACCATTTGGACCCCTTTTGAACCCGTCAACCCCCTCTCTGGCTTCGGGAACGACCCCGCTGGTCCC

ACCAAGCCTCAGTGTCGAGGCAGCCAACCATCTACTCCTCGCCTGTCACCCACATTTCCAGAAAGTCTGG

AGCACCCCTTGGCCAGAAGAGTGAGGAGCGACCCACCGAGCGTTGGCCACCAAGCTGGCCTCCCCATCTA

CATCCCTGCTTTCTCCAATGGTACCAACAGCTATTCCTCTTCCAACGGTGGCTCCACGTCCAGCTCTCCC

CCCGAGTCCAGAAGGAAGCACGACTGTGTCATCTGCTTCGAGAGTGAGGTCATTGCAGCCCTGGTCCCCT

GTGGCCACAACCTCTTCTGCATGGAGTGTGCCAACAAGATCTGTGAGAAGGAGACGCCGGTGTGTCCCGT

TTGCCAGGCCGCTGTTACTCAGGCAATCCAAATCCACTCTTAA

>Chimney swift MYO5B

ATGTTGGTGTGTTTGCAGCAAGAGCTGTTAAGGGAGCAGCAGACATACCTGGCACACCTGGCTCAACAGC

TGGAGGAGGAGAGACAGCGAGAACAAGAAGCAGAGAGAGCCCACAAGGAAGAGATTGAGAAGGTTTGGGC

TAAGAAGGCAGAGAAAATGAGAGCAGAAAAGGAGGCTAGAAAGCAGCTACTGAAGGATGTCATGGACACA

TGGCAATGGCAGATAGAGCAGAAAGTGCAGAGAAATGTGAAGGAGCAGGAAGAACTTGCTGAGGAGAAGA

AGTTATTAGATGAAGCAATCTCGGAACTCAACCACATAGAAGAGAAGAAATATTATACTAGGGTTTGGAT

TCCTGACCCTGATGAAGTTTGGAGATCTGCAGAAATTATCAAGGACTACAAAGAGGGAGATAAAAGCCTC

CATCTGAAGCTGGAAGATGAAACTCTCTATGAATATCCTATTGACCCCCAAGGGAATGAGCTGCCTTTCC

TGCGCAACCCAGATATCCTCGTGGGAGAGAATGACCTGACAGCCCTGAGCTACCTGCACGAGCCCGCGGT

ACTCCACAACCTCAAAGTCAGGTTCCTCGAGTCCAACCACATCTACACATACTGTGGTATTGTGCTTGTT

GCCATTAATCCATATGAGCAGCTGCCAATCTATGAGCAAGATGTCATCTATGCCTACAGTGGCCAGAACA

TGGGGGACATGGATCCTCACATCTTTGCAGTGGCAGAGGAGGCCTACAAGCAGATGGCCAGGGATGAGAA

GAACCAGTCCATCATTGTGAGCGGGGAGTCGGGCGCTGGGAAGACGGTCTCTGCCAAGTACGCCATGCGC

TTCTTCGCAACCGTCGGCGGCTCTGCCAGCGAGACCAACATCGAAGCCAAAGTCCTGGCATCCAGCCCAA

TTATGGAGGCAATTGGCAACGCGAAAACCACGAGGAACGACAACAGCAGCCGCTTCGGGAAGTACATTCA

GATTGGCTTTGACAAGAGATACCACATCATTGGTGCTAACATGAGGACTTACCTGTTGGAAAAATCACGA

GTTGTGTTCCAGGCAGAAGATGAGCGTAACTATCACATCTTCTATCAGCTTTGTGCCTCAGCAAGCCTTC

CAGAATTCAAAGACCTTGGACTAACATGTGCTGAAGACTTTTTCTACGCTTCTCAGGGAGGTGACACGTC

TATTGACGGTGTGGATGACGCTGATGACTTTGAGAAAACCAGGCATGCCTTCACCTTGCTTGGAGTGAAG

GAGTCTCATCAGGTGACCATTTTTAGGATAATTGCTGCCATTCTGCACCTGGGGAACCTGGAAATCCAAG

CGGAGCGGGACGGTGACGCCTGTAGCGTCTCGAGTGAGGACGAGCACCTGAGCAACTTCTGTGACCTGCT

GGGGGTGGAGCACAGCCAGATGCAGCACTGGCTTTGCCACCGCAAACTGGTCACCACGGCTGAGACCTAT

GTGAAGAACATGTCCTTGCAGCAAGTGGTGAATNCCTTGGCCAAGCACATCTATGCCCAGCTCTTCAACT

GGATCGTGCAGCACATCAACAAGGCCCTGCACACCACGGTCAAGCAACACTCCTTCATCGGTGTGCTCGA

TATCTACGGGTTTGAAACTTTTGAAGTGAATAGCTTTGAGCAGTTCTGTATCAACTATGCCAACGAAAAG

CTCCAGCAGCAGTTCAACTCGCACGTGTTCAAGCTGGAGCAAGAAGAGTACATGAAGGAGGGAATCCCTT

GGACTCTCATTGACTTCTACAATAACCAGCCCTGCATAGACCTTATAGAGGCAAAACTTGGTGTCTTGGA

CCTTCTGGATGAAGAGTGTAAGGTTCCCAAAGGCACCGACCAGAACTGGGCTCAGAAACTGTACGACCGG

CACGGCAGCAGCCAGCACTTCCAGAAGCCTCGCATGTCCAACACCTCCTTCATCATCCTGCACTTTGCTG

ACAAGGTGGAGTACCAGAGTGAGGGATTTCTGGAGAAGAACAGGGACACTGTGTATGAGGAGCAGATCAA

CATCCTGAAGGCCAGCAAGTATCAAATGGTAGCAGACTTGTTCCAAGATGAGAAGGATGCTCCACCCACC

ACTTCCATGGGCAAGGGAACATCCAAAATCAGTGTCCGTTCTGCCAGACCAGTAATCAAAGCTGCCAATA

AGGAGCACAGGAAAACAGTGGGACACCAGTTCCGCAATTCCCTGCATTTGCTCATGGAGACTCTGAACGC

CACCACGCCCCACTACGTGCGCTGCATCAAGCCCAACGACGAGAAGCTGCCCTTCAAGTTTGATCCCAGG

AGAGCAGTGCAGCAGCTGAGAGCTTGTGGAGTGTTGGAGACAATCCGCATCAGTGCTGCTGGCTTCCCTT

CCAGATGGTCCTACCATGACTTCTTCAACAGGTATCGTGTGCTTATGAAAAAGAGAGACCTCTCGAAGAA

CGACAAGAAGCAGATTTGTCAGACCCTCTTGGAAGACCTCATTAAGGATCCAGACAAGTTCCAGTTTGGA

CGTACCAAGATCTTTTTCCGTGCAGGCCAGGTGGCATATCTGGAGAAGCTACGAGCAGATAAGTTCAGAG

CTGCCACAATCATGATTCAGAAGACAGTGAGGGGCTGGCTGCAAAGGATCAAGTACCAAAGGCTGAGACA

AGCTGCCATCCTCATCCAGAGACACACACGTGGACACCTGGCACGGAGGCTTGCTGAGCACCTGAGGAGG

ACAAGAGCTGCCATCACCTTCCAGAAGCAGTACCGAATGCTGCGGATCCTCCGAGCTTTCCAGAGGGTCC

GCAAGGCCACCCTCACCATTCAGGCTTTTGCTCGGGGCATGTTTGTCAGGAGGGCTTATCGCAAGATTCT

GGCAGAGCACAAAGCCATCATCCTGCAGAAGTACGCCAGGGGCTGGTTGGCCTGCACACACTTCCGCAGG

CTCAAGGGGGCCACCCTTGTCCTCCAGTGCTGCTACCGCCGCATGAAGGCCAGGCAGGAGCTGAAGGCGC

TGAAGATTGAGGCCCGTTCAGCCCAGCACCTGAAGAAGCTCAACATTGGCATGGAGAACAAGGTGGTCCA

ACTTCAAAGGAAGATCAATGAGCAGAACAAGGAATATAGACTTCTGAACGAGCAGCTCTCTACACTCACA

TCTGCCCACTCCTCTGAGGTGGAAAAGTTGAAGAAGGAACTGCTGCATTATCAGCAGAGTCACCAGGGTG

ATGGCAACCAGCTGGTCAGCTTGCAAGAAGAGATGGAGCATCTTCGGCTGGAGCTTGAAAGAGCTCATGG

GGAGAGGAAGGTTGTGGAAGACAGCTACACCAAAGAGAAAGATCTGCTGAGAAGGAGGATATCCGACTTG

GAAGAAGAAAATGCTCTCCTGAAGCAGGAAAAAGAGGAGCTGAACAGCAGGATCCTGTGTCAAGCTGAAG

ACGAATTTGCACAAAACACAGCTGAGGAAAACATCCAGATGAAGAAAGAGCTGGAAGAAGAAAGGTCTCG

TTACCAGAACCTGGTAAAAGAATATTCAAGGCTGGAGCAGAGATATGACAACTTGAGGGATGAAATGACT

GTTATAAAGCAAGCACCAGGACACAGGAGGAACCCATCCAACCAGAGCAGCTTGGAGTCTGATTCCAATT

ATCCATCCATCTCAACCTCTGAACTAGGAGACACAGAGGATGTGATACAGCAAGTGGAGGACGTTGGGAT

GGAGAAAGCAGCCATGGACATGACTCTCTTCCTAAAGCTCCAGAAGAGAGTGAGGGAGCTGGAGCAGGAA

AGGAAGAAGCTGCAAACCCAGCTGGAGAAAAAAGAGCAAGAGAGCAAGAAATCCCAGGTGATGGAAACAA

AGACTGAAGGGACTTCAGAGCACGAAGATTTTGCATACAACAGCCTGAAGAGGCAAGAGCTGGAGTCAGA

GAACAAGAAGCTGAAAAATGAACTCAATGAGCTGAGGAAGGCTGTGGCAGAACAAGCAACCCAGAAAAAC

TCCTCCAATGACATTCAGGACAGCTACAACCTCCTACTGAATCAGCTGAAATCGGCCAGCGAGGAGCTGG

AAGTGAGGAAGGAAGAGGTGCTCATCCTGAGGACACAGATTATGAAGGCAGCCCAGCAAAAAGAGATGGG

GAAAAACATGGAGAGCATCACCATCAGTGCCAGCTGGCCCAACAGTGACAAGCACATTGACCAGGAGGAC

GCGATCGAAGCCTACCAGGGGATGTGCGAGACCAACCGCAAGACTGAGGACTGGGGGTATCTCAATGAAG

ATGGAGAGCTCGGCTTGGCTTACCAAGGTTTAAAGCAAGTTGCCAGGTTGCTGGAAGCCCAGCTGCAGGA

TCAGAGGAGAGAGCATGAGGAGGAGATAGAAGCTCTGAAAACCCAAGTGGATGCCCTGCAAGAAGAGATG

GAGAAGCAGCACCAGGCTTTCCTGCAGACCCTGCAGCTGTCTCCTGAGGCCCAGGTGGAGTTTGGACTTC

AGCAAGAAATCACACGGCTCACCAATGAAAATCTGGATCTTAAAGAATTGCTGGAAAGGTTGGAAAAGAA

TGAAAGGAAGCTGAAGAAGCAGCTGAAGATTTACATGAAGAGGGTCCAAGATTTTGAAGCATCCCAAGCC

ACGGTGCCGGCGGAGAGAAGGCGGCACGAGCAGAACATGCAAGTTTCAGTCCAGAGGAAAGAGAAGGATT

TTCAGGGCATGTTGGAATATTACAAAGAAGATGAGCCACTCCTCATCCGAAACCTCATTACAGATCTCAA

GCCCCAGACAGTGTCGGCTACTGTTCCCTGCCTCCCTGCCTACATCCTCTACATGTGCATCAGACACGCG

GATTACATCAACGATGACCAGAAAGTGCACTCTTTGCTCACCTCCACCATCAATGGCATTAAGAAAGTGC

TGAAGAAACACAACGATGACTTCCAGGTGACGTCGTTCTGGCTGGCGAACACGTGTCGCCTCCTGCACTG

CCTGAAGCAGTACAGCGGGGACTCGGGTTTCATGACCCAAAACACGCCGAAGCAGAACGAGCACTGCCTG

AAGAACTTCGACCTGACCGAGTACCGCCAGGTGCTGGGCCACCTCTCCATCCAGATCTACCAGCAGCTCA

TCAAGATAGCAGAGGCCATTCTGCAACCCATGGTCGTGTCTGCGGTGCTGGAAAATGAGAGTATCCAAGG

ACTTTCTGGTGTCAAACCAATGGGCTACAGGAATCGCTCCTCCAGCATGGGAGATGGTGACAATTCTTAC

AGCTTAGATGCCATCATTCGTCAGCTCAACACCTTCCACAGCATCATGAGTGACCAGGGGCTGGACCCGG

AGATTGTGCAGCAGGTCTTCAAGCAGCTCTTCTACATGATCAACGCCGTTGCCCTCAACAACCTCCTGCT

GAGGAAGGACGTCTGCTCGTGGAGCACGGGCATGCAGCTGAGGTTTAACATAAGCCAGCTGGAGGAATGG

CTGTGTGGGAAGAATCTGCAGCAGAGTGGAGCAGCACAGATGTTGGAGCCCTTGATTCAGGCAGCCCAGC

TCCTGCAGCTGAAGAAGAAAACCTCAGAAGATGCTGAGGCCATCTGCTCCTTGTGCACGTCGCTCACCAC

ACAGCAGATTGTGAAGATACTTAACCTCTACACTCCTGTGAATGAGTTTGAAGAACGTGTGACGGTCGCT

TTCATACGAGACATACAGATGCACTTGCAAGAGCGAAACGACCCTCCACAGCTGCTCTTAGACTTCAAGC

ACACGTTCCCTGTCATGTTTCCCTTCAACCCTTCTTCCATAACCATGGACTCCATTCACCTCCCTGCTTC

TCTCAACTTGGAATTTCTCAATAAAGTCTGA

>Chimney swift NARS

ATGCAAATAAAGCTCCTCAAGCGGCGGCTGCTCCTGGCCTGGGTGTGTGGGACGGAAGAGCTGTACGTGT

CTGAGCGAGAGGGCAGTGATGCCTCGGGTGATGGGACACAGAAGAAGCCATTCAAGACTGTTTTGAAGGC

TCTGATGACAGCAGGGAAGGAACCCTTTCCTACTATTTATGTGGATTCACAAAAGGAAAACGAGAGATGG

GCCATTATTTCAAAGTCGCAGATGAAAAATGTCAAAAAACTGTGGCACAGGGAACAAATGAAGAATGATG

CTAAGGAGAAGAAGGAGGCAGAAGATCTCTTGAGAAGAGAGAAGAATCTGGAGGAGGCTAAGAAGGTTAT

TATCAAGAATGATCCTAGTCTTCCAGAGCCAAAATGTGTCAAGATTGATGCTCTGGAGGCCTACAGAGGC

CAGAGAGTGAAGATTTTTGGCTGGATTCACAGGTTACGGAGACAAGGAAAAAACTTGATGTTCATTGTTT

TGAGAGATGGCACAGGTTTTCTTCAGTGTGTCCTATCAGATGAATTGTGTCAGTGCTACAACGGGCTGGT

CCTCTCGACGGAGAGCAGCGTGGCGGTGTACGGGACGCTTAACCTGGTCCCTGCAGGAAAGCAGGCTCCA

GGAGGCCATGAGCTGAACTGTGACTACTGGGAGCTCATTGGTCTGGCCCCAGCAGGAGGGGCTGACAATC

TCCTCAATGAGGATTCGGAGGTTGATGTGCAACTGAACAACAGGCACATGATGATCCGAGGCGAGAACAT

GTCCAAAATCTTCAAGGTGCGCTCCATGGTGGTGCAGGCCTTCAGGGATCATTTCTTTGCCAATGGATAT

TATGAGGTCACACCACCAACTTTAGTCCAGACACAGGTGGAAGGAGGCTCAACCCTATTCAAGCTGGATT

ACTTTGGTGAAGAGGCATACTTAACACAGTCCTCCCAGCTCTATCTGGAGACCTGCATTCCAGCACTAGG

AGATGTTTTCTGTATTGCTCAGTCATACAGAGCTGAGCAGTCCAGGACCCGCAGACACTTGGCAGAATAC

ACTCACATTGAGGCTGAGTGTCCTTTTATAAGTTTTGAGGATTTGCTGGACCGTCTGGAGAACTTGGTTT

GTGACGTGGTGGACAGAGTCTTGAAATCACCTGCATCGAGCTTACTGCACGACCTCAACCCGGGCTTCAC

GCCCCCTAAGCGTCCTTTCCGACGAATGAACTACACTGAAGCCATCGAGTGGTTAAAGGAACATGATGTG

AAGAAGGAAGATGGCACTTACTATGAATTTGGAGAAGATATTCCTGAAGCTCCTGAGAGGCTGATGACAG

ACACCATCAATGAGCCAATCTTGTTGTGCCGATTCCCTGCAGAGATCAAGTCCTTCTACATGCAGCGCTG

TCAGGACGATGCCCGCCTTACAGAGTCTGTTGATGTGTTGATGCCCAATGTTGGTGAGATCGTGGGCGGG

TCCATGCGTATCTGGGACAGCGAGGAGCTACTCGAGGGCTACAAGAGAGAGGGCATTGATCCCACACCAT

ACTACTGGTACACTGACCAGAGGAAATACGGTACGTGCCCTCATGGTGGATATGGTTTGGGATTGGAGCG

GTTCCTGACCTGGATTCTGAACCGCCACCACATCCGAGACGTGTGTCTGTACCCGCGCTTCGTGCAGCGC

TGCCGGCCTTAG

>Chimney swift ONECUT2

ATGNNNNCAGCCTGCAAACGCAAAGAGCAAGAGCCGAGCAAGGAGCGGAACAGCTCCCAGAAGAAATCCC

GCCTGGTTTTCACGGACCTCCAGCGCAGAACGCTTTTTGCCATCTTCAAGGAGAACAAGCGCCCTTCCAA

GGAGATGCAGATCACCATCTCCCAGCAGCTGGGCCTGGAGCTCACCACCGTCAGCAACTTCTTCATGAAC

GCCCGGCGGCGCAGCCTGGAGAAGTGGCAGGACGACCTGAGCTCCGGGGGCTCCTCCTCGGCCCCCAGCA

CCTGCACCAAGGCGTGA

>Chimney swift POLI

ATGGAGCCCTGCCCTTCTTCCCCGGAGAAGAACCGGCCGTGCCCGCAGCCCGGTGCCTGCAGGTCCCCAG

GGGGCAGGGTGGTCCTGCACCTGGACCTGGACTGCTTCTATGCCCAAGTGGAAATGATCCGCAACCCTGC

CCTGAGGAACCAGCCTCTAGGTGTGCAACAGAAATCCCTCGTTGTCACCTGTAACTATCAAGCCAGAAAT

CTTGGAGTTAAGAAACTGATGTCTGTCAAGGATGCTAAAGAGAAATGTCCTCAGCTGGTGCTGGTTAATG

GAGAAGACCTGACCCCATACAGGGAAATGTCCTACAAGGTTACAGAGCTGTTGGGGGAATTTTGTCCACT

GGTGGAAAGGCTTGGGTTTGATGAAAATTTTGTGGATGTCACCGAGATGGTTGAGAAGAGACTAAATCAG

CTGCAGCAAAGTGGATGTTCCCAAGTCTGTGTGTCTGGCCACGTGTACAACAACCAAGCTATCAATCTGC

ATGACACAAGGCACGTAAAACTAGTTATTGGATCTCAGGTTGCAGAAGAGCTCAGGGGAGCCATCTGTGC

CAGACTGGGCCTCACAGGCTGTGCAGGGGTGGCCTGGAACAAAGTGCTGTCTAAACTAGTGTCTGGCACC

TTTAAACCAAACCAGCAAACTGTTCTTCTGCCTGAAAGCTGCCAAGATCTGATGTGCAGCCTTGATCACA

TCCAGAAAGTGCCTGGCATTGGCTACAAAACCAGCAAACGTCTGGAGACGTTGGGTGTTAGGAACCTGTG

TGACCTCCAAACATTTCCATCTGCTGTGTTAGAGAAGGAACTGGGTGTTTCTGTTGCTCAGCGTATCCAA

AAACTCAGCTATGGAGAAGATGACTCCCCTGTGACTCCCTCAGGCCCTCCTCAGTCCTTTAGTGATGAAG

ATTCCTTTAAAAAATGTTCATCAGAAGAGGAAGTTAAAAAGAAACTTGAAGAGCTGCTTCCTAACCTATT

AGACAGAATCCAGAAAGATGGAAGACAGCCCCACACAGTCAGGTTGACCATTCGCCAGTTCTCCTCCACC

AACCGCTGGTTCAGCCGGGAAAGCCGCCAGTGTCCTATTCCCCCTCAGCTCCTTCAGAAGATTGGGAAAG

AAGGCAGCAACATTGTATCCCCATTGGTTAATGTCCTGATGAAGCTCTTTCGAAAGATGATAGATGTGGA

TCTTCCCTTTCATCTCACCCTTCTGAGTGTCTGCTTCTCCAACCTCAAAGACCATCCTAGCAGCAAGAAA

GGATCCATTGGCTTCTATCTCAAGCAGATGTCACCACCATCAGGCTCTGGTAAACGTGGCCAGGAAGTGG

AAGATGTCTCACAAGGTGAGGGAAGTGCTTCCTGGAACCAGAACTGCAACAGCACTGGAGCCACAAAAAC

TAGGAAGCTTTCCGAGGAAAAGAAAAGCAATATAAAAAAAGGAGGAATTCCTGACTTGCCATTTGGTTTG

TCTCCTGGTGATATTGACCAGGAAGTCTTCAGAGAACTTCCAGAAGCTATTCAAAAAGAAATTATTTCTG

GAAAAGCAGAAGAGATCCCTAGAGAGAACGTTCTGGGTCAGCCAGCAGGAGGTTCTGCAGAGGAGGGACA

GAGCAACTCCCCAGGTTGTCAGGGACTGGAGGGTGACTCTGCAGGGTGCAGTGACCACAGCTCCAGGCCC

AGTTGTTCTTCTGAGTGTTCCAGGAGTGCACTGGCAACCAGCAGAGTGGAAGAACAACCCATGGACTCTC

CAAACTTCAGAGAGAAAGGTCTGTCACCTCTGGAAGCCCTGCATGTCCCTGGCTTGGAGCAAGATAAGCA

GGCCTTTGAAACAACTTCTGAGGATACAACTTGCAGGAGGCAAGCAGGGATTGTTCTTCCTCCTCATGTT

GACACAAAGACTTTTTATGAACTACCTGCAGATGTGCAGGAAGAACTGCTAGCTGAATGGAAGAACCAGA

GCCCTGTGTCCAAGGCTTCTCTGGAAAAGAAAGCCCCTGAAAAGCCTAGAACAAATAAAGGAAGAAGGAA

CACAGCCCCATGTTTATCACAGTCCAACAGTTTGCTAAGGTATTTCAAACCACAGTGA

>Chimney swift RAB27B

ATGACTGATGGAGACTATGATTATCTGATCAAACTCCTGGCCCTTGGAGACTCTGGGGTTGGAAAAACAA

CGTTCCTGTACAGATACACTGATAACAAATTTAATCCAAAATTCATCACGACAGTCGGGATAGACTTCCG

GGAAAAGCGAGTGGTGTACAACAGCAGAGGACCAAATGGATCTCCAGGAAAAGCCTTCAAGGTACATCTC

CAGCTCTGGGACACAGCTGGACAGGAAAGGTTTCGAAGCCTCACCACAGCATTTTTCAGGGATGCTATGG

GCTTTCTGCTGATGTTTGATCTCACCAGCCAGCAGAGCTTCTTTAATGTCAGAAATTGGATGAGTCAGCT

CCAAGCCAATGCATATTGTGAGAATCCAGATATAGTCCTAATTGGGAACAAAGCTGATTTATCAGACCAA

AGGGAGGTGAATGAAAGGCAAGCAAAAGATCTGGCAGACAAATATGGCATACCCTACTTTGAAACAAGTG

CAGCTACTGGGCAGAACGTGGAGAAGGCTGTGGACACACTTCTGGACTTGATAATGAAACGGATGGAGCA

GTGTGTGGACAAGACACAGGCCTCTGACACAGCCAACGGGGGGAGCTCGGGCAAGCTCGACTCGGCACCA

CCCGAGGAGAAAAGATGTGCCTGCTGA

>Chimney swift RPL17

ATGGTGCGTTACTCCCTGGATCCGGAGAACCCCACGAAATCGTGCAAGTCCCGAGGGTCCAACCTGCGGG

TGCATTTCAAGAACACCCGTGAGACCGCCCGGGCCATCAAGGGCATGCACATCCGCAAGGCCACCAAGTA

CCTGAAGGATGTCACCCTGAAGAAGCAGTGTGTCCCCTTCCGGCGCTACAACGGGGGCGTGGGGCGCTGC

GCCCAGGCCAAGCAGTGGGGCTGGACCCAGGGCCGGTGGCCCAAGAAAAGTGCTGAGTTCCTGCTGCACA

TGCTGAAGAACGCAGAGAGCAATGCTGAGCTCAAGGGCCTGGATGTGGACTCCCTGGTGATCGAGCACAT

CCAGGTGAACAAGGCCCCCAAGATGCGCCGACGCACCTACCGGGAGCGTGGCAGGATCAACCCCTACATG

AGCTCCCCCTGCCACATCGAGATGATCCTCACCGAGAAGGAGCAGATCGTCCCCAAGCCCGAGGAGGAGG

TTGCTCAGAAGAAAAAGATATCCCAGAAGAAGCTGAAGAAGCAGAAGCTCATGGCTCGGGAGTAA

>Chimney swift SKA1

ATGGCATCCTCAGACCTGGAAGATTTGTGCTTTCACATCAACACCAAGATTTCAACGATTAAAAAGACTC

TTCAGTTAAGGAACATAGGTCAAGAACCATCCCTCAAGTCTGTGCTGTGTAAAATAGGGCAGGAGATGGT

TGTCCTGCATGACCTCCTGAATAAAATGGAAACTGAGGTTCAACAGCAGAAAAAACTGAATGCTTTGCTA

AAAGAGCTCCAGAAATCTGCTGAGAGAGAGCAGGGTGAGGCTCAGCACCTCCGTGAGAACATCCCTCCCC

ATCTGCCCAAACCAACTCAGAGCAGCATCACTGGGCTAACTGGGAAATGTGAAGAACAAGGAAAAGCCGC

GGAACCTGAACGTGCAAAGAAACCTATAAAAGAGCAAAGACCTATTAAAGAAGCAGCCTTAATAACTGCA

GAAGAATTTGAAAGTGTTCCTGCGTACATGAAGGGTCGTTTAACCTATGATCAGATTAATGCAGTTGTGC

AAGAGATGAACAAGGCTGTGGTGGGCAAGTACAAGATCCTGCATCAACCTTTGAAGTCTATGAGTGCACC

AGTCAGAAATCTCTACCACAGGTTTATAGAAGAAGAAACCAAGGACACAAAAGGTGAGTTCTTCATCGTG

GAGGCAGATATCAAGGAGTTCACCCAGCTGAAAGCCGACAAGCGCTTCCACAGCATCCTCAACATCCTGC

GCCACTGCCAGCGGGTCAGGGAAGTCCGGGGCTCCCGCCTCGTCCGCTACGTCATCTGCTGA

>Chimney swift SMAD4

ATGGACAATATGTCTATTACTAACACACCAACAAGTAATGATGCTTGTCTGAGCATTGTTCACAGCTTGA

TGTGCCATCGTCAAGGTGGAGAGAGTGAAACTTTTGCCAAACGCGCAATTGAAAGTTTAGTTAAAAAGCT

AAAGGAGAAAAAAGATGAATTGGATTCTTTGATTACAGCTATAACCACAAATGGAGCTCATCCTAGCAAG

TGTGTTACAATACAGAGAACGCTGGATGGGAGGCTTCAGGTGGCTGGTCGCAAGGGATTCCCTCATGTGA

TTTACGCTCGGCTTTGGAGGTGGCCTGATCTTCATAAAAATGAACTCAAGCATGTTAAATATTGTCAGTA

TGCTTTTGACTTAAAATGTGACAGTGTCTGTGTAAACCCTTACCATTATGAGCGTGTAGTATCACCTGGC

ATTGATCTCTCAGGACTGACACTACAGAGTTCTGCTCCATCGAGCATGTTGGTGAAGGACGAATACGTTC

ATGACTACGAGGGGCAACCATCCTTGTCTTCTGCTGAAGGCCACTCGGTCCAAACCATCCAGCACCCACC

AAGTAACAGGGCATCTACAGAGCCTTATAGCACCCCAACCATGTTAGCTCCCACTGAGGCTAGCACTACC

AGCACCACTAATTTTCCCAACATTCCTGTGGCTTCAACAAGTCAACCTACCAGTATATTGACAGGTAGCC

ATAGTGATGGACTCTTACAGATTGCTTCAGGGCCTCAGCCAGGAGCTCAGCAGAATGGGTTTACAGCTCA

GCCAGCCACTTACCATCACAATAGCACCACAACTTGGACTGGAAGTCGGCCGGCGGCCTACACACCCACC

ATACCTCACCACCAGAATGGCCATCTTCAGCATCACCCACCCATGCACCCTGGGCATTACTGGCCAGTTC

ACAATGAGCTTGCATTCCAGCCTCCTATATCAAACCATCCTGCTCCAGAATACTGGTGTTCAATTGCATA

TTTTGAAATGGACGTGCAAGTTGGGGAGACATTCAAGGTCCCTTCAAGCTGTCCCATTGTCACCGTTGAT

GGATACGTGGATCCTTCCGGAGGAGACCGGTTTTGCCTGGGCCAGCTTTCCAATGTGCACAGGACAGAAG

CCATCGAGAGGGCAAGGTTGCACATAGGCAAAGGGGTGCAGCTGGAGTGCAAAGGAGAAGGGGATGTGTG

GGTGAGATGCCTCAGTGACCACGCAGTCTTTGTCCAGAGCTACTACCTGGATCGAGAGGCAGGACGTGCT

CCAGGGGATGCTGTTCACAAGATTTACCCAAGTGCATACATCAAGGTGTTTGATTTACGCCAGTGTCACC

GTCAGATGCAGCAACAAGCAGCCACTGCCCAAGCTGCTGCTGCTGCCCAGGCTGCTGCAGTAGCTGGAAA

CATCCCTGGACCAGGATCAGTAGGTGGAATAGCCCCAGCCATTAGTTTGTCAGCTGCTGCTGGGATTGGT

GTAGACGATCTTCGCCGCTTGTGCATCCTCAGGATGAGTTTTGTCAAAGGTTGGGGACCTGATTACCCGA

GACAGAGCATCAAAGAGACCCCCTGCTGGATTGAAATCCACCTACACCGGGCCCTCCAGCTGCTAGATGA

AGTACTTCACACCATGCCCATTGCAGACCCACAACCTTTAGACTGA

>Chimney swift SMAD7

ATGNNNCCCCCTCCACCCTACTCCAGATATCCAATGGATTTTCTCAAACCAACTGCAGATTGTCCAGACT

CTGTGCCTTCCTCCACTGAAACAGGGGGAACTAATTGTCTAGCCCCTGGGGGGCTTTCAGATTCCCAAGT

TCTTCAGGAGCCCGGGGATCGATCACACTGGTGCGTGGTGGCATACTGGGAAGAGAAGACTCGTGTGGGT

CGGCTGTACTCTGTCCAAGAGCCCTCCCTGGATATCTTCTATGATCTACCTCAGGGGAATGGTTTCTGCC

TCGGACAGCTCAACTCAGACAACAAAAGCCAGCTGGTGCAGAAGGTCCGGAGCAAGATTGGCTATGGCAT

CCAGCTCACCAAGGAAGTGGACGGCGTGTGGGTGTACAACCGCAGCAGTTACCCCATCTTCATCAAGTCG

GCCACACTGGACAACCCCGATTCCAGGACGTTGTTGGTTCACAAAGTGTTCCCGGGTTTTTCCATCAAGG

CTTTTGACTACGAGAAGGCCTACAGCTTGCAGAGACCCAACGACCACGAGTTCATGCAGCAACCATGGAC

CGGATTTACTGTCCAGATCAGCTTTGTGAAAGGCTGGGGCCAGTGCTACACCAGACAGTTCATCAGCAGT

TGCCCGTGCTGGTTGGAGGTTATTTTTAATAACCGATGA

>Chimney swift ST8SIA3

ATGGACAGGTCCCAGTTCGCGCTGAAGTTCCTGGACCCCTCGTTCGTGCCCATCACGAACTCGCTGAGCC

AGGAGCTGCAGGAGAAGCCCTCCAAGTGGGCCTTCAACCGGACTGCCTTCGCACAGCAGAGGCAAGAAAT

CCTTCAGCACGTTGATGTCATAAAAAATTTTTCTTTGACCAAGAGCAGTGTCCGGATTGGGCAGCTGATG

CACTATGATTACTCCAGCCATAAGTACGTGTTCTCTATCAGCAATAACTTCAGGTCGCTGCTTCCCGACG

TGTCTCCCATCCTGAACAAGCATTACAACATCTGTGCTGTGGTTGGGAACAGTGGGATCCTGACTGGGAG

CCAGTGTGGGCAAGAGATAGATAAATCTGATTTTGTTTTTCGTTGCAATTTTGCTCCAACTGAGGCTTTC

CAGAAAGATGTTGGCAGGAAAACCAACCTCACGACCTTCAACCCCAGCATCCTGGAGAAGTATTACAACA

ATCTTCTGACCATTCAGGATCGCAACAACTTCTTCTTAAGTTTGAAAAAGCTTGATGGGGCCATTCTTTG

GATCCCTGCCTTTTTCTTCCACACGTCAGCAACGGTGACGAGAACACTGGTTGACTTCTTTGTGGAGCAC

AGAGGGCAACTAAAGGTCCAGTTGGCTTGGCCAGGAAATATAATGCAACATGTTAACAGATACTGGAAGA

ACAAGCACTTGTCACCCAAGAGGCTGAGCACAGGTATTCTCATGTACACCCTCGCCTCTGCTGTGTGTGA

GGAGATCCACCTGTACGGGTTCTGGCCCTTCGGCTTCGACCCCAACACGCGGGAGGACCTCCCGTACCAC

TACTATGACAAGAAGGGAACCAAGTTCACCACCAAGTGGCAGGAGTCCCACCAGCTGCCTGCAGAGTTCC

AGCTGCTCTACAGGATGCACGGTGAAGGACTGGCCAAACTCACCTTGTCGCATTGTGCCTAA

>Chimney swift STARD6

ATGGACTATAAGAGGATGGCAGAGGAAGTCTCAGGAAAAATCTTCTCATACAGTCAAGATACCTCAGGAT

GGAGGGTGATCAAAGTTTCTAAAAATGTTACAGTTTCCTCAAAGCCTTCAAAAGAGTATGCAGGAAATAT

ATACCGTGGAGAAGGGATAATTCAGGAAGTCCCTAGTAAAATCATCCCTTTTATGTATCTTCCTGAACAT

CGGAGAAAATGGGACAAGGCATTGCAGTCTTACAAGCTGTTAGAAAGGATTGACCAGGACACTGGTATCT

ACCACAGTGTAACACACAGCTATGGCATGGGACTGGTTTCAGCACGAGATTTTGTTGACCTGCTGCATGT

TAAGCCCTACCCTGGGGATGTCCTCACAACTAACTCTGTCAGCGTTGAACACTCCAGCTGCCCTCCGACT

CCCTCTTGTGTCCGAGGATATAACAATCCCTGCGGATACGTCTGTTCCCCTTTGCCCGAGAATCCAGAGC

ATTCCAAGCTAGTTGTATTTATTCAGCCAGAACTAGGAGGGATGCTTCCCTGTTCTGTAGTGGAGTCAGT

GCTCCCTGCTACTCTCATCAACTTAATCACTGCAACAAAAGCTGGACTGAAAAGCCTGGGAGCCCATAAC

TAA

>Chimney swift TCF4

ATGCATCACCAACAGCGAATGGCTGCCTTAGGGACGGACAAAGAGCTGAGTGATTTACTGGATTTCAGTG

CGATGTTTTCACCTCCTGTGAGCAGTGGGAAAAATGGACCAACTTCCTTGGCAAGTGGACATTTTACTGG

CTCAAATGTAGAAGACAGAACTAGCTCAGGGTCCTGGGGGAATACAGGACATCCTAGTCCATCCAGGAAC

TATGGAGATGGGACTCACTATGATCATATGGCGAGCAGAGACCTTGGGTCGCATGACAATCTCTCTCCTC

CCTTTGTCAATTCCAGAATACAAAGTAAAACAGAAAGGGGCTCATACTCATCCTATGGAAGAGATTCGAA

TTTACAGGGTTGCCACCAGCAAAGTCTCCTTGGAGGTGACATGGATATTGGCAACCCAGGAGCGCTCTCC

CCCACTAAGCCTGGCTCCCAGTACTATCAGTATTCTAGCAATAACCCCCGGCGGAGGCCTCTCCACAGCA

CCTCTATGGAAGTACAAACAAAGAAAGTTCGAAAAGTTCCTCCAGGTTTGCCATCTTCAGTTTATGCCCC

GTCAGCAAGCACTGCCGACTACAATAGGGATTCACCAGGTTATCCATCCTCAAAACCAGCAGCCAGCACT

TTTCCCAGCTCCTTCTTCATGCAAGATGGGCATCACAGCAGCGACCCGTGGAGCTCCTCCAGTGGGATGA

ACCAGCCTGGCTACGGGGGGATGCTGGGCAACTCTTCCCACATCCCCCAGTCCAGCAGCTACTGCAGCCT

GCACCCGCACGACCGCTTGAGCTACCCATCCCACTCCTCAGCAGACATCAACTCCAGTCTTCCTCCGATG

TCCACCTTCCACCGCAGTGGCACCAATCATTACAGCGCCTCTTCTTGCACACCCCCTGCCAACGGGACAG

ACAGCATCATGGCAAACAGAGGAAGTGGGGCAGCAGGCAGCTCGCAGACTGGTGATGCGCTGGGGAAAGC

ACTCGCCTCTATCTATTCTCCAGATCACACCAACAACAGCTTTTCATCAAATCCTTCAACTCCTGTTGGT

TCTCCCCCTTCTCTCTCAGCAGGCACAGCTGTTTGGTCTAGAAATGGAGGTCAAGCGTCATCATCTCCCA

ATTATGAAGGTCCCTTACACTCTTTGGTTTTCATTTCAAGAATGCCAACTGGCCTGCAAGGGCAGAGTGT

CTCTTCGGGCAGCTCTGAGATCAAATCCGATGACGAGGGAGATGAAAACCTCCAGGACACCAAGTCTTCT

GAGGACAAGAAACTAGAGGATGACAAGAAGGATATCAAATCAATTACTAGCAATAACGATGATGAGGACC

TGACACCAGAGCAGAAGGCTGAGAGGGAGAAGGAGAGGAGAATGGCCAACAACGCGCGGGAGCGCCTGCG

CGTCCGGGACATCAACGAGGCCTTCAAGGAGTTGGGCCGGATGGTGCAGCTCCACCTGAAGAGTGACAAG

CCCCAGACCAAGCTCCTGATCTTACACCAGGCTGTGGCTGTCATCCTCAGCTTAGAGCAGCAAGTCAGAG

AAAGAAATCTGAATCCTAAAGCAGCGTGTCTGAAAAGAAGGGAAGAAGAGAAAGTATCTTCAGATCCTCC

TCCACTTTCCCTGGCAGGACCCCACCCTGGGATGGGAGATGCCTCCAATCACATGGGACAGATGTAA

>Chimney swift TSPAN3L

ATGGACTGCGGCGTGATCACCTCTAAGACTGTCCTGCTGCTGCTCAGCCTCGCCTTCTGGGCAGCAGCAG

CAGGTCTCACCTATGTTGGGGCCTATGTCATCAACACCTACAGGAGCTATGACAGCTTTCTGCAGGACAA

ATATGCTCTCTTGCCAGCTGTGATCATCCTTGCCATCGCTGTGGTCATGTTCATCATCGGGTTGATTGGC

TGGTGTTCCACCTTCCGGGAGTCTCGCCTGGGTCTGGGGCTGTTCTTGGCCATTATCCTGATTATCTTTA

TTGCAGAAGTATCTGCTTTTGTCCTGGGATTTGTGTACAGGGAAAAGGTGAAAGCTGACGTGCAAGTCAC

AATGCGCTCGGTCTTTGAGAAGTATGATGGGAAGAGTGAAGAGTCTACTATTATGGATTACTTGCAAGAG

CAGCTTCATTGTTGTGGGGTGAAGAACTACAGTGACTGGACCACCACACAGTGGTTTAATGCCACTGGTA

ACAACAGTGTCCCCCTGAGCTGCTGCATGCAGGAGATGAACTGCACAGGGCGTCTGGATCAGCCCCAGGA

GCTCTACACACGGGGCTGTGCTGATGAGCTGGAGTCTGGGCTGCAGAGTGTTATCAGCTATGCTATGCTT

GTCATCCTGGGCTTTGCCATTGTAAAGTTCTTTGGCATGCTGAGTGTGTGTGTGCTGACCTGCAGGAGAG

AAGACAGTGGGTATCAGCCTCTTTACTCAGGGGTGTTCGCTTAA

>Chimney swift TXNL1

ATGNNNNNATGTGGCCCTTGTTTAAGGATAGCTCCAGCTTTCAATGCTCTGAGTAACAAATATCCCCAGG

CAACCTTTTTGGAAGTAGATGTGCATCAGTGCCAGGGAACAGCTGCTACCAATAATATATCAGCAACACC

CACATTTCTGTTTTTCCGAAACAAAGTGCGAATCGACCAATACCAAGGAGCAGATGCTGTGGGGTTAGAA

GAAAAAATTAAACAGCACCTGGAAAATGATCCTGGAAACAGTGAAGATACAGATATTCCCAAAGGATATA

TGGATTTAATGCCATTTATCAATAAAGCTGGCTGTGAATGTCTTAATGAGAGTGATGAGCACGGATTTGA

TAATTGTTTACGAAAAGACTCAACCTACTTAGAATCAGACTGTGATGAGCAGCTGCTTATTACTGTAGCT

TTTAGTCAACCTGTCAAGCTTTATTCAATGAAACTTCAGGGGCCAGATAATGGGCAAGGTCCAAAGTACA

TAAAAATCTTCATCAATCTTCCTCGATCGATGGATTTTGAAGAAGCAGAAAGAAGTGAACCAACTCAAGC

CCTGGAGCTGACACCAGATGATATTAAAGAAGATGGTATTGTCCAGCTTCGCTATGTAAAATTTCAGAAT

GTTAACAGTGTAACTTTGTTTGTCCAGTCCAATCATGGTGATGAAGAGACAACAAGAATTACATACTTCA

CGTTTATTGGAACTCCAGTCCAAGCAACAAACATGAATGACTTCAAGCGAGTAGTTGGCAAAAAAGGAGA

GAGCCACTAG

>Chimney swift WDR7

ATGGCAGGGAACAGCCTGGTGCTGCCCATCGTGCTGTGGGGCCGCCGGGCGCCCACGCACTGCATCTCCA

GCCTGCTGCTCATGGAGGAGTCCTCTGTCATTGTCACTGGCTGCCACGATGGGCAGATCTGTCTCTGGGA

CCTGTCTGCAGATCTAGAGATTAATCCCAGAGCTCTGTTGTTTGGTCATACAGCCTCAATTACTTGTTTA

TCAAAGGCCTCTGCTTCCAGTGAAAAGCAGTACATAGTGAGTGCATCAGAGAGCGGGGAGATGTGTCTGT

GGGATGTGAATGATGGGAGATGCATAGAGTTTACTAAACTAGCCTGCACACACACTGGCATACAGTTCTA

TCAGTTTACAGTTGGGACTCAGCACGAAGGGAGACTATTATGCCATGGACATTATCCAGAAATTCTTGTT

GTGGATGCTACCAGCCTTGAGGTTCTTTATTCTTTATTGTCAAAGATATCTCCTGACTGGATCAGCTCCA

TGGCTATCATCCGATCCACCAGAACACAAGAGGATACAGTTGTAGCAGTGTCAGTGACTGGCATCCTGAA

AGTGTGGATAATAACCTCTGAAGTCAGTCGCATGCAGGATACAGCTCCAGTGTTTGAGGAGGAGTCCAAG

CCCATTTACTGTCAGAACTGCCAGAGCATCTCCTTCTGTGCCTTCACCCAGCAGTCGCTGCTGGTGGTTT

GCTCCAAGTACTGGCGGGTGTTTGATGCTGGAGATTATTCCCTGTTGTGTTCAGTCCCGAGTGAAAATGA

TCAGACCTGGACTGGTGGCAACTTTGTGTCAGCTGATAAAGTGATTGTTTGGACAGAAGATGGACAAAGT

TTCATTTACAAATTACCAGCCAGCTGCCTACCAGCTAGTGACTCCTTTCGCAGAGATGTGGGGAAGGCAG

TGGAAAATCTGATACCTCCTTTATTGTACAGTGTGTTGGACAGAGCAGATAAACAGCTGCTAATATGTCC

TCCGGTCACTCGATTCTTCTATGGGCATAGGGAATTCTCCTATAAACTGTTAATCCAGGGAGACTCTTCA

GGGAGGCTGTGTATTTGGAGTGTGCCTGATACTCTGGAACAAGACGATAGTGCAAAAGTCATGGGGCCGG

CCCCGCAGACGGCCATCGTCCAGCTGCTGCAGGGGGAGCACATGCTGAGGAGGGGTTGGCCACCTCACCG

GACACTCCGAGGCCATCGGAACAAAATCACATGTTTACTGTATCCTCATCAGGTCTCTTCTCGTTATGAT

CAGAGGTATTTGATCTCAGGTGGTGTGGATTTCTCAGTCATCATATGGGATATATTTTCTGGAGAGATGA

AACATATCTTCTGTGTTCATGGTGGAGAGATCACACAGCTTCTAGTTCCACCAGAAAACTGTAGTGCAAG

AGTCCAGCACTGTGTTTGCTCTGTGGCCAGTGACCACTCAGTAGGTCTTCTGAGTCTGAGGGAGAAAAAG

TGCATCATGTTGGCATCCCGGCACCTCTTCCCTATTCAGGTGATAAAGTGGAGACCTTCTGATGACTACC

TGGTGGTAGGATGTTCAGATGGGTCCGTGTACGTCTGGCAAATGGATACTGGTGCCCTGGACAGGTGTGT

GATGGGGATCACAGCAGTGGAGATCCTGAATGCCTGTGATGAGGCAGTGCCTGCTGCTGTGGACGCCCTG

AGCCATCCCGCCGTCAACCTGAAGCAGGCCATGACACGCCGCAGCCTGGCTGCGCTCAAGAACGTGGCCC

ATCAGAAGCTGCAGACTCTTGCCACCAACCTGCTGGCTTCTGAGGCCTCTGACAAGGGGAATTTACCAAA

ATACTCCCACAACTCCCTGATGGTCCAAGCTATAAAGACTAACTTAACAGACCCAGACATACACGTGCTC

TTCTTTGACGTGGAAGCCCTGATTATCCAGCTGCTGACTGAGGAGGCCTCCAGGCCCAACACTGCTCTCA

TTTCTCCTGAGAATTTGCAGAAAGCCTCTGGGGGGGCTGACAAGGGAGGCTCCTTCTTGGCTGGGAAACG

AGCAGCCGTGCTCTTCCAGCAGGTCAAGGAAACAATCAAAGAGAACATAAAAGAGCATCTTCTGGATGAT

GAAGATGAGGATGAGGAATCGATGAGGCAGAGAAGAGAAGATGGTGACCCAGAGTATCGCTCTAGCAAAT

CCAAACCATTAACTTTATTAGAATATAATCTAACCATGGACACAGCAAAGCTTTTCATGTCCTGTCTCCA

TGCCTGGGGCCTGAATTCTGTTCTAGATGAGCTTTGCCTGGATCGCCTGGGGATGCTCAAGCCACACTGC

TCCGTGTCCTTTGGCCTCCTGTCCAGAGGAGGCCACATGTCTCTGATGCTTCCTGGCTATAATCAGCCTG

TAGGCAAACCCTCGTGTGGCAGTGAGGAGCTGGGAAGGAAAATGTCCCTGACAGAAGGCCTGGGAAAGGG

GACATACGGTGTGTCCCGGGCTGTCACCACCCAGCACCTCCTGTCAGTCATCTCACTGGCCAACACGCTG

ATGAGCATGACCAATGCGACCTTCATTGGAGACCACATGAAGAAAGGTCCCACCAGGCCACCTAGGCCAG

GCACTCCTGAGATGTCAAAAGCGAAGGCGTCCCCTTCAGTTTCAAGTCATGCAGTCCAAGGACAGATTAA

GCAAGTTGCTCCTGCTGTTTCTTCTAGCACTGAAGCTGGTCACTCTGGCTCTGACACTGCTCCCACTTTA

CATACCTGTTTTTTAGTCAATGAAGGGTGGAGCCAGCTGGCTGCCATGCACTGTGTGATGCTCCCTGACC

TGCTGGGGCTGGACAAGTTCAGGCCTCCCCTGCTGGAGATGCTGGCGCGCAGGTGGCAGGACCGATGCTT

GGAGGTCAGAGAAGCTGCCCAGGCCTTGTTGCTGGCTGAGCTGAGAAGAATTGAGCAGGCAGGTCGGAAA

GAAACCATTGATGCCTGGGCTCCCTACCTGCCACAGTACATGGACAGCGTCATATCTCCTGGAGTGACCA

CGGAAGCCATTCAGACTGGGAGTGCAAGTCCAGATTCTCTGGGGACAGAAGCAAAAGTTCAGGAAGAGGA

ACATGACCTGGTTGATGATGACATCCCACCTGGCTGCCTGCCGGGCCTCCCTCAGCTGAAGAAGAGCTCC

ACGTCCTACGAGGAGAGGAGGAAGCAGGCCACGGCCATCGTCCTGCTGGGCGTCATTGGGGCCGAGTTCG

GCGCGGAGATTGAGCCGCCCAAGCTGCTGACCCGGCCGCGCAGCTCCAGCCAGATCCCCGAGGGCTTTGG

CTTGACGAGTGGTGGATCAAATTATTCCTTGGCAAGGCACACGTGCAAGGCGCTGACGTTCCTGCTGCTG

CAGCCGCCCAGCCCGCGCCTGCCCGCGCACAGCACCGTCCGCAGAACCGCCATCGACCTCATCGGCCGCG

GCTTCACCGTCTGGGAGCCCTACATGGACGTGTCGGCCGTGCTGATGGGCCTCCTGGAGCTCTGCGCCGA

CGCCGAGAAGCAGCTGGCCAACATCACAATGGGGCTGCCCCTCAGCCCGGCGGCCGACTCGGCGCGCTCG

GCTCGGCACGCCCTGTCCCTCATCGCCACCGCCAGACCTCCCGCCTTCATCACCACCATCGCCAAGGAGG

TGCACCGCCACACCGCCCTGGCCGCCAACACCCAGTCCCAGCAGAACATCCACACCACCACCCTGGCCCG

GGCCAAGGGGGAGATCCTGAGGGTCATTGAGATCCTGATTGAGAAGATGCCTACCGACGTCGTGGACCTT

CTGGTGGAGGTTATGGACATCATCATGTATTGCCTTGAAGGATCTTTGGTTAAGAAGAAGGGGCTTCAGG

AATGCTTTCCAGCCATCTGCAGGTTCTACATGGTGAGCTACTACGAGCGCAGCCACCGGATCGCGGTGGG

CGCTCGCCACGGCGCCGTTGCCCTCTACGACATCCGCACTGGCAAGTGTCAGACCATCCACGGCCACAAA

GGAGCCATCACCGCGGTGGCCTTCGCGCCCGACGGCCGCTACCTCGCCACCTACTCCAACTCCGACAGCC

ACCTCTGCTTCTGGCAGATGAACACCTCTCTCCTGGGGAGCATTGGCATGCTGAACTCGGCCCCCCAGCT

GCGCTGCATCAAGACGTTCCAGGTGCCCCCGGTGCAGCCCGCGTCCCCGGGCTCGCTCAGCGCCCTGCGC

CTGGCGCGCCTCATCTGGACCTCCAACCGCAACGTCATCCTCATGGCCCACGACGGCAGGGAGCACCGCT

TCATGGTCTAG

>Chinese alligator ACAA2

ATGGCGCTGCTGCGAGGTGTATTTATTGTTTCAGCCAAGCGAACACCTTTTGGGGCTTATGGAGGCTTGC

TCAAAGATTTCACAGCCACTGACCTGTCTGAGTATGCTGCTCGTGCTGCACTATCTGCTGGGAAAGTCTC

TCCTGAGATCATTGATAGTGTCATCGTTGGCAATGTCATGCAGAGCTCTGCAGATGCAGCTTATATTGCA

AGACATGTTGGTCTGCGTGTGGGAGTTCCTGTCCCAGTTCCAGCCCTCACTGTCAACAGACTCTGTGGCT

CTGGTTTCCAGTCTATCACCAGTGGATGTCAGGAGATTTGCCTTAATGAGTCAGAAGTGGTTCTTTGTGG

CGGAACAGAAAATATGAGCCAAGCTCCTTACGCTGTTCGAAACGTTCGATTTGGAACAAAATTCGGAGTG

GATCTTAAGATGGAAGATACTTTATGGGCGGGCCTAACAGACCTACACATTAAAACTCCTATGGGAGTTA

CAGCAGAAAATCTAGCTGCAAAACATAACATCACACGAGAGGACTGTGACCGATATGCTTTGAAAACGCA

ACAGAGATGGAAAGCGGCTCATGAGGCTGGTTATTTTAACGCTGAGATGGCACCTATTGAAGTGAAAACG

AAGAGAGGAAAAGAGAGCATGGCTCAGGATGAGCATCCAAGACCCCAGACTACCCTGGAACAGTTGGGAA

AGCTCCCAACCGTCTTTAAGAAAGATGGGACTGTCACTGCTGGAAATGCTTCGGGTGTGTGTGACGGGGC

TGGTGCAGTCATCATTGCCAGTGAAGGAGCACTTAAAAAGCACAGCCTTACTCCACTGGCTAGAATAGTA

GCCTACCATGTGTCTGGATGCGACCCCAACATCATGGGCATTGGCCCGGTGCCTGCCATTACTGAAGCTC

TGAAGAAAACAGGACTGTCCCTTGAAGACATGGATTTGGTGGAGGTCAATGAGGCCTTTGCTCCCCAGTA

CTTGGCAGTGGAAAAAGTTTTGGGTCTAGACCCTGAAAAAACTAATGTCAGTGGAGGTGCCATTGCTCTA

GGTCATCCCTTGGGAGCATCAGGATCAAGGATCACTGCTCATTTGGTTCATGAATTAAGGCGTCGTGGTG

GCAAATATGCAGTTGGGTCAGCTTGCATTGGAGGTGGCCAAGGTATTGCTGTCATCATTGAGAACATTGC

CTAA

>Chinese alligator C18ORF32

ATGGTGTGCATTCCTTGCATTGTCATTCCAATTCTTCTCTGGGTCTACAAGAAGTTTCTTGAGCCATATA

TCTACCCTATAATTTCACCTTTCATTAAGCGCTTATGGCCCAGGAGAGCAGCACAGGAAACAATGGCCAC

AAAACAAGGTCATGGTGACAGTACTGCAACTGCAGCTACAAAAAAAGACCAGAAGGATGATTCTGGGCCT

TATAAGTTTGAAAGCAATGGTGTTGCAAATGGAGTATCTGCAAAGGGACCAGCAGAGGTTTTTGACAAGA

AGACCGACTAA

>Chinese alligator C18ORF54

ATGNNNNATGGCGTGCTCTTTAAAAAAGGTGGCTTTTATTCTCCTGAATCTACAATAACGTCTCTTCTTG

CAAGTTGCAACCTTGACAGTAATAACTCCTGCTCAAGCAGTTCAATTCACTACAAAGAGAAGCTGTACAA

CTCTGCATCTCAGGCACTCGAGGCCTACATTGAAGATTTTGATCTCAGCCTGGTGTCTCCAGAAGTAAGC

ACTGGAAAAATATGCATAAATCAAAGTACTCCTAAATGCATCAAATCCTCAGAATATTGTTCCAAGCAAA

AATATGGTTTGGAGGACCTTAACCAACATGTAAAACTAGATTCTAAAGCATCGTCTTTTAGAAGACAACG

TTTTTGTGACCTAGACTTGGTTAGTCTCACAACAGATGATTTACTAGCATTTCCACCAGATGGATCACTG

CCTTCTGTCCACTCTACTCCCTTTGGGTTAGAGCTTCAAAGTAGTGAAAGGAAAAAGAAGTCACTGGGAA

AATCAAGATTCTGTCCTTATTATGGAATGTCTTCCTTCAAGATTGAAAGAGACTTTGAAGACTATGGCAG

TATTGGCAATCAACATCCACCCAAAGATTTGGGCAAGAAGAAACGCAATGTATATAAATCTCTCAAATAT

GATTCTATATCTGAAGGAAATGAAAAAGACTTTCCTTCTGAACAGCACTTTAACTCTGTTTCTGTTAAGA

ACTATCCAAGGTGGCTTACTAGCCAGAAGTCCGACTTGAATGTGTCAGGGATAAGTAGCGTTCCTGATTT

TAAGTATCCTGTCTGGCTCAAGAATCATAACCTTTTATCCGATTCAGATGATCAAAGTTTTAATCAAACA

CATAGCATATGGGGTGATCCTTCCATATTACAAACTCACCAAAATCTGAAAAAGAGCCACTTCATGGATA

AACCGGACAGTTCCCATAACTGCTGTGAACAAAATAGTTATTTGGATCTAGTGGTTGACAAAATAGCAGG

AAAATGTGACTGTGACAGTCCAAATACATGCTTCCAGCCTGGCAGTATTTTATCAAGTCAGTCCAAGCAG

CCGTTCAGAGATGACCAGCTTGAATTGCTTATCTTGAAGGCAAGAAGAACTCTAGAATCTTCAGCTGAGG

ATTTAGCCAGTATTATGAAAAATGATGGCAGTCCCTGTACAGTAGACATACTGGAAGCAGAAAGATCATG

GGAAAATGTGCCAATTGCTTTTAAGCCTCCAGTGCCTGTACAGTGCGAGGAAGAGGAGAACGCTTTACAA

TCCCCCAAGGCAAATATTGTTAATGAATTCCTTGAAGACTGTTTAAATAATGCCGATAAGGAAAATACCT

TTTCTGGAGGTAATCATCATGGACCAGTTGAAGCCTTGAAGCTAATGTTGTTTAACCTTCAAGCAGTTCA

TGAAAGTTTTAAGCAGACTGAAACTACTGAACGGAAGGAAGAACTTATTAAACTTTCTGAAGTAGCAGTT

TCTGAATTAAAGCTAAATGATAATGAGGTGATCCCTGTTACTAAGTCCCTGCAAAAGGCTCTGCACCATT

TGTCCAGGCTCAAAAGCCTAGTTGATGATAACAGTAGCAAAGAAGATCAAGATCATGACCATCAAGGAGA

TAAAAAGAAAGAAAACTAA

>Chinese alligator CCDC68

ATGACAACCACCCTGGTACTCACAGAACGTATCACAAGAGAAGACCGAGGCTCAGATGGAAACTATTTAC

TTTATGGTTCCTCATGTGCCCAAATCTCTGAAGAAACTGAATATGTAAAAAAGGTTAGATCCACACTAGC

AAAGATTCAAAATCAGCAGCCTTTGCAAGCCTCTGATGGAACAGAATGTGAGAAGGATGCTCTTAGCTCC

AGCTACGATCCAATCATGGCAAAGATGAAGGAGACGGACAAACAGCTTGCATGTATAAGCAGGGAGAATG

AAATTCTCAAGATCAAGCTGGAAGCTACGAGAGAGGCCGGTGCAGAATCTCTCAGACATGCCTCACGGAG

GCTCTATGAGAATTACCAGAGACGGTCCGAAGAATTGAAAAAAGGGTGTGAAGAAGATAAACGCCTAATG

CAGGCCAGCAACACTGACAGAGAACAAAAACTCAAACAAAATGCAGAGAATGTCAGTTGCCTTACTGAAA

GACTTGAAGAAAAATACAGCCGGATTGCAGAAACGGAGAGACTAGTGCAAAGGATGGAAGAGGAAAAGAA

AATTTTGCTAGAAAAGAAACGGTCATTTGAAGAGATGCTCCTGCACATGATATCAAACCATGAAGATGCT

AAACGGTGTGTGGATCTTCAAGAGGAGCTTTTCAGTCTGCAACAGCAGATCCGTCATTTGCAGAATTTGA

TCATGTTCCAGAATCAAGGCCTACGCAGTGTGATACAGGAGATTGAAGAATTGAACAGAGAACTAAAAAT

CCAAGATAAAACAATAGAGGATCTGAAAGAAAAGATAAATATGCTTGAAGCTCAGAATAAATTACTTAAA

TACAAAGTGGAAGTGTCATCTGTCCAGTCAACAATGAAGGTTTCAAAAGCTGTCTCAACAGACTCAACCA

GGATCGCTGGGTTGTCACCCTACATGATGGTAGCTAGTCTACAGAAGCAAAATGGCTAG

>Chinese alligator CFAP53

ATGGAGCAGCTGTGGAGCCGGCGGCCGCGGGAGGTGTCGGGCCCCACGCCGCACTCCGTAGCGCTGAGAG

CCAAGGGCCCTAAATCGAATAAAGCTGAAAGTTTCCTGTTGGCGTGCAGACGTAAAGAAGAGGAACGTCT

TAAATACTTGGAGTTTGCAAAGCTCCACAGCAAGGGACGTGAGATCAGTCAGTGGGAGGAACACAATGAA

TGCAAAAGACTACACAGTACTGTACGGAAGAAGGTTGCTGAGACGATGCAAGAGTACCTGGCAGGGACTG

AGGATAGGAGAGAGAGACTTCGTGAACTTCTGGAAGCAGAGGAAAATAGCTATTTTTCAGAGTTGGAGTC

ATTGGAAGAAACTGTTCTTGAAAGACAAGCAAAAATGAGAGAGAGACTAAAACTATTGAGAGAGAAGCGA

GAAGAAGAAAGGCAAAAATTGGTTGCTGAAAAACGAGAGCAGCAGTTCAGAGAACAATGTGAGGAGTTTC

GTTTACACTGGAGTCAAAGGCATCAGAAGGAGGTGTGTGCAGACCGGCTGGCCCAGCTAGCACTTAAGGA

GGAGTTGAAAAACCAAGAGAAGAAGGAGGAACAGATGTTTGCAGATCTCTGGGAAGAGGACAGGTTGGCC

AAGGAAAAACGAGAGGCAGTAGATATCCAGAAACAAGCTGAACAGAATCAAGAAAGGCTGAATGTGCTTA

ATGCCCAGGTATCTGTTCTGAATGCTCAGAAGGAGGCGGCTAAGCGACTGAAAGAAGAGGAGGCACGATT

ACTGGAGGAAGAAAAGCAGCTGCTTAAGCTAGAGAATGAGCGACTTCAGTTGGAAAAATTGCAAAAACAG

AAGGAATGCAGAGACATGTTGGTCAGTTCCATACGAGAGAAGATGAAGCGTCTTAATCGAGAAAAACAAG

AAGAACTTGCTCTTGACATGAAGATTTTAGAACAAGTTCTCCAAGAATCCCAGGAAGACACTGAAGGAAA

AAAGAAAAAAAAACAAGAACTATTTAAGGAGCAGCAGATTTATCGGGAACATCTAGCTGCACAACTGGAA

GAGGAGAAGCGTCGGGAGAAGGAGATGGACAAGCTGCTGGAGGAAGAGATGGAGCGGACGTGGGCCAAGA

AGGCTGAGCAAATGAGACTGGAAAAGGAGGCTAGAAAACGGCTAATGAAAGATGTCCTGGATACAAGACG

ACTGCAGATTGAGGAGAAGTTGGAGAGAAATGCAAAGCAGCAGGAAAAGCTGGCTCAGGACAAGTTGCTG

CTGGCTGAAGCCATTAAAGAACTCAATCATATTGACGGAGAGAAATATTCCAGAAAAATACAGGAGGCAA

AGGAATACCGGGAGCAGCTTCAGGCTCAAATTGCCTATCAGCAGCAGGCCCACCATGCTGAGGAAGAAGA

GAAGCAGCGAGAGTATGAATCAGGTCTAGCAGCAGAGCAGGAATACCAAGAAAAGATCAAGCACATTCTG

TCCAGATCCCACCTGAAACTAACAGAAATCCATCCCTTGAGAAGAAAGCTAATGACTGACCTGCAAGTAT

AG

>Chinese alligator CTIF

ATGGAAAACTCCTCTGTGGCATCTGCCTCCTCTGAGGCAGGGAGTAGTCGATCTCAGGAAATTGAAGAGC

TGGAGAGATTCATCGACAGTTATGTCCTTGAGTACCAAGTCCAAGGGCTGCTGACTGACAAAACAGAAGG

GGACGGAGAGAGCGAGAAAACACAGTCCAATATCTCTCAGTGGACAGCGGATTGTAGTGAGCAGCTAGAT

GGCAGCTGTTCCCCATCCAGAGGGAAGGGCTCCTTGGCTAATGAACGCAATCAGAACGGCAACAAAGACA

GCTCCCTTGACATGCTGGGCACAGATATCTGGGCAGCCAATACCCTTGAGTCTTTCAGTGGTGCAACCTG

GGATCTGCAGCCTGAAAAGCTGGATTTCACACAGTTTCACCGGAAGCTGAGGAACACCTCCAAACACCCT

CTCCCCCACATAGACAGAGAAGGGCTTGGGAAAGGGAAGTATGAGGATGGAGACGGGATCAACCTGAACG

ACATAGAGAAGGTCCTTCCAGTCTGGCAGGGTTACCATCCATTGCCGCATGAAGCTGAAATTGCACACAC

CAAGAAGCTGTTCAGACGGAGGAGAAATGACCGGAGGCGACAGCAGAGGCCTCCAGGTGGAAACAAATCG

CAGCAGCACACGGATAATCAGCAAGGGAGCACCAAACACAACAGGGAACACCAGAAACTCTACCAGGGAG

GCCAAGCCCCGCACTCCTCGGGGAGGACAGGCCACCATGGGTACAGCCAAAACCGGCGATGGCACCACAA

CCAGAAGCACTCGCCCAATGACAAAGAAACGCACAGAAATGCCAAAGAGACTGAGAATCTGAAAATCGAG

GATGCCTCTGTCTGCACAGTGCACATCCCCCTGGAGATGCACCGGAGCACAGAAGCGGTGGAGAAGCAGT

CTCAGCAGTATATTCAGGAGCAGGAAACCAAGCGGAAAGATAGTATTCATGAGCGCATCGGGGAAAGGCC

CAAGATCAACTTGCTTCAGTCATCCAAAGACAGACTGCGAAGGAGACTAAAAGAAAAGGACGAAGTCACG

GTGGAGACGACCAACCCCGAAAAGAACAAAATGGACAAATTAATTGAAATCCTCAACAGCATGAGGAACA

ACAGCAGCGACGTGGACTCGAAGCTCACCACCTTCATGGAGGAGGCCCAGAACTCCACTAACTCAGAGGA

GATGCTGGGCGAGATTGTCAAGACCATTTACCAGAAAGCAGTCACGGACCGTAGCTTTGCCTCCACAGCA

GCCAAGCTGTGCGACAAAATGGCGCTCTTCATGGTGGAAGGGACCAAGTTCCGAAGTTTGCTCCTCAACA

TGTTGCAGAAGGATTTCACAATGCGGGAGGAGCTGCAGCAACGGGACGTGGAGCGCTGGCTGGGGTTCAT

CACCTTTCTCTGCGAAGTCTTCGGCACCATGAGGAGCAGCACTGGGGAGCCCTTCCGGGTCCTTGTCTGT

CCCATTTATACCTGCCTCAGGGAGTTGTTGCAATCTCAGGATGTGAAAGAAGACGCTGTCCTTTGCTGTT

CCATGGAGCTGCAAAGCACAGGCCGGCTGCTGGAGGAGCAGCTGCCCGAGATGATGACAGAGCTGCTGGC

GATAGCACGCGACAAGATGCTGTGCCCCTCGGAGTCCATGCTGACACGGTCCCTCCTCCTCGAGGTCATT

GAGCTGCACGCCAACAACTGGAACCCCCTGACGCCCACCATTACACAGTACTACAACAAGACAATCCAGA

AACTGACAGCCTGA

>Chinese alligator DCC

ATGGAGAATAGTCTTGGATGTGTTTGGGTACCAAAGCTGGCTTTTCTACTCTTCGGGTTTTCTCTGATCA

GCCTGCATCTTCAAGTCACCGGTTCACAGGTTAAGGGTTTTACATCATTACGGTTCCTGACTGAGCCTTC

AGATGCTGTCACCATGCGTGGAAGTAATGTGCTGCTGAACTGCACAGCGGAGTCAGATCAAGGAATTCCA

GTCATCAAGTGGAAGAAAGATGCTGTGTTCTTAAACTTGGCGGTGGATGAAAGGAGACAGCAGTTGGCCA

ATGGTTCCCTCTTGATACAAAACATAGTCCATTCCAGACACCACAAGCCAGATGAGGGTGTCTACCAGTG

TGAGGCATCTTTAGAAGGCACTGGGGCTATCATCAGTCAGACAGCTAAAGTCACAGTAGCAGGACCACTG

AGGTTTCTTTCCCAGACAGAATCTGTCACAGCTTTCACAGGAGACACCGTGTTGTTGAAGTGTGAAGTGG

TTGGAGAGCCCATGCCAACGGTACACTGGCAGAAAAACCAGGAGGACATGACCCTTAGCTTCAGTGACAC

CCGGATAGTTGTCTTGCCCTCTGGAGCTTTACAAATCAGCAGAATTCAAATGGGGGATAGTGGGATCTAC

AGATGCCTTGCAAAAAATCCAGCTAGTTCAAGAACAGGAAATGAAGCAGAAGTCAGAGTATTGGCAGATC

CAGGCTTGCACAGACAGCAGTTTTTCCTGCAGAGACCGTCAAACGTGATGGCTGTTGAAGGAAATGATGC

CATTCTCGAGTGTTGTGTTTCTGGATTTCCCACCCCCACTTTCACATGGATGCGTGGAGATGAAGTGATC

CCAGTCAGGTCCAAAAAGTATTCCTTATTGGCCGGCAGTAACTTACTTATCTCTAATGTGACTGATGATG

ATTCGGGGACATACACGTGTATAGTCACCTATAAGAATGAGAATAGCAGTGCCTCTGCAGAGCTCTCAGT

GATGGTTCCTCCATGGTTTTTAATTCGTCCTTCAAATCTCTATGCGTATGAGAGTATGGATATTGAGTTT

GAATGTGCTGTCTCTGGTAAGCCCGTCCCTACAGTGGATTGGATCAAGAATGGAGAAGTGGTGATTCCTA

GTGATTATTTTCAGATTGTGGGTGGCAGCAACTTGCGGATTCTGGGCTTAGTAAAGTCAGATGAAGGTTT

TTACCAGTGTGTAGCTGAAAATGATGCTGGCAATGCACAGACCAGTGCGCAGCTAATCATCCCCGAGCCT

GCTATCCCAAGTTCCAGTGTCCTCCCCTCTGCTCCCCGAGATGTGGTCCCTGTTTTGGTCTCCAGCCGAT

TTGTCCGTCTCAGCTGGCGTCCACCTGCAGAAACTAAAGGGACTGTTCAGACATACACTGTCTACTTCTC

CAGGGAAGGTGTCAACAGGGAGCGGGGACTGAACACATCTCAGTCTGGGATACTTCAGCTTACAGTCGGA

AATCTGAAACCAGAAGAGACCTACACGTTTCGAGTGGTGGCTTACAATGACTGGGGACCAGGGGAGAGCT

CGCAGCCCATTAAGGTTGCTACACAGCCTGAATTGCAAGTTCCAGGGCCAGTAGAAAACCTGCGGGCTGT

ATCTACCTCACCTACCTCAATTCTTCTTTCCTGGGACCCCCCTGCCTATGCAAATGGTCCAATCCAAGGA

TACAGACTGTTCTGCACAGAGACTGCCACTGGAAAAGAACAGAATATAGAGGTTGATGGACTATCTTACA

AGCTGGAAGGGCTGAAGAAATTCACAGAGTACACACTACGATTCCTGGCTTATAATCGTTATGGTCCTGG

GGTATCCAGTGAAGATATTACAGTCATGACACTATCTGATGTGCCAAGTGCAATGCCTCAGAACGTCTCC

TTGGAAGTGGCTAATTCAAGGAGCATCAAAGTTAGTTGGCTACCTCCACCATCAGGTACTCAAAATGGAT

TTATTACTGCCTATAAAATCCGACACAGAAAGACTAACCGCAGGGGTGAGATTGAAACGCTGGAGCCAAA

CAACCTCTGGTATTTGTTCACAGGACTTGAGAAAGGAAGCCAGTACAGCTTCCAGGTAGCTGCCATGACT

GTCAATGGAACAGGCCCCCCTTCTGAATGGTATACAGCGGAGACTCCAGAGAATGATCTTGATGAATCTC

AGGTTCCTGATCAGCCAAGCTCTCTTCATGTCAGGCCATTGATAAACAGTATCGTCATGAGTTGGACTCC

GCCACTGAACCCAAACATTGTTGTACGTGGATACATCATTGGCTATGGAGTGGGCAGCCCGTATGCTGAG

ACAGTGCGAGTGGACAGCAAACAGCGTTATTATTCCATTGAAAATTTGGAGCAAAGTTCCCATTATGTAA

TTTCTCTGAAAGCCTTTAATAATGCAGGTGAGGGAGTGCCTCTTTATGAAAGTGCCACCACTAGATCCAT

GACAGATCCCATTGATCCATTAGAAGTTGATTTTTATCCTTTGCTTGATGATTTCCCTACCTCAGTCCCA

GATATCTCCACCCCCATGCTCCCACCAGTAGGTGTCCAGGCTGTTGCACTTACCCATGATGCGGTGAGGG

TCATCTGGGCAGACAACTCTGTCCCAAAGAATCAAAAGACTACTGAGGTTCGGTTTTACACCGTCCGTTG

GAGAACCAGCTATTCTACCAGTGCTAAATACAAGTCAGCAGATACAACAGCTTTGAGTCACACTGTAATA

GGCCTTAAGCCAAATACCATGTATGAGTTCTCTGTCATGGTAACCAAAGGTCGAAGGTCAAGTACATGGA

GCATGACAGCTCATGCCACAACTTATGAAGCAGCTCCCACTTCTGCTCCCAAGGATTTGACAGTCATTAC

CCGGGAAGGGAAGCCTCGAGCTGTCATTGTTAGCTGGCAGCCTCCATTAGAAGCCAATGGAAAAATTACT

GCTTACATCCTGTTCTATACCTTGGACAAGAATGCTCCAATTGATGACTGGGTTATGGAATCAATCAGTG

GTGACCGACTTACCCATCAGATTATGGAACTCAACCTAGACACGGTGTATTATTTTAGAATCCAAGCTCG

CAATGCCAAAGGAGTGGGGCCTCTCTCTGATCCTGTTCTCTTCCGGACTCTGAAAGTTGAGCACCCTGAC

AAAATGGCTAATGACCAAGGTCGTCATGGAGATGGAGCCTACTGGCCAGTTGACACAAACTTGATTGATA

GAAGTAGCCTGAATGAACCTCCCATAGGGCAGATGCATCCTCCACACGGCAGTGTGACACCTCAGAAGAA

CAGCAACCTTCTGGTGATCATTGTAGTCACTGTTGGGGTCATTACAGTGGTGGTGGTGGTGATTGTGGCT

GTGATCTGTACCCGCCGTTCTTCAGCGCAGCAAAGAAAAAAACGTGCTACGCATAGTGCGGGTAAAAGGA

AGGGCAGCCAGAAAGATCTGAGACCCCCAGACCTCTGGATACACCACGAAGAGATGGAAATGAAGAACAT

TGAAAAGCCATCAGGGTCTGACCCCTCAGGAAGAGACTCACCAATGCAGAGCTGTCAAGACATCACTCCA

GTCAGCCATAGCCAGTCTGAAACCCAGATGGGCAGCAAGAGTGCCTCGCAGTCTGGTCCAGATACAGAGG

AGGTGGGAAGTAGTATGTCCACGTTAGAGCGTTCACTTGCTGCCCGCAGAGCCACTCGTGCCAAACTCAT

GATTCCAATGGATTCACAACCAAGCAACCCTCCTGTTGTCAGTGCCATTCCAGTACCAACACTGGAAAGT

GCCCAGTACCCAGGAATCCTACCATCTCCAACATGTGGATACCCACATCCACAGTTCACTCTTCGACCTG

TGCCATTCCCAACGCTCTCTGTTGACCGGACTTTCGGAACAGGAAGAACGGTGAATGAAGGACCAGCCTC

CCAACAGCCATCCATGCTGCCACAGACACAGCCTGAACATCCCAGCACTGAGGACGCCCCCAGCAGAACG

ATCCCTACAGCCTGTGTCCGGCCTACACACCCGCTCCGCAGCTTTGCCAACCCCTTGCTACCTCCACCAA

TGAGTGCAATAGAACCGAAAGTCCCTTATACACCACTTTTGTCTCAAACAGGGCCTAACCTTCCCAAGGC

TCAGGTTAAAACAGCATCCCTTGGATTGGCTGGGAAAGCAAGATCACCATTGCTTCCTGTCTCAGTGCCA

ACAGCTCCTGAAAAAGCAAGATCACCTTTGCTTCCTGTCTCAGTGCCAACAGCTCCTGAGGTTTCAGAAG

AGGGCCACAAGCAGACAGAAGACCCCGCTAATGTATATGAACAGGATGATCTGAGTGAACAAATGGCCAG

TTTGGAGGGGTTAATGAAGCAACTTAATGCTATCACAGGCTCAGCCTTCTAA

>Chinese alligator DYM

ATGGGAGCAAACAGCAGCAGCCTTAGTGAGCTGCCAGAAAATGAGTACTTAAAAAAATTGTCGGGAGCTG

AAGCAATCTCTGAGAATGACCCTTTCTGGAATCAGCTGCTTTCATTTAGTTTTACTACCCCAACAAACAG

TGCTGAGTTAAAGCTCTTGGAAGAAGCCACCATATCAGTCTGCAAGTCTTTAGTTGAGAACAATCCTCGG

TCAGGAAATCTTGGAGCGTTGATTAAAGTCTTCCTTTCTAGAACCAAGGAGTTAAAAATTTCAGCAGAAT

GTCAGAACCACCTCTTTATTTGGCAGGCCCACAATGCATTGTTTATTATTTGCTGTTTGCTAAAAGTGTT

CATCAGTCAGATGACAGAAGAGGAACTGCAACTTCATTTTACGTACGAGGAAAGAACACCTGGCTTATAT

GGAGTGGAATGTGAAGATCTGATAGAAGAATTGCTATGTTGTCTCATCCAGCTCATTGTTGAAATTCCTC

TTCTAGATATAACATACAGCATTTCCTTGGAAGCTGTGACGACACTGATTGTCTTCCTTTCCTGCCAGCT

ATTTCATAAGGAAATTCTGCGGGAGAGCATCATTCATAAATACCTGATGCATGGGCGATGTCTCCCATAT

ACCAGCAGACTTGTGAAGACATTGCTATATAACTTCATTAGACAAGAAAGGAGCCCTCCCCCAGGCACCC

ATGTCTTTCAGCAGCAAACAGACGGAGGAGGATTGCTTTATGGAATTGCATCAGGGGTGGCAACTGGCTT

GTGGACGGTGTTCACATTGGGTGGTGTGGGAAGTAAACCAACACCACAACAGGAACAGTCCTCACCTCTA

GCAAATCAAAGCCTTCTGCTACTGCTTGTGCTAGCTAATTTGACTGATGCACCAGATTCACCAAATCCAT

ACAAGCAAGCTATTATGTCCTTCAAGAACACACAAGATAGCACTGCTTTTCCTTCACCAAGTCCACATGC

TTTCCAGATTAATTTTAACAGTTTGTACACAGCTTTGTGCGAACAGCAAAAATCTGATCAAGCGACTCTC

CTTTTATACATGTTACTGCATCAGAACAGCAATGTTCGGACATACATGTTGGCCCGCACTGACATGGAAA

ATCTTGTTTTGCCCATTCTTGAGATTCTCTATCATGTTGAAGAAAGGAATTCACACCATGTTTACATGGC

TCTTATAATTTTGTTGATCCTTACAGAAGATGATGGCTTCAATCGATCTATTCATGAAGTGATATTGAAA

AATATTACTTGGTACTCGGAGCGTGTTCTAACAGAGATTTCACTGGGGAGTCTCCTTATACTGGTGGTAA

TAAGAACCATTCAGTACAACATGACAAGGACAAGGGACAAATACCTTCATACAAATTGTCTGGCAGCCTT

AGCAAATATGTCAGCACAGTTCCGTTCACTTCATCAGTATGCTGCTCAGAGAATCATCAGTTTGTTTTCT

TTGTTGTCCAAAAAGCACAACAAAGTTCTGGAGCAAGCCACACAATCCTTGCGAGGTTCCCTCAGTTCAG

ACGACTCTCCGCTTCCTGATTACGCACAGGATCTGAATGTGATTGAAGAAGTGATCCGAATGATGCTTGA

GATTATCAACTCCTGCCTGGCCAACTCTCTCCATCACAATCCCAATTTGGTATATGCACTGCTTTACAAG

AGGGATCTCTTTGAGCAGTTTCGAACTCACCCTTCCTTCCAAGATGTAATGCAAAATATAGACCTGGTGA

TCAGCTTTTTCAGCTCCCGGTTAGAGCAAGCTGGAGCTGAGTTGTCAGTGGAGCGAGTTCTGGAAATCAT

CAAACAAGGAGCTGTCGCATTGCCCAAAGATAGGCTAAGGAAATTCCCAGAGTTGAAGTTTAAGTATGTG

GAAGAGGAGCAGCCTGAGGAGTTTTTCATTCCCTACGTCTGGTCCCTGGTATACAATTCTGCCGTGGCGC

TGTACTGGAACCCCCACGACATCCAGCTCTTCACTATGGACTCCGGCTGA

>Chinese alligator DYNAP

ATGNNNTGGTCCATAATGAACATTTTCCTAATCTGTCTGTGGGTCTGTATAACATCTACCATAATGGGAG

TGCTGATCTGGTCCTTGGTCTACATTAAAAACGCTGGGAGTATGATACAGCCAAACATGCAAAAGCAGGA

AGTATCCTCCCCACAAACACCAGCAGGAATGTCAGACAGAAAGAGAGTGGATGTCAGATTCCAGTTTCTG

AATCATCTGGGTAAATCTAAGGTACATCATTTTCCTGGGGGTGATATTCAATGGGCAAGATTCAGGCATG

ATATAAATGAGTATCAGAGTGATGAAGAAATGGCATTTGGAACCAGCATCAACACCCGTCAGTCTGAGAT

GACTTTTGGCACCTTACAGATCAAGAGCAAAGGACTCCGGGCCCCACATTGGCATTTTAATGCCAATGAA

CATGGTTTTCTACTTAAGGGTACTGCCTGGATTGGTGTTGTTGATGCTGATGCCAGTACAGTAACCACAT

ACAATGTTACAGCTGGACAAGTGATTTTCTTCCCAAAAAATACATTGCACTGGATAAAAAATGTGGGAGA

AGATGACTGCTTGTTCTTGCTGTTTTTTACAACACATGAAGAACTTCAAACTCTGGATGTTGATGATGTA

TTTTTCTCTACACCAGAAGACATAGCAGCAAGGTCATTAAAGCCGGAAGGTGGAATTAGTTTTATTAGAT

CGTTCAAGAAACAGACAGAAGACCAGGCCATTAACCTACCACCGAACTTAATGGAACTGATTCAGAATGC

CAGCTATGTGCAGTCTCCAGACAAGCTTGTGTGGCGGTACTTCTACAACCTCAAAGCATCAGCAGAATAT

CGTTTTCCAGGAGGAATAATCCAGTGGGCTCGTTATCGCACAAATGGATCTGGTCTAAATAGCACTGAGC

AAATTTTCAGTGAGTCACTGAATAAGCATGAAAATACCCTTACCATAGGAACTCTCAGGATATATAGCAA

TGGACTACGTCAGCCTCATTTTCACTTCAATGCTAATGAGATGGGCTATGTCATTAGTGGCTGTGGAAAG

GTGGGCATTATTGTTTCATCTGAAGTTACCACAAACTTCGACATTGGTGTTGGAGATGTTGTATTTTTCC

CTCTTGGAACTCAACATTATATCAAAAGCATTTGTGATGAGGACTTGGTTTTGGTTCTAGCCTTCAGTAC

AGGCAACCAGCTTCAGACACTTGATATGGATGACTATTTCCATGCCACCGCAGACCATATTCTAGCCCAG

CTCTTCTGGAAGAAACAGGAGGAGTTCAAGAAGATTCCAAGGTTTAGGGAGGATCAGGCAATTAA

>Chinese alligator ELAC1

ATGTCCATGGATATCACTTTTCTGGGAACAGGTTCGGCATATCCATCTCCAACAAGAGGAGCCTCCGCTA

CAGTCATTCGTAATGAAGGAGAATGTTGGCTTTTTGACTGCGGAGAAGGAACTCAAACACAGTTTATGAA

AAGCCATCTAAAAGCAGGTAGAATTACAAAGATTTTCATAACCCACCTTCATGGCGACCATTTTTTTGGG

CTTCCTGGTTTGTTGTGCACAGTTAGTCTCCAAAGTAGTTCTTGTCCAAGCAAGCAGCATGTTGATATTT

ATGGGCCTGTAGGACTGAGAGACTTCATTTGGAGAACTATGGAGCTCTCCCACTCGGAGCTCCTGTTTTC

ATACGTTGTTCATGAACTAGTACCTACACCAGACCAATGCCCTCCAGAAGAGTTTAAAGAATTACCCTGG

CTAAACAGAGATGGAGGATCGTCCAAAGAAGTGCAAGGGAGAACAATTTATCTGGATTCTGTAGAAAACT

CCTACACAATGGTCAATGATGAACAGTTTGTTATGAAAGCCTTCCGTTTATTTCACCGCATTCCTTCCTT

TGGATTTCTAGTAGAGGAAAAACAGCGAATTGGTAAACTCAATGTACAGAAGTTAAGAGACCTTGGAGTT

CAGCCAGGTCCTGTATATGGGAAGCTGAAAAATGGTATTACAGTCGTCCTAGAAAATGGAATAACTATTT

CTCCTTCAGATGTCTTGGAAGATCCTATTCCTGGAAGGAAAATATGTATTTTAGGTGACTGTTCAGGTGT

TGTTGGAGATGAAGCAGTGAAACTTTGCTTTGAAGCAGATCTGTTGGTTCATGAAGCCACATTGGATGAC

AGTCAAGTGGACAAAGCCATTGAGTATGGTCACAGCACTCCAAGAATGGCAGCTGAGTTTGCAAAGCTAT

GTAAAGTGAAGAATCTGGTTCTGACTCACTTCAGTCAGAGATATAAACCGGCTGCTCTGGTGGGAGAAGG

AGACACTGATGTCACAGAACTAAAGAGACAAGCAGAAGCAGTATTAGCTGGCCAAGAAGTAACTTTAGCG

GAGGATTTTATGACAATAGACATTCCAATGAAAAAACAAAAATAG

>Chinese alligator FECH

ATGNNNNTTGTGAAAAGCAGAAGTCAGGTCACAGTCCAAATACAGTGGAGAGGTCAGGCAACAGCAGCCT

CAGAAACAAAAAGCACAAAGCTTCAAAGTCAGCCAGAAAAGAGGAACCCTAAAACTGGAGTTCTGATGTT

AAACATGGGAGGGCCAGAAACACTGGAGGATGTCCACGACTTCCTCCTTAGGCTTTTTCTAGATAAAGAC

CTAATGACGCTTCCTGTACAAAATAAGATGGCACCATTCATTGCCAAACGTCGCACACCAAAAATCCAAG

AACAATATAGCAAGATTGGAGGCGGATCACCAATTAAGAAATGGACTGCAGTACAAGGAGAAGGCATGGT

GAAACTGCTGGATGGAATGTCTCCTCACACTGCACCTCATAAATACTATATTGGTTTTCGGTATGTCCAT

CCTCTGACAGAAGAAGCAATTGAAGAGATGGAAAAAGATGGAGTTGAAAGGGCTATTGCTTTCACACAGT

ACCCACAGTACAGCTGTTCTACCACAGGAAGCAGCTTAAATGCCATTTATCGGTACTATAATAAAAAGGG

GGAGAAGCCAAAGATGAAATGGAGCATTATTGACAGGTGGCCTACACATCCCCTTCTCATTCAGTGCTTT

GCAGACCACATACAGAAAGAACTGGATCTGTTCCCACCAGAGAAAAGGAAAGAAGTTGTGATTCTTTTCT

CAGCTCACTCACTTCCAATGTCTGTTGTGAACCGCGGTGATCCATACCCTCAAGAGGTGGGAGCTACTGT

CCAAAAAGTCATGGAGAAGCTGAACTATTCCAACCCTTACAGGCTTGTTTGGCAGTCCAAAGTAGGACCA

ATGCCGTGGCTTGGTCCACCGACAGATGAAGCTATTAAAGGACTCTGTGAAAGGGGAAAGAAGAATATTT

TGTTGGTTCCAATAGCATTTACTAGTGACCACATTGAAACACTCTATGAACTGGATATTGAATATGCACA

AGGTGTAGCTAATGAGTGTGGAGTTGAGAACATCAGAAGAGCTGAGTCCCTTAATGGAAATCCATTGTTT

TCCAAGGCACTCGCAGACTTGGTCTATTCACATATCCAGTCAAATGAAATATGCTCCAAGCAATTAACCC

TCCGTTGTCCACTTTGTGTAAATCCTGTCTGCGGGGAGACAAAAGTCTTCTTCACTAATCAACAGCTATG

A

>Chinese alligator LIPG

ATGTGCCCATCCTTTGATCTGTTACCAGATGACACCACGGCTGCACACTCTCAAAGACTGCAAGTGAAGT

TTAATCTTCTCTCTTCAACACGTCCAGAGAATGAAGGCTGCTATCTCTCTGTAGACCAAGAAAAATGCCT

GGAGGACTGCAAATTCAATGCGACAGCTAAAACCTTCTTTATTATTCACGGATGGACAATGAGTGGCATA

TTTGATAAGTGGCTGAACAACCTAGTGTTTGCCCTCCAGAACAGAGAAAAGGATGCTAATGTGGTAGTGG

TAGACTGGCTGGTACTTGCCCATCAACTCTATACAGATGCTGTGAACAACACAGAGGTGGTTGGACAAAG

AACTGCAAAGTTGCTCAACTGGTTACAGGAAAAACAATACCTTCAGTTTGAAAACGTTCACTTAATTGGG

TACAGTCTTGGTGCCCATGTTGCTGGCTATGTTGGTAACTATGCAGCTGGGACAATAGGCAGAATTACAG

GCTTGGATCCAGCTGGCCCTATGTTTGAAGGAGTTGAACCTCACAGACGCCTCTCCCCTGATGATGCAGA

CTTTGTGGATGTCCTTCATACATATACGAGAGAAACGCTGGGCGTCAGCATTGGGATCCAGATGCCTGTG

GGTCACGTTGATATCTACCCCAATGGGGGCGATTTCCAGCCTGGTTGTGGATTAAGTGATGCTCTGGGAG

CAATCGCGTATGGGAATATTGGTGATGTTGTGAAATGTGAACATGAACGATCTGTGCACCTCTTTGTGGA

CTCTCTTGTGAACCAAGATAAGCGGAGCTTTGCTTTTCAGTGCACAAATTCCAGCCGTTTCAAGAAGGGA

ATCTGTCTGAGCTGCCGAAAGAACCGCTGCAACAGCATTGGCTACAACGCTAAGAAAATGAGGAATAAAA

GGAACAGCAAGATGTACTTAAAAACCAGAGCTGACATGCCTTTCAGAGTTTACCATTATCAGATGAAAAT

GCATGTTTTCAGCTATAAAAACTTAGGAGAAACTGAGCCTACCTTCTTGGTTACCCTTCACGGCACGAAC

GGAGACTCTCAACCCCTCTCTCTGGAAATACTTGACCAAATTGGCCTGAATTTCACAAACACTTTCCTGG

TTTATACTGAAGAAGATATTGGTGACCTCTTAAAGATCAAGCTTACTTGGGAAGGGTCATCTCAGTCCTG

GTACAGTCTGTGGAAACAGTTCAAGAGTTACTGGTCCCAACCTGAGAATTCCTCCAAGGAGCTGCACATC

AGACGTATACGTGTGAAGTCTGGGGAAACTCAACAGAAGCTTGCTTTCTGTGCTGAGGACCTTCATCAGA

CAGACATCTCTCCTGGCAAAGAGCTCTGGTTTGTGAAATGTAGAGATGGATGGCAAACAAAAAATAGCCC

AAGGTCAGCCTTGA

>Chinese alligator MAPK4

ATGGCAGAGAAGTGCGACTGCATTGCCAGCATGTATGGCTACGATCTGGGCTCCCGCTTCATCAACTTCA

GTCCCCTGGGCTTTGGTGTGAATGGACTGGTTCTGTCCGCAGTGGACAGCAAGAGCTGTCGCAAAGTGGC

GGTGAAGAAGATCGCCATCAGCGATGCACGGAGCATGAAACATGCCTTCCGAGAGATCAAAATCATCCGT

AGGCTTGACCATGACAACATCGTGAAGGTGTATGAGGTGCTGGGGCCAAAGGGGGCTGACCTGCAAGGGG

ATTTCTTCAAGTTTAACATGGTGTACATAGTCCAGGAGTACATGGAGACTGATCTGGCTCGGTTACTAGA

GCAGGGGAAGCTGACAGAGGAACACGCCAAACTCTTCATGTACCAGCTCCTCAGAGGGCTGAAGTATATT

CACTCAGCCAACGTACTACACAGAGACCTCAAACCTGCCAATATCTTCATTAGTACGGAGGACCTGGTGT

TGAAGATTGGTGACTTTGGGCTGGCGAGGATTGTGGATCAGCACTACTCACATAAGGGTTATCTTTCTGA

AGGCTTAGTAACAAAATGGTACCGGTCTCCACGCCTGCTTCTCTCCCCGAACAACTACACCAAAGCAATT

GATATGTGGGCTGCAGGCTGCATCCTGGCGGAAATGCTAACGGGAAGGATGCTGTTTGCAGGGGGCCACG

AGCTGGAACAGATGCAGCTTATTCTGGAGACGATCCCCGTCATCCGTGAGGAGGACAAAGAGGAGCTGCT

CAAGGTGATGCCCAATTTCATCAACAGCACCTGGGAGGTGAAGAAGCCTTTGCGTAAGCTGCTCCCTGAA

GTGAACAGCGAAGCTATTGACTTTCTGGAGAAGATACTGACATTTAACCCCATGGATCGATTAACAGCAG

AAATGGGTCTACAGCATCCTTACATGAGCCCCTATTCCTGCCCAGAGGATGAACCAGTTTCACAGCATCC

ATTCCGGATTGAGGATGAGATCGATGATATCTTACTGATGGAAGCCAACCAGAGCCAGATGTCAAACTGG

CACAGGTGCAGTTCAAGGTACCAAGTAAGTCTCTCTTCTGATTTGGAATGGCGACATGATAAATACCATG

ACATGGATGAGGTACAACGAGACCCAAGAGCAGGATCTGACTCGCTTGCCGAAGAAGCACAAGTTGATCC

ACGCAAATATTCTCAAAGCAGCTCAGAGAGGTTCTTAGAACTATCACATTCGTCGATGGACCGGGTGTTT

GATGTAGATTATGGAAGATCATGTGATTACAAAGTAGGGTCACCTTCCTACTTGGACAAACTGCTGTGGA

GGGACAATAAGCCCCATCACTACTCAGAGCCCAAACTGATCTTAGATTTATCCCACTGGAAAAAAGCAAC

TATAACACCCGCAACAGAGTTAGCACTGGAAGAAGAACCATCCAACCTCTTTCTAGAGATAGCTCAGTGG

GTGAAGAGCACTCAAGCAGGGCTCGAGTGCCCCAGTCCTCTTCCAGAGATGCAAGAACGCAGTCTGCCAC

CTTCTCCTCACCATCACCATAAAGAACCTAAGGAGGTAAAGAGTGAAACAGACCCTCAGTTTGATTTGGA

TGTCTTTATCTCTAGGGCACTGAAACTTTGCACCAAGCCTGAGGACCTCCCAGACAACAAGCTCAATGAC

ATCAATGGAGCTTGTATTTCAGAACATCCTAATGAGATTGTGCAAACAGAGGTGTTTCAAAAGGAAAGGT

GGTAA

>Chinese alligator MBD2

ATGGAGAAGCGCGGCAGGATGGACTGCCCAGCGCTGCCCCCGGGCTGGAAGAAGGAGGAGGTCATCCGCA

AGTCGGGCCTCAGCGCCGGCAAGAGCGATGTCTACTACTTCAGTCCAAGTGGTAAGAAGTTCAGAAGCAA

GCCTCAGTTGGCAAGATACCTGGGAAACACTGTTGATCTCAGCAGTTTTGACTTCAGAACGGGAAAGATG

ATGCCCAGTAAATTACAGAAGAACAAACAGAGACTAAGGAATGATTCTCTCAATCAAAATAAGGGTAAAC

CAGACTTAAATACAACGCTGCCAATCAGGCAAACGGCATCTATCTTCAAACAGCCAGTAACCAAAGTTAC

CAACCATCCTAGTAACAAAGTGAGATCTGATCCGCAACGAGTGACAGAGCAGCCACGGCAACTCTTCTGG

GAGAAGAGGCTACAAGGCTTAAGTGCATCTGATGTAAGTGAACAAATTATAAAATCCATGGAGCTACCTA

AAGGCCTTCAAGGAGTTGGCCCAGGTAACAATGACGACACCCTTTTGTCTGCTGTTGCCAGTGCTTTGCA

CACTAGTTCTGCACCTATCACAGGGCAGCTCTCTGCAGCTGTGGAAAAGAACCCAGCTGTTTGGCTTAAT

ACATCTCAACCCCTCTGCAAAGCTTTCATAGTTACGGATGAGGATATTAGAAAACAAGAAGAGCGGGTGC

AGCAGGTGCGTAAAAAACTGGAAGAGGCTCTAATGGCAGATATCTTGTCAAGGGCTGCTGATGCATCAAA

AGATATGGATGTAGAAATGGACAACGGAGATGAAGCATAA

>Chinese alligator ME2

ATGCTGTCCCGATTAAGAACAGTGGCCACTCCCTGTGTGCTGGCCTGCCGCAGGGTGCACACAAAGGAAA

AAGGCAAGCCACTCATGTTAAACCCAAGAACGAACAAGGGTATGGCATTTACACTAAAGGAACGACAAAT

GCTTGGGCTCCAAGGACTTTTACCTCCTAAAATAGAGACACAAGACATTCAAGCCTTACGCTTCCATAAG

AATTTGGCAAAAATGAGTGACCCCTTGGAAAAGTACATCTACATAATGGGAATCCAGGAGAGAAATGAGA

AGCTGTTCTATAGGGTGTTACAAGATGATATTGAGAGATTAATGCCAATAGTATACACACCCACCGTAGG

TCTAGCCTGCTCGCAGTATGGACATATCTTTAGGAGACCAAAGGGATTATTTATTTCTATCTCAGACAGA

GGTCATATTAGGTCAATTGTGGACAACTGGCCAGAAAATGATGTCAAGGCTGTTGTAGTAACCGATGGAG

AGAGGATATTGGGCCTTGGAGATCTGGGTGTATATGGAATGGGGATTCCTGTAGGAAAGCTGTGTTTATA

TACCGCATGCGCTGGGATACGGCCAGATAAATGCCTTCCTGTGTGTATTGACGTTGGAACTGATAATCCA

ACATTATTAAAAGATCCATTTTATATGGGCCTGTATCAAAAAAGAGACCGCTCACAATTTTATGATGATC

TGATTGATGAATTTATGGAAGCCATTGTAGACAGATATGGCCAGAACACGCTTATTCAGTTTGAAGACTT

TGGGAACCATAATGCTTTCCGGTTTTTGAGAAAATATAGAGAGAAATATTGTACCTTCAATGACGATATT

CAGGGGACAGCCTCAGTGGCTTTAGCAGGGCTGCTGGCTGCACAGAAGGTTACTGGTAAAGCAATCACAG

AGCATAAAGTTTTGTTTCTTGGAGCAGGCGAGGCTGCCCTGGGAATTGCAAACCTCATTGTTATGGCTAT

GATGGAAAATGGCCTTTCTGCAGAGGAAGCGCATGAGAAAATATGGATGTTTGATAAATTTGGTTTATTA

GTTCAGGGCCGAGAACAAAAGGTAGATTCCAATCAAGAATCGTTTACACACCGAGCACCCAATCAGGTGC

CAAAGACATTTGAGGATGCAGTGAATATACTTCAGCCTTCAGCTATTATTGGTGTTGCAGGTGCAGGTCG

GCTTTTCTCTCCTGACGTGATCAAAGCAATGGGTTCCATCAATGCAAGACCTATAATATTTGCGTTGAGT

AACCCTACGGTGAAAGCTGAATGCACTGCTGAAGATGCCTATACATTAACAGAGGGACGCTGTTTGTTTG

CCAGCGGCAGCCCATTTGAATGCGTGACTCTGAAGGATGGGAGGATCTTCAAACCAGGCCAGGGAAACAA

TGCCTATATTTTTCCAGGGGTGGCCCTGGCAGTAGTCCTCAGTGGTGTTCGGCATATTAGTGATAAGGTC

TTCCTGGAGGCTGCAAAAGCATTGACTGAGCAATTAACAGGTGAAGAACTTGCCCAAGGGAGACTTTACC

CCCCACTTTCCAACATCAAAGAAGTTTCCATTTGCATTGCTGTTAAAGTTATGGAATTTTTGTATGCGAA

CAACATGGCCTTCCACTACCCGGAGCCAGCAGACAAGAATCAGTATATTCAATCCAAGATATGGAGCTAC

GAATATGACTCTTTCATACCAGATGTGTATGACTGGCCTGAGTCACCAGATCAGCCTCCCAAAATGCACT

GA

>Chinese alligator MEX3C

ATGGTAGCAGTGGAGAACTACAGGCTATTTCTCTCATTTGTATTGATCACTTATGCTGACTTGGAGACAA

ATGAGCATGTCCACAAAGTGCTGTTGACTACACAAAGTTGCAAAATAAAAGCACTAAGGGCCAAGACAAA

TACTTATATTAAGACCCCTGTTCGTGGAGAAGAACCCATTTTTGTTGTCACTGGACGAAAAGAGGATGTA

GCCATGGCCAAAAGGGAAATTCTCTCAGCTGCTGAACACTTCTCCATGATCAGAGCATCACGCAACAAAA

ACGGTCCTGCCTTGGGAGGGTTACCATGTACTCCCAATTTGCCAGGTCAAACCACAGTTCAAGTCAGGGT

GCCTTACCGTGTAGTAGGGCTAGTGGTTGGACCCAAAGGAGCTACAATCAAAAGAATTCAGCAGCAAACA

CACACATACATAGTTACTCCCAGCAGAGACAAGGAGCCTGTCTTCGAGGTTACAGGCATGCCTGAAAATG

TGGATCGTGCACGTGAAGAAATAGAAATGCATATTGCCATGCGTACTGGAAACTACATTGAGCTGAACGA

AGAGAACGATTTCCATTACAACGGTACAGATGTCAGTTTTGAAGGAGGCACACTTGGATCTGCTTGGCTT

ACTTCCAATCCTGTCCCTCCTAGCCGCACCAGAATGATTTCTAATTACAGAAATGACAGCTCCAGCTCCT

TAGGAAGTGGCTCTACAGATTCTTATTTTGGAAGCAATAGATTGGCTGACTTCAGCCCAACAAGCCCATT

CAGCACAGGAAACTTTTGGTTTGGAGAAACATTGCCGCCTGTGGGGACAGAAGATCTTGCAGTTGATTCT

CCTGCATATGACTCTTTACCAACACCTTCCCAAACTATCTGGACTCCTTTTGAACCAGTAAACCCACTCT

CTGGCTTCGGTGGTGATCCTACTGGTAACATGAAGACTCAGCGTCGAGGAAGTCAGCCATCCACTCCTCG

TCTGTCGCCTACATTTCCCGAAACTCTGGAACACCCACTTGGTAGGAGATTAAGAAATGATCCACCCGGT

ACAGGCAACCAGGCTGGCCTTCCTATATACATCCCTGCTTTTTCTAATGGTACCAACAGCTACTCCTCTT

CCAATGGTGGTTCCACATCCAGCTCACCTCCTGAGTCGAGACGAAAGCACGACTGTGTGATATGCTTTGA

GAACGAAGTTATTGCTGCCCTAGTTCCATGTGGCCACAATCTCTTTTGCATGGAATGTGCCAACAAGATC

TGTGAAAAAGAAACGCCATCATGTCCAGTTTGCCAGACAGCTGTTACTCAGGCAATCCAAATTCACTCTT

AA

>Chinese alligator MYO5B

ATGNNNTCCCAGTACACAAGGGTTTGGATTCCTGACCCTGATGAAGTCTGGAGATCAGCAGAAATTATCA

AGGATTACAAAGAGGGAGACAAAAGCCTGCGCCTAAAACTTGAAGATGAAACTGTCTGTGAATATCCCAT

CGATCTCCAAGGCAGTCAATTGCCCTTCTTGCGGAATCCAGATATTTTGGTTGGAGAAAATGACCTGACA

GCTCTAAGTTACCTCCATGAGCCTGCAGTGCTGCATAACCTAAAAGTCAGATTCTTGGAATCAAACCACA

TATATACCTACTGTGGGATTGTGCTTGTTGCCATCAATCCATATGAACAGTTGCCAATCTATGAGCAAGA

TGTCATCTATGCGTACAGCGGCCAAAACATGGGAGACATGGATCCTCACATCTTTGCGGTGGCAGAAGAA

GCCTACAAGCAGATGGCCAGGGATGAGAAGAACCAATCCATCATCGTCAGTGGAGAGTCGGGTGCTGGAA

AAACTGTCTCTGCCAAATATGCCATGCGTTTCTTTGCCACTGTTGGGGGCTCTGCCAGCGACACCAATAT

CGAAACAAAGGTCCTCGCATCCAGTCCAATCATGGAGGCAATTGGAAATGCTAAAACCACCAGGAATGAT

AATAGCAGTCGTTTTGGGAAATACATTCAGATTGGCTTTGATAAGAGATACCATATCATCGGTGCCAATA

TGAGGACGTATCTCCTGGAAAAGTCGAGAGTTGTGTTCCAGGCAGAAGATGAACGAAACTACCATATCTT

CTATCAGCTGTGTGCCTCTGCCAGTCTTCCAGAATTTAAAGACCTTTCCCTAACATGTGCTGAAGACTTC

TTCTACACTTCTCAGGGAGGTGACACATCTATCGACGGGGTGGATGATGCTGATGATTTTGAAAAAACTA

GGCATGCTTTCACTCTACTTGGAGTGAAGGAGTCTCATCAGATGACCATTTTTAGGATAATTGCTTCCAT

CTTGCACCTAGGAAATTTGGAAATTCAAGCAGAGCGAGATGGAGATGCCTGCAGTATATCGAGCCAGGAT

GAACACCTGAATAATTTTTGCAGCTTGCTTGGAGTGGAGCACAGCCAGATGCAACACTGGCTGTGTCATC

GGAAGTTTGTCACAACAGCTGAGACGTATGTCAAGACCATGTCTGTGCAGCAAGTCGTGAATGCCAGGAA

TGCCCCAGCCAAGCATATATATGCCCAGCTGTTTAACTGGATTGTGGAGCATGTCAACAAAGCCCTCCAT

ACCACCATCAAGCAGCACTCATTCATTGGGGTCCTGGATATCTACGGGTTCGAAACTTTTGAGGTGAATA

GTTTTGAACAATTTTGTATCAACTATGCCAATGAAAAGCTCCAGCAGCAGTTCAACTTGCATGTATTTAA

GCTGGAGCAAGAAGAGTACATGAAGGAACAAATCCCTTGGACTCTTATAGATTTTTATGACAACCAGCCC

TGCATAGACCTTATAGAAGCTAAACTTGGTATCTTGGACCTGCTGGATGAAGAGTGTAAGGTTCCTAAAG

GAACTGACCAGAACTGGGCCCAGAAATTGTATGATAGACACTCTACCAGCCAACACTTCCAGAAACCACG

AATGTCCAACACCTCTTTCATTGTGGTGCACTTTGCAGATAAGGTTGAATATCAGTGTGAGGGATTTCTG

GAGAAAAACAGGGACACTGTATATGAAGAACAGATCAACATCCTGAAGGCCAGCAAGTATCAGATGGTAG

CAGACTTATTTCATGATGAAAAGGATTCCATGCCTGCTCCTCCTGTTGGAAAGGGAACCTCCTCCAAAAT

CAGTGTTCGTTCTGCCAGACCCGCTGTGAAAGCTGCCAATAAAGAGCACAAGAAAACCGTGGGACATCAG

TTCCGTAACTCCTTGCATTTGCTCATGGAAACGCTGAATGCCACCACTCCACACTATGTGCGCTGCATCA

AGCCAAATGATGAGAAACTCCCCTTTAAATTTGATCCAAAGAGAGCAGTCCAGCAGCTAAGAGCCTGTGG

TGTGCTAGAGACCATCCGCATCAGTGCAGCTGGCTACCCATCCAGGTGGTCCTACCATGACTTTTTGAAT

AGGTATCGTGTTCTTATGAAAAAGAGGGATATCTCTAAGAATGACAAGAAACAAATCTGTAAAACGCTGC

TGGAAGACCTCATTAAGGATCCTGACAAGTTCCAGTTTGGACGTACCAAGATCTTTTTCCGTGCAGGCCA

GGTGGCATATCTGGAGAAACTCCGGGCAGATAAATTTAGAGCTGCTACTATTATGATTCAGAAGACTGTG

CGGGGCTGGTTGCAGAGACTGAAGTACAGAAGGATGAAGGAGGCTGCAGTAACCATACAGAGGTATACAC

GGGGACATCTGGCTCGCAGGTTAGCAGACCACCTAAGGAAAACAAGAGCTGCCATCATCTTCCAGAAACA

ATATCGAATGCTCAGGATCTTTCGAGCTTTCCAGAATATCCGCCAGGCCACTATCACTATTCAAGCTTTT

GCTCGAGGCATGTTTGTCAGGAGAGTTTACCACAAGATACTCTTGGAGCACAAAGCTACCATCATCCAGA

AACACTCCCGGGCTTGGCTGGCTCGTAAACAGTTTCTACGCCTCAGGTGTGCCACTGTGGTTCTCCAGTG

CTACTACAGGCGCATGAAGGCCAAGCAAGAGCTAAAGGCACTGAAGATAGAGGCGCGATCAGCAGAGCAC

TTAAAGAGACTCAATATTGGCATGGAGAACAAGGTGGTTCAACTTCAGAGGAAGGTAGATGAACAGAACA

AAGAGTACAAGCTGCTGAACGAGCAACTCTCAGCACTTACCTCTGCCCACTCCACCGAGATGAAGAAGCT

GAAGAAGGAACTGGAGCGATACCAGCAGAACCAGGGGGATGGCAACCAGCTTGTTGATTTGCAAGAAGAG

ATTGAGAGCCTCCAGCTGGAACTCAAGAAAGCCCATAGTGAAAGGAAAATTATAGAGGATGCTTATGTTA

AGGAGAAGGATCTTCTCAAGAAGCGTGTATCAGACCTAGAAGAGGAGAATGCTCTTTTGAAACAGGAAAA

AGAGGAGCTTAACAACAGAGTCCTGTGTCAATCTGAAGATGAATTTGCACGCAACACTGTTGAGGAAAAT

CTGTTGAAGAAGGAGCTAGAAGAGGAAAGATCTCGTTATCAAAACCTTGTGAAGGAATTTTCGAGGCTGG

AGCAGAGATATGACAATTTGAGGGATGAGATGACTATTATAAAGCAAACCCCAGGGCACAGAAGAAACCC

CTCTAATCAGAGCAGTCTCGAGTCTGACTCCAACTACCCGTCATTCTCTACATCTGAGATAGGAGACACA

GAGGATGCAATACAACAAGTGGAGGAGGTTGGTATTGAGAAAGCAGCCATGGACATGACTGTCTTCCTGA

AACTACAAAAGCGAGTGAGGGAGCTTGAACAGGAGAGAAAGAAGTTGCAAGCCCAGCTGGAGAAGAGAGA

GAAAGAGAAAGAAAACAAAAGCTCCCAGGTAATTGAAATGAAGAATGAAATGGATTTGGACCGTGATACT

GATCTAGCATACAACAGTCTGAAGAGGCAAGAACTGGAATCAGAGAACAAGAAACTGAAAAATGAACTTA

ATGAACTAAGAAAGGCTATAGCAGACCAAGCAACCCAAAACAACTCTTCCAATGATATCCAAGACAGTTA

TAACCTCCTACTGAATCAGCTTAAATCAGCCAATGAAGAGCTGGAAGTGAGGAAGGAAGAAGTACTTATT

CTAAGGACACAGATTATAAACGCAGCCCAGGAAAAAGAGACCAGCAAAAATATGGAATNNNNGCTCCTTG

AAGCCCAGCTCCAGGATCAGAGACGAGAGCACGAAGAAGAGGTGGAGGCTCTGAAAGCTCAGGTGGAAGC

TATGAGAGAGGAGATGGAGAAACAGCAACAGGCCTTTTTCCAGACCCTTCAGCTCTCCCCAGAGGCCCAG

GTGGAGTTTGGTCTTCAGCAAGAAATCACACGTCTCACTAATGAAAATTTGGATCTCAAAGAGTTGTTGG

AAAAACTGGAAAAGAATGAGAAGAAGCTTAAGAAACAGCTGAAGATTTACATGAAGAAGGTCCAAGATTT

TGAAGCAACCCAAGCAACGGTACAGACAGAGAGGAAGCGACAGGAGCTTACCAGGCAAGTCACTGTCCAA

AGGAAGGAGAAGGATTTCCAGGGGATGTTGGAATATCACAAGGAAGATGAGCCACTACTCGTCCGGAACC

TCATTACAGATCTCAAGCCCCAGACCGTTTCCACCACAGTTCCCTGTCTTCCTGCTTACATCCTCTACAT

GTGCATCAGACATGCAGACTACGTCAACGATGACCAAAAAGTGCACTCCTTGCTCACCTCGACCATCAAC

GGCATTAAGAAAGTATTAAAGAAACACAATGATGACTTTCAGATGACGTCGTTTTGGCTGGCTAACACGT

GTCGCCTCTTGCATTGCCTAAAGCAGTACAGCGGGGATACGGGTTTTATGACACAGAACACTGCAAAGCA

GAATGAGCACTGTCTGAAGAACTTTGATCTCGCAGAGTATCGCCAGGTTTTAAGTGACCTCTCCATTCAG

ATCTATCAGCAGCTTATTAAGATAGCAGAAGGCATGCTGCAACCCATGATAGTGTCAGCAATGTTGGAAA

ACGAAAGCATCCAGGGCCTCTCTGGTGTGAAGCCAATGGGCTACAGGAAGCGCTCCTCCAGCATGCCGGA

TGGCGACAACACCTACAGCTTAGATGCGGTCGTTCGCCAACTGAACACCTTTCACAGCATCATGTGTGAG

CAGGGCCTGGACCCGGAGATCATCCAACAAGCCTTCAAGCAGCTGTTCTACATGATTAATGCTGTCACGC

TGAACAACCTCCTGCTGAGGAAGGACGTCTGCTCATGGAGCACTGGCATGCAGCTCAGGTTTAACATCAG

CCAGCTGGAGGAATGGTTGCATGGAAAGAACCTGCAACAAAGTGGAGCAGCACAGACCATGGAGCCCCTG

ATCCAGGCAGCACAACTTCTGCAGCTGAAAAAAAAAACTTCAGAAGATGCTGAGGCCATCTGTTCCTTAT

GTACATCCCTTACCACGCAGCAGATTGTAAAAATACTAAATCTCTACACTCCTGTGAATGAGTTTGAAGA

GCGTGTGACTGTAGCCTTCATAAGAAACATTCAGGCGCATTTGCAAGAGCGAAACGACCCTCCACAGTTG

CTGCTAGACTTCAAATACACGTTTCCAGTTTTGTTTCCATTCAGCCCATCCTCCATCACCATGGACTCTA

TTCACATCCCTGCTTCTCTCAACTTGGAATTTCTCAATAAAGTCTGA

>Chinese alligator NARS

ATGTATGTTTCTGAACGAGAGGGGAATGATTCTACTGGCAATGGAACAAAAGAGAAGCCTTTCAAAACTG

CTTTAAAGGCTTTGATGACAGTAGAAAAGGAGCCATTTCCTACTATTTACGTGGACTCTCAAAAAGAAAA

TGAGAGGTGGGATGTTATCTCAAAATCACAAATGAAGAATGTAAAGAAGTTGTGGCACCGAGAACAGATG

AAGAATGAAACTAAAGAAAAAAGAGAGGCAGAAGACCTCTTACGACGAGAAAAAAACCTGGAGGAAGCTA

AGAAGATCATCATCAAGAAAGATCCCAGTCTTCCGGAGCCCAAATGTGTGAAGATTCGTGATCTGGTAGC

ACACAGAGGCCAACGAGTGAAGGTTTTTGGCTGGGTTCACAGGTTACGTAGACAAGGAAAAACGTTGATG

TTTCTGGTGTTAAGGGATGGCACAGGGTTTCTTCAGTGTGTTCTCTCTGATGAGTTGTGTCAGTGTTACA

ATGGATTGATCCTCTCAACAGAGAGCAGTGTTGTAGTGTATGGGATGTTAAACCTTGTTCCTGAAGGCAA

GCAGGCTCCAGGAGGCCATGAGTTGAGTTGTGACTTTTGGGAGCTGATTGGTCTGGCTCCAGCAGGAGGA

GCTGACAACCTAATCAATGAGGAGTCTGAGGTAGATGTGCAGCTTAACAACAGGCATATGATGCTCCGAG

GCGAGAACATGGCCAAGATCTTTAAGGTGCGCTCCGTAGTTATGCAGTGCTTCAGGGATCACTTCTTTGA

CAATGGCTACTACGAGGTCACACCACCAACTTTAGTGCAAACACAGGTTGAAGGAGGCTCCACACTATTC

AAACTGGATTATTTTGGTGAAGAGGCCTTTTTGACACAGTCATCCCAGCTGTATCTGGAGACCTGCATCC

CTGCATTAGGAGACACTTTTTGTATTGCCCAGTCATACAGAGCTGAGCAGTCCAGGACACGCAGGCACCT

GGCAGAATATACTCACATTGAAGCTGAATGCCCTTTCATAACCTTCGAAGACCTGTTGGATCGTCTGGAG

AATCTGGTTTGTGATGTAGTGGACAGAGTCTTGAAATCACCTGTGTCAGCCTTCTTGTATGAGCTAAACC

CGAACTTCCAGCCCCCTAAACGCCCTTTTAAACGAATGAACTATACAGATGCCATCGTTTGGCTAAAGGA

ACATGATGTGAAGAAGGAAGATGGCACTTACTATGAATTTGGAGAAGATATCCCAGAAGCTCCTGAGAGA

CAGATGACAGACACCATTAATGAGCCAATCTTGCTGTGCCGATTTCCTGAGATAAAGTCCTTTTACATGC

AGCGTTGTCCTGAGGATTCCCGGCTTACTGAATCTGTTGATGTGTTGATGCCTAATGTTGGTGAGATTGT

TGGAGGCTCAATGCGTACCTGGGACAGTGATGAGCTTTTAGAGGGTTATAAAAGGGAAGGCATTGATCAC

ACACCATACTACTGGTACACAGATCAGCGTAAATATGGCACATGCCCTCATGGTGGGTACGGCCTGGGCT

TGGAGCGATTCTTAACATGGATTCTGAATAGATACCACATCCGTGACGTATGCCTGTACCCGCGCTTTGT

ACAGCGCTGCAAACCATAG

>Chinese alligator NEO1

ATGTGGCTTTTCCAAAATATTCAGGGGCATATTTTCCGCTCATCCAGTGTGAGAACCTTCACGCCCTTCT

ATTTTGTGGTGGAGCCCACAGACACGTTATCTGTTCGGGGAGCTTCAGTCGTACTGAACTGTTCAGCTTT

TTGTGAGACTTCTCCAAAAATTGAGTGGAAAAAAGATGGAACTTTTCTCAACTTGGTGTCAGATGATCGT

CGCCAGCTGCTACCTGATGGATCTTTATTAATAAATAGTGTAGTGCATTCCAAACACAATAAGCCTGATG

AAGGATACTATCAGTGTGTGGCGACTGTGGAAAGCCTTGGGACCATTGTAAGCAGAACAGCCAAGCTCAC

AATAGCAGGACTTCCTAAGTTCATCAGTCAGCCAGAATCGACATCTGTCTATAGAGGAAACAGTGCAATT

CTTAACTGTGAAGTTAATGCTGATCTAGTACCATTTGTGAGGTGGGAGCAGGCCCGTCAGCCACTTTTTC

TGGATGACCGCGTATTTAAACTACCGAGTGGAGCTCTAATAATTAGCAATGCAACGGATGGGGATGAAGG

ACTGTATCGTTGCATCATTGAAAGTGGTGGGTCCCCCAAATACAGCGATGAGGCAGAGCTTAAAATTCTT

CCAGATCCAGAGTTTCCCTCAGACTTGGTGTTTCTGAGGCAACCCTCTTCGCTTATCAGAGTTACTGGGC

AGAGTGCAGTTTTGCCATGTGCTGCTGCAGGATTTCCAACTCCAGTTATCAGGTGGACAAGAAATGAAGA

AGAGCTTATCACAGAAGGCTCTGAAAGGTTTCTTTTGCTGGCGGGAGGTAGCCTAGAGATCAGTGATATC

ATGGAAGATGATGCTGGAACATACGCCTGCATCGTGGACAATGGGAATGAGACAGCTGAAGCTCAAGCAG

ATCTTACAGTTTTAGCTCCACCTGAATTTCTGAAGCAGCCTGCTAATATTTACGCTCATGAATCCATGGA

TATTGTGTTTGAATGTGAAGTGACTGGGAAACCAACTCCAACTGTGAAATGGGTCAAGAATGGCGACATG

GTGATCCCTAGTGACTACTTCAAAATTGTTAAGGAACACAACCTGCAGGTCTTGGGTCTGGTGAAATCAG

ATGAAGGATTCTATCAGTGCATTGCAGAAAATGATGTTGGAAACGTGCAGGCTGGAGCCCAGCTAATAAT

ACTTGAACACGATGTTGCCATCCCAACATTACCTCCCACTTCACTGACCAGTGCCACTACTGACCATCTA

GCACCAGCTACAACTGGACCGTTGCCTTCAGCCCCTCGGGATGTCGTGGCCACCCTCGTCTCTACTCGCT

TCATCAAACTGACGTGGCGGACACCTGCATCAGATCCTCAGGGAGACAACCTTACCTATTCTGTATTCTA

CACCAAGGAAGGTATAAACAGGGAACGTGTTGAAAATACAAGTCGCCCTGGGGAGATGCAAGTGACAATC

CAAAACCTGATGCCAGAAACAGTCTACATTTTTAGAGTTGTGGCTCAAAATAAACATGGCCCTGGAGAGA

GCTCAGTACCATTGAAAGTGGCAACTCAGCCTGAAGTTCAACTTCCTGGTCCAGCACCCAACATTCGAGC

ATATGCCAACTCACCCACTTCAATCACTGTCACCTGGGAAACGCCATTGTCTGGCAATGGAGAAATTCAG

AACTACAAGCTTTATTACATGGAAAAGGGGACAGATAATGAGCAGGATGTTGATGTGGGAGGTCACTCCT

ACACCATTAATGGCTTGAAGAAATACAGAGAGTATAGCTTCCGAGTGGTGGCCTACAATAAACATGGCCC

TGGAGTGTCTACCCAAGATGTTGTTGTGCGAACACTGTCAGATGTTCCCAGTGCTCCACCCCAGAATCTG

TCCCTGGAAGTCCGAAATTCCAAGAGCATCATGGTACATTGGCAGCCCCCTCCTCCTGGGACCCACAATG

GTCAAATCACTGGCTACAAAATTCGCTACCGTAAAGTTTCTCGTAAGAGTGATGTAACAGAGAGCATTGG

AGGGACACAGATTTTTCAGCTAATTGAAGGTCTTGAGCGAGGTACAGAATACAGCTTCCGGGTGGCTGCC

TTAACAATCAATGGTACAGGACCAGCTACTGACTGGGTATCAGCAGAAACTTTTGAGAGTGACTTGGATG

AAACTCGCGTCCCTGAAGTACCCAGTTCGTTACATGTCCGTCCACTGGTTACCAGTATAGTAGTAAGCTG

GACTCCTCCTGAAAATCAGAATATTGTGGTGAGAGGATACGCTATAGGGTATGGTGTTGGCAGTCCTCAT

GCACAGACCATCAAAGTGGACTACAAACAGAGATATTACACCATTGAGAACTTAGATCCAAGCTCCCACT

ATGTCATAACTCTGAAAGCTTTCAACAATGTTGGCGAAGGCATCCCACTCTATGAGAGTGCAGTGACCAG

GTCACAGACAGACACTTCTGAAGTTGATGTATTTGTTATTAATGCTCCATACACTCCAGTGCCAGATCCC

TCCCCCATGATGCCGCCAGTGGGCGTTCAGGCTTCCATTCTGAGTCATGACACCATAAGGATCACTTGGG

CAGACAACTCTCTGCCGAAGAATCAGAAGATCACAGATGCTCGCTACTACACAGTGCGCTGGAAAACCAA

CATTCCTGCAAACACCAAGTACAAGACTGCAAACACGACCACTTTGAGCTATTTAGTGACTGGGTTAAAA

CCTAACACCTTGTATGAATTCTCTGTGATGGTGACTAAAGGTCGAAGATCAAGCACTTGGAGTATGACAG

CACATGGAACCACCTTTGAATTAGTTCCTACCTCTCCTCCAAAGGATGTGACTGTAGTAAGCAAAGAAGG

AAAACCTCGGACCATAATTGTGAACTGGCAGCCTCCATCTGAAGCTAATGGCAAAATTACAGGATACATC

ATTTACTACAGCACAGATGTGAATGCTGAGATACATGATTGGGTCATTGAACCTGTTGTAGGAAACAGAT

TGACTCACCAGATCCAAGAATTAACCCTTGATACACCATATTATTTCAAAATCCAGGCACGCAACTCCAA

AGGCATGGGACCTATGTCAGAGGCAGTCCAGTTCAGAACACCCAAAGCTTCGGGAACTGGAGGTAAAGGA

AGTCGGCCAGTGGATCTAGGACCGGACTACAAACCACCAATGAGTGGCAGTAACAGTCCTCACGGAAGCC

CCACATCGCCCTTGGATAGCAACATGCTTCTAGTAATCATAGTATCTGTTGGAGTGATCACCATTGTGAT

AGTGGTGATTGTTGCCGTCTTCTGCACTCGTCGTACCACTTCTCACCAGAAAAAGAAACGAGCTGCCTGC

AAATCAGTGAATGGGTCTCATAAATACAAAGGAAACTCCAAGGATGTCAAACCCCCAGACCTCTGGATCC

ATCATGAGAGACTGGAGCTAAAACCTATTGATAAATCTCCAGACCCCAACCCAATCATGACAGACACCCC

AATTCCTCGTAACTCTCAAGACATCACACCAGTTGACAATTCCATGGATAGCAATATCCATCAAAGGCGG

AACTCTTACCGGGGACATGAGTCGGAGGACAGCATGTCCACACTGGCAGGAAGAAGGGGGATGAGGCCAA

AAATGATGATGCCGTTTGATTCTCAGCCACCTCAGCCTGTGATTAGTGCTCATCCCATCCATTCACTTGA

TAACCCTCACCATCATTTCCACTCCGGCAGCCTCGCTTCTCCAACTCGCAGCTATCTTCATCATCAGGTC

AACCCATGGCCGATTGGCACGTCCATGTCCCATTCAGACAGGGCCAATTCTACAGAATCTGTTCGAAACA

CACCTAGCACAGACACGATGCCAGCTTCCTCATCTCAGACCTGTGCTGACCATCAAGATCCAGACAGTGC

CACAGGATCATATCTGGCCAGTGCACAAGAGGAGGATTCAAGTCAAAACCTCCCTACTGCCCATGTCCGT

CCCTCTCACCCTCTGAAGAGCTTTGCAGTGCCAGCAGTTCCACCCAGCAGTTCCACATATGACCCTGCCT

TGCCAAGCACACCATTACTGTCCCAGCAAGCTTCCAACCATCCAGTTCACTCGGTGAAGACTGCATCGAT

TGGGACTTTAGGAAGAACTCGACCTCCAATGCCAGTGATAGTCCCCAGCGCCCCTGATGTGCAGGAGACC

ACTCGAATGCTTGAGGATTCGGAAAGTAACTATGAACCGGATGAGCTGACCAAAGAGATGGCCCACCTGG

AAGGACTGATGAAGGACCTTAATGCCATCACTACCGCATGA

>Chinese alligator ONECUT2

ATGAACCCGGAGGCGGTGACCATGGACATGGGCAGCCTGCACGGCGCGCCTGCCGGCCATGAGCAGGAGC

TGCTGGGCAGCCCCAGCCCGCACCACGCGGGGCGGCGCGAGCTGGGCTCCCGCATGGTGCCCGGCATGGC

CTCGCTGCTGGACGCCGGCGGCGCCGGGGACTACCGCGACCTGCCGCTGCCGCTGCACCACGCCATGAGC

ATGCCCTGCGAGGCGTCGCCGCCCGGCATGGGCATGAGCAGCACCTACACCACGCTCACGCCGCTGCAGC

CGCTGCCGCCCATCTCCACCGTGTCCGACAAGTTCCACCCGCACCCGCACGCGCACCCGCACCCGCACCC

GCACGCGGCCCCGCACCCGCACCCGCACCCCGCGCAGCGCCTGCCCGCCAACGTCAGCGGCAGCTTCACC

CTCATGCGCGACGAGCGCGGGCTGCCGGCCATGAACAACCTCTACGGGCCCTACAAGGAGATGCCGGGCA

TGGGGCAGAGCCTGTCGCCGCTCGGCTCGCTGCACGCCCCGCAGCAGGGCCTGCACGGCTACGGCCCGCC

CGCCCACGACAAGATGCTCAGCCCCGCCGGCTTCGACGCCCACCCGGCCATGCTGGCCCGCGGCGACCAG

CACCTGCCCCGCGGGCTGGGCACGCCCCCCAACATGCTGCCCCACTTGAACGGCATGCACCACCCGGGCC

CGCCGCCGCCCCACGGGCCCGTGCTGTCGGCCGGCCGCGACCGGCCGCCCTCGTCCGGCTCGCAGGTGGG

CGGCTCGGGGCAGCTGGAGGAGATCAACACCAAGGAGGTGGCGCAGCGGATCACGGCGGAGTTGAAGCGC

TACAGCATCCCCCAGGCCATCTTTGCGCAGAGGGTGCTGTGCCGGTCTCAGGGCACCCTTTCCGACCTGC

TAAGGAACCCTAAGCCTTGGAGTAAACTGAAATCTGGCAGGGAGACGTTCAGGAGGATGTGGAAGTGGCT

GCAGGAGCCGGAGTTTCAGAGGATGTCGGCGCTGAGGCTCGCAGCATGCAAACGTAAAGAACAAGAACCG

AACAAAGAGAGGAACAATTCCCAGAAGAAATCTCGCCTAGTTTTCACCGATCTCCAGCGCAGAACACTTT

TTGCCATCTTCAAGGAGAACAAACGTCCATCCAAAGAAATGCAGATCACCATTTCCCAACAGCTGGGCTT

GGAACTTACCACTGTCAGTAACTTCTTCATGAATGCTAGGAGGCGCAGTCTTGAGAAATGGCAGGACGAT

TTGAGTACAGGGGGATCCTCTTCAACCTCCAGCACTTGTACCAAAGCATGA

>Chinese alligator POLI

ATGGCAAGGGCTTTGGCAGCAGGCAGTACAGCACACAGAGTGATTGTGCATATTGACCTGGATTGCTTTT

ATGCACAAGTAGAAATGATCCATAATCCTGAATTGAGAGAGAAGCCTTTAGGTGTACAACAGAAATACTT

GGTTGTAACCTGCAATTATGAGGCTAGAAAACTTGGAATTAAGAAACTTATGTCTGTCAGAGATGCTAAG

GAAAAATGTCCACAGCTGATACTAGTTAATGGAGAAGACTTAACTCCTTACAGGGAAATATCATACAAGG

TCACAGAACTATTGGAAGAATTTAGTCCACTGGTGGAAAGGCTTGGGTTTGATGAAAATTTTGTGGATAT

CACAGAGATGGTAGAGAAGAAACTAGGACAGCTACAAAGGGATGGATTGTCCAAAGTGTCTGTGTGTGGT

CACGTGTATAATGATCAATCTATCTGTTTACAAGATATGATGCATGTAAGACTTATCATTGGATCTCAGA

TTGCAGCAGAAATGAGGGAAACCATGCACAACAGACTGGGTCTTACAGGGTGTGCAGGAGTGGCTTCTAA

TAAGTTGCTATCAAAACTAGTATCTGGTACCTTTAAACCAAATCAACAGACAGTACTTCTGCCTGAGAGT

TGTCACCACCTAATAGAAAGCCTTGATCACATCAGAAAAGTGCCCGGCATTGGCTTTAAGACTACCAAGC

GCCTTGAGTTGCTGGGTCTCAGTAGTATACGTGATCTCCAAACCTGTTCTCCTGAGATATTAGTAAAGGA

ACTGGGAGTTGCAGTTGCTGAGCGGATCCAAAAACTCAGCTGTGGAGAGGATGATTCTCTTGTGACCCCC

TCTGGACCTCCACAGTCTTTTAGTGATGAAGATTCCTTTAAAAAATGTTCATCAGAAGTGGAAGTTAAAA

AGAAAATTGAAGAATTACTCACTAATCTTTTAGACAGAATACACAATGATGGAAGAAAACCACGTACAAT

AAGATTAACTATTCGTCAGTTTTCACTAACTAATAAATGGTTTAATCGGGAGAGTCGTCAATGCCCTATT

CCACCGCACATCATCCAGAAATTTGGGACAGGAACCTATGATGTTGTGTCCCCACTGGTTGATATCCTTA

TGAAGCTGTTTCGAAAGATGATAAACGTGGAGATGCCGTTTCATATTACCCTTCTGAGCGTCTGTTTCTC

CAACCTCAAAGCCCTGCCTAGCTGCACCAGAGGATCCATCGGATTTTATCTAACTCGGACATCATCCCAT

GCAAGTGCTCGCACATGTATTCAAGAAATGGAAAGTGTTCCACAAGACCAGGCACACTGTTGCAGTTCTG

CACCAGCTGAAAGAACTGAAAGTACAACAGACGGAAAACCTTCACTAGAAACCAAAAGCTTCACGGAGGA

AACAAGAATTCCCGAATACCCACTTCACTTGCTTCCTGCTGGTCTTGACCAGGAAGTCTTCAGTCAGCTT

CCAGTAGATATTAAAGAAGAAATTATTTCTGGCAGCAGCGGGGCAAAGATTCATACAGAAAATATTTTGG

ATCCACCATTACTTGTTTCTACACAAGCACCACCATATTTTGCCCAAGAAAAAATGCAAAACGCTTTCCC

AAATTTCCACAAAGTAGATCATAGTATCAGCTTTTCAGCGTGCACTGAATTCTTTACATCTACCATTGAG

CCAGTGACAGCCATGGTACAGAGGTCTACTCCAGGTTGTACTCCTGCGAATTCCCTAATGGATGACTGCA

TAGAACAAGAGCCCACAGAATCACTCCATTTCAAACAGAAAGACTCAGTTGTTTCAAGCCTGCAAAACCA

ACAACCTTTTCTGCACCCTCATTATTCAGGCGCACAGGAACAGGCTCTTGAAAAGGTTTCTCAGGCTGAA

GGTTCTAGAGGACAAGAATCACGTGAAGTAAAAATTGTATTCCCTGTTAATGTTGACCCAAAGACTTTTT

TTGAACTACCTACAGAAGTGCAAAAAGAACTACTGGCTGAGTGGAAGAATCAAGAGCCTGTTTCCAAAAT

GCGCATGAATAAACCATGTGAAAAGCTAAAAACGAGCAAAGGAAAAAAGAATGCAGCATTGTGTTCATCA

CAGTCTAACAGTTTACTAAGGTATTTTAAACCAAACTGA

>Chinese alligator RAB27B

ATGACTGATGGGGACTATGACTATCTGATCAAACTCCTGGCCCTTGGAGACTCTGGGGTTGGGAAGACAA

CATTTCTGTATAGATACACAGACAACAAGTTTAACCCCAAATTCATCACAACAGTAGGAATCGACTTTCG

GGAAAAGCGTGTGGTATACAACAGCAGAGGACCCAATGGATCTTCAGGAAAAGCCTTCAAAGTACATCTC

CAGCTTTGGGACACAGCTGGACAGGAAAGATTTCGAAGTCTCACCACAGCATTTTTCAGAGATGCCATGG

GCTTTTTATTAATGTTTGATCTCACCAGTCAACAGAGCTTCTTAAATGTCAGAAACTGGATGAGTCAACT

GCAAGCCAATGCCTATTGTGAGAATCCTGATATAGTATTAATTGGTAATAAAGCTGACTTATCAGATCAG

AGGGAAGTAAATGAAAGACAAGCAAAAGATCTTGCAGACAAATACGGCATACCCTACTTTGAAACAAGTG

CAGCTACTGGACAAAATGTGGAGAAGTCCGTGGACACACTGCTGGACTTGATAATGAAACGTATGGAACA

ATGTGTTGACAAGACACAAGTATCTGACACAGCAAATGGAGGAAGCTCGGGGAAGCTAGAATCAGCAAAA

CCAGAGGACCGAAAGTGTGCCTGCTAG

>Chinese alligator RPL17

ATGGTGCGCTACTCCCTGGATCCGGAGAACCCCACCAAGTCCTGCAAGTCCAGGGGCTCCAACCTCCGGG

TCCACTTTAAGAACACTCGGGAAACTGCTCAAGCTATCAAGGGCATGCATATCCGGAAAGCCACCAAGTA

CTTGAAGGATGTGACCCTAAAGAAACAGTGTGTTCCTTTCCGTCGGTACAACGGTGGAGTTGGTAGATGT

GCCCAGGCCAAGCAGTGGGGCTGGACACAGGGCCGTTGGCCTAAAAAAAGCGCTGAGTTCTTACTGCACA

TGCTCAAGAACGCCGAGAGTAATGCTGAGCTTAAAGGTCTTGATGTGGATTCTTTGGTAATAGAGCACAT

CCAGGTGAACAAGGCCCCTAAAATGCGCAGACGTACCTACAGGGCCCACGGTCGGATCAACCCCTACATG

AGTTCCCCCTGTCACATTGAGATGATCCTCACTGAGAAGGAGCAGATTGTTCCTAAACCAGAAGAGGAAG

TTGCACAAAAGAAAAAGATATCCCAGAAGAAGCTGAAGAAGCAGAAGCTTATGGCTCGGGAGTAA

>Chinese alligator SKA1

ATGGCTTCTTTAAATATGGAAGACTTATGCTCTTACATCAATACAAGGATTTCAACGATCAAGAAAACTC

TTCAGTTAAGAAACATAGATCAGGAGCCATCCTTGAAATCTGTGCTATGTAAAATCGGACATGAGATGTC

TCTTTTAAATGAACTCCTGAATAGAATGGAAATAGAAGTTCAGCATCAAGAAAAACTGAAAAATACACTT

AAGGAGATCCAGGAGTCTGCTGAGAGGGACTATAATGAAGTGCTGCACCTGTGTGAAAATATGCCTCCTC

ACCTACCCAAAACAAACCAAAGCTGTACCACGGGACTATCTGGAAAGTCTGAAGAACAGAGCAACAGCAC

AGAACCTGAACGTGCAAAGAAATCTGTAAAAGAGCCAAAATTTATTAAAGAAGTGCCCATAATAACAGCA

GAAGAATTTGAAAACGTTCCTGCGTATATGCGAGGTCGTCTAACATATGATCAAATAAATGGAGTTATTG

AAGATATGAACAGGGCTGTGATAAGCAAGTATAAGATCCTACATCAGCCAGTAAAATCAATGAACTCTGC

GGCCAGAAGTCTATACAACAGATTTTTAGAAGAAGAAACAAAAGATACTAAAGGTGAGTTTTTTATTGTG

GAAGCTGATATCAAAGAATTCACTCAACTGAAGGTGGACAAGCGCTTTTATAGCATCCTGAACATCCTGC

GCCACTGCCAGAGAGTGCGAGAAGTCCGTGGCTCACGGCTTGTCCGTTATGTTATCTGCTAA

>Chinese alligator SMAD4

ATGGACAATATGTCTATTACTAACACACCAACAAGTAATGATGCTTGTCTGAGCATTGTTCACAGCTTGA

TGTGCCATCGACAAGGTGGAGAGAGTGAAACTTTTGCAAAACGAGCAATTGAAAGTTTAGTTAAAAAGCT

AAAGGAGAAAAAAGATGAATTGGATTCTTTGATTACAGCTATAACTACAAATGGAGCTCATCCTAGTAAA

TGTGTTACAATACAGAGAACATTGGATGGAAGGCTTCAGGTGGCTGGTCGTAAAGGGTTCCCTCATGTGA

TCTATGCTCGTCTTTGGAGGTGGCCTGATCTTCACAAAAATGAACTCAAGCATGTTAAATATTGTCAGTA

TGCTTTTGACTTAAAATGTGACAGTGTCTGTGTGAATCCTTACCATTATGAGCGTGTGGTGTCCCCGGGC

ATCGATCTCTCAGGACTGACACTGCAGAGTTCTGCTCCATCAAGTATGCTGGTGAAAGACGAATATGTTC

ATGACTTTGAGGGGCAGCCATCGTTATCTTCTGCGGAAGGTCATTCAGTCCAAACCATTCAGCATCCACC

AAGTAACAGGGCAGCCACAGAGACGTACAGCACCCCAGCAATGTTAGCTCCTTCTGAGTCTAGTGCTACG

AGCACCACTAATTTTCCCAACATTCCTGTGGCTTCCACAAGTCAACCTACCAGTATATTGACAGGTAGCC

ATAGTGATGGACTCCTGCAGATCGCGTCAGGACCTCAGCCAGGAGCACAGCAGAATGGGTTTGCAGCTCA

GCCAGCTACTTACCATCACAATAGTACTACAACTTGGACTGGAAGCCGAACAGCAGCCTACACACCTACT

ATACCTCACCATCAGAATGGACATCTTCAGCATCACCAACCTATGCATTACTGGCCAGTTCATAATGAAC

TTGCATTCCAGCCTCCTATATCAAATCATCCTGCTCCAGAGTACTGGTGTTCGATTGCTTATTTTGAAAT

GGATGTGCAGGTTGGGGAGACATTTAAAGTTCCTTCAAGCTGTCCAGTTGTTACGGTTGATGGATATGTG

GACCCTTCTGGCGGAGACCGCTTTTGCTTGGGCCAGCTTTCCAATGTACATAGAACAGAAGCCATTGAGA

GAGCAAGGTTGCACATAGGTAAAGGGGTGCAGCTGGAATGTAAAGGTGAAGGTGATGTGTGGGTTAGGTG

CCTCAGTGACCATGCAGTCTTCGTTCAGAGCTACTACTTGGACAGAGAAGCTGGGCGGGCGCCAGGAGAT

GCTGTTCACAAGATTTACCCCAGTGCATATATAAAGGTGTTTGATTTGCGGCAATGTCATCGTCAGATGC

AGCAGCAGGCTGCCACTGCACAAGCTGCAGCTGCTGCTCAGGCTGCTGCAGTAGCAGGAAACATCCCTGG

GCCAGGATCAGTGGGTGGAATAGCCCCAGCTATCAGTTTGTCAGCTGCTGCTGGAATTGGTGTAGATGAC

CTTCGCCGTTTATGCATACTCAGGATGAGTTTTGTGAAAGGTTGGGGACCTGATTACCCAAGGCAGAGCA

TCAAAGAAACACCCTGTTGGATTGAAATCCATTTACACCGTGCGCTCCAGCTTCTAGATGAAGTACTTCA

TACCATGCCTATCGCAGACCCACAACCTCTAGACTGA

>Chinese alligator SMAD7

ATGTTCAGGACCAAACGCTCGGTGCTCGTCCGGCGACTCTGGAGGAGCCGCGCTCCCGGCGGGGAGGACG

AGGCGGGCGACGCCGCCGCGGACAGCCGGGCGCATGGGGCCGGCGGCGGCCGGGGCTGCTGCATGGGCAA

GGCGGGCAAGCTCGGCAAGGCGGCGCTGGGCTCCGAGGCGGAGCTGAAGGCGCTGACCCACGCCGTGCTC

AAGAGGCTGAAGGAGAAACAGCTGGAGGGGCTTTTGCAAGCCGTGGAGTCCCGAGGGGGCACCCGGACCT

CCTGCCTCCTGCTGCCCGGCAAGGTGGACTCCAGACTGGGTCAGCATTGGTACTCCCTCCCTTTGCTGCT

ATGTAAAATATTCAGGTGGCCCGATCTCAGGCATTGCTCGGAAGTCAAGAGGTTATGTTGCTGTGAATCT

TATGGAAAAACTCATCCAGAGCTGGTCTGCTGCAATCCCCATCACCTTAGCAGGCTCTGCGAACTAGAGT

CTCCCCCTCCGCCCTACTCCAGATATCCAATGGATTTTCTCAAACCAACTGCAGATTGTCCAGACTCTGT

GCCTTCCTCCACTGAAACAGGGGGAACTAATTGTCTAGCCCCTGGGGGGCTTTCAGATTCCCAAGTTCTT

CAGGAGCCGGGGGATCGGTCACACTGGTGCGTGGTGGCATACTGGGAAGAGAAAACGCGCGTGGGTCGGC

TGTATTCTGTCCAAGAGCCCTCCCTGGATATCTTCTATGATCTACCTCAGGGGAACGGTTTCTGCCTCGG

ACAGCTCAATTCAGACAACAAAAGCCAGCTGGTGCAGAAAGTCCGCAGCAAAATTGGTTACGGCATCCAG

CTGACCAAGGAAGTGGACGGCGTATGGGTATACAACCGCAGCAGTTACCCAATCTTTATCAAGTCGGCCA

CACTGGACAACCCCGACTCCAGGACGTTGCTGGTTCACAAAGTGTTTCCAGGATTTTCCATCAAGGCTTT

TGACTATGAGAAGGCGTACAGCTTACAGAGGCCTAACGACCATGAGTTCATGCAGCAACCATGGACCGGC

TTTACTGTTCAGATCAGCTTTGTGAAGGGCTGGGGCCAGTGCTACACAAGACAGTTTATCAGCAGTTGCC

CGTGCTGGTTGGAGGTTATTTTTAATAACCGATGA

>Chinese alligator ST8SIA3

ATGCTCCTTTGCAGGTCCCAGTTTGCACTGAAGTTTCTGGACCCTTCCTTTGTGCCGATCACCAATTCTT

TGACACACGAGCTACAAGAAAAGCCTTCCAGGTGGACCTTTAACAGAACAGCATTCTTACATCAAAGGCG

AGAAATTCTTCAGCATGTTGATGTGATAAAAAATTTTTCTTTGACCAAGAATAGTGTTCGGATTGGACAG

CTGATGCATTATGATTATTCCAGCCATAAGTATGTTTTTTCTATTAGCAATAACTTCAGATCATTGCTTC

CAGATGTCTCACCTATCCTGAATAAGCATTATAACATTTGTGCTGTGGTTGGAAATAGTGGAATCCTTAC

AGGGAGTCAGTGTGGACAAGAAATAGATAAATCTGATTTTGTTTTCCGTTGTAATTTTGCTCCAACTGAG

GCTTTCCAGAGAGATGTTGGAAGGAAAACCAACCTTACAACCTTCAACCCCAGCATCCTGGAAAAGTATT

ACAACAACCTTTTGACCATTCAGGATCGCAATAACTTTTTTCTAAGTTTAAAAAAGCTTGATGGGGCCAT

TCTTTGGATCCCAGCTTTTTTCTTCCACACTTCAGCAACCGTCACAAGAACATTGGTTGACTTCTTTGTT

GAGCACAGAGGGCAATTAAAGGTCCAGTTGGCTTGGCCAGGGAATATAATGCAACATGTCAACAGGTATT

GGAAAAATAAACATTTGTCACCCAAGCGACTGAGCACAGGTATTCTAATGTACACCCTTGCTTCTGCTAT

ATGTGAAGAAATCCATTTGTATGGCTTTTGGCCATTTGGATTTGATCCCAACACAAGGGAAGACCTCCCG

TACCATTACTATGATAAAAAAGGAACAAAGTTTACTACCAAGTGGCAGGAGTCCCACCAGCTGCCTGCAG

AGTTCCAGCTGCTCTACAAAATGCATGGGGAAGGACTCACTAAACTGACTTTGTCACGTTGTGCCTAA

>Chinese alligator STARD6

ATGGACTTCAAGAAAATTACGGATGAAGTTTCCAAAAAAATGTTGTCATACAGTCAAGATACATCAGGAT

GGAGAGTAATAAAAGTTTCGAAAAATGTTACAGTTTCTTCAAAACCTTCAAAAGAATATGCAGGACATCT

ATACTGTGGAGAAGGGATAATTAAGGAGGTACCTTCTAAAGTTATTCCCTTTATGTATCTTCCTGAACAT

CGATCCAAGTGGGACAAGGCATTACAGTCTTACCGGCTGCTAGAAAAGATTGATCAGGACACGGGCATAT

ATCACAGTGTTACACAGAGTTATGGTATGGGACTGATTTCGTCAAGAGATTTTGTTGATGTGGTACACAT

TAAGCCATATGCTGGAGGTATCCTTACTACTAACTCTGTCAGTGTGGAGTATCCAGAATGCCCTCCGTCT

CCCTCTTGCATCCGTGGTCATAACAACCCTTGTGGCTATGTTTGTTCCCCACTGCCAGAGAACCCAGAAC

ATTCCAAACTAGTTGTTTTTATCCAGCCAGAATTGGGAGGAATCTTGCCTCATTCAGTGGTGGAAACAGC

ATTGCCAACTATCCTCATAAACTTAATCACTGACACAAGGGCTGGACTAAAGACGTTAAATAA

>Chinese alligator TCF4

ATGCATCACCAACAGCGAATGGCTGCCTTAGGGACGGACAAAGAACTGAGTGATTTACTGGATTTCAGTG

CGATGTTTTCACCTCCTGTGAGCAGTGGGAAAAATGGACCAACTTCTTTGGCAAGTGGACATTTTACTGG

CTCAAATGTAGAAGACAGAACTAGCTCAGGGTCCTGGGGGAATGGAGGACATCCTAGTCCATCCAGGAAC

TATGGAGATGGGACTCACTATGATCACATGGCAAGCAGGGACCTGGGGTCACATGACAATCTTTCTCCTC

CTTTTGTCAATTCCAGAATACAAAGTAAAACAGAAAGGGGTTCATATTCATCATATGGAAGAGACTCAAA

TTTACAGGGTTGCCACCAGCAAAGTCTCCTTGGAGGTGACATGGATATTGGTAACCCAGGAGCTCTCTCA

CCAACCAAACCTGGCTCTCAGTACTACCAATATTCTAGCAATAACCCCCGAAGGAGGCCTCTTCACAGTA

CCTCTATGGAAGTACAAACAAAGAAAGTTAGAAAAGTTCCTCCAGGTTTACCATCTTCAGTTTATGCCCC

GTCAGCAAGCACTGCCGACTACAATAGGGATTCACCAGGTTATCCATCCTCAAAACCAGCAGCCAGCACT

TTTCCTAGCTCCTTCTTCATGCAAGATGGTCATCACAGCAGTGACCCATGGAGCTCCTCCAGTGGGATGA

ATCAGCCTGGTTATGGAGGAATGTTGGGCAATTCTTCTCATATTCCACAGTCAAGTAGCTACTGCAGTCT

GCATCCACATGACCGTTTGAGCTACCCATCACACTCTTCAGCAGACATCAATTCCAGTCTTCCTCCAATG

TCCACTTTCCACCGTAGTGGTACAAATCATTACAGCGCTTCTTCCTGCACACCACCTGCTAATGGGACAG

ATAGTATAATGGCAAACAGAGGAAGTGGGGCAGCAGGCAGCTCGCAGACTGGTGATGCACTGGGGAAAGC

GCTTGCCTCTATCTATTCTCCAGATCACACCAACAACAGCTTTTCATCAAATCCTTCAACTCCTGTTGGT

TCTCCCCCTTCTCTCTCAGCAGGCACAGCTGTTTGGTCTAGAAATGGAGGGCAAGCATCATCATCTCCCA

ATTATGAAGGTCCCTTACACTCTTTGCAAAGCCGGATTGAAGACCGCTTGGAAAGACTGGACGATGCTAT

TCATGTTCTCCGGAACCACGCAGTGGGGCCTTCAACAGCCATGCCTGGTAGTCATAGCGACATGCATGGA

TTAATAGGGCCTTCTCATAATGGAGCAATGGGAGGTCTTGGGTCAGGATATGGGACAGGCCTTCTTTCAG

CCAATAGACATTCACTAATGGTTGGAGCCCATCGTGAAGATGGTGTCAGTCTGCGTGGCAGCCATTCACT

TGTGCCAAATCAGGTTCCAGTTCCCCAGCTGCCAGTTCAGTCTGCTACTTCCCCAGATTTAAATCCACCC

CAAGATCCATATAGGGGCATGCCAACTGGACTGCAAGGGCAGAGTGTGTCTTCAGGTAGCTCCGAAATCA

AATCTGATGATGAGGGAGATGAAAACCTCCAGGACACAAAATCTTCTGAGGACAAGAAACTAGAGGATGA

CAAGAAGGATATCAAATCAATTACTAGGTCAAGATCTAGCAATAATGACGACGAGGACCTTACCCCGGAG

CAGAAGGCTGAGAGAGAGAAGGAAAGGAGAATGGCCAATAATGCCCGGGAGCGCCTACGCGTGCGTGACA

TCAATGAGGCTTTCAAGGAGTTGGGCCGGATGGTGCAACTCCATCTGAAGAGTGACAAGCCCCAGACCAA

ACTCCTGATCCTACACCAGGCTGTGGCTGTCATTCTCAGCTTAGAACAGCAAGTCAGAGAAAGGAATCTG

AACCCTAAAGCAGCATGTCTGAAAAGAAGGGAAGAAGAGAAAGTATCTTCAGACCCTCCTCCACTTTCCT

TGGCAGGACCCCATCCTGGGATGGGAGATGCATCCAATCATATGGGACAGATGTAG

>Chinese alligator TSPAN3L

ATGGACTGCGGCGTGATCACCTCCAAGACCGTGCTGCTGCTGCTCAGCCTCGTCTTCTGGGCGGCAGCAG

CTGGTCTTAGCTATGTTGGGGCATATGTCATTAACACCTACAAGAATTACGACCACTTCCTGCAGGACAA

GTATGCTCTGCTGCCAGCTGTGATCATCATTTGTGTTGCTGTAGTGATGTTCATCATTGGACTGATTGGC

TGTTGTGCCACCATCCGGGAGTCCCGTGTTGGGCTGGGTTTTTTTCTGGTCATTATTTTGATTATCTTTG

TTGCAGAAGTCTCTGCTTTTGTCCTGGGATTCATTTACAGAGAAAAGGTCAAAACAGATGTGCAAAGCAC

AATGCGTGCAGTCTTCCAGAAATATGATGGGAAAAGCGCAGAGGCTGATGTTGTGGATTACCTGCAAGAA

CAGTTTCAGTGTTGTGGAGTGAATAACTACTCTGACTGGACAACCACCCCATGGTTTAATAGCACTGGTA

ATAACAGCGTTCCTGTGAGCTGCTGTAAACAGGATATTAAGAACTGCACAGGACAACTGAGTGAACCACA

GGATCTCAATACTGAGGGCTGTGCACCGAAGGTGGAACTTGGGCTGCAGAGCGTTCTCAGCTATGTTATG

CTTGTAATCCTGGGATTTGCCATCATAAAGTTCTTTGGAATGATGAGTGTCTGTGTGCTCACATGCAAGA

GGGAGGACAGTGGCTACCAGCCCTTGTATTCGGGGGTGTTTGCGTGATAA

>Chinese alligator TXNL1

ATGGTCGGCGTGAAGGTGATCGCGAACGACACCGAGTTCCAGCCCGAGCTCAGCGCCGCCGGCTCCCGCC

TCGCCGTGGTCAAGTTCACCATGCGCGGATGTGGCCCGTGTTTAAGGATAGCTCCTGCTTTCAATGCTCT

GAGTAACAAATATCCCCAGGCAACGTTTTTGGAGGTAGATGTACACCAATGCCAGGGAACAGCTGCAACC

AATAATATATCAGCAACACCAACATTTCTGTTTTTCCGAAACAAAGTGCGAATTGACCAGTATCAAGGAG

CAGATGCTGTAGGATTAGAAGAAAAAATTAAACAGCATCTAGAGAATGATCCTGGAAATAACGAGGACAC

CGATATTCCAAAAGGATATATGGATTTAATGCCATTTATTAATAAATCCGGCTGTGAATGTCTTAACGAA

AGTGATGAGCATGGGTTTGATAACTGTTTACGTAAAGACTCTACCTATCTGGAATCAGACTGTGATGAGC

AGCTGCTTATTACTGTAGCTTTTAATCAGCCTGTCAAGCTTTATTCTATGAAGCTTCAAGGACCAGATAA

TGGTCAAGGTCCTAAGTATGTAAAAATCTTTATAAACCTCCCACGGTCAATGGATTTTGAAGAGGCAGAG

AGAAGTGAACCAACCCAAGCCCTGGAACTAACACCAGATGACATTAAAGAGGATGGTATTATCCCACTTC

GCTATGTAAAGTTTCAGAATGTTAACAGTGTAACTTTATTTGTCCGGTCAAATCATGGTGATGAAGAAAC

AACAAGAATTACATATTTTACGTTTATTGGAACTCCAGTCCAGGCAACAAATATGAATGACTTCAAGCGA

GTAGTAGGCAAGAAAGGAGAGAGTCACTAA

>Chinese alligator WDR7

ATGGCAGGAAATAGCCTTGTTCTACCTATTGTCCTTTGGGGTCGCAAAGCACCCACTCACTGCATATCAA

CCCTTCTGTTAATGGATGATGTGTCAATGATTGTCACAGGATGCCATGATGGACAGATATGTCTCTGGGA

CCTTTCATTAAATCTGGAGATTAATCCCAGAGCTCTGTTGTTTGGTCACACAGCCTCAATTACTTGTTTG

TCCAAGGCCTCTGCCTCCAGTGAAAAACAGTATATAGTGAGTGCATCTGAAGGCGGGGAGATGTGTCTCT

GGGATGTGAATGATGGGAGATGCATAGAATTTACTAAATTAGCCTGCACACACACTGGCATACAGTTCTA

TCAGTTTACAGTTGGGACTCAACGTGAAGGGAGGCTGCTATGCCATGGGCATTATCCAGAAATTCTTGTT

GTGGATGCTACCAGCCTTGAGGTCCTGTATTCTTTAGTGTCAAAGATTTCTCCTGACTGGATCAGCTCTA

TGAGTATCATTAGGTCCCACAGAACACAAGAGGATACTGTTGTGGCAGTTTCAGTGACTGGTATTCTGAA

AGTATGGATTGTAACTTCTGAAGTTAGTCGCATGCAAGATACAGAGCCAATATTTGAAGAGGAGTCTAAA

CCAATTTATTGTCAGAACTGCCAAAGCATTTCCTTTTGTGCATTTACGCAGAGATCTCTTTTGGTAGTAT

GCTCAAAGTACTGGAGGGTGTTTGATGCTGGAGATTATTCCCTGTTATGTTCAGCTCCCAGTGAGAATGG

ACAGACCTGGGCTGGCGGTGACTTTGTATCGGCTGATAAAGTGATCATATGGACAGAAGATGGCCAAAGT

TTTATTTACAAACTACCAGCCAGTTGCCTACCAGCTAGTGATTCATTTCGCAGTGATGTTGGAAAAGCTG

TTGAAAACTTAATTCCTCCTTTGCTGTACAGTGTATTGGACAGAGCAAATAAGCAGTTACTAATTTGTCC

TCCTGTCACTCGGTTCTTCTATGGACGCAGGGAATGTTTCCATAAACTTCTAATCCAAGGAGACTCTTCA

GGGAGACTGTGTATTTGGAGCGTGCCAAATACACTTGAGCAACAAGACATTGCTGAAGGGTTGCAAATGA

CAGTTTCAACTTCCTTGCAAGAAGCTTTTAATAAACTCACTCCCCATCCTGCTGGAATTATTGATCAATT

GAGTTTAATACCAAACAGCAGTGAACCGCTCAAAGTTACAGCTAGTGTGTATATTCCAGCACATGGACGC

CTGGTTTGTGGTCGTGAAGATGGAAGCATTATTATTGTGCCAGCAACACAAACTGCTATAGTTCAGCTTC

TTCAAGGAGAGCATATGTTCAGGAGAGGTTGGCCACCTCACAGAGCTCTTAGAGGTCATCGTAACAAAAT

CACGTGTTTACTGTACCCTCATCAGGTTTCATCTCGTTATGACCAAAAATATTTGGTATCAGGTGGTGTG

GATTTTTCAGTCATTATATGGGACATATTTTCTGGCGAAATGAAACATATTTTCTGCGTGCATGGCGGAG

AGATAACGCAGCTCCTAGTTCCACCGGAAAACTGTAGTGCAAGAGTCCAGCACTGCATTTGCTCTGTTGC

CAGTGACCACTCAGTAGGACTTCTGAGTCTACGAGAGAAAAAATGCATCATGCTTGCTTCTCGTCATCTT

TTCCCTATTCAAGTAATTAAGTGGAGACCTTCAGATGATTACCTGGTGGTAGGATGTTCAGATGGATCTG

TGTATGTCTGGCAAATGGACACTGGTGCACTGGATCGTTGTGTGATGGGAATAACAGCTGTTGAGATCTT

GAATGCTTGTGATGAGGCAGTTCCTGCTGCAGTAGACTCCCTCAGTCACCCAGCTGTAAACCTGAAACAG

GCCATGACTAGACGTAGCCTTGCTGCTTTAAAAAATATGGCCCACCAGAAATTACAGACCCTTGCCACTA

ACCTTCTGGCTTCTGAAGCATCTGACAAAGGGAATTTGCCTAAATATTCACATAACTCTCTGATGGTTCA

AGGTATAAAGACTAACCTAACAGACCCAGATATCCACATCCTCTTCTTTGATGTAGAAGCTCTGATTATT

CAGCTACTGACAGAAGAAGCATCTAGGCCTAACACTGCATTTATTTCCCCAGAGAATTTACAGAAAGCAT

CTGGTAGTTCTGACAAGGGGGGCTCTTTTCGGGCTGGCAAACGAGCAGCAGTTCTCTTCCAGCAGGTCAA

GGAAACAATCAAAGAGAACATAAAAGAACATCTTCTGGATGATGATGATGAGGATGAAGAAGCAATGAGG

CAGAGAAGAGAAGAGAGTGACCCGGAATATCGCTCCAGCAAATCTAAACCATTAACCCTATTAGAATATA

ATCTTACCATGGACACAGCAAAACTATTTATGTCCTGTCTCCATGCCTGGGGTTTGAATGAAGTTCTCGA

TGACATTTGCCTTGATCGCCTTGGGATGCTTAAACCACACTGTTCTGTGTCCTTTGGTCTTCTATCTAGA

GGAGGCCATATGTCTCTGATGCTTCCTAGCCATAACCAGTTGATGGGCAAGCCATCCTATGATGGTGTAG

AATTAGCAAGGAAAATGTCTATTACAGAGGGACTGGGAAGGGGGACTTATGGAGTCTCACGTGCAGTCAC

CACCCAGCATCTCCTGTCTATTATCTCTCTGGCAAATACCTTGATGAGTATGACTAATGCAACTTTTATT

GGAGATCATATGAAGAAAGCACCAACCAGGCCTCCAAGGCCAGGCACTCCAGAGCTGTCTAAAGTGAAGG

CTCCTCCACCCCCGGTTTCAAGTCCTGCAGCTCAAGGGCAAATTAAACAAGTTGCTCCTGCTGTTTCTGC

TAGCACTGAGGCTGGTCACTCTGGCTCTGACCCTGTTCCTACTTTACATACCTGTTTCTTAGTAAATGAA

GGATGGAGTCAATTAGCTGCTATGCACTGTGTCATGCTCCCTGATCTATTGGGATTGGATAAATTTAGAC

CTCCTCTTCTTGAAATGCTTGCACGCAGGTGGCAAGATCGATGCTTGGAGGTAAGAGAAGCTGCCCAGGC

CTTATTGTTAGCAGAACTGAGAAGAATTGAACAGGCAGGACGGAAAGAAACAATTGATGCATGGGCTCCA

TATTTGCCACAATATATTGACAGTGTGTTATCCCCTGGAGTGACAGCAGAAGCTATTCAGACTGGTACTG

CAAGTCCAGATTCATCCAGCACAGAAGCCAAAGTTCAAGAAGAAGAGCATGATCTGGTTGATGATGACAT

CACAGCAGGTTGTCTGTCTAGCCTCCCACAAATTAAAAAGATATCTACATCATATGAAGAGAGACGGAAG

CAAGCTACTGCTATTGTCTTGCTAGGAGTAATTGGAGCAGAATTTGGAGCTGAAATAGAACCTCCAAAGT

TACTGACCAGACCGCGCAGTTCCAGTCAGATTCCTGAAGGGTTTGGTTTAACCAGTGGTGGATCAAATTA

TTCCTTGGCAAGGCATACCTGCAAGGCATTGACATTTCTGCTGCTGCAGCCACCCAGCCCCAAACTTCCT

CCACACAGCACCATTCGCAGAACTGCAATTGATCTGATTGGGCGAGGTTTTACTGTATGGGAGCCATACA

TGGACGTCTCTGCTGTATTAATGGGCCTTTTGGAGCTCTGCGCTGATGCTGAGAAACAGCTTGCTAACAT

CACAATGGGGTTGCCTCTGAATCCAGCAGCTGACTCCGCCCGCTCTGCACGACATGCTCTCTCACTCATT

GCCACTGCCAGACCACCCGCCTTCATAACAACTATTGCCAAGGAGGTTCACAGACACACTGCCTTGGCAG

CAAACACTCAGTCTCAACAAAATATTCACACCACAACCCTTGCTCGAGCTAAAGGAGAGATCCTAAGAGT

CATTGAAATTCTTATTGAAAAGATGCCGACAGATGTGGTTGATCTCCTTGTAGAGGTTATGGACATCATC

ATGTATTGCCTTGAAGGATCCTTAGTCAAGAAAAAGGGACTTCAGGAATGCTTTCCAGCCATATGCAGGT

TCTACATGGTCAGCTATTATGAGCGGAGTCACAGAATAGCAGTGGGAGCTCGCCATGGTTCAGTAGCCCT

CTACGACATCCGGACTGGAAAATGTCAGACTATCCATGGCCATAAAGGACCAATAACTGCTGTTGCTTTT

GCTCCTGATGGACGGTATCTTGCCACTTATTCCAACACAGATAGCCATATCTCCTTTTGGCAGATGAACA

CCTCTCTCCTGGGAAGTATTGGAATGCTGAATTCGGCACCCCAGCTCCGCTGCATTAAAACATACCAGGT

GCCCCCTGTCCAGCCTGCCTCCCCTGGCTCACACAATGCACTCAAACTGGCCCGGCTGATCTGGACTTCT

AATCGCAACGTTATTCTCATGGCTCATGATGGCAAAGAACATCGATTTATGGTCTAG

>Common cuckoo ACAA2

ATGCAGTTCTTCTTCTTGTTCTTGCAAGCAACTATCATGACAGTGAGTTCAGAAAGTGTTTTCATAGTTG

CAGCGAAGCGAACGCCTTTTGGGACCTATGGGGGGCTGCTCAAGAACTTCACAGCCACCGATCTGACAGA

ACATGCTGCTCGAGCTGCCTTGGCTGCTGGCAAAGTGTCTCCTGAGATCATTGACAGTGTCATTGTCGGC

AATGTCATGCAGAGCTCTGCAGATGGGATTTATATTGCAAGACACGTTGGTTTACGCGTGGGAGTCCCTG

TCCCAGTTCCAGCCCTCACTGTCAACAGACTTTGTGGCTCGGGTTTCCAGTCCATTGCCAATGGATGTCA

GGAAATTTGCCTCAATGACTCTGAAGTTGTTCTGTGTGGTGGAGCTGAAAATATGAGTCAGGCTCCTTAT

GCAGTTCGAAACATTCGATTTGGAACCAGATTAGGAGCAGAACTGAAGTTGGAAGACACACTGTGGGAAG

GTCTAACAGATACGCATGTTAAAATTCCTATGGCAGTCACAGCTGAGAATCTGGCTGCAAAATACAACAT

CACGCGAGAGGACTGTGACCGATACGCATTCAAAACGCAGCAGAGATGCAAAGCTGCTCAGGATGCTGGT

TACTTTAATGCTGAGATGGCACCAATTGAAGTGAAAACGAAAAAGGGGAAGGAAAATATGCAAAAGGATG

AGCACCCAAAGCCCCAGACCACTCCGGAACAACTGGCGAAACTCCCATGTGTCTTTAAAAAGGATGGAAC

AGTCACTGCTGGGAATGCTTCAGGGGTGTGTGATGGAGCTGGTGCAGTCATTATTGCCAGTGAATCAGCG

CTTAAAAAGCACAGCCTTACACCTCTGGCAAGAGTAGTAGCCTATCACTCAGCTGGCTGCGACCCTTCCA

TAATGGGCATCGGCCCTGTACCTGCAATTACTGAGGTTCTGAAGAAAGCAGGATTGACCCTGAAGGACAT

GGATCTGATAGAGGTGAATGAGGCGTTTGCACCTCAGTATCTGGCTGTTGAAAAAGTTTTGGGCCTTGAC

CCTGAAAAAACCAACGTCAACGGAGGTGCCATCGCTATAGGTCATCCCTTGGGGGCTTCAGGGTCACGGA

TCACAGCTCATCTGGTTCATGAATTAAGGCGTCGTGGCGGGAAATATGCCGTTGGATCAGCTTGCATCGG

TGGTGGACAAGGTATTGCTCTTATCATTGAGAACACAGCCTGA

>Common cuckoo C18ORF32

ATGGTGTGCATTCCTTGTATTGTCATTCCGGTCCTCCTCTGGGTCTATAAGAAATTCCTTGAACCATATA

TCTACCCCATCATCGCACCATTCATTAAGCGCGTGTGGCCCAAGAGAGCGGTGGAAGAAACGACAAACAC

AAAACAAGGTCAAGGAGGCAGCGCTGGAAATCCACAGGCAGCTTCAGCCATGAAAAGAGATCAAGAGGAT

GAGCCTGGAATTTGTAAATTTGAAAGCAACAGCCTTGCAAATGGAATTGCTGCAAAGAGATCCACGGAGG

TGTCTGACAAAAAAGCAGATTGA

>Common cuckoo C18ORF54

ATGNNNGTCTACAGCTCCGCTTCCGAAGCTTTGGACGCTTCCATGGAGGACTTCCATCTGAGTCGCACCT

CTTTGGACAGAAGATCCGGAAGAATC

>Common cuckoo CFAP53

ATGTGTTCGAGAACCAATCTGTCTAAAGGACAAGGGCTTGAGAGGTCTCTCTTGGCCAGCAGACAGAAAG

CCGACGAACTTCAAAAATACACTGATTTCCTAAAGCTCTACAGCCACTATCACAGCGTCCATGAGTGGCA

GCATCGCAGTGATCAGAAATGGCTGCACAACACGGCACAGCGAAGGGTGGATGCAACAATGCAGGAACTC

TTGGCAGGGACTGATGAGAGACGAAAAAGGCTCCGTGAGCTTCTGGAGGCTGAGGAGAGTAGGTACTTCA

CTGAGATGGCATCACTTGAAGAAACAGTGCTGGAGAAACAAGAGAAGATGAGGATACGAGCGAAACTGCT

GAAAGAGAAGAGAGAAAGGGAAAGACAACAACTGGCAGCTGAGAAACGAGAGCAGCAATTCAGGGAACAA

TGCGAGGAGCTTCGTATACAGTTGATGAAACAGCATCAGATGGAATTGTGTGAAGATCGGCTGGCCCAGC

TAGCTCTGAAGGAGGAACTGAAAAAGCAACAGAAGAAGGAGGAGCAGATGTTTGCAGAGCTCTGGAAAGA

GGATAGGCTGGCAAAGGAAAAGCGGGAGTTGGCGGCCATGCAGAAATTCACAGAACAGAACCGGGAAATC

CTGAATACACTCAGTTCTCAGGTAGCGATGCTGAATGCTCACAAAGAAGAGGCAAAACGCTTGAAGGAGG

AAGAGGCTCGATTGTTGGAAGAACAGAAGCAACTGCTTAAGCTGGAACATGAACAACTTAAGATGGAGAA

ACGACAGAAGCAGAAGGAATGTAGGGATATGTTGCTTAGTGCAGCACAGGAAAAACAGAAGCGTCTTAAT

GAGGAAAAACAAGGTGAACTTGCCCTGGAGATGAAGATATTAGAAAAATGCCTTCAGGAACCCCAGGAAT

GCACCAAGGAGAAAACAAAAAGAAAACAAGAGCTGATGAAGGAACAACAGACTTACCTGGCATACCTGGC

ACAGCAGCTGGAGGAGGAGAAACAGCGAGAAAAAGAAGTGGACAAGGTCTTCAGTGAAGACATTGAGAAG

GTTTGGGCCAAGAAGGCTGAGCAAATGCGATTAGAAGAGGAGGGCAGAAGGCTTCTAATGAAAGATGTCC

TGACTACAAGACAAATGCAGATTGAGGAGAAGTTGAAGAGAAATGCAAAGGAGCAGGAAGAACTCGCTCA

AGAGAGGAAGTTATTAGAGGAGGCAGCCACAGAATTAGAACTTGTGGAAGAAGAGAGATTTTCCAGAAAA

GCAAAGGAAGGAAGAGAATACCAAGAGCAACTCAGGGCTCAAATTGCTGATCAACAGCAGGCCCGCGAGG

CTGAGGAAGAAGAGAAGCAGCAAGAACATGAATCACGTCTAGCAGAAGAGAGAGCATACCAAGAGAGGGT

ACGGGACATTCTATCAAGGCCTTGTGAAAAATTAGCAAAGACGCATCCTTTGAGAAGAAAACTAATGTGT

AACTTTCAAGATCATTTACAATGA

>Common cuckoo CTIF

ATGGAGAACTCATCGGTGGCATCAGCCTCCTCGGAGGCAGGGAGCAGCCGCTCCCAGGAGATTGAGGAGC

TGGAGCGTTTCATCGACAGCTATGTCCTGGAGTACCAGGTCCAGGGGCTGCTGACGGATAAAACAGAGGG

GGATGGTGAGAGTGAGAAGACACAGTCCCACATCTCACAGTGGACGGCAGACTGTAGCGAGCAGCTTGAT

GGCAGCTGTTCCCCATCCCAAGGGAAGGGCTCACCACCCCAGAAGAATGGGAACAAGGAGAGCTCCCTTG

ACATGCTGGGCACAGACATCTGGGCTGCCAACACCTTTGACTCCTTCAGTGGTGCGACGTGGGACTTGCA

GCCTGAAAAACTAGATTTCACCCAATTTCACAGGAAGCTGCGAAACACCTCCAAACACCCGTTGCCTCAC

ATAGACAGAGAAGGGCTTGGAAAAGGGAAATATGAGGATGGTGACAGCATCAACTTGAACGACATAGAGA

AGGTCCTTCCAGTGTGGCAGGGTTACCATCAGTTGCCTCATGAAGCTGAAATCGCACACACCAAAAAACT

GTTCAGAAGGAGGAGAAACGACCGGAGGCGACAGCAGAGACTTCCCAGTGGGAACAAGTCTCAGCAGCAC

ACAGATCATCAGCAGGGTGGCACCAAACACAACAGGGACCACCAGAAACTCTACCAGGGAGGCCAAGCCC

CTCACTCCTCAGGCAGGACGGGCCACCATGGCTACAGCCAGAACCGGAGATGGCACCACAATCAGAAGCA

CTCACCCAACGACAAAGAAACGCACAGAAATGCCAAAGAGACTGAGAATTTGAAAATTGAGGACAACTCA

GTCTGCACAGTGCATATTCCTGCAGAAACACATCGGAGCCCAGAGGCTGTGGAGAAGCAGTCTCAGCAGT

ACATACAGGAGTCAGAGACCAAGCGGAAAGACAGTATTCACGAGCGCATTGGGGAAAGACCCAAGATCAA

TTTGCTCCAGTCTTCCAAGGACAGGCTGCGGAGGAGACTAAAAGAAAAGGACGAAGTCACAGTGGAAAAC

ACTAATCCTGAAAAGAACAAAATGGACAAATTAATTGAAATCCTCAATAGCATGAGGAACAACAGCAGCG

ATGTTGACTCCAAGCTCACCACCTTCATGGAGGAAGCCCAGAACTCTACCAACTCTGAGGAGATGCTGGG

GGAGATAGTCAAGACCATCTACCAGAAAGCAGTGACAGATCGCAGCTTTGCTTCCACAGCAGCCAAGCTC

TGTGACAAAATGGCTCTCTTCATGGTGGAAGGAACCAAATTCCGAAGTCTGCTCCTCAACATGTTGCAGA

AGGATTTCACCATGCGGGAGGAGTTGCAGCAGCGGGACGTGGAGCGCTGGTTGGGCTTCATCACCTTTCT

CTGCGAAGTCTTCGGCACCATGAGGAGCAGCACCGGAGAGCCCTTTCGAGTCCTTGTTTGCCCCATCTAT

ACCTGCCTCAGGGAGTTGTTGCAATCTCAGGATGTGAAAGAAGACGCTGTGCTTTGCTGCTCCATGGAGC

TGCAGAGCACCGGCCGGCTGCTGGAGGAGCAGCTGCCTGAGATGATGACGGAGCTGTTGGCGATAGCACG

CGACAAGATGCTGTGTCCCTCCGAGTCCATGCTGACGCGCTCCCTGCTGCTGGAGGTCATCGAGCTGCAT

GCCAACAACTGGAACCCGCTGACGCCCACCATCACGCAGTACTACAACAAGACCATCCAAAAACTGACGG

CTTGA

>Common cuckoo DCC

ATGNNNCTTTCAGGGTCACAGATGGGGGCTTTTACGTCGCTGAGGTTCTCGGTGGAACCTTCGGACGCCG

TCACGGTGCGCGGAAGCGATGTCCTCCTCGAATGCCGAGCGGAATCCCATCCGGCCAATCCGGTGACGGT

GAAATGGAAGAAAGATGGCGTCTTCTTCAACCTGGCGGTAGACGAAAGGAGGAGACAATTCCCTAACGGA

TCTCTCCTGATCCAAAACGTGGTCCATTCCCGGCACCACAAACCCGATGAAGGGCTCTACCAGTGCGAAG

CATCCCTGGAAGGCATCGGAGCCATCGTCAGCCGCGCGGCCAAGGTGACGGTCGCAGGACCATTGCGGTT

TCTTTCCCAGACGGAATCTCTCACGGCTTTCATCGGAGACACCGTCCTCTTGAAGTGTGACGTCATCGGA

GAACCTTCCCCCGTAGTGCGATGGCAACGCAACCAAGAGGATTTCATTCCGAGCCTCGCGGATCCACGGA

TTGCCGTCTTACCTTCCGGAAGTCTCCAGATCAGTCGGATCCAAGATGGAGACAGCGGGATCTACAGGTG

CCTGGCGAAAAATCCCGCCAGCTCCAAAACCGGGAATGATGCCGAAGTCCGAGTTTTAGCAGATCCAGGA

TTCCAGAGGCAACAGTTTTTCCTGCGGCGCCCATCGAACGTGGTGGCCATGGAAGGGACGGATGCCGTTC

TGGAATGTTGCGTTTCCGGATCTCCCTCTCCCACCTTCACGTGGCTCCGAGGAGATGAAGTTCTTCCCAT

CAGGTCGGGAAAATATTCCTTATTGGCCGGAAGTAACCTCCTCATCTCCAACGTGACCTCCGATGACTCC

GGGACCTACACCTGCGCGGTCACCTCCAAAAGCGAGAACAGCAGCGGCTCAGCCGAGCTGACGGTGATGG

TCCCACCGTGGTTTTTAATCCGCCCCTCCAACCTTTACGCCTACGAGAGGACGGATATCGAGTTGGAATG

TTCGGTATCCGGGAAACCGGTGCCGACGGTGGAGTGGATCAAGAACGGAGAAGTGGTGATTCCCAGCGAT

TATTTTCAGATCGTGGGCGGCGGTAACTTACGGATTTTGGGAGTGGTGAAGTCGGACGAAGGGTTTTATC

AGTGCGTAGCGGAAAACGAAGCGGGAAACGCTCAGGCCAGCGCGCAGCTCATCGTCCCCCAACCAGTTAT

CCCAAGCCCTGGTGTCCTCCCCTCACCTCCTCGAGATGTTGTCCCCGTTTTGGTCTCCAGTCGCTTTGTC

CGTCTTTCCTGGCGACCACCGCTTGAGGTCCACGGCCACATCCAAACCTACACCGTCTTCTTCTCCAAAG

ATGGCATCAACCGGGAACGCGCCCTCAACACCTCTCAAAGTGGACCACTTCAGGTGACGGTGGGCAACCT

CAAACCTGAGGAGACCTACACCTTCCGCGTGGTGGCCTTCAACGAATGGGGTCCAGGAGAGACCTCTCAA

CCCATCAAAGTGGTCACTCAACCCGAACTTCAAGTTCCGGGACCTGTGGAGAACTTCCGAGTGGTTTCCA

CCACTCCGACGTCTCTTTTGGTTTCTTGGGATCCACCTTCTTCCCTCCATTCACCCATCGAAGGTTATCG

GATCTTCTGGATGGAAACCACCACCGGAAGAGAACAGACCGTGGAGGTGGACGGCTTCTCCTATCGCTTG

GAAGGGCTGAAGAAGTTCACCGAGTACACCTTGAGGTCCATCGCCTACAACCGCTACGGTCCTGGGGTCT

CCTCAGAAGATGTCACCGTCACCACTTTATCCGACGTTCCCAGCGCGACGCCTCAGAACGTCTCTTTGGA

GGTGGTCAACTCCAGGAGCATTAAGGTCTCGTGGTTACCACCACCTCCGGGAACTCAGAATGGGTTCCTC

ACGGGTTATAAAATCCGTCATCGCAGAACCTCACGGAGAGGAGAAATGGAGACGTTGGAACCCAACAACC

TCTGGTACTTATTCACAGGTCTGGAGAAAGGAAGTCAATACAGTTTCCAAGTGGCCGCGATGACCATCAA

CGGCACCGGACCTTCTTCCGAGTGGATCACCACCGAAACGCCGGAGAACGATCTCGACGAATCCCAAGTC

CCCGACCAGCCGAGTTCCCTCCACGTCCGCCCGTTGTCCACCAGCATCGTCATGACGTGGACCCCACCGC

TCAACCCCACCGTGGTGGTCCGCGGGTACATCATCGGTTACGGCGTGGGAAGTCCTTACGCCGAGACCAT

CCGCGTGGACCACAAGCAGAGGTTCTACTCCATCGAGAAGTTGGAGTCCAGTTCCCACTATGTCATCTCC

TTAAAGGCCTTCAACAACGCCGGCGAAGGAGTTCCTCTCTACGAAAGTGCCACCACGAGGTCCATCACAG

ATCCCGTTGACCCTTTCGAAGTTGACTTTTCTCCTCTTCTCGATGGTTTCCCCACCGCCGTCCCAGAGAG

CTCCACCCCCATGCTCCCACCAGTAGGTGTCCAAGCGGTTGTCCTCACCCACGACGCCGTCCGTGTCCTT

TGGGCGGAGAATTCCATCGCCAAAAGCCCAAAGACCACCGAAATCCGTTTCTACACCGTCCGCTGGAGAA

TGAGTTACTCCACCGCCGCCAAATATAAGTCCGCAGATACCACCGCTTTGAGTCATACGGTGATGGGGTT

GAAGCCCAACACGATGTACGAGTTCTCCGTGATGGTCACCAAGGGGAGAAGATCCAGCACGTGGAGTATG

ACGGCCCACGCCACCACCTACGAAGCAGCTCCGTCTTCTCCTCCGAAGGATCTGACGGTCATGGGTCGAG

AAGGAAAGTCCCGAACGGTGACGGTCAGTTGGGAACCACCTCTGGAGGCCAACGGGAAGATCACCGCTTA

TGTCCTCTTCTACACTTTGGAGAAGAACGCTCCCATCGATGAGTGGTTTATGGAGTCCATCAACGGCGAT

CGTCTCACCCATCAGGTGGTGGACCTCCATTTGGACACCATCTACTACTTCCGTATCCAAGCTCGGAATT

CCAAAGGCGTTGGACCTCTTTCCGATCCCATTTTCTTCCGGACTTTGAAAGTGGACCATCCGGATAAAAT

GGCCAACGATCAAGGTCGTCACGGAGACGGAACCTTTTGGCCGGTGGACACCAATCTCCTCGACCGGAGC

AGTTTGAACGAGCCACCCATCGGGCAGATGCATCCGCCCCACGGCAGCGTCACTCCTCAGAAGAGCAATA

ACCTCTTGGTGGTGGTGGTGGTGACCGTTGGGGTCCTCACGGTGGTGGCGGTGGGGGCGCTGGTGGCGGC

GGTGGTCATCTGCAACAGAAGGTCTTCGGCGCAACAGAGGAAGAACCGGAGCAGTCACAGCGTTGGGAAA

CGCAAAGGGAGTCAAAAGGATCTGAGACCTCCGGATCTTTGGATCCACCACGAGGAGATGGAGCTGAAGA

ACATGGAGAAAGGTTCCAGTACGGAAGGGACCACCGGAGAGTCTCCGAGACAGAGCTGTCAGGACCTCAC

GGCCGTCGGTCACAGCCAATCGGAGACCCAACTGGGCCCCAAGAGCGCTCCTCAACCCGGTCCCGAAGGA

GAAGACGTCGGGGGGACAATGTCCACCTTAGAGAGGTCCTTGGCCGCTCGTAGGGCCACTCGGGCCAAAC

TGATGATCCCTATGGAGACCCAACCAAGCGCCAACCCTGGGGTCGTCAGTGCTATTCCGGTTCCGACGTT

GGAAAGCGCTCAATATCCCGGAATTCTTCCGTCTCCGACGTGCGGTTATCCTCACCCTCAGTTTACTCTT

CGGGCGGTGCCTTTCCCGACGCTGGCGGTGGAGAGGACCTTCGGAGGAGGAAGAAGTGGCAACGAAGGAG

CATCGAGTCACCAAAGCGCGTTGGTCCCTCAAAGCCAAGCGGACCATCCCAGCGGGGAGGACGCGCCCAG

CAGGACCATCCCCACCGCCTGTGTCCGCCCCACCCACCCCTTGAGGAGCTTCGCCAACCCCTTGTTACCT

CCACCCATGAGTGCCATTGAACCCAAAGTCCCCTACACACCTCTGCTCAGCCAACCAGGTTCCTCTCTTC

CCAAGGCGCCCCTGAAGACGGCGTCTCTGGGTTTGCCCAAATGCCGATCTCCTCTCCTCCCGGTCTCCGT

TCCCACGGCTCCTCCGGAAGCCCCAGAAGAAGGCCACAAACCCACCGACGACACCACCAACGTCTACGAA

CAGGATGACCTCAGCGAGCAGATGGCCAGCTTGGAAGGGTTGATGAAGCAACTCAACGCCATCACGGGGT

CGGCCTTCTGA

>Common cuckoo DYM

ATGGGAGCAAATAGCAGCAGCATCAGCGAGCTTCCAGAAAATGAGTACTTAAAGAAATTATCAGGAGCAG

AGCCCATCTCTGAGAATGACCCGTTCTGGAATCAGCTGCTGTCCTTCAGCTTTACCACCCCAACGAACAG

TGCTGACTTAAAGCTCTTGGAAGAAGCCACGGTCTCAGTGTGCAGGTCCTTAGTTGAGAAGAATCCTCGT

ACAGGAAACCTTGGGTCGTTGATTAAAGTCTTTCTGTCTAGAACCAAAGAGTTAAAAATTTCAGCAGAAT

GCCAGAATCACATCTTCATCTGGCAGGCCCACAATGCACTGTTTATTATTTGCTGTTTGCTGAAAGTGTT

CATCAGTCAAATGACAGAAGAGGAGCTCCAGCTTCATTTTACTTATGAAGAGAAAGCACCAGGCTCGTAT

GGAACAGAGTGTGAAGACCTCATAGAAGAGTTGCTGTGTTGCCTCATCCAGCTCATTGTTGAAATTCCCC

TATTAGATATTACATACAGCATCTCCCTAGAAGCTGTGACAACTCTCATAGTCTTCCTCTCCTGCCAATT

ATTCCACAAAGAAATTCTACGAGAGAGCGTCATTCACCGATACCTGATGCAAGGTCGATGCCTCCCGTAC

ACCAGCAGGCTTGTGAAAACTTTACTGTATAATTTCATCAGGCAAGAGAGGAGCCCTCCTCCAGGGACCC

ACGTTTTCCAGCAGCAGACGGATGGAGGAGGATTGCTTTATGGAATTGCATCCGGAGTGGCAACTGGCCT

GTGGACGGTCCTCACGCTGGGTGGGGTGGGGAGTAAACCCACGCCACAGCTGGAGCAGTGCTCGCCACTC

GCTAACCAGAGCCTGCTGCTTCTGCTTGTCTTGGCTAATCTGACTGATGCTCCAGATACACCAAATCCCT

ACAGGCAAGCTATTATGTCCTTCAAGAACACACAAGATAGCACTGCTATTTCATCATCAAATCCACACGC

TTTCCAGATTAATTTTAACAGTTTATACACAGCGTTGTGTGAGCAGCAGAAATCAGATCAAGCAACTCTT

CTTCTATACATGCTTCTGCATCAAAACTGCAACGTGCGGACGTATGTGTTGGCACGCACAGACATAGAAA

ATCTTGTTCTGCCAATTCTTGAAATCCTGTATCACGTTGAAGAAAGGAATTCACACCATGTTTACATGGC

TCTTATCATTTTGTTGATCCTTACGGAAGACGATGGCTTCAACCGATCCATTCATGAAGTGATATTGAAA

AATATCACTTGGTACGCTGAGCGTGTCTTAACAGAGATCTCGCTTGGGAGTCTCCTGATCCTAGTTGTGA

TAAGAACCATCCAGTACAACATGACACGGACAAGGGACAAATACCTTCATACAAATTGTCTGGCAGCCTT

GGCAAATATGTCAGCGCAGTTCCGCTCACTTCATCAGTATGCTGCTCAGAGGATCATCAGTTTGTTTTCT

TTGTTGTCTAAAAAACACAACAAAGTGCTGGAGCAAGCCACGCAGACCTTAAGAGGTTCCCTTGGATCAA

ATGAGGCACCACTTCCCGATTATGCGCAAGACCTGAATGTGATAGAGGAAGTGATTCGAATGATGTTGGA

GATCATCAACTCCTGCCTGACCAATTCCCTTCATCACAACCCAAACTTGGTGTACGCGCTGCTTTACAAG

CGGGATCTGTTTGAGCAGTTTCGAACTCACCCTTCCTTCCAGGACATAATGCAAAATATAGATCTGGTGA

TCAGCTTTTTTAGCTCTCGATTAGAGCAAGCTGGAGCTGAGCTGTCAGTGGAGCGAGTTCTGGAAATCAT

CAAGCAAGGAGCTGTTGCTTTGCCCAAAGACAGGCTAAGAAAGTTCCCTGAGCTGAAGTTCAAGTATGTG

GAGGAGGAACAGCCTGAAGAGTTCTTCATCCCCTACGTCTGGTCCTTGGTCTACAACTCCGCTGTGGCCC

TGTACTGGAACCCGCATGACATCCAGCTCTTCACTATGGACTCCGGCTGA

>Common cuckoo DYNAP

ATGGACAACCAAACCTTGGAGATGGATGGAGAAGACCTTGCGCGGCCTTCGAGGGACAAAGAGGGGCCAA

AAAAGGAGGTAGAGGTCCAGAAGATGAAATGGTCCCTCCTGAAGGTCTTCTTGATGTGTCTTCTGGCGTG

TGTGGTCACCACAACTCTCGGAGTCCTCATCCTCTCCTTGGTCTACTTGAAGAATCCCCTTTATCTCCAA

GTGGTGGACCTCAAAACTGAAGGTCCACCTTCTCCCAAACCAGAAGAAAACAACGTGGACATCAAATTCC

AGTTCCTCAATCATCTCCCGACTTCAAAGGTCCACCGTTATCCCGGTGGAGAGATCCAATGGGGGAGGTT

CCGAGAGGACCTTGGACAATACGATAGTCCTCAAGAGATGACTTTTGGCCAAAGTCTCAACCGCCATCGC

TCGAAGATGACTTTCGCAACGTTGAAGATCAAATCTCGAGGCCTTCGAGTCCCTCATTGGCATTTCAACG

CCAACGAACACGGATACGTCCTCCAGGGCTCGGTCTGGGTTGGAGTCATCTCCTCCGAGGACCCAACGGT

CACCACCTACACGGTCAGCGCTGGACAGGTCTTCTTCTTCCCCCGAAACACCGTCCATTGGTTGAAGAAC

GTTGGCCTTCAAGACTGTGTCCTTCTGCTCTTCTTCTCCACCCACCAAGAACTTCAGACCATGGACGTGG

ACGATGCCTTCTTCTCCACTCCAGAAGACATCGTGGCGAGAGCTTTCAAGCCTCGCGGTGGTGTCAACTT

CATCCGTACCTTCAAGAAACGACTTGAAGACCAAGCGGTCAATTTACCGCCCAACTTAGACCTTCTCCTT

CACAACGTCACCTACGGACAATCTCCGGCCGAGAAGGTCTGGAGATACTTCTATGATCTTCAAGGGTCGG

CCACGTTTCCTTTCCCCGGAGGCCTCTTCCAGTGGGCTCCTTACCAGAGAGACACCACCACCTTCACCCA

CCTGGAGAAGATCTTCAGCGACTCCTTGAACACGCATGAAGACACTCTGACCTTGGCCACTCTCCGCATC

TTCAGCAACTCTTTGGGTCAACCTCATTACCACTTCAACGCTAACGAGATGGGTTACGTCGTCAGCGGCT

GCGGAGAG

>Common cuckoo ELAC1

ATGGCGATGGATATAACTTTCCTCGGCACAGGCTCAGCGTATCCCTCTCCAACACGAGGAGCATCGGCGT

TAGTGCTTCGCCGGGAAGGAGAGTGCTGGCTCTTCGACTGTGGAGAGGGAACTCAAACACAGTTCATGAA

GAGCCACCTCAAAGCAGGCAGAATTACCAAGATTTTCATAACTCATCTTCATGGTGACCACTTTTTTGGA

CTTCCTGGCCTTCTGTGTACCATTAGCCTGCAAAGTAACCCTGACACGAACAAATCACCTGTTGAAATTT

ACGGACCATTGGGACTGCGAGACTTCGTATGGAGGAGTCTGGAGCTTTCCNNNNNGCTGGTACCCACTCG

GGACCAGTGCCCCACAGAAGAATTCAAAGATTTTTCTTACTTGGAGCGAGATGAGGTGTCTCCGCAGGGA

GCACAGGGGAGAATACTCCGCCTGGATCCCATAGAAAACTCTTATTTGCTGTTTGAGGATGAGCAGCTGG

TTCTGAAAGCATTTCGCCTATTTCACCGCATTCCCTCCTTTGGCTTTGTGGTGGAAGAGAAGCCTCGGAC

CGGTAAACTCAATGTGCAGAAACTGAAAGAACTTGGAGTTCAACCAGGTCCTTTATACGGGAAACTGAAG

AGCGGAACTACAGTTGTTCTAGAAAATGGAGTAATGATTTCTCCTTCAGACGTCTTAGAAGACCCTATTC

CTGGAAGAAAAATCTGCATTTTGGGGGATTGTTCAGGGGTGGTTGGAGATGCAGCCGTGAAGCTTTGCTG

CGAAGCAGATGTACTGATACATGAAGCCACACTGGATGATACCCAGGAGGAAAAGGCCAGAGAGCATGGT

CATAGCACTCCAAAAATGGCATCGGATTTTGTGAAGTTGTGTAAAGTTAAGAGACTGGTTTTGACTCACT

TCAGCCAACGCTATAAACCAGCTGCTCAGAGAGGCGAGGGGGATACAGACATCACCGAACTGAAGAGACA

AGCAGAGTTGGCGTTAGATGGTCAAGAAGTAACACTAGCTGAGGACTTTCTGACAATAGAAATTCCAATG

AAAAAGTGA

>Common cuckoo FECH

ATGAGAAACGTGGTGTGGAATAACCAGCAGCGGTGGGGGGACCGGCTGGAACTGCCCGGAGTGAGGCCAG

TAGTGAGGATCGGCAGCCAGCTGAGGGTCCCGGTGCGATGGACAGCTCAGGCGACAGCAGCCGCAGTGAC

AGAGAGCACGACACCTCAGATCCAGCCAGAAGCACGGAAACCTAAAACAGGAATCTTGATGTTGAACATG

GGTGGTCCTGAACGGCTGGATGATGTGCACGACTTTTTACTTCGTCTCTTCCTGGACAGAGATCTCATGA

CACTTCCAGCTCAAAACAAATTAGGCCCATTCATCGCCAAACGCCGCACACCAAAAATCCAGGAGCAGTA

CAGCCGGATTGGGGGCGGCTCGCCCATCAAGAAGTGGACAGCGGTGCAGGGAGAAGGCATGGTGAAGCTG

CTGGACAGCATGTCTCCTCACACCGCGCCTCACAAATACTACATTGGCTTCCGGTATGTCCACCCTCTGA

CGGAAGAAGCCATCGAGGAGATGGAGAATGACGGCATCAGAAGGGCTATCGCCTTCACACAGTACCCGCA

GTACAGCTGCTCCACCACAGGAAGCAGCTTGAATGCCATTTATCGCTACTATAATCAAAAAGGGGAGAAG

CCAAAGATGAAGTGGAGTATAATTGACCGATGGCCCACACATCCCCTCCTTATTCAGTGCTTCGCCGATC

ACATCCAGAAGGAGCTGAACCTCTTTCCACCCGACAAAAGGAAAGATGTCGTCATCCTCTTCTCGGCTCA

CTCGCTGCCCATGTCTGTAGTGAACCGTGGCGATCCGTACCCTCAAGAAGTGGGAGCGACGGTCCAGAAA

GTCATGGAGAAGCTGAACTACTCCAACCCGTACCGGCTGGTGTGGCAGTCCAAGGTTGGACCGATGCCCT

GGCTCGGACCCCAGACGGACGAGGCCATCAAAGGGCTGTGCCAGAGAGGGAAGAAGAACATGTTGTTGGT

CCCGATAGCATTTACGAGTGACCACATCGAAACCCTTTATGAACTGGATATTGAGTACGCCCAGGTTTTA

GCAAATGAGTGCGGCGTTGAGAATATCCGACGAGCGGAGTCTCTGAATGGAAACCCACTGTTTTCCAAGG

CTCTGGCAGACTTGGTCTGCTCCCACATCCAGTCCAACGAGATCTGCTCGCGGCAGTTAACCCTCTGCTG

CCCACTCTGCGTCAATCCCGTCTGCAGGGAGACCAAAGCCTTCTTCACGAACCAGCAGCTGTGA

>Common cuckoo LIPG

ATGGGGCTGCAGAAGGTGATGGTCTGTGTGCTAGGCGATGCCCTTTGGGAATTCTCTTGTGCCCTGTGGG

TCCAGAGCATCCTGATTGGCAAAGAGCTGGGGGATCCCACAGATGCCACCGAGCTGCTGGAGGACAAACG

GGAGCGTGCACCGGCTCCGAAGAAGCAGGTGAAGTTCAATGTCCGCTCCTCGCTGGACACCGAAGAGGAT

GGTTGTGCGCTCATCGTCGGGCAGGACAAGTGTCTGGAGGATTGCAAGTTCAACCTGACGGCCAAGACCT

TCTTCATCATCCATGGCTGGACGATGAGTGGCATGTTCGAAACCTGGCTGGGCAGCTTGGTATCCGCTCT

TCAGGAGAGGGAGAAGGATGCCAACGTGGTCGTGGTGGATTGGCTGTCGCTTGCCCACCAGCTCTATACT

GATGCTGTGAACAACACACAGGTTGTTGGAAAAAGCATAGCAAGGCTGCTTGACTGGTTACAGGTAATGA

AAACNCCTCTCTTCCAGCTTGAGAACGTCCACCTGATTGGGTACAGCCTGGGCGCTCACGTTGCTGGCTT

TGCTGGTAACCATGTCCATGGGACAATAGGCAGAATTACAGGCTTGGATCCAGCTGGGCCAATGTTTGAA

GGAGTGGACCCTAGCAAGCGCCTCTCCCCAGATGATGCTAACTTTGTGGATGTCCTTCACACCTACACAA

GGGAAACACTAGGTGTTAGTATCGGGATCCAGATGCCTGTGGGACATCTTGACATCTACCCCAATGGGGG

AGACTTCCAGCCTGGCTGTGGATTAAGTGATGTCTTGGGAGCAATTGCTTATGGAACAATAGGTGAAGTT

GTTAAATGTGAACATGAGCGGTCTGTGCACCTCTTTGTGGACTCTCTTGTGAACCAAGATAAACAGAGCT

TCGCGTTCCAGTGTACCGATTCCAGTCGCTTCAAGAAGGGAATCTGCCTAAGCTGCCGGAAGAACCGCTG

CAATGGCATTGGCTACAATGCCAGGAAGACAAGGAGCAAAAGGAACAGCAAAATGTACTTAAAAACAAGA

GCTGACATGCCATTCAAAGTCTACCATTATCAGATGAAAATGCACGTCTTCAGCTACAATAGCCTGGGGG

AGGCTGATCCCACTTTCTCTGTCACCCTTCATGGCACCAATGGAGACTCTGAACCTCTCTCTTTAGAAAT

GCTTGATCAAATTGGCCTAAATGCCACCAACACCTTCCTGGTCTATACTGAGGAGGACATGGGTGAACTT

CTAAAAATAAAGCTCACCTGGGAGGGAACATCTCAGTCATGGTATGACCTGTGGAAAGAGCTGAAGAGCT

ACTGGTATCGGCCTGTGAAGTCCTCCCAGGAGCTGCATATCAGACGCATACGTGTGAAGTCTGGGGAAAC

ACAACAGAGGTTTGCATTTTGTGTGGAGGATTCCCAGCTGACCAGCATATCTCCTGGTAAAGAGCTCTGG

TTTGTTAAGTGCCCAGAGGAATGGCAAAAAAGATGCTAA

>Common cuckoo MAPK4

ATGGCAGAGAAGTGCGACTGCATCGCCAGCATGTATGGGTACGACCTGGGCTGTCGCTTCATTAATTTTC

GCCCCTTGGGCTTTGGGGCCAATGGGTTGGTGCTGTCAGCCCTCGACAGCAAGAGCTGCCGCAAAGTGGC

AGTGAAGAAGATCACCATCAGCGATGCAAGGAGCATGAAGCACGCTTTCCGGGAGATCAAAATCATCCGC

CGTCTGAACCACGATAACATTGTGAAGGTGTACGAGGTGTTGGGGCCAAAGGGGACCAACCTGCATGGGG

ATTTTTTCAAGTTTAACATGGTTTACATCGTCCAGGAGTACATGGAGACAGACCTGGCGCGGCTGCTGGA

GCAGGGGAAGCTCGCTGAGGAGCACGCCAAGCTCTTCATGTACCAGCTGCTGCGGGGGCTGAAGTACATC

CACTCAGCTAACGTCCTCCACCGTGACCTCAAGCCAGCCAATATTTTCATCAGCACGGAGGACCTGGTCC

TGAAGATTGGTGACTTTGGGCTGGCCAGAATCGTGGATCAGCATTACTCACACAAGGGTTACCTTTCTGA

AGGCTTGGTAACGAAGTGGTATCGCTCCCCTCGCCTCCTCCTCTCGCCAAACAACTACACCAAAGCCATC

GACATGTGGGCAGCTGGCTGCATCCTGGCAGAGATGCTGACGGGAAGGATGCTCTTTGCTGGGGGTCATG

AGCTGGAACAGATGCAGCTTATCCTGGAGACGATCCCTGTTATCCATGAAGAAGACAAAGAAGAGCTGCT

CAAAGTGATGCCCATGTTCATCAATAGCACCTGGGAAGTGAGGAAGCCACTGCGCAAGCTGCTCCCCGAA

GTGGACAGTGAAGCTATTGATTTCCTGGAGAAAATACTGACGTTTAACCCTATGGATCGATTGACGGCTG

AGATGGGTCTGCAGCACCCTTACATGAGTCCGTATTCCTGCCCAGAGGATGAGCCGGTGTCGCAGCACCC

ATTCCGGATTGAAGATGAGATTGATGATATCTTACTGATGGAAGCCAACCAGAGCCAGATGTCCAACTGG

GACAGGTATCACGTAAGCCTCTCCTCTGACTTGGAATGGAGACATGATAAATACCATGATATGGATGAGG

TTCAGCGCGACCCCCGGGCAGGGTCTGAATCCATCGCTGAAGAAGTACAAGTCGACCCACGGAAATACTC

GCAAAGCAGCTCAGAGAGGTTCTTGGAGCTATCCCACTCATCTATGGACCGAGTATTTGATGCCGATTGT

GGGAGATCGTGTGATTACAAAGTGGGATCACCTTCCTACTTGGACAAGTTATTGTGGAGAGACAATAAGC

CCCATCATTACTCAGAGCCCAAGCTGATTTTGGATTTATCCCACTGGAAAAGAGCAACCATAACACCTGC

AGCTGAGCTATCACTGGAAGAGGAACCATCTAACCTCTTTCTGGAGATTGCTCAGTGGGTGAAGAGCACG

CAGGTGGGTCTCGAGTGTCCCACTTCTCTTCCGGAGATTCAGGAACGGAGTCTGCCGGCTTCTCCTCACC

ATCTCCACCAAGAACCCACAGAGGTGAACAAAGAAACAGACCCTGAGTTTAACTTGGACGTCTTCATCTC

CAGAGCACTGAAACTTTGCACAATACCCGAGGATATGCCAGACAACAAGCTCAGTGACATCAATGGGGCC

TGCATATCTGAGCACCCCGGTGAGATTGTACAAACAGAGGTGTACCAGAAAGAACGATGGTGA

>Common cuckoo MBD2

ATGNNNAGCGGGAAGAAGTTCCGGAGCAAACCTCAGCTGGCGAGATACCTGGGCAACACGGTGGACCTCA

GCAGCTTCGACTTCCGGACGGGGAAGATGATGCCCAGTAAATTGCAGAAGAACAAACAAAGACTAAGGAG

CGAGTCCCTCAACCAAACCAAGGGCAAACCGGACCTCAACACCACGTTGCCCATCAGGCAGACGGCCTCC

ATCTTCAAGCAACCCGTCACCAAAGTCACCAACCACCCCAGCAACAAAGTCCGCACGGACCCGCAACGAG

TGACCGAACAGCCTCGGCAGCTCTTCTGGGAGAAGAGGCTCCAAGGACTGAGCGCCTCCGACGTCACTGA

GCAGATCATCAAGTCCATGGAGCTCCCCAAAGGTCTTCAAGGAGTTGGTCCGGGGAACAACGATGACACT

TTATTGTCCGCTGTCGCCAGTGCTTTACACACCAGTTCGGCGCCCATCACCGGACAACTCTCGGCGGCAG

TGGAGAAGAACCCGGCGGTTTGGCTCAATACGGCGCAGCCGCTCTGCAGGGCCTTCATCGTCACCGATGA

GGACATCAGAAAACAAGAAGAACGCGTTCAACAAGTCCGGAAGAAACTGGAAGAAGCACTTATGGCCGAC

ATATTGTCCCGAGCGGCCGATACCACCAGAGACCTCGAGGTGGACATGGACAACGGAGATGAAGCATGA

>Common cuckoo ME2

ATGTTCTCCCGACTCCGAGTAGCTGCCACTCCCTGTGTGATGGCATGTCGCAGCGCCCATACGAAAGAAA

AAGGAAAGCCACTGATGTTAAACCCACGAACAAACAAGGGCATGGCCTTTACATTACTTGAACGACAGAT

GCTTGGGTTGCAAGGACTTCTACCTCCTAAAATAGAGACACAGGACATTCAAGCGTTACGTTTCCATAAG

AATTTGGCAAAAATGACTGACCCTTTGGAAAAGTATATCTACATAATGGGAATCCAAGAGAGAAATGAAA

AGTTATTCTACAGAGTATTACAAGATGACATTGAGCGTTTAATGCCAATTGTATACACACCAACAGTGGG

CCTTGCCTGCTCCCAGTATGGACACATCTTCAGGAGACCAAAAGGATTATTTATTTCCATCTCAGACAGA

GGTCATATTAGGTCAATTGTGAACAACTGGCCAGAGAATGACGTTAAGGCTGTTGTTGTCACTGATGGAG

AAAGAATACTGGGTCTCGGAGACCTTGGTGTGTATGGGATGGGAATTCCAGTAGGAAAACTGTGTTTGTA

CACAGCCTGTGCAGGAATACATCCAGATAAATGCTTGCCTGTGTGCATCGACGTTGGAACTGATAATACA

ACACTCTTAAAAGATCCATTTTATATGGGCCTGTACCAAAAAAGAGATCGCTCGCAGCTCTATGATGACC

TAATTGATGAATTTATGGAGGCCATTACAGACAGGTATGGTCAGAACACACTCATCCAGTTTGAAGACTT

CGGAAACCACAACGCTTTTCGGTTTTTGAGAAAATACAGAGAGAAATACTGTACCTTCAATGATGATATT

CAAGGGACAGCTTCAGTGGCCTTGGCAGGACTGCTCGCAGCACAGAAAGCCACTGGTAAACCACTTGCAG

AGCAGAAAGTGCTGTTCCTTGGAGCTGGAGAGGCTGCCCTGGGAATTGCAAACCTCATTGTTATGGCTAT

GATGGAAAGTGGTGTTTCTGCTGAGGAAGCCTACAGGAGAATATGGATGTTTGACAAATATGGGTTACTG

GTTCAGGGGCGAGAACAAAATGTAGATTCCAATCAAGAACCATTTGCGCACCAGGTTCCAGAGCAGTTAC

CAAAGACGTTTGTAGAGGCAGTAAATGTACTTCGGCCTTCAGCTATCATTGGAGTTGCAGGAGCTGGGCG

GCTCTTCTCTCAGGATGTGATCAAAGCGATGGCCTCTATCAATGAGCGACCCATCATATTTGCACTGAGT

AACCCGACAGTGAAAGCTGAGTGCACAGCGGAGGAGGCGTATACACTAACAGAGGGCCGTTGCTTGTTTG

CCAGTGGCAGCCCCTTTGACCTGGTCACTCTGAAAGATGGAAGAACCTTCAAACCAGGCCAAGGAAACAA

CGCTTATATTTTTCCAGGCGTGGCTCTCGCTGTGATCCTCAGCAGTGTTCGACATATTAGCGATAAGGTT

TTCCTAGAGGCTGCTAAGGCATTGGCAGAACAATTGACCGATGAAGAACTTGCACAAGGAAGACTCTATC

CTCCGTTGTCTAATATCAGGGAAGTTTCTATTTATATTGCTGTCAAGGTTATGGAATTTTTGTATGCAAA

CAACATGGCTTTCCATTACCCTGAGCCTGCGGACAAGAACCGTTACATTCGATCGAAGGTTTGGACCTAC

GAATACGAATCCTTCATGCCAGATGTGTACGACTGGCCTGAATCTAAGGTTCACTGA

>Common cuckoo MEX3C

ATGAGGGGACAGTCAGTGTCCACAGGCTCCTCGGCTCCCAGCGCCGGGGCGGCCGAGCCCAAGTTGTGCG

CCCTGTACAAGGAGGCCGAGCTGAGGCTCAAAAGCAGCTCCAACACCACCGAGTGCGTCCCGGTGCCGAG

CTCCGAGCACGTGGCGGAGATCGTGGGGCGGCAAGGTTGCAAAATAAAAGCGCTGAGGGCCAAGACAAAT

ACTTACATTAAGACCCCTGTTCGTGGAGAAGAACCCATCTTTGTTGTCACTGGACGAAAAGAGGATGTAG

CCATGGCCAAAAGGGAAATTCTCTCAGCTGCTGAGCACTTCTCCATGATCAGAGCATCACGCAACAAGAA

CGGTCCTGCCCTGGGAGGCGTGCTGGGTACCCCCAACCTGCCAGGTCAGACCACGGTCCAAGTCCGGGTG

CCTTACCGCATGGTTGGACTCGTGGTGGGACCCAAAGGAGCTACCATCAAAAGGATCCAGCAGCAAACAC

ACACCTACATAGTCACTCCCAGCAGAGACAAGGAACCTGTCTTTGAGGTAACGGGGATGCCAGAAAACGT

CGACCGTGCGCGCGAGGAGATTGAGATGCACATAGCCATGCGTACGGGCAACTACATCGAGCTCAGCGAA

GAGAACGATTTCCATTACAACGGTACGGATGTCAGCTTTGAAGGAGGCGCTCTCGGATCTGCATGGCTTG

CTTCTAATCCTGTCCCTCCTAGCCGCACAAGGATAATTTCTAATTATAGAAACAACAGCTCCAGTTCTTT

GGGAAGTGGCTCCACAGATTCCTATTTTGGAAGCAATAGATTGGCTGACTTCAGCCCAACGAGTCCATTC

AGCACAGGCAACTTCTGGTTTGGAGAAACGCTGCCTACAGTGGGCACAGAAGACCTTGCGGTCGACTCTC

CCGTGTACGACTCCTTACCAACGCCTTCCCAAACCATTTGGACTCCTTTTGAACCCGTAAACCCTCTCTC

TGGCTTTGCTAGTGACACCACTACTAACGCCAAGCCTCTGTGCCGAGGGAGCCAGCCATCTACTCCTCGC

CTGTCGCCCACATTTCCTGAAAGTCTGGATCACCCGCTGGCTCGGAGAGTGAGAAGCGACCCACCTGGTA

TCTGCCACCAAGCTGGCCTTCCCATATACATCCCCGCTTTCTCCAATGGTACCAACAGTTATTCCTCTTC

CAACGGCGGCTCCACCTCCAGCTCGCCCCCCGAGTCGAGACGGAAGCACGACTGCGTGATCTGCTTTGAG

AGCGAAGTCATCGCGGCCCTGGTCCCCTGCGGCCACAACCTCTTCTGCATGGAGTGTGCCAACAAAATCT

GTGAGAAAGAAACACCATCATGTCCTGTTTGCCAGACAGCTGTTACTCAGGCAATCCAAATTCACTCTTA

A

>Common cuckoo MYO5B

ATGTCCACCGCGCAGCTCTACACCAAGCCGGAGATCTGCCGGCTACGGATTGATAACCAGTACGGAGAGA

GCTCTCTCCCTGTGGTCCGCGTCTTCCCAAGGCTACCCTACACTAGGGTTTGGATTCCTGACCCTGATGA

AGTTTGGAGATCGGCAGAAATTATCAAGGATTACAAAGAGGGAGATAGAAGCCTCCATCTGAAACTTGAA

GATGAAACTCTCTACGAGTATCCTATCGACCTCCAAGGAAACGAGCTGCCTTTCCTTCGCAATCCGGATA

TCCTGGTGGGAGAGAATGACCTGACTGCCCTGAGCTACCTGCACGAGCCTGCAGTACTCCACAACCTCAA

AGTCAGGTTCCTCGAGTCCAACCACATCTACACATACTGTGGTATTGTTCTTGTTGCCATCAATCCATAT

GAACAGCTGCCAATCTATGAACAAGATGTCATCTATGCGTACAGTGGCCAAAACATGGGGGATATGGACC

CTCACATCTTTGCAGTGGCAGAGGAAGCCTACAAGCAGATGGCCAGGGATGAGAAAAACCAGTCCATCAT

CGTGAGCGGGGAGTCAGGTGCTGGAAAGACAGTCTCTGCCAAATATGCCATGCGCTTCTTTGCAACCGTC

GGTGGTTCTGCCAGCGAGACTAATATCGAAGCCAAAGTCCTCGCGTCAAGCCCAATTATGGAGGCGATTG

GAAATGCTAAAACAACTAGGAATGACAACAGCAGTCGCTTTGGGAAATACATTCAGATCGGCTTTGATAA

AAGATACCATATCATTGGTGCCAACATGAGGACATATCTGTTGGAAAAGTCACGGGTTGTATTCCAGGCA

AAGGACGAGCGCAACTACCATATCTTCTATCAGCTTTGTGCCTCAGCGAGTCTTCCAGAATTCAAAGACC

TTGGACTAACATGTGCTGAAGACTTTTTCTACACTTCTCAGGGAGGTGACACATCTATCGATGGTGTGGA

TGATGCTGATGACTTTGAGAAAACCAGGCATGCCTTCACCCTGCTTGGAGTGAAGGAGTCTCATCAGATG

ACCATTTTTCGGATAATTGCTGCCATTCTGCACCTAGGGAACTTGGAAATCCAAGTGGAACGAGACGGTG

ATGCCTGTAGCATATCGAGCGAGGATGAGCACTTGAACCACTTCTGCAGCTTGCTGGGCGTGGAGCACAG

CCAGATGCAGCACTGGCTTTGCCATCGCAAGCTCATCACCACAGCTGAGACCTATATTGCCCTGGCCAAG

CACATCTACGCCCAGCTCTTCAACTGGATTGTGCACCACATCAACAAGGCCCTGCACACCACCGTCAAGC

AGCACTCCTTCATTGGCGTGCTTGATATCTACGGGTTTGAAACTTTTGAAGTGAATAGCTTTGAACAGTT

TTGTATCAACTATGCGAATGAAAAGCTTCAGCAGCAGTTCAACTCGCACGTGTTTAAGCTGGAACAAGAA

GAGTACATGAAGGAGGGAATCCCTTGGACTCTCATAGACTTCTACGATAACCAGCCCTGCATAGATCTTA

TAGAGGCGAAACTTGGTATCTTGGACCTACTGGATGAAGAGTGCAAGGTTCCCAAAGGCACCGACCAGAA

CTGGGCACAGAAGCTGTATGACCGGCACGGCAGCAGCCAGCACTTCCAGAAGCCCCGCATGTCCAACACC

TCCTTCATTGTCCTGCACTTCGCCGATAAGGTGGAATACCAGAGTGAGGGGTTTCTGGAGAAGAACAGGG

ACACCGTGTATGAAGAACAGATCAACATCCTGAAAGCCAGCAAGTATCAAATGGTAGCAGACTTATTCCA

AGATGAGAAGGATGCTGCACCCACCACTTCCATGGGAAAGGGAACATCCAAAATCAGCGTCCGTTCTGCC

AGACCAGTCATCAAAGCTGCCAATAAGGAGCACAAGAAAACAGTGGGACACCAGTTCCGCAACTCACTGC

ATTTGCTCATGGAGACTCTGAACGCCACCACCCCGCACTATGTGCGCTGCATCAAGCCAAACGACGAGAA

GCTCCCTTTTAAATTTGATCCAAAGAGAGCAGTGCAGCAGTTGAGAGCTTGTGGAGTGCTGGAGACCATC

CGCATCAGTGCAGCTGGCTTCCCGTCCAGATGGTCCTACCACGACTTTTTCAACAGGTATCGTGTTCTTA

TGAAAAAGAGAGACCTTTCTAAGAAGGACAAGAAGCAGATCTGTCAGACCCTGTTGGAAGACCTCATTAA

GGATCCAGACAAGTTCCAGTTTGGACGTACCAAGATCTTTTTCCGTGCAGGCCAGGTGGCATATCTGGAG

AAACTGCGAGCAGATAAGTTCAGAGCTGCCACAATCATGATTCAGAAGACAGTGCGGGGCTGGCTGCAGC

GGGTCAAGTACAAAAGGATGAGACAAGCTGCAGTCATCATCCAGCGCTACACACGTGGGCACCTGGCACG

GAGACTTGCTGAGCACCTGAGGAGGACAAGAGCTGCCATCATCTTCCAGAAGCAGTACCGAATGCTGCGG

ATCCTCCGAGCGTTCCAGAGAGTCCGCAACGCAACCATCACCATTCAGGCTTTTGCTCGGGGCATGTTTG

TCAGGAGGATTTATCACAAGATCCTTGCAGAGCACAAGGCCACCATCCTCCAGAAGTACGCACGTGGCTG

GCTGGCCCGCACTCACTTCCATCGGGTCAGGGGTGCCACCATCGTCCTGCAGTGCTACTACCGGCGCATG

AAGGCCAGGCAGCAGCTGAAGGCGCTGAAGATCGAGGCCCGCTCGGCACAGCACCTGAAGAAGCTCAACA

TTGGCATGGAGAACAAGGTTGTCCAGCTTCAAAGGAAGATTGATGAGCAGAACAAGGAATACAAACTTCT

GAATGAGCAGCTCTCTACTCTCATGTCTGCCCACTCCACCGAGGTGGAAAAGCTGAAGAAGGAACTGGTG

CAATATCAGCAGAGCCACCAGGGTGATGGCAACCAGCTTGTCAGCTTGCAAGAAGAGATGGAGCACCTCC

GGTTGGAGCTTGAAAAAGCTCATGGTGAGAGAAAGGTCATGGAAGACAGCTATGTTAAGGAGAAAGACCT

ACTGAGAAAGCGCATATCCGACTTGGAAGAAGAAAATGCCCTCCTGAAGCAGGAGAAAGAGGAGCTCAAC

AGCAGGATCCTGTGTCAGTCTGAAGATGAATTTGCGCGAAACACAGTTGAGGAAAATATCCAGATGAAGA

AAGAGCTGGAAGAAGAGAGGTCTCGTTATCAGAACCTGGTAAAAGAGTATTCGAGGCTGGAGCAAAGATA

TGACAATTTGCGGGATGAAATGACTATTATAAAGCAAGCACCAGGGCACAGGAGAAACCCATCCAACCAG

AGCAGTTTGGAGTCCGATTCCAATTATCCATCCATATCAACCTCTGAGATAGGAGACACTGAGGATGTAA

TACAACAAGTGGAGGAGGTTGGAATGGAGAAAGCTGCCATGGACATGACCCTCTTCCTAAAGCTGCAGAA

GAGAGTGAGGGAGCTCGAGCAGGAGAGGAGGAAGCTGCAAACCCAGCTGGAGAAAAAGGAGCAAGAAAGC

AAGAAATCCCAGGTAATTGAAATGAAGACTGAAGTGACTTCAGACCACGAAGATTTTGCGTACAACAGTC

TGAAGAGGCAAGAGCTGGAGTCAGAGAACAAGAAGCTGAAAAATGAACTTAATGAGCTGAGGAAGGCCAT

TGCAGACCGAGCAACCCAGAACAACTCATCCAACGATATTCAGGACAGTTATAATCTCCTACTGAATCAG

CTGAAATCGGCCAACGAGGAATTGGAAGTGCGTAAGGAAGAGGTGCTCATCTTGAGGACACAGATTATGA

ATGCAGCCCAGCAAAAACAGACTGGGAAAAACATGGAAAGCATCCCCACCAATGCCAGCTGGCCGAACAG

TGACAAGCACATTGATCAGGAGGACGCGATCGAAGCCTACCAGGGGATGTGCGAGACGAACCGCAAGACT

GAGGACTGGGGCTATCTCAATGAAGATGGAGAGCTCGGCTTGGCTTATCAAGGCTTAAAGCAAGTTGCCA

GGTTGCTGGAAGCACAGCTCCAGGATCAGAGAAGAGATCATGAAGAGGAGGTAGAAGCTCTGAGAAACGA

GGTGGATGCAATGAGAGAAGAGCTGGAGAAGCAGCAGCAGGCTTTCCTGCAGACCCTGCAGCTGTCTCCA

GAGGCACAGGTGGAGTTTGGACTTCAGCAAGAAATTACACGTCTCACCAATGAAAACCTGGATCTTAAAG

AATTGCTGGAGAAGTTGGAAAAGAATGAAAAGAAGCTGAAGAAGCAGCTCAAGATTTACATGAAGAGGGT

TCAAGATTTTGAAGCATCCCAAGCCATGGTACCAGCAGAGAGGAGGCAGCATGAGCGCAACATGCAAGTT

GCTGTCCAGAGAAAGGAGAAAGATTTTCAGGGCATGTTGGAATATTATAAAGAAGATGAGCCACTCCTCA

TCCGAAACCTCATAACGGATCTGAAGCCCCAGGCCGTGTCTGCTACTGTTCCCTGCCTTCCTGCCTACAT

CCTCTACATGTGCATCAGACATGCGGATTACATCAATGATGACCAGAAAGTGCACTCCTTGCTCACCTCC

ACCATCAACGGCATCAAGAAAGTGCTGAAGAAACACAACGATGATTTTGAGATGACGTCGTTTTGGCTGG

CAAATACATGTCGCCTTCTGCACTGTTTGAAGCAGTACAGCGGAGATGCGGGCTTCATGACACAAAACAC

ACCTAAGCAGAATGAGCACTGTCTGAAGAACTTTGACCTGACCGAGTACCGCCAGGTGCTGAGCCACCTC

TCCATCCAGATCTACCAGCAGCTCATAAAGATAGCAGAGGGCATACTCCAACCTATGATCGTGTCTGCCG

TGTTGGAAAATGAGAGTATACAAGGGCTTTCTGGTGTCAAACCAATGGGCTACAGGAATCGCTCCTCCAG

CATGGCAGATGGTGACAGTTCCTACAGCTTAGAAGCAATCATTCGCCAGCTGAACGTATTCCACGGCATC

ATGTGTGACCAGGGTCTGGACCCAGAGATCGTGCAGCAGGTCTTCAAGCAGCTCTTCTACATGATCAACG

CAGTTGCTCTGAACAACCTCTTGCTGAGGAAGGATGTTTGCTCGTGGAGCACTGGGATGCAGCTAAGGTT

TAACATAAGCCAGCTGGAGGAATGGCTGCGTGGGAAGAATCTGCAGCAGAGTGGAGCAGCAGAGACTTTG

GAGCCCTTGATTCAGGCGGCGCAGCTTCTGCAGCTGAAAAAGAAAACCTCAGAAGATGCCGAGGCCATCT

GCTCCTTGTGCACATCGCTCACAACGCAGCAGATTGTAAAGATACTTAATCTCTACACTCCTGTGAATGA

GTTTGAAGAACGTGTGACCGTAGCTTTCATACGAGACATACAGATGCACTTACAAGAGCGGAATGACCCA

CCGCAGCTGCTGTTAGACTTCAAGCACATGTTCCCGGTTTTGTTTCCTTTCAATCCATCCTCCATAACCA

TGGACTCGATTCATCTCCCTGCTTCTCTCAACTTGGAATTTCTCAATAAAGTCTGA

>Common cuckoo NARS

ATGGACAAGGAGCTTTATGTCTCCGATCGGGAAGGCAGTGACTCCACTGGTGATGGGACGCAGAAGAAAC

CCTTTAAGACTGTTCTGAAGGCTTTGATGACTGCGGGAAAGGAACCATTTCCTACCATTTACGTGGACTC

ACAAAAGGAAAATGAGAGATGGGCCATTATTTCCAAGTCGCAGATGAAGAATGTCAAGAAGCTGTGGCAC

CGGGAGCAAATGAAGAATGAAGCGAAGGAGAAGAAGGAGGCAGAAGATCTCTTGAGAAGAGAGAAGAACC

TGGAGGAAGCCAAGAAGATTGTCATCAAGAATGATCCCAGCCTTGCGGAGCCAAAATGCGTGAAGATCGA

AGCCCTGAAGGCTTACAGAGGCCAGCGCGTGAAGGTTTTTGGCTGGATTCACAGACTGCGGAGGCAAGGA

AAAAACTTGATGTTCATTGTTCTGAGAGATGGCACAGGTTTTCTTCAGTGCGTCCTGTCAGATGAACTGT

GTCAGTGTTACAACGGGCTGGTTCTCTCCACGGAGAGCAGCGTCGCAGTGTACGGAACGCTCAACCTGGT

TCCTGAAGGCAAGCAGGCTCCAGGAGGCCACGAGCTGAACTGCGATTACTGGGAGCTCATGGGCCTGGCC

CCGGCAGGGGGGGCTGACAACCTCCTCAATGAGGATTCCGAGGTGGATGTGCAGCTCAATAACCGGCACA

TGATGATCCGAGGCGAGAACATGTCCAAGATCTTCAAGGTGCGCTCCACAGTGGTGCAGGCCTTCAGGGA

CCATTTCTTTGCCAACGGATACTATGAAGTCACGCCACCGACCCTGGTCCAGACGCAGGTGGAAGGTGGC

TCCACCCTGTTCAAGCTGGATTACTTTGGAGAAGAGGCCTACTTGACGCAGTCGTCCCAGCTCTACCTAG

AGACCTGCATTCCGGCGCTGGGAGATGTGTTCTGCATCGCGCAGTCGTACAGAGCTGAGCAATCCAGGAC

CCGCAGGCATCTGGCAGAATACACCCACATCGAAGCCGAATGTCCCTTTATAAGCTTTGAGGATTTATTG

AATCGTCTGGAGAACTTGGTGTGCGATGTAGTGGACAGAGTCTTGAAATCGCCTGCCTCAAGCTTACTGT

ACGACCTGAACCCGGGCTTCAAGCCGCCCAAGCGACCCTTCCGCCGGATGAACTATGCAGAGGCCATTGA

GTGGCTGAAGGAACATGATGTGAAGAAGGAGGATGGCACATACTATGAGTTTGGGGAGGATATTCCAGAA

GCTCCCGAGAGGCTGATGACGGACACCATCAACGAGCCCATCTTGTTGTGCCGATTCCCTGCTGAGATAA

AGTCTTTCTACATGCAGCGCTGCCACGATGATTCCCGGCTTACGGAATCTGTGGATGTGCTGATGCCCAA

CGTCGGTGAAATCGTCGGAGGCTCCATGCGTATCTGGGACAGCGAGGAGCTGCTCGAAGGCTACAAGAGA

GAGGGCATCGATCCCACGCCGTACTACTGGTACACAGATCAGAGAAAATACGGCACGTGCCCCCATGGCG

GATATGGTTTGGGCTTGGAGCGGTTCCTAACCTGGATTCTGAACAGACACCACATCCGAGACGTCTGTCT

CTACCCACGCTTCGTCCAGCGCTGTAAACCGTAG

>Common cuckoo ONECUT2

ATGNNNCTGAAGCGCTACAGCATCCCGCAGGCCATCTTCGCGCAGAGGGTGCTCGGCCGCTCGCAGGGCA

CCCTGTCCGACCTGCTGCGGAACCCCAAGCCCTGGANNNNNGCCTGCAAACGCAAAGAGCAAGAACCCAA

CAAAGAGCGGAACCACGCCCAGAAGAAATCCCGCCTGGTTTTCACGGACCTCCAGCGCCGGACGCTCTTA

GCCATCTTCAAGGAGAACAAACGCCCTTCCAAAGAAATGCAGATCACCATCTCCCAGCAGCTGGGCCTGG

AGCTCACCACCGTCAGCAACTTCTTCATGAACGCCCGCCGGCGCAGCCTGGAGAAGTGGCAGGACGACCT

GAGCTCCTGCGGCTCCTCCGCGGCCTCCAGCACCTGCACCAAAGTGTGA

>Common cuckoo POLI

ATGGTGGTGGCCGGGACTGGCCATAAGGGCACTGATACTGGTGGCCATGAGGGCAGCCAGAGTGGTGGCA

GTGAGGGGGTGATGGTAGTGGCCATAACGGGAGTAACAATGGTGGCCATGAAACACGTTTACTTCCCTGC

CTGCTACTCCAAATCAAACATTTCTGCATCCGGGATGAAGTACCTGTCAGGAGGACACAACACATCCGTT

TCAATGTCCCGAGTGATTGTCCACATGGACCTGGACTGCTTTTACGCCCAAGTGGAGATGATTCGGAAGC

CAGAACTGAAAGACAAACCTTTAGGTGTGCAGCAGAAGTCCATCGTGGTCACCTGTAACTACGTCGCCAG

AAATCTCGGCGTGAAGAAACTGATGGCGGTGAAGGCCGCCAAGGAGATATGTCCTCACCTGGTCCTGGTT

AACGGAGAAGACCTGACGCCCTACAGAGAAACGTCCTACAAGGTTACAGAGTTGTTGGAGGAGTTCTGTC

CTCTGGTGGAAAGACTCGGCTTCGATGAGAACTTCGTGGACGTCACAGAGATGGTGGAGAAGAGACTAAA

GCAGCAGAAGACTTCTTCTGACCTCGTCGTGGCCGGTCACGTCTACAACAACCACGCCATCGATCTCCAC

GACAGAACCCACGTGGCGCTGGTTCTGGGCTCTCAGATCGCAGAGGACATCAGGCGAGCCGTGGCCACCA

CCCTGGGCCTCACCAGCTGCGCGGGAGTGGCCTCCAACAAACTCTTGGCCAAACTTGTCTCCGGGACCTT

CAAACCCAACCAACAAACCGTTCTCCTGCCACAAAGCCGACAAGATCTTCTCCGTGCTCTTCGTGGTGTC

CAAAAGATTCCTGGCATCGGCTACAAAACAGCCAAACGCTTGGAAACGTTGGGAATCGAGAGCGTTTGGC

AGCTCCAAACCTTTCCGGCCACCGCGTTGGAGAAGGAATTGGGCCTTTCTCTCGCTCGGCGCATCCAGAA

GCTCAGCTACGGCGACGATGACTCTCCGGTGACTCCATCAGGTCCTCCTCAGTCCTTCAGCGACGAAGAT

TCCTTCAAGAAGTGCTCAACGGAAGCAGAAGTCAAAGAGAAACTTCTGGAGCTGCTCCCGAACCTCTTAG

AAAGGGTCTACAAAGATGGCCGACGACCTCACACGTTGAGACTCACCATCCGCCAGAACTCCTCGAGCGA

GCGATGGTTCCACCGCGAGAGCCGCCAATGTCCAATCCCTCCTCATCTCCTTCAACGCTTCGGTCAAGGT

AACGCCAACCTTCTCTCGCCGTTGGTGGACGTCTTGATGAAGCTCTTCCATAAGATGATAAACGTGGAGG

TCCCGTTTCATCTCACCCTTCTAAGCGTGACCTTCTCCAACCTCAAAGAACTTCCCAGCACCAAGAAAGG

ATCCATCGGTTTCTACCTCAAGGAGATGCCATCAAAACGTGACCGGGAAGTGGACGATGG

>Common cuckoo RPL17

ATGGTGCGCTACTCGCTGGATCCGGAGAACCCCACGAAATCGTGCAAGTCCCGGGGCTCCAACCTGCGGG

TGCACTTCAAGAACACCCGTGAGACAGCCCAGGCCATAAAGGGCATGCACATCCGCAAGGCCACCAAATA

CCTGAAGGACGTCACCCTGAAGAAGCAGTGTGTTCCCTTCCGGCGCTACAATGGGGGAGTCGGCAGGTGT

GCCCAGGCCAAGCAGTGGGGCTGGACGCAGGGACGCTGGCCGAAGAAAAGCGCTGAGTTCTTGCTGCACA

TGCTGAAGAACGCAGAGAGCAACGCTGAGCTCAAGGGTCTGGATGTGGATTCATTGGTGATAGAACATAT

CCAGGTCAACAAGGCTCCCAAAATGCGCCGACGCACCTACAGAGCGCATGGGCGGATCAACCCCTACATG

AGCTCCCCCTGCCACATTGAGATGATACTTACCGAGAAGGAGCAGATCGTTCCCAAGCCGGAGGAGGAGG

TGGCCCAGAAGAAAAAGATCTCCCAGAAGAAACTGAAGAAGCAGAAGCTGATGGCTCGGGAGTGA

>Common cuckoo SKA1

ATGTCTTCTTCAAGCTTTGAAGATTTATGCACTCATATGAATGCGAAGATTTCAGCAACTAAAAAGCGTC

TTGTACTTAGAAGTATAGGCAATGAACAATCGCTCAGATCTGTGGTCTCCAACTTGGGACAGGACTTGGC

TGTCTGTCACAGGCTCCTGGTTGAAATGGAAGCAGAGGGTCAGCAGCAAGAAAAACTAAGGGGTCGGGAA

ATGATAAAGATGTCTGTTGAGAGACAGCGAAGCGAAGCAGAGCACCTCCAGGAAAACGTTCCTCCATACC

TGCCCAAACCAACTCGGAGCTGCAACACCGGGCCAGCTGTCAAACGGGAGGAGGCAACAAAAGCCGTTGA

ACCTGAATGGTTAAAGAAATGTAGAGAAGAGGCAAGAGCTTATAAAGAAGTGCCGTTAATAAGCCCTGAA

GAATTTGATAACATTCCTGCGTACTTGAGAGGCCGTTTAAGATGTGAGCAGATTAATGTTGTCATTCAAG

AGATCAACAATGCTGTGGCTGCCAAGTACAAGATCATGCATCAGCCGTTGAAATCTATGAAAGCACCAGC

CAGGAAACTCTACAGCAGGTTCCTGGAAGAAGAAATAAAGGAAACAAGAGGTGAATTTTTCATCATGGAG

GCTGACATCAAAGAATTCACCCAGCTGAAGATGGATAAGCGCTGCTACAACATCCTTAGTATCCTGCGCC

ACGCCCAGAGACTGAGAGAAATTCGTTGTTCTGGATTTGTCCGCTACATCATCTGCTGA

>Common cuckoo SMAD4

ATGGACAATATGTCTATTACTAACACGCCAACAAGTAATGACGCTTGTCTGAGCATTGTCCACAGCTTGA

TGTGCCATCGACAAGGTGGAGAGAGCGAAACTTTTGCCAAACGCGCCATTGAAAGCTTAGTTAAAAAGCT

AAAGGAGAAGAAAGACGAATTGGATTCTCTGATTACAGCTATAACCACAAACGGAGCTCATCCTAGCAAG

TGCGTTACAATACAGAGGACACTGGATGGGAGGCTTCAGGTGGCTGGTCGCAAGGGGTTCCCTCATGTGA

TTTACGCTCGTCTTTGGCGGTGGCCTGATCTTCATAAAAATGAACTCAAGCACGTTAAATATTGTCAGTA

TGCTTTTGACTTAAAGTGTGACAGTGTCTGTGTAAATCCTTACCATTATGAGCGTGTAGTATCGCCTGGC

ATCGATCTCTCAGGACTGACACTACAGAGCTCTGCTCCATCAAGCATGCTGGTGAAAGACGAATATGTTC

ATGACTATGAGGGGCAGCCATCACTTTCATCTGCCGAAGGCCATTCAGTCCAAACCATCCAGCATCCGCC

AAGCAACAGGGCATCTACGGAGCCTTACAGCACCCCAGCCATGTTAGCTCCCTCTGAGGCCAGCACTACC

AGCACCACTAATTTTCCCAACATTCCTGTGGCTTCAACAAGTCAACCGACCAGTATATTGACAGGTAGCC

ATAGTGATGGACTCTTACAGATTGCTTCAGGGCCTCAGCCAGGAACTCAGCAGAATGGGTTTACAGCTCA

GCCAGCTACTTACCATCACAACAGTACTACCACTTGGACTGGGAGTCGGACGGCAGCCTACACGCCGACC

ATACCTCACCACCAGAACGGCCACCTTCAGCACCATCCGCCTATGCATCCAGGACACTACTGGCCAGTTC

ACAACGAACTTGCATTCCAGCCTCCTATATCAAATCATCCTGCTCCAGAATACTGGTGTTCAATCGCATA

TTTCGAAATGGATGTGCAAGTTGGGGAAACATTTAAGGTCCCTTCAAGCTGTCCAATTGTTACCGTCGAT

GGATATGTGGATCCTTCCGGAGGAGACCGTTTTTGCCTAGGCCAGCTTTCCAACGTGCATAGAACAGAAG

CCATTGAGAGAGCAAGGTTGCACATAGGGAAAGGGGTGCAGCTGGAGTGCAAAGGAGAAGGCGACGTGTG

GGTTAGGTGCCTCAGCGACCACGCGGTCTTCGTTCAGAGTTATTACCTGGATAGAGAAGCAGGTCGTGCA

CCAGGGGATGCTGTCCACAAGATCTACCCAAGTGCATATATAAAGGTGTTTGATTTGCGCCAGTGTCACC

GTCAGATGCAACAGCAGGCTGCCACTGCCCAAGCTGCTGCTGCTGCTCAAGCTGCAGCTGTAGCAGGAAA

CATCCCCGGACCAGGATCAGTAGGTGGAATAGCCCCAGCCATTAGTTTGTCAGCTGCCGCTGGAATTGGT

GTCGATGACCTGCGCCGCTTGTGCATACTCAGGATGAGTTTTGTAAAAGGTTGGGGACCTGATTACCCAA

GGCAGAGCATCAAAGAGACGCCGTGCTGGATTGAAATTCACTTACACCGCGCCCTCCAGCTTCTCGATGA

AGTACTTCATACCATGCCTATCGCAGACCCACAACCTTTAGACTGA

>Common cuckoo SMAD7

ATGNNNGAACTGAAGGCGCTGACCCACGCCGTGCTCAAGCGATTGAAGGAGCGGCAGCTGGAGGGGCTGC

TGCACGCCGTGGAGTCCCGCGGCGGGGCGAGGACCCCCTGCCTGCTGCTGCCCGCTAAAGCCGATTCCCG

GCTGGGGCAGCACTGGTACCCGCTGCCCGTGCTGCTCTGCAAGGTGTTCCGCTGGCCCGACCTCCGGCAC

TGCTCCCAGGTGAAGCGCCTGTGTGGCTGTGAATCCTACGGCAAGGCTCACCCCGAGCTGCTCTGCCTTT

ATTTTCTCTCTACTTTTCTAATTCCAGAGTCTCCCCCTCCACCCTACTCCAGATATCCAATGGATTTTCT

CAAACCAACTGATTGTCCAGACTCTGTGCCTTCCTCCACTGAAACAGGGGGAACTAATTATCTAGCCCCT

GGGGGGCTTTCAGATTCCCAAGTTCTTCAGGAGCCGGGGCATCGGTCACACTGGTGCGTGGTGGCATACT

GGGAAGAGAAAACGCGTGTGGGTAGGCTGTACTCTGTCCAAGAGCCCTCCCTGGATATCTTCTATGATCT

ACCTCAGGGGAATGGTTTCTGCCTCGGACAGCTCAACTCGGACAACAAAAGCCAACTGGTGCAGAAGGTC

CGCAGCAAGATTGGCTATGGCATCCAGCTTACCAAGGAAGTGGACGGCGTGTGGGTGTACAACCGCAGCA

GTTACCCCATCTTCATCAAGTCGGCCACACTGGACAACCCTGACTCCAGGACGTTGCTGGTTCACAAAGT

GTTCCCAGGTTTTTCCATCAAGGCTTTTGACTACGAGAAGGCGTACAGCTTGCAGAGACCCAACGACCAT

GAGTTCATGCAGCAACCATGGACCGGATTTACTGTTCAGATCAGCTTTGTGAAAGGCTGGGGCCAGTGCT

ACACGAGACAGTTCATCAGCAGTTGCCCATGCTGGTTGGAGGTTATTTTTAATAACCGATGA

>Common cuckoo ST8SIA3

ATGGGCGACGCCAATCATGGGTGGACGCTCGATCTTCTAAGGTCGCAGTTTGCTCTCAAGTTCCTGGACC

CGTCCTTCGTGCCCCTCACCAACTCGCTGAGCCAGGAGCTGCAGGACCAGCCCCCCAAGTGGACCTTCAA

CCGCACGGCCTTCTCCCGCCAGAGGCAAGAAATCCTTCAGCACGTTGATGTCATCAAGAACTTCTCGCTG

ACCAAGAGCAGCGTCCGCATCGGCCAGCTGATGCATTACGACTACTCCAGCCATAAGTACGTCTTCTCNN

NNNGCAACAACTTCCGCTCGCTGCTCCCCGAGGTCTCGCCCATCCTCCACAAGCACCACAACATCTGCGC

CGTCGTTGGCAACAGCGGGATCCTGACCGGCAGCCAGTGCGGCCAAGAGATCGATCGATCCGATTTCGTT

TTCCGTTGCAATTTCGCTCCCACCGAGGCCTTCCAAAAAGACGTCGGAAGGAAAACCAACCTGACCACCT

TCAACCCCAGCATCCTGGAGAAGTACTACAACAACCTCTTGACCATTCAAGACCGCAACAACTTCTTCTT

GAGTTTGAAGAAGCTGGACGGCGCCATCCTTTGGATCCCCGCCTTCTTCTTCCACACGTCGGCCACGGTG

ACCAGAACGCTGGTTGACTTCTTTGTGGAGCACAGAGGACAGCTGAAGGTCCAGCTGGCTTGGCCGGGGA

ATATAATGCAGCACGTCAACAGGTACTGGAAGAACAAGCACTTGTCTCCCAAGCGGCTGAGCACGGGTAT

CCTCATGTACACCCTCGCCTCGGCCATCTGCGAGGAGATCCACCTCTACGGCTTCTGGCCCTTCGGGTTT

GACCCCAACACGCGGGAGGACCTCCCCTACCACTACTACGATAAGAAGGGCACCAAGTTCACCACCAAGT

GGCAGGAGTCCCACCAGCTGCCGGCAGAGTTCCAGCTGCTCTACAGGATGCACGGTGAAGGACTGGCCAA

GCTCACCTTGTCGCGTTGTGCCTAA

>Common cuckoo TCF4

ATGCATCACCAACAGCGAATGGCTGCCTTAGGGACGGACAAAGAACTCAGTGATTTACTGGATTTCAGCG

CGATGTTTTCACCTCCTGTGAGCAGCGGGAAAAACGGACCAACTTCCTTGGCAAGCGGACATTTTACGGG

CTCAAGTGTAGAAGAACGAAGCGGTTCCGGCTCCTGGGCCAACGCGGGACATCCAAGTCCATCCCGGAAC

TATGGAGATGGGACTCCCTACGACCACTTGGCCAGCAGAGACCTTGGTTCTCACGACAATCTTTCTCCTC

CCTTCGTTAACTCCCGAATACAAAGTAAAACGGAACGAGGGTCCTACTCGTCCTATGGAAGAGACTCCAA

TCTACAAGGTTGCCACCAGCAAAGTCTTCTCGGCGCCGAGATGGAGATGGGCACCGCCACCGCGCTGTCC

CCCACCAAGCCCGGCTCCCAGTACTACCAGTACTCCAGCAATAACCCCCGCCGGCGGCCTCTCCACGGCA

CCTCCATGGAAGTCCAAACAAAGAAAGTTCGGAAAGTTCCTCCGGGTTTGCCATCTTCTGTCTACGCTCC

GTCGGGCAGCACTGCCGACTACAACCGCGACTCGCCGGGGTATCCGTCCTCCAAACCAGCAGCCGGCACC

TTCCCCACCTCCTTCTTCATGCAAGATGGACATCCCAGCAGCGACCCGTGGAGCTCCTCCAGCGGGATGA

ACCAACCGGGCTACGGCGCCATGTTGGGCAACTCTTCTCACATCCCACAGTCCAGCAGCTACTGCAGCCT

CCACCCCCACGAGCGCCTGAGTTACCCGTCCCACTCCTCGGCCGACATCAACTCCAGTCTTCCTCCGATG

TCCACCTTCCACCGCGGTGGCTCCGCCCACTACAGCGCAGCGTCCTGCACGCCGCCCGCCAACGGCACCG

ACGCCATCATGGGCGGCCGAGGGGGCGGAGCCGGCGCTTCGCAGACCGGAGACGCGTTGGGCAAAGCTCT

CGCCTCTATCTACTCTCCAGACCACACCACCACCAGCTTCCCATCAAATCCTTCAACTCCTGTCGGTTCT

CCCCCTTCTCTCGCAGCAGGCCCCGCTGGGTGGTCTCGCGGTGGAGGTCCAGCGGCGTCATCTCCTAATT

ATGAAGGTCCTTTACACTCCTTGCAAAGCCGCATCGAGGACCGTTTGGAGCGCTTGGACGACGCCATCCA

CGTTCTGCGCAACCACGGATCTTCATGGAATGGGTTGGAGGCATCTGGAGGGCCTTTGGACGATGGGCTG

GGCGCGGGGCTCCCAGCGTCGTCATCGGGAAGCTCCGAGATCAAATCGGACGAGGAAGGAGATGAGAACC

TTCAGGACCCCAAATCCACGGAGGAGAAGAAGCTGGAGGAGGACAAGAAGGAGCTCAAATCCATTACTAG

GTCAAGATCTAGCAATAACGACGATGAGGATCTGACCCCGGAGCAGAAGGCGGAGCGGGAGAAGGAGCGG

CGCATGGCGAACAACGCCCGCGAGCGCCTCCGCGTCCGCGACATCAACGAAGCCTTCAAAGAGTTGGGGC

GGATGGTGCAGCTCCACCTCAAGAGCGACAAACCCCAGACCAAGCTCCTCATCCTCCACCAGGCCGTGGC

CGTCATCCTCAGCCTCGAGCAGCAGGTCCGAGAACGCAACCTGAACCCGAAAGCCGCGTGTCTCAAACGG

AGAGAAGGAGAGAAGGTCTCCTCAGAT

>Common cuckoo TSPAN3L

ATGGACTGCGGCGTCATCACCTCCAAAACCCTGCTAATAACTTTCTCCATTGCATTTCAGGCAGCAGCAG

CAGGTCTCAGCTATGTCGGGGCGTATGTCATTAACACCTACAAGAGCTATGACAACTTTCTGCAGGACAA

GTACGCTCTCTTGCCTGCCGTCATCATTATTTGCGTCGCTGTGGTGATGTTCATCATCGGGTTGATAGGC

TGCTGTGCCACCTTCCGGGAGTCTCGAGTCGGCCTCGGACTGTTCTTAGCCATTATCCTGGTTATCTTTA

TCGCAGAAGTATCCGCTTTTGTCCTGGGATTTGTTTACAGGGAAAAGGTAAAAACTGATGTGCAGGACAC

AATGCGCTCAGTCTTTGAGAAGTACGATAGCAGTAATCCAGAGTCTACTGTTGTGGATTACTTGCAAGAA

CAGCTCCATTGTTGTGGGGTGAAGAACTACAGTGACTGGACAACCACGCAGTGGTTTAATTCCACTGGTA

ACAACAGTGTCCCCTTGAGCTGCTGCAGAGAAGACATGAAGAACTGCACAGGGCGCCTGGATCAGCCAGA

GGAGCTCAATAGGCAGGGCTGTGCAGGGGAGCTGGAGTCTGGGCTGCAGAGCGTTATCAGCTATGCTATG

CTTGTGATCTTGGGGTTTGCCATTGTAAAGTTCTTTGGCATGCTGAGTGTCTGTGTGCTCACTTGCAAGA

GAGAAGACAGCGGATACCAGCCTCTCTACTCAGGGGTGTTTGCTTGA

>Common cuckoo TXNL1

ATGGGGGGCGTGAAGGTGATCGGCAACGACGCCGAGTTCCAGCCCGAGCTGAGCGCCGCGGGGGCGCGCC

TCGCCGTCGTCAAGTTCACCATGAGGGGGTGTGGTCCGTGTTTGAGGATCGCTCCGGCCTTCAACGCGTT

GAGCAACAAATACCCGCAGGCGACGTTCTTGGAGGTGGATGTCCACCAGTGCCAGGGAACAGCGGCCACC

AACAACATCTCGGCCACTCCTACTTTCCTCTTCTTCCGGAACAAGGTGCGAATCGACCAATACCAAGGAG

CCGACGCCGTTGGTTTAGAAGAGAAGATCAAACAACACTTGGAGAACGATCCCGGCAATAACGAAGACGT

CGATATCCCCAAAGGCTACATGGACCTGATGCCCTTCATCAACAAGGCCGGGTGCGAGTGCCTCAACGAG

AGCGACGAGCACGGCTTCGAGAATTCCTTACGGAAAGACTCCACCTACTTGGAGTCGGACTGCGACGAGC

AGCTGCTCATCACCGTGGCGTTTAGTCAACCTGTCAAGCTTTACTCCATGAAGCTCCAGGGACCAGATAA

CGGCCAAGGTCCAAAGTACGTCAAGATCTTCATCAACCTCCCTCGCTCGATGGACTTTGAAGAAGCCGAG

AGGAGCGAACCCACGCAGGCCTTGGAGCTGAGCCCGGAAGACATCAAAGAAGATGGCATCATCCAACTCC

GCTACGTCAAATTCCAGAACGTCAACAGCGTCACCTTGTTTGTCCAATCCAACCACGGCGATGAGGAGAC

AACCAGGATCACGTACTTCACCTTCATCGGAACTCCTGTCCAAGCCACCAACATGAATGACTTCAAACGA

GTAGTTGGGAAGAAGGGCGAGAGCCATTAG

>Common cuckoo WDR7

ATGGCGGGTAACAGCCTGGTGCTGCCCATCGTCCTGTGGGGCCGCCGGGCCCCCACCCACTGCATCTCCA

CCCTCCTCCTGATGGAGGACGCTTCCATGATCGTCACCGGCTGCCACGACGGGCAGATATGTCTCTGGGA

CCTCTCCTCGGACCTGGAGATCAGCCCCAGAGCTCTGCTCTTTGGTCACACGGCCTCCATCACGTGTCTG

GCCAAGGCCTCCGCTGCGGGTGACAAGCAGTACATCGTCAGCGCCTCAGAGAGCGGGGAGATGTGTCTGT

GGGACGTCAACGATGGGAGATGCATCGAGTTCACCAAGCTGGCCTGCACCCACACCGGCATCCAGTTCTA

CCAGTTATCCGTGGGGTCCCAGCGGGAGGGTAGATTGCTCTGCCACGGCCACTACCCAGAAATACTCGTC

ATGGACGCAACCAGCCTTGAAGTTCTCTACTCCTTGTTGTCCAAGATCTCTCCAGACTGGATCAGCTCCA

TGACCATCATCCGCTCCAGCAGAACCCAAGAAGACACGGTGGTGGCGGTTTCAGTGACCGGCATCCTCAA

GGTGTGGATCATCTCCTCCGAAGCCGCTCGGATGCAGGAGACAACGCCGGTGTTTGAAGAGGAGTCCAAA

CCCATCTACTGTCAGAACTGCCAAAGCATCTCGTTCTGCGCCTTCACCCAGCGCTCCGTCCTGGTGGTCT

GCTCCAAGTACTGGAGGGTCTTCGACGCTGGTGATTATTCCCTCTTGTGCTCCGTGCCCGGCGAGGCCGA

CCAGACCTGGACCGGTGGGGACTTTGTGGCAGCTGACAAAGTCATCGTTTGGACAGAAGATGGCCGAAGC

TTCATCTACAAGTTACCAGCCAGTTGTCTCCCAGCCAGCGACTCCTTCCGCAGCGACGTGGGGAAACCGG

TGGAGAACCTCATCCCACCGCTGCTCCACAGCGTCTTGGACCGAGTAGACAAACAGCTGCTGATCTGCCC

GCCGGTGACTCGGTTCTTCTACAGTCCCCAGGACTTGGCCTACAAGCTCTTGATCCAAGGCGATTCTTCT

GGAAGGCTCAGGATTTGGAACGTTCCGGATACGCTGGAGCAGGAGGGGACGAAAGGCCTCCAAAGCACCA

CGTCGGTGTCCCTCCAGGAAGCCTTTGAGAAGCTGAGCCCCGGTCCCGCTGGCATCATCGACCAGCTGAG

CGTGGTGCCCAGCAGCCCGGAGCCCCTCAAGGTGACGGCCAGCGTCTACATCCCGGCCCACGGGCGCCTG

GTCTGCGGGCGCGAGGACGGCAGCATCGTCATCGTCCCGGCCACGCAGACGGCCATCGTGCAGCTGCTGC

AGGGCGAGCACACGCTCCGCAGAGGTTGGCCACCCCACCGGACTCTGCGAGGTCACCGCAACAAAGTCAC

CTGTTTGCTGTACCCTCATCAGGTTTCTTCTCGTTACGATCAAAGGTTTTTGATCTCGGGGGGGGTGGAT

TTCTCCGTCATCGTCTGGGACATCTTCTCCGGTGAGATGAAACACATCTTCTGCGTCCACGGAGGAGAAA

TCACCCAACTCCTCGTCCCACCAGAGAACTGCAGCGCGCGGGTGCAGCACTGCGTCTGCTCGGTGGCCAG

TGACCACTCGGTGGGGCTGCTCAGTCTCCGAGAGAAGAAATGCATCATGTTGGCATCTCGGCACCTTTTC

CCCATCCAAGTCATCAAGTGGAGACCTTCTGATGACTACCTGGTGGTGGGATGCTCCGATGGATCCGTCT

ACGTCTGGCAGATGGACACAGGTGCTCTGGACCGTTGCGTGATGGGCATCACGGCGGTGGAGATCCTCAG

CGCCTGCGACGAGGCCGTGCCGGCCGCCGTGGACAACCTGAGCCACCCGGCGGTCAACCTGAAGCAGGCC

ATGATCCGGCCGAGCCTGGCCTCTGAAGCGGCCGACAAGGGGAACCTGCCCAAATACTCCCATAATTCCC

TCATGGTTCAAGCTCTAAAGACCAACGTGACGGACCCCGACATCCACGTGCTCTTCTTCGACGTGGAGGC

CCTCATCATCCAGCTCCTGACCGAGGAGGCCTCCAGGCCCAACGCCGCCATGGTCTCTCCTGAGAACCTG

CCGAAAGCTTCTGGTGGCTCCGAGAAAGGAGGGTCCTTCTTGGCCGGGAACCGGGCGGCCGTCCTCTTCC

AGCAGGTGAAGGAAACCATCAAAGAGAACATCAAAGAACATCTCTTAGATGATGAAGATGAAGATGAGGA

GACCTTACGGCAAAGAAGAGAAGATGGTGACCCCGAGTATCGGTCGAGCAAGTCCAAACCCTTGACCTTG

CTGGAGTACAACCTCACCATGGACACGGCCAAGCTCTTCATGTCCTGTCTTCACGCCTGGGGCCTCAACT

CGGTCCTGGATGAGCTTTGTCTCCACCGTCTGGGGATGCTGAAGCCCCACTGCTCCGTGTCCTTCGGTCT

CCTCTCCAGAGGAGGCCACATGTCCCTCATGCTCCCGGGCTTCAACCAGCCGGTGGGCCGAACTCCGTCG

GGGCCGCTGGAGGTGGGCAGGAAGATGTCCATCACCGAAGGGTTGGGAAAGGGCACCTACGGAGTGTCCC

GGGCCGTCACCACTCAACATCTCCTCTCCGTCATCTCCTTGGCCAACACGCTGATGAGCATGACCAACGC

CACCTTCATCGGGGACCACATGAAGAAAGGGCCCACCAGGCCACCGAGACCGGGGACTCCAGAGATGACC

AAAGCGAAGGCCCCACCTTCCGTTTCGAGTCACGCGGCCCAAGGGCAGATCAAGCAAGGTAGGAGAGCTC

TTGCTTTGGAGCAATCATGGATGGGTTGGGTTGGAGGGACCTCCAAGCCCAGCCGGTTCCATGGGCAGGG

CCACCAGGGGTGGAGCCAGCTGGCGGCCATGCACTGCGTCATGCTCCCGGACCTCCTGGGCCTGGAGAAG

TTCCGCCCTCCTCTCCTGGAGATGCTGGCGCGCCGCTGGCAGGATCGCTGCTTGGAGGTGCGAGAAGCCG

CCCAGGCCCTGTTGCTGGCCGAGCTGAGAAGAATCGAGCAGGTGGGACGGAAGGAAACCATCGACGCGTG

GGCTCCTTACCTCCCCCAGTACATGGACAACGTCATCTCCCCCGGAGTCACCACGGAAGCTCTCCAGACC

GGAACGGCCAACTCGGACACCTCGGGGACAGAGGCCAAAGTTCAAGAAGAAGAACACGATCTGGTGGACG

ATGACGTCACAGCAGGTTGTCTCCCTGGCCTGCCGCCCATGAAGAAGGTCTCCACGTCCTACGAGGAGAG

GCGCAAGCAGGCCACGGCCATCGTCCTCCTGGGCGTCATCGGGGCCGAGTTTGGAGCCGAAATCGAACCT

CCCAAACTTCTGACCCGCCCTCGGAGCTCCAGTCAAATTCCGGAGGGCTTCGGCCTGACCAGCGCTGGCT

CCAACTACTCCTTGGCCCGGCACACCTGCAAAGCGCTGACGTTCCTGCTGCTGCAGCCGCCCAGCGCCAA

ACTGCCGGCGCACAGCACCATCCGCAGGACGGCCATCGACCTCATCGGCCGCGGCTTCACCGTCTGGGAG

CCCTACATGGACGTCTCGGCCGTGCTCATGGGCCTCCTGGAGCTCTGCGCCGACGCCGAGAAGCACTTGG

CCAACATCACGCTGGGTTTGCCCCTCAGCCCGGCGGCCGACTCGGCCCGCTCGGCTCGCCACGCCCTGTC

CCTCATCGCCTCCGCCCGCCCGCCCGCCTTCATCACCACCATCGCCAAGGAGGTGCACCGGCACACGGCG

CTGGCGGCCAACACGCAGTCGCAGCAGAACATCCACACCACCACCCTGGCCCGCGCCAAGGCCGAGATCC

TGCGCGTCATCGAGATCCTCATCGAGAAGATGCCCACCGACGTGGTGGACCTTCTCGTGGAGGTGATGGA

CATCATCATGTACTGCTTGGAAGGTTCTTTGGTCAAGAAGAAGGGCCTTCAGGAATGCTTTCCGGCCATC

TGCAGGTTCTACATGGTCAGCTACTACGAGCGGAGCCACCGGATCGCGGTGGGAGCTCGTCACGGCTCCG

TCGCCCTCTACGACATCCGCACGGGCAAGTGTCAGACCATCCATGGTCATAAAGGTCCCATCACTGCGGT

GGCTTTTGCTCCTGATGGCCGCTACCTTGCTACCTACTCCAACTCCGACAGCCACCTCTCCTTCTGGCAG

ATGAACACATCTCTCCTGGGGAGCATCGGCATGTTGAACTCGGCGCCTCAGCTCCGCTGCATCAAGACCT

ACCAGGTCCCACCAGTCCAACCAGCTTCTCCCGGTTCCCACAATGCCCTGCGCTTGGCCCGCCTCATCTG

GACCTCCAACCGCAACGTCATCCTCATGGCCCACGATGGCAAGGAGCATCGCTTCATGGTCTAG

>Drosophila melanogaster Frazzled

ATGGCCATCACAACAAACAGAAGCAGCAGGACTTTGTGGAACTGGCTGCTCAGCAGTTGTCTCATATTCC

AGCTGATTGGCAGCAGTTTGGCAAGTCAAGCTCTTTCGTTCACCTTGGAGCCCCAGGATGCCGTGGTGCC

CGAGGGCCACTCGGTGCTGCTCCAGTGTGCAGGCACTGCATCCATTGGACGTGGAGGCAAGAGCAAGTCC

AACTTACCCAGCTCAGTGAGCATCCGGTGGCGAGGACCTGATGGGCAGGACCTGGTCATCGTGGGCGATA

CGTTCCGCACTCAGCTGAAGAATGGATCACTGTACATTAGCTCCGTTGAGGAGAACCGTGGACTGACGGG

AGCCTACCAATGCCTGTTGACTGCCGAGGGCGTGGGCAGTATACTCAGTCGACCCGCACTGGTGGCCATT

GTCCGACAGCCAGATCTGAATCAGGACTTCCTGGAAACCTACCTGCTGCCAGGTCAGACGGCCTACTTCC

GTTGCATGCTGGGCGAAGCCAACTGGCAGGAGGGCGTTAAGCATTCGGTACAGTGGCTCAAGGATGACCT

GCCACTGCCCCTGGACAAACTGCGCATGGTGGTGCTGCCCAACGGAGCGCTGGAGATCGACGAAGTGGGT

CCATCGGATCGCGGATCCTATCAGTGCAACGTGACTTCGGGCAGTTCCTCCCGGCTAAGCAGCAAAACGA

ACTTGAACATTAAGAAACCCAGTGACCCGGGTGTCGAGAACTCAGTGGCACCATCGTTCCTTGTGGGACC

TTCGCCAAAAACCGTGAGAGAGGGCGACACCGTCACCCTGGACTGTGTGGCCAATGGAGTGCCAAAGCCG

CAGATTAAGTGGCTGCGAAATGGCATGGATCTGGATTTCAACGACCTGGACTCGCGCTTTTCCATCGTTG

GAACGGGTTCGCTGCAAATCTCCAGTGCCGAGGACATTGATTCAGGAAACTACCAGTGCCGGGCCAGCAA

TACGGTGGATTCACTTGATGCTCAGGCCACGGTGCAGGTACAGGAACCACCTAAGTTCATTAAGGCGCCC

AAGGATACCACTGCCCACGAGAAGGACGAACCGGAATTGAAGTGCGATATCTGGGGAAAGCCAAAGCCCG

TCATAAGGTGGCTTAAGAACGGAGATCTCATAACGCCCAATGACTACATGCAACTGGTGGATGGTCACAA

TCTGAAGATCCTCGGACTTCTCAACTCCGACGCTGGAATGTTCCAGTGCGTGGGCACCAATGCCGCTGGC

AGTGTTCATGCTGCAGCTCGTCTGCGAGTTGTTCCGCAAGGAGACTCCCCCGAACAGGATCCGAGTGTTC

CGCATCCAGGTGGCAAGCCGTTGGACAGCGGACTCCAGGCCCGATTGCCCAGCCAGCCGCGCGATCTGGT

GGCGCAGATAGTGAAGTCCCGATTCGTGACCCTCAGCTGGGTGGAACCACTCCAAAATGCCGGCGATGTA

GTCTACTACACGGTCTACTACAAGATGAACAACAGCGAGAGGGAGCAAAAAATGGTTACCAAGTCCCATG

ACGATCAGCAGGTCAATATTCAATCGTTGCTGCCCGGCAGAACCTATCAGTTCCGAGTGGAGGCCAATAC

CAATTTCGGCAGCGGTGCATCTTCTGCTCCGCTAGAAGTTAGTACCCAACCGGAGGTGAACATTGCAGGT

CCGCCGCGCAACTTTGAAGGCTATGCACGCAGTCACAAGGAGATCTACGTCAAATGGGAAGAGCCCACGG

TAACCAATGGAGAAATACTCAAGTATCGAGTGTACTATTCCGAGAACGACAGTGGTGCCGATCTATATCA

CGATAGTACTGCTTTGGAAGCAGTTCTTACCGAGTTGCGTCCCCACACGGATTACGTGATTTCCGTAGTG

CCTTTTAATCGAAACGGAATGGGTGACTCCTCAGCGGAGATACGTGTTAAGACGTTTTCGTCCACGCCGT

CGGAACCTCCTAATAATGTTACTCTCGAGGTGACCAGCTCCAGCTCCATTACCGTCCACTGGGAGCCGCC

TGCAGAGGAAGATCGCAATGGCCAAATTACGGGCTACAAGATTCGCTATCGCAAGTTCAAGGATGCACCG

CAGGTCAAGAGTACTCCTGCCAATATTCGTTACTTTGAGTTGAGCAACTTGGACCGCAATGCCGAGTATC

AGGTGAAGATCGCTGCAATGACAGTGAACGGTTCCGGACCCTTCACGGAATGGAATCGTGCTAACACCTT

GGAAAACGATCTGGACGAGACCCAAGTTCCGGGCAAACCAATTTGGATTAGCATTCATCCTGGAGCCAAC

AACATTGCCCTGCATTGGGGTCCCCCACAGCATCCGGAAATCAAGATACGCAACTACGTGCTGGGCTGGG

GCCGTGGCATTCCCGATGAGAACACCATAGAGCTAAAGGAGACTGAGCGCTATCATATACTCAAGAATCT

AGAATCGAATATGGATTATGTGGTGTCGCTAAGGGCTAGAAATGTTAAAGGTGACGGTCCGCCTATATAT

GACAACATTAAGACACGCGACGAGGAGCCCGTGGACGCACCCACTCCACTTGAGGTTCCGGTCGGTCTGC

GAGCCATTACCATGTCCAGCTCGTCGATTGTGGTCTACTGGATTGACACGATGCTGAACAAAAATCAGCA

TGTAACAGATAATCGCCACTATACGGTTAGTTATGGCATCACGGGATCCAATCGCTATCGCTATCACAAC

ACCACGGATCTAAACTGCATGATTAATGATCTACGACCCAATACGCAGTATGAGTTTGCAGTTAAAGTGG

TCAAAGGACGAAGGGAGTCCTCCTGGTCGATGTCTGTGTTAAATAGTACATATCAGAATGTTCCTGTCAC

CCCGCCTCGAGAGGTTACTGTGCGCTTGGATGAGATGAATCCGCCCACTGTGATCGTCCAGTGGATACCT

CCGAAACATACTCTTGGTCAGATTACCGGATACAATATTTACTATACCACCGACACCACGAAAAGAGATC

GCGACTGGTCAGTTGAAGCATTTGCCGGCGAGGAAACTATGCTCATGCTACCGAATCTGAAACCTTACAC

AACGTATTACTTTAAGGTTCAGGCTAGAACAACCAAGGGGGCTAATAATGCTCCTTTCTCGGCTCTGGTA

TCGTACACCACAAGTGCAGCCGTTACAATGCAGGAGCCCGACACCATAGCCAAGGGAATCGACAACGAGA

AGCTGTTGTATATAATTATAGCAGCTACCGCTGTAGTTCTACTTGTGGTGCTCTTGGGCGTCCTATTGCT

CTGCCGACGTAAGCCACAATCTTCGCCAGAACACACTAAGAAGAGCTATCAAAAGAACAATGTTGGCGTG

CCCAAGCCACCAGATCTATGGATACACCATGATCAAATGGAGCTCAAAAACATAGACAAGGGCCTGCACA

CAGTGACACCCGTCTGCAGCGATGGAGCCTCGAGCAGTGGTGCCCTCACCCTGCCACGATCAGTGGTGCA

CAGTGAATACGAGGTGGAGACGCCAGTGCCCGGTCATGTGACCAATTCTCTGGACAAGCGGTCCTATGTG

CCAGGATATATGACCACATCAATGAACGGAACGATGGAGCGACCTCAGTACCCGCGCACCCAGTACAGCC

ACCAAAATCGTTCTCACATGACCATGGAGGCTGGCCTCTCCCAGCAGAGTCTCACCCAACCGCAAAGCAA

CTCGATGGCTCAGACCCCAGAGCATCCTTATGGCGGCTACGATGCCAACTTCTGTAACGCGGGAAATGCA

GCTGCAGGCAATGGCTGTGTGTCCACCATTGAAAGTTCTAAGCGTGGCCATCCTCTAAAGAGCTTCAGTG

TGCCGGGGCCACCACCCACTGGCGGAGCCACCCCAGTTACCAAGCATACTCCCGCCGTCACAATACGTCC

ACAAAATCAATCGCCCTACAAGAAACCATCTTTCTCGGCTGCCACGCCCAATCGCCTGCAGGGCGGTGGC

TCAGTAGTGCACTCAACCGATGAGATTCAAAGACTTGCCCCCAGCACATCTACCGAGGAGCTAAACCAAG

AAATGGCCAATCTGGAGGGCCTCATGAAGGATCTAAGCGCAATCACGGCCAACGAGTTCGAGTGTTAA

>Duck ACAA2

ATGGCGCTGCTCAGGGGTGTCTTCATCGTTGCGGCGAAGCGAACTCCTTTCGGGACCTACGGGGGCTTGC

TCAAGGACTTCACGGCCACCGATCTGACCGAACATGCCGCTCGGGCTGCCCTGGCTGCTGGCAAAGTCTC

CCCTGAGATCATCGACAGCGTCATCGTTGGCAGCGTCATGCAGAGCTCTGCGGACGCTATTTACATCGCG

CGACACGTCGGCTTGCGGGTGGGGGTTCCTGTCCCGGTTCCAGCCCTCACCGTCAACAGACTTTGTGGCT

CTGGTTTCCAAGCCATCGCCAATGGATGTCAGGAAATTTGCCTCAATGACTCCGAAGTGGTCCTGTGTGG

TGGAGCTGAGAACATGAGCCAGGCTCCTTACGCAGTTCGAAACATTCGATTCGGAACCAGATTAGGAGCA

GAACTCAAGTTGGAGGACACCTTGTGGGAAGGTCTGACGGACACGCACGTGAAAATACCCATGGCAGTTA

CAGCCGAAAACCTGGCTGCGAAGTACAGCATCACGCGGGAGGACTGTGACCGCTACGCGTTCAAAACACA

GCAGAGATGCAAAGCTGCTCAAGAAGCCGGTCATTTTAACGCTGAGATGGCACCGGTGGAAGTGAAAACA

AAGAAAGGGAAAGAAAGCATGCAAAAGGACGAGCACCCGAAACCCCAGACCACTCTGGAACAATTGGCAA

AACTCCCGGCTGTTTTTAAAAAGGATGGGACAGTCACGGCTGGGAATGCTTCAGGGGTGTGTGACGGAGC

CGGCGCAGTCATCGTTGCCAGCGAATCGGCCCTTAAAAAGCACAGTCTCACTCCTCTGGCGAGAGTTGTA

GCTTATCACTCCTCTGGCTGTGACCCTTCCATAATGGGCATTGGCCCTGTGCCTGCCATTACTGAGGTTC

TGAAGAAAGCAGGGCTGACCCTGAAGGACATGGATTTGGTAGAGGTGAACGAGGCATTTGCACCTCAGTA

TCTGGCTGTCGAGAAAGTGCTGGGCCTCGACCCTGAAAAAACCAACGTCAACGGAGGCGCCATCGCTATC

GGTCATCCTTTGGGTGCTTCGGGAGCACGCATCACAGCTCACCTGGTGCATGAATTAAGGCGTCGTGGTG

GGAAATACGCAGTTGGCTCAGCTTGCATTGGAGGTGGACAAGGCATTGCTCTGCTCATTGAGAACACCGC

CTGA

>Duck C18ORF32

ATGGTGTGCATTCCCTGTATCGTCATTCCCGTTCTTCTCTGGGTCTACAAGAAGTTCCTGGAGCCTTACA

TCTATCCTGTCATCGCACCTTTCATTAAGCGCGTGTGGCCCAAGAAAGCCGTGCAGGAAACGACAGCCAC

AAAACGAGGGCAAGGAGGCAGTGCTGGAAACCCCCGGGCACCTTCAGCCAGCGAAAGGGATAAGGAGGAT

GAGTCTGGAGTTCACAGATTTGAAAGCAACGGGGTTGCGAATGGAATTGCTGCAAAGAGAGCCACAGAAC

TTCCTGACAAGAAAACAGATTAA

>Duck C18ORF54

ATGNNNACGACCAGCTTAATAAAANAAAGCAGCGTCTCCTCTCCTGATTCAACGGTATCTGTTCTTCTAA

CCAGCATTGGCAGCAGCAGTTCATGCTCCTGCAGTTTGATTCAATACAAGGACAAGCTTTACAGCTCTGC

ATCTGAGGCGCTGCAAGCTTATATCGAAGATTTTGATCTAAGTCTCACGTCTTCAGAAATAACCCCTGGG

AAGATCTGCCTGTGTCAAAGTACTCCCAAACCAGCGGAGTTTTCAAAGCATCACGTCAAAGGAAAACGCT

GTGTGTTTTCTGCAACGTTGGATGACTGTAACGAGCACGTGGGATTAAGTTCTTTCGCTTCACCCTGTAG

AGGGGAGATGGAATGTGACCCAGACTCGATTAGTCTTGCGACAGATGATCTGTTAGCTTTTCCAGCAGAT

GGATCGCTGCCCTGCGTCCCGAGCAGGCCTTTTAAATCAAGACATCAAAATAGTGAGTGGAGCAGGTGGC

CACTTAAAAAATCTGTCTGCCCTTCCCACACCATGTCACTCGATACTGAAAGGGGTTCCTGTCTTCAGGA

GAACAGCAAAGCTGATGCTAATCAGAATGTACACAAACCCTTTAATAAAAAGAAATACGATGTGTTTACA

CCTAACAAGTACTCTATCTCCTCGAAAGGAAGTTCCAGACCCTTATCTGTTGAGAACTCTGCCACTTTTC

CTGCTAAGAATTACCCAAGGTGGCTTACCAGTCAGAAGGCTGATTTAAGTGTATCAGGGATAAGCAGTAT

TCCCAGTTTTCACTACCCAGTCTGGCTGAAGAGTCACAACCTTTTCCCTGCTGCAACTAAAGAAAGTGAT

GGACACAATTTTAACACCCAAAGCAAAGCTTCCTCTTCGCAGATTTCTGAAATGCTGAAAAACCAGTGCT

CCGTAGATGAGGACGGTGCTGATTTTCTTAAAAAAAGTAGTTGCCTGGATCTGACAGGTGATAATGAAGT

AGCAGAAAGTTGCAAGTATGACAGCCCAGATGCACTCCTCCAATCTGGTACCTCCTTTTCAAAGCACACC

AAACAATCATTCGGAGAAGACCAACTTGAGCTGCTCACTTTGAAGGCTGAGAGAGATCTGGAGAGTTCAA

ATGAAGGTTTGTCAAACGCTCTGGAAGGTGATGGCAGTCCTTCTACCACAGATATACTCGGAGCAGAAAG

ATCCTGGGAAGATGTTCCGGGTGCTTTCAAATCACCGGTGCCTGCATGCTGGGAGGACAGAAACAATCCT

CTGCCATTCCCCAAGGCAGACATCATCGATAAATTCTTGGAAGACTGTTTAGATGACAAGAACAAGGAAA

ACATCTTTTCTGGAGATCATAAGCACAGACCCCTGGAAGCCTTGAAGCTAATGTTGTTCAAGCTTCAAGC

AATTCAGGGAAGACTAAACCCGAATGAAACAGCTGAGCAAAAGGAAGAGTTTGAAAAACTTTTTGAAAAA

GGAGATGCTGAATTAAAACTCTGTGACAGCGAGATAATCCCTCTTACTAATTCTATTCAGAAGGCTTTAC

ACCATGTGTCACATCTTAAGAGTCTTCCTGAAGATACCAATGTGAAACAAGAACAAAGTGACAATCATCA

A

>Duck CCDC68

ATGTCCGCAGGTCAGAGGAACGCACGCATCGTGACCACCACCCTGCTGCTCACTGAAGAAGTGATGCGGG

AAGACCGGGGCTCGGAGGGGAGCTACGTCCTGTACGGCTCCTCCTGCGCCGAGCTCACCGAAGAAGCTGA

GTATGTGAAAAAGCAGCATCCTCTGGTGCCGGGCAGGAGCGCAGAGCCCAGGAGCAGCGGCTGGAGCTCG

AGCTGCAGCCCCGTGGCGGCGAAGATGAGGGAAACGGAGCAGCAGCTGCTGCTGGTCAGCAGGGAGAACC

AGCTGCTGCGGATCAAGCTGGAAGCCACGAGAGAAGCGGGCGTGCAGGCTCTCCGGGCTGCCTCCCAAAA

ACTCTACGAGAGCTACCAGGCTCGCTCCGAAGAACTTCACAAAACTCAGGAGAAGGAGAAGCAGCAAATA

CAGGCCCGCAGTCGGGAGCAGGAGGGAAAGCTGCAGCAGCAGGAGGAGAAGGCCACGCAGCTCGCCGGTG

CCGTGGAGGAGAGGTGCGCCTGCATCGCGGCCATGGAGCAGCGCGTGCGCAGGATGGAGGAGGAGAAGAA

AACGCTGATGGAAAAGAAAACGTCCTATGAAAAGATGCTTCTGCAGATGATGTCGAGGAACGAAGACAGC

AAACGGTGCCTGGCGGTGCAGCAGGACATCGCCACGCTGCGGGAGCAGATCGGCCACCTGCAGCGCCTGA

TCGGGGCGCAGCACCAGAGCCTGCGCCGCGTCATCCAGGAGGCGGAGGAATTGAACGAAGAACTCAAAAT

CCAGGACAAGAAAATAGAAGATCTGACCGAAAAGGTGACGGCGCTTGAGACTCAGGCGGAGGAATTGAAT

GAAGAACTCAAAATCCAGGACAAGAAAATAGAAGACCTGACTGAAAAGGTGACGGCGCTTGAGACTCAGA

ATAAAGAACTGAAAGACAAAGTGGCGCTCTGGTCCAGCCAGCCCAAGACAAAAGTTTCAAAAGCCGTCCT

GACAGACCCTCTGCGGGACTACAGAGCATCCCCTTACCTGCTGCTGACCAGGATGAAGAAGCAGGACTGC

TAA

>Duck CFAP53

ATGACGGCGCGGAGGGGGCTGGGGTGGCGGCGGGAGGTGCTGGGGCCCTGCCCGCACTCTGTGGCCATGA

GAGCCAGGCCTCCTAAAGAGCGAAGAAATGACAATTTTGTCTTGGCCCGCAGAGGCAGAGAAGAGGAGCT

CCTGGAATACGCCGCTCTCCTCAAGCTCTACAACCGCTGCCGCAGCGTCCACGAGTGGCAGCAGCGCAAC

GAGCAGAAATGGCTGCACAGAGCCGTGCAGAGAAAGGTCGAGGCGGCGATGCAGGAGTGTCAGGCAGGGA

TCGATGGCAGAAGAGAGAGGCTTCGCGAGCTCCTGGAGGCCGAGGAAAATAAATACTTTGCTGAGATGGA

GGCGTTTGAAGAAACCCTGCTGGAGAAGCAAGCGAAAATGAGGGAGCGAGCGAAATTGTTGCGGGAGGAG

AGGGAGAAGGAAAGGCAGCAGCTGGTGGCTGAAAAACGGGAGCAGCAATTCAGAGAACAATGCGATGAGC

TCCGCACGCAGCGGATGAAGCAGCATCAGAGGGAATTGTGCACGGAGCGGCTGGCCCAGCTAACTCTGAA

GGAGGAACTGAAAAAACAGCGGGAGGAGGAGGAGGAGATGTTCGCAGAGCTTTGGAAGGAGGACAGGTTG

GCTAAGGAAAGGCGAGAGGCGGTGGATGTGCAGAAATTATCCAAACAGAATCGGGAAATCCTGGACGTCC

TCGGCGCCCAGGTAGCAGCGCTCAGCGCTCACAAAGAGGAGGAGAAGCGGCTGAAGGAAGAAGAGGCTCG

ATTGCTGGAAGAACAGCAGCAGCTGCTGAAACTTGAAAATGAAAAACTTCAAATGGAGAAATTACAGAAG

CAGAAGGAATGCAGGGAGATGTTGCTCGGCGCAGCAGAGGACAAGAGGAAGCGGCTTAATGAAGAAAAAG

AAGGCGAGCTTGCCCTCGAGATGAAAACCTTAGAAAAATCGCCTTGGAAACCTGAGGAAGACCCCGAGGA

GAAAACCAGGAGAAAACAAGAGCTGTTCAAGGAGCAACAGGCTTACCGGGCGCACCTGGCTCAGCAGCTG

GAGGAGGAAAAAGAGCGAGAAAAAGAAGTGGACAAGCTCCTCGAGGAAGAGAGGGCGAAGGTTTGGGCCA

AGAAGGCTGAGCAAACGCGGTTGGAAAAGGAGGCTAGAGAGCAGCTCCTGAAAAACGTCCTGGATACGAG

ACAGCTACAGATTGAGGAGAAGATGCAGAGAAACGCAAAGGAGCAGGAAGAGCTTGCTCAGGAGAAGAAG

TTACTAGCTGAGGCCGTCGCAGAACTCAAACGCATAGAGGAAGAAAAATATGCGAGAAAACTGAAGGAAG

CAAAAGAATACCAGGAGCAGCTCAGGGCTCAGATTGCCCATCAGCAACGGGCCCGTGATGCTGAGGAAGA

AGAGAAGAAGCAAGAACACGAGCTGGGCCTAGCAGAAGAGCGAGCTTACCAAGAAAGGATACAGGACGCT

CTGTCAAGGCCTTACGAGAAACTAGCAAAAATTCACCCTTTGAGAAGAAAACTCACGTCTAACTCCCAAG

ATCACTTAACTCTGACATTTTAG

>Duck CTIF

ATGGAAAGCTCGTCAGTGGCATCCGCCTCCTCAGAGGCAGGGAGCAGCCGCTCACAGGAGATCGAGGAGC

TGGAGCGCTTCATCGACAGCTACGTGCTGGAGTACCAGGTGCAGGGGCTGCTGACCAACAAGATGGAGGG

GGACAGGGAGAGTGAGAAGATGCAGTCCAACATCTTGCAGTGGACGGCAGATTGTAGTGAGCAGCTTGAT

GACCGCTGTTCCCCATCCAGAGGGAAGGGCTCATCGTCTCCTGAACACAATCAGAATGGCAACAAGGAGG

GCTCCCTTGACATGCTGGGCACTGACATCTGGGCTGCCAACACCTTCGACTCCTTCAGTGGTGCAACATG

GGACTTGCAGCCTGAAAAACTAGATTTCACGCAGTTTCACAGGAAGCTCAGAAACACCACCAAACATCCA

ATGCCTCACATAGACAGAGAAGGGATCGGAAAAGGGAAGTACGAGGACGGTGACAGCATCAACCTGAACG

ACATAGAGAAGGTCCTCCCGGTGTGGCAGGGTTACCATCCATTGCCTCATGAAGATGAAATCGCACACAC

CAAAAAGCTGTTCAGAAGGAGGAGAAACGACCGGCGGCGACAGCAGAGGCTCCCCGGAGGGAACAAGTCT

CAGCAGCACGCAGATCATCAGCAAGGCGGCACCAAACACAACAGGGACCACCAGAAGCTCTACCAAGGAG

GCCAGGCCCCGCACTCCTCGGGCAGGACGGGCCACCACGGCTACAGCCAGAACCGGAGATGGCACCACAA

CCAGAAGCACTCGCCCAACGACAAAGAAACGCACAGAAACGCCAAAGAGACTGAGAACCTGAAAATCGAG

GACAGCTCCGTCGGCACGGCCGAGACGCAGCGGGGCCCCGAGGCGGGGGAGAAGCAGTCCCAGCAGTACA

TCCAGGAGCCCGAGACCAAGAGGAAAGACAGTATTCACGAGCACATTGGGGAGAGACCCAAGATCAATTT

GCTTCAGTCTTCCAAAGACAGGCTGCGGAGGAGGCTAAAAGAAAAGGACAAAATCACAGTTGAAACTACC

AATCCTGAAAAGAACAAAATGGACAAATTAATTGAAATCCTTAACAGCATGAGGAACAACAGCAGTGATG

TTGACTCCAAGCTCACTACCTTCATGGAGGAGGCCCAGAACTCCACCAACTCTGAGGAGATGCTGGGGGA

GATAGTTAAGACCATCTACCAGAAAGCGGTGACAGACCGCAGCTTTGCTTCCACAGCAGCCAAGCTCTGT

GACAAAATGGCCCTCTTTATGGTGGAAGGAACCAAATTCCGGAGTCTGCTCCTCAACATGTTGCAGAAGG

ATTTCACCATGCGGGAGGAGCTGCAGCAGCGGGACGTGGAGCGCTGGCTGGGGTTCATCACCTTCCTCTG

TGAGGTCTTTGGCACCATGAGGAGCAGCACTGGAGAGCCCTTTCGAGTCCTCGTCTGCCCCATTTATACC

TGCCTCAGGGAGTTGTTGCAATCTCAGGACGTGAAGGAGGACGCCGTGCTCTGCTGCTCCATGGAGCTGC

AGAGCACCGGCCGGCTGCTGGAGGAGCAGCTGCCTGAGATGATGACGGAGATGCTGGCCATAGCTCGCGA

CAAGATGCTGTGCCCCTCCGAGTCCATGCTGACACGGTCCCTCCTGCTGGAGGTCATCGAGCTGCATGCC

AACAACTGGAACCCGCTGACACCTACCATCATGCAGTACTACAACAAGACAATCCAAAAACTGACAGCCT

GA

>Duck DCC

ATGGAGAGGAGGCTCGGCTGCGCCCGGGTACCCAAACTGGCTTCGCTCCTCTTCTCCTGCGCCCTGGCGA

GCCTCAAGCTTGGGGTTTTTGGTTCCCAGATTAAGCCTTTTACGTCGTTGAGGTTTGTAACGGAGCCGTC

AGATGCTGTCACCATGCGTGGAAGCAACGTGCTGTTGAACTGTGGGGCAGAATCTGACCGAGGAGCTCCG

GTCATCAAGTGGAAGAAAGACGCCGTCTTCTTGAACCTGGCGGTAGACGAAAGGAGGCAGCAGCTGGCCA

ACGGCTCGCTCTTGATACAAAACATAGTCCACTCCAGGCACCACAAGCCAGACGAGGGGCTTTATCAGTG

TGAAGCATCTCTGGAAGGCATCGGAGCCATCATCAGTCGGACAGCGAAGGTCATGGTAGCAGGACCGCTG

AGGTTTCTTTCCCAGACGGAATCGGTCACGGCTTTTGCGGGAGACACGATCCTGCTCAAGTGCGAAGTCG

TCGGGGAGCCCATGCCTGTGGTGCACTGGCAGCGAAACCAGGAGGATTTATTCCTGAGCCCGGCCGACGC

ACGGGTGGCCGTCTTGCCCTCTGGAGCTTTACAAATCAGCAGGATTCAGCCTGGGGACAGTGGGATCTAC

AGGTGCCTGGCGAAAAACCCAGCCAGTTCAAGGACCGGGAATGATGCAGAAGTCAGAGTGTTGTCAGATC

CAGGTCTGCACAGGCAACAATTTTTCCTGCAGCGCCCGTCCAACGTGATGGCCATGGAAGGGAAGGATGC

TGTTTTGGAGTGCTGTGTCTCTGGGTACCCCCCTCCCACCTTCACGTGGCTGCGAGGAGAGGAAGTGCTC

CCCATCAGGTCCAAAAAGTATTCATTACTGGCTGGCAGTAATTTACTCATATCAAACGTGACCGATGATG

ATTCAGGGACATACACATGCGTCGTCACCTACAAGAACGAGAACAGCAGCGGCTCTGCGGAACTGTCAGT

GATGGTTCCACCATGGTTTTTAGTTCGCCCATCGAATCTTTATGCCTACGAGAGCATGGACATCGAGTTT

GAATGCGCCGTGTCTGGCAAGCCCATCCCTACGGTGGAGTGGATCAAGAATGGAGAAGTGGTCATTCCTA

GCGACTATTTTCAGATAGTGGGTGGCAGCAACTTGCGGATTCTGGGCTTGGTAAAGTCAGATGAAGGATT

TTATCAGTGTGTAGCTGAAAATGAAGCTGGAAACGCACAGGCCAGTGCACAGCTAATCATCCCAGAGCCT

GCTGTCCTAAGCTCCAGTGTCCTCCCCTCTGCCCCCCGAGATGTGGTCCCTGTCTTGGTCTCCAGCCGAT

TTGTCCGTCTCAGCTGGCGCCCGCCCGCCGAAGCCCGAGGCAGCATCCAGGCGTACACGGTCTTCTTCTC

CAGGGAAGGCATCAACAGGGAACGGGCGGTCAACACGTCGCAGTCCGGGACTCTCCAGCTCACCGTGGGC

AACCTGCGACCGGAGGAGACCTACACCTTCCGCGTGGTGGCCTTCAACGAGTGGGGACCTGGCGAGAGCT

CGCAGCCCGTCAAGGTGGCCACGCAGCCCGAGTTGCAAGTGCCAGGGCCGGTGGAAAACCTGCGGGCTGT

GTCCACTTCACCTACCTCCATCCTCGTGTCCTGGGATCCCCCTGCCTACGCCAACGGCCCCGTGCAAGGC

TACAGGCTCTTCTGGACGGAGACAGCAACAGGAAGGGAGCAGAACGTGGAGGTGGAAGGGCTCTCCTACC

GGCTCGAGGGGCTGAAGAAGTTCACCGAGTACAGCCTGCGCTTCCTGGCCTACAACCGCTACGGCCCCGG

CGTCTCCACCGAGGACGTCACCGTCACCACGCTTTCGGATGTGCCCAGCGCCATGCCCCAGAACGTCTCC

TTGGAAGTGGTCAACTCCAGGAGTATCAAAGTTAGCTGGTTGCCTCCACCACCAGGCACTCAAAATGGAT

TTATTACGGGCTATAAAATCCGACACAGAAAGACTACCCGCAGGGGTGAGATTGAAACACTGGAGCCAAA

CAACCTCTGGTACTTGTTCACAGGACTGGAGAAAGGAAGCCAGTACAGTTTCCAGGTTGCTGCCATGACG

GTGAACGGGACCGGGCCGCCCTCGGACTGGTACACGGCAGAAACACCCGAGAATGATCTCGATGAATCTC

AGGTTCCTGACCAGCCAAGCTCTCTCCACGTCAGACCTTTGACCACGAGCATCGTCATGAGCTGGACCCC

GCCGCTCAACCCCAACATTGTCGTCCGCGGGTACATCATCGGCTACGGCGTGGGCAGCCCATACGCTGAG

ACCGTGCGGGTGGACAATAAACAACGTTACTATTCCATTGAAAATTTGGAGCCGAGCTCCCACTACGTGA

TCTCCTTGAAGGCCTTTAACAACGCCGGCGAAGGAGTGCCTCTGTACGAAAGTGCCACCACCAGGTCGAT

GACAGACCCCATCGATCCATTAGAAGTTGATTTTTATCCTTTGCTTGATGATTTCCCCACGTCAGTCCCA

GATATCTCCACCCCCATGCTCCCACCAGTAGGTGTGCAGGCTGTTGCGCTGACCCACGACGCGGTGAGGG

TCATCTGGGCAGACAACTCTGTCCCGAAGAACCAAAAGACGACGGAGGTCCGCTTCTACACGGTCCGATG

GAGAACCAGCTACTCTACAAATGCTAAATACAAGTCAGCAGATACGACAGCCCTAAGTCACACCGTCATC

GGCCTGAAGCCTAACACCATGTACGAGTTCTCGGTCATGGTCACCAAAGGGCGGAGGTCCAGCACCTGGA

GCATGACTGCCCACGCCACCACCTACGAAGCAGCTCCGACCTCTGCACCCAAGGACTTGACAGTCATTAC

ACGGGAAGGGAAGCCCCGAGCCGTCGTTGTCAGCTGGCAGCCACCATTAGAAGCCAATGGGAAAATTACC

GCTTACATCCTCTTCTATACCCTGGACAAGAATGCTCCCATCGATGACTGGGTGATGGAGTCCATCAGCG

GGGACCGGCTCACCCACCAGATCATGGATCTCAGCCTGGACACCGTCTACTACTTCCGAATCCAAGCCCG

CAACGCCAAGGGAGTGGGGCCTCTCTCCGATCCCGTTTTCTTCCGGACGCTGAAAGTTGAGCACCCCGAC

AAAATGGCCAATGACCAAGGTCGTCACGGCGATGGCTCCTACTGGTCGGTGGACACCAACCTGATTGACA

GAAGCAGCCTGAATGAGCCCCCCATCGGGCAGATGCACCCTCCCCACGGCAGCGTCACGCCCCAGAAGAA

CAGCAACCTCCTCGTCATCATCGTCGTCACCGTCGGCGTCATCACCGTGGTGGTGGTGGTGGTGGTGGCC

GTCATCTGCACCAGGCGCTCCTCGGCACAGCAGAGAAAGAAACGTGCGACCCACAGCGCCAGCAAGCGGA

AGGGCAGCCAGAAGGACCTGAGGCCCCCGGATCTGTGGATCCACCACGAGGAGATGGAGATGAAGAACAT

GGAGAAGCCGACGGGCTCCGACCCCGCGGGGAGGGACTCGCCCATGCAGAGCTGCCAGGACATCACCCCC

GTCAGCCACAGCCAGTCCGAAACGCAGCTGGGCACCAAGAGCACCCCGCAACCCGCTGCTGAGACAGAAG

ATGTTGGCAGCAGCATGTCCACCTTAGAGCGCTCGCTCGCCGCCCGCAGAGCCACCCGTGCCAAGCTCAT

GATCCCGATGGATTCCCAACCCACCAACCCCCCTGTGGTCAGTGCCATCCCTGTGCCCACCCTGGAAAGC

GCCCAGTACCCCGGGATCCTGCCGTCCCCCACCTGCGGATACCCACACCCCCAATTCACCCTGCGGCCGG

TGCCTTTCCCCACCCTGGCTGTGGACAGGACCTTTGGAGCAGGAAGGACTGTAAACGAAGGAGCAGCCCC

GCAGCAACCCTCCTTGCTACCACCGACGCAGCCCGAGCACTCCAGCAACGAGGACGCCCCCAGCAGGACC

ATCCCCACCGCCTGCGTGCGCCCCACGCACCCGCTCCGCAGCTTCGCCAACCCCTTGCTACCTCCGCCCA

TGAGTGCAATAGAACCGAAAGTCCCTTACACGCCACTTCTGTCTCAAACAGGGCCTAACCTTCCCAAGGC

TCAGGTTAAAACAGCATCCCTCGGCTTGGCAGGGAAAGCGAGGTCCCCTCTGCTGCCCGTCTCGGTACCC

ACAGCCCCGGAGGTTTCGGAGGAGGGCCACAAGCAGACGGAAGACTCCTCAAACGTTTACGAACAGGACG

ATCTGAGCGAACAGATGGCCAGTTTGGAGGGGCTGATGAAGCAGCTCAACGCTATCACGGGCTCAGCCTT

CTAG

>Duck DYM

ATGGGAGCAAATAGCAGCAGCATCAGCGAGCTTCCAGAAAACGAATACCTAAAGAAGTTGTCAGGAGCAG

AGCCCATCTCCGAGAATGACCCCTTCTGGAATCAACTGCTGTCTTTTAGCTTTACCACTCCAACAAACAG

TGCGGAATTAAAGCTCTTGGAAGAAGCCACCATCTCAGTCTGCAAGTCTTTAGTTGAGAAGAATCCTCGA

ACAGGAAACCTTGGGTCATTGATAAAAGTCTTTCTTTCTAGAACCAAAGAATTAAAAATATCAGCAGAAT

GTCAGAATCACCTCTTTATCTGGCAGGCTCACAATGCCTTATTTATCATCTGCTGCTTGCTCAAAGTGTT

CATCAGTAGAATGTCAGAAGAGGAGCTGCAACTTCATTTTACTTACGAGGAAAAAACACCAGGCTCATAT

GGAACCGAGTGTGAAGACCTCATAGAAGAGTTGCTGTGTTGCCTCATCCAGCTCATTGTTGAAATTCCCC

TCTTAGATATTACCTACAGCATTTCCTTGGAAGCTGTGACAACGCTCGTCGTCTTCCTCTCCTGCCAGTT

ATTTCACAAGGAAATTCTGCGAGAAAGTCTCATCCACAAATACCTGATGCATGGTCGATGTCTCCCGTAT

ACCAGCAGACTTGTGAAAACTCTGCTCTACAATTTTATTAGACAAGAAAGAAGCCCTCCTCCGGGGACCC

ACGTCTTTCAGCAGCAAACAGATGGAGGAGGACTGCTGTACGGAATTGCATCCGGGGTGGCAACTGGCCT

GCGGACAGTCTTCACGTTAGGTGGAGTGGGGAGTAAAGCAACGCCGCAGCTGGAGCAGTGCTCCCCTCTA

GCTCATCAGAGTCTGCTGCTTCTCCTGGTCTTAGCTAATCTGACTGATGCTCCGGATACACCGAACCCCT

ACAGACAAGCTATTATGTCCTTCAAGAACACCCAAGATAGCAGTGCTTTTTCGTCATCAAATCCGCACGC

TTTCCAGATCAATTTTAACAGCTTGTACACGGCTTTGTGTGAGCAGCAGAGATCTGATCAAGCGACCCTC

CTTTTGTACATGCTTCTGCATCAAAACGGCAACGTACGGACATACGTGTTGGCACGAACAGACATAGAAA

ATCTTGTTCTGCCAATTCTTGAGATCCTGTACCACGTTGAAGAAAGGAATTCACACCATGTTTACATGGC

TCTTATAATTCTGCTGATCCTTACCGAGGACGACGGCTTCAACCGATCCATTCACGAAGTGATACTGAAA

AATATCACTTGGTACGCTGAGCGTGTTTTAACAGAGATCTCACTTGGGAGCCTCCTGATACTCGTTGTGA

TAAGAACCATCCAGTACAACATGACACGGACAAGGGACAAATACCTTCATACAAATTGTCTGGCTGCCTT

AGCAAATATGTCAGCGCAGTTCCGCTCTCTTCATCAGTACGCAGCTCAGAGGATCATCAGTTTATTTTCT

TTGTTGTCTAAAAAGCACAACAAGGTGTTGGAGCAAGCCACGCAGTCCTTAAGAGGTTCCCTTGATTCAA

ATGACTCTCCGCTTCCTGATTATGCACAAGACCTGAATGTGATCGAGGAAGTGATCCGAATGATGTTAGA

GATTATCAACTCCTGCCTGACCAATTCCCTTCATCACAACCCAAACTTGGTGTACGCACTGCTTTACAAG

AGGGATCTGTTTGAGCAATTTCGAACTCACCCTTCCTTCCAGGACATAATGCAAAATATAGATCTGGTGA

TCAGCTTTTTCAGCTCCCGATTAAAGCAAGCTGAAGCTGAACTGTCAGTGGAGCGGGTTCTGGAAATCAT

CAAGCAGGGAGCTGTTGCTTTGCCCAAAGACAGGCTAAGAAAGTTCCCGGAGCTCAAGTTCAAGTACGTG

GAGGAGGAGCAGCCCGAGGAGTTCTTCATCCCCTACGTTTGGTCTCTGGTGTACAACTCCGCCGTGGCCC

TGTACTGGAACCCTCAGGCCATCCAGCTCTTCACTATGGACTCTGGCTGA

>Duck DYNAP

ATGGATAACCAGGCCTTTGAAATGCACGGGGAGAAAATACAAAGCTCTTCGAAAGGAAACGAGTGGCCCA

AGAAAGAGGAAAGAAAAGGCAACTGGTCGCTGATGAAAATCTTCCTGGTTTGCCTGCTGGCCTGCGTTAT

CACCACCGCCATCGGAGTGCTGGTCTTGTCTTTGGTCTACACAACCACCACCCACATCGTGAAAGAGGTC

AGCGTTACAGACGATGGGGCAACCTCCATGAAACCAGAGGACAAGAACGTGGACGTCAAGTTCCAGTTCC

TCAATCATCTGACAAAATCCAAGGTGTATAATTTCCCCGGTGGTGAAATTCAGTGGGCACGATTCAGGAA

TGATGTAAACGAGTATGAAAACGATGAAGAAATGGAATTTGGGAAAAGTATCAACAACCACCGCTCTTCC

ATGACTTTTGGCACCTTGAGGATCAAAGGCAAAGGGCTCCGGGTCCCCCACTGGCACTTCAATGCCAACG

AACATGGTTACCTGCAGCAGGGCACTGCCTGGATTGGAGTCATTGGGGCAGACGGCAGCGGGGTCACCAC

GTACAATGTCACGGCTGGCCAGGTGATCTTCTTCCCCAGAAACACCGTGCACTGGATAAAGAACGTAGGC

ACGGAGGACTGCGTGTTCTTGCTGTTTTTCACGACACACGAAGAGCTTCAGACCTTGGATGTAGACGATG

CATTCTTCTCTACCCCAGAGGATATAGCAGCAAGAGCTTTAAAGCCACAAGGTGGGGTCAACTTCATCAG

GACCTTCAAGAAACAAATGGAAGATCAAGCGGTCAACCTCCCTCCAAACCTGGACGAGCTCGTAAAAAGT

GCCGACTACGTGCAGTCCCCGGACCACCAGGTGTGGCAGTACTTCTACGACCTCAAAGGGTCCAGAGAAT

ATCCTTTCCCGGGAGGCATCTTCCAGTGGGCTCGCTACCGCAGAAATACCACCGGGCTGAATGAAACCGA

GAAAATCTTCAGTGAGTCACTGAACAAGCATGAAAATACCCTTACCTTGGCAACCCTCAGGATATTCAAT

AATCAGCTGGGACAGCCTCATTTCCACTTCAATGCTAATGAGATGGGTTATGTCATTAGCGGCTGCGGAC

AGGCTGGAGTTATCACCTCCTCTGGAGCCACCAGCAGCTTCAACATTGGCATCGGAGATGTTATATTCTT

CCCCGTTGGGACCCAGCATTACCTTAAAAGCATATGTGATGAAGACTTGCTTCTGATTCTAGCCTACAGC

ACAGGCGACCAGCTGGAAACTCTTCGCATGAACGACTACTTCCACGCCACAGCAGACCACATCCTTGCTC

AGATTTTTTTCAAGGAACAGGACGAGTTCAAGAAGTTCCCAAGGTCTAAAAAATAG

>Duck ELAC1

ATGTCGATGGATGTAACGTTCCTCGGGACGGGCTCAGCGTACCCCTCTCCAACGAGGGGGGCGTCCGCGT

TGGTGCTCCGCCGAGAAGGCGAGTGCTGGCTCTTCGACTGCGGGGAGGGGACGCAAACCCAGCTGATGAA

AAGCCACCTGAAAGCAGGCAGAATAACCAAGATTTTCATAACTCATCTCCACGGCGACCACTTTTTCGGG

CTTCCCGGCTTGCTGTGTACCCTCAGCCTCCAAAGCAGCCCTGACCCAAACAAACCACCCCTCGATATTT

ACGGCCCGATCGGACTGAGAAACTTCATCTGGAGGACCATGGAGCTCTCCCACTCCCAACTCGCGTTTCC

CTACGTTGTCCACGAGCTCGTCCCTACGCGGGACCAGTGCCCTGCAGAAGAATTTAAAGATTTTTCTTTG

TTGAGAGACGAGGTGCCTCCCCGGGAAGCCCAGGGGAGAATTCTCCACCTGGATCCCGGGGAAAACTCGT

ACTTGCTGGAGGACAACGAGCAGCTGGTGGTGAAAGCCTTTCGCCTCTTCCACCGCGTGCCTTCCTTCGG

CTTCGTGGTGGAAGAGAAGCCCCGGCCTGGGAAACTCAACGTAGAAAAACTGAAAGACCTCGGAGTTCAG

CCAGGTCCGGCGTACGGGAAACTGAAGAACGGAATTTCCCTCGTTCTGGAGGATGGCAGAACCATTTCTC

CTTCGGACGTCTTAGAAGAGCCGGTGCCCGGCCGAAAAATCTGTATTTTAGGGGATTGTTCGGGGCCGGT

TGGGGACGAAGCCACGGAGCTTTGCCGCGAAGCCGATTTGCTGATCCACGAAGCCACGCTGGACGACACC

CAAGAGGAGAAGGCCAAAGAGCACGGCCACAGCACCCCAAAAGCGGCGTCTGCTTTCGCCAAGCTGTGCG

GAGCTAAAAAATTGGTTTTGACTCATTTCAGCCAGCGCTACAAAGCTCGGCTCGGCGAGGGGGACGCCGA

TATCACCCAGCTGAAGGGGCAGGCGGAGGCGGTGTTGGATGGCCAAGAGGTCGCGTTGGCCGAGGACTTG

ATGACGCTCGAAATCCCCATGAAAAAGTCAATTTTTTTTGAAAAAAATGAGCATTAA

>Duck FECH

ATGNNNNNCACGAAGCCTCAGGTTCAGCCAGAAGCGCGGAAACCTAAAACGGGAATCTTGATGTTAAACA

TGGGAGGTCCGGAAAGGCTGGATGATGTGCATGACTTCTTACTTCGTCTCTTCCTGGACAGGGATCTAAT

GACGCTGCCAGCACAAAATAAGTTAGCACCGTTCATCGCTAAGCGCCGCACGCCGAGGATCCAGGAGCAG

TACAGCAGGATTGGAGGTGGGTCCCCGATCAAGAAGTGGACGGCAGTGCAGGGAGAAGGCATGGTGAAGC

TGCTGGATAGCATGTCTCCTCACACAGCGCCTCACAAGTACTACATCGGGTTCCGGTACGTGCACCCTCT

GACGGAAGAGGCGATCGAGGAGATGGAGCAGGACGGCATCGAGCGGGCCATCGCCTTCACGCAGTACCCG

CAGTACAGCTGCTCCACCACAGGAAGCAGTTTAAATGCGATTTATCGCTACTACAATAAAAAGGGGGAGA

AGCCAAAGATGAAGTGGAGTATAATTGACCGGTGGCCCACACACCCCCTGCTCATCCAGTGCTTTACCGA

CCACATACAGAAGGAACTGGACCTGTTTCCACCTGACAAAAGGAAAGATGTTGTCATCCTTTTCTCAGCT

CACTCGCTCCCCATGTCTGTAGTGAACCGTGGTGATCCATACCCACAAGAAGTGGGAGCTACTGTTCAGA

GAGTCATGGAGAAGCTGAACTACTCCAACCCCTACCGGCTGGTCTGGCAGTCCAAGGTTGGGCCAATGCC

TTGGCTTGGTCCGCAGACAGATGAGACCATTAAAGGCCTGTGTCAAAGAGGAAAGAAGAACATGTTGTTG

GTCCCAATAGCATTTACAAGCGACCACATTGAAACACTTTATGAACTGGATATTGAGTATGCCCAGGTTT

TAGCAAATGAATGTGGAGTTGAAAACATCAGAAGAGCAGAATCGCTGAATGGAAATCCACTGTTCTCTAA

GGCTCTGGCAGACTTGGTCTGTTCACACATCCAGTCGAACGAAGTCTGCTCGAAGCAGTTAACGCTCTGC

TGTCCGCTCTGTGTAAATCCTGTCTGCAGGGAGACAAAAGCTTTCTTCACTAATCAGCAGCTGTGA

>Duck LIPG

ATGTCGCCGACAGTATCGCCGACCCCTGTGTCGCCACGGGTGAGGTTCAGCCTCCACTCGGCCTCCGAGG

CCGAAGGCTGCCCGCTGGCCCTGGGGCAGGAGGGGAGCCTGAAGGAGTGCGGCTTCAACGCCACCGCCAA

GAGCTTCTTCATCATCCACGGCTGGACGATGAGCGGCATGTTCGAGACGTGGCTGGGCAGCCTGGTGGCC

GCCCTGCAGGAGCGGGAGAAGGAGGCCAACGTGGTGGTGGTGGACTGGCTCTCGCTGGCCCACCAGCTCT

ACACGGACGCGGTGAACAACACGCAGGTGGTGGGGAGAAGCATAGCCCGGCTGCTCGACTGGTTACAGGA

AAATCCTCTTTTTAAGCTTGAGAACGTCCACCTGATCGGGTACAGCCTGGGCGCACACGTCGCTGGCTTC

GCTGGCAACCACGTCCACGGGACGATTGGCAGAATTACAGGCTTGGATCCAGCCGGCCCCATGTTTGAAG

GAGTGGACCCCAGCAAGCGCCTCTCCCCTGACGATGCCAGCTTCGTGGACGTCCTGCACACCTACACGAG

GGAGACGCTGGGGGTGAGCATTGGGATCCAGATGCCCGTGGGCCACGTTGACATCTACCCCAACGGGGGA

GACTTCCAGCCCGGCTGCGGCTTAAGCGATGTCTTAGGAGCGATTGCCTACGGGACACTTGGTGAAGTGG

TTAAATGTGAGCACGAGCGGTCTGTGCACCTGTTTGTGGACTCCCTCGTGAACCAAGATAAACAAAGCTT

CGCGTTTCAATGTACTGATTCCAGTCGCTTCAAGAAGGGAATCTGCCTGAGCTGCCGGAAGAACCGCTGC

AGCGGCATCGGCTACAACGCCAGGAGAACGCGGAACAAAAGGAACAGCAAGATGTACTTAAAAACAAGAG

CTGACATGCCCTTCAAAGTCTACCATTACCAGATGAAAATGCACGTCTTCAGCTACAAAAACCTGGCAGA

GGCTGACCCCACGTTCTCTGTCACCCTGCATGGCACCAACGGAGACTCCGAGCCCCTCTCCTTGGAGATG

CTGGATCAAATCGGCCTCAATGCTACCAACACCTTCCTGGTCTATACAGAAGAGGACATGGGGGAGCTGT

TAAAAATCAAGCTCACCTGGGAGGGCACGTCGCAGTCCTGGTACGATCTGTGGAAGGAGCTGAAGAGTTA

CTGGTATCGGCCCGCCAAGGCTGCCCAGGAACTGCACATCAGGCGGATACGCGTGAAATCGGGGGAAACG

CAGCAGAGGTTTGCTTTCTGTGTAGAGGACTTCCAGCTGACCCGCATATCTCCTGGCAAAGAGCTCTGGT

TTGTCAAGTGCGCAGACGACTGGAAAAAAAGGCCTGTCTCAAACTCGCTCTGA

>Duck MAPK4

ATGGCCGAGAAGTGCGACTGCATCGCCAGCGTTNTCGGCTACGACCTGGGCTGCCGCTTCGTCAACTTCC

GCCCTTTGGGTTTCGGGGCCAACGGTTTGGTGCTGTCGGCCCTGGACAGCAGGAGCTGCCGTAAGGTGGC

GGTGGTGAAGAAAATCGCCATCAGCGACGCGCGGAGCTTGAAGCACGCCTTCCGCGAGGTGAAGATCATC

CGGCGGCTGGAGCACGACAACATCGTCAAGGTGTACGAGGTGCTGGGGCCGAAGGGGAGCAGCCTGCGGG

GGGATTTCTTCAAGTTCAACGTGGTCTACATCGTCCAGGAGTACATGGAGACGGACCTGGCTCGCCTGCT

GGAGCAGGGGAAGCTCGCCGAGGAGCACGCCAAGCTCTTCATGTACCAGCTCCTCCGCGGGCTCAAATAC

ATCCACTCGGCCAACGTGCTCCACCGAGACCTCAAGCCGGCCAACATTTTTATCAGCACCGAGGACCTGG

TGTTGAAGATCGGCGATTTCGGGCTCGCCAGGATCGTGGATCAGCATTACTCCCACAAGGGTTACCTTTC

GGAAGGCTTGGTCACCAAGTGGTACCGCTCGCCTCGCCTCCTCCTCTCGCCCAACGACTACACCAAAGCC

ATCGACATGTGGGCGGCCGGCTGCATCCTGGCGGAGATGCTGACGGGGAGGATGCTCTTTGCAGGGGGAC

ACGAGCTGGAGCAGATGCAGCTGATTTTGGAAACGATCCCCGTCGTCCACGAGGAGGACAAAGAGGAGCT

GCTCAAGGTGATGCCCAGCTTCATCAGCAGCACCTGGGAGGTGAGGAAGCCGCTGCGCCAGCTGCTCCCC

GAGGTGGACAGCCAAGCTATCGATTTCCTGGAGAAAATCCTGACCTTCAACCCCATGGATCGCTTAACGG

CCGAGATGGGGCTGCAGCACCCTTACATGAGCCCCTATTCCTGCCCCGAGGACGAGCCGGTGTCGCAGCA

GCCGTTCCGGATCGAGGACGAGATCGATGACATTTTACTGATGGAAGCCAACCAGAGCCAGATGGCGAAC

TGGGACAGGTACCACGTCAGCCTCTCGTCTGATTTGGACTGGCGGCACGATAAATATCACGAGATGGACG

AGGTTCAGCGGGACCCGCGGGCGGGGTCGGGATCCATCGCCGAGGAGGCGCAAGTGGATCCCCGGAAATA

TTCGCAGAGCAGCTCGGAGAGGTTCCTGGAGCTCTCTCACTCCTCCATGGACCGCGTGTTTGACGCCGAG

TGCGGCAAATCCTGCGATTACAAAGTGGGGTCGCCCTCTTACCTGGACAAATTGCTGTGGAGAGACAACA

AGCCCCACCACTACTCGGAACCCAAGCTGATTTTGGATTTATCCCACTGGAAACGAGCAGCCATCGCGCC

CGAAAGCGAGCTGTCCCTGGAGGAGGAACCCTCCAACCTCTTCCTGGAGATCGCCCAGTGGGTGAAGAGC

ACCCAAGTGGGGCTCGAATGTCCCGCGAGTCTTCCGGAGATTCGGGAGCCGTCTCCTCCTCCCCACCTCC

GCCAGGAATCCGAGGAGGTGAGCGCCGAAACCGACCCCGAGTTTAATTTGGACGTCTTCATCTCCAGGGC

GCTGAAACTTTGCACGAAACCCGAGGATCTGCCGGACAACAAGCTCAGCGACATCAACGGGGCCTGCATC

TCCGAGCACCCCAGCGAGATGGTGCAAAGCGAGGTGTACCAGAAGGAGCGCTGGTGA

>Duck MBD2

ATGGAGCGGCAGGGCAGGATGGACTGCCCTGCGCTGCCCCCCGGCTGGAAGAAGGAGGAGGTGATCCGCA

AATCGGGCCTCAGCGCCGGCAAGAGCGATGTCTACTACTTCAGTCCAAGTGGTAAGAAGTTCAGAAGCAA

GCCTCAGTTGGCAAGATACCTGGGAAACACTGTTGATCTCAGCAGTTTTGACTTCAGAACGGGAAAGATG

ATGCCCAGTAAATTGCAGAAGAACAAACAGAGACTAAGGAATGATTCTCTCAATCAAAATAAGGGAAAAC

CAGACTTGAATACGACTTTGCCCATCAGACAAACGGCATCAATTTTCAAGCAGCCCGTCACCAAAGTCAC

CAACCATCCTAGTAACAAAGTGAGATCCGATCCGCAGCGAGTGACGGAACAGCCACGGCAGCTTTTCTGG

GAGAAGAGGCTACAAGGCCTCAGCGCGTCGGATGTCAGCGAACAAATCATAAAATCCATGGAGCTCCCCA

AGGGTCTTCAAGGAGTTGGCCCAGGTAACAACGACGATACCTTGTTGTCGGCTGTTGCCAGCGCTTTGCA

CACCAGTTCGGCACCCATCACGGGCCAGCTCTCCGCAGCGGTGGAGAAGAACCCGGCCGTCTGGCTCAAC

ACATCTCAACCCCTCTGCAAAGCTTTCATAGTCACAGACGATGACATTAGAAAACAAGAAGAGCGGGTGC

AACAAGTGCGTAAGAAACTGGAGGAAGCACTAATGGCAGACATTCTGTCACGGGCGGCTGATACGACAAA

AGATCTAGAGGTAGAAATGGATAACGGAGATGAA

>Duck ME2

ATGCTCTCCCGGATNAGGGCGGCCGCCGTTCCCTGCTCGCTGGCGCGCCGCGGGGCGCACACGAAAGAAA

AAGGCAAACCCTTGATGTTAAACCCCCGGACAAACAAGGGGATGGCCTTTACGTTGCACGAACGGCAGAT

GCTCGGCTTGCAAGGGCTTCTTCCTCCCAAAATAGAGACCCAAGACATTCAAGCCTTACGCTTCCACAAG

AATTTGGCCAAAATGACGGACCCCTTGGAGAAGTATATCTACATAATGGGAATCCAGGAGAGGAACGAGA

AGTTATTCTACAGGGTTTTACAAGACGACATCGAGCGGTTAATGCCCATTGTGTATACACCAACAGTGGG

CCTCGCCTGTTCCCAGTACGGACACATCTTCAGGAGACCAAAAGGATTATTTATTTCTATCTCAGACAGA

GGTCACATCAGGTCCATTGTGAATAACTGGCCAGAGAACGACGTCAAGGCTGTTGTTGTCACTGATGGAG

AAAGGATCTTGGGTCTCGGAGACCTCGGGGTGTACGGGATGGGAATTCCCGTGGGGAAACTCTGCTTGTA

TACGGCCTGTGCAGGAATCCATCCAGATAAATGCCTGCCTGTGTGCATCGACGTTGGAACTGACAACACA

ACCCTCTTAAAAGATCCGTTTTACATGGGCCTGTACCAAAAGAGGGATCGCTCGCAGGTCTACGACGACC

TAATCGATGAGTTCATGGAGGCCATCACAGACAGGTATGGCCAGAACACCCTCATCCAATTCGAAGACTT

CGGAAACCACAACGCTTTTCGGTTTTTGAGAAAATACAGAGAGAAATACTGCACCTTCAACGATGACATC

CAAGGGACGGCTTCGGTGGCCTTGGCGGGACTGCTGGCAGCACAGAAAGCCACGGGCAAGCCGATTTCAG

AGCAGAAAGTGCTGTTCCTGGGAGCAGGAGAGGCTGCCCTGGGAATCGCGAACCTCATCGTCATGGCCAT

GATGGAAACTGGTGTTTCAGCAGAGGAAGCCTACAAGAGAATATGGATGTTTGACAAATACGGATTGCTG

GTTCAGGGCCGAGAACAAAAGGTGGATTCCAACCAGGAACCATTTACCCACCCGGCTCCGGAGCAGATCC

CAAAGACGTTTGTTGACGCGGTGAACGTGCTGCGACCTTCTGCTATCATCGGAGTCGCGGGAGCCGGGCG

CCTCTTCTCTCCCGACGTGATCAAGGCCATGGGCTCCATCAACGAGCGGCCCATCATATTTGCCCTGAGC

AACCCCACGGTGAAGGCCGAGTGCACGGCCGAGGAAGCGTATACGTTAACAGAGGGACGCTGCTTGTTCG

CCAGCGGCAGCCCCTTCGACCTGGTGACCCTGGAAGATGGAAGAACCTTCAAACCGGGCCAAGGAAACAA

CGCTTACATTTTCCCAGGCGTGGCTCTCGCTGTCATCCTCAGCAGCGTTCGACATATTAGTGATAAGGTT

TTCCTGGAGGCCGCCAAGGCGCTGACGGAGCAGCTGACTGACGAGGAGCTCGCCCAGGGAAGGCTTTACC

CTCCGCTGTCCAATATCAGGGAAGTTTCTATTTACATCGCCGTCAAGGTGATGGAGTTTTTGTACGCCAA

CAACATGGCTTTCCATTACCCCGAGCCTGCGGACAAGAACCGGTACATTCGATCCAAAGTCTGGACCTAC

GAATACGAATCCTTCATGCCAGACGTGTACGACTGGCCGGAGTCTAAGAGCCCTCCCCACGCTAA

>Duck MEX3C

ATGNNNNGTTGCAAAATCAAAGCGCTGAGGGCCAAGACGAACACGTACATCAAGACCCCCGTTCGCGGCG

AAGAACCCATCTTCGTCGTCACCGGGCGGAAAGAGGACGTAGCCATGGCCAAAAGGGAAATCCTCTCGGC

CGCCGAGCACTTCTCCATGATCAGAGCGTCGCGGAACAAGAACGGCCCTGCTTTGGGAGGTTTGCCCTGT

ACCCCCAACCTGCCCGGGCAGACCACGGTCCAGGTCAGGGTGCCTTATCGCGTAGTCGGGCTGGTGGTGG

GCCCGAAAGGAGCTACCATCAAAAGGATCCAGCAGCAGACCCACACCTACATCGTCACCCCCAGCCGGGA

CAAGGAACCCGTCTTCGAAGTGACGGGGATGCCGGAGAACGTGGACCGGGCCCGCGAGGAGATCGAGATG

CACATCGCCATGCGCACGGGCAACTACATCGAGCTGAACGAGGAGAACGATTTCCACTACAACGGGACCG

ACGTCAGCTTCGAGGGAGGCGCCTTGGGGTCCGCCTGGCTCGCTTCCAACCCCGTCCCCCCCAGCCGCGC

CAGGATGATCTCCAATTACAGGAACGACAGCTCCAGCTCTTTGGGAAGCGGCTCCACCGATTCCTATTTC

GGAAGCAATAGATTGGCTGACTTCAGCCCCACCAGTCCCTTCAGCACGGGCAACTTTTGGTTCGGAGAAA

CGCTGCCTTCCGTGGGCGCCGAAGAGCTCGGGGCCGACTCCCGCCCCGCCTACGACTCCTTCCCGCCGCC

GTCCCAAACCATTTGGAGCCCTTTCGAACCCGTCAACCCCCTCGCCGGCTTCGCCAACGACCCCACGGGG

AACGCCAAGCCCCAGCGCCGCGGGAGCCAGCCCTCCACCCCTCGCCTGTCGCCCACTTTGGCGGAAAGCC

TGGAGCACCCCTTGGCGAGGAGGGCGAGGAGCGATCCCCCCGACGCCGCTCTTCCCGTCTACATCCCGGC

TTTTTCCAACGGTACCAACAGCTACTCCTCTTCCAACGGGGGCTCCACCTCCAGCTCGCCCCCGGAGTCG

AGGCGCAAGCACGACTGCGTCATCTGCTTCGAGAGCGAAGTGGTGGCCGCCCTGGTCCCCTGCGGCCACA

ACCTCTTCTGCATGGAGTGTGCCAACAAGATCTGCGAGAAGGAAACGCCCTCGTGTCCCGTTTGCCAGAC

AGCTGTTACTCAGGCAATCCAAATCCACTCTTAA

>Duck MYO5B

ATGTCCGCAGCTCAGCTCTACACCAAGTACACCAGGGTTTGGATTCCTGACCCCGACGAAGTTTGGAAAT

CGGCCGAAATTATCAAGGATTACAAAGAGGGAGATAAAAGCCTCCAGCTGAAGCTTGAAGATGAAACTCT

CTACGAGTACCCCATTGACCTCCAAGGCAATGAGCTGCCTTTCCTCCGCAACCCCGATATCCTGGTGGGA

GAGAATGACCTGACGGCCCTGAGCTACCTGCACGAGCCTGCGGTCCTCCACAACCTCAAAGTCAGGTTCC

TCGAGTCCAACCACATCTACACGTACTGTGGCATTGTCCTCGTGGCCATCAATCCCTACGAGCAGCTGCC

GATCTACGAGCAAGATGTCATCTACGCCTACAGCGGCCAGAACATGGGGGACATGGATCCCCACATCTTT

GCGGTGGCAGAGGAAGCCTATAAGCAGATGGCCAGGGACGAGAAGAACCAGTCCATCATCGTGAGCGGGG

AGTCGGGTGCTGGAAAGACGGTCTCTGCCAAATATGCCATGCGCTTCTTCGCGACCGTCGGGGGCTCTGC

CAGCGAGACCAACATCGAAGCCAAAGTCCTCGCGTCAAGCCCAATTATGGAGGCAATCGGGAATGCGAAA

ACAACGAGGAACGACAACAGCAGTCGCTTTGGGAAGTACATTCAGATCGGCTTCGATAAAAGGTACCACA

TCATCGGTGCCAACATGAGGACGTACCTGCTGGAAAAATCCAGAGTCGTGTTCCAGGCGGAGGACGAGCG

CAACTACCACATCTTCTATCAGCTTTGTGCCTCGGCGAGTCTCCCAGAGTTCAAAGACCTTGGACTCACC

TGTGCTGAAGACTTTTTCTACACTTCTCAGGGAGGCAGCACGTCTATCGACGGCGTGGATGATGCTGATG

ACTTTGAGAAAACCAGGCACGCCTTCACCCTGCTCGGAGTGAAGGAGTCTCACCAGATGGCCATTTTTCG

GATAATCGCTGCCATCCTGCACCTAGGAAATTTGGAAATTGAGTCGGAGCGGGACGGCGAGGCCTGTAGC

ATGTCGAGCGAAGACGAGCACTTGAACCACTTCTGCGGCCTGCTGGGCGTGGAGCAGGGCCAGATGCAGC

ACTGGCTTTGCCACCGCAAGCTGGTCACCACGGCCGAGACCTACGTGAAGAGCATGTCGGTGCAGCAGGT

GGTCAACGCCAGGAACGCCCTGGCCAAGCACATCTACGCCCAGCTCTTCGGCTGGATCGTGCAGCACATC

AACAAGGCCCTGCACACCACCGTCAAGCAGCACTCCTTCATCGGCGTGCTCGACATCTATGGGTTTGAAA

CTTTTGAAGTGAATAGCTTCGAACAGTTCTGTATCAACTACGCCAACGAGAAGCTCCAGCAGCAGTTCAA

CTCGCACGTGTTCAAGCTGGAGCAAGAGGAGTACATGAAGGAGGGAATCCCTTGGACTCTCATCGACTTC

TACGATAACCAGCCCTGCATAGACCTCATAGAGGCAAAGCTTGGGATCTTGGACCTGCTGGATGAAGAGT

GCAAGGTTCCCAAAGGCACCGACCAGAACTGGGCGCAGAAGCTGTACGACCGGCACGGGGGCAGCCAACA

CTTCCAGAAGCCCCGCATGTCCAACATCTCCTTCATCGTCCTGCACTTTGCAGATAAGGTGGAGTACCAG

TGTGAGGGATTTCTGGAAAAAAACAGGGACACGGTGTACGAGGAGCAGATCAACATCCTGAAAGCCAGCA

AGTACCAGATGGTAGCAGACTTGTTCCAAGACGAGAAGGACGCCGCGTCCGCCACCTCCGTGGGGAAGGG

GACGTCCAAAATCAACGTCCGTTCGGCTAGGCCAGTGATCAAAGCTGCCAATAAGGAGCACAAGAAGACG

GTGGGGCACCAGTTCCGCAACTCGCTGCACTTGCTCATGGAGACCCTGAACGCCACCACCCCGCACTACG

TGCGCTGCATCAAGCCGAACGACGAGAAGCTCCCGTTCAAGTTTGACCCAAAGAGAGCAGTGCAGCAGCT

GAGAGCTTGCGGCGTGCTGGAGACCATCCGCATCAGCGCCGCTGGCTTCCCCTCCAGGTGGACCTATCAC

GACTTTTTCAATAGGTACCGTGTGCTAATGAAGAAGAGAGACCTCTCAAAGAATGACAAGAAGCAGATCT

GTCAGACCCTGCTGGAAGACCTCATTAAGGATCCAGACAAGTTCCAGTTTGGCCGTACCAAGATCTTTTT

CCGTGCAGGCCAGGTGGCATATCTGGAGAAACTGCGAGCGGATAAGTTCCGAGCTGCCACCATCATGATT

CAGAAGACGGTGAGGGGCTGGCTGCAGAGGATCAAGTACCAAAGGCTGAGACGGGCTGCAATAGTGATCC

AGCGCTACGCACGTGGGCACCTGGCGCGGAGGCTTGCGGAGCACCTGAGGAGGACGAGAGCTGCCATCAC

CTTGCAGAAGCAATACCGAATGCTGCGCATCCGCCGAGCTTTCCAGAGGGTCCGCAAGGCGACCGTCACC

ATCCAGGCTTTTGCTCGGGGCATGTTTGTCAGGAGGATTTATCGCCAGGTCCTCGCGGAGCACAAAGCCA

CCATCCTCCAGAAATACGCCCGCGGCTGGCTGGCCCGCACCCGTTTCCGACGGATCCGAGGTGCCACCAT

CGTCCTGCAGTGTTATTACCGGCGTAGGAAGGCCAGGCAGCAGCTGAAGGCGCTGAAGATCGAGGCCCGC

TCGGCGCAGCACCTGAAGAAGCTCAACATTGGCATGGAGAACAAAGTGGTCCAGCTTCAGAGGAAGATCG

ACGAGCAGAACAAGGAATACAAACTTCTGAACGAGCAGCTCTCTACCCTCACGTCTGCCCACTCCTCTGA

GGTGGAGAAGCTGAAGAAGGAGCTGGAGCAGTACCAGCAGAGCCAGCAGGGTGATGGCAACCAGCTCGTC

AGCCTGCGGGAGGAGATGGAGCACCTCCGGCTGGAGCTGGAAAAAGCCCACGGCGAGAGGAAGGTGGTGG

AAGACAGCTACGTGAAGGAGAAAGACCTGCTCAGAAAGCGTATCTCCGACCTGGAAGAAGAAAACGCCCT

CCTGAAGCAGGAGAAAGAGGAGCTTAACAGCAGAATCCTCTGCCAGTCGGAAGATGAATTTGCACGAAGC

ACAGCTGAGGAAAACATCCAGATGAAGAAGGAGCTGGAGGAGGAGAGGTCTCGGTACCAGAACCTGATAA

AAGAGTATTCGAGGCTGGAGCAGAGATACGACAACTTGAGGGATGAAATGACCATTATAAAGCAAGCACC

GGGGCACAGGAGGAACCCATCCAACCAGAGCAGCTTGGAGTCTGACTCTAATTATCCATCGATCTCCACC

TCTGAGATAGGAGACACCGAGGATGTGATTCAGCAGGTGGAGGAGGTTGGGACGGATAAAGCAGCCATGG

ACATGAGCCTCTTCCTAAAGCTACAGAAGCGAGTGAGGGAGCTCGAGCAGGAGAGGAAGAAGCTGCAAAC

CCAGCTGGAGAAGAAGGAGCAAGAAAGCAAGAAAGCCCAGGTAATCGAGACGAAGACCGAAATGGCTTCG

GACCATGAAGATTTTGCCTACAACAGTCTGAAGAGGCAAGAGCTGGAGTCGGAGAACAAGAAGCTGAAAA

ACGAACTTAACGAGCTGAGGAAAGCTATCGCAGACCGAGCAACCCAGAACAACTCTTCCAACGATGTTCA

GGACAGTTATAATCTCTTGCTGAATCAGCTGAAGTCGGCCAACGAGGAGCTGGAAGTGCGGAAGGAAGAA

GTGCTCATCCTGAGGTCGCAGATAATGAAGGCGGCCCAGCAGAAAGAGCCGGGCAAAAATACGGAGCCCA

TCACCAGCCCTGCCAGCTGGCCCAACAGTGACAAGCACATCGACCAGGAGGACGTGATCGAGGCCTACCA

GGGGATATGCGAGACGAACCGCAAGACTGAGGACTGGGGTTATCTCAATGAAGATGGAGAGCTCGGCTTG

GCTTATCAAGGTTTAAAGCAAGTTGCCAGGTTGCTGGAAGCACAGCTCCAGGATCAGAGAAGAGAGCACG

AGGAGGAGGTGGAAGCTCTGCAAAACCAGGTGGAGGCGATGAAAGAAGAGATGGAGAAACAGCAGCAGGC

TTTCCTGCAGACCCTGCAGCTCTCTCCAGAGGCTCAGGTGGAGTTTGGACTCCAGCAGGAAATCACACGG

CTCACCAACGAAAACCTGGATCTTAAAGAACTGCTGGAGAAGCTGGAAAAGAATGAGAAGAAGCTGAAGA

AGCAGCTGAAGATTTACATGAAGAAGGTCCAAGATTTTGAAGCATACCAAGCCATGGTCCCGGAGGAGAG

GAGACAACACGAGCGTAACAGGCAGGTTGCTGTCCAGAGGAAGGAGAAAGATTTCCAGGGGATGCTGGAA

TATTACAAGGAAGATGAGCCGCTCCTCATCCGAAACCTCATCACAGATCTCAAGCCCCAAGCAGTGGCTG

CTACTGTTCCCTGCCTTCCTGCCTACATCCTCTACATGTGCATCAGGCACGCGGATTACATCAACGACGA

CCAGAAAGTGCACTCCTTGCTCACCTCCACCATCAATGGCATCAAGAAGGTGCTGAAGAAACACAACGAT

GACTTTGAGATGACGTCCTTTTGGTTGGCGAATACGTGTCGCCTCCTGCACTGTTTGAAGCAGTACAGCG

GAGATGCGGGTTTTATGACGCAGAACACGCCGAAGCAGAACGAGCACTGCCTGAAAAACTTCGACCTGAC

CGAGTACCGCCAGGTGCTGAGCCATCTCTCCACCCAGATCTACCAGCAGCTCATCAAGGTGGCGGAGGGC

ATCCTGCAACCCATGATCGTGTCTGGGGTGCTGGAATATGAGAGCATCCAAGGGCTTTCGGGAGTGAAAC

CGATGGGCTACCGCAACCGCTCGTCCAGCATGGAAGACGGCGACAACTCCTACAGCTTAGAGGCGATCGT

TCGCCAGCTCAACACCTTCCACACCACCGTGTGTGAGCAGGGCCTGGACCCCGAGATCATCCAGCAGGTC

TTCAAGCAGCTCTTCTACATGATCAACGCCGTTGCCTTGAACAACCTCCTGCTGCGGAAGGACGTCTGCT

CCTGGAGCACGGGCATGCAGCTAAGGTTTAACATCAGCCAGCTGGAGGAGTGGCTGCGTAGCAAGAACCT

GCAGCAGAGCGGAGCCCTCCAGACTCTGGAGCCCTTGATTCAGGCCGCCCAGCTCCTGCAGCTGAAAAAG

AAAACGGCGGAGGACGCCGAGGCCATCTGCTCCCTGTGCACAGCGCTCACGACGCAGCAGATTGTGAAGA

TCCTTAATCTCTACACTCCTGTGAACGAGTTTGAAGAACGGGTGACAGTAGCTTTCATACGAGACATACA

GACACATTTGCAAGAGCGAAACGACCCCCCGCAGCTGCTGCTAGACTTCAAGCACATGTTCCCCGTTTCG

TTCCCCTTCAACCCTTCCTCCATAACGATGGACACCATTCATATTCCGGCTTCTCTCGACTTGGAGTTTC

TCAATAAAGTCTGA

>Duck NARS

ATGTTATCTACCTCCTTGCCTAGAAGTTATGTCCAAAGTCTTACAGAGGAGCTGTATGTGTCCGAACGAG

AGGGCAGTGATTCCACCGGCGATGGGACACAGAAGAAACCGTTCAAAACTGTCTTAAAGGCTTTGATGAC

AGCAGGAAAGGAACCGTTTCCTACTATTTACGTGGATTCACAAAAGGAAAATGAGAGATGGGCCATTATT

TCAAAGTCACAGATGAAAAATGTCAAAAAACTGTGGCACAGGGAACAAATGAAGAATGAGGCTAAGGAGA

AGAAAGAGGCAGAAGATCTCTTGAGAAGAGAGAAGAACCTGGAGGAAGCTAAGAAAGTTGTTATCAAGAA

TGATCCCAGTCTTCCAGAGCCAAAATGTGTAAAGATCTGTGCTCTGGAGGCTTATAGAGGCAAGAGAGTA

AAGATTTTTGGCTGGATTCACAGGTTACGTAGGCAAGGAAAAAATCTCATGTTCATTGTTTTGAGAGATG

GCACAGGTTTTCTTCAGTGTGTCCTTTCAGATGAACTGTGTCAGTGTTACAACGGGCTAATTCTCTCCAC

AGAGAGCAGTGTTGCAGTGTATGGTGTGCTGAACCTTGTTCCTGAAGGCAAGCAGGCTCCAGGAGGCCAC

GAGCTGAACTGCGATTACTGGGAGCTTATAGGTCTGGCCCCAGCAGGAGGGGCTGACAATCTCCTCAATG

AGGATTCGGAGGTTGACGTGCAGCTTAACAACAGGCACATGATGATTCGAGGCGAGAATATGTCCAAAAT

CTTCAAGGTGCGCTCCATGGTAGTACAGGCCTTCAGGGATCACTTCTTTGCCAATGGATATTATGAAGTC

ACACCGCCGACTTTAGTCCAGACGCAGGTGGAAGGAGGTTCAACCTTATTCAAGCTGGATTATTTTGGAG

AAGAGGCATACCTAACACAGTCGTCCCAGCTCTATCTGGAGACCTGCATTCCAGCATTAGGAGATGTTTT

CTGTGTTGCTCAGTCATACAGAGCTGAGCAATCCAGGACACGCAGACACTTGGCAGAGTACACTCATATT

GAAGCTGAATGTCCCTTTATAACTTTTGAGGATTTGTTGGACCGTCTGGAGAACTTGGTGTGTGATGTAG

TTGACAGAGTCTTGGCGTCACCTGCATCAAGCTTACTGTTGGAGCTAAACCCGGGCTTCAAGCCTCCCAA

ACGTCCTTTCCGAAGAATGAACTACGCTGAAGCAATTGAGTGGTTAAAGGAACATGATGTGAAGAAGGAA

GATGGTACTTATTACGAGTTTGGGGAGGATATTCCTGAAGCTCCTGAGAGACTGATGACAGACTCCATTA

ATGAGCCAATCTTGTTGTGCCGATTTCCTGCAGAGATAAAGTCTTTCTATATGCAGCGCTGTCACGACGA

TTCCCGTCTTACTGAATCTGTTGATGTGTTGATGCCTAATGTTGGTGAAATTGTTGGAGGCTCCATGCGT

ATTTGGGACAGTGAGGAGCTACTGGAAGGCTATAAGAGAGAGGGCATTGATCCCACGCCGTATTACTGGT

ACACCGATCAGAGAAAATATGGTACCTGTCCTCACGGTGGATATGGTTTGGGATTAGAGCGGTTCCTGAC

CTGGATTCTGAATAGGCACCATATCCGAGATGTCTGTCTCTATCCACGCTTTGTCCAGCGCTGCAAACCA

TAG

>Duck NEO1

ATGGAGAATGAGACCAAAGGAATTTTTAGCTTTATAATAGTGTCTGACAGTGGTCAGTGGTGGATGCCTA

GGCGGGGATCCGTTGTGAGGACCTTCACTCCGTTTCACTTTTTGGTGGAGCCAATGGACGTTCTGTCAGT

TCGTGGGGCGTCTGTTATAATGAACTGTTCAGCTTATTGTGAAACCTCCCCAAAAATTGAGTGGAAAAAA

GATGGGACTTTGCTGAACTTGGTGTCAGATGACCGTCGCCAGTTGCTGCCAGATGGGTCTTTATTAATAA

ACAGCGTGGTGCATTCCAAGCATAATAAACCTGATGAAGGATATTATCAGTGTGTGGCAACTGTAGAAAG

CCTGGGGACCATTGTCAGCAGAACAGCAAAGCTCACGGTAGCAGGTCTTCCCAGGTTCACCAGTCAGCCA

GAATCATCGTCTGTCTACAAAGGAAACAGTGCAATCCTAAACTGTGAAGTCAATGTTGACCTTGCACCAT

TTGTGAGGTGGGAGCAGGATCGTCAGCCAGTCTTTCTGGATGATCGTGTGTTTAAATTACCAAGTGGAGC

TCTTATTATTAGTAATGCTACTGATATGGATGGAGGAGTCTATCGCTGCATCATTGAAAGTGGTGGAACC

CCTAAATACAGTGATGAAGCGGAGCTCAAAATTCTTCCAGATCCAGAGGAGCCACAGAGCTTGGTCTTTG

CAAGGCAGCCATCATCGCTTACAAAAGTTACTGGGCAAAGTGCGGTTTTCCCATGTGTTGCTGTAGGATT

TCCAACTCCGTATGTCAGGTGGACGAGAAATGAAGAAGAGCTTACTACAGAAGGCTCCGAAAGGTTTCTG

TTTCTTGCGGGAGGCAGTCTGGTGATCAGTGAAGTTATGGAAGAGGATGTGGGGATGTATACCTGCATAG

CAGATAATGGAAACGAGACGATTGAAGCTCAAGCAGAACTTGCAGTGCAAGTTCCTCCTGAATTTCTGAA

GCGGCCAGCGAACATTTATGCTCATGAATCTATGGACATTGTCTTTGAGTGTGAGGTGACTGGAAAACCT

ACTCCAACTGTGAAATGGGTCAAGAATGGAGACATGGTGATTCCAAGTGACTACTTCAAAATTGTTAAAG

AACATAATCTGCAAGTTCTGGGTCTGGTGAAATCAGATGAAGGATTCTATCAATGCATTGCAGAAAATGA

TGTTGGAAATGCACAGGCTGGAGCCCAGCTGATAATACTTGACCTTGATGTTGCCATCCCAACATTACCT

CCCACTTCACTGACCAGTGCCACTAATGACCATCTAGCACCAGCTACAACGGGACCACTGCCTTCAGCCC

CTCGGGATGTTGTGGCCACCCTCGTCTCCACTCGCTTCATCAGGCTGACGTGGCGGACACCTGTGTCAGA

CCCTCAGGGAGACAACCTCACCTACTCAATATTCTACACCAAGGAAGGTATAAACAGGGAACGTGTTGAA

AATACGAGTCGTCCTGGAGAGACACAAGTGATGATCCAGAACCTGATGCCAGAAACGGTTTATGTCTTCA

GAGTTGTGGCTCAAAACAAGCATGGCCCTGGAGAGAGCTCAGCACCGCTGAAGGTGGCCACTCAGCCTGA

GGTTCAGCTTCCTGGTCCAGCACCCAACATCCGAGCATACGCCAATTCACCCACCTCCGTTACTGTCACA

TGGGAAACGCCGTTGTCTGGCAATGGAGAAATCCAGAACTACAAGCTGTATTACATGGAGAAGGGACAGG

ACAGTGAGCAGGATGTTGATGTAGCAGGACTCTCCTACACCATTAATGGATTGAAGAAATACACAGAGTA

TAGTTTCCGAGTGGTAGCTTACAATAAACATGGCCCTGGCGTCTCTACGCAAGATGTTGTTGTACGTACG

CTGTCAGATGTCCCCAGTGCTCCACCACAGAATCTAACGCTGGAGGTGCGGAATTCCAAGAGCATCATGC

TACACTGGCAGCCTCCTCCTGCAGGGACACACAGTGGGCAAATCACAGGCTACAAAATTCGCTACCGAAA

AGTGTCCCGTAAGAGTGATGTAACTGAGAGCATCGGTGGGACCCAGCTTTTTCAGCTAATTGAAGGTCTT

GAACGAGGTACAGAATACAGCTTCCGAGTAGCTGCCTTGACTGTCAACGGTACTGGACCAGCTACTGACT

GGGTATCAGCAGAAACATTTGAGAGTGATCTAGATGAAACTCGTGTTCCTGAAGTTCCAAGCTCCTTACA

TGTCCGTCCACTTGTCACCAGTATCGTGGTGAGCTGGACTCCACCTGAAAACCAGAACATCGTGGTAAGA

GGCTATGCTATAGGGTATGGCATTGGCAGTCCTCATGCACAGACCATCAAGGTGGACTATAAACAAAGAT

ACTACACCATTGAAAACTTAGACCCGAGCTCCCACTATGTTATAACTCTGAAAGCGTTCAACAACGTTGG

TGAAGGAATTCCTCTCTATGAGAGCGCGGTGACCAGGCCTCATTCAGACACTTCCGAAGTTGATTTGTTT

GTTATTAATGCTCCATACACTCCAGTGCCAGATCCGTCTCCCATGATGCCACCGGTGGGAGTTCAGGCTT

CCATTCTGAGTCATGACACCATAAGGATCACTTGGGCAGACAACTCTCTGCCAAAGAACCAGAAGATCAC

TGATGCTCGCTACTACACAGTTCGCTGGAAAACCAATATTCCTGCAAATACAAAGTACAAGACTGCAAAT

GCAACCACTTTGAGCTATTTAGTGACTGGGTTAAAACCAAATACCTTGTATGAATTCTCTGTGATGGTGA

CTAAAGGTCGAAGATCAAGTACTTGGAGTATGACAGCACATGGAACAACTTTTGAATTAGTTCCTACTTC

TCCTCCCAAAGATGTGACTGTGGTGAGCAAAGAGGGAAAACCTCGGACAATAATTGTTAACTGGCAGCCT

CCGTCCGAAGCCAATGGCAAAATTACAGGGTACATCATTTACTACAGTACGGATGTGAATGCTGAAATAC

ACGACTGGGTTATTGAACCCGTTGTGGGAAACAGACTGACGCATCAGATACAAGAATTGACCCTCGATAC

ACCATATTATTTCAAAATTCAGGCCCGCAACTCAAAGGGCATGGGGCCTATGTCTGAGGCAGTTCAGTTC

AGAACCCCAAAAGCTGAATCCTCAGATAAAATGCCTAATGATCAAGCTTCAGGATCTGCAGGAAAGGGAA

GCCGCCCAGTGGACATAGGGCCAGATTACAAACCACCACTCGGTGGCAGTAACAGTCCCCATGGAAGCCC

TACTTCTCCCTTGGATAGCAACATGCTCCTTGTGATCATCGTATCTGTTGGAGTCATCACCATTGTGATC

GTGGTGATAGTTGCTGTCTTCTGCACTCGTCGTACCACTTCTCACCAGAAAAAGAAACGAGCTGCCTGCA

AATCAGTGAACGGGTCCCACAAGTACAAAGGAAACTCCAAAGATGTCAAGCCTCCTGACCTTTGGATCCA

TCATGAAAGACTGGAGCTAAAACCCATTGATAAATCTCCAGATCCCAATCCAATCATGACAGATACCCCA

ATCCCTCGCAACTCCCAAGACATCACCCCAGTTGATAATTCCATGGACAGCAATATCCATCAAAGACGAA

ATTCCTACAGAGGGCATGAGTCAGAGGATAGCATGTCCACGCTGGCAGGAAGAAGGGGAATGAGGCCCAA

GATGATGATGCCTTTTGATTCTCAGCCACCTCAGCCTGTGATTAGTGCTCATCCCATCCATTCACTCGAT

AACCCTCACCATCATTTCCACTCCGGCAGCCTCGCTTCTCCAACTCGCAGCTATCTCCATCACCAGGTCA

ACCCGTGGCCGATTGGCACATCCATGTCCCATTCAGACAGGGCCAATTCCACAGAATCTGTTCGAAACAC

ACCTAGCACGGACACCATGCCGGCTTCCTCCTCTCAGACATGTGCTGACCATCAGGATCCCGAAAGCACG

ACAGGAGCTTACCTGGCTAATGCACAAGAGGAGGATTCAGCTCAGAACCTCCCCACGGCACATGTTCGTC

CTTCCCACCCGCTGAAGAGTTTTGCAGTGCCAGCAGTCCCACCAGCTGGTTCCACATACGACCCTGCATT

GCCAAGCACCCCGTTACTGACTCAGCAAGCTCCTAACCATCCCGTTCACTCGGTGAAGACTGCGTCAATT

GGGACCTTAGGAAGAACTCGACCTCCTATGCCAGTGGTAGTTCCCAGTGCCCCTGACGTTCAGGAGACCA

CCAGGATGCTCGAGGACTCGGAAAGCAATTATGAACCAGATGAGCTGACCAAAGAGATGGCCCACCTGGA

AGGACTTATGAAGGACCTTAATGCCATCACTACAGCATGA

>Duck ONECUT2

ATGAGGAGCGGGCGCGGCGCCTACCGATGCCTCGCCGCCGCGCCGGCCGCCTGCGCCATGAACCCCGAGC

TGGCGATGGAGCCGCTGGGCAGCCTGCACGGGGCGGCCGGCCATGAGCCCGAGCTGATGGGCAGCCCCAG

CCCGCACCACGGCGGCCGCGGCCCCGCCGGGCCCCTACGGGTGCCCCCCCCGCTNNNNCAGGAGCGGGCC

CCGGCCGCCGCCCGGCCGGCCATGGTGCCCGGCATGGCCTCGCTGCTGGACGGCGCCGCCGAGTACCGGC

CCGAGCTGTCCATCCCGCTGCACCACGCCATGAGCGTGCCCTGCGAGCCCTCGCCGCCCGGCATGGGCAT

GAGCGGCACCTACACCACGCTGACGCCGCTGCAGCCCCTGCCGCCCATCTCCACCGTGTCCGACAAGTTC

CACCACCCGCACGCCCACCCGCACGCCCACCACCACCACCACCACCAGCGCCTGTCGGGCAACGTCGGCG

GGAGCTTCGCCCTGATGCGGGCCGAGCGCGGGCTGCCCGCCGTCAACAACCTCTACGGGCCCTACAAGGA

GATGTCCGGCATGGGGCAGAGCCTGTCTCCCTTGGCTGGTACCCCCCTGGGCAACGGATTGGGGTCCATC

CACAACACCCAACAGAGCCTCCACAACTATGGGCCTCCCAGCCACGACAAGATGCTCAGCCCCAACTTTG

ATGCCCACACTGCCATGCTGGCCAGGGGTGAGCAGCACCTCTCCCGGGGGTTGGCGACCCCTCCCGCGGC

CATGATGTCCCACTTAAACGGCATGCACCATCCTGGACACCCTGGCCATGCTCAGTCTCACGGGCCCGTG

TTGGCTTCCAGCCGGGAAAGGCCGCCCTCCTCCTCTGGATCTCAAGTTAACAACTCCGGGCAGTTAGAAG

AAATCAACACCAAAGAAGTGGCACAACGGATCACTGCAGAGCTGAAGCGCTATAGCATCCCCCAGGCGAT

CTTTGCCCAGAGAGTGCTGTGCCGTTCTCAGGGGACCCTTTCAGACCTTCTAAGGAACCCTAAACCTTGG

AGTAAACTGAAATCTGGCAGGGAGACTTTCAGGAGAATGTGGAAATGGCTGCAGGAACCCGAGTTCCAGA

GAATGTCAGCCTTAAGACTTGCAGCATGCAAACGCAAAGAGCAAGAACCAAATAAAGACAGGAACAATTC

CCAGAAGAAATCTCGTCTGGTTTTCACCGATCTCCAGCGCAGAACACTTTTTGCCATCTTCAAAGAGAAC

AAACGTCCATCCAAAGAAATGCAGATCACTATTTCCCAGCAGCTGGGCTTGGAACTTACCACGGTCAGTA

ACTTCTTCATGAACGCTAGGAGGCGCAGCCTTGAGAAATGGCAGGATGATTTGAGTACAGGGGGATCCTC

GTCAACCTCCAGCACTTGTACCAAAGCATGA

>Duck POLI

ATGNNNNCAGGCCACAGCAAGTCGGTGCCAGCCAGAAACGCGGCGGGCAGAGTAATCGTGCACGTGGACT

TGGACTGCTTTTATGCCCAAGTAGAAATGGTCCGTAATCCCGAGCTAAGAGACAAGCCTCTAGGTGTGCA

GCAGAAATTCCTTGTAGTAACCTCAAACTATGAAGCCAGAAAACTCGGAGTTAAGAAACTGATGTCTGTG

AAAGATGCTAAAGAGAAGTGTCCTCAGCTGGTACTGGTTAATGGAGAAGATCTAACTCCATACAGGGAAA

TGTCATACAAGGTTACAGGATTGTTGGAAGAATTTTGTCCTCTAGTGGAAAGGCTCGGGTTTGACGAAAA

TTTTGTAGATATCTCAGAGATTGTCGAAAAAAGGCTAAACCAGCTACAACAAAGTGGATGTTCCAGAGTG

TGTGTGTCTGGCCACGTGTACAACAACCAAGCTATCGATTTACGTGATACAACGCACGTAAGGCTGGTTA

TTGGATCTCAGATCGCAGAGGAGTTCAGGCAAGCCATGCACACTCGTCTCGGCCTCACAGGCTGTGCAGG

AGTGGCCTGTAACAAGTTGCTGTCTAAGCTGGTATCTGGGACCTTTAAACCAAACCAACAAACACTTCTG

CTGCCTGAAAGCTGTCAAGATCTGATGCGCAGCCTTGATCACATCCAAAAAGTGCCTGGCATTGGCTACA

AAACTGCCAAACGTCTCGAAACACTGGGCGTGAGGAGTGTGTGTGATCTCCAAACATTTCCATCCCCCGT

GTTAGAGAAGGAACTTGGGGCTTCCGTTGCTCAGCGCATCCAAAAACTCAGCTACGGAGAAGACGACTCC

CCCGTGACTCCATCAGGCCGCCCTCAGTCCTTTAGCGATGAAGATTCCTTTAAAAAATGTTCCTCAGAAG

CGGAAGTGAAAGAGAAAATGGAAGAACTGCTTCGGAGCCTCTTAGACAGGGTATCCAAAGACGGAAGGCA

GCCACACACCGTGAGGTTGACTATCCGCCACTTCTCTGCGACCGATAGGTGGTTTAATCGGGAAAGCCGT

CAGTGCGCTCTTCCACCTCATCTTGTTCAGAAATTTGGAAAAGAAAGCAGCAACATTATATCTCCACTGA

TTGATATACTCCTGAAACTCTTTCGAAAGATGGTAAATGTAAACTTACCATTTCATCTTACCCTCTTGAA

CGTTTGCTTCTCTAATCTCAAAGATGTTCCTAGCAGCAAGAAAGGATCGATAGGTTTTTATCTCAAGCAG

ATGTCACCACCAACGTCAGGCTCTTGTAAAAGTGTCCAGGAGATGGAAGACGGCTCACAAGGTGAAGGAA

GTGCTTCTTGGAACCAGAACTGGAACAGAACTGGCACTACAAAAGCGAAGAGACCTTCAGAGGAAAAGGA

AAGCGATACAAAGAAAGCAAGAATTCCTGCCTTCCCATGTCATTTGTCTCCTGGTGGTATTGACCAGGCA

GTCTTCAGGGAACTTCCAGAGGATATTAAAAAGGAAATCATGTCTGAAAAAGCAGGAGAAGTGATCTCTG

CTGGGGATGTTTTGGGTCAGACACCGGTATGTTTCGCAGAGGAGGTACAGAGCTCCTCCCCGAATTCTAA

GGGTGATGAGAACTCTGCAGGGTACAGTGCACACCTCACACCAGCTTGTGAATCAGCATCAGCTTGGACA

CACAGCTCCAGCACCAGTTTTTCTGCTGAGTATCCTAAAAGTGTGTTAACAGGCATGGAAGAAAACCCTC

CTGACTCTCATAGCTTCTGGGATTTATCAGTTGAAGCTGGCGCCAGCCAAACTGCTCCACGTGTCCCTGC

CTCGGATAAAGATGAGCAGGATTCTGAAATCACTTCCGAGGATAAAAACGACAGCAGGAAAGTGGGAATT

GTACTTCCTCCTAGTGTTGATCCGAAGACTTTTTATGAACTACCCAGAGATGTGCAGGAAGAACTCCTAG

CCGAATGGAAGAATCGGGAACCTGTGTCCAAAACCTGTATAGAAAAAACCCCAGAAAGGCCTAAAACAAA

CAGAGGAAAAAAGAACGCAGCGTTGAGTTCCTCACAGTCTAACAGTTTGCTGAGGTATTTTAAACCACGG

TGA

>Duck RAB27B

ATGACTGATGGAGACTACGATTATCTGATCAAACTCCTGGCCCTTGGAGACTCCGGGGTTGGCAAGACCA

CGTTCCTCTACAGATACACCGACAACAAATTCAATCCCAAATTCATCACGACAGTAGGGATAGATTTTCG

GGAAAAACGAGTGGTGTACAATAGCAGAGGAGCAAATGGATCTCCAGGAAAAGCCTTCAAGGTGCACCTC

CAGCTTTGGGACACCGCTGGACAGGAAAGATTTCGAAGCCTCACCACAGCGTTTTTCAGAGATGCTATGG

GCTTTTTACTGATGTTTGATCTCACCAGTCAACAGAGCTTCTTAAATGTCAGGAATTGGATGAGTCAGCT

GCAAGCCAATGCGTACTGTGAGAACCCAGATATCGTGTTAATTGGTAATAAAGCGGATTTATCCGACCAG

CGGGAGGTAAATGAAAGGCAAGCGAAAGATCTGGCGGACAAATACGGCATCCCCTACTTCGAAACGAGTG

CTGCTACCGGCCAGAATGTGGAGAAGGCCGTGGACACGCTGCTGGACTTGATAATGAAACGGATGGAGCA

GTGCGTGGACAAGACGCAGGGCTCCGACACGGCCAACGGGGGAAGCTCGGGGAAGCTGGATTCAGCAAAA

CCGGAGGAGAAGAAGTGTGCCTGCTAA

>Duck RPL17

ATGGTCCGCTACTCGCTGGATCCGGAGAACCCCACGAAATCATGCAAGTCCCGGGGCTCCAACCTGCGGG

TGCACTTCAAGAACACTCGTGAGACTGCCCAGGCCATCAAGGGCATGCACATCCGCAAGGCCACCAAGTA

CCTGAAGGACGTCACCCTGAAGAAGCAGTGCGTTCCCTTCCGGCGCTACAACGGAGGCGTTGGTAGATGC

GCCCAGGCCAAGCAGTGGGGCTGGACGCAGGGCCGCTGGCCCAAGAAAAGCGCGGAGTTCTTGCTGCACA

TGCTGAAAAACGCGGAGAGCAACGCTGAGCTCAAGGGGCTGGACGTGGATTCGCTGGTGATCGAGCACAT

CCAGGTGAACAAGGCCCCCAAAATGCGCCGGCGCACCTACCGAGCCCACGGGCGGATCAACCCCTACATG

AGCTCCCCCTGCCACATCGAGATGATCCTCACCGAGAAGGAGCAGATCGTCCCCAAGCCCGAAGAAGAAG

TGGCTCAGAAGAAAAAGATCTCGCAGAAGAAGCTGAAGAAGCAGAAGCTGATGGCCCGGGAGTAG

>Duck SKA1

ATGGCTTCTTCGGATCTGGAAGACTTGTGCTTTCACATCAACATGAAGATTTCAACTATTAAAAAGACTC

TGCAATTAAGGAGCATCGGTCAAGAGCCATCCTTCAAGTCTATGCTCTGCAAAATAGGACAAGAGATGGT

TCTCTTGCATGATCTCCTGAATAAAATGGAAATGGAAGTTCAACAGCAAGAAAAACTGAAGAATTTGCTC

AAAGAGCTCCAGAAGTCTGCTGAGCGAGATCAAAGCGAAGCCCAGCACCTCCGCGAAAACATTCCCCTCC

ACCTGCCTAAACCGACTCGGAGCTGTATTGCTGGGCCGGCTGCGAAAAGCGAGGACCAAATGAAAGCTGC

AGAACCTGAACACGCAAAAAAACCTGCCAAGGAGCCAAGATTTATTAAAGAAGCATCTTTAATAACCACG

GAAGAATTTGAAAGCGTTCCCGCGTACATGAAAGGCCGCTTGACGTACGATCAAATTAACGCCGTCGTTC

AAGACCTCAACAAGGCCGTGGTGGGCAAGTACAAGATCCTGCACCAGCCTCTGAAATCCATGAACGCGTC

GGTCAGGAATCTCTACCACCGATTCCTGGGGGAGGAAACGAAGGATACGAAAGGCGAGTTTTTTGTCGTG

GAGGCCGACATCAAGGAGTTCACCCAGCTGAAAGTGGATAAGCGCTTCCACAGGATCCTCAACATCCTGC

GCCACTGCCAGCGGGTCAGAGAAGTCCGCGGCGCGCGCCTCGTCCGCTACGTCATCTGCTGA

>Duck SMAD4

ATGGACAATATGTCTATTACTAACACGCCAACAAGTAATGATGCTTGTCTGAGCATTGTTCACAGCTTGA

TGTGCCATCGCCAAGGTGGAGAGAGTGAAACTTTTGCCAAACGGGCAATTGAAAGTTTAGTTAAAAAGCT

AAAGGAGAAAAAAGATGAATTGGATTCTTTGATTACAGCTATAACCACAAATGGAGCTCATCCTAGCAAG

TGCGTTACAATACAGAGAACGCTGGATGGGAGGCTTCAGGTGGCCGGTCGCAAGGGATTCCCTCATGTGA

TTTACGCTCGTCTTTGGAGGTGGCCCGATCTTCATAAAAATGAACTCAAGCATGTTAAATATTGTCAGTA

TGCTTTTGACTTAAAATGTGACAGTGTCTGTGTAAATCCTTACCATTATGAGCGTGTAGTATCGCCTGGC

ATCGATCTCTCAGGACTGACACTACAGAGTTCTGCCCCATCAAGCATGTTGGTGAAAGACGAATACGTTC

ACGATTACGAGGCGCAGCCGTCGTTGTCTTCAGCTGAAGGACATTCAGTCCAAACCATCCAGCACCCGCC

GAGTAACAGGGCATCTACGGAGCCTTACAGCACCCCAGCCATGTTAGCTCCCACCGAGGCTAGCACTACC

AGCACCACTAACTTTCCCAACATTCCTGTGGCTTCAACAAGTCAACCTCCCAGTATATTGACAGGTAGCC

ATAGTGATGGACTCTTACAGATTGCTTCAGGGCCTCAGCCAGGAGCTCAGCAGAATGGGTTTACAGCTCA

GCCAGCTACTTACCATCACAATAGTACTACAACTTGGACTGGAAGTCGAACGGCAGCCTACACACCTACC

ATACCTCACCACCAGAATGGCCATCTTCAGCATCACCCACCTATGCATCCTGGACATTACTGGCCAGTTC

ACAATGAACTCGCATTCCAGCCTCCTATATCAAATCATCCTGCTCCAGAGTACTGGTGTTCAATTGCGTA

TTTCGAAATGGACGTGCAAGTCGGGGAGACGTTCAAGGTCCCTTCGAGCTGTCCCATCGTTACCGTCGAT

GGCTACGTGGATCCCTCCGGAGGAGACCGCTTCTGCCTGGGCCAGCTTTCCAACGTGCATAGAACAGAAG

CCATCGAGAGAGCAAGGTTGCACATAGGGAAAGGGGTGCAGCTGGAGTGCAAAGGAGAAGGTGACGTGTG

GGTTCGATGCCTCAGTGACCACGCGGTCTTCGTTCAGAGCTACTACCTGGACAGAGAAGCAGGGCGTGCG

CCGGGGGATGCTGTTCACAAGATTTACCCAAGTGCATACATAAAGGTGTTTGATTTACGCCAGTGTCATC

GTCAGATGCAGCAGCAGGCTGCCACTGCTCAAGCCGCCGCTGCTGCTCAAGCTGCAGCAGTAGCAGGAAA

CATCCCCGGCCCGGGATCAGTAGGTGGAATAGCCCCAGCCATTAGTTTGTCAGCTGCTGCTGGAATCGGC

GTAGATGACCTCCGCCGCTTGTGCATACTCAGGATGAGTTTTGTAAAAGGTTGGGGACCTGATTACCCGA

GACAGAGCATCAAAGAGACACCGTGCTGGATTGAAATTCACTTGCACCGTGCCCTCCAGCTTCTAGACGA

AGTACTTCATACCATGCCTATCGCCGACCCACAACCTTTAGACTGA

>Duck SMAD7

ATGTTCAGGACCAAACGCTCGCTGCTCGTCCGGCGGCTCTGGCGGAGCCGTGCCCCCGGCGGCGAGGAGG

AGGCGGGCGAGCCCGGCGGGGCGGCCGAGGGTCGGGCGCATGCCGGCGGCGGGGGGCGAGGGTGCTGCCC

CGGCAAGGCGGGCAGGGGCGGCCGGGGGGCTGCGGAGGCGGAATTGAAGGCTCTGACCCACGCCGTGCTG

AAGCGGCTGAAGGAGCGGCAGCTGGAGGGGCTGCTGCACGCCGTGGAGTCCCGCGGAGGGGCGCGCACCC

CCTGCCTGCTGCTGCCCGCCAAGGGCGACTCGCGGCTGGGCCAGCACTGGTACCCGCTGCCCGTGCTGCT

CTGCAAGGTGTTCCGCTGGCCCGACCTCCGGCACTGCTCCGAAGTCAAGAGGCTGTGCTGCTGCGAGTCG

TACGGCAAGGCGCACCCCGAGCTCGTCTGCTGCAACCCGCACCACCTCAGCCGGCTCTGCGAGCTAGAGT

CTCCCCCTCCACCCTACTCCAGATATCCAATGGATTTTCTCAAACCAACGGCAGATTGTCCAGACTCTGT

GCCTTCCTCCACTGAAACAGGGGGAACTAATTGTCTAGCCCCTGGGGGGCTTTCAGATTCCCAAGTTCTT

CAGGAGCCGGGGGATCGGTCACACTGGTGCGTGGTGGCATACTGGGAAGAGAAAACGCGCGTGGGTCGGC

TGTACTCTGTCCAAGAGCCCTCCCTGGATATCTTCTATGATCTACCTCAGGGGAATGGTTTCTGCCTCGG

ACAGCTCAACTCGGACAACAAAAGCCAGCTGGTGCAGAAGGTCCGCAGCAAGATCGGGTATGGCATCCAG

CTCACTAAGGAAGTGGATGGCGTGTGGGTGTACAACCGCAGCAGTTACCCCATCTTCATCAAGTCGGCCA

CACTGGACAACCCCGACTCCAGGACATTGCTGGTGCACAAAGTGTTTCCAGGTTTTTCCATCAAGGCTTT

TGACTATGAGAAGGCGTACAGTTTGCAGAGGCCAAATGACCATGAGTTCATGCAGCAACCATGGACCGGA

TTTACTGTTCAGATCAGCTTTGTGAAAGGCTGGGGCCAGTGCTACACGAGACAGTTTATCAGCAGTTGCC

CGTGCTGGTTGGAGGTTATTTTTAATAACCAATGA

>Duck ST8SIA3

ATGTCTATGGGGGTGAGCTTCATAAGGAAAAAGAAATGGAGCTTGAAGAAACACGGCAAGTCGCAGTTTG

CGCTGAAGTTCCTGGACCCCTCGTTCGTGCCCATCACGAACTCCCTGAGCCACGAGCTGCAGGAGAAGCC

CTCCAAGTGGGTGTTCAACCGGACCGCCTTCGCGCACCAGAGGCAAGAAATCCTTCAGCATGTCGATGTC

ATAAAAAATTTTTCTTTGACCAAGAATAGTGTCCGGATCGGCCAGCTGATGCATTATGATTATTCCAGCC

ATAAGTACGTTTTTTCTATCAGCAATAACTTCAGATCGCTGCTTCCAGACGTGTCTCCGATCCTCAATAA

GCATTACAACATCTGTGCCGTGGTTGGAAACAGCGGGATCCTGACCGGGAGTCAGTGCGGACAAGAAATA

GACAAATCCGATTTTGTTTTTCGTTGCAATTTTGCTCCGACTGAGGCATTCCAAAAAGATGTTGGAAGGA

AAACCAATCTCACAACCTTCAACCCCAGCATCCTGGAGAAGTATTACAACAATCTCTTGACCATTCAGGA

TCGCAACAACTTCTTTTTAAGTCTCAAAAAGCTCGATGGAGCCATTCTGTGGATCCCGGCTTTCTTCTTC

CACACATCAGCAACGGTCACAAGAACACTGGTTGACTTCTTTGTCGAGCACAGAGGGCAACTAAAGGTCC

AGTTGGCTTGGCCAGGAAATATAATGCAACATGTGAACAGATACTGGAAAAACAAACACCTGTCACCCAA

GCGGCTGAGCACAGGTATTCTCATGTATACACTCGCTTCTGCCATATGCGAAGAGATTCACTTGTACGGA

TTCTGGCCGTTCGGGTTCGACCCCAACACGAGGGAAGACCTCCCATACCACTACTATGATAAGAAAGGAA

CAAAGTTCACAACCAAGTGGCAGGAGTCCCACCAGCTGCCTGCAGAGTTCCAGCTGCTCTACAGGATGCA

CGGTGAAGGACTGGCCAAACTGACCTTGTCGCATTGTGCCTAA

>Duck STARD6

ATGGACTATAAGAAAATCACGGATGAAATCTCAGAAAAAATCTTATCCTACAGCCAGGATACTTCAGGAT

GGAGGGTGATAAAAGTTTCGAAAAATGTTACAGTTTCTTCCAAGCCTTCCAAAGAGTATGCGGGAAATAT

ATACCGTGGAGAAGGGATAATTGAGGAAGTCCCTAGTAAAATTATCCCTTTTATGTATCTTCCTGAATAT

CGAAACAGATGGGACAAAGCACTAAAATCTTACAGTCTGTTAGAAAGCATTGACCAGGACACCGGTATAT

ACCACAGCGTAACGCACAGCTATGGCATGGGCCTGATTTCCTCGCGAGATTTTGTCGACCTGCTGCATGT

CAAAGCTTATCCTGGTGGCATCCTTACAACTAACTCCATCAGCGTGGAATATTCCAGCTGCCCTCCAACT

CCCTCTTGTGTCCGAGGCCGTAACAATCCCTGTGGGTACGTGTGTTCTCCCTTGCCTGAGAATCCAGAGC

ACTCTAAGCTAGTTGTATTTATTCAGCCAGAACTCGGAGGAATGCTTCCCTGCTCCGTGGTGGAGACAGC

GTTACCTACGACTCTCGTAAACTTAATCACTGAAACAAGGGCTGGACTGAAAAGCTTTAAAGACCGTAAT

TAA

>Duck TCF4

ATGCATCACCAACAGCGAATGGCTGCCTTAGGGACGGACAAAGAACTGAGTGATTTACTGGATTTCAGTG

CGATGTTTTCACCTCCTGTGAGCAGTGGCAAAAATGGACCAACTTCCTTGGCAAGTGGACATTTTACTGG

CTCAAATGTAGAAGACAGAACTAGCTCAGGGTCCTGGGGGAATACAGGACATCCTAGTCCATCCAGGAAC

TATGGAGATGGGACTCACTACGATCATATGGCGAGCAGAGACCTCGGGTCACACGACAATCTCTCTCCTC

CTTTTGTCAATTCCAGAATACAAAGTAAAACAGAAAGGGGCTCGTACTCGTCGTATGGAAGAGACTCGAA

TTTACAGGGTTGCCACCAGCAAAGTCTCCTGGGAGGGGAGATGGACATCGGCAACCCCGGGGCGCTCTCC

CCCAGCAAGCCGGGCTCCCAGTACTACCAGTACTCCAGCAATAACCCCCGCCGGAGGCCTCTGCACAGCA

CCTCCATGGAAGTACAAACAAAGAAAGTTCGGAAAGTTCCTCCAGGTTTGCCGTCCTCAGTTTATGCCCC

GTCAGCAAGCACTGCCGACTACAATAGGGATTCACCAGGTTATCCATCCTCAAAACCAGCAGCCAGCACT

TTTCCTAGCTCCTTCTTCATGCAAGATGGCCATCACAGCAGCGACCCGTGGAGCTCCTCCAGCGGGATGA

ACCAGCCCGGCTACGGGGGCATGCTGGGCAACTCTTCCCACCTCCCCCAGTCCAGCAGCTACTGCAGCCT

GCACCCCCACGACCGCTTGAGCTACCCATCCCACTCCTCAGCAGACATCAACTCCAGTCTTCCTCCGATG

TCCACCTTCCACCGCAGCGGCACGAATCATTACAGCGCCTCGTCGTGCACGCCGCCGGCCAACGGGACCG

ACAGCATCATGGCAAACAGAGGAAGTGGGGCGGCAGGCAGCTCGCAGACTGGTGATGCGCTGGGGAAAGC

ACTTGCCTCTATCTATTCTCCAGATCACACCAACAACAGCTTTTCATCAAATCCTTCAACTCCTGTTGGT

TCTCCCCCTTCTCTCTCAGCAGGCACAGCTGTTTGGTCTAGAAATGGAGGTCAAGCGTCATCATCTCCCA

ATTATGAAGGTCCCTTACACTCTTTGCAAAGCCGGATCGAGGACCGCCTGGAGAGGCTGGACGACGCCAT

CCACGTGCTGCGCAACCACGCCGTTGGGAGCTCTGGGACTGTCCCGGGCGGCCACGCCGACATGCACGGC

TTGATAGGGCCCTCGCACAACGGCGCCATGGCGGGCCTGGGCTCCGGCTACGGCACCGGCCTGCTCTCCG

CCAACAGGCACTCGCTCATGGTCGGCGACAAAAAAGAGGCAGGGGTAGGCCTCCGCGGCAGCCACTCGCT

CGTGCCAAACCAGGTGCCGGTGCCCCAGCTGCCCGTGCAGTCGGCCACGTCCCCGGACCTGAACCCGCCC

CAAGACCCCTACAGGGGCATGCCAGCTGGACTGCAAGGGCAGAGCGTCTCTTCAGGGAGCTCCGAAATCA

AATCCGACGACGAGGGAGACGAAAACCTCCAGGACACAAAATCTTCCGAGGACAAGAAACTAGAGGATGA

CAAGAAGGATATCAAATCAATTACTAGCAATAACGACGACGAGGACCTGACACCCGAGCAGAAAGCCGAG

CGAGAAAAGGAGAGGCGGATGGCCAACAACGCTCGGGAGCGCCTGCGCGTGCGCGACATCAACGAGGCGT

TCAAGGAGTTGGGCCGGATGGTGCAACTCCACCTGAAGAGCGACAAGCCCCAGACGAAGCTCCTGATTTT

GCACCAGGCTGTGGCTGTCATCCTCAGCTTAGAGCAGCAAGTCAGAGAAAGAAACCTGAATCCTAAAGCA

GCCTGTCTGAAAAGAAGGGAAGAAGAGAAAGTCTCTTCGGATCCTCCTCCACTTTCCCTGGCAGGACCCC

ACCCTGGGATGGGAGATGCCTCCAATCACATGGGACAGATGTAA

>Duck TSPAN3L

ATGGACTGCGGCGTGATCACCTCCAAAACCCTGCTGCTAATTTCTCACATTGCCTTTCAGGCGGCGGCGG

CTGGCCTCAGCTATGTCGGGGGCTACGTAATGAACACCTACAGGAGCTACGACAACTTCCTGCAGGACAA

GTACGCGCTGCTGCCCGCCGTCATCATCCTCTGCGTGGCAGCCGTGATGTTCATCATCGGGCTGCTTGGC

TGCTGCGCCACCTTCCGGGAATCTCGGGTTGGCCTGGGGCTGTTCTTGGCCATTATCCTCATTATCTTCA

TCGCAGAGGTTTCCGCTTTTGTCCTGGCGTTTGTTTACAGGGAGAAGGTAAAAAGTGACGTTCAAGGCAC

GATGCGCGCAGTCTTCGACAAGTACGACGGGAAAAACTCAGAGTCTGCTGTGATGGATTACTTGCAAGAA

AACCTTCACTGCTGTGGGGTGAAGAACTACAGCGACTGGACAACCACGCAGTGGTTTAATTCCACTGGGA

ACAACAGCGTCCCCCTGAGCTGCTGCAAGCAAAATCTGAAGAACTGCACGGGGAGCCTGGATCAGCCGCA

GGAACTCAACACGCAGGGCTGTTCAGGGGAGCTGGAGGCCGGGCTGCAGAGTGTCATCAGCTACGCTATG

CTTGTAATCCTGGGCTTTGCCATTGTGAAGTTCTTTGGCATGCTGAGCGTCTGCGTGCTTACTTGCAAGC

GAGAAGACAGCGGATACCAGCCTCTTTACTCGGGGGTGTTCGCTTAATAA

>Duck TXNL1

ATGNNNNNATGTGGCCCTTGTTTAAGGATAGCCCCAGCATTCAATGCGCTGAGTAACAAATATCCCCAGG

CAACTTTTTTGGAAGTGGATGTGCATCAGTGCCAGGGAACAGCTGCTACCAATAATATATCAGCAACACC

GACGTTTCTGTTTTTTCGAAACAAAGTTCGAATCGACCAATACCAAGGAGCAGATGCTGTAGGTTTAGAA

GAAAAAATTAAGCAGCACCTGGAGAATGATCCTGGAAACAATGAAGATACAGATATTCCAAAAGGATATA

TGGATTTAATGCCATTTATCAATAAAGCCGGCTGTGAATGTCTTAATGAAAGCGATGAGCACGGATTTGA

TAATTGTTTACGTAAAGACTCTACCTACTTGGAATCAGACTGCGATGAGCAGCTGCTTATTACTGTAGCT

TTTAGTCAACCGGTCAAACTTTATTCTATGAAACTTCAGGGGCCAGATAATGGACAAGGTCCAAAGTATA

TAAAAATCTTTATCAACCTTCCTCGATCTATGGATTTTGAAGAGGCAGAGAGAAGTGAACCAACTCAAGC

CCTGGAGCTAACACCAGATGATATTAAAGAAGATGGCATTATCCAGCTTCGCTATGTTAAATTTCAGAAT

GTTAACAGTGTAACTTTGTTTGTCCAATCTAATCATGGTGACGAAGAGACAACAAGAATTACATACTTCA

CGTTTATTGGAACTCCAGTCCAAGCAACAAATATGAATGACTTCAAGCGAGTAGTTGGCAAAAAAGGAGA

GAGCCACTAG

>Duck WDR7

ATGGCAGGGAACAGCCTGGTGCTGCCCATTGTGCTGTGGGGTCGGAAGGCTCCCACCCACTGCATCTCAA

CGCTGCTGCTAATGGATGACGTCTCCATGATCGTCACGGGCTGCCACGACGGACAGATATGTCTCTGGGA

CCTCTCTCTGGATTTAGAGATTAATCCCCGAGCTCTGCTGTTTGGTCACACAGCCTCAGTTACGTGTTTA

TCAAAGGCCTCTGCTTCCAGTGAAAAGCAGTACGTAGTGAGCGCGTCGGAGAGCGGGGAGATGTGTCTGT

GGGACGTGAATGATGGGAGATGCATAGAGTTTACGAAGCTGGCCTGCACACACACTGGCATACAGTTCTA

TCAGTTCACGGTCGGGACTCAGCGCGAAGGGAGACTGTTATGCCACGGCCATTATCCAGAAATTCTTGTT

GTGGATGCTACCAGCCTTGAAGTTCTTTATTCTTTGGTATCAAAGATATCTCCCGACTGGATCAGCTCCA

TGACTATCATTAAATCCAATAGAACACAAGAGGATACTGTTGTAGCAGTTTCGGTGACTGGCATCCTGAA

AGTATGGATAATAACCTCTGAAGTGAGTCGCATGCAGGATACCGCACCAATATTTGAAGAGGAGTCAAAA

CCTATTTATTGTCAGAACTGTCAAAGCATTTCTTTCTGCTCATTTACCCAGCGGTCGTTGTTGGTGGTGT

GCTCCAAGTACTGGAGGGTTTTTGATGCTGGAGATTATTCCCTTTTATGCTCGCTCCCTAGTGAAAATGA

ACAGACCTGGACCGGTGGTGATTTTGTGTCAGCTGATAAAGTCATTGTGTGGACAGAAGATGGACAAAGT

TTCATATATAAATTACCACCCAGCTGTCTACCAGCTAGTGATTCATTTCGCAGTGATGTGGGGAAAGCAG

TAGAAAATCTAAATCCTCCTTTATTGTACAGCGTATTGGACAGAGCAGATAAACAGTTACTTATATGTCC

CCCAGTCACTCGATTCTTCTGCAGACGTAGGGACTTGTCCTATAAGCTGCTAATCCAAGGAGACTCTTCA

GGAAGACTGTCTATTTGGAGTATGCCTGAAAATCTTGAACAACAAGATAGTGCAGAAGGACTGCAAACAA

CAACTTCAATATCCCTGCAGGAAGCTTTTGATAAACTTACGCCTCGCCCAGCTGGGATTATAGACCAACT

AAGCTTAATACCCAACTTCAATGAACCACTTAAAGTTACGGCCAGTGTGTATATCCCAGCACATGGGCGT

TTGGTCTGCGGTCGGGAGGACGGAAGCATTGTTATTGTGCCAGCAACACAAACTGCTATAGTTCAGCTTT

TGCAAGGAGAGCATATGCTTCGGAGAGGTTGGCCACCTCACCGGACTCTCAGAGGCCATCGAAACAAAAT

TACATGCTTACTGTATCCTCATCAGGTTTCTTCTCGTTATGATCAAAGGTATTTGATCTCAGGTGGTGTG

GATTTCTCAGTCATCATGTGGGATATATTTTCTGGAGAGATGAAACACATCTTCTGTGTGCATGGTGGAG

AAATTACACAGCTTCTAGTTCCACCAGAAAACTGTAGTGCAAGAGTCCAGCACTGCGTTTGCTCTGTTGC

CAGTGATCACTCGGTAGGTCTTCTGAGCCTGCGGGAGAAAAAATGCATCATGCTGGCATCCCGTCACCTC

TTCCCTATTCAAGTAATAAAATGGAGGCCTTCTGATGACTACCTGGTAGTGGGATGTTCAGATGGCTCTG

TGTACGTCTGGCAAATGGATACCGGTGCGCTGGACAGGTGTGTGATGGGAATAACAGCAGTAGAAATCCT

GAACGCCTGTGATGAGGCAGTTCCTGCGGCCGTGGACTCCCTCAGTCATCCCGCTGTCAACCTGAAGCAG

GCCATGACTCGGCGGAGCCTGGCTGCTCTGAAAAACGTGGCACATCAAAAACTGCAGACTCTTGCCACTA

ACCTGCTAGCTTCTGAGGCATCCGACAAAGGGAACTTGCCTAAATATTCGCATAACTCCCTGATGGTTCA

AGCTATAAAGACTAATTTAACAGATCCAGATATACACGTGCTCTTCTTCGATGTGGAGGCCCTGATTATT

CAGCTGCTGACCGAAGAGGCCTCGAGACCCAACAGCGCACTCATTTCTCCGGAGAATTTGCAGAAGGCAT

CTGGCGGTTCTGACAAAGGAGGCTCCTTTTTGACTGGCAAACGAGCAGCCGTCCTCTTCCAGCAGGTCAA

GGAGACGATCAAAGAGAATATCAAAGAGCATCTCCTCGACGACGAGGATGAGGATGAAGAATCGATAAGG

CAGAGAAGAGAAGATGGTGACCCGGAATATCGCTCTAGCAAATCTAAACCATTAACTTTATTAGAGTACA

ATCTAACCATGGATACAGCAAAGCTTTTTATGTCTTGTCTTCATGCCTGGGGCTTGAATTCTGTCCTAGA

TGAGCTTTGCCTTGATCGCCTGGGGATGCTTAAGCCGCACTGCTCTGTGTCCTTTGGCCTCCTGTCCAGA

GGTGGCCACATGTCTCTGATGCTTCCCGGTTACAACCAGTCTGTAGGTAAGCCATCCTACGAGGGCGTGG

AGCTGGGAAGGAAAATGTCCATTACAGAAGGACTGGGAAAGGGGACGTACGGAGTGTCGCGTGCAGTCAC

CACTCAGCACCTCCTCTCTGTCATCTCGCTGGCAAACACGCTGATGAGCATGACGAATGCAACTTTCATT

GGAGACCACATGAAGAAAGGCCCGACCAGGCCACCTCGGCCTGGCACTCCAGAGATGACAAAAGTGAAGG

CACCTCCTTCGATGGCAGCAAGTCACGCAGCTCAAGGACAAATTAAGCAAGTTGCTCCTGCTGTTTCTTC

TAGCACTGAAGCTGGTCACTCTGGCTCTGACACTGCTCCTACTTTACATACCTGTTTCTTAGTAAATGAA

GGTTGGAGCCAGCTGGCCGCCATGCACTGCGTGATGCTCCCTGACCTGCTGGGGCTGGACAAGTTCCGAC

CTCCTCTCCTGGAGATGCTGGCCCGCCGCTGGCAGGACCGCTGCCTGGAGNNAAGAGAAGCTGCCCAGGC

CTTATTGTTAGCAGAACTGAGAAGAATCGAGCAGGCAGGTCGGAAGGAGACAATTGATGCGTGGGCTCCG

TATTTGCCACAGTACATTGACAGTGTTATATCNNCTGGAGTAACCACAGAAGCCATTCAGACCGCTACGG

CAAGCCCAGATGCGTTGGGAACAGAGGCCAAAGTTCAAGAAGAGGAGCACGATCTGGTTGATGACGACAT

CACAGCAGGTTGCCTGTCCGGTCTCCCGCAGATGAAGAAAATCTCGACTTCTTACGAAGAGCGCAGGAAG

CAAGCGACTGCCATCGTCCTGCTGGGGGTGATCGGGGCGGAATTTGGAGCTGAAATTGAGCCTCCCAAAC

TCCTGACTCGGCCGCGTAGCTCTAGTCAAATTCCCGAGGGATTTGGTTTAACCAGCGGCGGATCAAATTA

TTCCCTGGCGAGGCATACGTGCAAGGCGCTGACGTTCCTGCTGCTGCAGCCGCCGAGCCCCAAGCTGCCC

GCGCACAGCACCATCCGCAGGACTGCCATCGACCTGATCGGCCGCGGCTTCACCGTCTGGGAGCCCTACA

TGGACGTCTCCGCCGTGCTCATGGGTCTGCTGGAGCTCTGCGCCGACGCCGAGAAGCAGCTCGCTAACAT

CACAATGGGGCTGCCCCTGAGCCCGGCGGCCGACTCTGCCCGCTCGGCGCGACACGCTCTCTCGCTCATC

GCCACGGCCAGACCACCCGCCTTCATCACCACCATCGCCAAGGAGGTGCACAGACACACCGCCCTGGCTG

CGAACACGCAGTCTCAGCAGAATATCCACACCACCACCCTTGCTCGAGCTAAAGGAGAGATCTTACGGGT

CATTGAAATACTTATTGAGAAGATGCCTACCGACGTCGTGGACCTTCTGGTGGAGGTTATGGACATCATC

ATGTATTGCCTTGAAGGATCTTTAGTTAAGAAGAAAGGTCTTCAGGAATGCTTCCCAGCCATCTGCAGGT

TCTACATGGTCAGCTATTACGAGCGGAGTCACAGAATAGCAGTTGGAGCTCGCCATGGTTCAGTGGCCCT

CTACGACATCCGGACTGGGAAATGTCAGACGATCCACGGTCACAAAGGGCCCATAACTGCAGTGGCGTTT

GCTCCCGACGGCCGGTACCTCGCCACCTACTCCAACTCCGACAGCCACCTCTCCTTCTGGCAGATGAACA

CGTCCCTGCTGGGCAGCATCGGCATGCTCAACTCGGCGCCTCAGCTGCGCTGCATCAAGACGTACCAGGT

GCCGCCCGTGCAGCCGGCCTCCCCCGGCTCGCACAACGCGCTGCGGCTGGCCCGCCTCATCTGGACCTCC

AACCGCAACGTCATCCTCATGGCTCACGACGGCAAGGAGCACCGCTTCATGGTCTAG

>Elephant shark DCC

ATGNNNACACTGGATGTGACCTTTAACCCATGGCTCTCGTCTCTTGTCGTTTCAGGGTCTCAGATTAAAA

GGTTCATCTCTCTGCGCTTTGTATCCGAGCCTTCGGACACGGTGACGGTTCGAGGTGGCTCTGTCCTCCT

GAACTGCTCGGGGGTGGCAGACCAGGGCCCTGCGGTGACTGTCAGCTGGCGGAAAGATGGCGTCTTCCTC

AACCTGGCCCTGGACGAGAGGAGGCAGCAGCTGCCTGATGGATCCCTCCTCATCCAGAACGTGGTACATT

CCCGGCACCACCGGCCCGACGAGGGACTGTACCAATGCCAGGCCTCGGTGGAGGGGACCGGGGCCATCGT

CAGCAGGACAGCCCGGATCACCGTGGCGGGCCCGTTGAGGTTCATCTCTATGACGGAGCCGGTCTCGGCT

TACGCGGGAGACAGTGCCATCCTGAGGTGTGAAGTGACGGGCGAGCCCATGCCCTCCATCCACTGGCAGA

AGAACCGGGAGGACCTGAAGCCTAGCGTGGGCGATGCCAGGCTCTCAGTTCTGCCCTCCGGCTCCCTGGC

CATCAGTAAACTACAGGCATCCGATGCTGCTTCCTACCGGTGCTTGGCTGAGAATCCCGGGAGCTCTCGC

ACCGGAAACGATGTCGACCTGAAAGTCCTGTCTGAATCTGGAGTTAATCGGCAAATCGCCTTCCTTCAGC

GGCCATCCAACCTGGCGGTCCTGGAGGGAAGGGACGGAATTCTGGAATGTTCTGTCTCTGGATTTCCAGC

CCCCTTGGTGATTTGGAAGAAAGGAGATGAAATTATTCAGACCAGGTCGAGGAAGCACTCACTGCTGGCT

GGCAGTAACCTGCTGATCAGAGGCGTGACTGAGATAGACGCTGGCTTGTACACATGTGCCACTAGCTACA

GGAACGAGACCATCAGTGCCTCGGCGGAGCTCACTGTGATGGTTCCTCCCAGCTTCGTTAGATACCCTTC

CAACCTGTACGCCTATGAGAGTATGGACATAGAGTTTGAATGTTCCTTCACTGGAGTGCCATCGCCTATT

GTCAAGTGGATCAAGAACGGTGAAGTGGTCATCCCCAGCGATTACTTTCAGATTGTGAGCGGCAGTAACC

TGAGAGTGCTGGGGCTGGTCAGGTCAGACGAAGGGTTTTACCAGTGTATGGTGGAGAATGACGTGGGCAG

CGTACATGCCAGTGGGCAGCTGATCATTTCCGAGCCAGCCTTACCAAGCTCCAGTGTCCTCCCCTCGGCC

CCTCGGGATGTGGTCCCTGTCCTAGTGTCCAGTCGCTTTGTCCGTCTGAGCTGGCGCCCACCTGTCGAGA

CCCACGGAGCAATCCAGACCTACTCCGTCTACTTCACAAGAGAAGGCATCAACAGGGAACGTGCCCTCAA

CACCTCTCAGCCTGGGGACCTGCAGCTCACCGTGGGGAACCTGAAGCCCGAAGAGACCTATTTGTTCAGG

ATTGTGGCCTACAGCGATGTGGGGCCAGGCCAGACCTCGCAGGCCATTAAGGTCTCCACCCAGCCTGAGC

TACAAGTTCCTGGATCACCAGAAAACCTTAGAGTTTCCTCCACCTCCTCCAGCTCCATCATGGCCTCTTG

GGAACCCCCAGCGTATGCAAATGGTCCAATCCAAGGCTACAGGTTCTTTTGGATGGAGATTACCAACCGC

AATGAGCAGAGTGTTGAGGTGGACGGCTTGTCGTACAGACTGGAGGGGCTCAGGAAGTTCACAGACTACA

GCCTGCGGGTGTTGGCTTTCAATAGACATGGTGCTGGGGTGTCTACCGAGGATGTGCTGGTCAGCACATT

GTCCGACGTGCCAAGTGGAGCTCCGCAGAATGTCTCTCTGGAGGTGGTCAACTCCCGGAGTATTAAGGTC

AGCTGGCTGTCACCACCTCCCAACCAGCAGAATGGCTTCATCACCGGCTACAAGATCCGGCACAGAAAGA

TTGGCCGCAGAGGCGACTTGGAAACCCTGGAGCCCAATAACCTCTGGTACCTCTTCACAGGTCTGGACAA

AGGCGGACACTACAGTTTTCAAGTTGCCGCTATGACAGTGAACGGGACAGGCCCACTGTCTGAGTGGCGC

ACGGTCGAAACACCTGAAAATGACCTGGACGAATCTCAGGTTCCAGACCAGCCAAGCTCCCTACATGTTA

GACCACTCACCACCAGCATTGTGATGAGCTGGACTCCACCTCTGAACCCCAACATCGCCGTGAGGGGGTA

CATCATCGGCTATGGGGTGGGGAGCCCCTATGCCGAGACTGTGAGGGTGGACAGCAAACAGCGCTACTTC

TCCATCGAAAACCTAGAACCCAGCTCCCACTATGTCATTTCCCTGAAGGCCTTAAACAACGCTGGGGAGG

GAGTTCCGCTGTACGAGAGTGCCACCACCAGGGCCTTGTCAGACCCTGTTGACCCTTTAGATGTTGATTT

GTTCCATTTGTTTGGTGACCTCCCAACCTCAGTCCCAGAGACCTCCACCCCCATGCTACCACCTGTAGGT

GTCCAGGCCATGTCGGTCAGCCACGACTCTGTCCGTGTCAGCTGGGCAGACAACTCTGCCCCCAAGAACC

AGCGGTTGGTGTCTGAGGTGCGTTTTTACACCGTGCGATGGAGGACCAGCTACTCTGCCAACACCAAGTT

CAAGTCTGCAGATACCACAGCCCTGAGCCATACCGTCACCGGCCTCAAACCCAACACCATGTATGAGTTC

TCCGTCATGGTAACCAAAGGTCGGAGGTCCAGCACGTGGAGCATGACGGCTCATTCTAAAACGTTTGAAG

CCGCACCCACCTCGGCCCCAAAGGATCTGACGATCATCAGCAGAGAGGGTAAGCCTCGAGCTGTCATCAT

CAGCTGGCAGCCACCAATAGAGGCCAACGGAAAGATTATCGGTTACTCCATGTTCTACACGACTGAGAAG

AACGCACCAATTGATGACTGGGTGATGGAGCCGGTCAGTGGGGACAGGCTGACCCACCAGATCCTGGACC

TCAACCTGGACGCGCTCTACTACTTCCGAATCCAGGGTCGCAACGCCAAGGGCCTGGGACCCCTGTCAGA

CCCCGTCCTGTTCAGAACACCTAAAGTTGAACACCCTGATAAAATGGCTAATGACCAAGGTCGGAGCGTG

CACGGAGATGGAGGCTTCTGGCCGGCCGACACCAACCTGATCGACAGAAGCAGCCTGAACGAGCCCCCGA

TTGGTCAGATGCACCCACCGCACGGCAGCGTGACTCCTCAGAAGAACAGTAACCTGCTGGTGATCATCGT

GGTGTCCATCGGAGCCGTGACCGTGGTCGTGGTCATTGTGGTGGCTATCATCTGTACCCGCCGTTCCTCA

GCACAGCAGAGAAAGAAACGTGCAGCACACAGTGGAGGTAAAAGGAAGGGGAGCCAAAAAGACCTGAGGC

CCCCAGACCTGTGGATCCACCACGAGGAGATGGAGATGAAAAACATTGAGAAGCCCCCAGGCACAGAGCC

CAGTGTCAGAAGCTCACCCGCACACCAAAGCTGTCAGAACATCACCCCCGTCAGCCAGAGCCAGTCAGAA

CACCAGCTGACCAGCAAGAGTGGCTCTCACTCAGGGCCCGATCCTGATGAAGTGGGGAGCAGCATGTCAA

CTCTTGAACGTTCAGTCACTGCTCGTAGAGGCACACGTCCTAAACTGATGATTCCTATGGACTCTCAACC

CAGCAACCCAACTGTGGTCAGTGCTATTCCTGTCCCGACATTGGAAAGTGCCCAGTATCCTGGCATCCTG

CCCTCCCCGACCTGCGGATACCCACACCCGCAGTTCACACTGCGCACCATGCCCTTCCCCACACTCACCG

TCGATCGGGGGTTCGTATCAGCCATGAGTGAGGTCCCAGTCTCGCACACTCAGCAGCCGTCTATGTTAAC

CCAGACCCAGGTTGAACACCCGTCGGGGGAGGAGGCCCCGAGCAGAACCATCCCTACAGCCTGTGTCCGA

CCCACTCATCCACTCCGCAGCTTCGCAAACCCCCTGCTACCTCCCCCCATGAGTGCCCTGGAGCCCAAAG

TCCCCTACACACCTCTCCTCTCTCAACAACCAGGTTCTAACCTTACCCTGAGTCACGGCAAGACAGCCTC

GCTGGGATTGGCCGGCAAAGCCAGATCACCGCTGCTTCCTGTGTCCGTACCCACAGCCCCAGAGGTGACA

GAGGAGAGCCACAGGACAGAGGAGCATTCTAATGTGTACGAACAGGATGACCTAAGTGAGCAAATGGCCA

GTCTGGAGGGTCTCATGAAGCAACTAAATGCCATCACAGGCTCCGCTTTCTAA

>Elephant shark NEO1

ATGGCGGAGCCCCGCGTGCGGCTCCTACTGACTCTGGCGGCGCTCAGCTCAGCTACAGGCACCTCAGAGT

CTGTCGTCAGGACGTTCACCCCTTTCTACTTCACTGCAGAACCAGCAGACACGCTTGCGATTCGAGGCTC

CTCGGTTATACTGAACTGTTCAGTCTTCACTGAACCCTCTGTCAAAATCGAATGGAAAAAAGATGGAACT

TTCCTAAATCTGGTGTCTGATGAACGCCGTGAGGTGCTGCCGGAAGGCTCCTTGTTGATACGAAATGTGG

TGCATTCGAAGCACCACAAACCTGACGAGGGATTTTATCAGTGCGTTGCCACCATTGATAGTCTGGGAAC

CATTCTCAGCAGAACAGCCAGGATGACTGTGTCAGGTCTTCCTCGGTTCAGCAGTCAGCCCGAAGCGATC

TCCGTGCACCAGGGAGACAGCACCCTTCTGAACTGTGAAGTAAACGCCGACCTCGGTCCCTTTGTGAGGT

GGGAGCAGAACCGCCAGCCCGTGGCGCTGGAAGGGAGGTTTCTGAAGCTGCCCAACGGAGCTCTGGAGAT

CAGCAATGTCTCAGACAGTGTGGCTGGATCATACCGTTGCGTTGTGGAGACCCCTGGTTCATCCAAAATC

AGCGAGGAAGCACAGCTTGACGTTTTACCCGATATGGGCATGGAGCGAAAGCTACTGTTTCTTCAACATC

CTGTCTCTGTGAACAAACTTACAGGTCAGACAGTAATATTACCATGTGTTGTCTCTGGTTATCCAACACC

CTCCATTACTTGGACCAGGAATGAGGAAAGCATCACAGAAAGTGATAGCTTCCAACGGATGGCTTTCGTT

GGTGGCGGGAGTCTGAGGATCAATGGTGTAACGGAAGAAGATGGTGGAATGTACAGTTGCATTGCAAAAA

GTGAAAATGAAACCTTGGAGATCCAGGCAGAACTGAACGTTCAAGTTCCTCCCCACTTTCTGAAGGGTCC

AGACAACATTTACGCTCGTGAGTCCATGGACATCGTCTTTGAGTGTGAGGTGACAGGAAAACCTACACCA

ACCGTGAAATGGATCAAGAATGGAGACGTGGTGATTCCGAGCGATTATTTTAAGATTGTTAACGAACAGA

ATCTGCAAGTGCTGGGCTTGGTGAAGTCGGACGAGGGTCTGTATCAATGTGTGGCAGAAAACGACGTGGG

CAATGTCCAGGCCAGTGCCCAGCTCATCATCCTGGAGCATGCAGCCGCCACGGCCGAACTCCTGTCCTCA

GCCCCTCGCGATGTGGTCGCTCTCCTCGTCTCCACACGTTTCATCAAGCTGACCTGGCGTCCACCAGCCG

AGCCCAAAGGAGAGATCCTCATGTACTCTGTGTACTACTCTCGGGATGGCATTAACAGGGAACGTGTGGA

AAATACCAGTGGACCTGGGAATCTGGAGGTAACCATCAAGGACCTTATGCCAGAAGTGAAGTACGTCTTC

CGAGTCATCGCTCACAACAAGCAGGGTCCAGGAGAGAGCTCGGCCCCATTCAAAGTGGCTACCCAGCCCG

AAGTTCAAGTTCCAGGTCCAGCTCCCAACCTCCAGGCTCTGACCACTTCGCCCACCTCCATCACCATCAC

CTGGGAGGCACCGCTCTCCGGCAATGGACCGGTTGTGAACTACAAGCTGTACTACATGGAGAAAGGGGCA

GTGAGCGAGCAGGATGTGGACGTTGAAAACCTTTCCTACACCATGAATGGGCTGAAGAAGTTCACGGAGT

ACAGCTTCCGCGTGGTGGCTTACAACAAGCACGGGCCCGGCGTCTCCACCGAAGACATCGTCACCAGAAC

CTATTCTGATGTGCCCAGTGCAGCTCCTCAGAATATCACAGTGGAAGTGCGCAATTCCAAGAGCATTGTG

GTCCATTGGCAGCCTCCCCCACCGGGCACCCATAACGGAGTGATCACAGGTTACAATATCCGCTACAAAA

AGGGAGGTCGCCGTGGCGAGACCGACAGCACCGGAGGGAACCAGCTGTCACAAATAATAGATGGATTGGA

GAGGAATACAGAGTACAGTTTCCGAGTCTCTGCTATTACAGTCAACGGCACCGGCCCCTCCTCTGAGTGG

CTGTCAGCTGAAACGTTTGAGAGTGACCTGGATGAAACCCGAGTCCCGGATGTGCCCAGCTCTCTTCATG

TCCGACCTCTGACTAACAGCATCGTGGTGAGCTGGACACCCCCCGAAAACCAGAACATTATTGTACGAGG

TTATGCGATTGGCTATGGCATTGGCAGCCCCCATGCAGAGACCATCAGGGTGGATTACAAGCAACGCTAT

TACACCATCGAACGGCTGGAGCCCAATTCGCACTACGTGATCACACTGAAGGCGTTCAACAAGGTTGGGG

AAGGAATCCCACTGTATGAGAGCGCAGCCACCAGACCCCTAACTGACCCCTTTGATCCTTCTAAGGTTTA

TTTATTTGATCCATACACTCCAGTACCAGACCCCAGCCCCATGCTGCCACCGGTTGGAGTCCAGGCCACT

GTGCTTAGCCACGACACTATCCGGGTGACGTGGGCTGACAGCTCACTGCCCAAAAACCAGAAGATCATCG

ATGCTCGCTTCTACACCGTACGCTGGAAAACCAACTACCCGGCCAACACCAAGTTCAAGACGGCGGACAC

CACAACGCTGAGCTATATTGTCTCGGGCCTGAAACCAAACGCGCTGTACGAGTTTTCAGTGCGAGTCACT

AAGGGACGGAGGACGAGTACTTGGAGTATGACCGCGCATGGGACCACGTTCGAGACAGTCCCAACCTCTC

CACCCAAAGACCTGACCGTGGTGAGTAAAGAGGGGAAGCCCAAGACCATCATCGTGAACTGGCAGCCACC

CTCTGAGGCTAATGGCAGAATAACAGGCTACATCATCTACTACAGTACTGACATCAGCGCTGAGATCCAT

GACTGGGTGGTGGAGCCAGTGGTTGGGGACCGGCTGACACACCAGATCCAGGACCTGACTCTGGACACCA

CCTATTACTTCAAAATCCAGGCCCGTAACTCCAAGGGGATGGGCCCTTTGTCAGACTTTGTGCAGTTCCG

CACACCTAAACAGTATGGAACGTCCGGGAAATCAGGAAATTCCTTTGTGATCGAGTCTCCTCCGGACCCC

AAGCATTCCACCAGTGGTCGAAACAGCCCTCACGGCAGCCCCACATCCAACCTGGACAGCAACATGATGC

TGGTCATTATCGTTTCTGTGGGCGTCTTCACCATCATCATCGTGGTCATCATCGCCGTCATCTGCACACG

ACGGATCAACACACAGCAAAAAAAGAAACATGCAGCCTGCAAGTCAGCCAATGGGTCTCACAAGTACAAA

GGCAACTGCAAGGATGTCAAACCCCCTGATTTGTGGATTCACCACGAACGGCTGGAGCTGAAACCTATCG

ACAAGTCGCCAGATCCCAACACGGTCATGACGGACACTCCCATTCCCCGCAATTCCCAGGACATTACTCC

TGTGGATGGTTCCATGGAGAAGGAGCAGCACCAGAGAAGGAATTCCTATCTGGCGCACGACCTCGACGAG

GGGTCTACACTGAGTGGACGCCGCGGGATGCGACCAAAGATGATGATGCCATTTGATGCACAGCCTCCAC

AACCTGTGATTAGTGCTCATCCCATCCATTCACTTGACAACCCTCATCACCATCACCACTCCGGCCTTTA

CTCCCCAACTCGCAGTTACCTTCATCACCAGATCAACAGCCGGCTGCTGGGCTCCTCTGTGCCGGTGGTA

GACAGGGCTCCTCCTACAGAATCTGTACGGAACACACCGAGCAGTGACATGTCACCGGCTCCAAGCTCCC

AGATCCATGCTGCAGAACACACGGAGCCTGACATCCCACCCGTGTCCTACATGCCGGCCTCCCAGGAGGA

AGAATCCAACCGAAATGTTCCCACAGCTCACGTGCGCCCATCCCATGCACTGAAAAGCTTTGCAGTGCCT

GCCATCCCGCCTGCCAGCACCAGCACCACCTACGAAACTGCATCATCCAGTACCCCATTATTATCCCAGC

CAGGAGGAAACCCTCAGGTGCACTCGGTGAAGACAGCGTCAATAGGAACTCTGGGGAGGAGCAGGTCACC

CATGCCAGTCACAGTGCCTAACGCACCTGACGTGGCAGAATCTGCAAAGATGCTGGAGGACTCTGATAGT

AATTATGGACCAGATGAACTCACTGAAGAGATGACTCACCTCAAAGGTTTAATGAAGGATCTGAATACTA

TTACCACAGCATAA

>Emperor penguin ACAA2

ATGNNNNNTGTATTCATTGTTGCGGCGAAGCGAACTCCTTTCGGGACCTATGGAGGTTTGCTGAAGGACT

TCACAGCCACCGATCTGACAGAACACGCTGCTCGAGCTGCGCTGGCTGCTGGCAAGGTCTCTCCTGAGAT

CATTGACAGTGTCATTGTCGGCAGTGTCATGCAGAGCTCCGCAGATGCGATTTATATTGCAAGACATGTT

GGTTTACGTGTGGGAATTCCTGTCCCAGTTCCAGCCCTCACTGTCAACAGACTTTGTGGCTCTGGTTTCC

AATCCATTGCCAGTGGATGTCAGGAAATTTGCCTTAATGACTCAGAAGTTGTTCTGTGTGGTGGAGCTGA

AAATATGAGTCAGGCTCCTTATGCAGTTCGAAACATTCGATTTGGAACCAGATTAGGAGCAGAACTCAAG

TTGGAAGACACACTGTGGGAAGGTCTAACAGATACGCATGTTAAAATACCTATGGCAGTTACAGCTGAAA

ATCTGGCTGCAAAATACAACATCACGCGAGAGGACTGTGACCGATACGCATTCAAAACGCAACAGAGATG

CAAAGCTGCTCATGATGCTGGTTACTTTAATGCTGAGATGGCACCAATTGAAGTGAAAACAAAAAAGGGG

AAAGAAAGCATGCAAAAGGACGAGCACCCAAAACCCCAGACCACTCTGGAACAATTGGCAAAACTCCCGT

CTATCTTTAAAAAGGATGGAACGGTGACTGCTGGGAATGCTTCAGGGGTGTGTGATGGAGCTGGTGCAGT

CATCGTTGCCAGCGAATCAGCACTTAAAAAGCACAGTCTTACTCCTCTGGCAAGAGTAGTAGCCTATCAC

TCAGCTGGCTGTGACCCTTCCATAATGGGCATTGGCCCTGTACCTGCAATTACCGAGGTTCTGAAGAAAG

CAGGATTGACCCTGAAAGACATGGATTTGGTAGAGGTGAATGAGGCATTTGCACCTCAGTATCTAGCTGT

CGAAAAAGTTTTGGGCCTTGACCCTGAAAAAACCAATGTCAACGGAGGTGCCATCGCTATAGGTCATCCT

TTGGGTGCTTCAGGATCACGGATCACAGCTCATCTGGTTCATGAATTAAGGCGTCGTGGCGGCAAATATG

CAGTTGGGTCAGCTTGCATTGGCGGTGGTCAAGGTATTGCTCTCATCATTGAGAACACAGCCTGA

>Emperor penguin C18ORF32

ATGGTGTGCATTCCTTGTATCGTCATTCCCGTTCTCCTCTGGGTCTACAAGAAATTCCTTGAACCGTACA

TCTATCCCGTTATCGCACCTTTCATTAAGCGCGTATGGCCCAAGAAAGCGGTGGAAGAAACAACAGCCGC

CAAACAAGGTCAAGGAGGCAGCGCTGGAAACCCACGGGCACCTTCAGCCACGAAGAGAGACCAGGAGGAT

GAGTCTGGAATTTATAAATTTGAAAGCAACGGCGTTGCAAATGGAATTGCTGCAGAGAGATCCACAGAAG

TTTCTGACAAGAAAACAGATTAA

>Emperor penguin C18ORF54

ATGACAAGCTCAAAAAAAGAAAGCAGTATCTGCTCTCCTGAGTCAACAATATCTTCTCTTTTAACAAGCT

ATAGCATTGATAGCAATAATCCATACTCAGACAGTTTGATTCAGTACAAGGACAAGCTTTACAGCTCTGC

ATCTGAGGCTCTGGAGGCTTATATTGAAGATTTTGATCGAAGTCTCACATCTTCAGAAATAAGCACTGGA

AAAATCTGCATATGTCAAAGCACTCCTAAACAAGTTCAGTTTGTAAAGCATCATGCCAGAAAAAAACATG

CATCGGATGATTTTAATCAGCACGTAGGACTGGGTTCTCTTGCTTCACCCTGTAGAAGGCAGACTGAGTG

TGACCCAGACTTGATTAGTCTCGCGACGGATGACCTGTTAGCATTTCCAGCAGATGGATCGCTGCCCTTT

GTCCAGCATACTCCTTTTAAATCAAGGCATCAAAGTAGTGAGTTGAACAGGCGGTCACTTAAAACATCTT

TCTGCCCTTACCAAACCTCATCACTCGATACTGAAAGCGGCTTCAGTAGTCTCCAGGAGAATGGCAAAGC

TGTTGCTCACCAGAATCCACACAAAGACTTCAGCAAAAAGAAACGCAATGTGTATACACCTGATAGGTGT

GATTCTGTCTCCTCTAAAGGAAGTTCAAGACCCTTCTCTTGTGAAGAGACCTCTAACACTTTTCCTTTTA

AGAATTATCCGAGGTGGCTTACCAGTCAGAAGTCTGACTTAAGTGTATCAGGGATAAGTAGTATTCCCAA

TTTTCACTACCCAGTCTGGCTTAAGAGTTACAACCTTTTTTCTGATTCAACTAAAGAAAATGATGGTCAA

AATTTTAATATACAAGGCAAAGCTTCCTCTTCACAAACTTTTGAGATCCTGACAAAAAGACACTCTGTCA

ATAAAGACAGTTCTAATTTTTTTGAACAAAACAGTTGCCTGGATCTGAGAGATGATAACAAAGTAGAAGA

ACGTTACAACTATGATAATCCAGATGCATGCTTCCAATTTGATAACTCGTTTTCAAGACACACCAAAAAG

CCATTCAGAGAAGACCAGCTTGAGCTACTTACCTTGAAGGCTGACAAAGGTCTGGAGAGTTCAACTGAAG

ACTTGTCAAATACTCTGGAAAATGATGGCAGTCCTTCTACAACAGATATACTAGGAGCAGAAAGATCATG

GGAAAATGCTCCAGGTGCTTTCAAACCACCAGTGCCTGTGTGCTGTGAGGACATGGAGAATGTGCTACCG

TTCCCCAAGGCAGATATTATACATAAGTTCTTGGAAGACTGTTTAAATGACAAAAATAAGGGAACTACTT

TTTCCGGCGGTTATCATCACAGGCCCCTTGAGGCTTTGAAGCTAACGTTGTTTAAACTTCAAGCAATTCA

GGGAAGTTTAAGCCAGAATGAAACTGCTGAGCAAAAGGAAGAGCTTGAAAAACTTTCTGAAAAAGCAGAG

GCTGAATTAAAACTGTGTGACAGTGAGATAATCCCCCTTACTAATTCTATTCAGAAGGCTTTACACCATT

TGTCGCGTCTTAAAAGTCTTGTTGAAGATAACAGTAACCAACAAGAGCAGACGGGTGATCATGAAGAAGA

TAAACAAGACAAAAAGAATGATCTGTGA

>Emperor penguin CCDC68

ATGACTACCCTGCTACTCACTGAGCAAATAACACAAGAAGACCACGGCTCGGAGGGAAACTACGTCCTTT

ATGGGTCCTCTTGTGCCCAAATCACTGAGGAAGCTGAATATGTGAAAAAGCTTCCTGAAGTCTTGGGCAC

TAAAGTGGGGTCACAGAACAGCGGCTGGAGCTGGAGCTGCGGTCCCGTTGCAAGGACGATGAAGGAAACA

GAAGAGCAGCTACTGCTGGTGAGCAGGGAAAACCAAGTGCTGAAGATCAAGCTGGAAGCCACGAGAGAAG

CGGGTGTACAGGCTCTCAGATCTGCCTCCCAGAAACTGTATGAGAATTACCAGACTCGGTCAGAAGAACT

GAAAAAAAGTCATGAGAATGAGAAGAAGCAAATCCAGGCCTTCAGTCTACAACAAGAAGAGAAGCTCCAG

CAAAGCTCAGAAAATGGCAGCCGCCTTGCTGAAGACGTCAGGGGAAAATGTACCCGCATCGCAGAGATGG

AGAAGCGCGTGCAAAGGATGGAGGAGGAAAAGAAAACTCTGATAGAGAAGAAAATGTCGTTTGAAAAGAT

GCTTCAACAGATGATGTCAAGGAATGAAGACAGCAAACGGTGCCTGGATCTCCAGAGGCAGATTTCCACC

CTGCAGGAGCAGATCTGCCACCTGCAGTGCGTGATCCGGGCGCAGCACCACGGCCTGCGCGGCGTGATAC

AGGAGGCAGAGGAACTGAACAACGAACTCAGAAGCCAAGATAAAAAAATAGAAAACCTGACAAAGAAGCT

GACTGCACTGGAAGCGCAGAATAAAGAACTGAAAGACAGAGTGGAGTTCTGGTCTGGCCAGTCCAAGACT

AAAGTTTCAAAAGCTGTCTGGACAGA

>Emperor penguin CFAP53

ATGAAGCACCGCTTTAGCCGAGCTGAAACACGGGAGTCTATTCGTGTGCATCAGCCAGTGTCTCTGCAGA

AGGGCCAAGTTCACACCTCCTACCCAACATCACCTACGTCCTACCCAACACTGCATTTGGAGGGAACAGC

TTTTAAGATGATCACCACAGGGAGAGCCAAGCCTCCTAAAGAACAAACAAGTGAAACTTTTGTCTTGGCC

CGCAGACAGAAAGAAAAGGAACTCCTCGAATATGCCGACTTCTTAAAGCTGTACAACCAGCATCGCACCG

TCTATGAGTGGCAGCAACGCAGCGAACAAAAATGGCTGCACAGCGTCGTGCGGAGAAAGGTGGACGCGAC

AGTGCAGGAGTATCTGGCAGGGACTGATGAGAGACGGGAGAGGCTTCGTGAGCTTCTGGAGGCAGAGGAA

AGTAGGTACTTAGCTGAGATGGAGTCACTTAAAGAAACGGTACTGGAGAAACAAGAGAAAATGAGGAAGC

AAGCAAAATTACTGGAAGAGAAGAGAGAAAAAGAAAGACAACAGCTGGTGGCTGAAAAACGAGAGCAGCA

ATTCAGAGAACAATGCGAGGAGCTTCGTGTACAGTGGATGAAGAAGCATCAGAAGGAATTGTGTGAAGAC

CGGCTGGCCCAGCTAGCTCTGAAGGAGGAATTGAAAAAGCAACAGGAGAAGGAGGAGCAGATGTTTGCAG

AGCTTTGGAAAGAGGATAGGCTGGCTAAGGAAAAGCGAGAGGCGGTGGAGATGCAGAAGTCAGCAGAGCA

GAATCGGGAAATCCTGAACGCGCTCAGTGCCCAGGTAGCGGTGCTCAGTGCTCACAAAGAGGAGGCGAAG

CGGCTGAAGGAAGAAGAGGCTCGATTGCTGGAAGAACAGCAGCAACTGCTTAAGCTAGAAAATGAACAAC

TTCAGATGGAGAAACTACAGAAACAGAAGGAATGCAGGGATACGTTGCTCAGTGCAGCACAGGACAAGAA

GAATCGTCTTAATGAAGAAAAACAAGGTGAACTTGCCCTAGAGATGAAGATCTTAGAAAAATCTCTTCAG

GAACCCCAGGAAGACACTGAGGAGAAAACAAAAAGAAAACAGGAGCTGTTCAAGGAGCAACAGACTTACC

TGGCACACCTGGCTCAACAGCTGGAGGAGGAGAAAGAGCGAGAAAAAGAAGTGGACAAACTCCTCAAAGA

AGAGGCGGCAAAGGTTTGGGCCAAGAAGGCTGAGCAAACGCGATTAGAAAAGGAGGCTAGAAAGCAGCTA

CTGAAAGATGTCCTGGATACAAGACAACTGCAGATGGAGGAGAAGTTGCAGAGAAATGCAAAGGAGCAGG

AAGAACTTGCTCAAGAGAGGAAGTTATTAGCTGAAGCAATCACAGAACTCAAACATACAGAAGAAGAAAA

ATATGCAAGAAGAGTAAAGGAAGCAAAAGAATACCAAGAGCAGCTCCGGGCTCAAATTGCCTATCGACAA

CAGGCCCGTGATGCTGAGGAAGAAGAGAAGCAGCGAGAATATGAATCGGGTCTAGCAGCAGAGAGAGCTT

ACCAAGAAAGAGTACAGGACATTCTATCAAGGCCTTGTGAGAAACTAGCCAAGACCCACCCTTTGAGAAG

AAAACTAATGTCTAACACTCAAGAAGATAAAGTACAATGA

>Emperor penguin CTIF

ATGGAGAACTCGTCGGTGGCATCGGCCTCCTCGGAGGCGGGGAGCAGCCGCTCTCAGGAGATCGAGGAGC

TGGAGCGTTTCATCGACAGCTATGTCCTAGAGTACCAGGTCCAGGGGCTGCTGACAGATAAAACGGAAGG

GGATGGCGAGAGCGAGAAGACGCAGTCCCACATCTCGCAGTGGACGGCGGATTGTAGCGAGCAGCTTGAT

GGCAGCTGTTCCCCATCCCGAGGGAAGGGCTCGTCGTCTCACGAACACAATCAGAATGGCAACAAGGAGA

GCTCCCTTGACATGCTGGGCACGGACATCTGGGCTGCCAACACCTTTGACTCCTTCAGTGGTGCGACGTG

GGACTTGCAGCCTGAAAAACTAGATTTCACCCAATTTCACAGGAAGCTGCGAAACACGTCCAAACACCCG

CTGCCTCAAATAGACAGAGAAGGGCTTGGAAAAGGGAAATATGAGGATGGTGACGGCATCAACTTGAATG

ACATAGAGAAAGTACTTCCAGTGTGGCAGGGTTACCATCCGTTGCCTCATGAAGCTGAAATTGCACACAC

CAAAAAACTGTTCAGAAGGAGGAGAAACGACCGGAGGCGACAGCAGAGACTTCCCGGTGGGAACAAGTCT

CAGCAGCACGCAGATCATCAGCAAGGTGGCACCAAACACAACAGGGACCACCAGAAACTCTACCAAGGAG

GCCAGGCCCCTCACTCCTCAGGCAGGACAGGCCACCACGGCTACAGCCAGAACCGGAGATGGCACCACAA

CCAGAAGCACTCACCCAACGACAAAGAAACGCACAGAAACGCCAAAGAGACTGAGAATTTGAAAATCGAG

GACACCTCCATCTGCACAGTGCATATTCCCGTGGAGACACACCGGGGCCCAGAGGCTGTGGAGAAGCAGT

CTCAGCAGTACATCCAGGAGTCGGAGACCAAGCGGAAAGACAGTATTCACGAGCGCATTGGGGAAAGACC

CAAGATCAATTTGCTTCAGTCTTCCAAAGACAGGCTGCGGAGGAGACTAAAAGAAAAGGACGAAGTCACG

GTGGAAACCACCAATCCTGAAAAGAACAAAATGGACAAATTAATTGAAATCCTCAACAGCATGAGGAACA

ACAGCAGTGACGTTGACTCCAAGCTCACCACCTTCATGGAGGAGGCCCAGAATTCTACCAACTCTGAGGA

GATGCTGGGGGAAATAGTCAAGACCATCTACCAGAAAGCGGTGACGGATCGCAGCTTTGCTTCCACGGCA

GCCAAGCTCTGTGACAAAATGGCCCTCTTCATGGTGGAAGGAACCAAATTCCGGAGTCTGCTCCTCAACA

TGTTGCAGAAGGATTTCACCATGCGGGAGGAGTTGCAGCAGCGGGATGTGGAGCGCTGGCTGGGGTTCAT

CACCTTTCTCTGCGAAGTCTTCGGCACCATGAGGAGCAGCACCGGAGAGCCCTTTCGAGTCCTTGTCTGC

CCCATTTATACCTGCCTCAGGGAGTTGTTGCAATCTCAGGATGTGAAGGAAGACGCTGTGCTTTGCTGCT

CCATGGAGCTGCAGAGCACCGGCCGGCTGCTGGAGGAGCAGCTGCCCGAGATGATGACGGAGCTGTTGGC

GATAGCACGCGACAAGATGCTGTGTCCTTCCGAGTCCATGCTGACGCGGTCCCTGCTGCTGGAGGTCATC

GAGCTGCACGCCAACAACTGGAACCCCCTGACGCCCACCATCACGCAGTACTACAACAAGACCATCCAAA

AACTGACGGCTTGA

>Emperor penguin DCC

ATGGAGAATAGTCTTGGATGTGTTTGGGTACCAAAGCTGGCTTTTCTCCTCTTCGGGTTTACGCTGGCGA

GCCTGCATCCTCGAGTCTCCGGTTCACAGATTAAAACTTTCACATCATTGAGGTTCTTGACTGAGCCTTC

AGATGCTGTCACCATGCGTGGAAGCAACGTGCTGTTGAACTGCATAGCAGAATCAGATCAAGGAGCCCCA

GTTATTAAATGGAAGAAAGACGCAGTCTTCTTAAACCTGGCAGTAGATGAAAGGAGGCAGCAGCTGGCCA

ACGGATCCCTCTTGATACAAAACATAGTCCACTCCAGGCACCACAAGCCAGATGAAGGTCTCTACCAGTG

TGAAGCATCTCTAGAAGGCATTGGAGCTATCATCAGTCGGACAGCTAAGGTCATGGTAGCAGGACCGCTG

AGGTTTCTTTCCCAGACAGAATCTGTCACGGCTTTTGCAGGAGACACCGTTCTTCTGAAGTGTGAAGTTG

TTGGAGAGCCCATGCCTGTGGTGCACTGGCAGCGAAACCAGGACGACTTGTTCCTGAGCCCGGCCGACAC

GTGGGTGGCTGTCCTGCCCTCCGGAGCTTTACAGATCAGCAGGATTCAGCATGGGGACAGCGGGATCTAC

AGATGCCTGGCGAAAAACCCAGCCAGTTCGAGAACTGGAAATGATGCAGAAGTCAGAGTTTTGGCAGATC

CAGGCTTGCACAGGCAGCAGTTTTTCCTTCAGCGACCGTCGAACGTGGTGGCCATGGAAGGGAAGGATGC

TGTTCTGGAGTGCTGCGTTTCTGGGTACCCCCCTCCCACCTTCACGTGGCTGCGAGGAGATGAAGTGCTC

CCTGTCAGGTCCAAAAAGTATTCGTTACTGGCTGGCAGTAACCTACTCATATCAAACGTGACTGACGATG

ATTCTGGGACATACACATGTGTAGTCACCTACAAAAACGAGAACAGCAGTGGCTCCGCAGAGCTGTCAGT

GATGGTTCCACCATGGTTTTTAATTCGCCCTTCAAATCTTTATGCCTACGAGAGTATGGATGTTGAGTTT

GAATGTGCCGTGTCCGGTAAGCCTGTTCCTACGGTGGAGTGGATCAAGAATGGAGAAGTGGTCATTCCCA

GCGACTATTTTCAGATAGTGGGTGGCAGCAACTTACGGATTCTGGGCTTGGTAAAGTCAGATGAAGGTTT

TTATCAGTGTGTAGCTGAAAATGAAGCTGGAAATGCACAGGCCAGTGCACAGCTAATCATCCCAGAGCCT

GCTGTCCCAAGTTCCAGTGTCCTCCCCTCTGCCCCCCGAGATGTGGTCCCTGTTTTGGTCTCCAGCCGAT

TTGTCCGTCTCAGCTGGCGCCCACCCGCGGAAGCCAGAGGCAGCGTCCAGACGTACACGGTCTTCTTCTC

CAGGGATGGCGTCAACAGGGAACGGGCAGTCAACACATCTCAGTCTGGGACGCTTCAACTTACCGTGGGC

AACCTGAAGCCGGAGGAGACCTACACCTTCCGAGTGGTGGCATACAACGAGTGGGGACCTGGAGAGAGCT

CACAGCCCATCAAGGTCGCCACGCAGCCGGAGCTGCAAGTTCCCGGGCCGGTGGAAAACCTGCGGGCTGT

GTCTACCTCACCTACCTCGATTCTCATCTCCTGGGATCCCCCTGCCTATGCAAATGGCCCCGTTCAAGGC

TACAGGCTCTTCTGTACGGAGACGGCGACTGGAAGAGAGCAGAACGTGGAGGTGGACGGGCTCTCCTACC

GCCTGGAGGGGCTGAAGAAGTTCACCGAGTACACCTTGCGCTTCCTCGCCTACAACCGCTACGGCCCGGG

CGTCTCCACCGAGGACGTGACGGTCACCACGCTTTCGGATGTGCCCAGCGCGATGCCTCAGAACGTCTCC

TTGGAAGTGGTTAACTCCAGGAGCATCAAAGTTAGCTGGTTGCCTCCACCACCGGGTACTCAAAATGGAT

TTATTACGGGCTATAAAATCCGACATAGAAAGACTACCCGCAGGGGTGAGATTGAAACACTGGAGCCAAA

CAACCTCTGGTACTTGTTCACAGGACTTGAGAAAGGAAGCCAGTACAGTTTCCAGGTGGCTGCCATGACA

GTGAATGGGACAGGGCCCCCCTCGGACTGGTACACAGCAGAAACACCCGAGAACGATCTCGACGAATCTC

AGGTTCCTGACCAGCCAAGCTCTCTTCATGTCAGGCCCTTGACAACAAGTATCGTCATGAGTTGGACTCC

GCCGCTGAACCCAAACATTGTCGTTCGCGGGTACATTATTGGCTATGGCGTCGGCAGTCCGTATGCTGAG

ACGGTGCGGGTGGACAGTAAACAGCGTTATTATTCCATTGAAAATTTGGAGCCCAGTTCCCACTACGTGA

TTTCCTTAAAGGCCTTCAACAACGCCGGCGAAGGGGTGCCTCTGTACGAAAGTGCCACCACCAGGTCGAT

GACAGACCCCATTGATCCATTAGAAGTTGATTTTTATCCTTTGCTTGATGATTTCCCTACCTCAGTCCCA

GATATCTCCACCCCCATGCTCCCACCAGTAGGTGTCCAGGCTGTTGCACTTACGCATGATGCAGTGAGGG

TCATCTGGGCAGACAACTCTGTCCCAAAGAACCAAAAGACTACGGAGGTTCGCTTCTACACAGTCCGATG

GAGAACGAGCTATTCGACGAGTGCTAAGTACAAGTCGGCAGATACGACCGCTCTGAGTCACACCGTGATA

GGTCTGAAGCCAAACACCATGTACGAGTTCTCCGTCATGGTCACCAAAGGTCGGCGGTCCAGCACCTGGA

GCATGACCGCGCACGCCACCACCTATGAAGCAGCTCCAACCTCTGCTCCCAAGGATTTGACAGTCATTAC

ACGGGAAGGGAAGCCCCGGGCCGTCATTGTCAGCTGGCAGCCGCCGTTGGAAGCCAATGGCAAAATTACT

GCTTACATCCTCTTCTATACTTTGGACAAGAATGCTCCAATTGACGACTGGATTATGGAGTCCATCAGCG

GCGACCGGCTCACCCACCAGATCATGGACCTCAACCTGGACACCGTCTACTACTTCAGAATCCAAGCTCG

CAACGCCAAGGGAGTCGGGCCCCTCTCCGATCCTGTTTTCTTCCGGACGCTGAAAGTCGAGCACCCTGAC

AAAATGGCTAATGACCAAGGTCGTCACGGGGATGGCTCCTACTGGCCGGTGGACACCAACCTGATTGACA

GGAGCAGTCTGAACGAGCCTCCCATCGGGCAGATGCACCCTCCCCACGGCAGCGTCACGCCCCAGAAGAA

CAGCAACCTCCTCGTCATCATCGTCGTCACCATCGGCGTCATCACCGTGGTGGTGGTGGTGGTGGTGGCC

GTCATCTGCACCAGGCGCTCCTCGGCGCAGCAGAGGAAGAAACGTGCAACCCACAGCGCTGGTAAAAGGA

AGGGCAGCCAGAAGGACCTGAGACCCCCGGATCTGTGGATACACCACGAGGAGATGGAAATGAAGAACAT

CGAGAAGCCGGCAGGCTCGGACCCTGCAGGAAGGGACTCCCCGATGCAGAGCTGCCAGGACATCACCCCC

GTCAGCCACAGCCAGTCGGAAACGCAGCTGGGCAGCAAGAGCACCCCGCAGCCCGGTCCTGAGACAGAAG

ATGTTGGAAGTAGCATGTCCACGTTAGAGCGCTCGCTTGCTGCCCGCAGAGCCACCCGTGCCAAGCTCAT

GATCCCCATGGATTCCCAACCAAACAACCCTCCTGTGGTCAGTGCCATTCCGGTGCCAACACTAGAAAGT

GCCCAGTACCCCGGGATCCTGCCGTCCCCGACCTGCGGATACCCACACCCGCAGTTCACCCTTCGGCCGG

TGCCGTTCCCGACCCTCTCTGTGGACAGGACCTTTGGAGCAGGAAGAAGTCAGTACACTGTGAGCGAAGG

ACCAGCACCACAGCAGCCGTCCTTGCTACCGCAGACGCAGCCTGAGCACTCCACCCACGAGGACGCCCCG

AGCAGAACCATCCCCACCGCCTGCGTCCGCCCTACGCACCCTCTCCGCAGCTTTGCCAACCCCTTGCTAC

CTCCGCCCATGAGTGCAATAGAACCGAAAGTCCCTTACACACCACTTCTGTCTCAAACAGGGCCTAACCT

CCCCAAGGCTCAGGTTAAAACAGCATCCCTTGGCTTGGCAGGAAAAGCCAGGTCACCTCTGCTGCCCGTC

TCGGTGCCCACGGCCCCGGAGGTCGCAGAAGAGGGCCACAAGCAGACGGAGGACTCCGCAAACGTTTATG

AGCAGGATGATCTGAGTGAACAGATGGCCAGTTTGGAGGGGCTAATGAAGCAACTCAATGCTATCACAGG

CTCAGCCTTCTAA

>Emperor penguin DYM

ATGGGAGCAAATAGCAGCAGCATCAGCGAGCTTCCAGAAAATGAGTACTTAAAAAAATTATCAGGAGCAG

AGCCCATCTCTGAGAATGACCCGTTCTGGAATCAGCTGCTGTCTTTTAGCTTTACCACTCCAACAAACAG

TGCTGACTTAAAGCTCTTGGAAGAAGCCACAATTTCAATCTGCAAGTCTTTAGTTGAGAAGAATCCTCGA

ACAGGAAACCTTGGGTCATTGATTAAAGTCTTTCTTTCCAGAACCAAAGAGTTAAAAATTTCAGCAGAAT

GTCAGAATCACCTCTTTATTTGGCAGGCTCACAATGCACTGTTTATTATTTGCTGTTTGCTGAAAGTATT

CATCAGTCGAATGTCAGAAGAGGAGCTGCAACTTCATTTTACTTACGAAGAGAAAGCACCGGGCTCATAT

GGAACAGAGTGTGAAGACCTCATAGAAGAGTTGCTGTGTTGCCTCATCCAGCTCATTGTTGAAATTCCCC

TCTTAGATATTACATACAGCATTTCCTTGGAAGCTGTGACGACTCTCATCGTCTTCCTCTCCTGCCAATT

ATTCCATAAAGAAATTCTACGAGAGAGCATCATTCACAAATACCTGATGCATGGGCGATGTCTCCCATAT

ACCAGCAGACTTGTGAAAACTTTACTATATAATTTCATTAGACAAGAAAGAAGGCCTCCTCCAGGGACCC

ATGTCTTTCAGCAGCAAACAGATGGAGGAGGACTGCTTTACGGGATCGCATCTGGGGTGGCAACTGGCCT

GTGGACAGTCTTCACACTAGGTGGGGTGGGGAGTAAACCAACGCCGCAGCTCGAGCAGTGCTCCCCGCTA

GCTAACCAGAGTCTGCTGCTTCTGCTGGTCCTGGCTAATCTGACCAATGCTCCAGATACGCCGAATCCCT

ACAGGCAAGCTATTATGTCCTTCAAGAACACACAAGATAGCACTGCTTTTTCGTCGTCACATCCACACGC

TTTCCAGATTAATTTTAACAGTTTATACACAGCTTTGTGTGAGCAGCAGAAATCTGATCAAGCAACTCTT

CTTTTATACATGCTTCTGCATCAAAACAGCAACGTACGGACGTATGTGTTGGCACGAACGGACATAGAAA

ATCTTGTTCTGCCAATTCTTGAAATTCTGTATCACGTTGAAGAAAGGAATTCACACCACGTTTACATGGC

TCTTATCATTTTGCTGATCCTTACAGAGGATGATGGCTTCAACCGATCCATTCATGAAGTGATATTGAAA

AATATCACTTGGTATGCTGAGCGTGTCTTAACAGAGATCTCACTTGGGAGTCTCCTGATACTAGTCGTGA

TAAGAACCATCCAGTACAACATGACACGGACAAGGGACAAATACCTTCATACAAATTGTCTGGCAGCCTT

AGCAAATATGTCAGCACAGTTCCGCTCACTTCATCAGTATGCTGCTCAGAGGATCATCAGTTTATTTTCT

TTGTTGTCTAAAAAACACAACAAAGTGCTGGAGCAAGCCACGCAGTCCTTAAGAGGTTCCCTCGGTTCAA

ATGACTCTCCGCTTCCTGATTATGCGCAAGATCTGAATGTGATCGAGGAAGTGATCCGAATGATGCTGGA

GATCATCAACTCCTGCCTGACAAATTCTCTTCATCACAACCCAAACTTGGTGTACGCGCTGCTTTACAAG

CGGGATCTGTTTGAGCAGTTTCGAACTCACCCCTCCTTCCAGGACATAATGCAAAATATAGATCTGGTGA

TCAGCTTTTTCAGCTCCCGATTAGAGCAAGCTGGAGCTGAGCTGTCAGTGGAGCGAGTTCTGGAAATCAT

CAAGCAAGGAGCTGTTGCTTTGCCCAAAGACAGGCTAAGAAAGTTCCCCGAGCTGAAGTTCAAGTACGTG

GAGGAGGAGCAGCCCGAGGAGTTCTTCATCCCCTACGTGTGGTCCTTGGTTTACAACTCTGCCGTGGCCC

TGTACTGGAACCCACATGACATCCAGCTCTTCACTATGGACTCTGGCTGA

>Emperor penguin DYNAP

ATGGATAACCACGCATTTGAAATGCACGGAGAGAGCATACGAAGTTCATCAAAAGAAAAAGAGTGGCCAA

AGACAGAGGAAAGAAAGGGCAGCTGGTCCCTAATGAAAGTCTTTCTAGTTTGTCTATTGGCCTGTGTTAT

CACCACCGCAATAGGAGTGCTGGCCCTGTCCTTGGTCTATGTAAAGAGCACTGCCTTTATGAAAGAGACG

GATATTAAAGACGATGGTGCATCTTCCCCAAAACCAGATGAAAAAAGTGTGGATTTCAAATTCCAGTTCC

TGAATCATCTGGAGAAATCAAAGGTACATAAGTACCCAGGTGGTGAAATTCAGTGGGCAAGATTCAGGAA

GGATGTAAAGGAATATCAAAGTGATGAAGAAATGGAATTTGGAAAAAGTATCAATAACCATCGCTCCAAA

ATGACTTTTGGAACCTTACGGATCAAAAGCAAAGGGCTTCGGGCTCCCCATTGGCATTTTAATGCCAATG

AACACGGCTACCTGCTACAGGGTACTGCCTGGATTGGAGTAATTGGTGCAGATGACAGTGTGGTTACCAC

ATACAATGTCACAGCTGGTCAAGTGATCTTCTTCCCTAGAAACACTGTGCATTGGGTAAAGAATGTAGGA

TCAGAAGACTGTGTGTTCTTGCTGTTTTTTACAACACATGAAGAACTTCAGACCTTGGATGTAGATGATG

CATTTTTCTCTACCCCAGAGGATATAGCAGCTAGAGCATTAAAGNCACAAGGAGGAGTTAACTTCATCAG

AACATTCAAGAAACAAGTAGAAGATCAAGCAGTTAACCTCCCACCAAACTTAAATGAGCTTGTACAAAAT

GCCACCTATGTGCAGTCTCCGGACAACCTTGTATGGCAGTACTTCTACAATCTCAAAGGGTCAGCAAAAT

ATCCTTTTCCAGGAGGAATCTTCCAGTGGGCTCGCTACCGCATAAATGGCACAGGACTAAATGAAACGGA

AAAAATTTTTAGTGAGTCACTGAACAAGCATGAAAATACCCTGACCTTAGCAACTCTCAGGATATTCAGC

AACAGACTGGGGCAGCCTCATTTCCACTTCAATGCTAACGAGATGGGTTATGTCATTAGCGGCTGTGGAC

AGGCTGGAGTTATTCTCTCATCTGGAGTCACCACCAGCTTCAACATTGGCATTGGAGATGTCATATTTTT

CCCTGTTGGAACCCAACATTATATCAAGAGCATATGTGATGAGGATTTGCTTTTGATTCTAGCCTACAGT

ACGGGGAACCAGCTGGAAACTCTTCGTATGAATAAATACTTCCGTGGAACAGCAGATCATATCCTTGCTC

AGCTTTTTTTCAAGAAGCAGGCTGAGTTTAAGAAGTTCTCAAATTAG

>Emperor penguin ELAC1

ATGTCGATGGATATAACTTTCCTCGGCACAGGCTCGGCATATCCCTCTCCAACAAGAGGAGCATCGGCGT

TAGTGCTTCGCAGGGAAGGAGAGTGCTGGCTCTTCGACTGCGGAGAGGGAACTCAAACACAGTTCATGAA

GAGCCATCTCAAAGCAGGCAGAGTTACCAAGATTTTCATAACTCATCTTCACGGTGACCACTTTTTTGGA

CTTCCTGGCCTGCTGTGTACACTTAGCCTCCAAAGTAGCCCTGACCCACACAAACCACCCGTTGATATTT

ATGGGCCATTAGGACTGCGAAACTTCATACGGAGGAGCATGGAGCTCTCCCACTCGCAACTTCTCTTTCC

CTACACTGTTCATGAACTGGTACCCACACGGGACCAGTGCCCCGCGGAAGAATTTAAAGAGTTTTCTTGC

TTGGACGGAGATGAGGTGTCTCCCCAGGGAGCACAAGGGAGAATACTCCAGCTGGATCCAGTAGAAAACT

CTTACTTGCTGGTTGAGGATGAGCAGCTAGTTCTGAAAGCATTTCGCCTATTTCACCGCATTCCTTCCTT

TGGCTTTGTGGTGGAAGAGAAGCCCCGGACCGGTAAACTCAATGTACAGAAACTGAAAGACCTTGGAGTT

CAACCAGGTCCTTTATATGGGAAACTGAAGGATGGAACAGCAATCGTTCTAGAAAACGGAGTAACGATTT

CTCCTTCAGACGTCTTAGAAGACCCTATTCCTGGAAGAAAGATTTGCATTTTGGGGGATTGTTCAGGGGT

GGTTGGAGATGCGGCCACGAAGCTTTGCTGTGAAGCAGATGTACTGATACATGAAGCCACGTTGGACGAT

ACCCAAGAGGAAAAGGCCAGGGAGCATGGTCACAGCACTCCAAAAATGGCATCAGAATTTGCAAAATTGT

GTAAAGTGAAGAAACTGGTTTTGACTCACTTCAGTCAGCGGTATAAACCAGCTGCTCAGAGAGGAGAGGG

AGACACGGACATCGCCGAACTGAAGAGACAGGCAGAGTCAGTGTTAGATGGTCAAGAAGTTACACTAGCT

GAGGATTTTATGACAATAGAAATTCCAATGAAAAAATAA

>Emperor penguin FECH

ATGCCGGGTGCTGGCGCAGCCAGCGCTGCCTGCTGCCTGCACCTGCTGCTGTGCCAAGCGCTCGGGACTG

GTTCGAGCGCTCAGGACCGGTTCCTAGCTGTTGTAAAAAGCAGCAGTCAACTGAGGGTCCCGGTACGATG

GAGAGGTCAGGCAACTGCAGCGGCAGTGACAGAAAGCACAAAACCTCAAATTGAGCCAGAAGTGCGGAAA

CCTAAAACAGGAATCTTGATGTTAAACATGGGAGGCCCAGAACGGCTGGATGATGTGCATGATTTTTTAC

TTCGTCTCTTCCTGGACAGAGATCTAATGACGCTTCCAGCTCAAAATAAATTAGCACCATTCATTGCCAA

ACGTCGCACACCGAAAATCCAGGAGCAGTACAGCAGGATTGGAGGTGGATCACCAATCAAGAAGTGGACA

GCGGTGCAGGGAGAAGGCATGGTGAAACTGCTGGACAGCATGTCGCCTCGCACTGCACCTCACAAATACT

ACATTGGCTTCCGGTACGTCCATCCTCTGACAGAAGAAGCAATTGAAGAGATGGAGAAAGATGGCATTGA

AAGGGCTATCGCTTTCACGCAGTACCCACAGTACAGTTGTTCTACCACAGGAAGCAGTTTAAATGCCATT

TATCGCTACTATAATAAAAAAGGGGAGAAGCCGAAGATGAAGTGGAGTATAATTGACAGATGGCCCACAC

ATCCCCTTCTTATTCAGTGCTTCACCGATCACATACAGAAGGAACTGAACCTGTTTCCACCCGACAAAAG

GAAAGATGTCATCATCCTCTTCTCGGCTCACTCGCTGCCCATGTCTGTAGTGAACCGTGGTGATCCGTAT

CCTCAGGAAGTGGGAGCTACTGTCCAGAGAGTCATGGAGAAGCTGAACTACTCCAACCCTTACAGGCTTG

TGTGGCAGTCCAAGGTTGGACCAATGCCTTGGCTTGGTCCACAGACAGATGAGGCCATTAAAGGACTGTG

CCAAAGAGGAAAGAAGAACATGTTGTTGGTCCCAATAGCATTTACAAGTGACCACATTGAAACACTTTAT

GAACTGGATATTGAGTATGCTCAAGTTTTAGCGAATGAGTGTGGAGTTGAAAATATCAGAAGAGCAGAGT

CTCTTAATGGAAATCCACTGTTCTCCAAGGCTCTGGCAGACTTGGTCTGTTCGCATATCCAGTCGAATGA

AATCTGCTCTAGGCAGTTAACCCTCTGCTGTCCGCTCTGTGAAAATCCCGTCTGCAGGGAGACAAAAGCC

TTCTTCACTAATCAACAGCTGTGA

>Emperor penguin LIPG

ATGCCCTTCGGGGAGCGCCTTGTGCCCACGAGGTCCAGCGCATCCCGAGCGGGCGCGGAGCTGGGGCTGA

AAACCGCAGACCCGCGCCGGTGCCCGGATGCCGCCGTCGCCGAGCCGCCGGAGGACAGGCGGGAGCCTGC

GCCGGCACCGAAGCCGCAGGTGATGTTCAACCTCCGGTCCTCGCCGGACGCCGAAGAGGACGGTTGCGCG

CTCGCCGTCGGCCAGCACAAGCGCTTGGAGGACTGCGAGTTCAACGCGACAGCCAAAACCTTCTTCATCA

TTCACGGCTGGACGATGAGCGGCATGTTCGAAACCTGGCTGGGCAGCTTGGTATCCGCTCTTCGGGAGAG

GGAGAAGGATGCCAACGTGGTCGTGGTGAACTGGCTTTCGCTTGCCCACCAGCTCTATACCGATGCTGTG

AACAACACGCAGATTGTTGGAAAAAGCATAGCGAGGCTGCTTGACTGGTTACAGGAGAACCCGCTCTTCC

AGCTCGAGAATGTCCACCTGATCGGGTACAGCCTGGGCGCCCACGTTGCCGGCTTTGCTGGTAACCACGT

CCATGGGACAATAGGCAGAATTACAGGCTTGGATCCGGCTGGCCCTATGTTTGAAGGGGTGGACCCTAGC

AAGCGCCTCTCCCCCGATGATGCTAACTTTGTGGATGTCCTTCACACCTACACAAGGGAAACACTAGGCG

TTAGCATTGGGATCCAGATGCCTGTAGGCCACGTTGACATCTACCCCAATGGGGGAGACTTCCAGCCTGG

CTGTGGATTAAGTGATGTCTTGGGAGCAATTGCTTATGGGACAATTGGTGAAGTTGTTAAATGCGAACAC

GAGCGGTCTGTGCACCTCTTCGTGGACTCCCTTGTGAACCAAGATAAACAAAGCTTCGCGTTTCAATGTA

CCGATTCCAGTCGCTTCAAGAAGGGCATCTGCCTGAGCTGCCGGAAGAACCGCTGCAACGGCATCGGCTA

CAACGCCAGGAAAACACAGAACAAAAGAAACAGCAAGATGTACTTAAAAACAAGAGCTGACATGCCGTTC

AAAGTCTACCATTATCAGATGAAAATGCATGTCTTCAGCTACAAAAGCTTGGGAGAGGCTGATCCCACTT

TCTCCGTCACCCTTCATGGCACCAATGGAGACTCTGAACCCCTCTCTTTAGAAATGCTTGATCAAATTGG

CCTAAATGCTACTAACACTTTCCTGGTCTATACTGAAGACGACATGGGTGAACTTTTAAAAATAAAGCTC

ACCTGGGAGGGAACATCTCAGTCATGGTACGATCTGTGGAAAGAGCTGAAGAGCTACTGGTATCGGCCTG

CAAAGTCTTCCCAGGAACTGCATATCAGACGTATACGTGTGAAATCTGGGGAAACGCAACAGAGGTTTGC

TTTCTGCGTGGAGGATTCCCAGCTGACCAGTATATCTCCTGGTAAAGAGCTCTGGTTTGTGAAGTGCACA

GAGGAATGGCAAAAAAGATCTGTCTCAAATTTGCTCTGA

>Emperor penguin MAPK4

ATGGCAGAGAAGTGCGACTGTATCGCCAGCATGTACGGGTATGACCTGGGCTGTCGCTTCATTAATTTTC

GCCCCTTGGGCTTCGGGGCCAATGGGCTGGTGCTGTCAGCCCTCGACAGCAAGAGCTGCCGCAAAGTGGC

GGTGAAGAAGATCACCATCAGCGATGCGCGGAGCATGAAGCACGCTTTCCGGGAGATCAAAATCATCCGC

CGCCTGGACCACGATAACATCGTGAAGGTGTACGAGGTGTTGGGGCCGAAGGGGACCAACCTGCGCGGGG

ATTTTTTCAAGTTTAACATGGTGTACATCGTCCAGGAGTACATGGAGACGGATCTGGCGCGGCTGCTGGA

GCAGGGGAAGCTTGCCGAGGAGCACGCCAAGCTCTTCATGTACCAGCTGCTGCGGGGGCTGAAGTACATC

CACTCGGCCAACGTCCTCCACCGTGATCTCAAGCCGGCCAATATTTTCATCAGCACAGAGGACCTGGTGC

TGAAGATCGGCGACTTTGGGCTGGCCAGAATCGTGGATCAGCATTACTCGCACAAGGGTTACCTTTCCGA

AGGCTTAGTAACAAAATGGTATCGCTCCCCCCGCCTCCTCCTCTCACCAAACAACTACACCAAAGCCATC

GATATGTGGGCGGCCGGCTGCATCCTGGCCGAGATGCTAACGGGAAGGATGCTCTTCGCTGGGGGTCACG

AGCTGGAACAGATGCAGCTTATTCTGGAGACGATCCCCGTTATCCACGAGGAAGACAAAGAGGAGTTGCT

CAAAGTGATGCCCACGTTCATCAACAGCACCTGGGAAGTGAGGAAGCCGCTGCGCAAGCTGCTCCCCGAA

GTGGACAGTGAAGCTATTGATTTTCTGGAGAAAATACTGACGTTTAACCCTATGGATCGATTAACGGCTG

AGATGGGTCTGCAGCATCCTTACATGAGTCCGTATTCCTGCCCCGAGGATGAACCAGTGTCTCAGCATCC

GTTCCGGATTGAGGATGAGATTGATGATATTTTACTGATGGAAGCCAACCAGAGCCAGATGTCTAACTGG

GACAGGTATCACGTAAGCCTCTCCTCTGATTTGGAATGGAGACACGATAAATACCACGACATGGATGAGG

TTCAGCGGGACCCCCGGGCCGGGTCTGAATCCATCGCTGAAGAAGCACAAGTTGATCCGCGGAAATACTC

ACAAAGCAGCTCGGAGAGGTTCTTGGAGCTATCCCACTCATCCATGGACCGAGTATTTGATGCCGATTGT

GGGAAATCGTGTGATTATAAAGTGGGGTCACCTTCCTACTTGGACAAATTGCTGTGGAGAGACAATAAGC

CCCATCATTACTCAGAGCCCAAGCTAATTTTAGATTTATCCCACTGGAAAAGAGCAACCATAGCACCCGC

AGCTGAGCTATCGCTGGAAGAAGAACCATCCAACCTCTTTCTGGAGATCGCTCAGTGGGTAAAGAGCACG

CAGGTGGGTCTCGAGTGTCCCAGTCCTCTTCCGGAGCTTCAGGAACGGAGCCTGCCATCTTCTCCTCACC

ATCTCCACAAAGAACCCACAGAGGTGAACAGTGAAACAGACCCTGAGTTTGACTTGGACGTCTTCATCTC

CAGGGCGCTGAAACTTTGCACAAAACCCGAGGATCTTCCAGACAACAAGCTCAATGACATCAACGGGGCC

TGCATATCTGAGCACCCCGGTGAGATTGTACAAACAGAGGTGTACCAGAAAGAGCGATGGTGA

>Emperor penguin MBD2

ATGGAGAAGCAGGGCAGGATGGACTGCCCCGCGCTGCCCCCGGGCTGGAAGAAGGAAGAGGTGATCCGCA

AGTCGGGCCTCAGCGCCGGCAAGAGCGATGTCTACTACTTCAGTCCAAGTGGTAAGAAGTTCAGAAGCAA

GCCTCAGTTGGCAAGATACCTGGGAAACACTGTTGATCTCAGCAGTTTTGACTTCAGAACGGGAAAGATG

ATGCCCAGTAAATTGCAGAAGAACAAACAGAGACTAAGGAATGATTCTCTCAATCAAAATAAGGGAAAAC

CAGACTTAAATACAACTTTACCAATCAGACAAACTGCATCAATTTTCAAACAACCAGTCACCAAAGTTAC

CAATCATCCTAGTAACAAAGTAAGATCTGATCCACAGCGAGTGACAGAGCAGCCACGACAGCTTTTCTGG

GAGAAGAGGCTACAAGGCCTCAGCGCATCGGATGTCAGTGAACAAATCATAAAATCCATGGAGCTACCTA

AGGGTCTTCAAGGAGTTGGCCCAGGTAACAATGACGATACCCTGTTATCAGCTGTTGCTAGTGCTTTGCA

CACCAGTTCTGCACCCATCACGGGGCAGCTCTCTGCAGCTGTTGAGAAGAACCCAGCTGTCTGGCTTAAT

ACATCTCAACCCCTCTGCAAAGCTTTCATAGTCACAGATGATGACATTAGAAAACAAGAAGAGCGGGTGC

AACAAGTGCGCAAAAAACTGGAGGAAGCACTAATGGCAGACATTTTGTCACGAGCGGCTGATACAACAAA

AGATATAGATGTAGAAATGGATAATGGAGATGAAGCATAA

>Emperor penguin ME2

ATGTTCTCCCGACTAAGAGTAGCTGCCACTCCCTGTGTGATGGCACGTCGCAGCGCACATACGAAAGAAA

AAGGCAAGCCACTGATGTTAAACCCACGAACAAACAAGGGTATGGCCTTTACATTACATGAACGACAGAT

GCTTGGGTTGCAAGGACTTCTACCTCCTAAAATAGAGACACAAGACATTCAAGCCTTACGCTTTCATAAG

AATTTGGCAAAAATGACTGACCCCTTGGAAAAGTATATCTACATAATGGGAATCCAAGAGAGAAATGAAA

AATTATTCTATAGGGTATTACAAGATGATATTGAGCGGTTAATGCCAATTGTATACACACCAACAGTAGG

CCTTGCCTGCTCCCAGTATGGACACATCTTCAGGAGACCAAAAGGATTATTTATTTCCATCTCAGACAGA

GGCCATATAAGGTCAATTGTGAACAACTGGCCAGAGAATGACGTTAAGGCTGTTGTCGTCACTGATGGAG

AAAGAATATTGGGTCTTGGAGACCTAGGTGTGTATGGGATGGGAATTCCCGTAGGAAAACTGTGTCTGTA

TACAGCCTGTGCAGGAATACATCCAGATAAATGCTTGCCTGTGTGCATCGACGTTGGAACTGATAATACA

ACACTCTTAAAAGATCCATTTTATATGGGCCTATACCAAAAAAGGGATCGCTCACAGGTCTATGATGACC

TAATTGATGAATTTATGGAAGCCATTACAGACAGGTATGGCCAGAACACCCTTATCCAATTTGAAGACTT

TGGAAACCATAATGCTTTTCGGTTTTTAAGAAAATACAGAGAGAAATATTGTACCTTCAATGACGATATT

CAAGGGACAGCTTCAGTGGCCTTGGCAGGACTGCTGGCAGCACAGAAAGCCACTGGTAAACCACTTGCAG

AGCAGAAAGTGCTGTTCCTTGGAGCAGGAGAGGCCGCCCTGGGAATTGCAAACCTCATTGTTATGGCTAT

GATGGAAAGCGGTGTTTCTGCTGAGGAAGCCTACAGGAGAATATGGATGTTTGACAAATATGGTTTACTG

GTTCAGGGGCGAGAACAAAAGGTAGATTCCAATCAAGAACCATTTACACATCAGGCTCCAGAGCAGATAC

CAAAGACATTTGGAGAGGCAGTGAATGTACTTCGGCCTTCAGCTATCATTGGAGTTGCAGGAGCTGGACG

TCTCTTCTCTCAGGATGTGATCAAAGCAATGGCCTCTATCAATGAGCGACCCATAATATTTGCACTAAGT

AACCCTACAGTGAAAGCTGAATGCACAGCAGAGGAAGCATATACATTGACAGAGGGCCGTTGCTTGTTTG

CCAGTGGCAGTCCCTTCGAGCTGGTGACTCTGAAAGATGGAAGAACCTTCAAACCAGGCCAAGGAAACAA

CGCTTATATTTTTCCAGGCGTGGCTCTCGCTGTGATCCTCAGCAGTGTTCGACATATTAGTGATAAGGTT

TTCCTAGAGGCTGCTAAGGCATTGACAGAACAGTTGACCGATGAAGAACTTGCACAAGGAAGACTTTATC

CTCCACTGTCTAATATCAGGGAAGTTTCTATTTATATTGCTGTCAAGGTTATGGAATTTTTGTACGCAAA

CAACATGGCTTTCCATTACCCTGAACCTGCTGACAAGAACCGTTACATTCGATCAAAGGTTTGGACCTAC

GAATACGAATCCTTCATGCCAGATGTGTATGACTGGCCTGAATCTAAGGTTCACTAA

>Emperor penguin MEX3C

ATGNNNNGTTGCAAAATAAAAGCACTAAGGGCCAAGACAAATACTTACATTAAGACCCCCGTTCGTGGAG

AAGAACCCATCTTTGTTGTCACTGGACGAAAAGAAGACGTAGCCATGGCCAAAAGGGAAATTCTCTCAGC

TGCTGAACACTTCTCCATGATCAGAGCGTCACGCAACAAGAACGGTCCTGCCCTGGGAGGCTTGCCATGT

ACCCCCAACCTGCCGGGTCAGACGACGGTCCAAGTCAGGGTGCCTTACCGTGTAGTTGGGCTGGTGGTCG

GACCGAAAGGAGCTACAATCAAAAGAATTCAGCAGCAGACCCATACCTACATAGTCACTCCCAGCAGAGA

CAAGGAGCCTGTCTTCGAAGTTACGGGAATGCCTGAAAACGTCGACCGTGCGCGCGAGGAGATCGAGATG

CATATAGCCATGCGTACCGGGAACTACATTGAGCTGAACGAAGAGAACGATTTCCATTACAACGGTACGG

ATGTGAGCTTTGAAGGAGGCACTCTCGGATCTGCATGGCTTGCTTCTAATCCTGTCCCTCCTAGCCGCAC

CAGAATGATTTCTAATTATAGAAATGACAGCTCCAGCTCCTTGGGAAGTGGCTCCACAGATTCCTATTTT

GGAAGCAATAGATTGGCTGACTTCAGCCCAACGAGTCCGTTCAGCACAGGCAACTTCTGGTTTGGAGAAA

CGCTGCCTTCGGTGGGCACGGAAGACCTTGCGGTCGACTCTCCCGCGTATGACTCCTTACCAGCGCCTTC

CCAAACCATTTGGACCCCTTTTGAACCTGTAAACCCACTCTCTGGCTTTGGCAGCGACCCTGCTAGTAAT

GCCAAGCCTCAGCGCCGAGGGAGCCAGCCATCTACCCCTCGCCTGTCGCCCACATTTCCGGAAGGTCTGG

ATCACCCGCTGGCTAGGAGAGTGCGGAGCGACCCACCTAGCACCGGCCACCAAGCCGGCCTTCCCATATA

CATCCCCGCTTTCTCCAACGGTACCAACAGCTATTCCTCTTCCAACGGCGGCTCCACGTCCAGCTCGCCC

CCCGAGTCGAGACGGAAGCACGACTGCGTGATCTGCTTCGAGAGCGAAGTCATTGCGGCCCTGGTCCCCT

GCGGCCACAATCTCTTCTGCATGGAGTGTGCCAACAAAATCTGTGAAAAGGAAACGCCATCGTGTCCCGT

TTGCCAGACAGCTGTTACTCAGGCAATCCAAATTCACTCTTAA

>Emperor penguin MYO5B

ATGGACGCTTTGGCTGGGCTGTACGGGTCCACCTTTGGGATTTCCTCTCGGAGGCTTAAGGGTGGCCTGG

ACTTCGGAGATGCTAAAAAGTACACTAGGGTTTGGATTCCTGACCCTGATGAAGTTTGGAGATCGGCAGA

AATTATCAAGGATTACAAAGAGGGAGATAAAAGCCTTCATCTGAAACTTGAAGATGAAACTCTCTACGAA

TATCCTGTTGACCTCCAAGGAAATGAGCTGCCTTTCCTACGCAATCCGGATATCCTGGTGGGAGAGAATG

ACCTGACGGCCCTGAGCTACCTGCACGAGCCCGCGGTCCTCCACAACCTCAAAGTCAGGTTCCTCGAGTC

CAACCACATCTACACATACTGTGGTATTGTACTTGTTGCCATCAATCCATATGAGCAGCTGCCAATCTAC

GAACAAGATGTCATCTACGCGTACAGTGGCCAAAACATGGGGGATATGGATCCTCACATCTTTGCGGTGG

CAGAGGAAGCCTATAAGCAGATGGCCAGGGATGAGAAGAACCAGTCCATCATCGTGAGCGGGGAGTCGGG

TGCTGGAAAGACGGTCTCTGCCAAATATGCCATGCGATTCTTTGCAACCGTCGGTGGTTCTGCCAGCGAG

ACCAACATCGAAGCCAAAGTCCTCGCGTCGAGCCCAATTATGGAGGCGATTGGAAATGCTAAAACAACAA

GGAATGACAACAGTAGTCGCTTTGGGAAATACATTCAGATCGGCTTTGATAAAAGATACCACATCATTGG

TGCCAACATGAGGACGTATCTGTTGGAAAAATCACGAGTTGTATTCCAGGCAGAGGATGAGCGCAACTAC

CACATTTTCTATCAGCTCTGTGCCTCGGCGAGTCTTCCAGAATTCAAAGACCTTGGACTAACATGTGCCG

AAGACTTTTTCTACACTTCTCAGGGAGGTGACACATCTATCGATGGCGTGGACGATGCTGATGACTTTGA

GAAAACCAGGCACGCCTTCACCCTGCTTGGAGTGAAGGAGTCTCATCAGATGACCATTTTTAGGATAATT

GCTGCCATTCTGCACCTAGGGAACTTGGAAATCCAAGCGGAACGAGACGGCGATGCCTGTAGCATATCGA

GCGAGGATGAGCACTTGAACAACTTCTGTGGCTTGCTGGGCGTTGAGCACAGCCAGATGCAGCACTGGCT

TTGCCACTGCAAGCTCGTTACCACGGCCGAGACCTACGTGAAGAACATGTCCGTGCAGCAAGTGGTGAAT

GCCAGGAACGCTCTGGCCAAGCACATCTACGCCCAGCTCTTCAACTGGATCGTGCAGCACATCAACAAGG

CCCTGCACACCACCGTCAAGCAGCACTCCTTCATCGGCGTGCTCGATATCTACGGGTTTGAAACTTTTGA

AGTGAATAGCTTTGAACAGTTTTGTATCAACTACGCCAATGAAAAGCTCCAGCAGCAGTTCAACTCGCAC

GTGTTTAAGCTGGAACAAGAAGAGTACATGAAGGAGGGAATCCCTTGGACTCTCATAGACTTCTACGATA

ACCAGCCCTGCATAGACCTTATAGAGGCGAAACTTGGTATCTTGGACCTACTGGATGAAGAGTGCAAGGT

TCCCAAAGGCACTGACCAGAACTGGGCGCAGAAGCTGTACGACCGGCACGCCAGCAGCCAGCACTTCCAG

AAGCCTCGCATGTCCAACACCTCCTTCATCATCCTGCACTTCGCCGATAAGGTGGAGTACCAGAGTGAGG

GATTTCTGGAGAAGAACAGGGACACCGTGTACGAGGAACAGATCAACATCCTGAAAGCCAGCAAGTATCA

GATGGTAGCAGACTTATTCCAAGATGAGAAGGATGCTGCACCCACCACTTCCATGGGAAAGGGAACATCC

AAAATCAGCGTCCGTTCTGCCAGACCGGTGATCAAAGCTGCCAATAAGGAGCACAAGAAAACGGTGGGGC

ACCAGTTCCGCAACTCGCTGCATTTGCTCATGGAGACTCTGAACGCCACCACCCCGCACTACGTGCGCTG

CATCAAGCCGAACGACGAGAAGCTCCCCTTTAAATTTGATCCGAAGAGAGCGGTGCAGCAGCTGAGAGCT

TGTGGAGTGCTGGAGACCATCCGCATCAGTGCAGCTGGCTTCCCGTCCAGATGGTCCTACCACGACTTTT

TCAATAGGTATCGTGTTCTTATGAAAAAGAGAGACCTCTCTAAGAATGACAAGAAGCAGATCTGTCAGAC

CCTGTTGGAAGACCTCATTAAGGATCCAGACAAGTTCCAGTTTGGACGTACCAAGATCTTTTTCCGTGCA

GGCCAGGTGGCATATCTGGAGAAACTGCGAGCAGATAAGTTCAGAGCTGCCACAATCATGATTCAGAAGA

CGGTGCGGGGCTGGCTGCAGAGGGTCAAGTACAAAAGGCTGAGACAAGCTGCAATCGTCATCCAGCGCTA

CACGCGTGGGCGCCTGGCGCGGAGGCTTGCCGAGCACCTGAGGAGGACGAGAGCTGCCATCGTCTTCCAG

AAGCAGTACCGAATGCTGAGGATCTTCCGAGCTTTCCAGAGAGTCCGCAGTGCGACCATCACCATTCAGG

CTTTTGCTCGGGGCATGTTTGTCAGGAGGATTTATCACAAGATCCTTGCGGAGCACAAAGCCACCATCCT

CCAGAAGTTTGCCCGTGGCTGGCTGGCCCGCACTCGCTTCCGCCGGGTCAGGGGTGCCACCATCGTCCTG

CAGTGCTACTACCGGCGCATGAAGGCCAGGCAGGAGCTGAAGGCGCTGAAGATCGAGGCCCGCTCAGCAC

AGCACCTGAAGAAGCTCAACATTGGCATGGAGAACAAGGTGGTCCAGCTTCAAAGGAAGATCGATGAGCA

GAACAAGGAATACAAACTTCTGAACGAGCAGCTCTCTACGCTCACATCTGCCCACTCCTCCGAGGTGGAA

AAGCTGAAGAAGGAACTGGTGCAATATCAGCAGAGCCATCAGGGTGATGGCAACCAGCTTGTCAGCTTGC

AAGAAGAGATGGAGCACCTCCGGCTGGAGCTTGAAAAAGCTCATGGTGAGAGGAAGGTTGTGGAAGACAG

CTACGTTAAGGAGAAAGACCTACTGAGAAAGCGCATATCCGACTTGGAAGAAGAAAATGCTCTCCTGAAG

CAGGAAAAAGAGGAGCTTAACAGCAGGATCCTGTGTCAATCTAAAGATGAATTTGCACAAAACACAGTTG

AGGAAAATATTCAGATGAAGAAAGAGCTGGAAGAAGAGAGGTCTCGTTATCAGAACCTGGTAAAAGAGTT

TTCGAGGCTGGAGCAGAGATATGACAACTTGCGGGATGAAATGACTATTATAAAGCAAGCACCGGGGCAC

AGAAGAAACCCATCCAACCAGAGCAGTTTGGAGTCTGATTCCAATTATCCATCCATATCAACCTCTGAGA

TAGGAGACACCGAGGATGTAATACAGCAAGTGGAGGAGGTCGGGACGGAGAAAGCCGCCATGGACATGAC

CCTCTTCCTAAAGCTACAGAAGCGAGTGAGGGAGCTCGAGCAGGAGAGGAAGAAGCTGCAAACCCAGCTG

GAGAAAAAGGAGCAAGAGAGCAAGAAATCCCAGGTAATTGAAACGAAGACTGAAGTGACTTTGGACCATG

AAGATTTTGCATACAACAGTCTGAAGAGGCAAGAGCTAGAGTCGGAGAACAAGAAGCTGAAAAATGAACT

TAATGAGCTGAGGAAGGCTATCGCAGACCGAGCAACCCAGAACAACTCGTCCAACGATATTCAGGACAGT

TATAACCTGTTACTGAATCAGCTGAAATCGGCCAACGAGGAGTTGGAAGTGCGGAAGGAAGAGGTGCTCA

TTCTGAGGACGCAGATTATGAAGGCAGCCCAGCAAAAAGAGACAGGCAAAAGCATGGAAAGCATCACCAC

CAACGCCAGCTGGCCGAACAGTGACAAGCACATTGATCAGGAGGACGCGATTGAAGCCTATCAGGGGATG

TGTGAGACGAACCGCAAGACTGAGGACTGGGGGTATCTCAATGAAGATGGAGAGCTCGGCTTGGCTTATC

AAGGTTTAAAGCAAGTTGCCAGGTTACTGGAAGCACAGCTCCAAGATCAGAGAAGAGAGCATGAGGAGGA

GGTAGAAGCTCTGAAAAACCAGGTGGATGCAATGAAAGAAGAGATGGAGAAACAGCAGCAGGCTTTCTTG

CAGACCCTGCAACTGTCTCCGGAGGCCCAGGTGGAGTTCGGACTTCAGCAAGAAATTACACGTCTCACCA

ATGAAAATCTGGATCTTAAAGAATTGCTAGAGAAGTTGGAAAAGAATGAAAAGAAGCTGAAGAAGCAGCT

GAAGATTTACATGAAGAAGGTCCAAGATTTTGAAGCATCCCAAGCCACGGTACCGGCGGAGAGGAGGCGG

CACGAGCGTAACATGCAAGTTGCTGTCCAGAGAAAGGAGAAAGATTTTCAGGGCATGTTGGAATATTATA

AAGAAGATGAGCCACTCCTCATCCGAAACCTCATTACAGATCTCAAGCCCCAGGCAGTGTCTGCTACTGT

TCCCTGCCTTCCCGCCTACATCCTCTACATGTGCATCAGACACGCGGATTACATCAACGATGACCAGAAA

GTGCACTCCTTGCTCACCTCCACCATCAACGGCATTAAGAAAGTGCTGAAAAAACACAACGATGACTTTC

AGATGACGTCGTTTTGGCTGGCGAATACGTGTCGCCTCCTGCACTGTTTAAAGCAGTACAGCGGAGACGC

GGGTTTCATGACACAAAACACGCCTAAGCAGAACGAGCACTGTCTGAAGAACTTCGACCTGACCGAGTAC

CGCCAGGTGCTGAGCCACCTCTCCATCCAGATCTACCAGCAGCTCATTAAGATAGCAGAGGGCATACTCC

AACCCATGATCGTGTCTGCGGTGCTGGAAAATGAGAGCATCCAGGGGCTTTCTGGTGTCAAACCGATGGG

CTACAGGAACCGCTCCTCCAGCATGGCTGATGGGGACAGCTCCTACAGCTTAGACGCAATCATTCGCCAG

CTGAACACATTCTACAGCATCATGTGTGACCAGGGTCTGGACCCAGAGATCGTGCAGCAGGTCTTCAAGC

AGCTCTTCTACATGATCAACGCCGTCGCCCTGAACAACCTCCTGCTGAGGAAGGATGTCTGCTCGTGGAG

CACGGGCATGCAGCTAAGGTTTAACATAAGCCAGCTGGAGGAATGGCTGCGTGGGAAGAACCTGCAGCAG

AGTGGAGCGGCACAAACTTTGGAGCCCTTGATTCAGGCAGCACAGCTGCTGCAGCTGAAAAAGAAAACCT

CGGAAGATGCCGAGGCCATCTGCTCCTTGTGCACATCGCTCACGACACAGCAGATTGTAAAGATACTTAA

TCTCTACACTCCTGTGAATGAGTTTGAAGAACGTGTGACAGTAGCTTTCATACGAGACATACAGACGCAC

TTGCAAGAGCGAAATGACCCTCCGCAGCTGCTGTTAGACTTCAAGCACATGTTCCCAGTTTTGTTTCCGT

TCAACCCATCCTCCATAACCATGGACTCGATTCATCTCCCTGCTTCTCTCAACTTGGGATTTCTCAATAA

AGTCTGA

>Emperor penguin NARS

ATGCCTGCGGCGGGCGGGAAGGGGGCAAGGACCGCTAATGGAGCCCATGGCGCGTTGGGAGCAGTTAACT

TCCAGAAGCGCTGGCCGCCTCCTGCCCGCTTCCCAAGGGGCTGGAGGAACCCCTTCTCGTGTATGGCCGG

CAGTCTAGTGGCCGTGTCCCAGCGTGATCGCAGGTGGAATCTAAGACAAGAGCTGTATGTTTCTGAACGA

GAGGGCAATGATTCCACTGGTGATGGGACGCAAAAGAAACCATTCAAGACTGTTTTAAAGGCTTTGATGA

CAGCAGGAAAGGAACCATTTCCTACTATTTATGTGGATTCACAAAAAGAAAACGAGAGATGGGCCATTAT

TTCAAAGTCACAGATGAAAAATGTCAAAAAACTGTGGCACAGGGAACAAATGAAGAATGAAGCTAAGGAG

AAGAAGGAGGCGGAAGATCTCTTGAGGAGAGAGAAGAACCTGGAGGAAGCTAAGAAGGTTGTTATCAAGA

ATGATCCTAGTCTTCCAGAGCCAAAATGTGTAAAGATTGATGCTCTGGAAGCTTACAGAGGCCAGAGAGT

GAAGGTTTTTGGCTGGATTCACAGATTACGGAGGCAAGGAAAAAATTTGATGTTCATTGTTTTGAGAGAT

GGCACAGGTTTTCTTCAGTGTGTCCTTTCAGATGAACTGTGTCAGTGTTACAGCGGGCTAATTCTCTCTA

CGGAGAGCAGTGTTGCAGTGTATGGTATGCTGAACCTTGTTCCTGAAGGCAAGCAGGCTCCAGGAGGCCA

TGAGCTGAACTGTGATTACTGGGAGCTTATTGGTCTGGCCCCAGCAGGAGGGGCTGACAATCTACTCAAT

GAGGATTCGGAGGTTGATGTGCAACTTAACAACAGGCATATGATGATTCGAGGCGAGAATATGTCAAAAA

TCTTCAAGGTGCGGTCCATGGTAGTACAGGCCTTCAGGGATCATTTCTTTGCCAATGGATATTATGAAGT

CACACCGCCAACTTTAGTCCAGACACAGGTGGAAGGAGGCTCAACCCTATTCAAACTGGATTATTTTGGT

GAAGAGGCGTACTTAACACAATCGTCCCAGCTCTATCTGGAGACCTGCATTCCAGCGTTAGGAGATGTTT

TCTGTGTTGCTCAGTCATACAGAGCTGAGCAATCCAGGACCCGCAGACACTTGGCAGAATACACTCACAT

TGAAGCTGAATGTCCTTTTATAAGTTTTGAGGATTTATTGGACCGTCTGGAGAGCTTGGTTTGTGATGTA

GTAGACAGAGTCTTGAAATCACCTGCATCAAGCTTACTATATGACCTAAACCCGGGCTTCAAGCCCCCTA

AACGTCCTTTCCGACGAATGAACTATACTGAAGCCATTGAGTGGTTAAAGGAACATGATGTGAAGAAGGA

AGATGGCACTTACTATGAGTTTGGGGAAGATATTCCTGAAGCTCCTGAGAGATTGATGACAGACACCATC

AATGAGCCGATCTTGTTGTGCCGATTTCCTGCAGAGATAAAGTCTTTCTATATGCAGCGCTGTCATGACG

ATTCCCGGCTTACTGAATCTGTTGATGTGTTGATGCCTAATGTTGGTGAAATTGTTGGAGGCTCTATGCG

TATCTGGGACAGTGAGGAGCTACTCGAAGGCTATAAGAGAGAGGGCATTGATCCCACGCCGTACTACTGG

TATACTGATCAGAGAAAATATGGTACGTGCCCTCATGGTGGATATGGTTTGGGATTAGAACGGTTCCTAA

CCTGGATTCTGAATAGACACCATATCCGAGATGTCTGTCTCTATCCACGCTTTGTCCAGCGCTGCAAACC

TTAG

>Emperor penguin ONECUT2

ATGNNNNCCATGGTGTCCAGCATGGCCTCGCTGCTGGACGGCGCCGCCGAGTACCGCCCCGAGCTCTCCA

TCCCGCTGCACCACGCCATGAGCATGCCCTGCGAGTCCTCGCCGCCCGGCATGGGCATGAGCAGCACCTA

CACCACCCTGACGCCACTCCAGCCCCTGCCGCCCATCTCCACCGTCTCCGACAAGTTCCACCACCCGCAC

GCCCACCCGCACGCCCACCACCACCACCACCACCAGCGCCTCTCGGGCAACGTCAGCGGCAGCTTCGCCC

TCATGCGGGACGAGCGCGGGCTGCCCGCCGTCAACAACCTCTACGGGCCCTACAAGGAGATGCCCGGCAT

GGGGCAGAGCCTCTCGCCGCTGGGCAACGGGCTGGGCCCCCTCCACAACGCCCAGCAGGGCCTCCACGGC

TACGGGCCGCCCGGCCACGAGAAGATGCTCAGCCCCAACTTCGACGCCCACGCGGCCATGCTGGCGCGGG

GGGACCAGCACCTCTCCCGGGGGCTCGGGACGCCCCCCGCCCTGATGCCCCACCTGAACGGCATGCACCC

CCCCGCGCACCCNGGCCACCCGCCGCCCCACGGGCCCGCGCTGCCCGCCGGCCGGGAGCGGCCGCCCTCC

TCCTCCTCCGGCTCGCAGGTGAGCAGCTCGGGGCAGCTGGAGGAGATCAACACCAAAGAAGTGGCACAAA

GGATCACGGCGGAGCTGAAGCGCTACAGCATCCCGCAGGCCATCTTCGCCCAGCGGGTGCTGTGCCGCTC

TCAGGGGACCCTCTCGGACTTGCTGCGGAACCCTAAGCCCTGGAGTAAACTCAAGTCCGGCCGGGAGACC

TTCCGGAGGATGTGGAAGTGGCTGCAGGAGCCGGAGTTCCAGAGGATGTCGGCCCTGCGGCTGGCAGCCT

GCAAACGCAAAGAGCAAGAGCCGAACAAAGAGCGGAACAACTCCCAGAAGAAATCTCGCCTGGTTTTCAC

GGACCTCCAGCGCAGAACGCTTTTCGCCATCTTCAAGGAGAACAAGCGCCCCTCCAAAGAAATGCAGATC

ACCATCTCCCAGCAGCTGGGCCTGGAGCTCACCACCGTCAGCAACTTCTTCATGAACGCCCGGCGGCGCA

GCCTGGAGAAGTGGCAGGACGACCTGAGCTCCGGGGGCTCCTCCTCGGCCCCCAGCACCTGTACCAAAGC

GTGA

>Emperor penguin POLI

ATGCCAGATGCTACCCAATCATCTGCCGCAGTATTAGCATACGGTTGGGAGCGCAAGTCAGACAAAGGCG

CAGGCCACAGCAAGTCTGTATCGGCAAGACGCGCCGCGTGCAGAGCGATTGTGCACGTTGACTTGGACTG

CTTTTATGCACAAGTAGAAATGATCCGTAATCCTGAATTAAGAGACAAGCCTTTAGGCGTGCAACAGAAA

TACATCGTAGTTACCTGTAACTATGAAGCCAGAAAACGTGGAGTTAAGAAAATGATGTCTGTCAAGGATG

CTAAAGAGAAGTGTCCTCAACTGATACTGGTTAATGGAGAAGATCTAACGCCATACAGGGAAATGTCATA

CAAGGTCACAGAGCTGTTGGGGGAATTTTGTTCGCTGGTGGAGAGGCTCGGGTTCGATGAAAATTTTGTG

GATATCACAGAGATTGTAGAGAAAAGACTAAACGAGCTACAGCAAAGTGGATGTTCCAGAGTCTGTGTCT

CTGGCCACGTGTACAACAACCAAGCTATCAATTTGCAGGATACAACGCATGTAAGACTAGTTATTGGATC

TCAGATTGCAGAAGAGTTCAGGGAAGCCATACATGCGAGACTGGGCCTCACAGGCTGTGCAGGAGTGGCC

TCTAACAAATTACTGTCTAAACTCGTATCTGGGACCTTTAAACCAAATCAGCAAACAGTTCTTCTGCCTG

AAAGCTCTCAAGATCTAATACGCAGCCTTGATCACATCCAGAAAGTGCCTGGCATTGGCTACAAAACTGC

CAAACGTCTTGAAACACTGGGTGTTAGGAACGTGTGTGATCTCCAAGCGTTTCCGTCTGCTGTGTTAGAG

AAGGAACTAGGTGTTTCTATTGCGCAGCGTATCCAAAAACTCAGCTACGGAGAAGATGACGCCCCTGTGA

TGCCATCAGGCCCTCCTCAGTCCTTTAGTGATGAAGATTCCTTTAAAAAATGTTCATCAGAAGTGGAAGT

TAAAGAGAAAATTGAAGAACTGCTTCCTAACCTACTAGACAGAGTGTACACAGATGGAAGACAACCCCAC

ACAGTAAGATTGACCATACGCCGGTTCTCCTCAACCAATAAATGGTTTAATCGGGAAAGTCGTCAGTGTC
[truncated: 653,133 more chars]
