# Supplementary material for: Evolution of the functionally conserved DCC gene in birds
Source: Sci Rep. 2017 Feb 27;7:42029. doi: 10.1038/srep42029 (PMC5327406; doi:10.1038/srep42029)
Supplement: Supplementary Dataset 3 [file srep42029-s4.doc]

**Evolution of the functionally conserved *DCC*gene in birds.**

**Cedric Patthey, Yong Guang Tong^1^, Christine Mary Tait^1^ and Sara Ivy Wilson***

Umeå Center for Molecular Medicine, Umeå University, 901-87 Umeå, Sweden.

**^1^** equal contribution

*corresponding author: sara.wilson@umu.se

**Supplementary data set 3: Sequence verification of the plasmids used for probes in the *in situ* hybridisation.**

**Supplementary data set 3: Sequence verification of the plasmids used for probes in the *in situ* hybridisation.**

In situ plasmids were in a PGEM-T, pBluescript or pCRII-Topo vector. Plasmids were sequenced at Starseq (StarSEQ GmbH, Germany) using a M13 forward primer. The data below is raw reads from the sequencing and contains part of the host plasmid sequences and does not represent the whole insert sequence.

| duck *DCC* |
| --- |
| GGTAGCTAGTCGCATGCTCCGGCCGCCATGGCCGCGGGATTGCCCAAGGACAAACCTGTAACAGATAAATTTGGGAAGGTACTGAAGATAGAAAACGTGTCTGCAGCTGATGAAGGAACCTACCAGTGCACAGCAAGCAACCCCGTGGGCAGAGCAAAACATGAATTCCATGTTCATGTGGAAGAGCCTCCTCGGTGGATTAAGGAGCCCAGAGGTGGTGTTTACAGCTTAGGAGAGAATCTTTTGCTGCTTTGTGAGGCCATCGGCAACCCAGAGCCAACCATTCAGTGGAAACTTAACGGGATGCCCATTGATAGTAGGACCTTCAGAGGGAGAATCTCTGATGGGGAGCTCAGCCTTATCAACCTTCAGCTTCAGGACACTGCTGTGTACCATTGTGAGGCCAGTAACAAGCATGGCACCCTCCTTGCTAGCGCCAACGTGAATGTCCTCAATATTGCTCCCTTAATCCTGACTTCAGATGGTGAAAACTATGCTGCAGTTGTTGGTTACAGTGCCTTCCTGCACTGCAAAATTTTTGCCTCGCCTGCAGCAGATGTTAGATGGACTAAGGATGATAGCATAGAGCCACTCTCAGCATTACGCTATGAATTAAATAAAAATGGCACCCTAGAAATCAAAGAAACAAAGAAGGAAGATTCGGGATCATACGCTTGCTGGGCTGCAAATTCTGTTGGAAAAAGAGCAATCACAGCAAATCTGGATATAAGAGATGCTACAAAAATTGTCATTACTCCGAAGAACCCCCAAGTGTTGAAATCACATTCAATTTTACTGAAATGTCAGTCTGAGTATGATTCACACTTGAAAAACAGCTTCAAATTATCCTGGAGGAAAGATGGATATGAGCTGTCAGTCAGCAGCATACAAGACAGCAGATAGTTATGGACATGGATACGCTGTTCATATCAAATGTGATGTTGGAGACCAAGGCGTTTACACGTGTGTGGCTAGCACTTCTCTTGACAGTGTCACTGCCAAAACGCGGTTAATTGTTCTGATGTTCTGACCCACCAGAGACTGCAGCTCTCAGACATCAAAACGAGTGTTCGACTGTCCTCAAAGCTGGGGCAGCACACAGCCTGGTATGAATTCGATATAGAGTTTGGAAGAAGCCGTGATCACTATGCGCCGCTGCAGTCGACATTGGAAGCTCATCGCGGTGCATGCATAGCTTGGAG |

| mouse *Dcc* |
| --- |
| GGGACGCTCTGATGCATGCTCGAGCGGCCGCCAGTGTGATGGATATCTGCAGAATTCGCCCTTTTCTGACTGCCCTTCCTCTTGCTCACACTGTGTGTGGCCCGTTTCTTCCTCTGCTGCGCTGAAGAACGCCGGGTGCAAATCACTGCTACAATCACCACGACCAACACAGTGAGAACACCAACGGTGACCACGGTTATTACAAGCAGGTTGCTGTTCTTCTGAGGAGTGACACTGCCATGCGGGGGGTGCATCTGTCCAATGGGTGGTTCGTTGAGAGTGCTTCTATCAATCAAGTTAGTATCAACTGGCCAATGACCCCCATCTCCATGACGACCTTGGTCATTAGCCATTTTGTCAGGGTGTTCCACTTTCAAAGTCCTGAAGAGGATGGGATCAGAGAGGGGCCCTACTCCTTTCACATTCCGTGCTTGAATTCGAAAGTAATACATCGTGTCAAGGCTGAGATCCATGATCTGATGGGTAAGCCGGTCACCGCTGATAGTTTCCATAATCCAATCATCTATTGGTATATTCTTGTCCAGTGTATAAAACAAGATATAAGCAGTGATTTTCCCATTGGCTTCCAAGGGAGGCTGCCAGCTCACAATGACAGCACGAGGCTTCCCCTCTCGAGTAATGACTGTCAGATCCTTGGGAGCAGAAGTTGGGGCAGCTTCGTAGGTGGTGGCGTGTGCAGCCATGCTCCACGTGCTTGACCTTCTGTTCTTGGTCACCATGACTGAGAACTCGTACATGGTATTGGGCTTGAGGCCAGTCGCTGTGTAACTGAGAGATGTTGTGTCTTCTGACTTGTACTTGGCACTGGCAGAAAAGCTGGTCCTCCACCGAACGGTGTACAGTCGCACATCGGATGTTTTCTGGTTCTTCGGGACAGAGTTGTCTGCCCAGCTGACTCTCACAGCCTCATGGGTAAGAGCTACAGCCTGTACACCTACTGGTGGGAGCATGGGGGTGGAGACATCTGGGCCCGAGTGGGGAAATCATCAGCAAGGGCGAATTCAGCACACTGGCGGCGTACTAGTTGATCGAGCTCGTACAGCTGGATGCATAGCTGAGTATTCTTATAGTGTCACTTAATAGGCCTGCCGTTATCATGGTCATAGCCTGGTTCATGTGTTGAATGTATCGCTCAAGTGCACAGACATACCGAGCTGAGCTTAG |

| duck *Neogenin* |
| --- |
| GGCAGCTAGTCGCATGCTCCGGCCGCCATGGCGGCCGCGGGAATTCGATTATTGGCCCTGTCTGAATGGGACATGGATGTGCCAATCGGCCACGGGTTGACCTGGTGATGGAGATAGCTGCGAGTTGGAGAAGCGAGGCTGCCGGAGTGGAAATGATGGTGAGGGTTATCGAGTGAATGGATGGGATGAGCACTAATCACAGGCTGAGGTGGCTGAGAATCAAAAGGCATCATCATCTTGGGCCTCATTCCCCTTCTTCCTGCCAGCGTGGACATGCTATCCTCTGACTCATGCCCTCTGTAGGAATTCCGCCTTTGATGGATATTGCTGTCCATGGAATTATCAACTGGGGTGATGTCTTGGGAGTTGCGAGGGATTGGGGTATCTGTCATGATTGGATTGGGATCTGGAGATTTATCAATGGGTTTTAGCTCCAGTCTTTCATGATGGATCCAAAGGTCAGGAGGCTTGACATCTTTGGAGTTTCCTTTGTACTTGTGGGACCCGTTCACTGATTTGCAGGCAGCTCGTTTCTTTTTCTGGTGAGAAGTGGTACGACGAGTGCAGAAGACAGCAACTATCACCACGATCACAATGGTGATGACTCCAACAGATACGATGATCACAAGGAGCATGTTGCTATCCAAGGGAGAAGTAGGGCTTCCATGGGGACTGTTACTGCCACCGAGTGAATCACTAGTGAATTCGCGGCCGCCTGCAGGTCGACCATATGGGAGAGCTCCCAACGCGTTGGATGCATAGCTTGAGTATTCTATAGTGTCACCTAAATAGCTTGGCGTAATCATGGTCATAGCTGTTTCCTGTGTGAAATTGTTATCCGCTCACAATTCCACACAACATACGAGCCGGAAGCATAAAGTGTAAAGCCTGGGGTGCCTAATGAGTGAGCTAACTCACATTAATTGCGTTGCGCTCACTGCCCGCTTTCCAGTCGGGAAACCTGTCGTGCCAGCTGCATTAATGAATCGGCCAACGCGCGGGGAGAGGCGGTTTGCGTATTGGCGCTCTTCCGCTTCCTCGCTCACTGACTCGCTGCGCTCGGTCGTTCGGCTGCGCGAGCGGTATCAGCCTCACTCCAAGCGGTATACGTTATCCACAGAATCAGGGGATACGCAAGAAAGAACATGTTGAGCCAAAGGCTAGCCAAGGTCAGACCGGTTAAAAGGCCGCGATTGCCTTGGCCGATTTTTTTCA |

| chicken *Neogenin* |
| --- |
| GAACTGTCGCATGCTCCGGCCGCCATGGCCGCGGGATTGGACACAGCTTTTCCAGCTCATTGAAGGTCTTGAACGGGGCACGGAGTACAACTTCCGGATAGCTGCCATGACTGTGAATGGCACTGGGCCAGCTACTGACTGGGTGTCAGCAGAAACATTTGAGAGCGATCTGGATGAAAGCCGTGTTCCTGAAGTTCCGAGCTCCTTACACGTCCGTCCTCTTGTCACCAGTATTGTGGTGAGCTGGACTCCACCTGAGAACCAAAACATCGTGGTGATAGGCTATGTCATAGGGTAAGGCATCGGCAGTCCCCCCCCTCCCACCACCGAGGTAAATAACAACCGCTGTTTATTCCCTTTTAAACCGAATGATTGAGGTACCTTTAAATAATAAACTGGTATCCACACTTATAACAGGGTGATTGCATTC |

| mouse *Neogenin* |
| --- |
| CTTTGTCAACACACCGGAATTAGGCTATGAGCATGATACGTCAAGCTCGGAATAACCTCCATTAAGGGAACCAAAGCTGAGCTCACGCGGTTGCGCGCGCTGTTGTGATGCATTAGTCATCATAGTCTGCAGGTGGTCATCTCTTGTCAGCTCATCTGGTCATAGCTACTCTCGAGTCTCAGCATCCTGGTGGTCTCTGTACTTCAGGGGCACTCGGAACAACCACTGGCATAGGAGGCCGGCTCTTCCTAACGTCCCGATGGAGGCTGTTTTCACTGAGTGAATGTGATGGTTCAGAGCTTGCTGGGACAGTAATGGTGTGCTTGGCAGTGCAGGATCATAGAGAGGAGGTCCTGGGGGTGGGATTGCTGGCACAGCGAAGCTCTTCAGAGGGTGGGAAGGGCGGACATGGGCTGTGGGAAGACTCTGGCTGAGTCTTCCTCTTGGGAGCTGGCCAAGTAAGAGGAGCTAGTAGCACCCTCAGGGTCCTGATGGTCAGTGCAGCACGTCTGAGACGAGGACGCTGGCATGGTGTCCGTGCTGGGGGTATTTCGAACAGATTGCTGAGGTGGCTGAGAGTCAAAGGGCATCATCATTTTTGGTCTCATTCCCCTCCTTCCAGCCAGTGTAGACATGCTGTCCTCTGACTCATGCCCTCTGTATGAATTCCGCCTTTGATGGATATTGCTATCCATGGAATTGTCCACTGGTGTGATATCTTGAGAGTTTCGAGGGATTGGAGTATCAGTCATGACAGGGTTAGGATCTGGAGACTTGTCAATAGGCTTCAACTCTAGTCTCTCGTGATGGATCCATAGGTCTGGAGGCTTCACATCTTTGCAATTGCCCTTGTACTTATGGGAGCCATTCACTGATTTGCACGCAGCTCGTTTCTTCTTCTGGTGAGAGGTGGTGCGCCGGGTACAAAAGACAGCAATGACCACAACCACCACGATAGTGATGACGCCAACAGAGACAATGATGACCAGCAGCATGTTGCTGTCCAGAGGGGAGGTGGGGCTCCCGTGAGGGCTGTTGCTGCCACTCATTGGAGGTTTGTAGTCAGATCCCAGGTCTGGTAGTCGGCTTCCTTTTCCTGCTGACCCTAAGGCTTGGTCATTAGGCATTTTATCAGAGGAGTCCGCTTTAGGTGTTCTGAACTGTACAGCTCAGAGCATGGCCACTCGCTC |
